# Enantioselective Synthesis of α‐Arylated Allene Ketones Through Sequential Bismuth(V)‐mediated Arylation and Organocatalytic Protonation

**DOI:** 10.1002/anie.202517136

**Published:** 2025-10-07

**Authors:** Kun Zhu, Yuli Sun, Yunhan Ma, Zugen Wu, Yixin Lu

**Affiliations:** ^1^ Department of Chemistry National University of Singapore Singapore 117543 Singapore; ^2^ Energy and Environmental Nanotech Platform National University of Singapore (Suzhou) Research Institute Suzhou 215123 China

**Keywords:** Asymmetric Arylation, Bismuth, Chiral Allenones, Enantioselectivity, Organocatalysis

## Abstract

Triarylbismuth(V)‐mediated arylation represents an important approach for synthesizing a wide range of α‐arylated ketones and enol derivatives. Since the seminal work by Barton and colleagues in the 1980s, these C─C bond‐forming transformations have been extensively explored. Despite significant progress, asymmetric variants of these reactions have yet to be developed. In this study, we document a sequential reaction consisting of bismuth‐mediated α‐arylation of allene ketones and an enantioselective protonation of α‐arylated alkynyl ketones, leveraging the isomerization between allenyl and alkynyl intermediates. Our approach relies on a reversible/irreversible isomerization sequence comprising three distinct stages. The process initiates with the generation of enolates through reversible isomerization, followed by oxidative arylation and a subsequent enantioselective, irreversible isomerization to yield α‐arylated allenones. Both experimental results and theoretical studies support our mechanistic proposal. The sequential bismuth(V)‐mediated arylation and enantioselective proton transfer is conceptually significant, as coupling bismuth chemistry with asymmetric catalysis may open new avenues for organobismuth(V) chemistry in enantioselective transformations and extend its utility in synthetic organic chemistry.

## Graphical abstract

We report an asymmetric synthesis of α‐arylated allenones achieved via sequential bismuth(V)‐mediated arylation and organocatalytic protonation. This method furnishes enantioenriched α‐arylated allenones with broad functional‐group tolerance. Mechanistic studies support a cascade sequence involving enolate formation, arly migration, and irreversible enantioselective isomerization. This report represents the first example whereby organobismuth(V)‐mediated arylation is coupled with asymmetric catalysis.

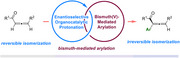

## Introduction

**Figure 1:**
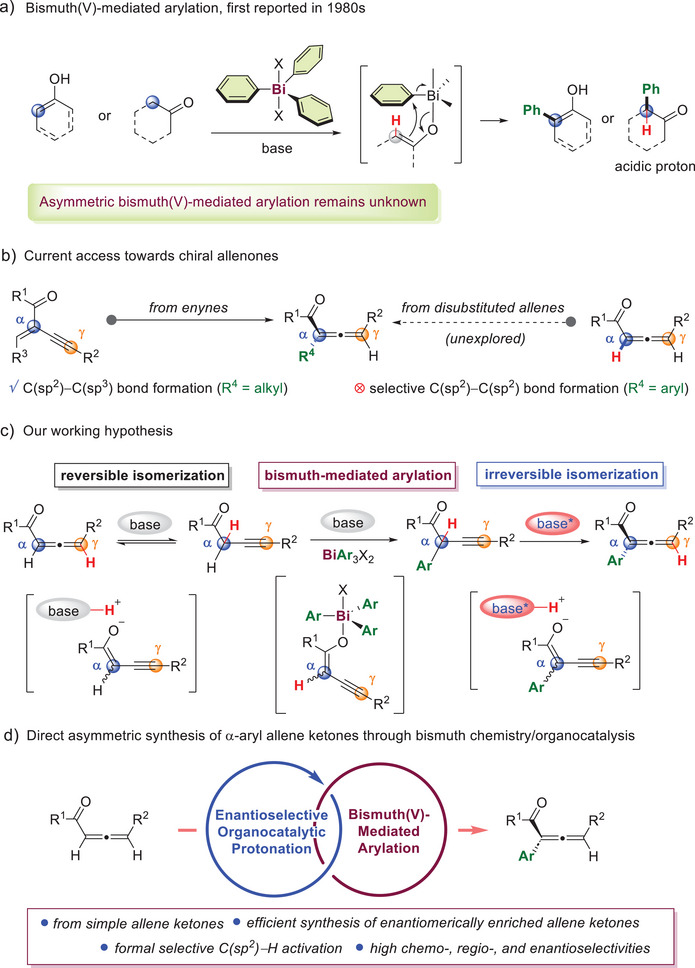
a) Bismuth(V)‐mediated arylation, first reported in 1980s. b) Current access towards chiral allenones c) Our working hypothesis. d) Direct asymmetric synthesis of α‐aryl allene ketones through bismuth chemistry/organocatalysis (this work).

Despite being located among toxic heavy metals in the periodic table, bismuth and bismuth‐containing compounds demonstrate exceptional stability and low toxicity.^[^ ^1^, ^2^ ^]^ This has resulted in a worldwide focus on producing bismuth as a safer alternative to hazardous elements like lead in various alloys.^[^ ^3^, ^4^ ^]^ Due to the soft Lewis acidity of Bi(III) and oxidizing nature of Bi(V), as well as the lability of the Bi(V)─C(sp^2^) bond, bismuth catalysis has found broad applications in carbonyl and π–bond activation,^[^ ^5^, ^6^ ^]^ oxidation,^[^ ^7^, ^8^ ^]^ as well as arylation.^[^ ^9^, ^10^, ^11^, ^12^ ^]^ Recently, bismuth redox catalysis has been harnessed as a powerful main group platform for organic synthesis, by leveraging the redox behaviors of bismuth species in various oxidation states.^[^ ^13^, ^14^, ^15^, ^16^, ^17^, ^18^, ^19^, ^20^ ^]^ In the realm of bismuth chemistry, arylation reactions through the cleavage of the bismuth–carbon bond in a bismuth(V) species represent one of the most classic reactions in the field. Ever since the pioneering reports by Barton and co‐workers in 1980s,^[^ ^21^, ^22^, ^23^ ^]^ such bismuth‐promoted arylation reactions have been extensively applied to a broad range of substrates, including phenols, ketones, β‐diketones, ketoesters, substrates bearing an acidic α‐proton, as well as α,β‐unsaturated carbonyls (Figure 1a).^[^ ^24^, ^25^, ^26^, ^27^, ^28^ ^]^ Very recently, through the use of in situ generated modular bismacylic organobismuth(V) arylating agents,^[^ ^29^ ^]^ Ball and co‐workers achieved selective C─H arylation of phenols, naphthols, and diketones.^[^ ^30^, ^31^, ^32^, ^33^ ^]^ Up to date, all the arylation reactions through the utilization of arylbismuth(V) reagents have produced only racemic products, and enantioselective version of such arylation remains unknown. We recognize that achieving a successful asymmetric process is challenging, primarily due to difficulties in controlling enantiomeric outcomes during the arly migration step and in preserving the stereochemical integrity at the newly formed stereogenic center at the α‐position of a carbonyl group. We therefore set out a goal to develop a bismuth‐based catalytic method to achieve asymmetric α‐arylation of ketone compounds. To address these challenges, we considered merging an additional asymmetric catalytic transformation to achieve asymmetric induction, alongside the organobismuth(V)‐mediated arylation reaction.

Due to their distinctive structural features and properties, allenes represent an important class of structural motifs that are found in natural products and therapeutic agents, and they are also tremendously useful in synthetic organic chemistry and materials science.^[^ ^34^, ^35^, ^36^, ^37^, ^38^, ^39^, ^40^, ^41^, ^42^, ^43^, ^44^, ^45^ ^]^ In particular, allene ketones^[^ ^46^ ^]^ are a class of compounds that find wide applications in organic synthesis.^[^ ^47^, ^48^, ^49^, ^50^, ^51^ ^]^ When the asymmetric synthesis of chiral allene ketones is concerned, there are only a handful of reports (Figure 1b).^[^ ^52^, ^53^, ^54^, ^55^, ^56^, ^57^ ^]^ These examples mostly required the use of enynes as substrates and are also somewhat limited in reaction scope. Given our continuous interest in axial chirality and allene chemistry, and in an effort to unleash the synthetic potential of arylbismuth(V)‐triggered arylation reaction, we questioned the feasibility of utilizing bismuth‐mediated arylation reaction for the straightforward asymmetric construction of α‐arylated allene ketones.^[^ ^58^ ^]^ Hinging on the nature of acidic proton at the α‐position of an alkynyl isomer or at the γ‐position of an allenyl isomer, isomerization processes between an alkyne and an allene are anticipated.^[^ ^59^, ^60^, ^61^ ^]^ Notably, Takemoto and co‐workers reported that allenyl esters, particularly those bearing an α‑alkyl substituent, exhibit a strong kinetic preference that drives essentially enantioselective irreversible isomerization away from the alkynyl form.^[^ ^62^, ^63^ ^]^ In our working hypothesis, we propose that a base‐induced reversible isomerization of an allene ketone leads to the formation of an alkynyl enolate intermediate, which undergoes bismuth(V)‐mediated arylation to yield the α‐arylated alkynyl ketone. Given the strong kinetic preference towards the formation of α‐substituted allenyl isomer, the subsequent isomerization is expected to be irreversible, yielding the final α‐arylated allene ketone products. By carefully selecting a chiral base catalyst to influence stereochemically controlled isomerization through an enantioselective protonation process, an effective catalytic asymmetric synthesis of α‐arylated allene ketones may be achieved. (Figure 1c). Herein, we document the first direct enantioselective synthesis of α‐arylated allene ketones by merging triarylbismuth(V)‐mediated arylation and enantioselective organocatalytic protonation (Figure 1d).

## Results and Discussion

**Figure 2:**
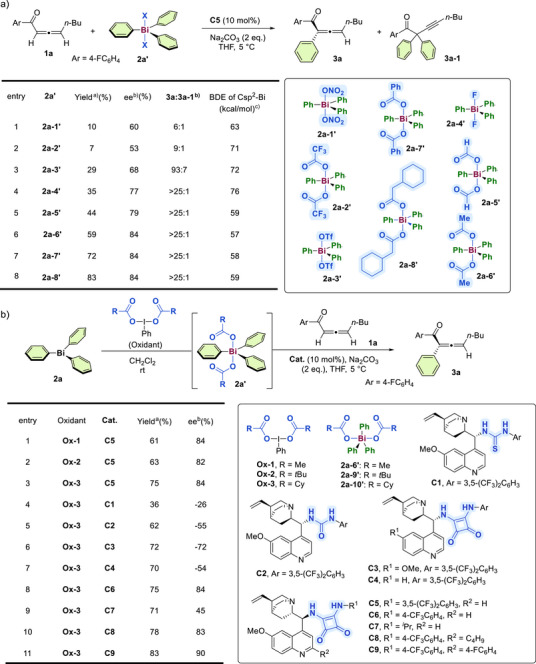
Screening the reaction conditions. a) Evaluation of triarylbismuth reagents. Reaction conditions: **1a** (0.06 mmol), **2a’** (0.05 mmol), **C5** (10 mol%), Na_2_CO_3_ (2 equiv.), THF (1 mL) at 5 °C, 16 h. ^a)^ Determined by crude ^1^H NMR analysis with mesitylene as an internal standard. ^b)^Determined by HPLC. ^c)^Bond dissociation energies of C(sp^2^)‐Bi bond were calculated via Gaussian 16. b) Modified arylation process directly from triphenylbismuth. Reaction conditions: **1a** (0.075 mmol), **2a’** (0.05 mmol), **Cat** (10 mol%), Na_2_CO_3_ (2 equiv.), THF (1 mL) at 5 °C, 16 h. **2a’** was prepared in situ, directly from **2a** (0.05 mmol) with **Ox** (0.055 mmol) in CH_2_Cl_2_ (0.5 mL), subsequently used after evaporation of the solvent. Cy = cyclohexyl.

We began our investigation by employing readily available allene ketone **1a** as a model substrate for the organocatalytic enantioselective α‐arylation reaction with triarylbismuth(V) reagents **2a’** (Figure 2a). Cinchona alkaloids are powerful organic catalysts widely used to promote asymmetric transformations. Given their potential utility in multicatalytic systems and proton‐transfer processes,^[^ ^64^ ^]^ we selected cinchona alkaloid–derived squaramides and evaluated their catalytic effects in our reactions. Upon treatment with BiPh_3_(NO_3_)_2_ (**2a‐1′**) in the presence of epi‐quinine–derived squaramide **C5**, the desired product **3a** was obtained in a 10% yield and with 60% ee, with a ratio of α‐arylated allene ketone (**3a**) to α,α‐diarylated alkyne ketone (**3a‐1**) of 6:1 (entry 1). A similar problem of low yield and overfunctionalization was observed when arylating bismuth agents containing hard nucleophilic counter anions were used (entries 2–4). After systematically investigating the effect of the anion, we found that triarylbismuth(V) reagents with carboxylate anions led to the formation of **3a** in moderate to good yields, with excellent selectivity for monoarylation over diarylation (**3a**:**3a‐1** = >25:1; entries 5–8). We hypothesized that the observed improvement in yield could be attributed to the bond dissociation energy (BDE) of the C(sp^2^)–Bi bond.^[^ ^65^, ^66^ ^]^ Arly migration is more feasible when the C(sp^2^)–Bi bond is relatively weaker, and the BDE is partially modulated by the nature of the counter anion. With weakly coordinating anions, the C(sp^2^)─Bi bond is strengthened, which leads to less efficient aryl transfer. However, their weak coordination also gives greater flexibility in ligand exchange,^[^ ^67^ ^]^ thus contributing to the formation of bis‐arylated products (**3a’**). To make our method more practical and versatile, we next focused on developing a more concise arylation method starting directly from triarylbismuth (Figure 2b). Upon treatment with a hypervalent iodine(III) reagent, triphenylbismuth(V) **2a’** could be readily prepared and used directly without further purification.^[^ ^68^ ^]^ Different hypervalent iodine reagents were employed, with **Ox‐3** proving to be the most effective, affording **3a** in a 75% yield (Figure 2b, entries 1–3). Subsequently, various cinchona alkaloid derivatives bearing different hydrogen‐bond donating moieties were examined. While thiourea and urea were less effective (entries 4 and 5), the squaramide motif efficiently induced asymmetry (entries 6 and 7). Further structural modifications of *epi*‐quinine‐derived squaramides led to improved enantiomeric excesses (entries 8–10; see Scheme S4 in the Supporting Information for detailed optimization). Under the optimal reaction conditions, treatment of triarylbismuth with the hypervalent iodine(III) reagent **Ox‐3** yielded triphenylbismuth(V) **2a’** in situ, which further reacted with allene ketone **1a** in the presence of **C9** to furnish the desired α‐arylated allene ketone **3a** in 83% yield with 90% ee (entry 11).

**Figure 3:**
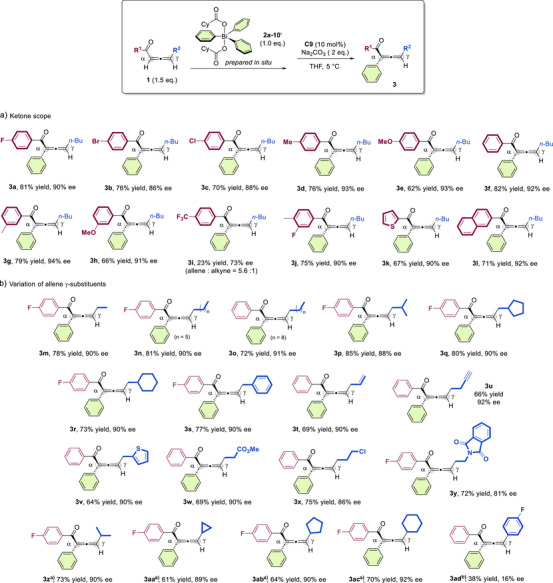
Scope of the allene ketones. Reaction conditions: **1** (0.15 mmol), **2a‐10′** (0.1 mmol), **C9** (10 mol%), Na_2_CO_3_ (2 equiv.), THF (2 mL) at 5 °C, 16 h. **2a‐10′** was prepared in situ, directly from **2a** (0.1 mmol) with iodobenzene dicyclohexanecarboxylate (0.11 mmol) in CH_2_Cl_2_ (1 mL), subsequently used after evaporation of the solvent. The yields refer to isolated yields. ee values were determined by HPLC analysis on a chiral stationary phase. ^a)^The reaction was performed at 5 °C for 24 h. ^b)^The reaction was performed at room temperature for 24 h, and no product was observed at 5 °C.

With the optimized reaction conditions established, the scope of the reaction was subsequently investigated, beginning with an evaluation of various allene aryl ketones (Figure 3). Arene moieties bearing either a halogen atom or an electron‐donating group at the *para‐*position of the phenyl ring were well‐tolerated (**3a**–**3e**), so were the aryl ketone substrates with a *meta‐*substituted phenyl substituent (**3** **g**, **3** **h**). In contrast, the electron‐deficient CF_3_ substituent resulted in a significant decrease in yield and eantioselectivity (**3i**), which can be attributed to the normally irreversible isomerization becoming reversible, thereby compromising product stability. Disubstituted phenyl ketone was also found to be suitable (**3j**). Additionally, allene ketone substrates bearing a 2‐thiophenyl or 2‐naphthyl group were compatible, forming products with high ee values (**3k**, **3l**). Allene ketones bearing different γ‐alkyl substituents were next examined. The reaction worked well for the substrates with linear alkyl groups of different length, and consistently high yields and excellent ee values were attainable (**3m**−**3o**). Allene ketones containing branched/cyclic alkyl substituents at the γ‐position were also good substrates (**3p**−**3r**). Moreover, allene substrates bearing a benzyl (**3s**), a terminal alkenyl (**3t**), an alkynyl (**3u**), or a heterocyclic moiety (**3v**) at the γ‐position were all well‐tolerated. Interestingly, functionalized substrates containing an ester (**3w**), a chloride (**3x**), or a terminal phthalimidyl‐protected amine group (**3y**) were also applicable to the reaction. Moreover, this reaction was equally effective for substrates with secondary alkyl groups, whether cyclic or acyclic (**3z**−**3ac**), affording the desired products with high enantioselectivities. However, the allene substrate bearing a γ‑aryl substituent showed reduced reactivity, forming the desired product with poor enantioselectivity (**3ad**).

**Figure 4:**
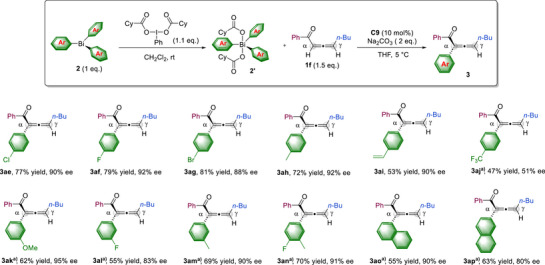
Scope of triarylbismuth. Reaction conditions: **1f** (0.15 mmol), **2′** (0.1 mmol), **C9** (10 mol%), Na_2_CO_3_ (2 equiv.), THF (2 mL) at 5 °C, 16 h. **2′** was prepared in situ, directly from **2** (0.1 mmol) with iodobenzene dicyclohexanecarboxylate (0.11 mmol) in CH_2_Cl_2_ (1 mL), subsequently used after evaporation of the solvent. The yields refer to isolated yields. ee values were determined by HPLC analysis on a chiral stationary phase. ^a)^ **2’** was prepared with iodobenzene diacetate (0.11 mmol).

We have also investigated the compatibility of various triarylbismuth(V) reagents under our standard reaction conditions (Figure 4). A diverse range of arenes bearing a *para*‐substituted halogen atom (**3ae**–**3ag**), an electron‐donating methyl group (**3ah**), or a vinyl substituent (**3ai**) were found to be suitable. The use of the corresponding triarylbismuth starting materials afforded the desired products in good yields with excellent enantiomeric excesses. In contrast, the electron‐deficient arene bearing a CF_3_ substituent resulted in decreased yield and enantioselectivity (**3aj)**. In addition, *meta*‐ or disubstituted arenes (**3ak**–**3an**) were applicable, affording α‐arylated allene ketones with very good enantiomeric excesses. The use of triarylbismuth reagents bearing a 1‐ or 2‐naphthyl substituent (**3ao**, **3ap**) afforded the corresponding arylation products with good enantioselectivities, although with somewhat reduced chemical yields.

**Figure 5:**
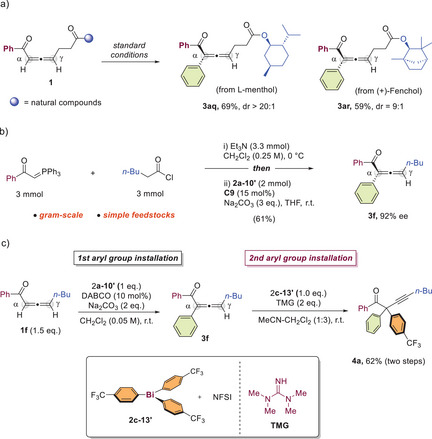
Reaction extension. a) Late‐stage functionalization of complex natural products. b) Gram‐scale, de novo synthesis of α‐arylated allene ketones. c) Tunable sequential arylation of allene ketones enabling unsymmetrical α‐diarylated alkynes.

The synthetic suitability of our method was next demonstrated by performing the reaction on substrates derived from natural products, including L‐menthol and (+)‐fenchol. All reactions proceeded in good yields with high diastereoselectivities. (Figure 5a). The α‐arylated allene ketones exhibit unique reactivity and hold significant potential for advancing allene chemistry, albeit with limited synthetic accessibility. Thus, starting from commercially available phosphonium ylide and acyl chloride, we performed straightforward synthesis of α‐arylated allene ketones on gram scale, obtaining **3f** in moderate yield with 92% ee (Figure 5b). Furthermore, we found that **3f** derived from our reaction could be activated as a nucleophile under stronger basic conditions. With the treatment of 1,1,3,3‐tetramethylguanidine (TMG) and arylbismuth reagent **2c‐13′**, a distinct aryl group was readily introduced at the α‐position of **3f**, resulting in unsymmetrical α‐diarylated alkyne product **4a** with 62% yield over two steps (Figure 5c).

**Figure 6:**
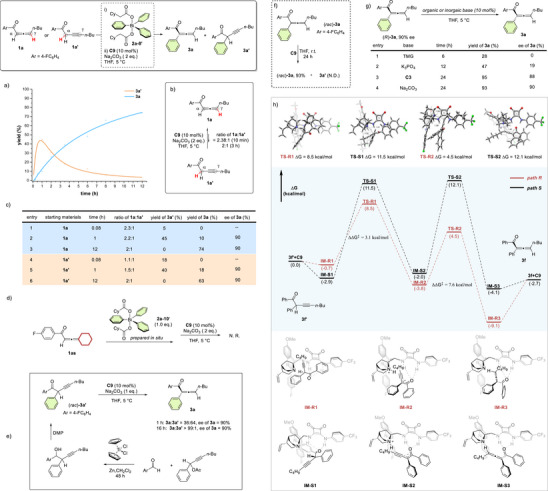
Reaction mechanisms. a) Monitoring reaction of **1a** and **2a‐8′** under standard condition. b) Equilibrium of reversible isomerization between **1a** and **1a’** under basic conditions. c) Control experiment of different starting materials. d) Examination for the necessity of an acidic proton at the γ‐position e) Monitoring irreversible isomerization of (*rac*)‐**3a’**. DMP = Dess‐Martin periodinane. f) Evaluation of irreversible proton transfer g) Evaluation for the stability of (*R*)‐**3a** under basic conditions h) Energy profiles along path *R* and *S*. The relative free energies are given in kcal mol^−1^.

To gain mechanistic insight, a number of experiments were carried out. We began by monitoring the reaction between allenyl substrate **1a** and triphenylbismuth reagent **2a‐8′** under the standard conditions (Figure 6a). As expected, the racemic alkynyl intermediate **3a’** was initially generated and gradually accumulated over the course of the reaction. The enantioselective, irreversible isomerization then required 12 h to fully convert **3a’** into the desired allenyl isomer **3a**. To further elucidate the relationship between reversible isomerization and bismuth‐mediated α‐arylation, we conducted additional control experiments. Treatment of **1a** with the chiral squaramide catalyst **C9** at 5 °C led to reversible isomerization, reaching equilibrium with an isomeric ratio of 2:1 (Figure 6b). Since both **1a** and its isomer **1a’** can form the active enolate intermediate during the reaction, we subjected each to the reaction separately (Figure 6c). The results indicated that **1a’** displayed higher reactivity in the arylation step, as evidenced by the yield of **3a’** and the isomeric ratio of **1a** to **1a’** at 5 min (entry 1 versus 4). Notably, similar enantioselectivities were observed at both the initial (entry 2 versus 5) and final stages (entry 3 versus 6) of the reaction, suggesting that both **1a** and **1a’** underwent the same arylation and subsequent isomerization processes. Moreover, the necessity of an acidic proton at the γ‐position of allene ketone was examined (Figure 6d). The 1,3,3‐trisubstituted allenone **1as** was employed, and no reaction was observed, supporting the requirement for enolate formation. To confirm the origin of the observed enantioselectivity, we investigated the irreversible proton transfer process using racemic **3a’** as the reactant (Figure 6e). Because **3a’** could not be separated from **3a**, it was synthesized through a titanium‐catalyzed metallation and carbonyl addition of propargylic acetate,^[^ ^69^ ^]^ followed by oxidation of the resulting alcohol. Upon treatment with **C9**, the desired product **3a** was smoothly generated over time, and the enantioselectivity matched that obtained in the one‐pot reaction, confirming that the axial chirality of **3a** arose from the enantioselective proton transfer of **3a’**. Moreover, only racemic **3a’** was detected during the reaction, indicating that its enantiomers were interconvertible. This observation provides evidence for a dynamic kinetic process in the irreversible isomerization step. Irreversibility was confirmed by treating (*rac*)‐**3a** with **C9** at room temperature: the allene product remained racemic and **3a′** was not formed (Figure 6f). The stability of (*R*)‐**3a** under basic conditions was examined (Figure 6g). Strong organic or inorganic bases caused decomposition, reducing both yield and ee (entries 1–2), whereas chirality was maintained with the chiral base **C3** and with the milder base Na_2_CO_3_ (entries 3–4).

To elucidate the origin of enantioselectivity, density functional theory (DFT) calculations were carried out using the Gaussian 16 program at the M062X‐D3‐IEFPCM(THF)/def2tzvpp//M062X‐D3‐IEFPCM(THF)/def2svp level, employing the alkynyl intermediate **3f’** and catalyst **C9** (Figure 6h). Taking (*rac*)**‐3f’** (consisting of (*R*)‐**3f’** and (*S*)‐**3f’**) as the starting material, the formation of complexes **IM‐S1** and **IM‐R1** served as the entry points for the two distinct pathways leading to (*S*) **‐3f** and (*R*) **‐3f**, respectively. In this Curtin–Hammett‐type dynamic kinetic resolution (DKR) system,^[^ ^70^ ^]^ both enantiomers interconvert rapidly, remaining racemic throughout the reaction. We first discuss the pathway originating from **IM‐R1** (red, Figure 6h), comparing it with the pathway from **IM‐S1** (black, Figure 6h). **IM‐R1** proceeds through transition state **TS‐R1** to generate intermediate **IM‐R2**, with a free energy barrier of 9.2 kcal mol^−1^ and an exergonicity of 3.1 kcal mol^−1^. This transformation is the rate‐determining step, and the calculated Gibbs free energy difference (ΔΔG‡) between the *R* and *S* products is about 3.0 kcal mol^−1^. Subsequently, proton transfer from the catalyst to the γ‐position of the substrate occurs readily via **TS‐R2**, with a free energy barrier of 4.5 kcal mol^−1^, whereas the corresponding barrier for the *S* pathway is 12.1−7.6 kcal mol^−1^ higher. In light of the above calculated results and experimental findings, we believe the deprotonation of **3f’** is the enantiodetermining step.^[^ ^71^ ^]^ Overall, these calculations indicate a strong preference for the *R* pathway, in agreement with the experimentally observed major product with *R*‐configuration.

**Figure 7:**
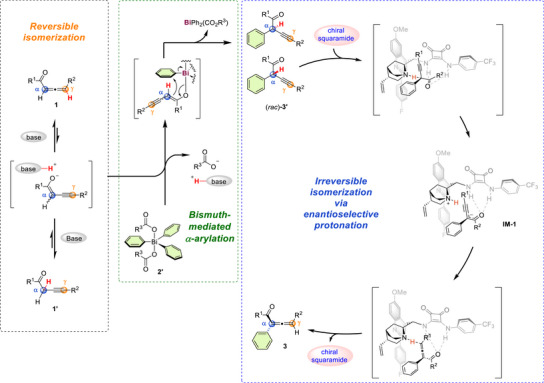
Proposed mechanism for α‐arylation reaction.

Based on the above studies, we propose a three‐phase mechanism, as illustrated in Figure 7. Under basic conditions, allene ketone **1** and its alkynyl isomer **1′** undergo rapid, reversible isomerization via an enolate intermediate. This enolate is then intercepted by the triarylbismuth(V) reagent **2′**, leading to the formation of the α‐arylated alkynyl isomer **3′** through a bismuth‐mediated arly migration. Notably, the resulting enantiomers of **3′** are interconverted via a DKR process in the presence of a base catalyst. During the subsequent enantioselective, irreversible isomerization step, deprotonation of (*S*)‐**3′** proceeds more rapidly than that of its enantiomer (*R*)‐**3′**, producing the enolate intermediate **IM‐1**. This is followed by a proton transfer from the catalyst to the γ‐position of the substrate, generating the desired (*R*)‐**3** product and regenerating the chiral squaramide catalyst.

## Conclusion

In summary, we have developed a straightforward method for synthesizing chiral α‐arylated allenones through a bismuth(V)‐mediated arylation in combination with a squaramide‐catalyzed protonation. Unlike existing protocols involving triarylbismuth(V) reagents, this three‐phase strategy is the first example to integrate bismuth‐mediated arly migration with asymmetric catalysis in a cascade process, affording the chiral products in high yields and with excellent enantioselectivities. The mechanism was elucidated using a combination of experimental and computational approaches. We believe that this work will open new avenues for the development of novel asymmetric reactions employing organobismuth(V) reagents.

## Conflict of Interests

The authors declare no conflict of interest.

## Supporting information



Supporting Information

Supporting Information

## Data Availability

The data that support the findings of this study are available from the corresponding author upon reasonable request.

## References

[1] R. Mohan , Nat. Chem. 2010, 2, 336. 21124518 10.1038/nchem.609

[2] H. Suzuki , N. Komatsu , T. Ogawa , T. Murafuji , T. Ikegami , Y. Matano , Organobismuth Chemistry 1st ed., Elsevier, Amsterdam 2001.

[3] O. Rohr , Ind. Lubr. Tribol. 2002, 54, 153–164.

[4] S. A. Singerling , R. M. Callaghan , *2018* USGS Minerals Yearbook: Bismuth, United States Geological Survey, Reston, VA (USA) 2018.

[5] J. M. Bothwell , S. W. Krabbe , R. S. Mohan , Chem. Soc. Rev. 2011, 40, 4649. 21589974 10.1039/c0cs00206b

[6] T. Ollevier , Bismuth‐Mediated Organic Reaction, Springer‐Verlag, Berlin, Heidelberg 2012.

[7] D. H. R. Barton , D. J. Lester , W. B. Motherwell , M. T. B. Papoula , J. Chem. Soc. Chem. Commun. 1979, 706–707.

[8] D. H. R. Barton , J. P. Kitchin , D. J. Lester , W. B. Motherwell , M. T. B. Papoula , Tetrahedron 1981, 37, 73–79.

[9] J.‐P. Finet , Chem. Rev. 1989, 89, 1487–1501.

[10] T. Ooi , R. Goto , K. Maruoka , J. Am. Chem. Soc. 2003, 125, 10494–10495. 12940712 10.1021/ja030150k

[11] P. K. Koech , M. J. Krische , J. Am. Chem. Soc. 2004, 126, 5350–5351. 15113193 10.1021/ja048987i

[12] A. Gagnon , J. Dansereau , A. L. Roch , Synthesis 2017, 49, 1707–1745.

[13] O. Planas , F. Wang , M. Leutzsch , J. Cornella , Science 2020, 367, 313–317. 31949081 10.1126/science.aaz2258

[14] A. Roch , H. Chan , A. Gagnon , Eur. J. Org. Chem. 2020, 2020, 5815–5819.

[15] H. W. Moon , J. Cornella , ACS Catal. 2022, 12, 1382–1393. 35096470 10.1021/acscatal.1c04897 PMC8787757

[16] M. Mato , D. Spinnato , M. Leutzsch , H. W. Moon , E. J. Reijerse , J. Cornella , Nat. Chem. 2023, 15, 1138–1145. 37264103 10.1038/s41557-023-01229-7 PMC10396954

[17] M. Mato , J. Cornella , Angew. Chem. Int. Ed. 2024, 63, e202315046. 10.1002/anie.202315046 37988225

[18] N. D. Chiappini , E. P. Geunes , E. T. Bodak , R. R. Knowles , ACS Catal. 2024, 14, 2664–2670. 40630115 10.1021/acscatal.3c05598 PMC12237399

[19] N. Tang , X. Li , C. Li , L. Le , Y. Ou , S. Yin , R. Qiu , Angew. Chem., Int. Ed. 2025, e202514967. 10.1002/anie.202514967 40904068

[20] M. Mato , A. Stamoulis , P. Cleto Bruzzese , J. Cornella , Angew. Chem., Int. Ed. 2025, 64, e202418367. 10.1002/anie.202418367 PMC11773318 39436157

[21] D. H. R. Barton , D. J. Lester , W. B. Motherwell , M. T. B. Papoula , J. Chem. Soc. Chem. Commun. 1980, 246–247.

[22] D. H. R. Barton , N. Y. Bhatnagar , J.‐C. Blazejewski , B. Charpiot , J.‐P. Finet , D. J. Lester , W. B. Motherwell , M. T. B. Papoula , S. P. Stanforth , J. Chem. Soc. Perkin Trans. 1 1985, 2657.

[23] D. H. R. Barton , M. T. B. Papoula , J. Guiihem , W. B. Motherwell , C. Pascard , E. T. H. Dau , J. Chem. Soc. Chem. Commun. 1982, 732.

[24] D. H. R. Barton , J.‐C. Blazejewski , B. Charpiot , J.‐P. Finet , W. B. Motherwell , M. T. B. Papoula , S. P. Stanforth , J. Chem. Soc. Perkin Trans. 1 1985, 2667.

[25] M. J. O'Donnell , W. D. Bennett , W. N. Jacobsen , Y. Ma , Tetrahedron Lett. 1989, 30, 3909–3912.

[26] M. S. Akhtar , W. J. Brouillette , D. V. Waterhous , J. Org. Chem. 1990, 55, 5222–5225.

[27] T. Arnauld , D. H. R. Barton , J. Org. Chem. 1999, 64, 6915–6917. 11674709 10.1021/jo9905928

[28] P. J. Krawczuk , N. Schöne , P. S. Baran , Org. Lett. 2009, 11, 4770–4773. 19795876 10.1021/ol901963v PMC2783297

[29] H. Suzuki , T. Murafujia , N. Azuma , J. Chem. Soc. Perkin Trans. 1 1992, 1593–1660.

[30] M. Jurrat , L. Maggi , W. Lewis , L. T. Ball , Nat. Chem. 2020, 12, 260–269. 32108765 10.1038/s41557-020-0425-4

[31] K. Ruffell , S. P. Argent , K. B. Ling , L. T. Ball , Angew. Chem. Int. Ed. 2022, 61, e202210840. 10.1002/anie.202210840 PMC9805042 35950691

[32] A. Fox , L. T. Ball , Org. Process Res. Dev. 2024, 28, 632–639. 38384679 10.1021/acs.oprd.3c00509 PMC10877598

[33] Y. Ma , A. A. Hussein , Chem. Front. 2022, 9, 4890–4901.

[34] B. J. Cowen , S. J. Miller , Chem. Soc. Rev. 2009, 38, 3102. 19847345 10.1039/b816700c

[35] N. Krause , A. S. K. Hashmi , Modern Allene Chemistry, Wiley‐VCH, Weinheim 2004.

[36] A. Hoffmann‐Röder , N. Krause , Angew. Chem. Int. Ed. 2004, 43, 1196–1216. 10.1002/anie.200300628 14991780

[37] S. Ma , Chem. Rev. 2005, 105, 2829–2872. 16011326 10.1021/cr020024j

[38] S. Ma , Acc. Chem. Res. 2009, 42, 1679–1688. 19603781 10.1021/ar900153r

[39] C. Aubert , L. Fensterbank , P. Garcia , M. Malacria , A. Simonneau , Chem. Rev. 2011, 111, 1954–1993. 21391568 10.1021/cr100376w

[40] S. Yu , S. Ma , Angew. Chem., Int. Ed. 2012, 51, 3074–3112. 10.1002/anie.201101460 22271630

[41] P. Rivera‐Fuentes , F. Diederich , Angew. Chem. Int. Ed. 2012, 51, 2818–2828. 10.1002/anie.201108001 22308109

[42] J. Ye , S. Ma , Acc. Chem. Res. 2014, 47, 989–1000. 24479609 10.1021/ar4002069

[43] X. Wang , X. Chen , W. Lin , P. Li , W. Li , Adv. Synth. Catal. 2022, 364, 1212–1222.

[44] C. Song , X. Bai , B. Li , Y. Dang , S. Yu , J. Am. Chem. Soc. 2024, 146, 21137–21146. 39024194 10.1021/jacs.4c07126

[45] C. Song , S. Yu , ACS Catal. 2024, 14, 15997–16002.

[46] J. M. Alonso , P. Almendros , Adv. Synth. Catal. 2023, 365, 1332–1384.

[47] J. A. Marshall , Y. Tang , J. Org. Chem. 1993, 58, 3233–3234.

[48] H. Ni , X. Tang , W. Zheng , W. Yao , N. Ullah , Y. Lu , Angew. Chem. Int. Ed. 2017, 56, 14222–14226. 10.1002/anie.201707183 28816392

[49] M. Wu , Z. Han , K. Li , J. Wu , K. Ding , Y. Lu , J. Am. Chem. Soc. 2019, 141, 16362–16373. 31545594 10.1021/jacs.9b07418

[50] X. Tang , C. X. A. Tan , W.‐L. Chan , F. Zhang , W. Zheng , Y. Lu , ACS Catal. 2021, 11, 1361–1367.

[51] G. Shen , F. He , W. Xie , H. Gu , X. Yang , ACS Catal. 2023, 13, 12472–12480.

[52] J. Ye , S. Ma , Org. Chem. Front. 2014, 1, 1210–1224.

[53] Q. Yao , Y. Liao , L. Lin , X. Lin , J. Ji , X. Liu , X. Feng , Angew. Chem. Int. Ed. 2016, 55, 1859–1863. 10.1002/anie.201509455 PMC4738401 26694204

[54] P. H. Poulsen , Y. Li , V. H. Lauridsen , D. K. B. Jørgensen , T. A. Palazzo , M. Meazza , K. A. Jørgensen , Angew. Chem., Int. Ed. 2018, 57, 10661–10665. 10.1002/anie.201806238 29917329

[55] J. Wang , S. Zheng , S. Rajkumar , J. Xie , N. Yu , Q. Peng , X. Yang , Nat. Commun. 2020, 11, 5527. 33139734 10.1038/s41467-020-19294-8 PMC7608664

[56] Y. Hu , W. Shi , B. Zheng , J. Liao , W. Wang , Y. Wu , H. Guo , Chem. Int. Ed. 2020, 59, 19820–19824. 10.1002/anie.202009460 32820579

[57] X. Zeng , F.‐H. Zhang , Z. Wang , Org. Chem. Front. 2023, 10, 310–316.

[58] For an earlier report on the synthesis of racemic α‐arylated allene ketones from propargyl alcohols via a Claisen rearrangement, see: J. M. Reuter , R. G. Salomon , Tetrahedron Lett. 1978, 19, 3199–3202.

[59] H. Liu , D. Leow , K.‐W. Huang , C.‐H. Tan , J. Am. Chem. Soc. 2009, 131, 7212–7213. 19422238 10.1021/ja901528b

[60] H. Y. Kim , J. Y. Li , K. Oh , J. Org. Chem. 2012, 77, 11132–11145. 23198987 10.1021/jo302253c

[61] N. J. Line , B. P. Witherspoon , E. N. Hancock , M. K. Brown , J. Am. Chem. Soc. 2017, 139, 14392–14395. 28985064 10.1021/jacs.7b09844 PMC5704961

[62] T. Inokuma , M. Furukawa , T. Uno , Y. Suzuki , K. Yoshida , Y. Yano , K. Matsuzaki , Y. Takemoto , Chem. ‐ Eur. J. 2011, 17, 10470–10477. 21812044 10.1002/chem.201101338

[63] T. Inokuma , M. Furukawa , Y. Suzuki , T. Kimachi , Y. Kobayashi , Y. Takemoto , ChemCatChem 2012, 4, 983–985.

[64] X. Gu , X.i. Mo , W. Bai , P. Xie , W. Hu , J. Jiang , J. Am. Chem. Soc. 2023, 145, 20031–20040. 37642381 10.1021/jacs.3c06906

[65] D. H. R. Barton , N. Y. Bhatnagar , J.‐P. Finet , W. B. Motherwell , Tetrahedron 1986, 42, 3111–3122.

[66] K. Akiba , K. Ohdoi , Y. Yamamoto , Tetrahedron Lett. 1988, 29, 3817–3820.

[67] J. Kuziola , N. Nöthling , M. Leutzsch , J. Cornella , Chem. Commun. 2024, 60, 10532–10535. 10.1039/d4cc03364g PMC11372555 39229666

[68] S. Combes , J.‐P. Finet , Tetrahedron 1998, 54, 4313–4318.

[69] J. L. Meloche , P. T. Vednor , J. B. Gianino , A. G. Oliver , B. L. Ashfeld , Tetrahedron Lett. 2014, 55, 5025–5028.

[70] Q. Peng , F. Duarte , R. S. Paton , Chem. Soc. Rev. 2016, 45, 6093–6107. 27722685 10.1039/c6cs00573j

[71] For detailed discussion, see the Supporting Information.

