# Supplementary material for: Enantioselective Synthesis of α‐Arylated Allene Ketones Through Sequential Bismuth(V)‐mediated Arylation and Organocatalytic Protonation
Source: Angew Chem Int Ed Engl. 2025 Oct 7;64(48):e202517136. doi: 10.1002/anie.202517136 (PMC12643346; doi:10.1002/anie.202517136)
Supplement: Supplementary file 1 — Supporting Information [file ANIE-64-e202517136-s001.pdf]

Supporting Information

Enantioselective synthesis of α-arylated allene ketones through sequential bismuth(V)-mediated arylation and organocatalytic protonation

Kun Zhu, Yuli Sun, Yunhan Ma, Zugen Wu, Yixin Lu*

[*] K. Zhu, Y. Sun, Y. Ma, Z. Wu, Prof. Y. Lu
Department of Chemistry, National University of Singapore, Singapore 117543, Republic of Singapore.

K. Zhu, Prof. Y. Lu
Energy and Environmental Nanotech Platform, National University of Singapore (Suzhou) Research Institute, Suzhou 215123, China.

E-mail: chmlyx@nus.edu.sg

Table of Contents

1. General Information....3
2. Optimization of Reaction Conditions....4
3. General Procedure for Enantioselective α-Arylation of Allenones....7
4. Control Experiments and Mechanistic Studies....8
5. Calculation Studies....14
6. Determination of the Absolute Configuration....48
7. General procedure for preparing allenones....53
8. Reaction Extension....60
9. X-ray Crystallography Data....64
10. Characterization Data of Products....65
11. Copy of NMR Spectra....107
12. References....214

1. General Information

Unless otherwise specified, all reagents and solvents were purchased from commercial suppliers and used without further purification. Flash silica gel column chromatography was performed using silica gel (particle size 300-400 mesh). NMR spectra were recorded on Bruker DPX 400 spectrometer at 400 MHz or 500 MHz or 600 MHz for ^{1}H NMR, 100 MHz or 125 MHz or 150 MHz for ^{13}C NMR, 376 MHz or 471 MHz for ^{19}F NMR. Chemical shifts were calibrated using residual solvent as an internal reference ($CDCl_3$: 7.26 ppm ^{1}H NMR, 77.00 ppm ^{13}C NMR). ^{1}H NMR spectroscopy splitting patterns were designated as singlet (s), doublet (d), triplet (t), quartet (q). Splitting patterns that could not be interpreted or easily visualized were designated as multiplet (m). Optical rotations were measured using an Anton Paar MCP-100 polarimeter. Enantiomeric excesses were determined by HPLC analysis on a chiral stationary phase using a chiral IC column, a chiral IA column, a chiral IE column, a chiral IG column, and a chiral OJ-H column. Allenones,[1-3] triarylbismuth(V) reagent,[4-11] hypervalent iodine(III) reagents[12] and catalysts[13,14] were all synthesized by following previously reported procedures.

2. Optimization of Reaction Conditions

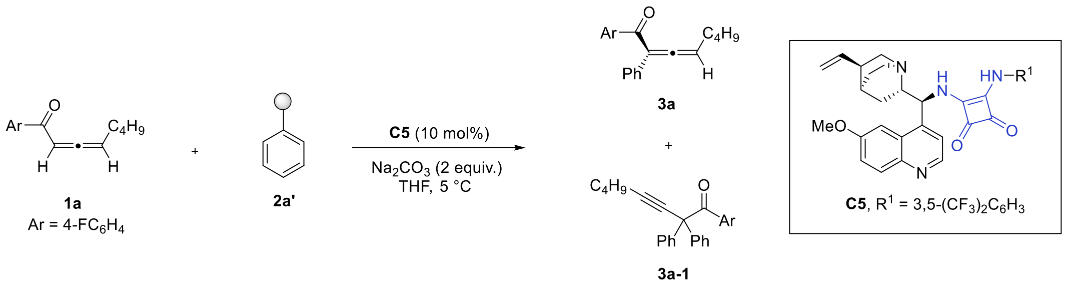

entry	2a'	Yield[a](%)	ee[b](%)	3a:3a-1[b]	BDE of Csp^2-Bi (kcal/mol)[c]
1	**2a-1'**	10	60	6:1	63
2	**2a-2'**	7	53	9:1	71
3	**2a-3'**	29	68	93:7	72
4	**2a-4'**	35	77	>25:1	76
5	**2a-5'**	44	79	>25:1	59
6	**2a-6'**	59	84	>25:1	57
7	**2a-7'**	72	84	>25:1	58
8	**2a-8'**	**83**	**84**	**>25:1**	59

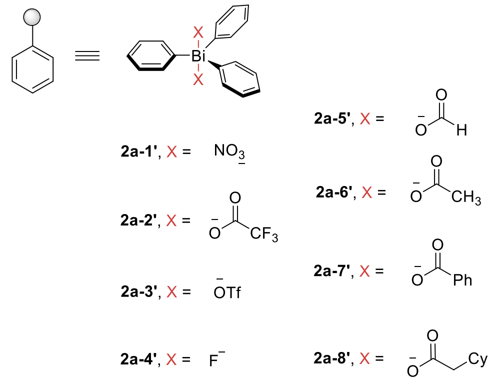

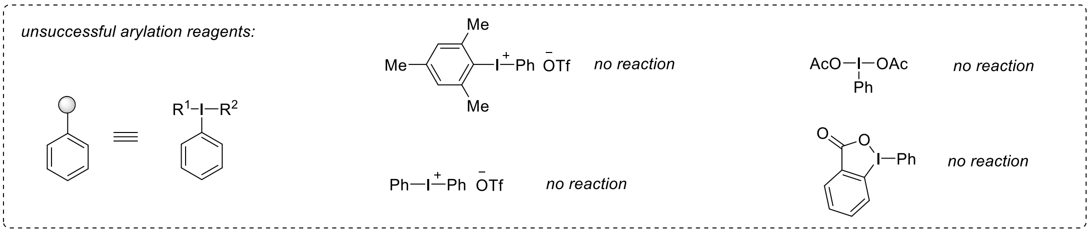

Reaction conditions: **1a** (0.06 mmol), **2a'** (0.05 mmol), **C5** (10 mol%), Na_2CO_3 (2 equiv.), THF (1 mL) at 5 °C, 16 h. [a]Determined by crude ^{1}H NMR analysis with mesitylene as an internal standard. [b]Determined by HPLC. [c]Bond dissociation energies of Csp^2-Bi bond were calculated via Gaussian 16.

Scheme S1: Screening triarylbismuth(V) reagents and other arylation reagents.

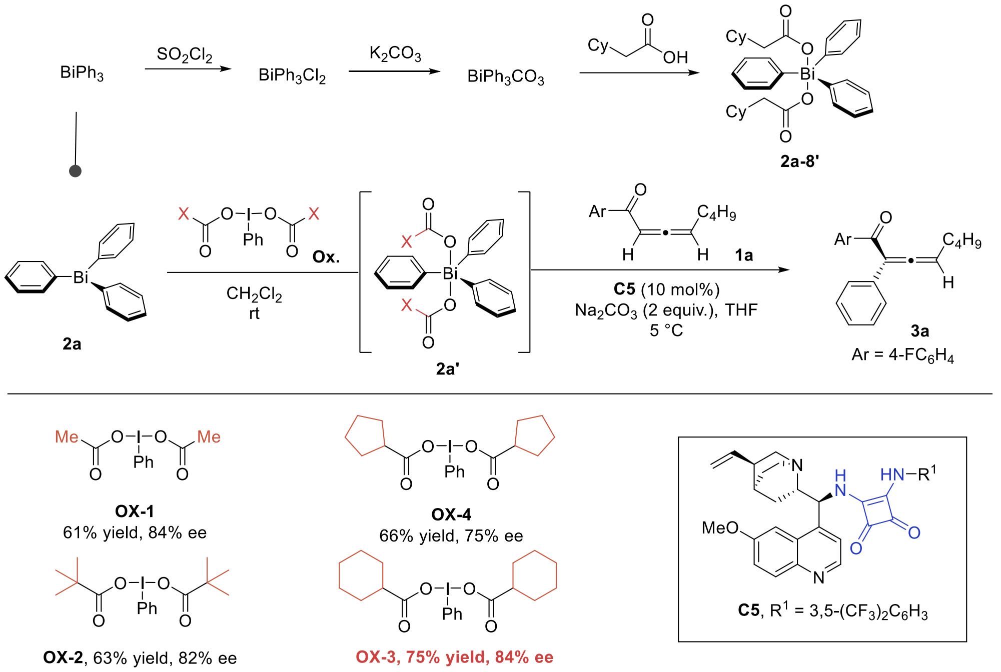

Reaction conditions: **1a** (0.075 mmol), **2a'** (0.05 mmol), **C5** (10 mol%), Na_2CO_3 (2 equiv.), THF (1 mL) at 5 °C, 16 h. **2a'** was prepared in situ, directly from **2a** (0.05 mmol) with **Ox** (0.055 mmol) in CH_2Cl_2 (0.5 mL), subsequently used after evaporation of the solvent. Cy = cyclohexyl.

Scheme S2: Modified arylation process directly from triphenylbismuth **2a** and evaluation of various hypervalent iodine(III) reagents.

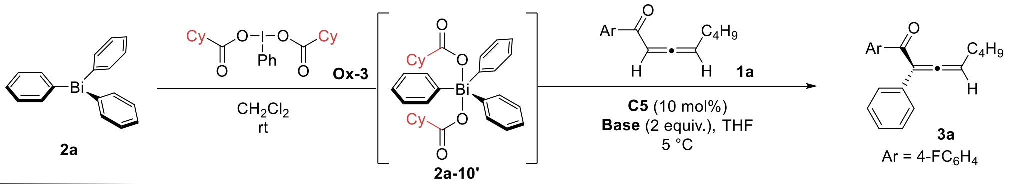

Na_2CO_3	**75% yield, 84% ee**	K_3PO_4	36% yield, 10% ee
$NaHCO_3$	trace	KOAc	80% yield, 23% ee
Cs_2CO_3	trace	NaOAc	73% yield, 77% ee
Na_2HPO_4	57% yield, 68% ee	NaOPiv	61% yield, 23% ee
NaOtBu	trace	NaOBz	70% yield, 78% ee

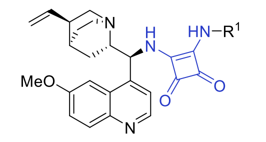

C5, R[1] = 3,5-$(CF_3)_2C_6H_3$

Reaction conditions: **1a** (0.075 mmol), **2a-10'** (0.05 mmol), **C5** (10 mol%), **Base** (2 equiv.), THF (1 mL) at 5 °C, 16 h. **2a-10'** was prepared in situ, directly from **2a** (0.05 mmol) with **Ox-3** (0.055 mmol) in CH_2Cl_2 (0.5 mL), subsequently used after evaporation of the solvent. Cy = cyclohexyl. OPiv = Pivaloyl. OBz = Benzoyl.

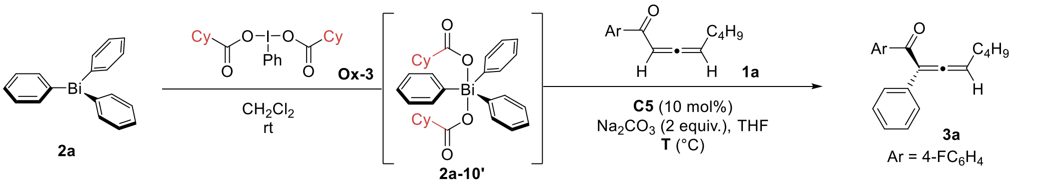

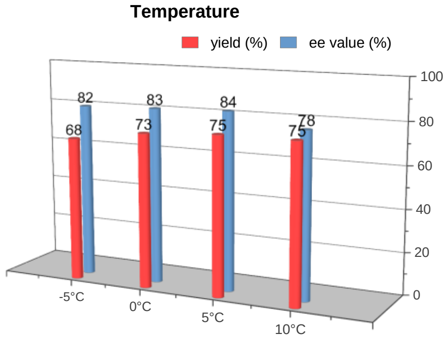

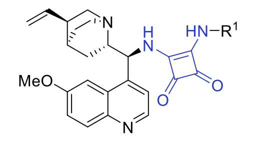

C5, R[1] = 3,5-$(CF_3)_2C_6H_3$

Reaction conditions: **1a** (0.075 mmol), **2a-10'** (0.05 mmol), **C5** (10 mol%), Na_2CO_3 (2 equiv.), THF (1 mL) at **T** (°C), 16 h. **2a-10'** was prepared in situ, directly from **2a** (0.05 mmol) with **Ox-3** (0.055 mmol) in CH_2Cl_2 (0.5 mL), subsequently used after evaporation of the solvent. Cy = cyclohexyl.

Scheme S3: Further optimization of reaction conditions.

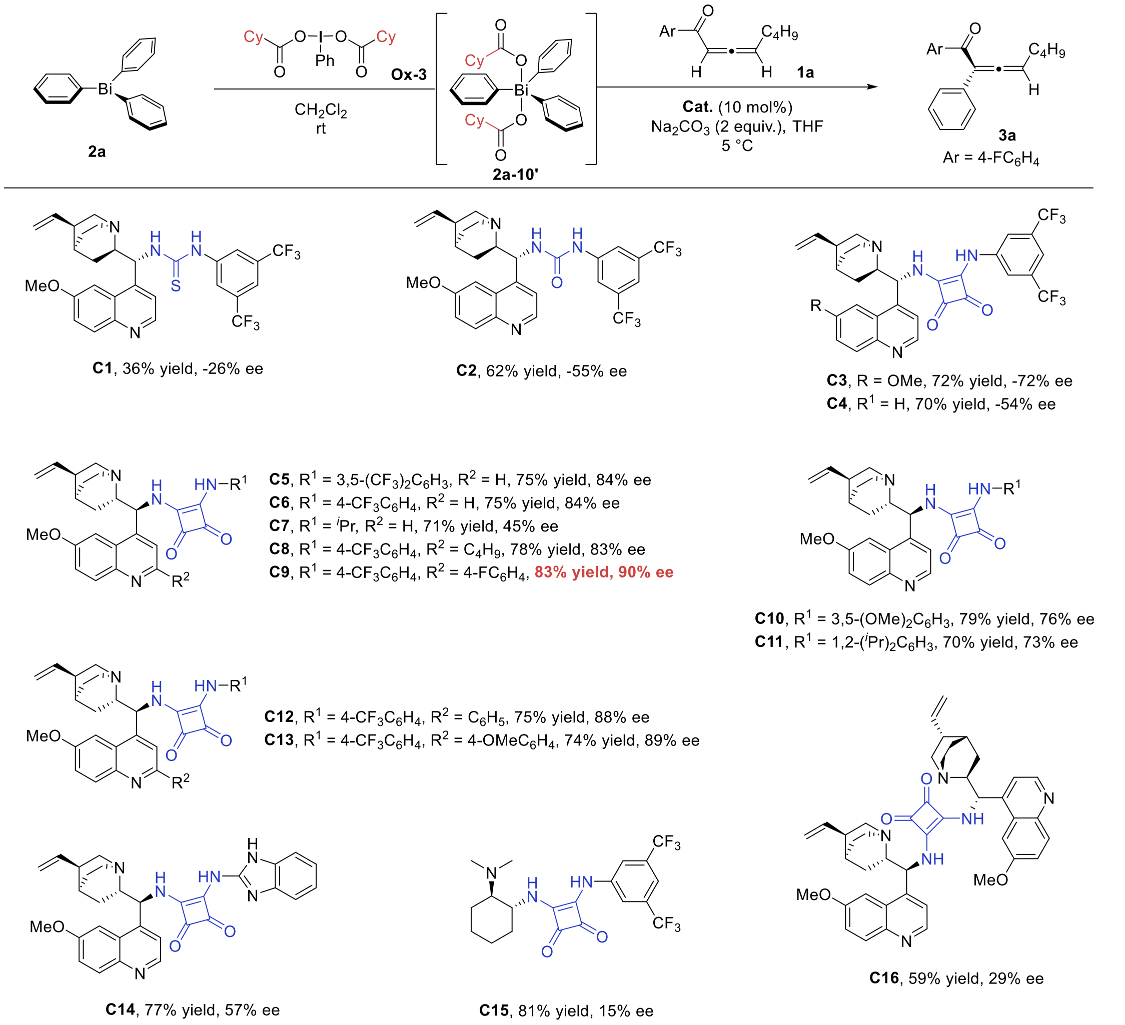

Reaction conditions: **1a** (0.075 mmol), **2a-10'** (0.05 mmol), **Cat** (10 mol%), Na_2CO_3 (2 equiv.), THF (1 mL) at 5 °C, 16 h. **2a'** was prepared in situ, directly from **2a** (0.05 mmol) with **Ox-3** (0.055 mmol) in CH_2Cl_2 (0.5 mL), subsequently used after evaporation of the solvent. Cy = cyclohexyl.

Scheme S4: Screening of catalysts.

3. General Procedure for Enantioselective α-Arylation of Allenones

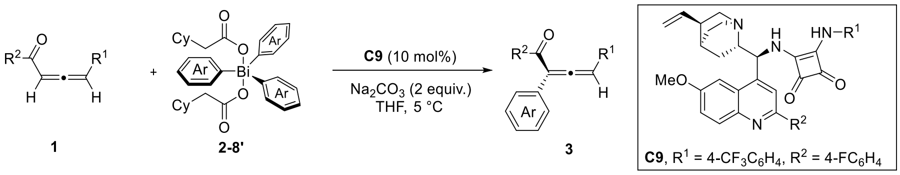

General procedure A (GP-A):

To a dried tube was added **2'** (0.1 mmol, 1 equiv.) and Na_2CO_3 (0.2 mmol, 2 equiv.) sequentially, which was followed by adding a solution of **1** (0.15 mmol, 1.5 equiv.) and **C9** (0.01 mmol, 10 mol%) in THF (2 mL). After stirring at 5 °C for 16 h, the mixture was filtered through celite and the filtrate was concentrated *in vacuo*. The residue was then purified by column chromatography on silica gel to give the desired product.

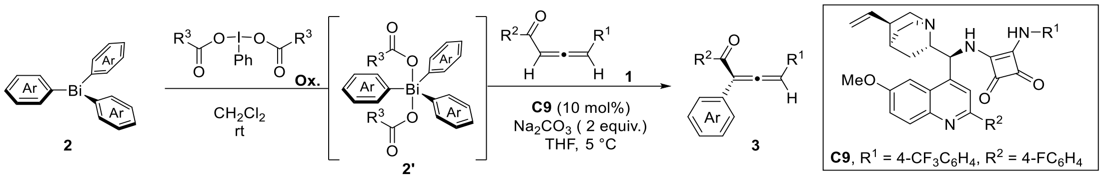

General procedure B (GP-B):

Preparation of Aryl$_3$Bi(O$_2$CR)$_2$. In a round bottom flask, **2** (0.1 mmol, 1.0 equiv.) and PhI(O_2CR)$_2$ (0.11 mmol, 1.1 equiv.) were dissolved in DCM (1 mL) and stirred until the full consumption of **2**. Upon completion, DCM was removed *in vacuo*, and the resulting crude of **2'** was directly used without further purification.

α-Arylation of allenones with Aryl$_3$Bi(O$_2$CR)$_2$. A solution of **2'** (0.1 mmol, 1 equiv.) in THF (1 mL) and Na_2CO_3 (0.2 mmol, 2 equiv.) were charged in a dried tube sequentially. A solution of **1** (0.15 mmol, 1.5 equiv.) and **C9** (0.01 mmol, 10 mol%) in THF (1 mL) was then added. After stirring at 5 °C for 16 h, the mixture was filtered through celite and the filtrate was concentrated *in vacuo*. The residue was subsequently purified by column chromatography on silica gel to give the desired product.

(For **3ae**-**3ai**, **2'** was prepared with iodobenzene dicyclohexanecarboxylate. For **3aj**-**3ap**, corresponding **2'** was prepared with iodobenzene diacetate.)

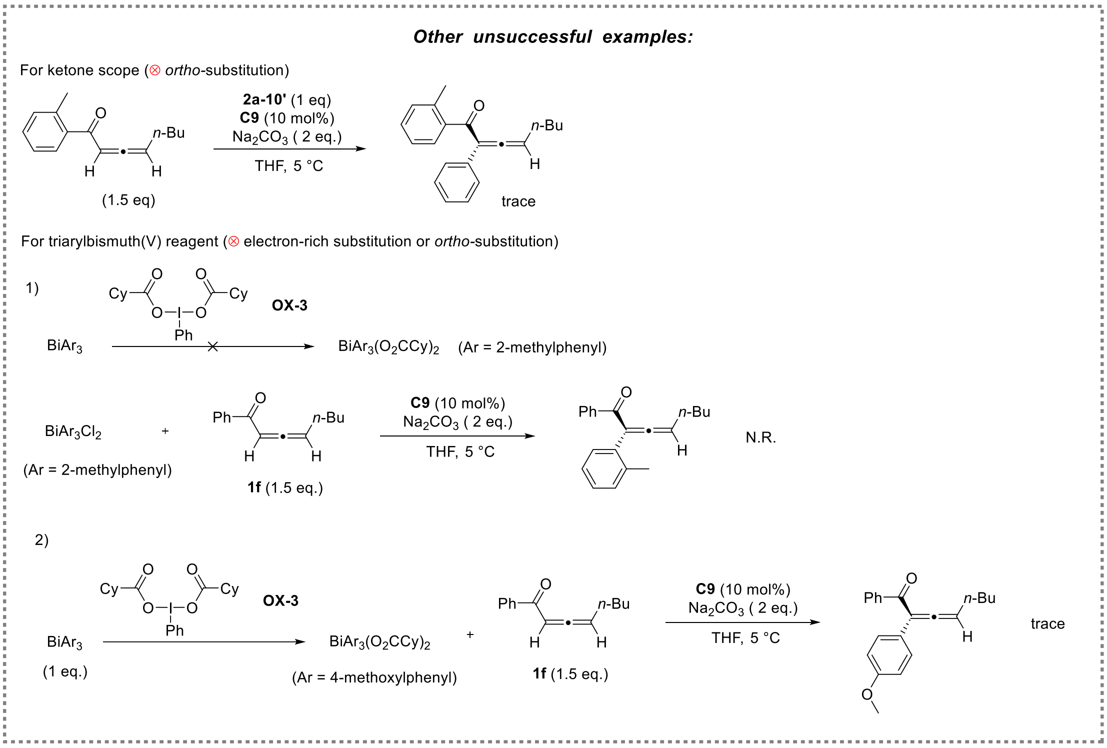

Scheme S5: Unsuccessful examples

4. Control Experiments and Mechanistic Studies

A. Monitoring reaction of 1a and 2a-8'

1a and **1a'** were synthesized via general synthetic procedure reported in literatures[2], and separated by column chromatography on silica gel. **2a-8'** was prepared from triphenylbismuth **2a** via known oxidation[10] and anion exchange reaction[6,7].

Following GP-A: To a dried tube was added **2a-8'** (18 mg, 0.025 mmol, 1 equiv.) and Na_2CO_3 (5 mg, 0.05 mmol, 2 equiv.) sequentially, which was followed by adding a solution of **1a** (8 mg, 0.0375 mmol, 1.5 equiv.) and **C9** (1.5 mg, 0.0025 mmol, 10 mol%) in THF (0.5 mL). The reaction mixture was then stirred at 5 °C. Following the same procedure, 9 reactions were conducted. After certain period of time, the corresponding yield was determined by HPLC analysis with 1,1-Diphenylethylene as internal standard and calibrated according to Beer–Lambert law (the ratio of molar absorption coefficient).
ε_{rel} = 0.66 (1,1-diphenylethylene:**3a**), ε_{rel} = 1.27 (1,1-diphenylethylene:**3a'**)

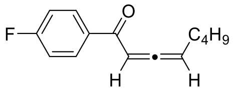

1a

1-(4-fluorophenyl)octa-2,3-dien-1-one (1a):

(1:30 ethyl acetate:hexanes as eluent, Rf = 0.3, yellow oil)
^{1}H NMR (400 MHz, $CDCl_3$) δ 7.91 (dd, *J* = 8.8, 5.2 Hz, 2H), 7.10 (t, *J* = 8.8 Hz, 2H), 6.32 – 6.29 (m, 3.0 Hz, 1H), 5.61 (dd, *J* = 13.2, 7.2 Hz, 1H), 2.34 – 2.03 (m, 2H), 1.50 – 1.37 (m, 1H), 1.36 – 1.28 (m, 2H), 0.88 (t, *J* = 7.6 Hz, 3H). ^{13}C NMR (100 MHz, $CDCl_3$) δ 213.8, 190.4, 165.4 (d, *J* = 252.2 Hz), 134.0 (d, *J* = 3.0 Hz), 131.2 (d, *J* = 9.1 Hz), 115.3 (d, *J* = 21.6 Hz), 95.2, 93.9, 30.9, 27.4, 22.0, 13.7. ^{19}F NMR (471 MHz, $CDCl_3$) δ -106.1. HRMS (ESI): m/z calcd. for $C_{14}H_{16}FO^+$([M+H]$^+$) = 219.1180, found = 219.1181.

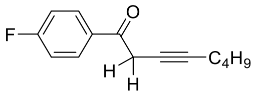

1a'

1-(4-fluorophenyl)oct-3-yn-1-one (1a'):
(1:30 ethyl acetate:hexanes as eluent, Rf = 0.3, yellow oil)
^{1}H NMR (400 MHz, $CDCl_3$) δ 8.04 (dd, *J* = 8.8, 5.2 Hz, 2H), 7.13 (t, *J* = 8.8 Hz, 2H), 3.77 (t, *J* = 2.8 Hz, 2H), 2.21 – 2.16 (m, 2H), 1.51 – 1.41 (m, 2H), 1.40 – 1.30 (m, 2H), 0.87 (t, *J* = 7.2 Hz, 3H). ^{13}C NMR (100 MHz, $CDCl_3$) δ 192.5, 165.88 (d, *J* = 253.0 Hz), 131.9 (d, *J* = 3.0 Hz), 131.4 (d, *J* = 9.4 Hz), 115.7 (d, *J* = 22.0 Hz), 86.1, 72.1, 31.0, 30.7, 21.9, 18.5, 13.5. ^{19}F NMR (471 MHz, $CDCl_3$) δ -104.6. HRMS (ESI): m/z calcd. for $C_{14}H_{16}FO^+$([M+H]$^+$) = 219.1180, found = 219.1181.

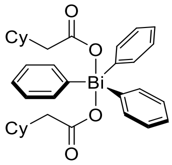

2a-8'

(2-cyclohexylacetoxy)triphenyl-l5-bismuthaneyl 2-cyclohexylacetate (2a-8'):
White solid. ^{1}H NMR (400 MHz, $CDCl_3$) δ 8.16 (d, *J* = 7.2 Hz, 6H), 7.57 (t, *J* = 7.6 Hz, 6H), 7.45 (t, *J* = 7.6 Hz, 3H), 2.37 – 0.40 (m, 26H). ^{13}C NMR (100 MHz, $CDCl_3$) δ 179.9, 161.4, 134.1, 130.9, 130.4, 43.1, 35.9, 32.8, 26.1. HRMS (ESI): m/z calcd. for $C_{34}H_{41}BiNaO_4^+$([M+Na]$^+$) = 745.2701, found = 745.2700.

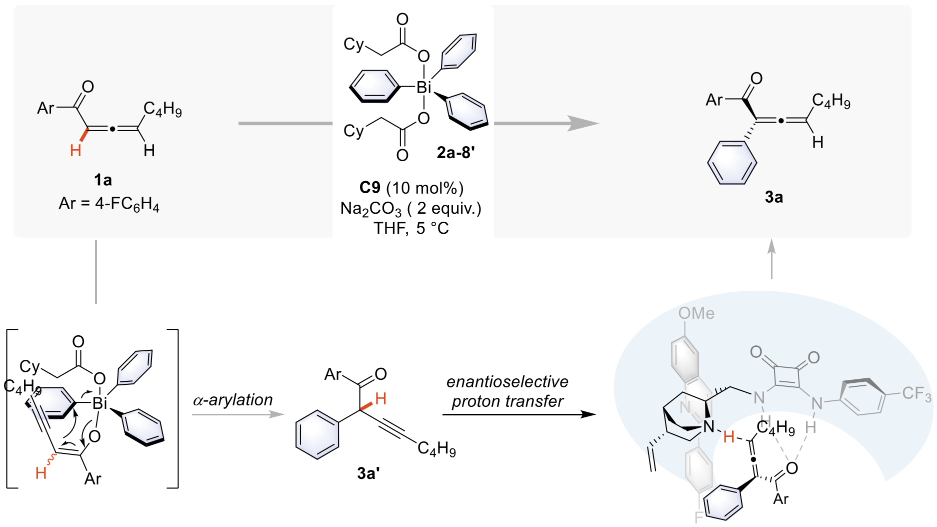

entry	time (h)	yield of **3a'** (%)	yield of **3a** (%)	ee of **3a** (%)
1	0.08	5.0	0	--
2	0.25	25.4	1	--
3	0.5	38.1	7.3	90
4	1	44.5	9.9	90
5	2	30.5	25.8	90
6	3.5	18	39	90
7	6	12.7	48.2	90
8	8	6.4	66	90
9	12	0	74	90

Scheme S6: Measured concentration data.

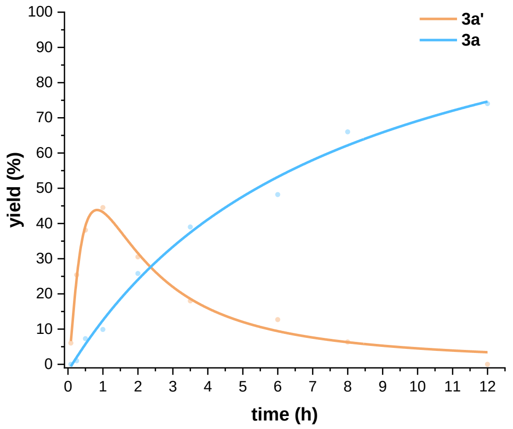

Figure S1: A. Monitoring reaction of **1a** and **2a-8'**.

B. Control experiment of different starting materials

Equilibrium of reversible isomerization: **C9** (1.5 mg, 0.0025 mmol), Na_2CO_3 (5 mg, 0.05 mmol) and a solution of **1a** (8 mg, 0.0375 mmol) in THF (0.5 mL) were added to a dried reaction tube at 5 °C. The reaction was then stirred for 10 min and 3 h at 5 °C. After flash column to remove the catalyst residue, the equilibrium ratio of **1a** and **1a'** was determined by ^{1}H NMR.

Control experiment of different starting materials: To a dried tube was added **2a-8'** (18 mg, 0.025 mmol, 1 equiv.) and Na_2CO_3 (5 mg, 0.05 mmol, 2 equiv.) sequentially, which was followed by adding a solution of **1a** (8 mg, 0.0375 mmol, 1.5 equiv.) and **C9** (1.5 mg, 0.0025 mmol, 10 mol%) in THF (0.5 mL). The reaction mixture was then stirred at 5 °C. Following the same procedure, 3 reactions were conducted. After certain period, the corresponding yield was determined by HPLC analysis with 1,1-Diphenylethylene as internal standard and calibrated according to Beer–Lambert law (the ratio of molar absorption coefficient).

ε_{rel} = 0.66 (1,1-diphenylethylene:**3a**), ε_{rel} = 1.27 (1,1-diphenylethylene:**3a'**)

ε_{rel} = 0.73 (1,1-diphenylethylene:**1a**). ε_{rel} = 1.9 (1,1-diphenylethylene:**1a'**)

Same procedure was followed when **1a'** was engaged as starting material.

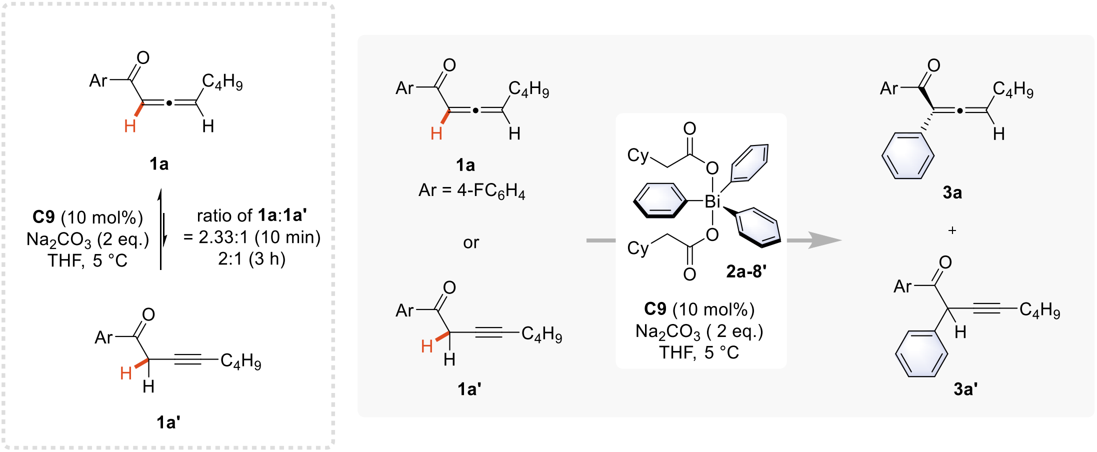

entry	starting materials	time (h)	ratio of **1a** and **1a'**	yield of **3a'** (%)	yield of **3a** (%)	ee of **3a** (%)
1	**1a**	0.08	2.3:1	5	0	--
2	**1a**	1	2.2:1	45	10	90
3	**1a**	12	2:1	0	74	90
4	**1a'**	0.08	1.1:1	18	0	--
5	**1a'**	1	1.54:1	40	18	90
6	**1a'**	12	2:1	0	63	90

Scheme S7: Equilibrium of reversible isomerization and control experiment of different starting materials.

C. Monitoring irreversible isomerization of (*rac*)-3a'

According to literature, (*rac*)-**3a'** was prepared via an initial titanium-catalyzed metallation and carbonyl addition of propargylic acetate,[15] followed by the oxidation of the resulting alcohol.[16]

Procedure: A solution of **3a'** (15 mg, 0.05 mmol, 1.0 equiv.) in THF (1 mL) and **C9** (3 mg, 0.005 mmol, 10 mol%), Na_2CO_3 (5 mg, 0.05 mmol, 1 equiv.) were added to a dried tube. The reaction mixture was then stirred at 5 °C. After certain period, the reaction was monitored by HPLC with 1,1-Diphenylethylene

as internal standard. The ratio of **3a** and **3a'** were calibrated according to Beer–Lambert law (the ratio of molar absorption coefficient).

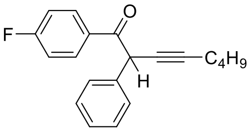

(*rac*)-**3a'**

(*rac*)-1-(4-fluorophenyl)-2-phenyloct-3-yn-1-ol (3a'):
^{1}H NMR (400 MHz, $CDCl_3$) δ 8.09 (dd, *J* = 8.8, 5.2 Hz, 2H), 7.44 (d, *J* = 7.2 Hz, 2H), 7.37 – 7.34 (m, 2H), 7.31 – 7.25 (m, 1H), 7.08 (t, *J* = 8.4 Hz, 2H), 5.32 (s, 1H), 2.39 – 2.16 (m, 2H), 1.53 – 1.45 (m, 2H), 1.42 – 1.32 (m, 2H), 0.87 (t, *J* = 7.6 Hz, 3H). ^{13}C NMR (100 MHz, $CDCl_3$) δ 193.1, 165.6 (d, *J* = 253.7 Hz), 136.0, 132.2 (d, *J* = 9.3 Hz), 131.4 (d, *J* = 3.1 Hz), 128.7, 128.1, 127.6, 115.5 (d, *J* = 21.8 Hz), 88.7, 75.9, 47.8, 30.6, 21.9, 18.6, 13.5. ^{19}F NMR (377 MHz, $CDCl_3$) δ -104.8. HRMS (ESI): m/z calcd. for $C_{20}H_{20}FO^+$([M+H]$^+$) = 295.1493, found = 295.1494.

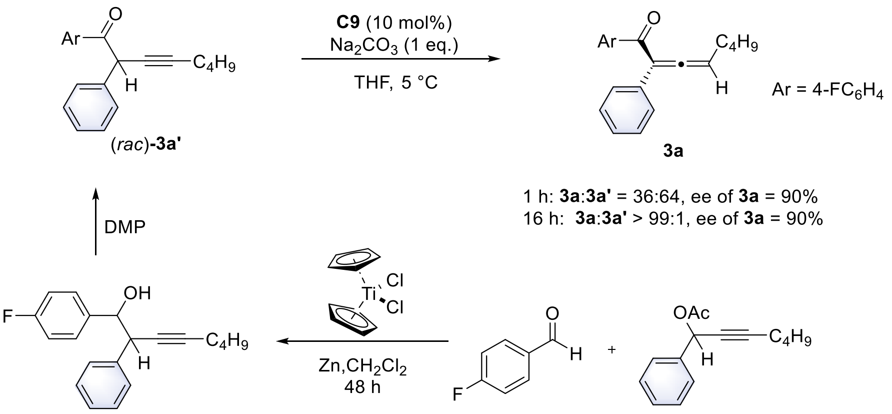

1 h:

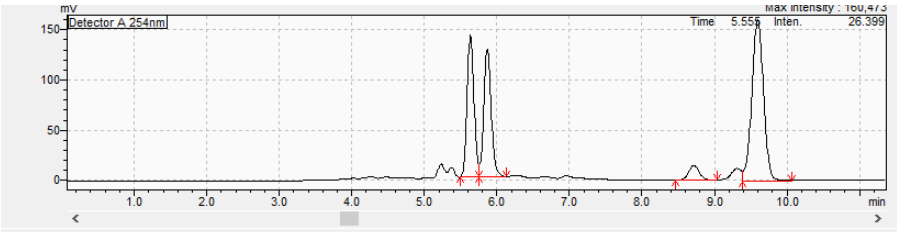

Results View - Peak Table

Peak Table | Compound | Group | Calibration Curve

Peak#	Ret. Time	Area	Height	Mark	Conc.	Area%
1	5.635	937521	140622	M	24.644	24.644
2	5.869	907079	127463	V M	23.844	23.844
3	8.714	145482	14692	M	3.824	3.824
4	9.596	1814185	160982	M	47.688	47.688
Total		3804267	443759		100.000	100.000

Scheme S8: Monitoring irreversible isomerization of (*rac*)-**3a'**.

D. Examination for the necessity of an acidic proton at the γ-position

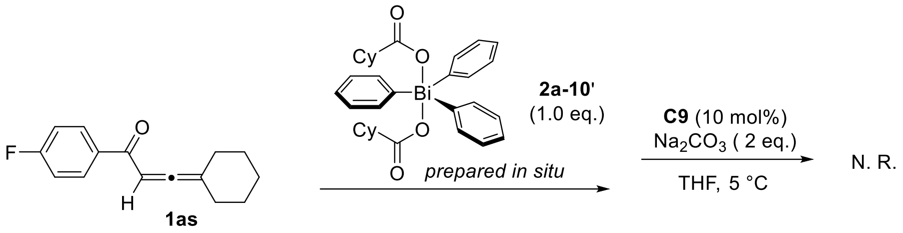

Following the General Procedure for α-Arylation of allenones with Aryl$_3$Bi(O_2CCy)$_2$ (**GP-B**), no product was detected.

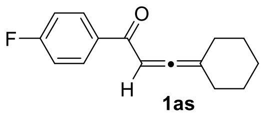

1as

3-cyclohexylidene-1-(4-fluorophenyl)prop-2-en-1-one (1as):
(**Method A**: 1:20 ethyl acetate:hexanes as eluent, Rf = 0.3, yellow oil)
^{1}H NMR (400 MHz, $CDCl_3$) δ 7.89 – 7.82 (m, 2H), 7.12 – 7.04 (m, 2H), 6.18 – 6.05 (m, 1H), 2.28 – 2.19 (m, 4H), 1.67 – 1.48 (m, 6H). ^{13}C NMR (100 MHz, $CDCl_3$) δ 209.4, 191.7, 165.2 (d, *J* = 253.2 Hz), 134.2 (d, *J* = 3.1 Hz), 131.2 (d, *J* = 9.0 Hz), 115.1 (d, *J* = 21.7 Hz), 105.7, 92.6, 30.1, 26.4, 25.6. ^{19}F NMR (377 MHz, $CDCl_3$) δ -106.7. HRMS (ESI): m/z calcd. for $C_{15}H_{16}FO^+$($[M+H]^+$) = 231.1180, found = 231.1186.

E. Evaluation of irreversible proton transfer

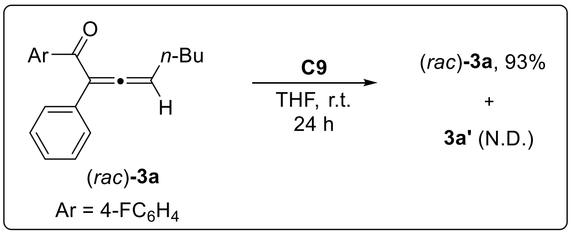

Procedure: A solution of (*rac*)-**3a** (15 mg, 0.05 mmol, 1.0 equiv.) in THF (1 mL) and **C9** (3 mg, 0.005 mmol, 10 mol%) were added to a dried tube. The reaction mixture was then stirred at room temperature for 24 h.

F. Evaluation for the stability of (*R*)-3a under basic conditions

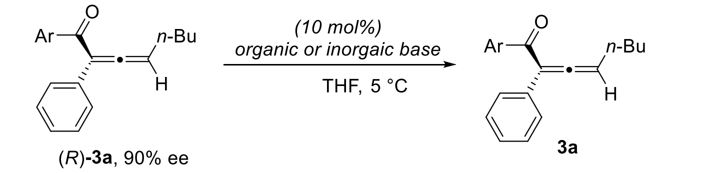

entry	base	time (h)	yield of **3a** (%)	ee of **3a** (%)
1	TMG	6	28	0
2	K_3PO_4	12	47	19
3	**C3**	24	95	88
4	Na_2CO_3	24	93	90

Procedure: To a solution of (R)-**3a** (7.5 mg, 0.025 mmol, 1.0 equiv.) in THF (0.5 mL) at 5 °C was added the base (0.00125 mmol, 10 mol%). The resulting reaction mixture was maintained at 5 °C and stirred for the indicated time. Upon completion, the reaction was processed according to the general workup procedure.

G. Exclusion of water in participation of enantioselective proton transfer process

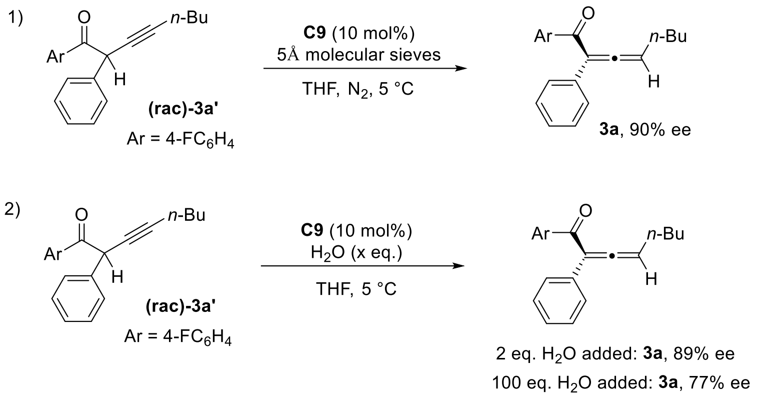

The reaction was carried out as described for the irreversible isomerization of (rac)-**3a′**, with the following modification: the reaction 1) was prepared in a glovebox.

Note: only a slight variation in ee between rigorously anhydrous conditions and with 2 equiv. of H_2O added, but a more significant decrease to 77% ee when 100 equiv. of H_2O was introduced. This trend suggests that water competes with substrate-catalyst hydrogen bonding interaction, thereby perturbing the chiral environment rather than participating in the enantioselective protonation step.

5. Calculation Studies

DFT Methods

All theoretical calculations were performed at the density functional theory (DFT) level using Gaussian 16 software package. Molecular geometries were optimized by using M06-2X functional and def2svp basis set for C, H, O, N, F and S atoms, while a LANL2DZ effective core potential was applied for Bi atom. Fully optimized geometries of all stationary points were characterized by frequency calculations to verify that the transition states (TSs) had one and only one imaginary frequency for the desired reaction coordinate. The Gibbs free energies were evaluated at 298.15 K and 1 atm. Final energies were retrieved from single-point energy calculations at M06-2X functional with dispersion energy corrections by Grimme’s dispersion correction D3 and def2tzvpp basis set for all nonmetallic atoms. The solvent effect of THF was evaluated by the IEFPCM model. All energetics throughout the text were in kcal/mol. The three-dimensional structures of the studied species were visualized using CYLview software[17].

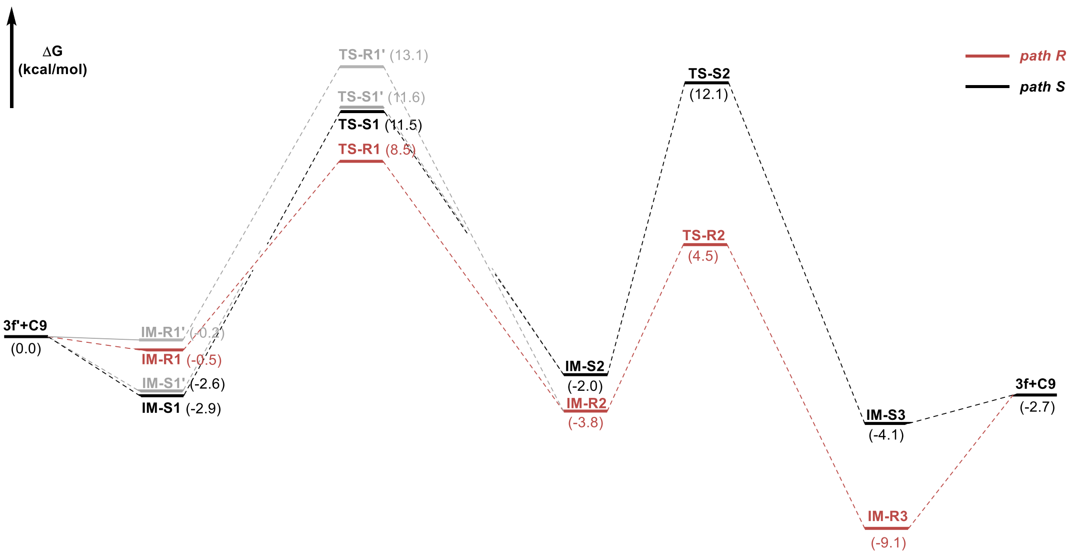

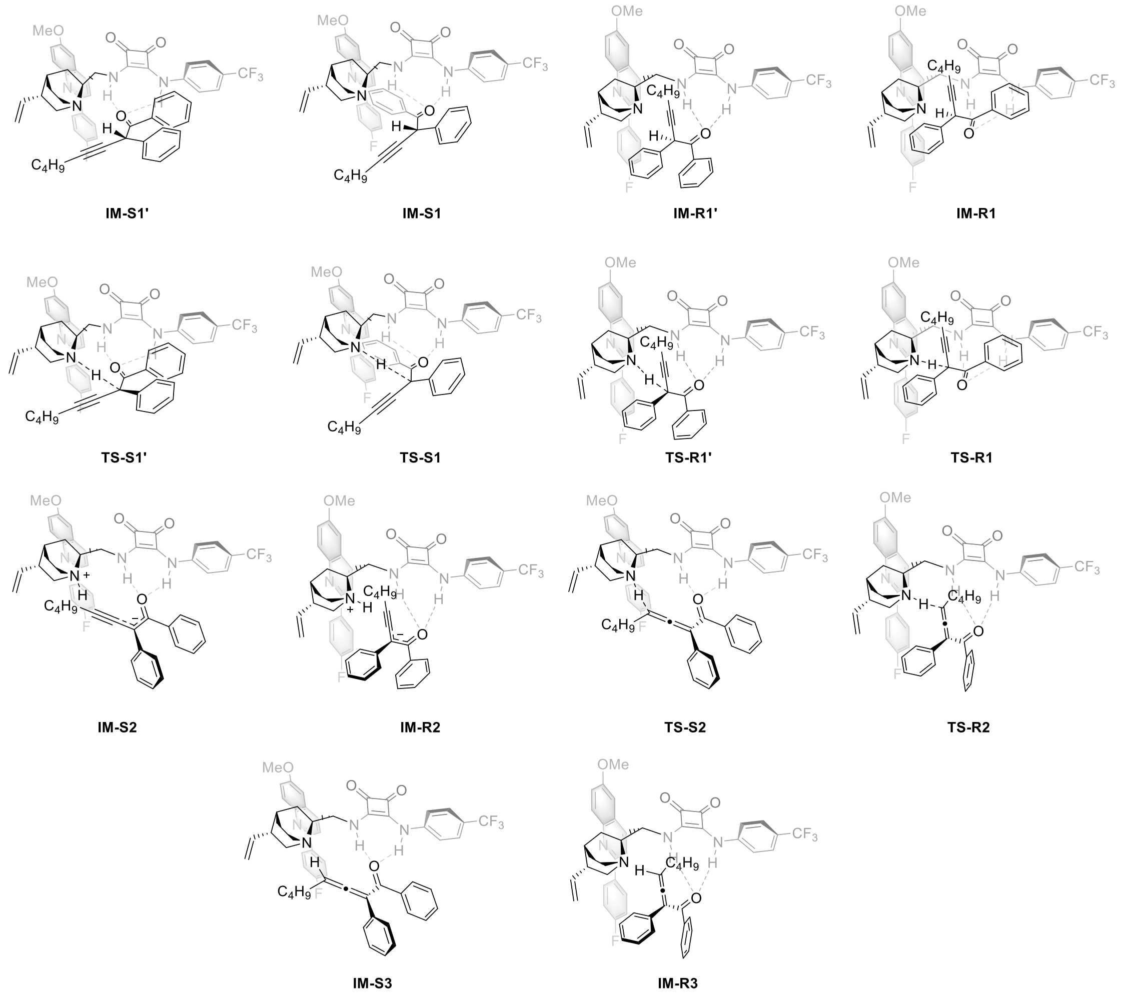

Scheme S9: Energy profiles for enantioselective proton transfer reaction between **3f'** and **3f** catalyzed by catalyst **C9** along path *R* and *S*.

Theory Level	G (**TS-S1**)	G (**TS-S1'**)	G (**TS-R1**)	G (**TS-R1**)	$^{a}\triangle\triangle G$ (kcal/mol)
**M062X-D3**	-3118.18177	-3118.18093	-3118.18547	-3118.17877	2.3
**B3LYP-D3**	-3121.93368	-3121.93355	-3121.93511	-3121.93107	0.9

[a] Gibbs free energy barrier between TS-R1 and TS-S1.

Structures	^{b}ZPE	$^{c}H_c$	$^{d}G_c$	E_z	H	G	^{e}G
**IM-S1'**	0.98324	1.04559	0.87855	-3118.09618	-3118.03383	-3118.20087	-3121.70070
**IM-S1**	0.98337	1.04556	0.87998	-3118.03852	-3118.03757	-3118.20315	-3121.70105
**IM-R1'**	0.98402	1.04602	0.88246	-3118.09686	-3118.03486	-3118.19842	-3121.69678
**IM-R1**	0.98274	1.04535	0.87640	-3118.09234	-3118.02973	-3118.19869	-3121.69755
**TS-S1'**	0.97955	1.04090	0.87891	-3118.08029	-3118.01894	-3118.18093	-3121.67802
**TS-S1**	0.97971	1.04126	0.87765	-3118.07972	-3118.01816	-3118.18177	-3121.67816
**TS-R1'**	0.97959	1.04091	0.87819	-3118.07737	-3118.01606	-3118.17877	-3121.67560
**TS-R1**	0.97878	1.03984	0.87275	-3118.07945	-3118.01744	-3118.18547	-3121.68285
**IM-S2**	0.98457	1.04672	0.88003	-3118.07887	-3118.01672	-3118.18341	-3121.68537
**IM-R2**	0.98538	1.04663	0.88673	-3118.10470	-3118.04345	-3118.20335	-3121.70227
**TS-S2**	0.97902	1.04089	0.87474	-3118.07413	-3118.01225	-3118.17841	-3121.67723
**TS-R2**	0.97913	1.04043	0.87836	-3118.09332	-3118.03202	-3118.19409	-3121.68931
**IM-S3**	0.98251	1.04526	0.87553	-3118.09563	-3118.03288	-3118.20261	-3121.70296
**IM-R3**	0.98292	1.04478	0.88070	-3118.11172	-3118.04986	-3118.21394	-3121.71097
**C9**	0.63544	0.67686	0.55557	-2270.04608	-2270.00465	-2270.12594	-2272.68829
**3f'**	0.34556	0.36625	0.29333	-848.01100	-847.99032	-848.06324	-849.00818
**3f**	0.34484	0.36538	0.29410	-848.01677	-847.99623	-848.06751	-849.01246

[b] Zero-point energy;

[c] Thermal correction to enthalpy;

[d]Thermal correction to Gibbs free energy;

[e]Gibbs free energy at the M062X-D3/def2svp(IEFPCM, THF)//M062X-D3/def2tzvpp(IEFPCM, THF) theoretical level

Table S1: Comparison of theory level, and the corrected electronic energies (E_z), enthalpies (H), Gibbs free energies (G) for all stationary points (in Hartree), obtained at M062X-D3// def2svp(IEFPCM, THF) theoretical level.

Discussion of enantiodetermining step

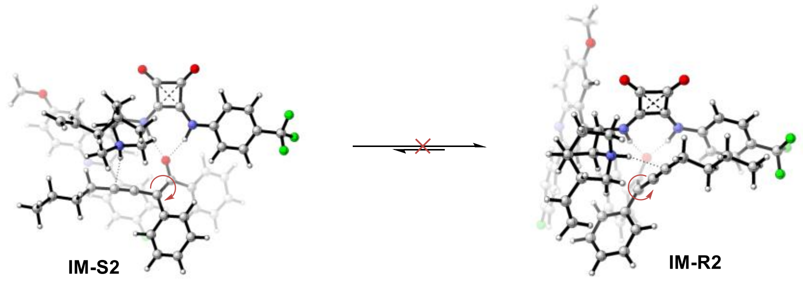

The calculated structures of **IM-R2** and **IM-S2** indicate that the enolate formed after deprotonation is essentially planar, with the proton approaching toward the alkyne π-orbital from either the Re or Si face. As the enolate is embedded in a chiral environment (quinoline sterics and squaramide H-bonding), interconversion between **IM-R2** and **IM-S2** would be difficult. If such a conformational change was feasible, all conformers should converge to **IM-R2** after deprotonation. Since deprotonation is rate-determining, it would differentiate (*R*)- and (*S*)-alkyne isomers, implying kinetic resolution. Experimentally, however, the alkyne substrate remains racemic, favoring a dynamic kinetic resolution pathway over simple stereoconvergence. Thus, **TS-1** is identified as the more likely enantiodetermining step.

BDE of triphenylbismuth(V) reagents

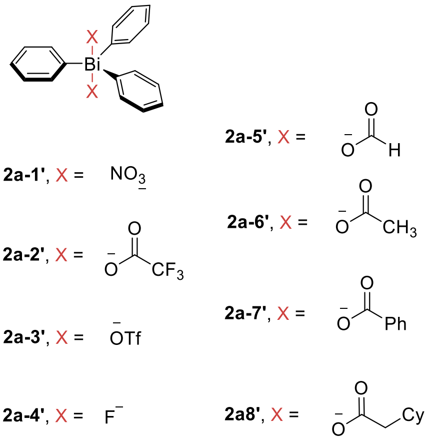

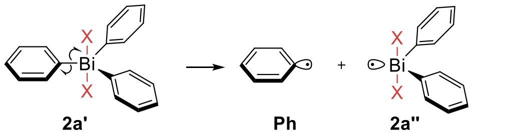

Structure	^{a}H	Structure	^{a}H
2a-1'	-1259.13483	2a-1''	-1027.84458
2a-2'	-1750.43314	2a-2''	-1519.12890
2a-3'	-2025.65034	2a-3''	-1794.34568
2a-4'	-898.70773	2a-4''	-667.39590
2a-5'	-1077.07570	2a-5''	-845.79041
2a-6'	-1155.56496	2a-6''	-924.28303
2a-7'	-1538.50938	2a-7''	-1307.22583
2a-8'	-1624.04157	2a-8''	-1392.75723
		Ph	-231.19047

[a]The corrected enthalpies (in Hartree)

Scheme S10: The corrected enthalpies (*H*) for all stationary points (in Hartree), obtained at M062X-D3// def2svp(IEFPCM, THF) theoretical level.

The Cartesian coordinates (Å) for the optimized structures

IM-S1'

C	-3.75051200	4.92277700	-1.08158600
C	-4.60786100	4.69195900	-2.19306000
C	-4.67651700	3.44118100	-2.75526200
C	-3.91435900	2.35695100	-2.24611100
C	-3.07637400	2.58491000	-1.11052800
C	-3.00211400	3.88478000	-0.55598100
N	-3.99545000	1.16155900	-2.88288400
C	-3.28354000	0.15083000	-2.44201600
C	-2.47883000	0.25709200	-1.27015100
C	-2.36377000	1.45205700	-0.60394500
C	-1.48128400	1.52439900	0.63255600
O	-3.61574200	6.12668700	-0.49273500
C	-3.32599600	-1.11818000	-3.21968600
C	-4.43314100	-1.39913800	-4.03394700
C	-4.47794300	-2.55467300	-4.80691000
C	-3.39220200	-3.42295500	-4.77223600
C	-2.27528600	-3.17187900	-3.98686400
C	-2.25179300	-2.01949600	-3.20431800
F	-3.42737700	-4.53639200	-5.50908800
N	-0.13839100	1.03352200	0.31528800
C	-2.14089600	0.78515300	1.80820400
C	0.85739100	1.91151400	0.13445100
C	2.14513300	1.86670400	-0.41800300
C	1.02025100	3.31348300	0.56212000

C	2.38968400	3.30134000	-0.09841700
O	0.34352900	4.12303400	1.15235200
O	3.26236400	4.11764800	-0.25508000
N	2.76734700	0.85051600	-1.05254800
C	4.09271200	0.75327000	-1.47496200
C	5.00793400	1.80926900	-1.37462100
C	6.32350400	1.61522200	-1.78151600
C	6.73822600	0.38272400	-2.28678100
C	5.82340700	-0.66604800	-2.39985400
C	4.50823900	-0.48243000	-1.99854100
N	-1.27942300	0.73793900	2.99728300
C	-1.15665300	2.05878900	3.62356000
C	-2.53490200	2.56208300	4.13259900
C	-3.60658800	1.56597200	3.67218000
C	-3.51413600	1.42819600	2.14897000
H	-2.29493900	-0.26229000	1.50387400
C	-1.86917800	-0.19233000	3.96622400
C	-3.32738400	0.19471200	4.32973300
C	-4.39415200	-0.80199000	3.95395600
C	-4.25800400	-1.96898300	3.32220600
C	-1.81866700	-2.78569300	0.64907000
C	-0.80436300	-2.39767200	1.18844800
C	0.35037600	-1.89780400	1.93996300
C	1.49757900	-1.44703400	1.04276700
C	0.88609300	-2.90637700	2.95127600
C	1.17989600	-4.21093700	2.54284600
C	1.70086200	-5.12960900	3.45191900
C	1.93256200	-4.74986600	4.77530700
C	1.64301300	-3.44872600	5.18499700
C	1.12328500	-2.52817000	4.27441300
C	-3.06223600	-3.21562600	0.00554400
C	-2.93708100	-4.49976500	-0.81904100
C	-4.21699600	-4.78498600	-1.59653400
C	-4.10357700	-6.00619200	-2.50038500
C	2.60075300	-0.68486000	1.69750600
O	1.51311300	-1.67809500	-0.15171100
C	2.32243000	0.33971100	2.61296700
C	3.36666600	1.12016400	3.10728400
C	4.68075600	0.86721700	2.70992400
C	4.95927200	-0.16814700	1.81452800
C	3.91935300	-0.93834300	1.30276300
H	-0.07534600	0.13125900	-0.15200700
H	2.26593600	-0.03886900	-1.05689900
H	0.03256700	-0.98626600	2.47854800
C	-4.33727300	7.22513600	-1.00391000
C	8.17494600	0.16562900	-2.65635900
F	8.89671200	-0.28257400	-1.61915300
F	8.76209000	1.29204400	-3.07007800
F	8.30906100	-0.73900700	-3.63115400
H	-5.20405200	5.50147100	-2.61134000
H	-5.31480900	3.24749800	-3.61781500
H	-2.34416500	4.11923100	0.28161100
H	-1.95990500	-0.62874300	-0.89508000
H	-1.34971300	2.57127300	0.92450100
H	-5.26043700	-0.68975500	-4.05290900
H	-5.33565600	-2.79281100	-5.43626500
H	-1.43988600	-3.87268700	-3.99816700
H	-1.36785600	-1.81975000	-2.59443700
H	4.69441200	2.77562100	-0.98121900
H	7.03500900	2.43847600	-1.70459600
H	6.13911300	-1.62799800	-2.80554900
H	3.79012300	-1.30113100	-2.07778600
H	-0.43886200	1.96646200	4.45186000
H	-0.71153100	2.75783400	2.90143300
H	-2.54512000	2.64090600	5.22984200
H	-2.75937900	3.56149100	3.73151200
H	-4.60713600	1.91949400	3.96123100
H	-4.33547100	0.81520700	1.75216600
H	-3.60788700	2.42767900	1.69561500
H	-1.23651400	-0.17928600	4.86556200
H	-1.81652500	-1.21026500	3.55311500
H	-3.40147500	0.34210700	5.42038300
H	-5.40589000	-0.49170300	4.24398900
H	-5.13354300	-2.58721200	3.11226400
H	-3.29111500	-2.35996400	2.99476900
H	0.99008600	-4.50580000	1.50780600
H	1.92615500	-6.14624700	3.12659100
H	2.33876100	-5.46956300	5.48742400
H	1.82098300	-3.14658400	6.21794500
H	0.90251100	-1.50692600	4.59315800
H	-3.83603100	-3.34153300	0.78013100
H	-3.42016900	-2.40020800	-0.64704700
H	-2.09213000	-4.40029700	-1.51859700
H	-2.69597600	-5.34350700	-0.15237000
H	-5.05018100	-4.91669100	-0.88615200
H	-4.46698900	-3.89521900	-2.20040600
H	-5.03651300	-6.18476900	-3.05347200
H	-3.29928000	-5.87481400	-3.23998000

H	-3.87734400	-6.91089400	-1.91614700
H	1.28762700	0.56341700	2.89083400
H	3.15141500	1.93694700	3.79755000
H	5.49337900	1.48257700	3.09866300
H	5.98644100	-0.36508100	1.50343200
H	4.12015200	-1.73787900	0.58732200
H	-4.06700800	8.08768400	-0.38636400
H	-4.06555700	7.43166300	-2.05076900
H	-5.42364200	7.05900400	-0.93562800

IM-S1

C	0.39370200	-5.43386000	-0.58638700
C	-0.27883500	-6.19108500	0.41092000
C	-1.39208800	-5.67174400	1.02648100
C	-1.88619000	-4.38343600	0.69766600
C	-1.19539700	-3.61649000	-0.28776600
C	-0.06318600	-4.17120100	-0.92448200
N	-3.01167900	-3.94757400	1.32256900
C	-3.48621900	-2.76468300	1.02041800
C	-2.82567400	-1.89062700	0.10182400
C	-1.69712200	-2.29885600	-0.56204500
C	-0.92208200	-1.38089700	-1.49723500
O	1.47911500	-5.88140100	-1.24554100
C	-4.74890900	-2.33158600	1.67582800
C	-5.06739600	-2.80789900	2.95455700
C	-6.22838900	-2.39538700	3.60095400
C	-7.08056000	-1.51235400	2.94552300
C	-6.80581900	-1.03637400	1.66936800
C	-5.63297400	-1.44921700	1.04133500
F	-8.19830500	-1.11637200	3.55837300
N	0.31674700	-0.98313200	-0.82571500
C	-1.75225900	-0.18016300	-1.98567500
C	1.52831700	-1.15699800	-1.36511000
C	2.84232000	-0.92562700	-0.92324500
C	1.99834300	-1.78100900	-2.61293700
C	3.41115100	-1.40368300	-2.21085100
O	1.47642000	-2.34708300	-3.54796500
O	4.49654200	-1.49367100	-2.73085900
N	3.26645700	-0.36745600	0.22468900
C	4.56420700	-0.14555300	0.68164300
C	5.70714300	-0.49417600	-0.04892700
C	6.96505100	-0.21221500	0.47371800
C	7.09693800	0.40662300	1.71688900
C	5.95710200	0.74022300	2.45282900
C	4.69877900	0.46570900	1.93980700
N	-0.95679700	0.76167500	-2.77868500
C	-0.64834800	0.22540900	-4.10945300
C	-1.94258300	0.02035800	-4.94077500
C	-3.13746400	0.26786100	-4.01485300
C	-3.00300800	-0.63844600	-2.78562900
H	-2.07303600	0.38903900	-1.09650100
C	-1.71515800	2.00553300	-2.93776900
C	-3.11429400	1.74365100	-3.56379400
C	-4.29531800	2.06570800	-2.68057300
C	-4.32935400	2.88745600	-1.62953700
C	-0.80234700	4.78277300	0.48173600
C	-0.30821000	3.69441700	0.28644000
C	0.28924000	2.37478900	0.04174300
C	0.11971900	1.42708800	1.24173300
C	1.72852200	2.47789600	-0.43156300
C	2.71703500	3.02327300	0.39656800
C	4.03332900	3.10848100	-0.04801700
C	4.37431900	2.64678300	-1.32261400
C	3.39064700	2.11580500	-2.15399600
C	2.06674300	2.03724500	-1.71212400
C	-1.47607100	6.06431800	0.70493700
C	-2.93280900	6.04018100	0.22697700
C	-3.66525300	7.35004500	0.49442700
C	-5.11094700	7.31536200	0.01283800
C	-1.24420900	1.16025000	1.78723600
O	1.07298700	0.81628100	1.68705300
C	-2.41563100	1.68422400	1.22046000
C	-3.65840700	1.35845300	1.75816600
C	-3.74101700	0.51052400	2.86419200
C	-2.58064000	-0.02590600	3.42549300
C	-1.33886300	0.29516200	2.88822200
H	0.25817100	-0.76295700	0.16449900
H	2.53621700	0.02708400	0.82210000
H	-0.27320000	1.87337000	-0.76922000
C	1.98337400	-7.16366300	-0.94596900
C	8.45414500	0.75953100	2.24601400
F	8.50387100	0.68848200	3.58060000
F	8.81234700	2.00990500	1.92027700
F	9.40580900	-0.04638800	1.76637700
H	0.07345000	-7.18401600	0.68604400
H	-1.93219000	-6.24106000	1.78358000
H	0.47351500	-3.63949700	-1.70966000
H	-3.22839300	-0.88773600	-0.03469200

H	-0.62262800	-1.96339700	-2.38201800
H	-4.38218800	-3.50331300	3.44013800
H	-6.48615300	-2.74577200	4.60063100
H	-7.50838800	-0.35770100	1.18557300
H	-5.41622300	-1.08122600	0.03677100
H	5.61323800	-0.96335400	-1.02780500
H	7.85510400	-0.48207300	-0.09613800
H	6.05494600	1.21276000	3.43117100
H	3.80193900	0.73107200	2.50327400
H	0.03387300	0.93343800	-4.60253000
H	-0.09481000	-0.71538800	-3.99834000
H	-1.98159400	0.71534500	-5.79277600
H	-1.98773300	-0.99987800	-5.34846900
H	-4.08147500	0.05635500	-4.53802700
H	-3.90973000	-0.58673300	-2.16643400
H	-2.89762000	-1.68769600	-3.10816300
H	-1.11947800	2.68167200	-3.56738600
H	-1.81109600	2.49230300	-1.95532000
H	-3.22380000	2.36053600	-4.47242600
H	-5.23292500	1.58409700	-2.98607600
H	-5.26320200	3.06950000	-1.09286300
H	-3.45064700	3.43733700	-1.28128700
H	2.45024400	3.37945700	1.39450800
H	4.80230300	3.52319200	0.60587200
H	5.40932000	2.70187200	-1.66382700
H	3.65222500	1.75516800	-3.15066100
H	1.28464600	1.60961700	-2.34593000
H	-1.43939000	6.30726400	1.77878900
H	-0.92378800	6.86286700	0.18492200
H	-2.95136400	5.81965700	-0.85302600
H	-3.46036800	5.20998900	0.72657300
H	-3.63491900	7.56739000	1.57484700
H	-3.12452200	8.17361500	-0.00032200
H	-5.62523800	8.26601000	0.20977300
H	-5.15974000	7.12266800	-1.06947800
H	-5.67363000	6.51622900	0.51872300
H	-2.36777300	2.35071200	0.36040000
H	-4.56556000	1.76346600	1.30658800
H	-4.71693900	0.24986900	3.27944500
H	-2.64848500	-0.69945500	4.28082900
H	-0.42228600	-0.11687500	3.31105900
H	2.85352400	-7.31326300	-1.59308700
H	2.29972000	-7.23490600	0.10632900
H	1.23840300	-7.94689200	-1.15583500

IM-R1’

C	6.14284200	-3.01042500	-1.95337100
C	7.01523600	-2.08793000	-2.59392200
C	6.67543000	-0.75896100	-2.66083800
C	5.46684800	-0.27255800	-2.09705000
C	4.59810000	-1.20065600	-1.44251200
C	4.95879700	-2.56712800	-1.39184800
N	5.18925900	1.05062500	-2.21890300
C	4.07556800	1.52019000	-1.70782500
C	3.16868600	0.68313300	-0.99571000
C	3.40646400	-0.66197800	-0.86255600
C	2.41329600	-1.49724000	-0.07444000
O	6.40457300	-4.32859600	-1.85774600
C	3.77912400	2.96938700	-1.87455900
C	4.82092000	3.86381900	-2.15805200
C	4.57337800	5.22332200	-2.31492000
C	3.26691000	5.68363800	-2.19336800
C	2.20821600	4.82502700	-1.92704500
C	2.47150100	3.46620200	-1.76856800
F	3.02249700	6.98781500	-2.34219000
N	1.07062500	-1.25979000	-0.59089200
C	2.53211200	-1.13961200	1.42473500
C	0.14962800	-2.21002100	-0.68579100
C	-1.20108300	-2.21940000	-1.06960100
C	0.17286700	-3.68331300	-0.56553700
C	-1.32999100	-3.66569700	-0.78441800
O	1.02123000	-4.52464600	-0.39259800
O	-2.20743500	-4.49790000	-0.73871300
N	-1.97482300	-1.18138800	-1.47128000
C	-3.35681900	-1.13146800	-1.64807500
C	-4.16487300	-2.27612900	-1.62945400
C	-5.54145300	-2.14959700	-1.79424600
C	-6.12293100	-0.89665500	-1.97684100
C	-5.31784200	0.24597700	-2.00571800
C	-3.94587500	0.13039000	-1.84643000
N	1.50839000	-1.79016600	2.24094100
C	1.83904100	-3.20072400	2.47869900
C	3.12676400	-3.34291700	3.33151100
C	3.77869900	-1.96005000	3.41501000
C	3.94854900	-1.42046000	1.98710800
H	2.34194400	-0.05444100	1.50491000
C	1.43990700	-1.09693000	3.53056500
C	2.83704900	-1.03520300	4.21665700

C	3.43951300	0.33825000	4.38005200
C	2.82753700	1.52065000	4.30159000
C	-2.46418100	-0.90615700	1.77531400
C	-1.64293900	-0.01954500	1.69295300
C	-0.63618800	1.01895600	1.45628800
C	-0.92156000	1.65919800	0.09177900
C	-0.45717700	2.06114600	2.54946800
C	-1.02266900	1.91011400	3.81606800
C	-0.78779800	2.86687500	4.80537900
C	0.00498800	3.98097400	4.53168000
C	0.57055900	4.13547400	3.26463100
C	0.34511100	3.17631400	2.28018800
C	-3.49520800	-1.94278500	1.83067400
C	-4.91026500	-1.37365800	1.67466000
C	-5.97510100	-2.46374500	1.68055400
C	-7.38235400	-1.90035800	1.52765700
C	-2.07073400	2.58341400	-0.08470800
O	-0.20948700	1.37156700	-0.85857600
C	-3.10962100	2.67489000	0.85261100
C	-4.21087100	3.48437300	0.58594100
C	-4.27034800	4.21795200	-0.60021200
C	-3.22690300	4.14651100	-1.52709600
C	-2.13244700	3.32715400	-1.27305000
H	0.78467800	-0.29288500	-0.75207900
H	-1.49045100	-0.29602300	-1.59562900
H	0.32358200	0.49213900	1.32554000
C	7.59205100	-4.83472400	-2.42536200
C	-7.60091600	-0.74893800	-2.17979900
F	-8.11673800	0.19613200	-1.38075600
F	-8.26249000	-1.88067500	-1.93024200
F	-7.90250200	-0.38417000	-3.43200700
H	7.95041400	-2.42537900	-3.03803500
H	7.32760800	-0.03776400	-3.15424300
H	4.32017700	-3.31325600	-0.91932100
H	2.28362400	1.13355700	-0.54200800
H	2.61387300	-2.56801000	-0.20588000
H	5.83386400	3.47233600	-2.25140600
H	5.37363900	5.93252000	-2.52738700
H	1.19564000	5.22336700	-1.85608000
H	1.63320200	2.79091400	-1.58683000
H	-3.72274200	-3.26083700	-1.47606700
H	-6.16769600	-3.04229100	-1.77618100
H	-5.76354300	1.23228500	-2.14780400
H	-3.32108800	1.02476400	-1.86585700
H	0.97596900	-3.66881000	2.97383000
H	1.95122300	-3.70536100	1.51181000
H	2.89674700	-3.70654500	4.34443100
H	3.81991700	-4.06434500	2.87448600
H	4.75516100	-2.01962000	3.91777400
H	4.55888000	-0.50583900	1.96901900
H	4.47631600	-2.17238400	1.37712500
H	0.71643800	-1.63289400	4.16113500
H	1.03486700	-0.08820500	3.36885200
H	2.75846000	-1.46591600	5.23014500
H	4.51074400	0.32867000	4.61932800
H	3.38497500	2.44555300	4.46568800
H	1.76070000	1.62660900	4.09061600
H	-1.64565800	1.03827000	4.02628600
H	-1.22926100	2.73923100	5.79470000
H	0.18405600	4.72855800	5.30550100
H	1.19549800	5.00173900	3.04363200
H	0.80555000	3.29386600	1.29447000
H	-3.29737900	-2.67923500	1.03188500
H	-3.40925900	-2.49124300	2.78225100
H	-5.10263800	-0.65478000	2.48836300
H	-4.96847800	-0.80382900	0.73122000
H	-5.76204900	-3.17370700	0.86386600
H	-5.90028400	-3.04241600	2.61627600
H	-8.13665500	-2.69805400	1.48133300
H	-7.63563800	-1.24305000	2.37310500
H	-7.46676800	-1.30086400	0.60924800
H	-3.07433500	2.09812100	1.77689300
H	-5.02680100	3.54180300	1.30675900
H	-5.13447600	4.85241000	-0.80293300
H	-3.27204900	4.72709900	-2.44896000
H	-1.31622700	3.24378800	-1.99228200
H	7.59195400	-5.91261100	-2.23402200
H	7.62319800	-4.66149600	-3.51234200
H	8.48320100	-4.38600800	-1.95948100

IM-R1

C	-1.01549400	-4.59332300	-2.67635100
C	-1.79059200	-5.59032200	-2.02416600
C	-2.75604300	-5.22133500	-1.11859600
C	-2.99601600	-3.85862000	-0.80540800
C	-2.20527900	-2.86053900	-1.45182700
C	-1.22768400	-3.25477900	-2.39169600
N	-3.97065800	-3.56707400	0.09516300

C	-4.21006200	-2.31296700	0.39138300	C	-3.55433800	1.80190000	3.68510300
C	-3.45138600	-1.24543300	-0.17850200	C	-3.02211100	0.73669700	2.95582500
C	-2.45303200	-1.49577800	-1.08536800	C	-1.89356500	0.93001100	2.16293700
C	-1.54099700	-0.38534400	-1.59572000	C	1.45808800	6.19976800	0.70396500
O	-0.06213600	-4.87523700	-3.58362100	C	2.67110800	6.07445900	-0.22710800
C	-5.28038800	-2.01389900	1.38246500	C	3.38261000	7.40365900	-0.45147800
C	-5.69865400	-3.01507800	2.27125700	C	4.58341100	7.27080800	-1.38087500
C	-6.67715100	-2.76188100	3.22622600	C	2.26634000	1.68465400	0.43158000
C	-7.24354900	-1.49311700	3.28280700	O	1.02405200	0.48270200	2.04293700
C	-6.86394100	-0.48074000	2.41107800	C	2.12070600	2.13161400	-0.88988700
C	-5.88069000	-0.74854400	1.46186200	C	3.24195200	2.22743000	-1.71094100
F	-8.18107700	-1.24212000	4.19892500	C	4.50635900	1.90337000	-1.21308700
N	-0.28268000	-0.46006100	-0.84142500	C	4.65440800	1.48208900	0.11000000
C	-2.23031400	0.98473500	-1.54447300	C	3.53368300	1.36620000	0.92894000
C	0.87769400	-0.79439200	-1.42239000	H	-0.36214600	-0.62537500	0.15831000
C	2.11192400	-1.25586300	-0.94550900	H	1.83587200	-1.19552700	1.04714500
C	1.31741900	-0.87559300	-2.82471200	H	-0.45380100	2.12316300	0.10000700
C	2.69764900	-1.25959700	-2.31583600	C	0.20076700	-6.22263800	-3.90700200
O	0.79639100	-0.71542000	-3.90414800	C	7.55862800	-2.79804400	2.54713800
O	3.76148800	-1.48249300	-2.83551900	F	8.30061100	-1.69035700	2.68458800
N	2.49346700	-1.49217400	0.32638900	F	8.26919100	-3.64135500	1.79282500
C	3.74852900	-1.84795700	0.81987900	F	7.45387700	-3.33900800	3.76468800
C	4.82675900	-2.18383600	-0.00896900	H	-1.63242500	-6.64576400	-2.24027900
C	6.05254100	-2.50845300	0.56201300	H	-3.36781000	-5.97017500	-0.61470300
C	6.21594900	-2.50357900	1.94754000	H	-0.62610700	-2.52768000	-2.93645000
C	5.13971700	-2.17531900	2.77412800	H	-3.66387900	-0.22988300	0.15019400
C	3.91243800	-1.84971800	2.21478700	H	-1.27861300	-0.58766500	-2.64378800
N	-1.34374000	2.10834000	-1.86376500	H	-5.23600200	-3.99945600	2.20258600
C	-1.05407100	2.16767400	-3.30153700	H	-7.00557200	-3.52741800	3.92944900
C	-2.34056600	2.48915500	-4.10751100	H	-7.34169700	0.49676800	2.47980800
C	-3.54049700	2.35462400	-3.16104700	H	-5.59879900	0.04767300	0.77072500
C	-3.47104000	0.98550200	-2.47766800	H	4.71415700	-2.17539300	-1.09246800
H	-2.54720800	1.17113900	-0.50657200	H	6.89116000	-2.77240000	-0.08370600
C	-2.02016100	3.35171000	-1.46618900	H	5.25970700	-2.17698200	3.85814600
C	-3.44253500	3.45492000	-2.07588400	H	3.06962100	-1.58352800	2.85599900
C	-4.59482800	3.34063900	-1.10765700	H	-0.28386100	2.93709200	-3.45905800
C	-4.56966400	3.02667100	0.19023100	H	-0.60981600	1.21155600	-3.61213000
C	0.81597800	4.90080400	0.91792300	H	-2.30512600	3.50929700	-4.51865800
C	0.36264400	3.78451400	1.03527700	H	-2.45177400	1.79872800	-4.95604700
C	-0.11104500	2.39916700	1.12289700	H	-4.48405600	2.45758500	-3.71607800
C	1.07385000	1.44209900	1.30019800	H	-4.38855000	0.78018600	-1.90979800
C	-1.27412700	2.18455500	2.07639700	H	-3.38087000	0.19474400	-3.24145500
C	-1.80925500	3.24023600	2.82016400	H	-1.39328100	4.18899800	-1.80456700
C	-2.93972200	3.05035800	3.61851000	H	-2.05741000	3.41337200	-0.36844600

H	-3.54750100	4.42613000	-2.58665100
H	-5.57390200	3.51882500	-1.56955300
H	-5.50012400	2.96785000	0.76009600
H	-3.64822700	2.83100700	0.74714100
H	-1.34367200	4.22480400	2.76180300
H	-3.34349500	3.89032700	4.18566400
H	-4.44199700	1.65457000	4.30217100
H	-3.48815800	-0.24986900	3.00115100
H	-1.49621600	0.08120300	1.60757900
H	0.72891800	6.90674100	0.27747000
H	1.77135400	6.61916900	1.67314700
H	3.37442900	5.33893700	0.19789100
H	2.33909100	5.65977900	-1.19333700
H	2.66648600	8.13242100	-0.86618000
H	3.70535200	7.80969200	0.52149600
H	5.08857600	8.23519600	-1.52901300
H	5.31939800	6.56276800	-0.97121100
H	4.27569600	6.89537100	-2.36840400
H	1.12657800	2.35772800	-1.28661500
H	3.12804800	2.54833400	-2.74750800
H	5.38074000	1.97781500	-1.86123400
H	5.64181700	1.23068200	0.50027800
H	3.63170000	1.01541900	1.95809900
H	1.00465500	-6.21143300	-4.64988500
H	0.53350600	-6.78913500	-3.02341100
H	-0.68538400	-6.71146300	-4.34081500

TS-S1'

C	-4.18924300	4.84962100	-0.29021200
C	-5.24301200	4.65998000	-1.22729200
C	-5.37417400	3.45734100	-1.87667600
C	-4.48266400	2.38046900	-1.62873900
C	-3.44243800	2.56580400	-0.66510300
C	-3.30845000	3.81777700	-0.01918500
N	-4.64679400	1.23419300	-2.33542500
C	-3.82041800	0.23303300	-2.13754800
C	-2.79303600	0.29697100	-1.15245500
C	-2.60006900	1.43866300	-0.41549900
C	-1.49466900	1.44598000	0.63078500
O	-3.98749600	6.00823200	0.36582400
C	-3.98529600	-0.98549100	-2.97671300
C	-5.22496400	-1.25596500	-3.57472400
C	-5.39891200	-2.37130300	-4.38740800
C	-4.31191000	-3.20912100	-4.61196900
C	-3.06716300	-2.96546000	-4.04740500
C	-2.91176600	-1.85403600	-3.22222400
F	-4.47149500	-4.28597500	-5.38566200
N	-0.21966200	1.09748200	0.02279100
C	-1.89492500	0.50871800	1.78620900
C	0.69236200	2.04477300	-0.21160400
C	1.94013000	2.09727900	-0.85197100
C	0.81852000	3.43020100	0.27822700
C	2.11021500	3.53834600	-0.51848000
O	0.16762600	4.14492000	1.00639700
O	2.91024500	4.41500400	-0.73143200
N	2.58806700	1.13259300	-1.53814400
C	3.95060000	1.05319000	-1.83164300
C	4.83655000	2.12408900	-1.65300500
C	6.19603300	1.93131100	-1.87413500
C	6.67902800	0.68644500	-2.27678800
C	5.79122700	-0.36912900	-2.49447300
C	4.43415000	-0.18669000	-2.27905200
N	-0.74501700	0.14401400	2.66022900
C	-0.08399200	1.34455800	3.21572500
C	-1.13908100	2.27178800	3.85932400
C	-2.44503700	1.48203900	4.02131100
C	-3.01670100	1.15856500	2.63803400
H	-2.23905500	-0.43983300	1.35742000
C	-1.26362300	-0.68991900	3.77438100
C	-2.12722700	0.15188100	4.73689200
C	-3.37008900	-0.53291100	5.24070700
C	-3.76621100	-1.78569200	5.01386300
C	-1.72467100	-2.82864800	0.52996200
C	-0.63554400	-2.44100500	0.90482000
C	0.60656800	-1.91020900	1.44274600
C	1.54685400	-1.47149100	0.40764400
C	1.15724200	-2.64187500	2.64568200
C	0.80588300	-3.97384100	2.89843100
C	1.29173000	-4.64237900	4.02252800
C	2.13896700	-3.99129200	4.91798200
C	2.49170700	-2.66183100	4.68127200
C	2.00142400	-1.99421800	3.56081800
C	-3.06236300	-3.23798500	0.09107300
C	-3.08807500	-4.45442200	-0.83888200
C	-4.48634500	-4.70459900	-1.39228100
C	-4.53847200	-5.86736700	-2.37548300
C	3.00009100	-1.25217600	0.73482800
O	1.17920100	-1.21734100	-0.74501900

C	3.44428800	-0.02031300	1.22305900
C	4.80958600	0.21551600	1.38239000
C	5.73307600	-0.78353700	1.07138700
C	5.28737600	-2.02344800	0.61270200
C	3.92416100	-2.25768700	0.43719000
H	-0.11075000	0.18515300	-0.42527900
H	2.10998600	0.22440400	-1.53613200
H	0.09571700	-0.75077800	1.97101000
C	-4.83844900	7.10231900	0.10540300
C	8.15258900	0.44845200	-2.40150200
F	8.66773500	-0.07825600	-1.27883600
F	8.83005500	1.57462100	-2.63926400
F	8.44165000	-0.40941000	-3.38564100
H	-5.94265100	5.46552000	-1.44456700
H	-6.16565300	3.29856800	-2.60964700
H	-2.49751900	4.02260000	0.68118500
H	-2.16729100	-0.58074000	-0.96874300
H	-1.36905700	2.45646400	1.03449600
H	-6.05332000	-0.57177200	-3.39119800
H	-6.35874200	-2.60210400	-4.84994000
H	-2.23740000	-3.64069800	-4.25889700
H	-1.92879100	-1.65979400	-2.78787300
H	4.46618100	3.09814400	-1.33470700
H	6.88811700	2.76068100	-1.72330300
H	6.16411700	-1.34142400	-2.82029400
H	3.73683200	-1.01495100	-2.41898300
H	0.65107600	0.99102900	3.95206400
H	0.47075200	1.85417700	2.41779900
H	-0.78359100	2.62846000	4.83551700
H	-1.30424300	3.15980100	3.22996500
H	-3.17196700	2.06115300	4.60752600
H	-3.87341300	0.47680000	2.73359600
H	-3.38512800	2.07553200	2.15476300
H	-0.40690900	-1.14131500	4.28953200
H	-1.83430400	-1.50413700	3.30718600
H	-1.52510800	0.40397900	5.62738100
H	-3.99581300	0.10079800	5.88028000
H	-4.69207000	-2.16103800	5.45354600
H	-3.19492200	-2.48978400	4.40453400
H	0.13855800	-4.48796900	2.20454900
H	1.00507100	-5.68082100	4.19695300
H	2.51788700	-4.51322500	5.79777800
H	3.14612900	-2.13563700	5.37806700
H	2.27291700	-0.94930000	3.40088500
H	-3.68392900	-3.43608300	0.97986600
H	-3.54052000	-2.38542300	-0.42432900
H	-2.38493500	-4.28784200	-1.67052000
H	-2.72566300	-5.34179200	-0.29458400
H	-5.18025900	-4.88789000	-0.55477800
H	-4.83890100	-3.78202300	-1.88595300
H	-5.55299300	-6.01324800	-2.77285700
H	-3.86981500	-5.68957500	-3.23128700
H	-4.22432200	-6.80613900	-1.89471500
H	2.72291100	0.76542200	1.46652400
H	5.15229400	1.18588300	1.74482700
H	6.80190700	-0.59312700	1.17976900
H	6.00657600	-2.80860800	0.37447900
H	3.57443300	-3.22162500	0.06144200
H	-4.48323800	7.92670600	0.73197800
H	-4.78949400	7.40578000	-0.95185400
H	-5.88214500	6.87192000	0.37029500

TS-S1

C	0.59500000	-5.03507500	-2.25552800
C	0.17578000	-6.12856000	-1.45075000
C	-0.80244800	-5.94225200	-0.50382800
C	-1.40218900	-4.67354400	-0.29810500
C	-0.96170900	-3.57198500	-1.09211200
C	0.02967400	-3.78358500	-2.07470100
N	-2.37774100	-4.56860300	0.64204600
C	-2.95531600	-3.40818000	0.82989400
C	-2.55355700	-2.23516000	0.11822200
C	-1.55856100	-2.29420700	-0.82358100
C	-0.98914600	-1.04231900	-1.48613500
O	1.53205500	-5.14116800	-3.21574800
C	-4.03361300	-3.31894500	1.85021800
C	-4.03940300	-4.20994500	2.93296800
C	-5.02040500	-4.13244600	3.91582900
C	-6.00938700	-3.16146400	3.79733900
C	-6.04629900	-2.27685100	2.72737800
C	-5.04982800	-2.35891000	1.75774100
F	-6.95419100	-3.08144400	4.73697100
N	0.28127800	-0.71921300	-0.85514200
C	-1.97668000	0.13370200	-1.41914200
C	1.47296600	-0.80826300	-1.45339100
C	2.78076000	-0.59481500	-0.99244000
C	1.92529900	-1.03084700	-2.83063600
C	3.35506500	-0.92210400	-2.32661500

O	1.37504500	-1.20803700	-3.89754600
O	4.44698800	-1.02677700	-2.82855500
N	3.17076500	-0.23205600	0.24072100
C	4.45715400	-0.09771800	0.75608200
C	5.61138600	-0.34288300	0.00182200
C	6.86153200	-0.16420900	0.58580700
C	6.97274400	0.25023000	1.91266800
C	5.82066100	0.48108700	2.66978900
C	4.57059700	0.30845400	2.09647300
N	-1.35945900	1.41698700	-1.85497200
C	-0.82845300	1.32196900	-3.23615900
C	-1.92291900	0.77156800	-4.17729400
C	-3.27172500	0.81748700	-3.44896300
C	-3.23809500	-0.15901200	-2.27087500
H	-2.24288300	0.31131500	-0.36731600
C	-2.40190700	2.47842200	-1.82324500
C	-3.47969800	2.24453700	-2.89868900
C	-4.89767300	2.46976700	-2.44260700
C	-5.34149300	2.71866000	-1.20941000
C	-0.99442400	4.88850300	0.24335900
C	-0.49611100	3.78071500	0.28279200
C	0.14494000	2.48011700	0.24259000
C	-0.09518700	1.49413700	1.29715000
C	1.60313500	2.61772900	-0.15299800
C	2.55765100	2.98120900	0.81066400
C	3.89584000	3.14570900	0.46615300
C	4.31105500	2.94274300	-0.85297000
C	3.37304500	2.59669600	-1.82183200
C	2.02745800	2.44710700	-1.47201500
C	-1.75384000	6.14091400	0.16616600
C	-3.24604400	5.87707700	-0.07721000
C	-4.07253500	7.15299900	-0.18669400
C	-5.54920600	6.86671300	-0.43351600
C	-1.44613800	1.29797500	1.95344900
O	0.77300200	0.65026500	1.57631900
C	-2.52852900	2.18897000	1.87425300
C	-3.75108300	1.87347900	2.46859800
C	-3.91664100	0.66835600	3.14997800
C	-2.84522300	-0.22131200	3.24669900
C	-1.62313800	0.09510800	2.65997600
H	0.28231600	-0.60347200	0.15871600
H	2.40864600	0.09369400	0.85081700
H	-0.53688200	1.82915500	-0.88251500
C	2.14856100	-6.38991400	-3.43811000
C	8.31753400	0.49371300	2.52804800
F	8.36098500	0.09205600	3.80360200
F	8.64052600	1.79498700	2.53266200
F	9.29451500	-0.14159500	1.87563000
H	0.61500900	-7.11617000	-1.58248200
H	-1.14916600	-6.76999100	0.11551500
H	0.36789300	-2.98744700	-2.73675900
H	-3.03065400	-1.29292800	0.38250400
H	-0.77709700	-1.26083100	-2.54385900
H	-3.25357200	-4.96301300	2.99486700
H	-5.03251100	-4.80828700	4.77111900
H	-6.84928700	-1.54210200	2.66456400
H	-5.08328300	-1.67076300	0.91112800
H	5.53221400	-0.65456100	-1.03975000
H	7.76060800	-0.35279100	-0.00188100
H	5.90257900	0.79412300	3.71186500
H	3.66474900	0.49422000	2.67713500
H	-0.51764500	2.33497600	-3.52505100
H	0.06299700	0.68492300	-3.24608000
H	-1.96259300	1.36990300	-5.09717200
H	-1.68898000	-0.26225300	-4.47286000
H	-4.08747500	0.55410100	-4.13605300
H	-4.14945300	-0.05584500	-1.66407400
H	-3.21011600	-1.19427300	-2.64106700
H	-1.89013900	3.43776000	-1.95874900
H	-2.81530600	2.47300500	-0.80716400
H	-3.30260300	2.93975600	-3.73639800
H	-5.63433500	2.40210400	-3.25132200
H	-6.40912200	2.85126200	-1.02396100
H	-4.68326300	2.80469600	-0.34125700
H	2.23767700	3.13073000	1.84464500
H	4.62447500	3.41920000	1.23148500
H	5.36276400	3.05543900	-1.12083500
H	3.68400400	2.44021600	-2.85648400
H	1.30364600	2.18034700	-2.24192100
H	-1.62583800	6.72218700	1.09357700
H	-1.35109700	6.76557500	-0.64746300
H	-3.36275400	5.28233400	-0.99915700
H	-3.64017000	5.25372600	0.74436800
H	-3.95284900	7.74312100	0.73686200
H	-3.66953300	7.77471100	-1.00320700
H	-6.13381600	7.79346700	-0.51448900
H	-5.68720000	6.29618700	-1.36455500
H	-5.97517500	6.26945100	0.38709100

H	-2.42094200	3.14045900	1.36017400
H	-4.57747000	2.58349000	2.40321800
H	-4.87571100	0.42210300	3.60858200
H	-2.96745200	-1.16860500	3.77596800
H	-0.78138900	-0.59331700	2.73322200
H	2.87120700	-6.23866500	-4.24627200
H	2.67986900	-6.74020900	-2.53963700
H	1.41458900	-7.15040200	-3.74705600

TS-R1'

C	-3.55478200	5.22783000	0.05154700
C	-4.57360900	5.34629500	-0.93282300
C	-4.99650600	4.23234300	-1.61589100
C	-4.44121500	2.95207600	-1.35965400
C	-3.42538800	2.83450700	-0.36015600
C	-2.99378400	3.99248600	0.32786700
N	-4.90271900	1.89855000	-2.07997900
C	-4.40594600	0.70423700	-1.86273300
C	-3.39023100	0.48413900	-0.88594200
C	-2.90123300	1.52117600	-0.13260000
C	-1.76786300	1.26903300	0.85465500
O	-3.08442100	6.27676500	0.75289500
C	-4.92512500	-0.42696600	-2.68011900
C	-5.56047400	-0.15914400	-3.90175300
C	-6.05376000	-1.18955300	-4.69406400
C	-5.91512900	-2.49982900	-4.24967000
C	-5.30389100	-2.80266800	-3.04008400
C	-4.80803000	-1.75968100	-2.26057400
F	-6.38708500	-3.49438700	-5.00439200
N	-0.51701700	1.08010800	0.14265100
C	-2.13566900	0.10353500	1.79491300
C	0.46489500	1.97769300	0.19290700
C	1.75464100	2.03100000	-0.35436600
C	0.68439900	3.20438300	0.97768500
C	2.03925800	3.33413700	0.29895900
O	0.05835000	3.81005400	1.81983900
O	2.94950400	4.12656100	0.35092800
N	2.34384200	1.13293000	-1.16783300
C	3.67919000	1.03868800	-1.54623300
C	4.61450200	2.05442000	-1.30932000
C	5.94453100	1.85992200	-1.67081100
C	6.34867700	0.66936000	-2.27193400
C	5.41189500	-0.33727100	-2.52817400
C	4.08595000	-0.15546300	-2.16939800
N	-0.96595500	-0.44249800	2.52806200
C	-0.23616000	0.62054800	3.26246700
C	-1.23679200	1.46660000	4.08224600
C	-2.55503600	0.68784500	4.18830500
C	-3.21091700	0.58071600	2.80526000
H	-2.51253200	-0.74052600	1.19853100
C	-1.46283500	-1.43170300	3.52070700
C	-2.21619200	-0.73532300	4.67159500
C	-3.41899300	-1.49200300	5.16930600
C	-3.76260800	-2.74633200	4.87436500
C	2.77687500	-0.39712100	1.52082400
C	1.85963700	-1.14421300	1.24431800
C	0.71725000	-2.00303000	0.97826700
C	0.38949500	-2.08443100	-0.46114900
C	0.61010300	-3.24025300	1.83577300
C	1.65944100	-3.64945000	2.66622900
C	1.52025500	-4.75841200	3.50490700
C	0.32956200	-5.48050400	3.52777900
C	-0.72668600	-5.08205700	2.70340200
C	-0.58841200	-3.97304100	1.87595500
C	3.90417800	0.48525800	1.83971900
C	5.27144200	-0.08177600	1.44094800
C	6.41073600	0.87763300	1.76508600
C	7.76866400	0.33710300	1.33419700
C	-0.18515300	-3.31572300	-1.10176800
O	0.55293900	-1.11662000	-1.21543800
C	0.38035000	-4.58431300	-0.92597400
C	-0.12507300	-5.67480200	-1.63097800
C	-1.20571000	-5.51074800	-2.49934900
C	-1.76341600	-4.24531200	-2.68869800
C	-1.24053100	-3.14883700	-2.00680800
H	-0.39902700	0.28629200	-0.49009400
H	1.79613800	0.27487200	-1.29436400
H	-0.18634000	-1.18716800	1.61297400
C	-3.60441900	7.56211000	0.49675000
C	7.77691700	0.44910600	-2.67035500
F	8.23989400	-0.72459900	-2.21682500
F	8.58676100	1.39970100	-2.19911900
F	7.93178000	0.42299500	-3.99987900
H	-5.02031800	6.31408800	-1.15528500
H	-5.77304100	4.30187200	-2.37814700
H	-2.19513300	3.96615200	1.07041500
H	-2.98297100	-0.51988800	-0.76548200
H	-1.62459700	2.15929400	1.47647300

H	-5.65827300	0.87812200	-4.22139800
H	-6.54139500	-0.99643100	-5.64977100
H	-5.23165700	-3.84196700	-2.71861600
H	-4.35048800	-2.00346700	-1.30074100
H	4.30767000	2.98368600	-0.82758900
H	6.67556900	2.64432000	-1.47189700
H	5.72388500	-1.27164600	-2.99803900
H	3.35091000	-0.94246300	-2.34865700
H	0.49387800	0.10895000	3.90414100
H	0.33810000	1.22584000	2.55461500
H	-0.83043400	1.66009300	5.08438800
H	-1.39587000	2.44566500	3.60574400
H	-3.23773900	1.17892800	4.89545600
H	-4.05373000	-0.12427400	2.84307600
H	-3.61736200	1.55497100	2.49330400
H	-0.60619600	-2.01530600	3.88111200
H	-2.12652900	-2.11567600	2.97739600
H	-1.53468000	-0.62783700	5.53399800
H	-4.04389400	-0.93110300	5.87419100
H	-4.64960700	-3.19955800	5.32061600
H	-3.17514100	-3.37718300	4.20193300
H	2.59476700	-3.08779300	2.65605500
H	2.35350300	-5.05691400	4.14328300
H	0.22023600	-6.34518100	4.18371700
H	-1.66820200	-5.63424600	2.71358700
H	-1.42944300	-3.66395300	1.25020600
H	3.75889300	1.45717900	1.33646600
H	3.89296300	0.70363900	2.92049000
H	5.42999900	-1.04421700	1.95510700
H	5.27076600	-0.29916600	0.35983200
H	6.21632000	1.84197400	1.26561200
H	6.41516100	1.08835200	2.84770800
H	8.57667200	1.05012600	1.54941100
H	8.00057800	-0.60442300	1.85463200
H	7.78092400	0.12722600	0.25400200
H	1.22146100	-4.71778800	-0.24445100
H	0.32667800	-6.65892400	-1.50004200
H	-1.60608000	-6.36986000	-3.03968900
H	-2.59573900	-4.10769100	-3.38071500
H	-1.64010300	-2.14751000	-2.18077200
H	-3.07515100	8.24911500	1.16476600
H	-3.42784600	7.86577500	-0.54682800
H	-4.68338400	7.60922600	0.71182300

TS-R1

C	3.38075700	-1.87633600	4.59752300
C	4.34780800	-2.90222200	4.41236600
C	4.72907900	-3.26015100	3.14266600
C	4.18336800	-2.62264900	1.99821400
C	3.22327100	-1.58009600	2.18809100
C	2.83283600	-1.23059800	3.50245300
N	4.59349100	-3.04273400	0.77521000
C	4.09684500	-2.47775400	-0.29970700
C	3.16565700	-1.40055900	-0.21465600
C	2.73038300	-0.94404700	1.00383300
C	1.73473000	0.20716200	1.05696400
O	2.94824000	-1.48313800	5.81045200
C	4.55843100	-2.97446300	-1.62454200
C	5.79014600	-3.63767900	-1.72309200
C	6.25261000	-4.10928400	-2.94660300
C	5.46580400	-3.91936000	-4.07730800
C	4.23437200	-3.28037000	-4.01557100
C	3.78676200	-2.80989600	-2.78313500
F	5.90463500	-4.36907100	-5.25487900
N	0.44429400	-0.19218400	0.50500600
C	2.31636700	1.43918100	0.35354800
C	-0.53770700	-0.59821800	1.32516600
C	-1.74689100	-1.28650700	1.16211500
C	-0.79197900	-0.34543900	2.74933100
C	-2.04807500	-1.19406700	2.62249100
O	-0.23799300	0.28270300	3.62435100
O	-2.89587900	-1.57835400	3.38651400
N	-2.27317200	-1.80944700	0.03866100
C	-3.49492100	-2.45695200	-0.14331900
C	-4.36566800	-2.75838200	0.91231400
C	-5.58288700	-3.37213900	0.64038000
C	-5.94267300	-3.69098800	-0.67001000
C	-5.06903600	-3.40542000	-1.72040200
C	-3.85034700	-2.79520500	-1.45880700
N	1.30911900	2.51804400	0.20201200
C	0.74564100	2.92672100	1.50766700
C	1.88661400	3.24344700	2.49919200
C	3.19396300	3.35632600	1.70535500
C	3.54857300	1.98187800	1.12721200
H	2.60154600	1.16501200	-0.67211800
C	1.97813000	3.67861400	-0.43574200
C	2.96880500	4.34406700	0.54045900
C	4.26137800	4.79366700	-0.08825300

C	4.58989600	4.77881900	-1.38064800
C	-1.71164800	4.57773000	-1.37635800
C	-1.18741400	3.50690300	-1.60455400
C	-0.53879800	2.21929600	-1.78618800
C	-1.40975000	1.04992300	-1.59086200
C	0.51724000	2.16054600	-2.85993700
C	0.80815600	3.29621100	-3.63205700
C	1.87184400	3.30638000	-4.53320700
C	2.67791100	2.17878700	-4.68970000
C	2.39135400	1.03514300	-3.94487600
C	1.32478300	1.02408800	-3.04601000
C	-2.41320600	5.81159800	-1.00970000
C	-3.32305800	5.59724500	0.20633100
C	-4.07765200	6.85782900	0.61225700
C	-4.97873900	6.63277400	1.82091500
C	-2.67767900	1.20527400	-0.79654600
O	-1.12378100	-0.08258400	-1.99270300
C	-2.70468900	1.85469100	0.44275500
C	-3.86574600	1.84307100	1.21322500
C	-5.01530700	1.20861200	0.73711900
C	-5.00166000	0.58490400	-0.51112300
C	-3.83269300	0.57468100	-1.26963800
H	0.40972400	-0.50150700	-0.46479600
H	-1.82466900	-1.49034100	-0.82751700
H	0.28509600	2.20226300	-0.70754200
C	3.45110000	-2.12607800	6.96046800
C	-7.28843900	-4.29162900	-0.94608500
F	-8.24406100	-3.35649100	-1.04407200
F	-7.67245400	-5.12419200	0.02651700
F	-7.30649900	-4.97797500	-2.09211900
H	4.78536600	-3.41365800	5.26832700
H	5.46192800	-4.05043500	2.97781800
H	2.07263400	-0.47495800	3.70408500
H	2.82410100	-0.92196700	-1.13422900
H	1.54363300	0.47380700	2.10235300
H	6.38315600	-3.77511700	-0.81906800
H	7.21177200	-4.61884700	-3.04063100
H	3.64003400	-3.16692700	-4.92230000
H	2.80542000	-2.33597200	-2.73294800
H	-4.09948000	-2.50306000	1.93759800
H	-6.26055600	-3.60564900	1.46299300
H	-5.33952700	-3.66367300	-2.74475900
H	-3.16408800	-2.56738300	-2.27692800
H	0.11200800	3.80399700	1.31208100
H	0.09610500	2.12689000	1.88678500
H	1.67699800	4.18093300	3.03170700
H	1.97047900	2.45100300	3.25897200
H	4.00625400	3.71526800	2.35251900
H	4.41752500	2.06236800	0.45911000
H	3.82598000	1.29272500	1.93935600
H	1.19887400	4.37154400	-0.78105200
H	2.49167700	3.28722100	-1.32381100
H	2.49856400	5.24465000	0.97313400
H	4.98979100	5.19107500	0.62840700
H	5.56307000	5.14966800	-1.70764000
H	3.91736200	4.41721000	-2.16283900
H	0.19706600	4.19117200	-3.50797800
H	2.07239000	4.20751300	-5.11544100
H	3.51578200	2.18843400	-5.38794600
H	3.00483100	0.13930100	-4.06088700
H	1.10844600	0.11324500	-2.49087100
H	-1.67996700	6.60451400	-0.78972600
H	-3.01295500	6.17056500	-1.86157100
H	-4.03762200	4.78865200	-0.01983600
H	-2.71228400	5.23800300	1.05224100
H	-3.35350500	7.66085900	0.82870700
H	-4.67912500	7.20925800	-0.24241300
H	-5.52018500	7.54768200	2.09855600
H	-5.72304200	5.84912300	1.61380900
H	-4.39128300	6.31023900	2.69382000
H	-1.80933900	2.35647100	0.81214500
H	-3.87300700	2.33078200	2.18930000
H	-5.92314200	1.20038600	1.34206500
H	-5.89721900	0.08601700	-0.88614100
H	-3.80442500	0.06220500	-2.23317400
H	2.95557400	-1.65929000	7.81772900
H	3.21952800	-3.20246900	6.95182700
H	4.53957300	-1.98849000	7.05391000

IM-S2

C	6.03848900	-2.36297000	-1.44603800
C	6.81178300	-1.20430200	-1.72745800
C	6.18594100	0.00168400	-1.93475300
C	4.77434300	0.12768300	-1.86541500
C	4.00212700	-1.03471000	-1.56806800
C	4.65810700	-2.27083700	-1.37226700
N	4.22463700	1.35296100	-2.06729600
C	2.92248200	1.49275200	-2.00462800

C	2.06219100	0.38391100	-1.73242500	C	-0.44794200	4.10508900	2.57525100
C	2.58026800	-0.85882800	-1.48604500	C	-2.55961400	2.36246400	-0.69543300
C	1.69301100	-2.00838800	-1.04001600	O	-1.02557900	0.56666800	-0.70622900
O	6.57647600	-3.57940900	-1.24229600	C	-3.36627300	1.68851800	-1.62385200
C	2.35043700	2.86021600	-2.15260700	C	-4.40852400	2.33722600	-2.28476700
C	3.21601100	3.95500800	-2.29824000	C	-4.64481000	3.69115200	-2.05517000
C	2.72399600	5.25152400	-2.40144500	C	-3.81664700	4.39139400	-1.17500500
C	1.34851300	5.44885400	-2.35703000	C	-2.78414500	3.73784000	-0.50741500
C	0.46047600	4.39052300	-2.21751900	C	7.97639800	-3.73399700	-1.33063800
C	0.96697300	3.09749200	-2.11490800	C	-8.32271300	-1.77783300	0.37191600
F	0.86849900	6.69153700	-2.45156300	F	-8.83881900	-3.00658400	0.46750400
N	0.29959000	-1.73356000	-1.29918800	F	-9.02141500	-1.14257900	-0.57861500
C	1.95408800	-2.31420900	0.45673300	F	-8.58079200	-1.14410300	1.52041300
C	-0.59329800	-2.69909500	-1.52642500	C	6.22045000	1.90278400	3.86078000
C	-1.98145000	-2.74199500	-1.36650900	C	5.55140000	1.67634900	2.51073000
C	-0.52051700	-4.12331900	-1.89234700	C	4.03967700	1.87079100	2.55884000
C	-2.04786600	-4.15281900	-1.84314400	C	3.39521200	1.65466800	1.18629000
O	0.37083300	-4.91378700	-2.08686900	H	7.89738300	-1.26265200	-1.78642200
O	-2.89364200	-4.98155500	-2.06639500	H	6.76170700	0.90025900	-2.15841600
N	-2.78161400	-1.75680700	-0.90141000	H	4.10785800	-3.19241600	-1.17687800
C	-4.14127500	-1.81357900	-0.59192800	H	0.99084300	0.55761800	-1.68142500
C	-4.68840300	-0.75542400	0.15265200	H	1.96623300	-2.92833600	-1.58165600
C	-6.03797400	-0.75087600	0.47418900	H	4.28942800	3.76952500	-2.32624700
C	-6.85464000	-1.80773000	0.06715300	H	3.38625700	6.11028200	-2.51295100
C	-6.31013500	-2.86886100	-0.65651900	H	-0.61282200	4.58343100	-2.18590700
C	-4.96052600	-2.87815200	-0.99191500	H	0.25355800	2.28042500	-2.00081900
N	1.39222500	-1.26577000	1.39072600	H	-0.07795600	-0.81006000	-1.02655900
C	-0.05040800	-1.46853600	1.77979300	H	3.03861800	-2.23787000	0.60398900
C	-0.17609700	-2.77620300	2.57817800	H	-2.29828500	-0.86564100	-0.71100300
C	1.14157000	-3.54895800	2.45959100	H	-4.04493300	0.06198300	0.48224800
C	1.48089800	-3.67853400	0.96702000	H	-6.45457800	0.07346000	1.05437100
C	2.21870400	-1.29714700	2.64309400	H	-6.94498500	-3.70104700	-0.96341100
C	2.23494400	-2.74493300	3.20221700	H	-4.54235700	-3.71401900	-1.55291300
C	3.59713500	-3.38180300	3.11535300	H	1.46002100	-0.30802400	0.98527500
C	4.23222900	-3.91783700	4.15609200	H	-0.32607200	-0.57542700	2.35545600
C	1.92834300	1.72349900	1.18278500	H	-0.64724100	-1.45952100	0.86683400
C	0.71347100	1.84386300	1.10590800	H	-1.00392800	-3.37424600	2.17484300
C	-0.67592300	2.15197300	1.00492200	H	-0.40021200	-2.56668600	3.63275000
C	-1.38925300	1.62752300	-0.08289200	H	1.04737800	-4.54109300	2.91782300
C	-1.22613800	3.07065400	2.02739400	H	2.27058900	-4.41943200	0.78156000
C	-2.53418800	2.90445800	2.51877800	H	0.59085600	-4.02031700	0.41990200
C	-3.05813500	3.76534300	3.47906600	H	1.76716000	-0.57921000	3.33887800
C	-2.27828500	4.80084500	3.99902200	H	3.22585300	-0.94526600	2.38335600
C	-0.96764700	4.95921200	3.54653800	H	1.94942600	-2.70122900	4.26274500

H	4.08182600	-3.40428000	2.13104800
H	5.21386500	-4.38133100	4.04441700
H	3.78777200	-3.91097900	5.15533600
H	-3.14430800	2.08407900	2.13458700
H	-4.07884200	3.61728800	3.83648300
H	-2.68537800	5.47078500	4.75773200
H	-0.34434200	5.76010600	3.94873800
H	0.57489600	4.24206000	2.21809900
H	-3.15949100	0.64251200	-1.84726500
H	-5.02863800	1.78217200	-2.99041300
H	-5.45586500	4.20490200	-2.57354000
H	-3.96887800	5.45971200	-1.01346900
H	-2.13377100	4.31105600	0.15207500
H	8.18296500	-4.79290400	-1.14560400
H	8.34645700	-3.46297300	-2.33140600
H	8.49516500	-3.12680400	-0.57275000
H	7.30759300	1.75574100	3.80147900
H	6.03716200	2.92523500	4.22312200
H	5.82635000	1.20621900	4.61606700
H	5.77033000	0.65609000	2.15045900
H	5.97807800	2.36051500	1.75875400
H	3.79927100	2.88675000	2.91131000
H	3.59769500	1.18059300	3.29605100
H	3.72015300	0.67868100	0.77476100
H	3.79326600	2.40223700	0.47854500

IM-R2

C	-2.29022900	5.39907700	-0.89963400
C	-3.39259500	5.43081800	-1.79669700
C	-4.13838200	4.29574600	-2.00519500
C	-3.83370900	3.07965000	-1.34190100
C	-2.73439200	3.05666000	-0.43250900
C	-1.97243900	4.22855800	-0.23040800
N	-4.59525200	1.98831100	-1.61346500
C	-4.32722700	0.85019600	-1.02166500
C	-3.25183100	0.72779500	-0.08647300
C	-2.46265200	1.80664100	0.21448500
C	-1.24081300	1.68305200	1.10947900
O	-1.51157300	6.46831300	-0.65116000
C	-5.13618500	-0.34191200	-1.39597900
C	-5.12342100	-1.51983900	-0.63593200
C	-5.81207600	-2.65396000	-1.05669500
C	-6.52686300	-2.59595200	-2.24587800
C	-6.59321100	-1.43435800	-3.00640400
C	-5.89628900	-0.31171300	-2.57363500
F	-7.17464000	-3.68480600	-2.66672300
N	-0.07310900	1.47747600	0.27480200
C	-1.38599400	0.53496000	2.11964900
C	1.04636200	2.19859400	0.32873400
C	2.28662600	2.01907700	-0.30527100
C	1.48375400	3.43499500	0.98732100
C	2.86996000	3.18710200	0.40951800
O	0.96327500	4.24283300	1.72903600
O	3.95043200	3.71448300	0.52127100
N	2.63528900	1.01790300	-1.12763200
C	3.90814100	0.58034400	-1.47533000
C	3.99117800	-0.62709200	-2.19345600
C	5.22856500	-1.16620000	-2.50571400
C	6.39746700	-0.50732500	-2.10881500
C	6.31709900	0.70079500	-1.41876300
C	5.07909300	1.25310800	-1.10199000
N	-0.10152100	0.34176500	2.88240500
C	0.29980200	1.54685200	3.67530700
C	-0.89415600	1.97985400	4.54192600
C	-1.93266800	0.85359600	4.54394300
C	-2.52877600	0.72616600	3.13791200
C	-0.25638300	-0.83671000	3.79428900
C	-1.22815900	-0.46555300	4.93033200
C	-2.21039200	-1.55157900	5.28169900
C	-2.24178400	-2.79606400	4.80717800
C	2.33575500	-0.94265800	1.28984000
C	0.17267200	-2.00603900	0.18239400
C	-0.18823700	-1.50983800	-1.08226700
C	-0.53647000	-3.07722400	0.92250300
C	0.17885500	-4.03057200	1.66957200
C	-0.47634700	-5.06740700	2.33421400
C	-1.86696500	-5.17613400	2.28200100
C	-2.59707000	-4.20966700	1.58784700
C	-1.94184000	-3.16890200	0.93417400
C	-1.23770400	-2.16536600	-1.93754400
O	0.34754300	-0.46662300	-1.59119600
C	-1.38133500	-3.55613100	-2.04061600
C	-2.34949700	-4.10437200	-2.87840300
C	-3.17987200	-3.27162100	-3.63330400
C	-3.01814200	-1.88832900	-3.56494400
C	-2.04778300	-1.34001700	-2.72711000
C	-1.77268900	7.67967700	-1.32433700
C	7.72326500	-1.12047500	-2.44515500

F	7.79468500	-2.39475500	-2.03418800
F	8.74148100	-0.46857300	-1.87886500
F	7.95193700	-1.14006200	-3.76389700
C	1.34968100	-1.44951200	0.77273200
C	7.35574500	-1.39863100	1.59034200
C	6.11834400	-0.59978000	1.98117500
C	4.83070600	-1.22774100	1.46024900
C	3.59159500	-0.40128900	1.82779500
H	-3.65168300	6.34698300	-2.32516200
H	-4.98426300	4.29837100	-2.69320400
H	-1.11372900	4.25798300	0.44260600
H	-3.04919500	-0.24756200	0.35225500
H	-1.10670900	2.62561900	1.66237500
H	-4.58665500	-1.56521800	0.31199000
H	-5.80303200	-3.57788700	-0.47838300
H	-7.17528000	-1.42934400	-3.92828600
H	-5.91236700	0.60760600	-3.15864300
H	-0.07783100	0.70771100	-0.42679500
H	-1.48722600	-0.40695200	1.56307500
H	1.86015300	0.35809300	-1.34261800
H	3.07001800	-1.13893000	-2.47878800
H	5.28798900	-2.11126100	-3.04856600
H	7.22937300	1.21415100	-1.11303900
H	5.02216400	2.19536400	-0.55522300
H	0.64249300	0.09556000	2.19755400
H	0.61929100	2.33662900	2.98957200
H	1.16845300	1.24076900	4.27132200
H	-1.34036000	2.90315800	4.14541600
H	-0.55080600	2.19382700	5.56194500
H	-2.72814600	1.07316700	5.26784200
H	-3.22264900	-0.12528700	3.09075600
H	-3.10122200	1.63108600	2.88815800
H	-0.62962400	-1.65597700	3.16639800
H	0.74525700	-1.10504400	4.15238700
H	-0.64343300	-0.25580300	5.84201100
H	-2.95321600	-1.25131900	6.02900800
H	-3.00104700	-3.50110200	5.15181600
H	-1.52671300	-3.17774300	4.07433000
H	1.26861300	-3.96577200	1.70027700
H	0.10812200	-5.80427000	2.88820400
H	-2.37762000	-5.99218100	2.79498800
H	-3.68741000	-4.26132800	1.56009400
H	-2.52494000	-2.43102100	0.37996700
H	-0.72908300	-4.21094700	-1.46064900
H	-2.45280200	-5.18836200	-2.94904500
H	-3.94425600	-3.70279100	-4.28261200
H	-3.65129200	-1.23268800	-4.16450600
H	-1.90086500	-0.25937500	-2.67488000
H	-1.01649500	8.39323500	-0.98185800
H	-1.68684500	7.55834800	-2.41528300
H	-2.77316100	8.06831800	-1.07847800
H	8.27727900	-0.92375200	1.95477600
H	7.31346500	-2.41761400	2.00368400
H	7.43161800	-1.48998300	0.49629600
H	6.19735400	0.42765900	1.58651600
H	6.06243400	-0.50457900	3.07871500
H	4.71750700	-2.24885300	1.86005100
H	4.88570600	-1.32287200	0.36306700
H	3.72830500	0.62871000	1.45248600
H	3.51738900	-0.31308900	2.92522900

TS-S2

C	6.01682900	-1.86858900	-1.96227600
C	6.59494100	-0.66351000	-2.44490300
C	5.81089400	0.45422400	-2.60144500
C	4.42872700	0.44546200	-2.27909300
C	3.85812000	-0.75711000	-1.76146400
C	4.67238100	-1.90568900	-1.63353500
N	3.71424500	1.58348400	-2.47829100
C	2.43039300	1.58202500	-2.21219900
C	1.76976200	0.42911000	-1.69115300
C	2.46592300	-0.71677100	-1.41558600
C	1.79818600	-1.91008600	-0.74840000
O	6.71216200	-3.01062200	-1.80098500
C	1.66674500	2.84887000	-2.39157700
C	2.36230900	4.05668200	-2.55435100
C	1.68296700	5.26242800	-2.69138700
C	0.29235000	5.25380900	-2.66923400
C	-0.43091900	4.07841300	-2.51878900
C	0.26339900	2.87879200	-2.37733100
F	-0.36526500	6.40956300	-2.79577500
N	0.35896100	-1.75348600	-0.70379800
C	2.40129800	-2.24706100	0.64024700
C	-0.48762100	-2.70192800	-1.10101100
C	-1.88439400	-2.80348300	-1.09393900
C	-0.32486400	-4.08388100	-1.59017000
C	-1.84496100	-4.17087700	-1.67824200
O	0.61344600	-4.80957700	-1.81477500

O	-2.63019000	-5.01239100	-2.03928700	H	6.23163100	1.38383600	-2.98587300
N	-2.78114500	-1.86805900	-0.70786800	H	4.26972900	-2.86156100	-1.29679200
C	-4.16558200	-1.96725400	-0.59182900	H	0.71078000	0.50192000	-1.46370100
C	-4.84821000	-0.88253600	-0.01435000	H	2.00359100	-2.80459600	-1.35924200
C	-6.22773300	-0.91483500	0.12275200	H	3.45193400	4.03269000	-2.56612200
C	-6.94423700	-2.03388500	-0.30750200	H	2.21115300	6.20869100	-2.81052000
C	-6.26855200	-3.11716300	-0.86934800	H	-1.52228900	4.10932300	-2.50784200
C	-4.88558900	-3.09266700	-1.01623400	H	-0.31911200	1.96371300	-2.26415900
N	2.01285600	-1.34710800	1.76736400	H	-0.03686400	-0.85900300	-0.39030500
C	0.70717300	-1.68916700	2.39760200	H	3.48305900	-2.09469400	0.54331000
C	0.79769100	-3.07351100	3.07191800	H	-2.37602900	-0.95874800	-0.46841400
C	2.09897700	-3.74219500	2.61476400	H	-4.28505900	-0.01525800	0.33525300
C	2.13739500	-3.69748100	1.08103000	H	-6.74754400	-0.06992600	0.57660300
C	3.07259100	-1.45228700	2.80321000	H	-6.82648000	-3.99551700	-1.19616700
C	3.28034000	-2.93891400	3.20848100	H	-4.36642900	-3.94303800	-1.45880300
C	4.62275900	-3.47375900	2.78699100	H	1.92949500	-0.14467100	1.50363800
C	5.47919300	-4.07405600	3.61225100	H	0.49217300	-0.88250900	3.11229300
C	1.83928800	1.30166000	1.29135500	H	-0.06721100	-1.65592300	1.62628200
C	0.61684000	1.60873200	1.37317400	H	-0.06595800	-3.68830500	2.78455000
C	-0.72112800	1.88589400	1.45902800	H	0.78586300	-2.97749700	4.16666000
C	-1.49711300	1.58334000	0.27092000	H	2.14672500	-4.77929600	2.97113400
C	-1.29174600	2.49178600	2.68325300	H	2.91456200	-4.35443800	0.66459500
C	-2.63269100	2.27088200	3.04281300	H	1.17622300	-4.05567800	0.68059200
C	-3.16578900	2.82756100	4.20310500	H	2.75329500	-0.83603700	3.65448000
C	-2.36645600	3.60127600	5.04595200	H	3.99019100	-1.00919800	2.39034100
C	-1.02736000	3.81180900	4.71252100	H	3.21658400	-3.01456800	4.30387400
C	-0.49772900	3.26792400	3.54453800	H	4.89920900	-3.35966800	1.73102600
C	-2.55706800	2.52055300	-0.22948100	H	6.43764500	-4.45709200	3.25745000
O	-1.24298400	0.58474800	-0.43437800	H	5.24608000	-4.20212300	4.67320300
C	-3.38113400	2.09149700	-1.27957200	H	-3.26560500	1.64866700	2.40620000
C	-4.29768800	2.96297800	-1.86370200	H	-4.21112700	2.64360500	4.45698400
C	-4.38714700	4.28129300	-1.41376200	H	-2.78247500	4.03256800	5.95746500
C	-3.55430900	4.72462200	-0.38371400	H	-0.39042400	4.41365200	5.36319800
C	-2.64305600	3.85188900	0.20500700	H	0.54672500	3.44835200	3.28145000
C	8.08286900	-3.03036900	-2.13417600	H	-3.28024400	1.06943600	-1.64748100
C	-8.43968700	-2.04383900	-0.20004000	H	-4.93622600	2.61547400	-2.67681400
F	-8.93111000	-3.28486900	-0.14954400	H	-5.09899300	4.96833000	-1.87385200
F	-9.02427200	-1.44170800	-1.24486600	H	-3.60660000	5.76029300	-0.04584700
F	-8.86372700	-1.39955500	0.89255400	H	-1.97573800	4.21373200	0.98723100
C	6.11700000	3.38952400	2.99370000	H	8.43591200	-4.04690300	-1.93331300
C	5.30906600	2.94862600	1.77902700	H	8.24032400	-2.79710200	-3.19856200
C	3.94777200	2.36987800	2.15178600	H	8.65543200	-2.32060300	-1.51715400
C	3.14725600	1.93332200	0.92252000	H	7.09239600	3.80313800	2.70219500
H	7.65163600	-0.61901200	-2.70345800	H	5.57945500	4.16265100	3.56300000

H	6.29901600	2.54299900	3.67295100
H	5.87440600	2.19450200	1.20563800
H	5.16201100	3.80215300	1.09660000
H	3.36574800	3.11678400	2.71716300
H	4.08363300	1.50969900	2.82932100
H	3.75212600	1.20994600	0.34863600
H	2.98513400	2.80311400	0.26444300

TS-R2

C	-2.74391900	5.26747900	-1.44340700
C	-3.83057700	5.09026100	-2.34278400
C	-4.44233900	3.86424500	-2.44394900
C	-4.01596400	2.75971100	-1.66283800
C	-2.93928800	2.94806800	-0.74508200
C	-2.30960000	4.21039100	-0.66089000
N	-4.63930100	1.56612300	-1.84059400
C	-4.25672400	0.52912000	-1.13637200
C	-3.20586700	0.62205200	-0.17142300
C	-2.54760100	1.80759200	0.03034400
C	-1.32545600	1.89662200	0.92990700
O	-2.08931400	6.43520000	-1.30104900
C	-4.90285000	-0.78269900	-1.41621300
C	-4.84399100	-1.85067000	-0.50948900
C	-5.38594500	-3.09298200	-0.82859100
C	-5.99948600	-3.25537100	-2.06389800
C	-6.11010800	-2.20867100	-2.97168200
C	-5.55821600	-0.97580500	-2.64061200
F	-6.50387500	-4.44890000	-2.38490200
N	-0.16075600	1.65337600	0.10083500
C	-1.39824600	0.92565200	2.11809200
C	0.94250700	2.39485700	0.10059000
C	2.20302700	2.18432400	-0.47371900
C	1.31974300	3.72488500	0.60215400
C	2.73331500	3.44669800	0.10384600
O	0.74738500	4.61718200	1.18814800
O	3.79407500	4.01883300	0.17401000
N	2.60202200	1.11072900	-1.18229200
C	3.89344500	0.67977500	-1.46437100
C	4.02991600	-0.56089200	-2.11173200
C	5.28960000	-1.09663800	-2.33146600
C	6.42619200	-0.39658300	-1.91751600
C	6.29324100	0.85252800	-1.31134100
C	5.03481600	1.39940200	-1.08439600
N	-0.13870700	0.91807600	2.90991600
C	0.13135100	2.21750700	3.56212200
C	-1.06178800	2.61035700	4.46421000
C	-2.07474500	1.46067600	4.46978900
C	-2.59664400	1.25083400	3.04578100
C	-0.23932500	-0.14059700	3.94178100
C	-1.36714600	0.17462400	4.94896500
C	-2.35534700	-0.93900000	5.18018500
C	-2.34808200	-2.16546200	4.65828400
C	1.91436300	-0.30780500	1.63266600
C	0.28933000	-1.96282500	0.40167400
C	-0.04445500	-1.67309600	-0.98182000
C	-0.36357800	-3.02186700	1.22133200
C	0.38201000	-3.76741500	2.14821900
C	-0.21970800	-4.76011700	2.92165100
C	-1.58165500	-5.03172800	2.78561000
C	-2.34136900	-4.27906700	1.88917000
C	-1.74227500	-3.27843000	1.12693600
C	-0.89225100	-2.61382700	-1.78222500
O	0.33663100	-0.63336100	-1.55279200
C	-0.75458400	-4.00533900	-1.70420300
C	-1.56536800	-4.82954300	-2.48056600
C	-2.52321400	-4.27114200	-3.33061100
C	-2.64995300	-2.88489000	-3.42588000
C	-1.82871700	-2.05790000	-2.66222300
C	-2.47796700	7.54071400	-2.08543200
C	7.78842700	-0.97872100	-2.14738800
F	7.77156500	-2.31589800	-2.08634800
F	8.67220200	-0.55094400	-1.24028400
F	8.28633000	-0.65937400	-3.34878700
C	1.22755600	-1.15892100	0.99261600
C	6.74586500	-1.85896100	1.47611000
C	5.76709600	-0.78269800	1.93205800
C	4.32055500	-1.12102300	1.58635400
C	3.33810700	-0.02234300	1.98864000
H	-4.18024300	5.91659800	-2.95968100
H	-5.27048200	3.70526100	-3.13520600
H	-1.46217500	4.39689200	0.00139500
H	-2.90937200	-0.27626600	0.36901100
H	-1.24385000	2.91889700	1.32664200
H	-4.39022600	-1.72007900	0.47407000
H	-5.34436800	-3.93331100	-0.13564700
H	-6.60955900	-2.37681500	-3.92618300
H	-5.61417300	-0.14016800	-3.33812700
H	-0.12633000	0.77419300	-0.42682800

H	-1.47162800	-0.10191400	1.73078200
H	1.85996600	0.42331700	-1.36623900
H	3.13416900	-1.10881600	-2.41127100
H	5.38979700	-2.07253000	-2.80838800
H	7.18148700	1.39891200	-0.99090500
H	4.93700600	2.37211200	-0.60044900
H	0.88682900	0.45450700	2.18838100
H	0.32343300	2.97550700	2.79507500
H	1.05965700	2.09388100	4.13585100
H	-1.54175000	3.52593600	4.08974300
H	-0.71729100	2.81712900	5.48647800
H	-2.90991300	1.69583700	5.14416300
H	-3.33273600	0.43534300	3.01709400
H	-3.10830700	2.16252300	2.70051100
H	-0.40191900	-1.08574000	3.40585800
H	0.74107400	-0.21086900	4.43188000
H	-0.91501700	0.40828200	5.92752000
H	-3.16379500	-0.67183400	5.87102100
H	-3.13298000	-2.88002300	4.91554600
H	-1.57814000	-2.52420400	3.97093000
H	1.45267600	-3.57323500	2.24376500
H	0.38447800	-5.33331600	3.62674800
H	-2.04984800	-5.81540300	3.38268300
H	-3.41213700	-4.46542500	1.78769800
H	-2.34841400	-2.70556200	0.42260900
H	-0.01250600	-4.44146800	-1.03319100
H	-1.45255500	-5.91284300	-2.42062300
H	-3.16961700	-4.91976700	-3.92447600
H	-3.39115900	-2.44639500	-4.09513400
H	-1.90958900	-0.97102600	-2.73058500
H	-1.80948200	8.36370700	-1.81318000
H	-2.36954800	7.32810400	-3.16031700
H	-3.51782800	7.83623500	-1.87583000
H	7.78633800	-1.58136900	1.69601100
H	6.53782500	-2.81774300	1.97491600
H	6.66517700	-2.02617400	0.39118200
H	6.02895700	0.17986100	1.46178200
H	5.85777000	-0.62932900	3.02067500
H	4.02602300	-2.06454700	2.07716900
H	4.23526100	-1.29424800	0.50053000
H	3.63046100	0.93053000	1.51117400
H	3.40285000	0.16985400	3.07285000

IM-S3

C	5.65029900	-2.89208900	-1.83513900
C	6.42169500	-1.80043100	-2.31817900
C	5.82580000	-0.57789500	-2.51490600
C	4.44919800	-0.36985100	-2.23972100
C	3.68283700	-1.46090300	-1.73317500
C	4.30666400	-2.71652900	-1.55217100
N	3.92372400	0.86062700	-2.47358100
C	2.64547700	1.06243900	-2.26039500
C	1.79913300	0.03088500	-1.75019200
C	2.30047000	-1.20607300	-1.44411000
C	1.45824700	-2.26860300	-0.76048800
O	6.15812700	-4.12307900	-1.62988100
C	2.11216500	2.43543400	-2.49915700
C	2.99885900	3.46472900	-2.85292300
C	2.55637600	4.77103500	-3.02357800
C	1.20622900	5.04958000	-2.83906800
C	0.29465300	4.05776600	-2.50536700
C	0.75539200	2.75248700	-2.34039200
F	0.77468700	6.30403000	-2.99205500
N	0.04998700	-1.92822600	-0.80527200
C	1.94660900	-2.51147600	0.68755200
C	-0.90650600	-2.79682800	-1.11032200
C	-2.30553200	-2.72895800	-1.09973700
C	-0.91878900	-4.22517100	-1.48859700
C	-2.43986900	-4.13081300	-1.56855500
O	-0.07969300	-5.07445400	-1.65926300
O	-3.32662500	-4.89805300	-1.85593800
N	-3.07904500	-1.65531100	-0.80695800
C	-4.46405100	-1.56079300	-0.71736000
C	-5.00691800	-0.32699500	-0.31698100
C	-6.37998000	-0.16708700	-0.20853900
C	-7.23072000	-1.23719300	-0.49495900
C	-6.69481300	-2.46439200	-0.88596600
C	-5.31928900	-2.63540700	-0.99977500
N	1.67791900	-1.40253400	1.61568200
C	0.35997500	-1.47907800	2.26970000
C	0.21972200	-2.76642400	3.12374900
C	1.42549400	-3.65906900	2.81634800
C	1.47774400	-3.84799300	1.29155100
C	2.68733900	-1.49867000	2.67449800
C	2.69372200	-2.92609600	3.32301300
C	3.95746400	-3.68818900	3.03675900
C	4.74404600	-4.22832500	3.96788000
C	2.36628300	1.71400700	0.93406000

C	1.08666200	1.75325300	1.20098200
C	-0.20306800	1.82320300	1.47048600
C	-1.15470800	1.68994600	0.31298700
C	-0.76427900	2.01147600	2.83260700
C	-2.10537100	1.69771200	3.09269300
C	-2.62968700	1.84134500	4.37715100
C	-1.82067800	2.29580600	5.41720400
C	-0.48190400	2.60758100	5.16607800
C	0.04240200	2.46988100	3.88466400
C	-1.93216500	2.88109400	-0.11702700
O	-1.25926900	0.62878800	-0.28745300
C	-2.82190400	2.75091100	-1.19430000
C	-3.51814700	3.86078200	-1.66073600
C	-3.32361600	5.10680900	-1.05721300
C	-2.43421500	5.24293300	0.01024200
C	-1.73757400	4.13320400	0.48222100
C	7.51914500	-4.35842200	-1.91416900
C	-8.71582500	-1.04503400	-0.42477500
F	-9.36076700	-2.19306700	-0.20125600
F	-9.21332700	-0.54098100	-1.56274600
F	-9.06178400	-0.19441800	0.54765800
C	6.04770500	4.75184800	2.60717000
C	5.11373100	4.40165100	1.45486100
C	4.17241500	3.24786800	1.78637600
C	3.23043400	2.90926100	0.62907200
H	7.48015400	-1.92409600	-2.54175200
H	6.39791200	0.26889500	-2.89535800
H	3.75248800	-3.58680900	-1.19874300
H	0.75135500	0.25283000	-1.57002500
H	1.57284700	-3.22306000	-1.29826300
H	4.05277800	3.21967400	-2.98107500
H	3.23891600	5.57771100	-3.29182200
H	-0.75905500	4.31288100	-2.37806700
H	0.02400200	1.98159900	-2.09765800
H	-0.22275700	-1.01303100	-0.44461500
H	3.04107200	-2.54821500	0.61093400
H	-2.56983600	-0.79201100	-0.62003900
H	-4.33995400	0.50587800	-0.08461400
H	-6.79126400	0.79212200	0.10893800
H	-7.35819800	-3.30292500	-1.10165300
H	-4.90956300	-3.59698200	-1.31012900
H	2.82882200	0.71817000	0.95892700
H	0.26036400	-0.57979800	2.89377900
H	-0.43591200	-1.41526700	1.51929200
H	-0.71260900	-3.29700500	2.88040700
H	0.19082600	-2.52765400	4.19749900
H	1.33506900	-4.62889100	3.32504700
H	2.15510600	-4.66164800	0.99106500
H	0.47368100	-4.12111200	0.92670400
H	2.46590100	-0.73046400	3.43025400
H	3.67730200	-1.26450100	2.25080300
H	2.60235800	-2.82691900	4.41587700
H	4.24217100	-3.79726700	1.98173000
H	5.65075000	-4.77535200	3.70229900
H	4.50287900	-4.13931000	5.03127600
H	-2.74981700	1.32583700	2.29189700
H	-3.67480200	1.59084000	4.56379400
H	-2.23032400	2.40756100	6.42189100
H	0.15664400	2.96599400	5.97480400
H	1.08685900	2.71877800	3.68544400
H	-2.94422400	1.77672400	-1.67043700
H	-4.20772900	3.75960100	-2.49925500
H	-3.86569900	5.97869800	-1.42617100
H	-2.27939100	6.21807200	0.47251600
H	-1.03273500	4.24064400	1.30810100
H	7.70638600	-5.41201400	-1.68318600
H	7.74472000	-4.17630300	-2.97641700
H	8.17401900	-3.73051700	-1.29014700
H	6.71656400	5.58385300	2.34672500
H	5.47636800	5.04552000	3.50060100
H	6.67338300	3.88917100	2.88137100
H	5.70353400	4.13750400	0.56136500
H	4.51518900	5.28473700	1.17563400
H	3.57562500	3.49985800	2.67945300
H	4.76217400	2.35339500	2.05074900
H	3.82995700	2.67859600	-0.26645800
H	2.59934500	3.77759800	0.38187500

IM-R3

C	-1.94905400	5.28041800	-1.98617600
C	-2.81680200	5.07452700	-3.09348800
C	-3.49010100	3.88396300	-3.22338400
C	-3.34397500	2.84523600	-2.26834100
C	-2.49173800	3.06522200	-1.14550000
C	-1.79301300	4.28871600	-1.03224600
N	-4.00380900	1.67734300	-2.48004500
C	-3.87784100	0.70082200	-1.61448100
C	-3.07374400	0.83518100	-0.44012800

C	-2.38137300	1.99408000	-0.19874900
C	-1.38317200	2.09696200	0.94080900
O	-1.24703200	6.41465700	-1.80205100
C	-4.54703500	-0.59291700	-1.92645400
C	-4.77398900	-1.57089400	-0.94796100
C	-5.33956300	-2.79997600	-1.28042300
C	-5.68568000	-3.04005000	-2.60355200
C	-5.50547900	-2.08128800	-3.59386300
C	-4.93472100	-0.86170600	-3.24740200
F	-6.21519000	-4.22063700	-2.93123400
N	-0.09812100	1.69632000	0.39158000
C	-1.75384600	1.26530200	2.17502900
C	1.03893300	2.35348600	0.55024100
C	2.35152400	2.08213200	0.13717400
C	1.42164300	3.67139100	1.08879500
C	2.87147100	3.31020900	0.78628200
O	0.82644400	4.62174200	1.54074900
O	3.94080700	3.83632200	0.98444700
N	2.78497000	1.00729700	-0.56261800
C	4.08631300	0.62395000	-0.86852600
C	4.26106000	-0.53753700	-1.64112000
C	5.53655800	-0.99423700	-1.93788400
C	6.65320400	-0.29633700	-1.47225000
C	6.48302600	0.87139900	-0.72814800
C	5.20890200	1.33781400	-0.42261200
N	-0.72128000	1.36453200	3.21218200
C	-0.72003600	2.67761200	3.86777700
C	-2.07731600	2.96443000	4.56279400
C	-3.01449300	1.79174800	4.26259700
C	-3.14635300	1.65163200	2.74124900
C	-0.98633200	0.32936300	4.21423100
C	-2.39542100	0.50289200	4.85165600
C	-3.34815200	-0.65515700	4.70735000
C	-3.12541100	-1.85774500	4.17607800
C	1.73371300	-0.51699100	2.07298200
C	0.02920100	-2.01258600	0.73258900
C	-0.08919100	-1.72891800	-0.73512500
C	-0.79341100	-3.04359600	1.42999400
C	-0.24152700	-3.79791000	2.47365600
C	-1.00992700	-4.74740600	3.14594500
C	-2.33956800	-4.96005400	2.78092600
C	-2.90034100	-4.20595000	1.74974600
C	-2.13644100	-3.25069900	1.08329900
C	-0.73359800	-2.71815100	-1.64392700

O	0.33094000	-0.67723900	-1.20568100
C	-0.57961000	-4.10063600	-1.47691900
C	-1.19592300	-4.97632900	-2.36628200
C	-1.98176100	-4.47748600	-3.40791400
C	-2.12971500	-3.10031900	-3.58124100
C	-1.49571600	-2.21958600	-2.70848200
C	-1.35974800	7.45356000	-2.74861500
C	8.02689800	-0.84604100	-1.70728700
F	8.39573500	-1.69735500	-0.73675300
F	8.95487800	0.11384400	-1.74229900
F	8.10636100	-1.52497300	-2.85577500
C	0.89843800	-1.26755000	1.40842300
C	5.71517900	-3.37750100	0.75713400
C	5.22585500	-2.12606600	1.47542700
C	3.70499300	-2.02587400	1.51061000
C	3.20727000	-0.78393800	2.23790800
H	-2.94889300	5.85021400	-3.84616700
H	-4.15158400	3.70266300	-4.07108200
H	-1.10715400	4.49109300	-0.20766300
H	-2.98302300	-0.00925100	0.24316500
H	-1.29492600	3.14650400	1.25319400
H	-4.53204200	-1.37720100	0.09821600
H	-5.52606400	-3.56894500	-0.53062100
H	-5.80030700	-2.30704800	-4.61907400
H	-4.76828700	-0.09493700	-4.00380400
H	-0.06024800	0.79886600	-0.09676700
H	-1.75441700	0.20197200	1.87786300
H	2.04885200	0.36684900	-0.86569600
H	3.38498900	-1.07837100	-2.00553100
H	5.66506000	-1.89766700	-2.53570200
H	7.35509900	1.42505500	-0.37770800
H	5.08417200	2.25432600	0.15532500
H	1.29305500	0.39357800	2.51395100
H	-0.47388400	3.45198800	3.13003400
H	0.10414800	2.67524800	4.59524500
H	-2.51986500	3.90048500	4.19126700
H	-1.95005800	3.07252900	5.65003900
H	-4.00285400	1.96463300	4.71345300
H	-3.89764300	0.89310800	2.47801200
H	-3.49119200	2.60790700	2.31364900
H	-0.87908600	-0.65062500	3.72515300
H	-0.20221700	0.39188800	4.98224300
H	-2.28043200	0.67877600	5.93529200
H	-4.34921700	-0.45020900	5.10792500

H	-3.92154800	-2.60475100	4.14638500
H	-2.16235300	-2.16331900	3.75926600
H	0.80291000	-3.64059200	2.75043200
H	-0.56347500	-5.32972100	3.95314600
H	-2.93856300	-5.70835400	3.30162100
H	-3.94279700	-4.35767300	1.46565300
H	-2.58502400	-2.67215900	0.27356200
H	0.02465100	-4.48982600	-0.65580100
H	-1.06896700	-6.05244000	-2.24367100
H	-2.47840600	-5.16778100	-4.09162000
H	-2.73859900	-2.71173700	-4.39805600
H	-1.59492800	-1.13909100	-2.83087600
H	-0.70502500	8.25988200	-2.40282500
H	-1.03078200	7.12468500	-3.74667800
H	-2.39281400	7.82964200	-2.81145300
H	6.81135700	-3.40211800	0.69208000
H	5.38057000	-4.28878900	1.27583900
H	5.31790700	-3.41407900	-0.26930300
H	5.62789700	-1.23020700	0.97193400
H	5.61912500	-2.10428700	2.50563300
H	3.27716700	-2.92710700	1.98342900
H	3.32285600	-2.01404500	0.47470800
H	3.74441700	0.10897900	1.87149000
H	3.43638200	-0.84244700	3.31469600

C9

C	-1.05476900	1.65121000	3.30905100
C	-1.98355800	1.02520500	4.18318300
C	-2.86566800	0.09834900	3.68358700
C	-2.88538200	-0.24385000	2.30613200
C	-1.96658100	0.40313600	1.42405800
C	-1.04742400	1.33475300	1.96248400
N	-3.77396600	-1.18619100	1.89714100
C	-3.80529000	-1.52611900	0.63128800
C	-2.97320200	-0.89159300	-0.33911200
C	-2.06504200	0.06393800	0.03161300
C	-1.25670300	0.76313900	-1.04628800
O	-0.14935000	2.56305900	3.72077200
C	-4.74806500	-2.59965700	0.21899500
C	-5.89949000	-2.84637100	0.97963800
C	-6.79215600	-3.84908700	0.61729500
C	-6.51563900	-4.61305200	-0.51131400
C	-5.37646900	-4.40677400	-1.27841900
C	-4.49432700	-3.39445100	-0.90735100
F	-7.36879100	-5.57846300	-0.86408400
N	0.17940100	0.66762300	-0.82624200
C	-1.63447500	2.25402800	-1.12630500
C	0.82119600	-0.50039000	-0.83621000
C	2.16006300	-0.87086500	-0.66789200
C	0.41009900	-1.89300000	-1.13447400
C	1.84854100	-2.31391600	-0.84353700
O	-0.60369800	-2.44689900	-1.47356300
O	2.43791300	-3.36561700	-0.83107600
N	3.21939200	-0.07886300	-0.37289500
C	4.57027100	-0.40753400	-0.26739500
C	5.04872600	-1.70823400	-0.47746500
C	6.41097000	-1.96446400	-0.36382000
C	7.30346100	-0.94142200	-0.04534000
C	6.82813700	0.35515600	0.16241400
C	5.47153800	0.62172400	0.05318300
N	-0.63440400	3.01924600	-1.87962800
C	-0.82331000	2.83217500	-3.32311200
C	-2.16166700	3.46231900	-3.78831300
C	-2.99467500	3.76362500	-2.53605600
C	-3.05422700	2.48594700	-1.68616000
H	-1.60066100	2.65619600	-0.10536700
C	-0.80361400	4.43831000	-1.55134600
C	-2.27982400	4.88483300	-1.73773800
C	-3.04633700	5.21018200	-0.47955300
C	-2.65656200	5.09625700	0.79119400
H	0.67405500	1.54810900	-0.98228700
H	3.00181900	0.89884900	-0.20720500
C	-0.10896100	2.91613900	5.08538000
C	8.76509600	-1.23130600	0.12199800
F	9.52391300	-0.20855200	-0.28635700
F	9.14447700	-2.31158300	-0.56558800
F	9.09068000	-1.45620500	1.40223100
H	-1.99817400	1.26435200	5.24537900
H	-3.57823500	-0.40657300	4.33647300
H	-0.29983900	1.81831400	1.33732300
H	-3.05958600	-1.16731300	-1.38960800
H	-1.50867500	0.27906900	-2.00551500
H	-6.08406300	-2.23533300	1.86329200
H	-7.69713700	-4.04926200	1.19132000
H	-5.18892900	-5.04170600	-2.14434600
H	-3.58001700	-3.24860000	-1.48534700
H	4.35954900	-2.51719200	-0.71845500
H	6.78101100	-2.97654200	-0.53213100

H	7.52183800	1.16173500	0.40371900
H	5.10113700	1.63592700	0.21226200
H	0.03382200	3.28025800	-3.84499300
H	-0.79607200	1.75334600	-3.53667800
H	-1.98513200	4.39153800	-4.35078100
H	-2.70695800	2.77659200	-4.45274400
H	-4.00710800	4.09102500	-2.81244100
H	-3.77818900	2.57999300	-0.86459800
H	-3.37776500	1.63988000	-2.31515200
H	-0.14185000	5.01572300	-2.21204900
H	-0.45540800	4.60885800	-0.52330700
H	-2.30562200	5.78957100	-2.36767000
H	-4.06561200	5.56927100	-0.66936200
H	-3.33665700	5.35955500	1.60366200
H	-1.66382700	4.74511900	1.08435400
H	0.69022600	3.65693000	5.19123400
H	-1.06056100	3.36315400	5.41263700
H	0.12014700	2.04529300	5.71910900

3f'

C	1.43309700	1.29007000	-1.40894000
C	0.25929900	0.99277900	-1.42251000
C	-1.14901900	0.58759700	-1.44005400
C	-1.29148700	-0.94907700	-1.45277500
C	-2.00859800	1.18841000	-0.32698000
C	-1.51081400	2.12918400	0.57675500
C	-2.33827800	2.66129400	1.56698100
C	-3.66907100	2.25759000	1.66306800
C	-4.17231200	1.31995400	0.75984200
C	-3.34732400	0.79019600	-0.23013000
C	2.86205800	1.60185000	-1.32242200
C	3.53086900	0.92981700	-0.11698500
C	5.01982900	1.24435000	-0.02419000
C	5.68563900	0.57670300	1.17330600
C	-0.67374200	-1.76924700	-0.36099800
O	-1.92623000	-1.46896400	-2.34036100
C	-0.04115900	-1.20893300	0.75687700
C	0.50441600	-2.03656600	1.73662800
C	0.42507100	-3.42350000	1.60674500
C	-0.20685100	-3.98747700	0.49623800
C	-0.75576300	-3.16320500	-0.48067200
H	-1.58741300	0.89418400	-2.40242100
H	-0.46850200	2.44543600	0.50397100
H	-1.93734800	3.39623500	2.26669400

H	-4.31393000	2.67276200	2.43883100
H	-5.21323300	0.99974800	0.82464000
H	-3.74629100	0.06103700	-0.93954700
H	3.35906900	1.28102700	-2.25172100
H	2.99260100	2.69409400	-1.26159100
H	3.02190500	1.25656700	0.80512000
H	3.38174100	-0.16029700	-0.18461200
H	5.51513400	0.92229700	-0.95513000
H	5.15540400	2.33712900	0.03315900
H	6.75728500	0.81391900	1.22318500
H	5.22261200	0.90752500	2.11510800
H	5.58412200	-0.51757200	1.11901400
H	0.02821900	-0.12760600	0.86985300
H	0.99402200	-1.59493500	2.60546100
H	0.85596500	-4.06778500	2.37477400
H	-0.27082000	-5.07157400	0.39435600
H	-1.25586600	-3.58121900	-1.35495700

3f

C	2.12294600	-1.52429800	1.91920000
C	-0.27989900	-0.79538600	1.15483600
C	-0.74437300	0.56492700	1.63312800
C	-1.13156300	-1.57201400	0.21673400
C	-0.58881800	-2.60358700	-0.56372000
C	-1.39498400	-3.33001700	-1.43499800
C	-2.75628600	-3.03598200	-1.54451600
C	-3.30470300	-2.01253600	-0.77275100
C	-2.49878500	-1.28543200	0.10267100
C	-0.96218000	1.61902600	0.58872000
O	-0.89924400	0.80257500	2.80843300
C	-0.50157300	1.46712600	-0.72528500
C	-0.68284200	2.49481600	-1.64927900
C	-1.33213800	3.66974900	-1.26784200
C	-1.79392800	3.82431900	0.04175000
C	-1.60379500	2.80425800	0.96887900
C	0.90363900	-1.19686300	1.57545700
C	3.92572100	0.98650600	-2.10635900
C	4.26992100	0.13121900	-0.89221300
C	3.03177800	-0.27798000	-0.10233100
C	3.34376500	-1.13044900	1.12068800
H	2.27477300	-2.11086000	2.83277900
H	0.47648200	-2.83019900	-0.48159000
H	-0.95795100	-4.12842100	-2.03663600
H	-3.38642800	-3.60389000	-2.23035000

H	-4.36743000	-1.77795000	-0.84820600
H	-2.94243900	-0.49070000	0.70708100
H	0.00859200	0.54912900	-1.02352800
H	-0.31696300	2.37757400	-2.67009100
H	-1.47788800	4.47068800	-1.99439700
H	-2.29971500	4.74427500	0.33795200
H	-1.94477300	2.90579100	2.00012200
H	4.82527800	1.28124700	-2.66426500
H	3.26474000	0.43974400	-2.79603900
H	3.40037500	1.90455200	-1.80184700
H	4.95663600	0.68302800	-0.22914100
H	4.81300600	-0.77323800	-1.21303000
H	2.34045500	-0.83025800	-0.76352300
H	2.48547300	0.62612500	0.21838700
H	4.03461600	-0.59223700	1.79125200
H	3.87568700	-2.04941100	0.82099300

2a-1’

Bi	0.00330400	-0.07055200	0.00067700
C	-0.05478900	2.08362100	0.00651800
C	-1.25194100	2.72975600	-0.30115000
C	1.10478200	2.79401900	0.31621600
C	-1.27983400	4.12401000	-0.30468900
H	-2.16114500	2.16515700	-0.51464400
C	1.05564900	4.18768500	0.32337000
H	2.04373500	2.28020900	0.52889300
C	-0.13121000	4.84883900	0.01015800
H	-2.20883200	4.64207200	-0.54563900
H	1.95457500	4.75565900	0.56588700
H	-0.16125100	5.93936300	0.01150100
C	1.77146900	-1.16294700	0.57643700
C	2.43554300	-1.90295300	-0.40397200
C	2.22078700	-1.13429900	1.89755100
C	3.57266600	-2.62638300	-0.05000600
H	2.07103300	-1.92819500	-1.43300800
C	3.36818000	-1.85590500	2.23255700
H	1.67668900	-0.57698700	2.66034300
C	4.04093500	-2.59773000	1.26378400
H	4.09588500	-3.21081500	-0.80789400
H	3.73104900	-1.83821700	3.26113400
H	4.93530900	-3.16105500	1.53392500
C	-1.70922300	-1.24340800	-0.58474300
C	-2.16148900	-1.22281700	-1.90503700
C	-2.33499800	-2.02527200	0.38830400
C	-3.27294900	-1.99577700	-2.24654400

H	-1.64631300	-0.63171700	-2.66246400
C	-3.43610300	-2.79943400	0.02788100
H	-1.96820000	-2.04351200	1.41668300
C	-3.90729900	-2.77966000	-1.28502700
H	-3.63787100	-1.98489100	-3.27449200
H	-3.92902300	-3.41673400	0.77998700
H	-4.77370700	-3.38287000	-1.56014500
O	-0.52783500	-0.21675800	2.13528900
O	0.54102500	-0.17204600	-2.13526000
N	-1.65235000	0.35917200	2.51757100
N	1.63545200	0.46320400	-2.51039100
O	1.87837400	0.48726800	-3.67952200
O	2.30964800	0.95870000	-1.62640600
O	-1.89421600	0.35969700	3.68718100
O	-2.35185900	0.82859300	1.63919500

2a-2’

Bi	0.00060500	0.00577300	-0.06625600
C	-0.00391000	-0.18463400	2.08206700
C	-0.55138000	-1.33138200	2.65914900
C	0.54019400	0.84052900	2.85707500
C	-0.54437000	-1.45052000	4.04892200
H	-1.00671400	-2.11046100	2.04596400
C	0.52707800	0.70955600	4.24569900
H	0.99726900	1.71671200	2.39503600
C	-0.00999000	-0.43273500	4.83790100
H	-0.96829500	-2.34180800	4.51315500
H	0.94838400	1.50357700	4.86348300
H	-0.01218800	-0.53064800	5.92452900
C	0.48477600	1.89340100	-0.99480900
C	-0.39086700	2.97212900	-0.85373000
C	1.66607800	2.00693400	-1.73122300
C	-0.06191700	4.18900200	-1.45296200
H	-1.32675700	2.86150100	-0.30531900
C	1.97333200	3.22614700	-2.33288600
H	2.34442100	1.15979200	-1.84598600
C	1.11470000	4.31558700	-2.18911800
H	-0.73692300	5.03913700	-1.34531600
H	2.89186200	3.32197600	-2.91322800
H	1.36358500	5.26895000	-2.65750400
C	-0.48104700	-1.69736700	-1.30156200
C	-1.65924500	-1.68513100	-2.05132300
C	0.39216700	-2.78671600	-1.33667900
C	-1.96564900	-2.78774300	-2.84713500

H	-2.33559800	-0.82889300	-2.02668100
C	0.06399300	-3.88751000	-2.12940500
H	1.32586200	-2.77012600	-0.77384700
C	-1.10948900	-3.88820400	-2.88111900
H	-2.88167000	-2.78462100	-3.43922800
H	0.73696300	-4.74547400	-2.16038700
H	-1.35806000	-4.75085900	-3.50100900
O	2.07432500	-0.66318700	-0.24634800
O	-2.07211600	0.69607200	-0.13260900
C	-3.02027500	-0.03721600	0.34915900
C	3.01727800	-0.02205700	0.36037800
O	2.91496500	0.95275300	1.06181900
O	-2.92582400	-1.12134600	0.86706700
C	-4.39780600	0.61488700	0.11258000
C	4.40052200	-0.61000400	0.01419300
F	5.34823200	-0.11581100	0.79030200
F	4.69451100	-0.30590800	-1.25374800
F	4.40585100	-1.93457100	0.12814400
F	-4.39700100	1.89112700	0.48613200
F	-4.68131400	0.56934700	-1.19260100
F	-5.35562700	-0.01573500	0.76789300

2a-3’

Bi	0.00061500	-0.13863500	-0.11300400
C	-0.71741700	0.17844400	-2.12352700
C	-1.96312600	-0.34156800	-2.48582700
C	0.07190700	0.89910600	-3.02240800
C	-2.42425600	-0.12141900	-3.78498800
H	-2.58030200	-0.90727000	-1.78292700
C	-0.41130000	1.11164400	-4.31409800
H	1.05173700	1.27531400	-2.72497800
C	-1.65355900	0.60415100	-4.69294000
H	-3.39338000	-0.52484500	-4.08145700
H	0.19402200	1.67456500	-5.02583400
H	-2.02248800	0.77207600	-5.70607700
C	-0.00711500	1.52023700	1.27297500
C	0.79329000	2.63436100	1.00567800
C	-0.81411500	1.45279000	2.41190500
C	0.77656800	3.70364800	1.90314800
H	1.44595500	2.67680500	0.12923100
C	-0.81077600	2.52909200	3.30117500
H	-1.46202700	0.59406900	2.60800200
C	-0.02043500	3.64988900	3.04659300
H	1.39585200	4.57904700	1.70234100
H	-1.43543700	2.48535600	4.19441000
H	-0.02576300	4.48840100	3.74464400
C	0.72833000	-2.06092300	0.54500700
C	1.97815500	-2.49804900	0.09721000
C	-0.05926400	-2.83128500	1.40321400
C	2.44560100	-3.73864700	0.53415900
H	2.59335100	-1.89662300	-0.57709300
C	0.43048900	-4.06571600	1.83148600
H	-1.04239300	-2.48032500	1.72031800
C	1.67708300	-4.51710200	1.39909500
H	3.41798700	-4.09275500	0.18920200
H	-0.17285500	-4.67716700	2.50377000
H	2.05094700	-5.48532900	1.73577600
O	1.94656700	0.41635000	-0.78546900
O	-1.94537700	-0.71287700	0.54416800
S	-3.43642800	-0.37065700	0.83071400
O	-4.27546600	-1.18877600	-0.02704200
O	-3.64309700	-0.40112100	2.26961800
S	3.43403800	0.76587700	-0.49147200
O	4.28031600	-0.22452800	-1.13233900
O	3.63764800	2.17555500	-0.78359300
C	3.58939700	0.53758500	1.26930000
H	2.92617200	1.23963900	1.78801900
H	3.34672800	-0.50391500	1.51393700
H	4.63721000	0.75048000	1.51113700
C	-3.60549800	1.31759100	0.28347700
H	-2.94554400	1.96054700	0.87771200
H	-3.36513800	1.36945800	-0.78581200
H	-4.65592000	1.58586900	0.45055500

2a-4’

Bi	-0.00007400	0.00012100	-0.00002500
C	0.37935400	-2.11471600	-0.00000700
C	0.49898500	-2.78315600	-1.21943800
C	0.49947200	-2.78302700	1.21945500
C	0.74546900	-4.15632700	-1.21070700
H	0.39871000	-2.22555500	-2.15201500
C	0.74592600	-4.15620600	1.21075900
H	0.39961300	-2.22531300	2.15200600
C	0.86817300	-4.83867200	0.00004000
H	0.84185600	-4.69463300	-2.15488800
H	0.84264900	-4.69442300	2.15495800
H	1.06096200	-5.91276200	0.00005600
C	1.64192500	1.38585100	-0.00006900

C	2.16086700	1.82377000	1.21935800
C	2.16084600	1.82388000	-1.21946300
C	3.22710500	2.72350100	1.21067900
H	1.72804400	1.45822400	2.15194900
C	3.22710100	2.72357500	-1.21070800
H	1.72800700	1.45846700	-2.15209600
C	3.75697100	3.17069200	0.00000000
H	3.64495600	3.07617800	2.15488900
H	3.64499500	3.07627800	-2.15489200
H	4.59100600	3.87441400	0.00002300
C	-2.02131100	0.72865400	0.00003900
C	-2.66005600	0.95904800	1.21946100
C	-2.66013900	0.95898300	-1.21935300
C	-3.97243800	1.43237700	1.21077500
H	-2.12711500	0.76696600	2.15207000
C	-3.97251700	1.43233900	-1.21060900
H	-2.12724900	0.76684000	-2.15198000
C	-4.62468900	1.66755900	0.00009900
H	-4.48674300	1.61804300	2.15498600
H	-4.48686300	1.61801300	-2.15479400
H	-5.65117800	2.03789700	0.00012100
F	-0.00010800	0.00014600	-2.04481000
F	-0.00008800	0.00014400	2.04478900

C	2.44884200	-4.26105700	0.60799600
H	1.64487400	-4.27354600	2.61151100
H	3.11147500	-3.98144600	-1.42942300
H	2.98983200	-5.19985800	0.73633200
C	1.31481100	1.71295700	-0.27813700
C	2.12865300	2.11581400	0.78175700
C	1.34308000	2.37950100	-1.50375500
C	2.98175500	3.20417800	0.60993100
H	2.10139400	1.58471400	1.73502600
C	2.19167500	3.47749800	-1.65767100
H	0.72567800	2.03350300	-2.33256600
C	3.00813100	3.88794100	-0.60570200
H	3.62426900	3.52144800	1.43247300
H	2.21549600	4.00930200	-2.61002900
H	3.67191700	4.74440200	-0.73357800
O	0.08749700	-0.17933400	-2.17662400
O	0.10761500	0.16848700	2.17732500
C	-0.38243700	1.25323400	2.70325200
C	-0.54672200	-1.18399600	-2.70798200
O	-1.07692800	-2.08150900	-2.09330200
O	-0.78065400	2.21322300	2.08310800
H	-0.39575100	1.22621200	3.81616700
H	-0.55186500	-1.15136500	-3.82083100

2a-5’

Bi	0.03014200	-0.00153200	0.00017800
C	-2.12776400	0.14420400	0.00021700
C	-2.72595700	1.40468100	0.03495500
C	-2.88823800	-1.02549500	-0.03369900
C	-4.11859500	1.48824800	0.02670200
H	-2.12350300	2.31129500	0.10182700
C	-4.27961100	-0.92338500	-0.02549100
H	-2.41167700	-2.00417900	-0.09956800
C	-4.89210400	0.32871600	0.00040300
H	-4.59705300	2.46828700	0.05128100
H	-4.88411400	-1.83111300	-0.04985700
H	-5.98084000	0.40136000	0.00031300
C	1.06770400	-1.87566800	0.27930000
C	1.00362800	-2.54005300	1.50476400
C	1.82015000	-2.38560600	-0.77993200
C	1.69469500	-3.74339400	1.65924200
H	0.43845200	-2.11333300	2.33324300
C	2.51706200	-3.57994700	-0.60749600
H	1.86684600	-1.85564600	-1.73307100

2a-6’

Bi	0.00973900	-0.04665400	0.00025300
C	-0.41841200	2.07514000	0.00133600
C	-1.70295900	2.51104000	0.32918500
C	0.59564800	2.97616800	-0.32634700
C	-1.96856400	3.88073200	0.33593300
H	-2.49944800	1.79812300	0.54466700
C	0.30817200	4.34146700	-0.33201800
H	1.60643200	2.62896500	-0.54303800
C	-0.96793900	4.79226300	0.00229100
H	-2.96876300	4.23195600	0.59357300
H	1.09320000	5.05388500	-0.58951100
H	-1.18423900	5.86180700	0.00274900
C	1.86048100	-0.88496600	-0.73769700
C	2.17295300	-0.82524100	-2.09616600
C	2.71410700	-1.51359800	0.17032600
C	3.36871300	-1.39151000	-2.54291000
H	1.48107800	-0.35970600	-2.79777200
C	3.89718100	-2.08805000	-0.29017000
H	2.45679400	-1.55932400	1.23039600

C	4.22729300	-2.02101300	-1.64402400
H	3.62429500	-1.34297800	-3.60255800
H	4.56585300	-2.58575300	0.41366600
H	5.15824200	-2.46537200	-1.99961500
C	-1.37366100	-1.53556900	0.73710400
C	-1.91887700	-2.44417200	-0.17155500
C	-1.68385800	-1.60333800	2.09577700
C	-2.78740500	-3.43201500	0.28840800
H	-1.66513000	-2.38584300	-1.23184300
C	-2.56722300	-2.58854400	2.54204100
H	-1.22534200	-0.90760600	2.79813900
C	-3.11647100	-3.49984400	1.64247500
H	-3.21227600	-4.14853400	-0.41608400
H	-2.82065800	-2.64399100	3.60186800
H	-3.80285100	-4.27008900	1.99768300
O	0.74334500	0.03327500	2.05007200
O	-0.69724200	-0.25438000	-2.04972300
C	-1.94736700	0.07269200	-2.26450300
C	1.77008900	0.81832200	2.26312500
O	2.36222300	1.39266300	1.37003400
O	-2.71716000	0.37249200	-1.37266900
C	-2.34998200	0.03394300	-3.72030800
H	-3.41185800	0.27853800	-3.82154100
H	-1.73833500	0.75381600	-4.28077200
H	-2.14272600	-0.96264700	-4.13251400
C	2.15968700	0.93754900	3.71807000
H	3.04401200	1.57448000	3.81772000
H	1.31820000	1.36329100	4.28112300
H	2.35619600	-0.06189100	4.12870100

2a-7’

Bi	-0.00037500	0.00435000	0.05797200
C	-0.00481400	-0.10856500	-2.10059400
C	-0.40312200	1.00539100	-2.84035400
C	0.38989000	-1.29490300	-2.71996100
C	-0.39382500	0.92590300	-4.23308600
H	-0.74802000	1.91379200	-2.34541100
C	0.37472700	-1.36316100	-4.11321200
H	0.73661900	-2.14622400	-2.13335000
C	-0.01093300	-0.25539900	-4.86668800
H	-0.69924900	1.79186600	-4.82199000
H	0.67766400	-2.28669700	-4.60847000
H	-0.01322800	-0.31327600	-5.95630200
C	0.29895000	-1.75112300	1.28157800
C	-0.62347700	-2.79749100	1.23681000
C	1.41657700	-1.81153000	2.11526700
C	-0.41197600	-3.92387600	2.03397700
H	-1.50393500	-2.72904000	0.59721500
C	1.60768900	-2.93460600	2.91849600
H	2.13657300	-0.99094300	2.14192500
C	0.69808500	-3.99108000	2.87393000
H	-1.12371700	-4.75009400	1.99851000
H	2.47623700	-2.98483200	3.57664700
H	0.85608900	-4.87176200	3.49836600
C	-0.29409400	1.87324600	1.10208100
C	-1.40681600	2.01689600	1.93203900
C	0.62756600	2.90967600	0.94702900
C	-1.59369400	3.21497600	2.61966000
H	-2.12621500	1.20300500	2.04417000
C	0.42028900	4.11032300	1.62843800
H	1.50388600	2.77756700	0.31177200
C	-0.68479500	4.26149800	2.46399800
H	-2.45854200	3.33136500	3.27430000
H	1.13133800	4.92878300	1.50599200
H	-0.83953700	5.20032700	2.99798000
O	2.11588600	0.51074800	0.16960400
O	-2.11632700	-0.48786300	0.22774600
C	-2.95724200	0.33077900	-0.34543100
C	2.95654700	-0.36060400	-0.32037100
O	2.61211100	-1.41612000	-0.82087700
O	-2.61332200	1.33249100	-0.94699300
C	-4.40563000	-0.03553300	-0.18651700
C	-4.79550100	-1.14607200	0.56679800
C	-5.36971000	0.76546100	-0.80451000
C	-6.14791700	-1.45470200	0.69887700
H	-4.02742600	-1.75405900	1.04581800
C	-6.72018400	0.45378100	-0.67367900
H	-5.03241600	1.62682700	-1.38244500
C	-7.10926000	-0.65645700	0.07800900
H	-6.45483000	-2.32053800	1.28753400
H	-7.47386400	1.07684100	-1.15752700
H	-8.16805500	-0.90027500	0.18102500
C	4.40528100	0.01872100	-0.19777600
C	4.79621400	1.19680000	0.44420500
C	5.36880000	-0.84006800	-0.73356200
C	6.14904600	1.51455100	0.54746000
H	4.02881100	1.84981100	0.86091900
C	6.71968100	-0.51916800	-0.63227300

H	5.03086100	-1.75331600	-1.22496800
C	7.10979100	0.65821300	0.00842500
H	6.45672300	2.43292700	1.04975500
H	7.47284800	-1.18760600	-1.05213200
H	8.16889000	0.90917100	0.08874500

2a-8’

Bi	0.00009300	0.51144100	-0.00131500
C	0.00039600	2.67589100	-0.00426800
C	0.59187200	3.35600800	-1.06944700
C	-0.59086500	3.35825900	1.05960100
C	0.57915400	4.75114900	-1.06734400
H	1.08700200	2.81424700	-1.87571000
C	-0.57764900	4.75338300	1.05485400
H	-1.08622700	2.81821800	1.86689000
C	0.00088800	5.44715700	-0.00690400
H	1.03564700	5.29278200	-1.89699900
H	-1.03396900	5.29675500	1.88346600
H	0.00108900	6.53835100	-0.00793900
C	-0.61932900	-0.68492600	1.68765100
C	0.09113000	-0.64657500	2.88760600
C	-1.72848800	-1.51790400	1.53466800
C	-0.33497500	-1.44107100	3.95391400
H	0.97697000	-0.01821200	2.98162800
C	-2.13831400	-2.31486000	2.60252300
H	-2.27675200	-1.54355000	0.58960800
C	-1.44590200	-2.27085900	3.81284700
H	0.20991500	-1.41127700	4.89874700
H	-3.00664400	-2.96631000	2.48792700
H	-1.77297000	-2.89024400	4.64943000
C	0.61869400	-0.69333200	-1.68462900
C	1.72747300	-1.52606800	-1.52760800
C	-0.09196600	-0.66072100	-2.88462200
C	2.13672700	-2.32860700	-2.59149400
H	2.27587500	-1.54726200	-0.58252100
C	0.33358100	-1.46080800	-3.94696600
H	-0.97754400	-0.03245700	-2.98165100
C	1.44413900	-2.29039800	-3.80190700
H	3.00474400	-2.97990700	-2.47374400
H	-0.21147500	-1.43554600	-4.89183400
H	1.77076100	-2.91416500	-4.63540100
O	-1.98227800	0.42211000	-0.90222500
O	1.98268600	0.42497700	0.89975100
C	2.95222800	0.96796400	0.20734500
C	-2.95234400	0.96780900	-0.21259900
O	-2.78895700	1.45063800	0.89351100
O	2.78802200	1.44622800	-0.90066600
C	4.31069400	0.87486800	0.86857600
H	4.97242900	1.62540300	0.41319500
H	4.19848400	1.08865300	1.94109900
C	-4.31040400	0.87152200	-0.87423300
H	-4.97251900	1.62404300	-0.42269000
H	-4.19763400	1.08036100	-1.94767000
C	4.89757200	-0.53048100	0.68118200
C	5.21853700	-0.82155800	-0.78876600
C	6.14659300	-0.74161200	1.54094900
H	4.13378800	-1.25552200	1.02236000
C	5.76864300	-2.23542300	-0.97004700
H	5.97403200	-0.08961400	-1.12794900
H	4.33304100	-0.65581300	-1.42080900
C	6.72001600	-2.14832400	1.36423300
H	6.90581900	0.00637500	1.24852400
H	5.90916900	-0.55446800	2.60013400
C	7.01050200	-2.45555600	-0.10534900
H	5.99829700	-2.42394200	-2.02998600
H	4.99240000	-2.96608300	-0.67789300
H	7.63166200	-2.26731300	1.96910000
H	5.98945700	-2.88190500	1.74750000
H	7.38185600	-3.48573800	-0.21508800
H	7.81536500	-1.78905200	-0.46111700
C	-4.89713200	-0.53305300	-0.68067100
C	-5.21916700	-0.81734800	0.79037500
C	-6.14547300	-0.74854500	-1.54035400
H	-4.13295200	-1.25953300	-1.01787900
C	-5.76896600	-2.23052700	0.97783600
H	-5.97516500	-0.08406800	1.12553300
H	-4.33423900	-0.64836200	1.42235400
C	-6.71861000	-2.15459600	-1.35752100
H	-6.90511500	0.00057300	-1.25193200
H	-5.90735200	-0.56625700	-2.60022900
C	-7.01011100	-2.45509300	0.11325100
H	-5.99934400	-2.41417800	2.03847100
H	-4.99226300	-2.96228300	0.68967000
H	-7.62976200	-2.27666500	-1.96252100
H	-5.98754700	-2.88973400	-1.73682800
H	-7.38121200	-3.48487500	0.22749800
H	-7.81545200	-1.78720000	0.46531100

2a-1’’

Bi	0.09564400	0.13453500	-0.72465200
C	1.08900900	-0.32685500	1.15507900
C	2.34228300	0.20228100	1.47448600
C	0.43273300	-1.17655100	2.05038800
C	2.92944500	-0.11127100	2.70056500
H	2.87466000	0.84474700	0.77009500
C	1.02578000	-1.48360400	3.27516200
H	-0.53938500	-1.60958200	1.79976600
C	2.27167000	-0.94927100	3.60005000
H	3.90861000	0.30008300	2.95001400
H	0.51216800	-2.14527300	3.97414300
H	2.73543500	-1.19182100	4.55739100
C	-2.14496700	0.22013700	0.03724100
C	-2.91732600	-0.92679000	-0.07734000
C	-2.56891400	1.39448600	0.64232700
C	-4.19251000	-0.89764900	0.49851700
H	-2.54694900	-1.82595400	-0.57333300
C	-3.84838700	1.39275900	1.20865100
H	-1.93657200	2.28294800	0.69155100
C	-4.65175000	0.25492700	1.13519900
H	-4.82472100	-1.78529000	0.44142000
H	-4.21273100	2.29356600	1.70524700
H	-5.65008300	0.26867800	1.57464400
O	0.18997500	2.43793300	-0.05021200
O	1.47713700	-1.47525100	-1.77326200
N	1.30934700	2.66829000	-0.58786600
N	0.75654600	-2.47944300	-1.48124600
O	1.05034800	-3.58824600	-1.78050700
O	-0.30830800	-2.19672500	-0.84442200
O	1.85323600	3.72101300	-0.53884300
O	1.82229100	1.66677100	-1.19563300

2a-2’’

Bi	-0.06072700	0.03352600	-0.44325300
C	-0.44279600	-1.59218200	0.98855800
C	0.21743100	-1.56601400	2.21782500
C	-1.38578000	-2.57245200	0.67930900
C	-0.09750200	-2.53993900	3.16573900
H	0.98106000	-0.81601100	2.43202100
C	-1.68203600	-3.54389800	1.63803500
H	-1.89397800	-2.58473100	-0.28690200
C	-1.04240700	-3.52446200	2.87598000
H	0.40766300	-2.52983700	4.13260800
H	-2.41665400	-4.31734600	1.40977500
H	-1.27782000	-4.28618600	3.62076000
C	0.62188900	1.73900200	0.72466000
C	1.88477700	2.29442800	0.50813700
C	-0.24274200	2.26359100	1.68998900
C	2.29797500	3.36968700	1.29421300
H	2.54870200	1.90610800	-0.26726000
C	0.18515800	3.33575400	2.47333000
H	-1.24415100	1.84958300	1.82773500
C	1.45213100	3.88481500	2.27625300
H	3.28476300	3.80624800	1.13445700
H	-0.47902000	3.74577400	3.23547800
H	1.78020500	4.72662000	2.88784000
O	-2.13424600	0.84421700	-0.31293700
O	1.91968200	-0.29336800	-1.39589700
C	2.82390700	-0.64645700	-0.54447400
C	-2.90912200	0.10897000	-1.02121200
O	-2.57373900	-0.84269100	-1.69740600
O	2.66655600	-0.85382300	0.63468500
C	4.22764000	-0.70326100	-1.18365300
C	-4.38803400	0.54966500	-0.95033200
F	-5.16220400	-0.27001800	-1.63756000
F	-4.51839600	1.77519500	-1.44604400
F	-4.79941500	0.56307900	0.31298600
F	4.22256900	-1.41414400	-2.30215400
F	4.61272700	0.54073000	-1.48171500
F	5.11145700	-1.22384200	-0.35198600

2a-3’’

Bi	0.06523200	-0.07895100	-0.79200800
C	0.12792500	1.72103400	0.46003700
C	-0.89093800	2.65932200	0.27222300
C	1.13112200	1.89009400	1.41528200
C	-0.91808800	3.78391900	1.09796000
H	-1.67811800	2.51855900	-0.47433000
C	1.07741300	3.01809000	2.23545200
H	1.94246600	1.17216300	1.55132400
C	0.06257400	3.96087400	2.07445800
H	-1.71244300	4.52059100	0.97003600
H	1.84473500	3.15726900	2.99799600
H	0.03909700	4.84585900	2.71221800
C	-0.21044800	-1.90790600	0.37483500
C	0.84177800	-2.35291200	1.17972400
C	-1.43956100	-2.57027900	0.31595000

C	0.62482100	-3.46756900	1.99160300
H	1.80949000	-1.84353600	1.18981600
C	-1.62698300	-3.69108600	1.12640200
H	-2.25267800	-2.22172400	-0.32800100
C	-0.60047100	-4.13394400	1.96175100
H	1.42607600	-3.81671500	2.64448500
H	-2.58242800	-4.21695000	1.09853100
H	-0.75478600	-5.01241300	2.59030600
O	2.19636600	-0.54717000	-0.84347500
O	-2.04759500	0.12446700	-1.04419700
S	-3.48944500	0.31404300	-0.48171400
O	-3.97052600	1.63329200	-0.85215700
O	-4.27649400	-0.85952700	-0.81906900
S	3.50891700	0.08374700	-0.29890500
O	3.56647700	1.49842900	-0.61986100
O	3.66263800	-0.30988500	1.10210400
C	4.73725300	-0.77431200	-1.24902000
H	4.63294000	-1.84755200	-1.06015200
H	4.57708600	-0.53291400	-2.30489200
H	5.71110300	-0.40640100	-0.90644700
C	-3.26064400	0.30491200	1.28849600
H	-2.82313000	-0.65338700	1.59214400
H	-2.62362400	1.14854300	1.57799800
H	-4.26305900	0.41866500	1.71785000

2a-4’’

Bi	-0.00000200	0.92398800	-0.00017800
C	-1.76967400	-0.31999200	0.00012800
C	-2.31931400	-0.71471700	-1.22031900
C	-2.31896300	-0.71462700	1.22076200
C	-3.43927500	-1.54644100	-1.21180200
H	-1.86490100	-0.37214800	-2.15242600
C	-3.43892700	-1.54635000	1.21262800
H	-1.86426900	-0.37200900	2.15271400
C	-3.99477800	-1.95895900	0.00050800
H	-3.88084200	-1.87200000	-2.15505400
H	-3.88022000	-1.87184500	2.15603100
H	-4.87288800	-2.60681800	0.00065800
C	1.76967500	-0.31999800	-0.00000900
C	2.31948400	-0.71400800	1.22059200
C	2.31878800	-0.71533800	-1.22049300
C	3.43944500	-1.54573700	1.21239900
H	1.86521000	-0.37088400	2.15256200
C	3.43875800	-1.54705400	-1.21203100
H	1.86395100	-0.37327300	-2.15257700
C	3.99477900	-1.95895600	0.00025000
H	3.88115200	-1.87074800	2.15577500
H	3.87992200	-1.87310000	-2.15530400
H	4.87289000	-2.60681300	0.00035300
F	-0.00006400	1.06138000	-2.04214500
F	0.00007900	1.06214400	2.04173800

2a-5’’

Bi	-0.13794600	0.82853500	0.00024100
C	-1.21218700	-1.06680700	-0.00055800
C	-0.53018100	-2.28574000	-0.00141500
C	-2.61044400	-1.04311600	-0.00017800
C	-1.24908800	-3.48237800	-0.00185700
H	0.56275300	-2.31469900	-0.00173600
C	-3.32294000	-2.24249900	-0.00064900
H	-3.14186500	-0.08860700	0.00056800
C	-2.64296900	-3.46038800	-0.00148200
H	-0.71586200	-4.43442200	-0.00250900
H	-4.41407900	-2.22447100	-0.00035000
H	-3.20254600	-4.39726500	-0.00183500
C	2.03743700	-0.03814400	0.00021100
C	2.63418200	-0.31747100	-1.22358200
C	2.63359500	-0.31953300	1.22382000
C	3.88705600	-0.93939500	-1.21076600
H	2.13455100	-0.07782800	-2.16416100
C	3.88648500	-0.94141600	1.21050000
H	2.13349300	-0.08152100	2.16455300
C	4.50798600	-1.24748700	-0.00024300
H	4.37607900	-1.18108100	-2.15613200
H	4.37510300	-1.18466900	2.15567600
H	5.48723600	-1.72869200	-0.00040000
O	-0.00245200	0.63464200	2.40951400
O	-1.74128800	1.61844200	-1.52020400
C	-1.10537300	1.20803700	-2.52397700
C	-1.10586500	1.20512000	2.52496000
O	-1.74149900	1.61637300	1.52132900
O	-0.00218300	0.63699300	-2.40855100
H	-1.54548600	1.36232100	-3.52956100
H	-1.54660700	1.35820200	3.53047100

2a-6’’

Bi	0.08322800	0.00722300	-0.74631900
C	0.95941100	-0.01434900	1.25176600

C	2.35343100	0.00278400	1.36717100
C	0.16372900	-0.04892400	2.39925700
C	2.94635700	-0.01354400	2.63027200
H	2.97392100	0.02961900	0.46826500
C	0.76206900	-0.06506800	3.66058200
H	-0.92677800	-0.06341400	2.32156900
C	2.15131600	-0.04729700	3.77591500
H	4.03410100	-0.00000100	2.71913000
H	0.13833100	-0.09166300	4.55567400
H	2.61661000	-0.05997500	4.76302500
C	-2.18052900	0.00861000	-0.07629400
C	-2.80607500	-1.21496000	0.12775500
C	-2.79419200	1.23217200	0.16115100
C	-4.11498900	-1.20239700	0.62165200
H	-2.28636400	-2.15432900	-0.07101400
C	-4.10320600	1.21875400	0.65470900
H	-2.26560900	2.17155400	-0.01257100
C	-4.75804000	0.00801400	0.88143700
H	-4.63032400	-2.14756300	0.80212600
H	-4.60936000	2.16358800	0.86104800
H	-5.78026200	0.00778100	1.26304100
O	1.74208500	1.51074100	-1.33548500
O	-0.07761400	-2.39312400	-0.54182700
C	1.08194800	-2.52275400	-0.99854500
C	1.09983000	2.53508200	-0.95419400
O	-0.06171800	2.40686100	-0.50290900
O	1.73041000	-1.49740200	-1.36537100
C	1.73902600	-3.87254300	-1.07832400
H	2.39733100	-3.91874100	-1.95259100
H	2.35535200	-3.99733600	-0.17646900
H	0.98044400	-4.66132800	-1.10294900
C	1.76735400	3.88091200	-1.01059800
H	1.01471300	4.67555900	-1.02874500
H	2.37832700	3.98839000	-0.10285700
H	2.43174500	3.93477400	-1.87978300

2a-7’’

Bi	-0.00004700	0.31593100	-0.55009600
C	0.00006100	0.02899300	1.61074100
C	-0.00036700	-1.27653600	2.11151200
C	0.00070100	1.11586100	2.48799900
C	-0.00016400	-1.49066200	3.49007900
H	-0.00087800	-2.12501700	1.42338700
C	0.00089500	0.89561900	3.86647200
H	0.00104100	2.14089200	2.10764700
C	0.00046800	-0.40571400	4.36650600
H	-0.00050200	-2.50976900	3.88042700
H	0.00138000	1.74568100	4.55079300
H	0.00063200	-0.57527300	5.44455300
C	-0.00006600	2.68228500	-0.54242900
C	-1.22393800	3.33760900	-0.50192700
C	1.22383600	3.33757800	-0.50222700
C	-1.21079000	4.73188800	-0.38716600
H	-2.16329100	2.78267800	-0.53953100
C	1.21075300	4.73185700	-0.38746500
H	2.16317300	2.78263400	-0.54004300
C	-0.00000300	5.42247900	-0.33142800
H	-2.15579100	5.27630500	-0.34236200
H	2.15578000	5.27624800	-0.34289200
H	0.00001900	6.51020600	-0.24575900
O	1.50012400	-1.44135300	-0.69545000
O	-2.40603900	0.52037700	-0.38795700
C	-2.53058000	-0.72025700	-0.52622400
C	2.53049300	-0.72029800	-0.52631200
O	2.40597900	0.52032000	-0.38787300
O	-1.50021300	-1.44134600	-0.69524000
C	-3.87471000	-1.36351600	-0.49283900
C	-5.00966900	-0.58001100	-0.26813900
C	-3.99160000	-2.74272200	-0.68582800
C	-6.26577900	-1.17935800	-0.23757900
H	-4.88472300	0.49279900	-0.11828300
C	-5.24999500	-3.33777100	-0.65570300
H	-3.08670400	-3.32657000	-0.85761900
C	-6.38489300	-2.55657000	-0.43181500
H	-7.15562200	-0.57351100	-0.06150000
H	-5.34846000	-4.41358100	-0.80668800
H	-7.37032600	-3.02491300	-0.40795000
C	3.87461800	-1.36358600	-0.49298100
C	3.99147800	-2.74278200	-0.68606100
C	5.00959500	-0.58012000	-0.26823900
C	5.24986000	-3.33786100	-0.65598200
H	3.08656600	-3.32659600	-0.85788700
C	6.26569300	-1.17949500	-0.23773700
H	4.88467300	0.49268100	-0.11830300
C	6.38477700	-2.55669800	-0.43205900
H	5.34830000	-4.41366400	-0.80703000
H	7.15555200	-0.57367700	-0.06163400
H	7.37020100	-3.02506300	-0.40823400

2a-8''

Bi	-0.00002600	-0.00026000	-0.52767800
C	0.95891800	-1.42458200	0.85677200
C	0.44688400	-2.71506000	1.00346100
C	2.06728800	-1.00842100	1.59981300
C	1.05262900	-3.59604400	1.90180600
H	-0.42905700	-3.02750900	0.43131000
C	2.66214600	-1.89477500	2.49962200
H	2.46357700	0.00414100	1.48345600
C	2.15816200	-3.18721700	2.64646800
H	0.65576300	-4.60571600	2.02027100
H	3.52101200	-1.57186000	3.09073000
H	2.62829800	-3.87814600	3.34811000
C	-0.95880100	1.42336900	0.85747600
C	-2.06679900	1.00682100	1.60087000
C	-0.44705300	2.71396500	1.00423100
C	-2.66156200	1.89287400	2.50103500
H	-2.46288400	-0.00581400	1.48448400
C	-1.05269900	3.59465200	1.90293300
H	0.42860600	3.02673600	0.43182700
C	-2.15786400	3.18542300	2.64791800
H	-3.52013600	1.56964200	3.09239700
H	-0.65604300	4.60440400	2.02142800
H	-2.62793000	3.87611400	3.34984100
O	1.84538100	1.31774500	-0.50000300
O	-1.84606600	-1.31830900	-0.49971200
C	-2.47829000	-0.83433100	-1.50823000
C	2.47816200	0.83439700	-1.50864800
O	1.98462400	-0.06149800	-2.20535200
O	-1.98400800	0.06158800	-2.20457800
C	-3.87197100	-1.35729400	-1.76420100
H	-4.20922500	-0.98562900	-2.74127300
H	-3.82708300	-2.45610000	-1.78758700
C	3.87158000	1.35843100	-1.76390700
H	4.20932300	0.98749800	-2.74109300
H	3.82601600	2.45722000	-1.78673700
C	-4.82298800	-0.90019800	-0.65232900
C	-4.91189700	0.62718600	-0.57450700
C	-6.21740400	-1.50543200	-0.83478100
H	-4.41258600	-1.27115500	0.30506400
C	-5.85983200	1.07543300	0.53628100
H	-5.27591400	1.00634000	-1.54681200
H	-3.91334900	1.06822300	-0.42640500
C	-7.17610200	-1.05660800	0.26851100
H	-6.61337700	-1.18743000	-1.81622600
H	-6.14908000	-2.60453300	-0.86186900
C	-7.25101400	0.46797500	0.35451900
H	-5.91523000	2.17387100	0.56909600
H	-5.44867600	0.75171700	1.50895600
H	-8.17621800	-1.48326000	0.09931200
H	-6.82197400	-1.45538000	1.23502400
H	-7.91656900	0.77337100	1.17598600
H	-7.69591500	0.86076900	-0.57628600
C	4.82262700	0.90135300	-0.65206200
C	4.91245700	-0.62602300	-0.57498100
C	6.21671900	1.50751100	-0.83391000
H	4.41181800	1.27158600	0.30544000
C	5.86040900	-1.07424300	0.53579800
H	5.27693400	-1.00445100	-1.54739700
H	3.91414700	-1.06775500	-0.42735600
C	7.17546000	1.05870300	0.26935300
H	6.61308600	1.19025200	-1.81543600
H	6.14774800	2.60658500	-0.86044800
C	7.25127000	-0.46587500	0.35462800
H	5.91643800	-2.17266400	0.56810900
H	5.44886100	-0.75122100	1.50853900
H	8.17535700	1.48603700	0.10057200
H	6.82089600	1.45678500	1.23599100
H	7.91684000	-0.77126400	1.17608500
H	7.69659700	-0.85795100	-0.57627500

Ph

C	0.00002000	-1.40098600	-0.00030600
C	-1.22440900	-0.76968300	0.00006900
C	1.22447300	-0.76959600	0.00051000
C	-1.21251900	0.63122900	0.00030000
H	-2.16467500	-1.32435700	-0.00087300
C	1.21249800	0.63124300	-0.00015400
H	2.16470100	-1.32433300	-0.00034100
C	-0.00004800	1.32323700	-0.00001800
H	-2.15579400	1.18091800	-0.00014100
H	2.15569400	1.18107300	-0.00064500
H	-0.00002100	2.41403700	-0.00040800

6. Determination of the Absolute Configuration

Lowe–Brewster model[18,19]

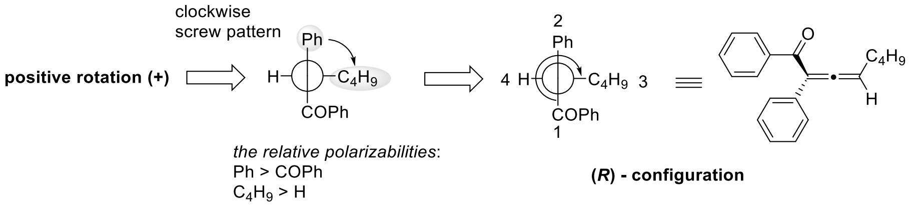

The ECD spectra of 3f

Calculations of the ECD spectra of (*R*)-**3f** were carried out using the PBE1PBE-SMD(2-Propanol)/6-311G** level with Gaussian 16. Electronic excitation energies (nm) and rotational strengths (Δε) were calculated for **3f**. In order to cover the 210–500 nm range, 95 transitions were calculated. As shown in below, the simulated spectra are in good agreement with the experimental spectral data, and the *R* configuration could be reliably assigned to compound **3f**.

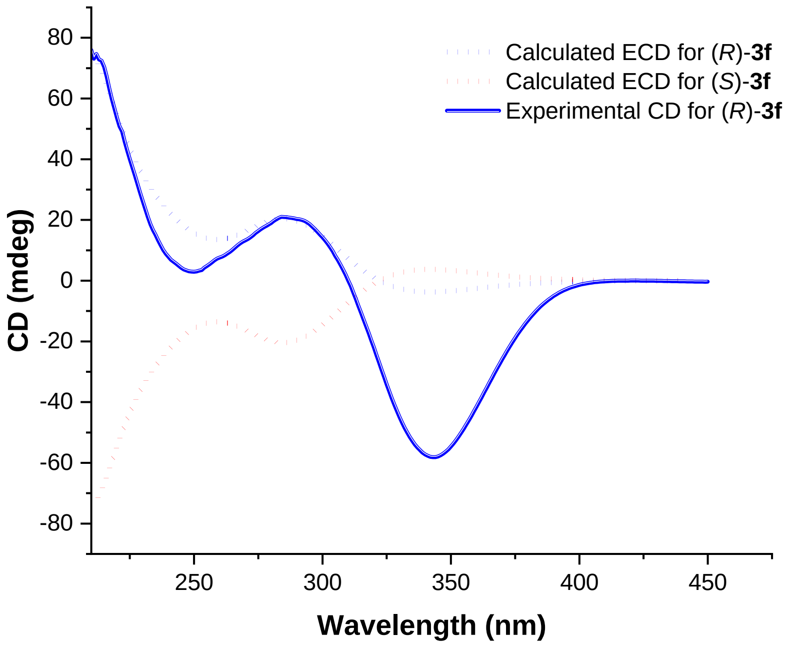

Conformation analysis（Boltzmann% >0.95%）：

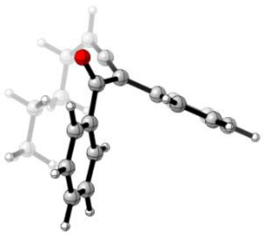	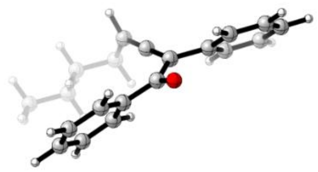	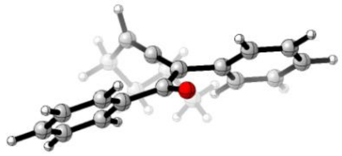
**3f-1** (5.51%)	**3f-2** (9.81%)	**3f-3** (0.98%)

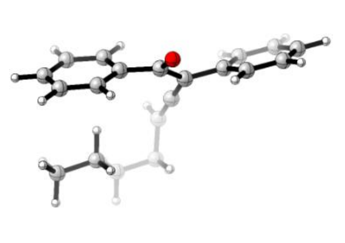	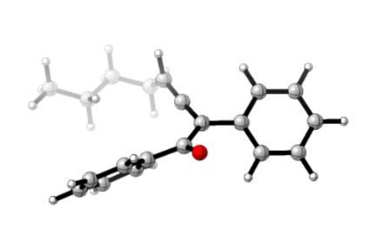	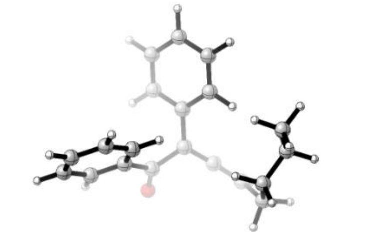
**3f-4** (17.43%)	**3f-5** (57.63%)	**3f-6** (0.95%)
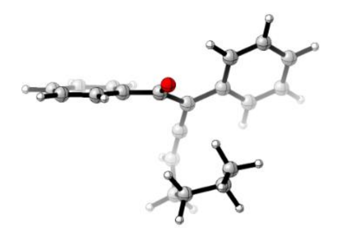	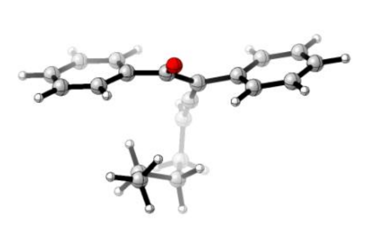	
**3f-7** (1.42%)	**3f-8** (6.27%)	

3f-1

C	1.44987200	-2.73846600	1.25017600
C	-0.31923900	-0.82266400	0.98588400
C	0.09799800	0.51628000	1.54844600
C	-1.61901600	-0.97052600	0.28069300
C	-2.67002800	-0.08423800	0.54377700
C	-3.89259200	-0.22683100	-0.10760800
C	-4.08142000	-1.25572800	-1.02705100
C	-3.03933500	-2.14438100	-1.29192000
C	-1.81638500	-2.00156100	-0.64642000
C	0.10271700	1.69407600	0.62927700
O	0.47037900	0.62142100	2.70424700
C	0.16265300	2.97727300	1.18298400
C	0.21964000	4.09031500	0.35309500
C	0.23544700	3.92556200	-1.03263100
C	0.18871200	2.64815300	-1.58827100
C	0.11336400	1.53208500	-0.75992900
C	0.54325900	-1.80717200	1.13756800
C	3.26695000	0.25555800	-1.77582000
C	3.12386800	-0.65589000	-0.56052800
C	2.59545800	-2.03969800	-0.93964800
C	2.52436400	-3.02632700	0.22446800
H	1.42623200	-3.37578600	2.13477800
H	-2.53800000	0.71566300	1.26801300
H	-4.69941000	0.46662400	0.10852600
H	-5.03425000	-1.36539100	-1.53537200
H	-3.17763900	-2.94744300	-2.00939500
H	-1.00244000	-2.68992700	-0.85861900
H	0.16151800	3.08704900	2.26295200
H	0.25629500	5.08630600	0.78277800
H	0.28563700	4.79555300	-1.68024700
H	0.20949400	2.52049100	-2.66594400
H	0.07870200	0.53563200	-1.19240400
H	3.58099900	1.26346200	-1.48796900
H	4.00911900	-0.14186200	-2.47667900
H	2.31802500	0.34403000	-2.31599500
H	2.45817200	-0.18692600	0.17388600
H	4.09763600	-0.76740000	-0.06677600
H	3.24780400	-2.46486200	-1.71205200
H	1.60032100	-1.93838200	-1.39601100
H	3.48966600	-3.06102900	0.74549800
H	2.35135400	-4.04209500	-0.15219700

3f-2

C	0.48803300	-1.54935700	1.22624000
C	-1.29233600	0.15386000	0.32101300
C	-0.90372300	1.58806200	0.13609200
C	-2.68817300	-0.29997600	0.04704900
C	-3.80716600	0.43260800	0.46171700
C	-5.09059200	-0.04427800	0.20412000
C	-5.27612600	-1.25207400	-0.46438100
C	-4.16650200	-1.98887900	-0.87468800
C	-2.88337900	-1.51458400	-0.62237100
C	0.53715400	1.93471500	-0.08338600
O	-1.74106100	2.47696400	0.16973000
C	0.99025400	3.16582000	0.40314300
C	2.30743200	3.55829200	0.19637800
C	3.17452500	2.73520700	-0.52246800

C	2.72098100	1.52072900	-1.03342800
C	1.40737900	1.11523200	-0.80975400
C	-0.39088400	-0.70090900	0.77324400
C	5.28796500	-2.38670600	-0.07055400
C	3.82810900	-2.73975000	-0.33632400
C	2.87525600	-2.01454900	0.61024700
C	1.40711300	-2.35800900	0.34244300
H	0.60446200	-1.65205100	2.30615100
H	-3.67220100	1.36785100	0.99033000
H	-5.94990300	0.53140800	0.53469700
H	-6.27874200	-1.61800000	-0.66320100
H	-4.29904100	-2.93114800	-1.39759300
H	-2.01718700	-2.08337900	-0.95049900
H	0.30275900	3.80178600	0.95223900
H	2.65855500	4.50628900	0.59143700
H	4.20182500	3.04412000	-0.68964200
H	3.38895400	0.88630000	-1.60858100
H	1.05456500	0.17589000	-1.22407200
H	5.95906300	-2.90883300	-0.75871600
H	5.45661400	-1.31081700	-0.18942400
H	5.57739200	-2.65747800	0.95032200
H	3.68271100	-3.82310900	-0.23940200
H	3.56868300	-2.48245100	-1.37146200
H	3.01453100	-0.93042300	0.50917500
H	3.12231100	-2.26586000	1.65013400
H	1.23949000	-3.42521700	0.53349400
H	1.16171300	-2.17331300	-0.70922100

3f-3

C	-0.17712600	1.51426100	1.68799200
C	0.15260400	-0.74242300	0.38720300
C	-1.05529600	-1.51876600	-0.03603500
C	1.51613700	-1.29377900	0.13448500
C	1.87380000	-2.59208600	0.51540000
C	3.16800400	-3.05409000	0.28865800
C	4.11623500	-2.23192800	-0.31797200
C	3.76500700	-0.93928900	-0.70157100
C	2.47132700	-0.47654300	-0.47936900
C	-2.36718300	-0.81259700	-0.18693100
O	-0.98243800	-2.72096800	-0.24020400
C	-2.46668300	0.50163700	-0.65663100
C	-3.71834700	1.08841100	-0.82015400
C	-4.87255400	0.37307800	-0.50596800
C	-4.77766700	-0.94031700	-0.04664700

C	-3.52975900	-1.53532800	0.09986100
C	-0.00502800	0.40202400	1.02898200
C	3.05193500	3.30381400	-0.97237000
C	2.13550400	3.13544000	0.23526400
C	0.65927600	3.07687700	-0.15514100
C	-0.26770900	2.88466100	1.04609200
H	-0.31030800	1.44235700	2.76749300
H	1.14353600	-3.23246500	0.99651700
H	3.43781100	-4.06079600	0.59327600
H	5.12354600	-2.59804100	-0.49112500
H	4.49411400	-0.29143300	-1.17931500
H	2.18504900	0.52399200	-0.79158700
H	-1.57124400	1.05586000	-0.92150900
H	-3.79150900	2.10402800	-1.19641500
H	-5.84693400	0.83676300	-0.62582100
H	-5.67614300	-1.49980900	0.19386400
H	-3.44167300	-2.55984600	0.44822200
H	4.10478700	3.33045000	-0.67637700
H	2.92537900	2.48073800	-1.68497600
H	2.82971100	4.23460700	-1.50531600
H	2.28572800	3.97011500	0.93168400
H	2.40502700	2.22340700	0.78243400
H	0.50088500	2.26274000	-0.87701100
H	0.38530000	4.00548400	-0.67016200
H	-1.31268100	3.04353800	0.74759300
H	-0.04574500	3.63770400	1.80927700

3f-4

C	-0.26634200	2.00431100	0.71006100
C	1.16854300	-0.10563100	0.09008400
C	0.44303300	-1.35866400	-0.29052700
C	2.65775900	-0.03180400	0.02965800
C	3.38989800	-0.57508500	-1.03344900
C	4.77798100	-0.46042200	-1.05817600
C	5.45265900	0.19493700	-0.03067800
C	4.72966700	0.74255800	1.02739000
C	3.34363500	0.62792900	1.05705600
C	-0.98345300	-1.51847900	0.13698000
O	0.97679500	-2.22234000	-0.96911500
C	-1.41771800	-1.18684900	1.42410400
C	-2.75457900	-1.36099600	1.77110400
C	-3.66329200	-1.84594600	0.83098500
C	-3.22981500	-2.18769100	-0.44994400
C	-1.88902500	-2.03997100	-0.79127900

C	0.45310000	0.95574000	0.42591400
C	-4.43026100	1.46028800	-0.84017000
C	-2.90681800	1.51570900	-0.80218300
C	-2.37810100	2.87582400	-0.35153600
C	-0.84545800	2.94767700	-0.31904900
H	-0.50913700	2.19342500	1.75757600
H	2.87362900	-1.07764100	-1.84168800
H	5.33328100	-0.88217100	-1.89055100
H	6.53472800	0.27981500	-0.05464400
H	5.24482300	1.25487700	1.83420200
H	2.77976900	1.04702000	1.88608900
H	-0.71031700	-0.80538900	2.15404900
H	-3.08832200	-1.11309200	2.77375500
H	-4.70899200	-1.96397700	1.09869300
H	-3.93576200	-2.57139500	-1.17991000
H	-1.53727300	-2.30754200	-1.78325400
H	-4.78485800	0.48564600	-1.18961000
H	-4.85156400	1.63030100	0.15686300
H	-4.83910800	2.22537000	-1.50922700
H	-2.49842300	1.28239900	-1.79480800
H	-2.53371900	0.73979500	-0.12426200
H	-2.77349100	3.11009900	0.64598300
H	-2.74853600	3.65652900	-1.02731900
H	-0.53039200	3.96714000	-0.07046100
H	-0.43916700	2.70689200	-1.30662100

3f-5

C	0.39311000	2.03774600	-0.59262900
C	-1.18413200	-0.03410800	-0.26200100
C	-0.64278600	-1.41646700	-0.47855100
C	-2.65001200	0.14580000	-0.06081000
C	-3.39963300	-0.81707100	0.62608400
C	-4.76091700	-0.62627500	0.84475300
C	-5.39352000	0.52527100	0.38094800
C	-4.65416600	1.48782000	-0.30426500
C	-3.29377200	1.29848000	-0.52491300
C	0.77262200	-1.72499800	-0.11210900
O	-1.35030300	-2.27807300	-0.97748400
C	1.43930000	-2.71521000	-0.84304600
C	2.73784200	-3.07573100	-0.50643700
C	3.36644100	-2.47371500	0.58481000
C	2.69684000	-1.50713300	1.33139000
C	1.40630200	-1.11983300	0.97716000
C	-0.36887400	0.99650600	-0.40380800

C	4.86073300	1.98256600	-0.37389300
C	3.41311100	1.74518100	0.04200100
C	2.59909100	3.03425000	0.10601000
C	1.13181400	2.80264900	0.48003500
H	0.50574400	2.39748000	-1.61831600
H	-2.91626000	-1.71632800	0.99425900
H	-5.32770000	-1.38048600	1.38215300
H	-6.45588600	0.67084000	0.55041800
H	-5.13851400	2.38637900	-0.67420300
H	-2.72089600	2.04699300	-1.06547400
H	0.93090300	-3.18679600	-1.67831100
H	3.25907000	-3.83024400	-1.08704900
H	4.37686300	-2.76400300	0.85613600
H	3.17972700	-1.05133800	2.19009800
H	0.88408800	-0.36859400	1.56212300
H	5.42647900	1.04632100	-0.39845300
H	4.91393500	2.43241300	-1.37103200
H	5.36397000	2.66053000	0.32384200
H	3.38531100	1.26108500	1.02627400
H	2.93685900	1.04408300	-0.65634100
H	2.64461800	3.54801500	-0.86378100
H	3.04836800	3.71273800	0.84120900
H	0.63261600	3.77066900	0.60749700
H	1.05675800	2.27044600	1.43445600

3f-6

C	2.37960800	-1.62446000	-1.54895300
C	0.16657800	-0.29113900	-1.09099900
C	-1.08843600	-1.13007100	-1.12065600
C	0.09467800	1.16950700	-0.82040900
C	1.14103300	1.82697400	-0.16427200
C	1.07747800	3.19608600	0.07278500
C	-0.03660200	3.92680800	-0.33806800
C	-1.08443300	3.27910600	-0.98943000
C	-1.02105700	1.90924500	-1.22972700
C	-2.11363800	-0.92867100	-0.05088900
O	-1.24844000	-1.98136700	-1.97934000
C	-1.78819500	-0.36633000	1.18793400
C	-2.76288900	-0.25003000	2.17428100
C	-4.06528500	-0.67845100	1.92269500
C	-4.39335000	-1.23876100	0.68803900
C	-3.41789600	-1.37243800	-0.29288900
C	1.29575300	-0.93221500	-1.32708400
C	3.83644200	0.36523100	2.25091200

C	4.03672600	-0.42625300	0.96304500
C	3.14543100	-1.66592900	0.88932100
C	3.19023100	-2.33759400	-0.48368200
H	2.70225200	-1.73084000	-2.58524600
H	2.00075500	1.25300700	0.16854400
H	1.89614100	3.69082100	0.58670400
H	-0.08898400	4.99441200	-0.14884600
H	-1.95430300	3.84114600	-1.31505700
H	-1.84017000	1.41387000	-1.74488100
H	-0.77424400	-0.03186400	1.38752900
H	-2.50669100	0.17712500	3.13847400
H	-4.82604600	-0.57683300	2.69071000
H	-5.40761900	-1.57275300	0.49380100
H	-3.65427800	-1.81476100	-1.25561700
H	4.49588400	1.23712100	2.29427000
H	2.80338500	0.72106400	2.33726700
H	4.04493800	-0.25680200	3.12811900
H	5.08484500	-0.73946100	0.87874400
H	3.84205200	0.21512000	0.09385200
H	2.10766300	-1.39521800	1.12920700
H	3.46310000	-2.38239600	1.65563200
H	2.81845600	-3.36743600	-0.42040400
H	4.22732700	-2.40807900	-0.82980000

3f-7

C	0.27979800	1.83637300	-1.68326400
C	-0.31662100	-0.32644100	-0.32320700
C	0.73285700	-0.74426100	0.66731400
C	-1.67595600	-0.93653300	-0.30500300
C	-1.95629500	-2.08984600	0.43921400
C	-3.22998900	-2.65355000	0.41589400
C	-4.24400500	-2.08147200	-0.34700500
C	-3.97117400	-0.94109600	-1.10132500
C	-2.70049500	-0.37860500	-1.08404000
C	2.17031900	-0.75682000	0.25856200
O	0.41451200	-1.01790900	1.81535900
C	2.56926400	-0.93540100	-1.07028300
C	3.92352300	-0.98579400	-1.38757600
C	4.88171400	-0.84681500	-0.38519600
C	4.48751800	-0.67062200	0.94155100
C	3.13645300	-0.63551200	1.26363300
C	-0.01514900	0.72851000	-1.06115000
C	-1.93014500	2.16370200	2.04116600
C	-1.69353700	2.78459000	0.66870000
C	-0.22218600	3.15011200	0.43768100
C	0.16962200	3.20439600	-1.03800500
H	0.65233100	1.78577700	-2.70656600
H	-1.18258700	-2.55167000	1.04043200
H	-3.42662500	-3.54743900	1.00003500
H	-5.23612500	-2.52182700	-0.35908700
H	-4.74882200	-0.48927600	-1.70954600
H	-2.49172000	0.49816900	-1.69091300
H	1.82491000	-1.05451100	-1.85072600
H	4.23072400	-1.13494500	-2.41773800
H	5.93739700	-0.87927200	-0.63687200
H	5.23414800	-0.56287500	1.72193600
H	2.81333100	-0.50552700	2.29169100
H	-2.99029700	1.95319900	2.21129400
H	-1.38094200	1.21885000	2.13317800
H	-1.58585800	2.82810900	2.84152600
H	-2.31076500	3.68291100	0.54708600
H	-2.02834700	2.07934800	-0.10017200
H	0.42178500	2.41476200	0.93988500
H	-0.00959100	4.11750000	0.90569800
H	1.14008400	3.69905100	-1.16093900
H	-0.55221500	3.80344000	-1.60904700

3f-8

C	0.11512300	1.75758300	-1.73173600
C	-0.79951900	-0.28502100	-0.35960300
C	0.13161900	-1.22335400	0.34238900
C	-2.27368900	-0.41735200	-0.15765400
C	-2.82884200	-0.51941200	1.12358000
C	-4.20935900	-0.60403100	1.28346900
C	-5.05044100	-0.58787400	0.17216600
C	-4.50418500	-0.48006300	-1.10488100
C	-3.12420900	-0.39536100	-1.26801000
C	1.57332400	-1.27062500	-0.05494800
O	-0.26623600	-1.91682100	1.26674900
C	1.99821400	-1.16944500	-1.38336800
C	3.35660200	-1.22676000	-1.68262300
C	4.29332000	-1.36943000	-0.66043100
C	3.87199100	-1.48280100	0.66429700
C	2.51486400	-1.44899200	0.96475200
C	-0.32594600	0.71664200	-1.08195900
C	1.64240500	1.73535300	2.36773700
C	1.77879600	2.23910500	0.93521300
C	0.53411700	2.99140700	0.45259600

C	0.45362500	3.07870600	-1.07045700	H	2.17135700	-1.54333100	1.99057000
H	0.24843000	1.67712100	-2.81067800	H	2.56132300	1.25273300	2.71453400
H	-2.17961000	-0.52253600	1.99174100	H	1.41436400	2.55679500	3.05617300
H	-4.62909100	-0.67758600	2.28216400	H	0.82847900	1.00376300	2.44405300
H	-6.12619100	-0.65635700	0.30163000	H	1.97433000	1.38768500	0.27213700
H	-5.15144800	-0.46527900	-1.97649900	H	2.64982200	2.90039500	0.85027100
H	-2.69528900	-0.31530800	-2.26315800	H	0.52965600	4.00177400	0.87623800
H	1.27072700	-1.06935800	-2.18212500	H	-0.36946600	2.49375500	0.82991800
H	3.68389400	-1.15960100	-2.71526700	H	1.40878000	3.42887000	-1.48105300
H	5.35253500	-1.40000300	-0.89692500	H	-0.30007000	3.81231200	-1.38113300
H	4.60049100	-1.60092800	1.46024800				

7. General procedure for preparing allenones

Method A:

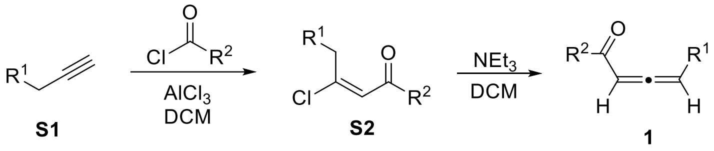

Allenone **1b-1e** and **1j**, **1k**, **1x** were prepared from corresponding compounds **S2** via the general synthetic route reported in literatures[1,3].

Method B:

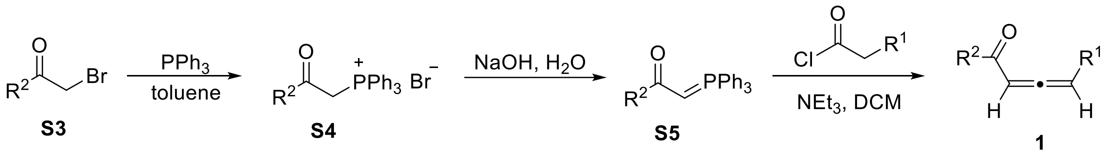

Allenone **1a**, **1f-1i**, and **1l-1w**, **1y-1ab** were prepared from corresponding compounds **S5** via the general synthetic route reported in literature[2].

Compounds **1** was isolated as a mixture of stereoisomers (**1** and **1'**) but was used without further purification.

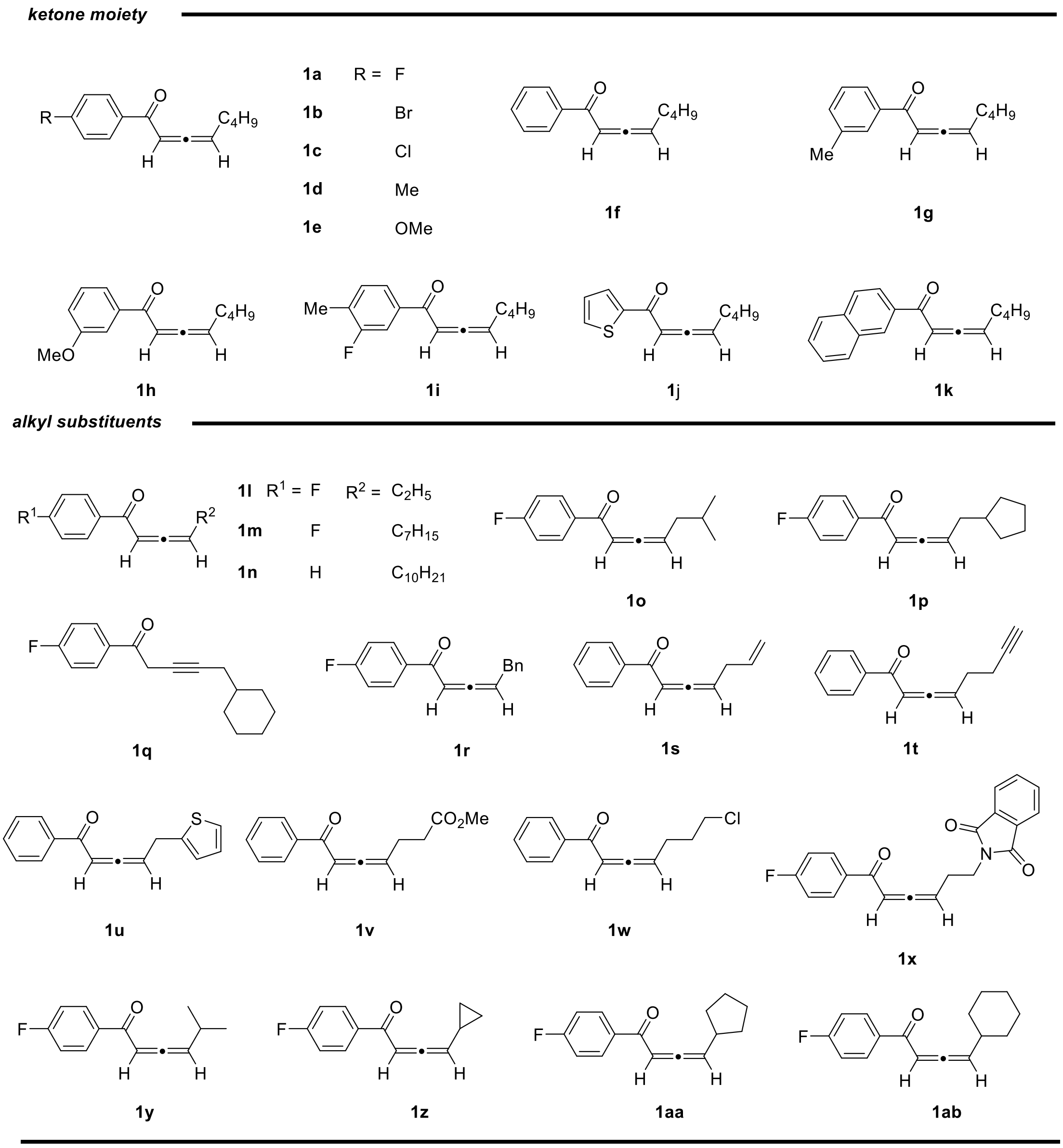

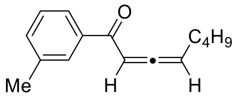

1g

1-(*m*-tolyl)octa-2,3-dien-1-one (1g):

(**Method B**: 1:30 ethyl acetate:hexanes as eluent, Rf = 0.4, yellow oil)

^{1}H NMR (400 MHz, $CDCl_3$) δ 7.70 – 7.63 (m, 2H), 7.38 – 7.29 (m, 2H), 6.49 – 6.21 (m, 1H), 5.59 (dd, *J* = 13.2, 7.2 Hz, 1H), 2.40 (s, 3H), 2.19 – 2.12 (m, 2H), 1.49 – 1.38 (m, 2H), 1.38 – 1.28 (m, 2H), 0.88 (t, *J* = 7.2 Hz, 3H). ^{13}C NMR (100 MHz, $CDCl_3$) δ 213.8, 192.2, 138.0, 137.8, 133.2, 129.2, 128.1, 125.9, 94.9, 94.0, 30.9, 27.4, 22.0, 21.3, 13.7. HRMS (ESI): m/z calcd. for $C_{15}H_{19}O^+$($[M+H]^+$) = 215.1430, found = 215.1429.

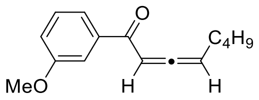

1h

1-(3-methoxyphenyl)octa-2,3-dien-1-one (1h):

(**Method B**: 1:15 ethyl acetate:hexanes as eluent, Rf = 0.3, yellow oil)

^{1}H NMR (400 MHz, $CDCl_3$) δ 7.48 – 7.44 (m, 1H), 7.41 – 7.39 (m, 1H), 7.33 (t, *J* = 7.9 Hz, 1H), 7.10 – 7.06 (m, 1H), 3.84 (s, 3H), 2.22 – 2.13 (m, 2H), 1.48 – 1.41 (m, 2H), 1.37 – 1.28 (m, 2H), 0.88 (t, *J* = 7.2 Hz, 3H). ^{13}C NMR (100 MHz, $CDCl_3$) δ 213.8, 191.7, 159.6, 139.1, 129.2, 121.2, 118.9, 113.1, 95.0, 93.9, 55.4, 30.9, 27.4, 22.0, 13.7. HRMS (ESI): m/z calcd. for $C_{15}H_{19}O_2^+$([M+H]$^+$) = 231.1380, found = 231.1380.

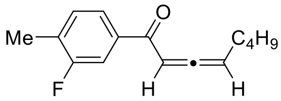

1i

1-(3-fluoro-4-methylphenyl)octa-2,3-dien-1-one (1i):

(**Method B**: 1:30 ethyl acetate:hexanes as eluent, Rf = 0.3, brown oil)

^{1}H NMR (400 MHz, $CDCl_3$) δ 7.80 – 7.65 (m, 2H), 7.03 (t, *J* = 8.8 Hz, 1H), 6.36 – 6.24 (m, 1H), 5.60 (dd, *J* = 13.2, 7.2 Hz, 1H), 2.31 (d, *J* = 2.0 Hz, 3H), 2.22 – 2.10 (m, 2H), 1.49 – 1.39 (m, 2H), 1.38 – 1.28 (m, 2H), 0.88 (t, *J* = 7.2 Hz, 3H). ^{13}C NMR (100 MHz, $CDCl_3$) δ 213.7, 190.6, 164.0 (d, *J* = 252.6 Hz), 133.7 (d, *J* = 3.3 Hz), 132.4 (d, *J* = 6.4 Hz), 128.5 (d, *J* = 9.1 Hz), 125.0 (d, *J* = 17.8 Hz), 114.8 (d, *J* = 23.0 Hz), 95.0, 93.8, 30.9, 27.4, 22.1, 14.5, 14.5, 13.7. ^{19}F NMR (471 MHz, $CDCl_3$) δ -110.3. HRMS (ESI): m/z calcd. for $C_{15}H_{18}FO^+$([M+H]$^+$) = 233.1336, found = 233.1335.

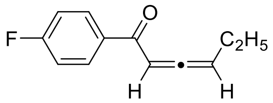

1l

1-(4-fluorophenyl)hexa-2,3-dien-1-one (1l):

(**Method B**: 1:30 ethyl acetate:hexanes as eluent, Rf = 0.3, yellow oil)

^{1}H NMR (400 MHz, $CDCl_3$) δ 7.91 (dd, *J* = 8.8, 5.6 Hz, 2H), 7.10 (t, *J* = 8.8 Hz, 1H), 6.47 – 6.21 (m, 1H), 5.76 – 5.45 (m, 1H), 2.29 – 2.03 (m, 2H), 1.07 (t, *J* = 7.2 Hz, 3H). ^{13}C NMR (100 MHz, $CDCl_3$) δ 213.6, 190.3, 165.4 (d, *J* = 253.9 Hz), 134.0 (d, *J* = 3.0 Hz), 131.2 (d, *J* = 9.1 Hz), 115.3 (d, *J* = 21.8 Hz), 96.8, 94.4, 21.1, 13.2. ^{19}F NMR (471 MHz, $CDCl_3$) δ -106.1. HRMS (ESI): m/z calcd. for $C_{12}H_{12}FO^+$([M+H]$^+$) = 191.0867, found = 191.0867.

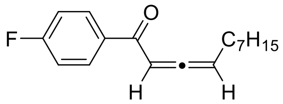

1m

1-(4-fluorophenyl)undeca-2,3-dien-1-one (1m):

(**Method B**: 1:30 ethyl acetate:hexanes as eluent, Rf = 0.3, yellow oil)

^{1}H NMR (400 MHz, $CDCl_3$) δ 7.91 (dd, *J* = 8.8, 5.6 Hz, 2H), 7.10 (t, *J* = 8.8 Hz, 2H), 6.41 – 6.13 (m, 1H), 5.61 (dd, *J* = 13.2, 7.2 Hz, 1H), 2.28 – 2.11 (m, 2H), 1.48 – 1.35 (m, 2H), 1.34 – 1.17 (m, 8H), 0.87 (t, *J* = 7.2 Hz, 3H). ^{13}C NMR (100 MHz, $CDCl_3$) δ 213.8, 190.5, 165.4 (d, *J* = 253.8 Hz), 134.0 (d, *J* = 3.0 Hz), 131.2 (d, *J* = 9.1 Hz), 115.3 (d, *J* = 21.8 Hz), 95.2, 93.9, 31.7, 29.0, 28.9, 28.8, 27.7, 22.6, 14.0. ^{19}F NMR (471 MHz, $CDCl_3$) δ -106.2. HRMS (ESI): m/z calcd. for $C_{17}H_{22}FO^+$($[M+H]^+$) = 261.1649, found = 261.165.

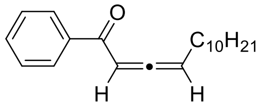

1n

1-phenyltetradeca-2,3-dien-1-one (1n):

(**Method B**: 1:30 ethyl acetate:hexanes as eluent, Rf = 0.3, yellow oil)

^{1}H NMR (400 MHz, $CDCl_3$) δ 7.87 (d, *J* = 6.8 Hz, 2H), 7.53 (t, *J* = 7.2 Hz, 1H), 7.43 (t, *J* = 7.2 Hz, 2H), 6.39 – 6.25 (m, 1H), 5.66 – 5.50 (m, 1H), 2.20 – 2.08 (m, 2H), 1.48 – 1.36 (m, 2H), 1.32 – 1.21 (m, 14H), 0.88 (t, *J* = 7.2 Hz, 3H). ^{13}C NMR (100 MHz, $CDCl_3$) δ 213.9, 192.1, 137.8, 132.5, 128.7, 128.2, 95.1, 94.0, 31.9, 29.6, 29.5, 29.3, 29.3, 29.0, 28.8, 27.8, 22.7, 14.1. HRMS (ESI): m/z calcd. for $C_{20}H_{29}O^+$($[M+H]^+$) = 285.2213, found = 285.2214.

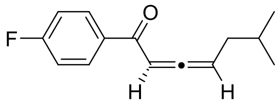

1o

1-(4-fluorophenyl)-6-methylhepta-2,3-dien-1-one (1o):

(**Method B**: 1:30 ethyl acetate:hexanes as eluent, Rf = 0.3, yellow oil)

^{1}H NMR (400 MHz, $CDCl_3$) δ 7.90 (dd, *J* = 8.8, 5.6 Hz, 1H), 7.10 (t, *J* = 8.8 Hz, 1H), 6.38 – 6.18 (m, 1H), 5.69 – 5.48 (m, 1H), 2.11 – 2.01 (m, 2H), 1.77 – 1.66 (m, 1H), 0.91 (d, *J* = 3.2 Hz, 3H), 0.90 (d, *J* = 3.2 Hz, 3H). ^{13}C NMR (100 MHz, $CDCl_3$) δ 214.1, 190.5, 165.4 (d, *J* = 253.8 Hz), 134.0 (d, *J* = 3.0 Hz), 131.2 (d, *J* = 9.2 Hz), 115.3 (d, *J* = 21.8 Hz), 93.7, 93.3, 37.0, 28.4, 22.1, 22.1. ^{19}F NMR (471 MHz, $CDCl_3$) δ -106.13. HRMS (ESI): m/z calcd. for $C_{14}H_{16}FO^+$($[M+H]^+$) = 219.1180, found = 219.1184.

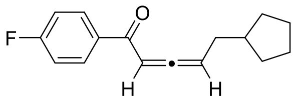

1p

5-cyclopentyl-1-(4-fluorophenyl)penta-2,3-dien-1-one (1p):

(**Method B**: 1:30 ethyl acetate:hexanes as eluent, Rf = 0.3, yellow oil)

^{1}H NMR (400 MHz, $CDCl_3$) δ 7.91 (dd, *J* = 8.8, 5.6 Hz, 1H), 7.10 (t, *J* = 8.8 Hz, 1H), 6.35 – 6.24 (m, 1H), 5.66 – 5.53 (m, 1H), 2.21 – 2.11 (m, 2H), 1.99 – 1.86 (m, 1H), 1.80 – 1.69 (m, 2H), 1.58 – 1.46 (m, 4H), 1.18 – 1.04 (m, 2H). ^{13}C NMR (100 MHz, $CDCl_3$) δ 214.1, 190.4, δ 165.4 (d, *J* = 253.8 Hz), 134.1 (d, *J* = 3.0 Hz), 131.2 (d, *J* = 9.1 Hz), 115.3 (d, *J* = 21.8 Hz), 94.5, 93.5, 39.6, 34.1, 32.2, 25.2, 25.2. ^{19}F NMR (377 MHz, $CDCl_3$) δ -106.2. HRMS (ESI): m/z calcd. for $C_{16}H_{18}FO^+$($[M+H]^+$) = 245.1336, found = 245.1337.

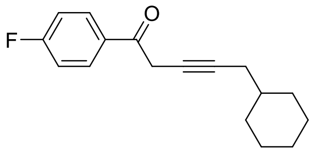

1q

5-cyclohexyl-1-(4-fluorophenyl)pent-3-yn-1-one (1q):

(**Method B**: 1:30 ethyl acetate:hexanes as eluent, Rf = 0.3, yellow oil)

^{1}H NMR (400 MHz, $CDCl_3$) δ 8.04 (dd, *J* = 8.8, 5.6 Hz, 2H), 7.13 (t, *J* = 8.8 Hz, 2H), 3.78 (t, *J* = 2.4 Hz, 2H), 2.12 – 2.01 (m, 2H), 1.78 – 1.60 (m, 4H), 1.47 – 1.26 (m, 4H), 1.27 – 1.03 (m, 1H), 0.99 – 0.85 (m, 2H). ^{13}C NMR (100 MHz, $CDCl_3$) δ 192.6, 165.9 (d, *J* = 255.3 Hz), 131.9 (d, *J* = 3.0 Hz), 131.4 (d, *J* = 9.4 Hz), 115.7 (d, *J* = 21.9 Hz), 85.1, 72.9, 37.3, 32.6, 31.1, 26.6, 26.2, 26.1. ^{19}F NMR (377 MHz, $CDCl_3$) δ -104.6. HRMS (ESI): m/z calcd. for $C_{17}H_{20}FO^+$([M+H]$^+$) = 259.1493, found = 259.1492.

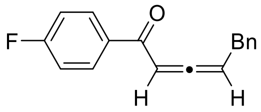

1r

1-(4-fluorophenyl)-5-phenylpenta-2,3-dien-1-one (1r):

(**Method B**: 1:30 ethyl acetate:hexanes as eluent, Rf = 0.3, yellow oil)

^{1}H NMR (400 MHz, $CDCl_3$) δ 7.84 (dd, *J* = 8.8, 5.6 Hz, 2H), 7.34 – 7.27 (m, 2H), 7.26 – 7.21 (m, 1H), 7.17 (d, *J* = 7.2 Hz, 2H), 7.10 – 7.04 (m, 2H), 6.40 – 6.23 (m, 1H), 5.87 – 5.67 (m, 1H), 3.50 (d, *J* = 7.6 Hz, 1H). ^{13}C NMR (100 MHz, $CDCl_3$) δ 214.1, 190.2, 165.4 (d, *J* = 254.1 Hz), 138.3, 133.9 (d, *J* = 2.9 Hz), 131.2 (d, *J* = 9.2 Hz), 128.6, 128.5, 126.7, 115.4 (d, *J* = 21.8 Hz), 94.6, 94.4, 34.2. ^{19}F NMR (377 MHz, $CDCl_3$) δ -106.0. HRMS (ESI): m/z calcd. for $C_{17}H_{14}FO^+$([M+H]$^+$) = 253.1023, found = 253.1024.

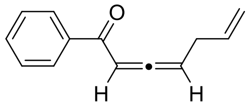

1s

1-phenylhepta-2,3,6-trien-1-one (1s):

(**Method B**: 1:30 ethyl acetate:hexanes as eluent, Rf = 0.3, yellow oil)

^{1}H NMR (400 MHz, $CDCl_3$) δ 7.87 (d, *J* = 7.2 Hz, 2H), 7.54 (t, *J* = 7.6 Hz, 1H), 7.43 (t, *J* = 7.6 Hz, 2H), 5.89 – 5.78 (m, 1H), 5.62 (dd, *J* = 13.2, 7.2 Hz, 1H), 5.15 – 5.10 (m, 1H), 5.09 – 5.07 (m, 1H), 2.98 – 2.86 (m, 2H). ^{13}C NMR (100 MHz, $CDCl_3$) δ 214.0, 191.9, 137.6, 134.6, 132.6, 128.7, 128.3, 116.6, 94.4, 93.1, 31.9. HRMS (ESI): m/z calcd. for $C_{13}H_{13}FO^+$([M+H]$^+$) = 185.0961, found = 185.0963.

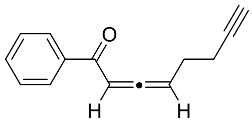

1t

1-phenylocta-2,3-dien-7-yn-1-one (1t):

(**Method B**: 1:30 ethyl acetate:hexanes as eluent, Rf = 0.3, yellow oil)

^{1}H NMR (400 MHz, $CDCl_3$) δ 7.88 (d, *J* = 7.2 Hz, 2H), 7.55 (t, *J* = 7.6 Hz, 1H), 7.44 (t, *J* = 7.6 Hz, 2H), 6.46 – 6.38 (m, 1H), 5.79 – 5.66 (m, 1H), 2.45 – 2.38 (m, 2H), 2.36 – 2.29 (m, 2H), 1.95 (t, *J* = 2.4 Hz, 1H). ^{13}C NMR (100 MHz, $CDCl_3$) δ 213.5, 191.5, 137.6, 132.7, 128.7, 128.3, 94.5, 93.5, 82.8, 69.4, 26.9, 18.2. HRMS (ESI): m/z calcd. for $C_{14}H_{13}FO^+$([M+H]$^+$) = 197.0961, found = 197.0962.

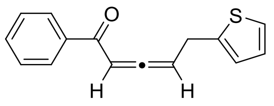

1u

1-phenyl-5-(thiophen-2-yl)penta-2,3-dien-1-one (1u):

(**Method B**: 1:30 ethyl acetate:hexanes as eluent, Rf = 0.3, yellow oil)

^{1}H NMR (400 MHz, $CDCl_3$) δ 7.84 (d, *J* = 7.2 Hz, 2H), 7.54 (t, *J* = 7.6 Hz, 1H), 7.43 (t, *J* = 7.6 Hz, 2H), 7.15 (dd, *J* = 5.2, 1.2 Hz, 1H), 6.94 – 6.90 (m, 1H), 6.85 – 6.81 (m, 1H), 6.45 – 6.37 (m, 1H), 5.85 – 5.76 (m, 1H), 3.73 – 3.64 (m, 2H). ^{13}C NMR (100 MHz, $CDCl_3$) δ 213.9, 191.4, 140.9, 137.54, 132.6, 128.7, 128.3, 126.9, 125.3, 124.1, 94.9, 94.1, 28.5. HRMS (ESI): m/z calcd. for $C_{15}H_{13}FOS^+$([M+H]$^+$) = 241.0682, found = 241.0684

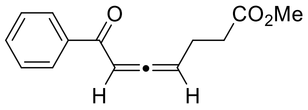

1v

methyl 7-oxo-7-phenylhepta-4,5-dienoate (1v):

(**Method B**: 1:20 to 1:10 ethyl acetate:hexanes as eluent, Rf = 0.3, yellow oil)

^{1}H NMR (400 MHz, $CDCl_3$) δ 7.87 (d, *J* = 8.0 Hz, 2H), 7.55 (t, *J* = 6.8 Hz, 1H), 7.44 (t, *J* = 7.6 Hz, 2H), 6.48 – 6.30 (m, 1H), 5.74 – 5.63 (m, 1H), 3.64 (s, 3H), 2.51 – 2.45 (m, 4H). ^{13}C NMR (100 MHz, $CDCl_3$) δ 213.4, 191.3, 172.7, 137.6, 132.7, 128.6, 128.6, 128.3, 94.7, 93.9, 51.7, 32.9, 22.9. HRMS (ESI): m/z calcd. for $C_{14}H_{15}O_3^+$([M+H]$^+$) = 231.1016, found = 231.1017.

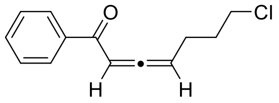

1w

7-chloro-1-phenylhepta-2,3-dien-1-one (1w):

(**Method B**: 1:30 ethyl acetate:hexanes as eluent, Rf = 0.3, yellow oil)

^{1}H NMR (400 MHz, $CDCl_3$) δ 7.90 – 7.83 (m, 2H), 7.55 (t, *J* = 7.2 Hz, 1H), 7.45 (t, *J* = 7.6 Hz, 2H), 6.56 – 6.27 (m, 1H), 5.62 (dd, *J* = 13.2, 7.2 Hz, 1H), 3.54 (t, *J* = 6.4 Hz, 2H), 2.42 – 2.22 (m, 2H), 1.99 – 1.71 (m, 2H). ^{13}C NMR (100 MHz, $CDCl_3$) δ 213.8, 191.5, 137.6, 132.7, 128.6, 128.3, 94.2, 93.7, 43.8, 31.3, 24.8. HRMS (ESI): m/z calcd. for $C_{13}H_{14}ClO^+$([M+H]$^+$) = 221.0728, found = 221.0730.

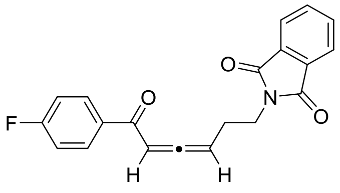

1x

2-(6-(4-fluorophenyl)-6-oxohexa-3,4-dien-1-yl)isoindoline-1,3-dione (1x):

(**Method A**: 1:10 to 1:5 ethyl acetate:hexanes as eluent, Rf = 0.3, yellow oil)

^{1}H NMR (400 MHz, $CDCl_3$) δ 7.86 – 7.77 (m, 4H), 7.76 – 7.68 (m, 2H), 7.06 (t, *J* = 8.8 Hz, 2H), 6.29 – 6.21 (m, 1H), 5.62 (dd, *J* = 13.2, 7.2 Hz, 1H), 3.81 (t, *J* = 7.2 Hz, 2H), 2.68 – 2.52 (m, 2H). ^{13}C NMR (100 MHz, $CDCl_3$) δ 213.7, 189.7, 168.2, 165.4 (d, *J* = 254.5 Hz), 134.0, 133.7 (d, *J* = 3.0 Hz), 131.9, 131.2 (d, *J* = 9.2 Hz), 123.3, 115.4 (d, *J* = 21.9 Hz), 94.1, 91.5, 36.9, 27.0. ^{19}F NMR (471 MHz, $CDCl_3$) δ -104.9. HRMS (ESI): m/z calcd. for $C_{20}H_{15}FNO_3^+$([M+H]$^+$) = 336.1030, found = 336.1031.

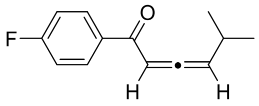

1y

1-(4-fluorophenyl)-5-methylhexa-2,3-dien-1-one (1y):

(**Method B**: 1:30 ethyl acetate:hexanes as eluent, Rf = 0.3, yellow oil)

^{1}H NMR (400 MHz, $CDCl_3$) δ 7.94 – 7.88 (m, 2H), 7.10 (t, *J* = 8.8 Hz, 2H), 6.43 – 6.27 (m, 1H), 5.63 (t, *J* = 6.4 Hz, 1H), 2.57 – 2.44 (m, 1H), 1.08 (d, *J* = 2.8 Hz, 3H), 1.06 (d, *J* = 2.8 Hz, 3H). ^{13}C NMR (100 MHz, $CDCl_3$) δ 212.7, 190.2, 165.4 (d, *J* = 253.8 Hz), 134.0 (d, *J* = 3.1 Hz), 131.2 (d, *J* = 9.1 Hz), 115.3 (d, *J* = 21.8 Hz), 102.2, 95.0, 28.0, 22.3, 22.3. ^{19}F NMR (471 MHz, $CDCl_3$) δ -106.1. HRMS (ESI): m/z calcd. for $C_{13}H_{14}FO^+$([M+H]$^+$) = 205.1023, found = 205.1023.

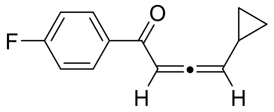

1z

4-cyclopropyl-1-(4-fluorophenyl)buta-2,3-dien-1-one (1z):

(**Method B**: 1:30 ethyl acetate:hexanes as eluent, Rf = 0.3, yellow oil)

^{1}H NMR (400 MHz, $CDCl_3$) δ 7.92 (dd, *J* = 8.8, 5.4 Hz, 2H), 7.11 (t, *J* = 8.8 Hz, 2H), 6.36 (dd, *J* = 6.4, 1.6 Hz, 1H), 5.45 (dd, *J* = 7.6, 6.0 Hz, 1H), 1.46 – 1.33 (m, 1H), 0.86 – 0.77 (m, 2H), 0.51 – 0.36 (m, 2H). ^{13}C NMR (100 MHz, $CDCl_3$) δ 214.3, 190.1, 165.4 (d, *J* = 253.9 Hz), 133.9 (d, *J* = 3.1 Hz), 131.3 (d, *J* = 9.2 Hz), 115.3 (d, *J* = 21.8 Hz), 99.7, 95.3, 8.7, 6.9, 6.8. ^{19}F NMR (471 MHz, $CDCl_3$) δ -105.1. HRMS (ESI): m/z calcd. for $C_{13}H_{12}FO^+$([M+H]$^+$) = 203.0867, found = 203.0866.

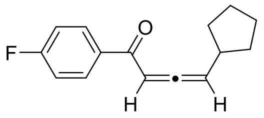

1aa

4-cyclopentyl-1-(4-fluorophenyl)buta-2,3-dien-1-one (1aa):

(**Method B**: 1:30 ethyl acetate:hexanes as eluent, Rf = 0.3, yellow oil)

^{1}H NMR (400 MHz, $CDCl_3$) δ 7.91 (dd, *J* = 8.8, 5.4 Hz, 2H), 7.10 (t, *J* = 8.8 Hz, 2H), 6.40 – 6.23 (m, 1H), 5.65 (dd, *J* = 6.8, 6.0 Hz, 1H), 2.70 – 2.57 (m, 1H), 1.88 – 1.80 (m, 2H), 1.66 – 1.53 (m, 4H), 1.46 – 1.35 (m, 2H). ^{13}C NMR (100 MHz, $CDCl_3$) δ 213.2, 190.4, 165.4 (d, *J* = 253.8 Hz), 134.0 (d, *J* = 3.0 Hz), 131.2 (d, *J* = 9.1 Hz), 115.3 (d, *J* = 21.8 Hz), 100.0, 94.7, 38.5, 32.7, 32.6, 24.8. ^{19}F NMR (471 MHz, None) δ -106.2. HRMS (ESI): m/z calcd. for $C_{15}H_{16}FO^+$([M+H]$^+$) = 231.1180, found = 231.1181.

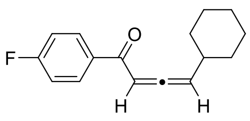

1ab

4-cyclohexyl-1-(4-fluorophenyl)buta-2,3-dien-1-one (1ab):

(**Method B**: 1:30 ethyl acetate:hexanes as eluent, Rf = 0.3, yellow oil)

^{1}H NMR (400 MHz, $CDCl_3$) δ 7.92 (dd, *J* = 8.8, 5.4 Hz, 2H), 7.10 (t, *J* = 8.8 Hz, 2H), 6.36 (dd, *J* = 6.0, 2.8 Hz, 1H), 5.62 – 5.59 (m, 1H), 2.26 – 2.14 (m, 1H), 1.85 – 1.65 (m, 4H), 1.36 – 1.07 (m, 6H). ^{13}C NMR (100 MHz, $CDCl_3$) δ 213.2, 190.2, 165.4 (d, *J* = 253.9 Hz), 134.0 (d, *J* = 3.1 Hz), 131.2 (d, *J* = 9.1 Hz), 115.3 (d, *J* = 21.8 Hz), 100.8, 94.7, 37.0, 32.8, 25.9, 25.7. ^{19}F NMR (377 MHz, $CDCl_3$) δ -106.2. HRMS (ESI): m/z calcd. for $C_{16}H_{18}FO^+$([M+H]$^+$) = 245.1336, found = 245.1336.

8. Reaction Extension

A. Late-stage functionalization of complex natural products

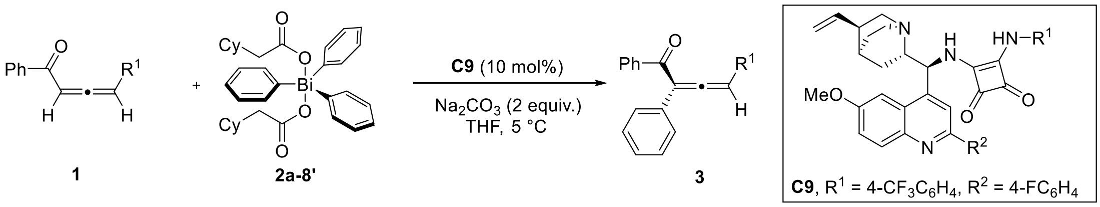

Following GP-A:

To a dried tube was added **2a-8'** (0.1 mmol, 1 equiv.) and Na_2CO_3 (0.2 mmol, 2 equiv.) sequentially, which was followed by adding a solution of **1** (0.15 mmol, 1.5 equiv.) and **C9** (0.01 mmol, 10 mol%) in THF (2 mL). After stirring at 5 °C for 16 h, the mixture was filtered through celite and the filtrate was concentrated *in vacuo*. The residue was then purified by column chromatography on silica gel to give the desired product.

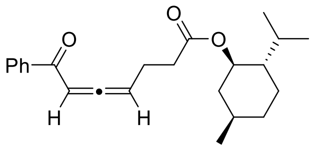

1aq

(1R,2S,5R)-2-isopropyl-5-methylcyclohexyl 7-oxo-7-phenylhepta-4,5-dienoate (1an):

(**Method B**: 1:20 ethyl acetate:hexanes as eluent, Rf = 0.3, yellow oil)

Mixture of diastereoisomers (dr = 1:1). ^{1}H NMR (400 MHz, $CDCl_3$) δ 7.90 – 7.83 (m, 2H), 7.55 (t, *J* = 7.2 Hz, 1H), 7.44 (t, *J* = 8.0 Hz, 2H), 6.45 – 6.37 (m, 1H), 5.74 – 5.62 (m, 1H), 4.72 – 4.65 (m, 1H), 2.54 – 2.40 (m, 4H), 2.00 – 1.91 (m, 1H), 1.87 – 1.79 (m, 1H), 1.75 – 1.63 (m, 2H), 1.51 – 1.43 (m, 1H), 1.41 – 1.31 (m, 2H), 1.09 – 0.93 (m, 2H), 0.90 – 0.86 (m, 6H), 0.74 (d, *J* = 7.2 Hz, 3H). ^{13}C NMR (100 MHz,

$CDCl_3$) δ 213.5, 213.4, 191.3, 191.3, 171.9, 137.6, 132.7, 128.6, 128.3, 94.6, 94.0, 94.0, 74.4, 46.9, 40.9, 34.2, 33.5, 33.4, 31.3, 26.2, 23.3, 23.1, 23.0, 22.0, 20.7, 16.3. HRMS (ESI): m/z calcd. for $C_{23}H_{31}O_3^+$([M+H]$^+$) = 355.2268, found = 355.2269.

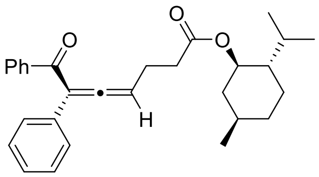

3aq

(1R,2S,5R)-2-isopropyl-5-methylcyclohexyl (R)-7-oxo-6,7-diphenylhepta-4,5-dienoate (3aq):
30 mg, 69% isolated yield, dr > 20:1; $[\alpha]_D^{25}$ = -21.5 (c 1, $CHCl_3$), light yellow oil, 1:20 ethyl acetate:hexanes as eluent, Rf = 0.3. ^{1}H NMR (400 MHz, $CDCl_3$) δ 7.92 (d, *J* = 7.2 Hz, 2H), 7.56 (t, *J* = 7.6 Hz, 1H), 7.44 (t, *J* = 7.2 Hz, 4H), 7.36 (t, *J* = 7.2 Hz, 2H), 7.31 – 7.26 (m, 1H), 5.77 (t, *J* = 6.8 Hz, 1H), 4.77 – 4.60 (m, 1H), 2.53 – 2.28 (m, 4H), 1.97 – 1.89 (m, 1H), 1.88 – 1.77 (m, 1H), 1.68 – 1.61 (m, 2H), 1.53 – 1.29 (m, 3H), 1.13 – 0.95 (m, 2H), 0.92 – 0.84 (m, 6H), 0.73 (d, *J* = 6.8 Hz, 3H). ^{13}C NMR (100 MHz, $CDCl_3$) δ 210.1, 193.7, 171.9, 138.3, 133.2, 132.8, 129.4, 128.5, 128.2, 127.9, 127.7, 109.6, 96.0, 74.5, 46.9, 40.9, 34.2, 33.7, 31.3, 26.2, 23.9, 23.3, 22.0, 20.7, 16.2. HRMS (ESI): m/z calcd. for $C_{29}H_{34}NaO_3^+$([M+Na]$^+$) = 453.2400, found = 453.2402.

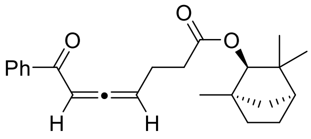

1ar

(1R,2R,4S)-1,3,3-trimethylbicyclo[2.2.1]heptan-2-yl 7-oxo-7-phenylhepta-4,5-dienoate (1ar):
(**Method B**: 1:20 ethyl acetate:hexanes as eluent, Rf = 0.3, yellow oil)
Mixture of diastereoisomers (dr = 1:1). ^{1}H NMR (400 MHz, $CDCl_3$) δ 7.88 (d, *J* = 7.2 Hz, 2H), 7.55 (t, *J* = 7.2 Hz, 1H), 7.45 (t, *J* = 6.8 Hz, 2H), 6.45 – 6.38 (m, 1H), 5.76 – 5.66 (m, 1H), 4.40 – 4.28 (m, 1H), 2.57 – 2.46 (m, 4H), 1.74 – 1.66 (m, 3H), 1.59 – 1.54 (m, 1H), 1.48 – 1.40 (m, 1H), 1.20 – 1.15 (m, 1H), 1.09 (s, 3H), 1.08 – 1.04 (m, 1H), 1.02 (s, 3H), 0.78 – 0.73 (m, 3H). ^{13}C NMR (100 MHz, $CDCl_3$) δ 213.5, 191.4, 172.7, 137.6, 132.7, 128.6, 128.3, 94.6, 94.6, 94.1, 94.0, 86.5, 48.3, 48.2, 41.3, 39.4, 33.3, 29.6, 26.6, 25.6, 23.1, 23.1, 20.1, 19.4, 19.4. HRMS (ESI): m/z calcd. for $C_{23}H_{28}NaO_3^+$([M+Na]$^+$) = 375.1932, found = 375.1931.

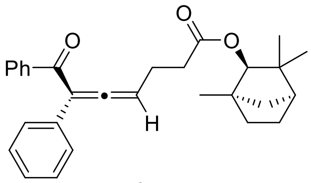

3ar

(1R,2R,4S)-1,3,3-trimethylbicyclo[2.2.1]heptan-2-yl (R)-7-oxo-6,7-diphenylhepta-4,5-dienoate (3ar):

25 mg, 59% isolated yield, dr = 9:1; $[\alpha]_D^{25}$ = 18.1 (c 0.9, $CHCl_3$), light yellow oil, 1:20 ethyl acetate:hexanes as eluent, Rf = 0.3. ^{1}H NMR (400 MHz, $CDCl_3$) δ 7.92 (d, J = 7.2 Hz, 2H), 7.56 (t, J = 7.6 Hz, 1H), 7.50 – 7.41 (m, 4H), 7.36 (t, J = 8.0 Hz, 2H), 7.32 – 7.21 (m, 1H), 5.78 (t, J = 6.8 Hz, 1H), 4.34 (d, J = 2.0 Hz, 1H), 2.55 – 2.38 (m, 4H), 1.73 – 1.65 (m, 3H), 1.59 – 1.53 (m, 1H), 1.50 – 1.38 (m, 1H), 1.21 – 1.15 (m, 1H), 1.11 – 1.05 (m, 4H), 1.01 (s, 3H), 0.73 (s, 3H). ^{13}C NMR (100 MHz, $CDCl_3$) δ 210.0, 193.8, 172.7, 138.3, 133.2, 132.8, 129.4, 128.5, 128.2, 127.9, 127.7, 109.7, 96.0, 86.4, 48.2, 48.2, 41.3, 39.3, 33.5, 29.6, 26.6, 25.8, 24.0, 20.1, 19.4. HRMS (ESI): m/z calcd. for $C_{29}H_{32}NaO_3^+$([M+Na]$^+$) = 451.2244, found = 451.2246.

B. Gram-scale synthesis of α-arylated allene ketones

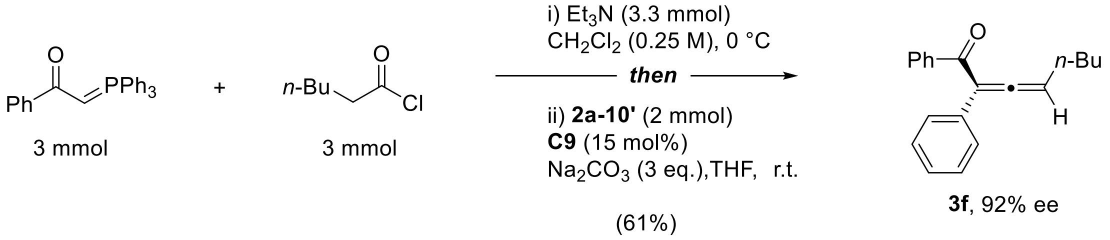

3f, 92% ee

General procedure:

Preparation of $Ph_3Bi(O_2CCH_2Cy)_2$. In a round bottom flask, $BiPh_3$ (2 mmol, 1.0 equiv.) and $PhI(O_2CCH_2Cy)_2$ (0.22 mmol, 1.1 equiv.) were dissolved in DCM (20 mL) and stirred until the full consumption of $BiPh_3$. The resulting solution of **2a-10'** was directly used after evaporation of the solvent.

Gram-scale, synthesis of (*R*)-3f. To an ice-cooled (0 °C) solution of phosphaneylidene)ethan-1-one (3 mmol, 1.5 equiv.) and triethylamine (3.3 mmol, 1.65 equiv.) in DCM (10 mL) was added dropwise a solution of hexanoyl chloride (3 mmol, 1.5 equiv.) in DCM (2 mL). After stirring at 0 °C for 5 h, approximately half of the solvent was removed and diethyl ether was added to precipitate the Ph_3PO. The precipitate was removed by filtration, and the filtrate was washed with 1 N HCl, dried over Na_2SO_4 and concentrated. The crude residue was redissolved in Et_2O and passed through a short pad of silica gel, eluting with Et_2O/hexane (1:2) to afford **1f** (used directly in the next step without further purification).

In a dried reaction tube, **C9** (0.30 mmol, 15 mol%) and Na_2CO_3 (0.20 mmol, 2.0 equiv.) were added sequentially, followed by the addition of a solution of **2a-10′** (2.0 mmol, 1.0 equiv.) and the crude **1f** in THF (60 mL). The mixture was stirred at 5 °C for 48 h. Upon completion, the reaction mixture was concentrated and purified by column chromatography on silica gel (hexane/EtOAc) to afford the desired product **(R)-3f**.

C. Tunable sequential arylation of allene ketones

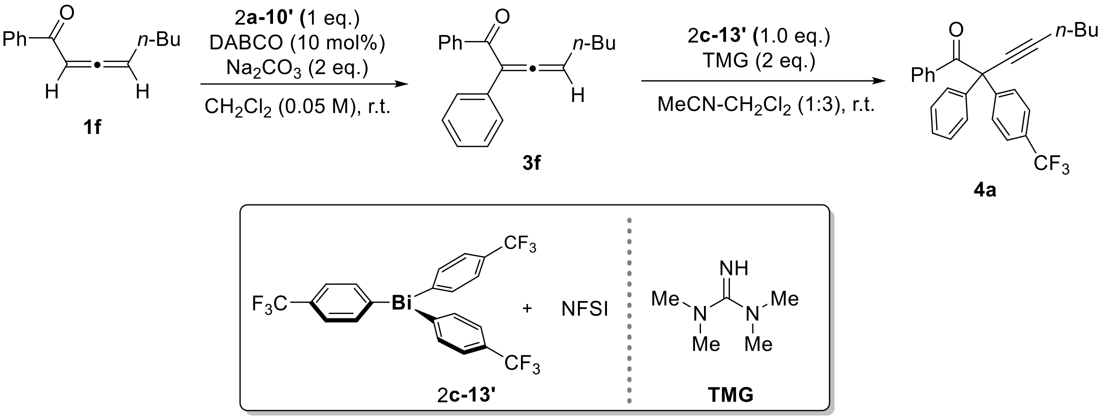

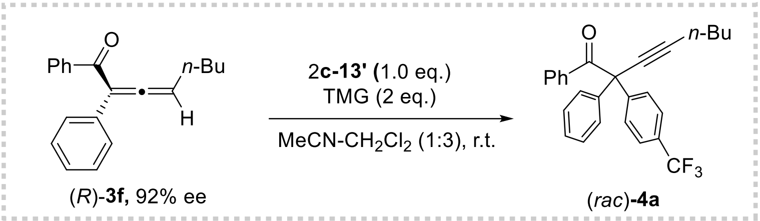

1st aryl group installation. To a dried tube was added **2a-10'** (0.1 mmol, 1 equiv.) and Na_2CO_3 (0.2 mmol, 2 equiv.) sequentially, which was followed by adding a solution of **1f** (0.15 mmol, 1.5 equiv.) and DABCO (0.015 mmol, 15 mol%) in DCM (2 mL). After stirring at room temperature overnight, the mixture was filtered through a pad of silica gel (eluent: Et_2O). The filtrate was concentrated and directly used without further purification.

Preparation of 2c'-13. In a round bottom flask, tris(4-(trifluoromethyl)phenyl)bismuthane (0.10 mmol, 1.0 equiv.) and *N*-Fluorobenzenesulfonimide (0.11 mmol, 1.1 equiv.) was dissolved in MeCN (0.5 mL) and stirred at 80 °C until the full consumption of $BiPh_3$. The resulting solution of **2c-13'** was directly used without further purification.

2nd aryl group installation. To a dried tube was added the solution of **3a** (0.1 mmol, 1 equiv.) in DCM (1.5 mL) and TMG (0.2 mmol, 2 equiv.) sequentially, which was followed by adding the solution of **2c-13'** (0.1 mmol, 1.0 equiv.) in MeCN (0.5 mL). After stirring at room temperature overnight, the mixture was filtered through celite and the filtrate was concentrated *in vacuo*. The residue was then purified by column chromatography on silica gel to give the desired product **4a**. (note: the second arylation step is intrinsically non-enantioselective)

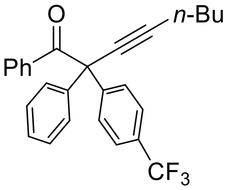

4a

1,2-diphenyl-2-(4-(trifluoromethyl)phenyl)oct-3-yn-1-one (4a):

26 mg, 62% isolated yield, light yellow oil, 1:3 dichoromethane:hexanes as eluent, Rf = 0.3. ^{1}H NMR (500 MHz, $CDCl_3$) δ 7.94 (d, *J* = 8.0 Hz, 2H), 7.57 (d, *J* = 8.5 Hz, 2H), 7.49 – 7.42 (m, 5H), 7.37 (t, *J* = 8.0 Hz, 2H), 7.33 (d, *J* = 8.0 Hz, 1H), 7.31 – 7.27 (m, 2H), 2.29 (t, *J* = 8.0 Hz, 1H), 1.47 – 1.40 (m, 2H), 1.33 – 1.25 (m, 2H), 0.82 (t, *J* = 7.5 Hz, 2H). ^{13}C NMR (125 MHz, $CDCl_3$) δ 195.5, 145.8, 140.2, 134.7,

132.6, 130.96, 129.5 (q, J = 32.5 Hz), 129.2, 128.7, 128.4, 127.8, 127.7, 126.3 (q, J = 270.0 Hz), 124.9 (q, J = 3.8 Hz)., 92.3, 80.8, 61.5, 30.3, 21.9, 18.7, 13.5. ^{19}F NMR (377 MHz, $CDCl_3$) δ -62.5. HRMS (ESI): m/z calcd. for $C_{27}H_{24}F_3O^+$($[M+H]^+$) = 421.1774, found = 421.1777.

9. X-ray Crystallography Data

CCDC 2368781 (**2a-8'**) contains the supplementary crystallographic data for this paper. These data can be obtained free of charge from The Cambridge Crystallographic Date Centre via www.ccdc.cam.ac.uk/data_request/cif.

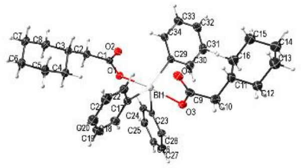

Note: The crystal is triclinic, space group P-1. The asymmetric unit contains one molecule of the compound C34H41O4Bi and half a hexane.

Final R values are R1=0.0294 and wR2=0.0605 for 2- theta up to 61°.

Sample and crystal data

**Identification code**	N345	
**Chemical formula**	$C_{37}H_{48}BiO_4$	
**Formula weight**	765.73 g/mol	
**Temperature**	100(2) K	
**Wavelength**	0.71073 Å	
**Crystal size**	0.170 x 0.205 x 0.207 mm	
**Crystal system**	triclinic	
**Space group**	P -1	
**Unit cell dimensions**	a = 10.5312(7) Å	α = 81.143(2)°
	b = 11.8456(7) Å	β = 69.798(2)°
	c = 14.4812(9) Å	γ = 81.606(2)°
**Volume**	1666.74(18) $Å^3$	
**Z**	2	
**Density (calculated)**	1.526 g/cm^3	
**Absorption coefficient**	5.326 mm^{-1}	
**F(000)**	770	

10. Characterization Data of Products

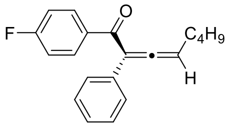

3a

(*R*)-1-(4-fluorophenyl)-2-phenylocta-2,3-dien-1-one (3a):

GP-B: 23.8 mg, 81% isolated yield; $[\alpha]_D^{25}$ = 4.6 (c 0.4, $CHCl_3$), light yellow oil, 1:30 ethyl acetate:hexanes as eluent, Rf = 0.3. ^{1}H NMR (400 MHz, $CDCl_3$) δ 7.95 (dd, *J* = 8.8, 5.4 Hz, 2H), 7.49 – 7.42 (m, 2H), 7.36 (t, *J* = 7.2 Hz, 2H), 7.32 – 7.25 (m, 1H), 7.11 (t, *J* = 8.8 Hz, 2H), 2.24 – 2.12 (m, 2H), 1.45 – 1.37 (m, 2H), 1.32 – 1.24 (m, 2H), 0.86 (t, *J* = 7.6 Hz, 3H). ^{13}C NMR (100 MHz, $CDCl_3$) δ 210.6, 192.5, 165.5 (d, *J* = 254.1 Hz), 134.8 (d, *J* = 3.0 Hz), 133.5, 132.0 (d, *J* = 9.2 Hz), 128.5, 127.9, 127.6, 115.2 (d, *J* = 21.9 Hz), 108.8, 97.5, 31.6, 28.2, 22.1, 13.7. ^{19}F NMR (471 MHz, $CDCl_3$) δ -105.9. HRMS (ESI): m/z calcd. for $C_{20}H_{20}FO^+$([M+H]$^+$) = 295.1493, found = 295.1494; the ee value was 90%, t_R (minor) = 9.0 min, t_R (major) = 9.9 min (Chiralpak IC, λ = 254 nm, 1% *i*-PrOH/Hexane, flow rate = 1.0 mL/min).

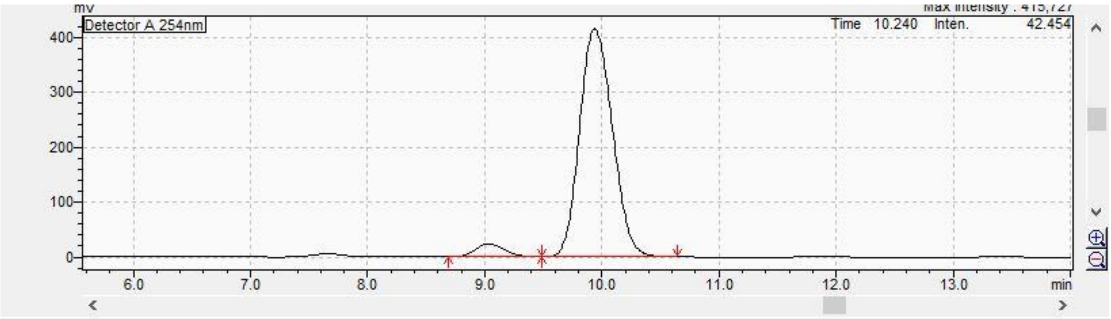

Results View - Peak Table

Peak#	Ret. Time	Area	Height	Mark	Conc.	Unit	Area%
1	9.034	410432	23678	M	4.825		4.825
2	9.939	8095647	415387	M	95.175		95.175
Total		8506079	439066		100.000		100.000

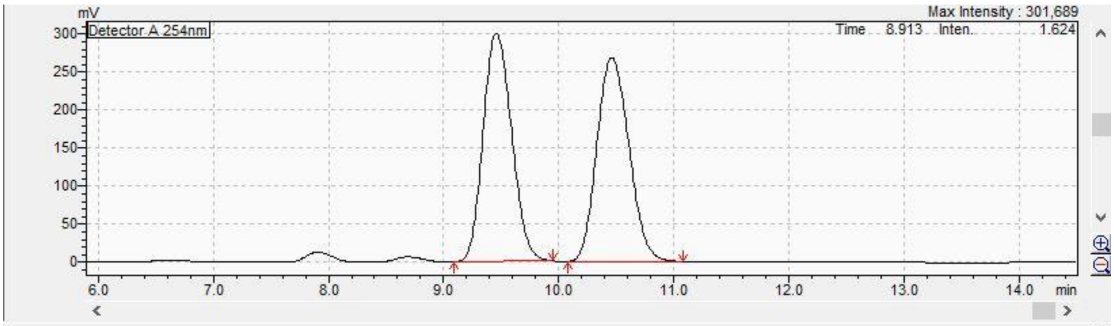

Results View - Peak Table

Peak#	Ret. Time	Area	Height	Mark	Conc.	Unit	Area%
1	9.458	5176260	300253	M	49.842		49.842
2	10.461	5209008	268028	M	50.158		50.158
Total		10385268	568281		100.000		100.000

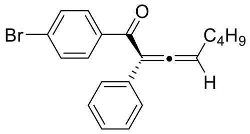

3b

(*R*)-1-(4-bromophenyl)-2-phenylocta-2,3-dien-1-one (3b):

GP-B: 26.9 mg, 76% isolated yield; $[\alpha]_D^{25}$ = 9.4 (c 1.0, $CHCl_3$), light yellow oil, 1:30 ethyl acetate:hexanes as eluent, Rf = 0.3. ^{1}H NMR (400 MHz, $CDCl_3$) δ 7.77 (d, *J* = 8.8 Hz, 2H), 7.57 (d, *J* = 8.4 Hz, 2H), 7.45 (d, *J* = 7.2 Hz, 2H), 7.36 (t, *J* = 7.2 Hz, 2H), 7.32 – 7.21 (m, 1H), 5.71 (t, *J* = 7.2 Hz, 1H), 2.32 – 2.07 (m, 2H), 1.47 – 1.38 (m, 2H), 1.31 – 1.20 (m, 2H), 0.86 (t, *J* = 7.6 Hz, 3H). ^{13}C NMR (100 MHz, $CDCl_3$) δ 211.2, 193.0, 137.4, 133.3, 131.4, 130.9, 128.5, 128.0, 127.6, 127.5, 108.9, 97.6, 31.0, 28.2, 22.1, 13.7. HRMS (ESI): m/z calcd. for $C_{20}H_{20}BrO^+$($[M+H]^+$) = 355.0692, found = 355.0691; the ee value was 86%, t_R (minor) = 10.1 min, t_R (major) = 11.3 min (Chiralpak IG, λ = 254 nm, 1% *i*-PrOH/Hexane, flow rate = 1.0 mL/min).

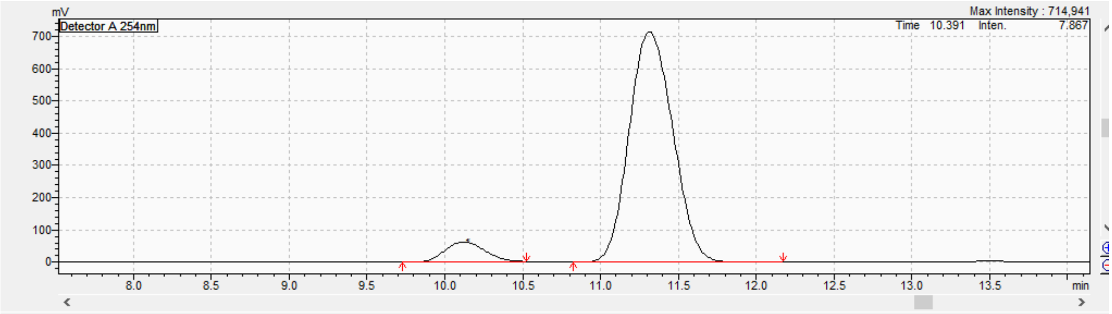

Results View - Peak Table

Peak Table | Compound | Group | Calibration Curve

Peak#	Ret. Time	Area	Height	Mark	Conc.	Unit	ID#	Name	Area%
1	10.119	1087150	61489	M	7.242				7.242
2	11.314	13924839	714340	M	92.758				92.758
Total		15011989	775829		100.000				100.000

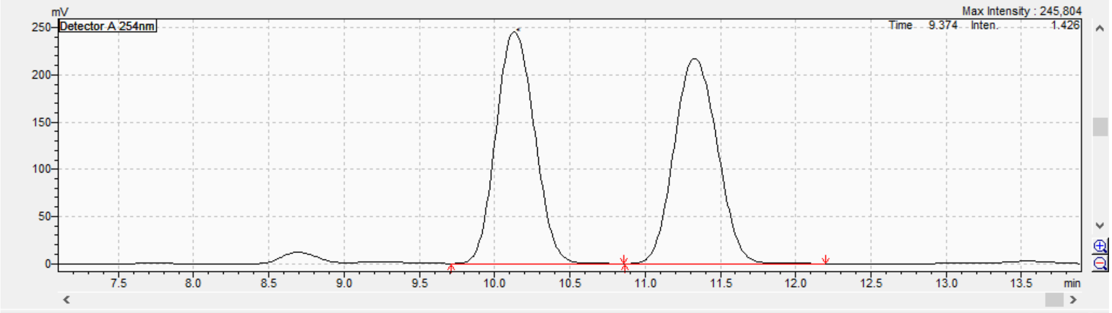

Results View - Peak Table

Peak Table | Compound | Group | Calibration Curve

Peak#	Ret. Time	Area	Height	Mark	Conc.	Unit	ID#	Name	Area%
1	10.130	4395067	246050		50.330				50.330
2	11.329	4337443	217384	M	49.670				49.670
Total		8732509	463434		100.000				100.000

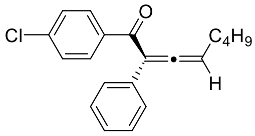

3c

(*R*)-1-(4-chlorophenyl)-2-phenylocta-2,3-dien-1-one (3c):

GP-B: 21.7 mg, 70% isolated yield; $[\alpha]_D^{25}$ = 7.8 (c 0.8, $CHCl_3$), light yellow oil, 1:30 ethyl acetate:hexanes as eluent, Rf = 0.3. ^1H NMR (400 MHz, $CDCl_3$) δ 7.85 (d, *J* = 8.4 Hz, 2H), 7.50 – 7.21 (m, 7H), 5.72 (t, *J* = 7.2 Hz, 1H), 2.30 – 2.03 (m, 2H), 1.48 – 1.33 (m, 2H), 1.32 – 1.19 (m, 2H), 0.86 (t, *J* = 7.2 Hz, 1H). ^{13}C NMR (100 MHz, $CDCl_3$) δ 211.1, 192.8, 138.9, 136.9, 133.3, 130.7, 128.5, 128.4, 128.0, 127.6, 108.9, 97.6, 31.0, 28.2, 22.1, 13.7. HRMS (ESI): m/z calcd. for $C_{20}H_{19}ClNaO^+$($[M+Na]^+$) = 333.1017, found = 333.1017; the ee value was 88%, t_R (minor) = 8.6 min, t_R (major) = 9.4 min (Chiralpak IC, λ = 254 nm, 1% *i*-PrOH/Hexane, flow rate = 1.0 mL/min).

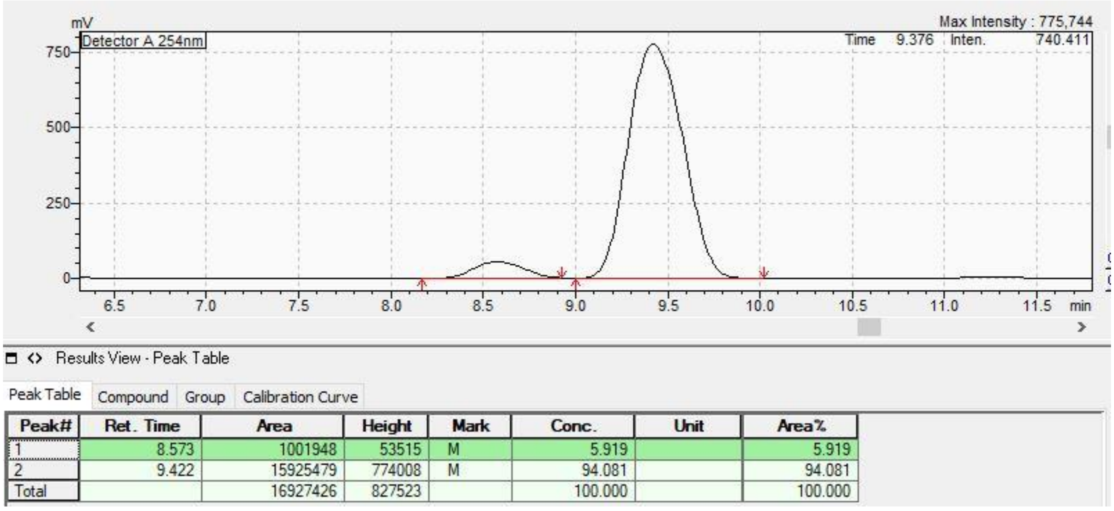

Peak#	Ret. Time	Area	Height	Mark	Conc.	Unit	Area%
1	8.573	1001948	53515	M	5.919		5.919
2	9.422	15925479	774008	M	94.081		94.081
Total		16927426	827523		100.000		100.000

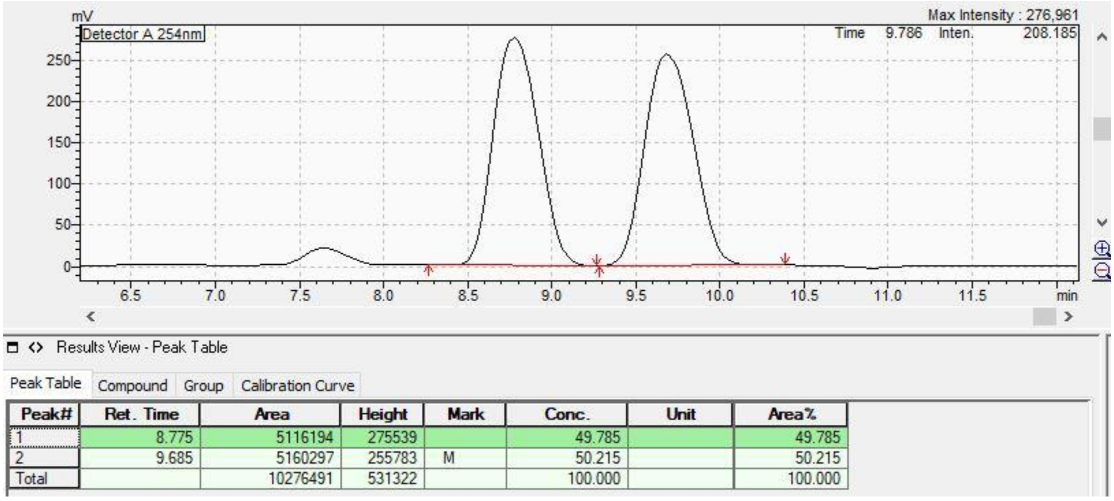

Peak#	Ret. Time	Area	Height	Mark	Conc.	Unit	Area%
1	8.775	5116194	275539		49.785		49.785
2	9.685	5160297	255783	M	50.215		50.215
Total		10276491	531322		100.000		100.000

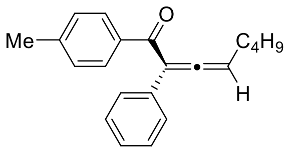

3d

(*R*)-2-phenyl-1-(*p*-tolyl)octa-2,3-dien-1-one (3d):

GP-B: 22.0 mg, 76% isolated yield; $[\alpha]_D^{25}$ = 21.3 (c 0.8, $CHCl_3$), light yellow oil, 1:30 ethyl acetate:hexanes as eluent, Rf = 0.3. ^1H NMR (400 MHz, $CDCl_3$) δ 7.84 (d, *J* = 8.0 Hz, 2H), 7.46 (d, *J* = 7.2 Hz, 2H), 7.35 (t, *J* = 7.6 Hz, 2H), 7.29 – 7.25 (m, 1H), 7.24 (d, *J* = 8.0 Hz, 2H), 5.69 (t, *J* = 7.2 Hz, 1H), 2.42 (s, 3H), 2.27 – 2.11 (m, 2H), 1.47 – 1.37 (m, 2H), 1.33 – 1.23 (m, 2H), 0.86 (t, *J* = 7.2 Hz, 3H). ^{13}C NMR (100 MHz, $CDCl_3$) δ 210.1, 193.7, 143.44, 135.8, 133.9, 129.6, 128.8, 128.4, 127.9, 127.38,

108.8, 97.2, 31.1, 28.20, 22.1, 21.6, 13.8. HRMS (ESI): m/z calcd. for $C_{21}H_{23}O^{+}$([M+H]$^{+}$) = 291.1743, found = 291.1742; the ee value was 93%, t_R (minor) = 19.9 min, t_R (major) = 21.6 min (Chiralpak IC, λ = 254 nm, 1% *i*-PrOH/Hexane, flow rate = 1.0 mL/min).

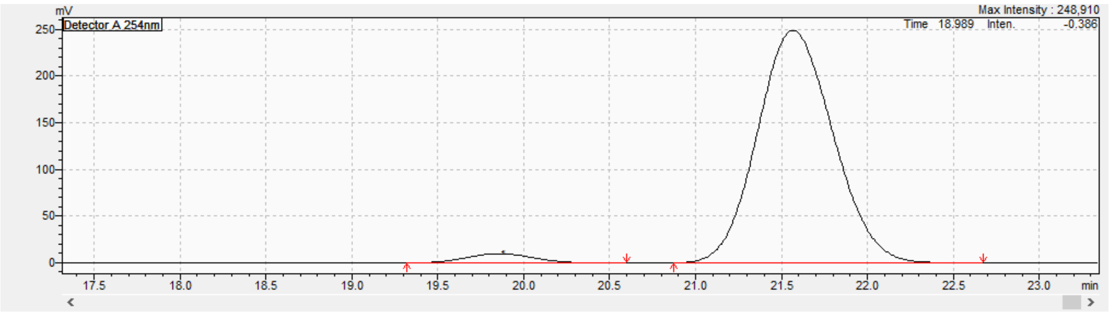

Results View - Peak Table

Peak Table | Compound | Group | Calibration Curve

Peak#	Ret. Time	Area	Height	Mark	Conc.	Unit	ID#	Name	Area%
1	19.857	271673	9941	M	3.438				3.438
2	21.566	7629699	249067	M	96.562				96.562
Total		7901372	259007		100.000				100.000

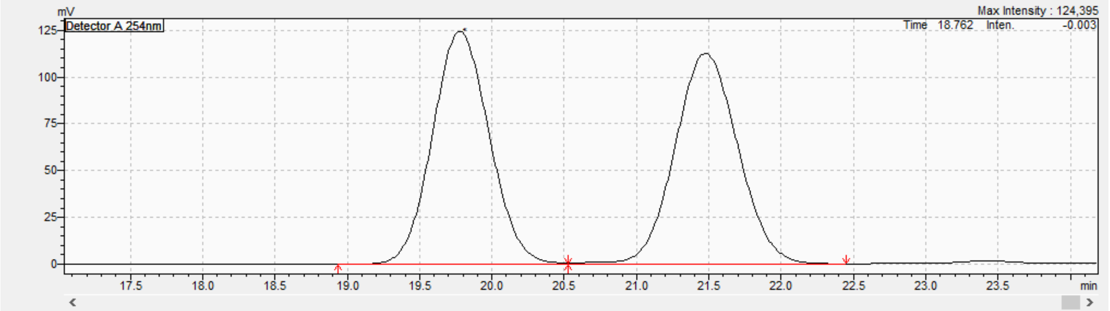

Results View - Peak Table

Peak Table | Compound | Group | Calibration Curve

Peak#	Ret. Time	Area	Height	Mark	Conc.	Unit	ID#	Name	Area%
1	19.778	3402650	124337		49.882				49.882
2	21.476	3418700	112540	V	50.118				50.118
Total		6821350	236877		100.000				100.000

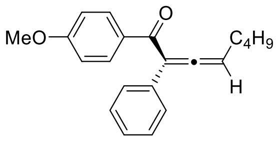

3e

(*R*)-1-(4-methoxyphenyl)-2-phenylocta-2,3-dien-1-one (3e):

GP-B: 18.9 mg, 62% isolated yield; $[\alpha]_D^{25}$ = 3.8 (c 0.6, $CHCl_3$), light yellow oil, 1:30 ethyl acetate:hexanes as eluent, Rf = 0.3. ^{1}H NMR (400 MHz, $CDCl_3$) δ 7.96 (d, *J* = 8.8 Hz, 2H), 7.44 (d, *J* = 7.2 Hz, 2H), 7.34 (t, *J* = 7.1 Hz, 2H), 7.29 – 7.20 (m, 1H), 6.92 (d, *J* = 8.8 Hz, 1H), 5.70 (t, *J* = 7.2 Hz, 1H), 3.87 (s, 3H), 2.36 – 2.08 (m, 2H), 1.51 – 1.38 (m, 2H), 1.36 – 1.24 (m, 2H), 0.86 (t, *J* = 7.2 Hz, 3H). ^{13}C NMR (100 MHz, $CDCl_3$) δ 209.0, 192.5, 163.4, 134.0, 131.9, 131.1, 128.5, 127.7, 127.3, 113.4, 108.6, 97.2, 55.5, 31.2, 28.3, 22.1, 13.8. HRMS (ESI): m/z calcd. for $C_{21}H_{23}O_2^{+}$([M+H]$^{+}$) = 307.1693, found = 307.1691; the ee value was 93%, t_R (minor) = 17.4 min, t_R (major) = 18.1 min (Chiralpak IA, λ = 254 nm, 4% *i*-PrOH/Hexane, flow rate = 1.0 mL/min).

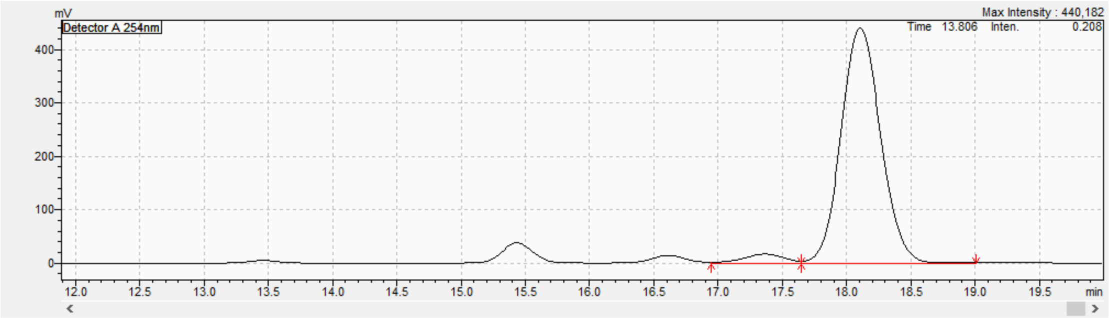

Results View - Peak Table

Peak Table | Compound | Group | Calibration Curve

Peak#	Ret. Time	Area	Height	Mark	Conc.	Unit	ID#	Name	Area%
1	17.364	364734	16714		3.739				3.739
2	18.105	9389668	439971	V	96.261				96.261
Total		9754402	456685		100.000				100.000

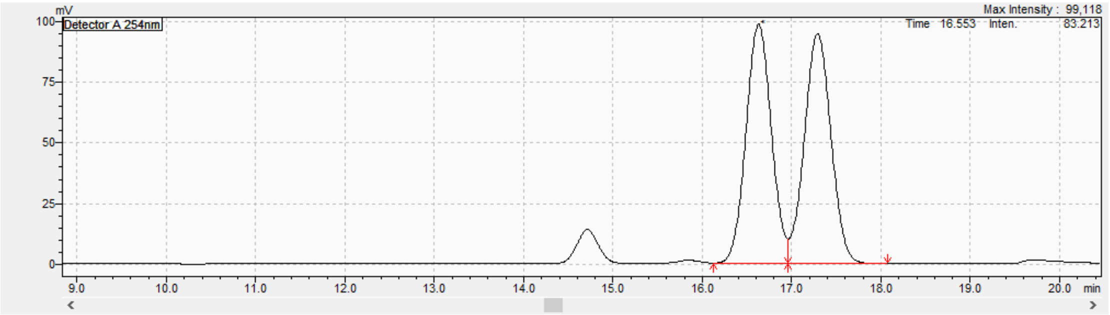

Results View - Peak Table

Peak Table | Compound | Group | Calibration Curve

Peak#	Ret. Time	Area	Height	Mark	Conc.	Unit	ID#	Name	Area%
1	16.630	1958909	98869		49.857				49.857
2	17.291	1970163	94930	V	50.143				50.143
Total		3929072	193798		100.000				100.000

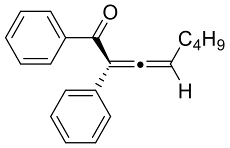

3f

(*R*)-1,2-diphenylocta-2,3-dien-1-one (3f):

GP-B: 22.6 mg, 82% isolated yield; $[\alpha]_D^{25}$ = 24.5 (c 0.9, $CHCl_3$), light yellow oil, 1:30 ethyl acetate:hexanes as eluent, Rf = 0.3. ^{1}H NMR (400 MHz, $CDCl_3$) δ 7.91 (d, *J* = 6.8 Hz, 2H), 7.55 (t, *J* = 7.2 Hz, 2H), 7.49 – 7.40 (m, 4H), 7.36 (t, *J* = 8.0 Hz, 2H), 7.31 – 7.21 (m, 1H), 5.69 (t, *J* = 7.2 Hz, 1H), 2.28 – 2.06 (m, 2H), 1.45 – 1.32 (m, 2H), 1.31 – 1.20 (m, 2H), 0.84 (t, *J* = 7.2 Hz, 3H). ^{13}C NMR (100 MHz, $CDCl_3$) δ 211.0, 194.1, 138.6, 133.7, 132.5, 129.4, 128.4, 128.1, 128.0, 127.5, 108.9, 97.3 31.1, 28.2, 22.1, 13.7. HRMS (ESI): m/z calcd. for $C_{20}H_{21}O^+$($[M+H]^+$) = 277.1587, found = 277.1586; the ee value was 90%, t_R (minor) = 13.7 min, t_R (major) = 15.2 min (Chiralpak IC, λ = 254 nm, 1% *i*-PrOH/Hexane, flow rate = 1.0 mL/min).

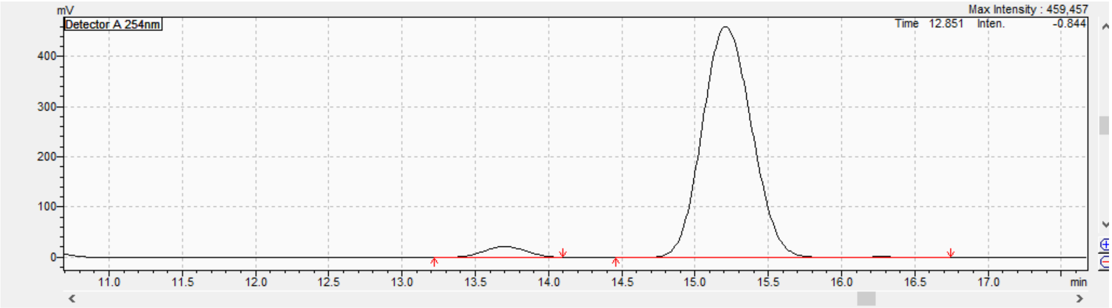

Results View - Peak Table

Peak Table | Compound | Group | Calibration Curve

Peak#	Ret. Time	Area	Height	Mark	Conc.	Unit	ID#	Name	Area%
1	13.702	429261	21249	M	3.813				3.813
2	15.209	10828759	460457	M	96.187				96.187
Total		11258020	481706		100.000				100.000

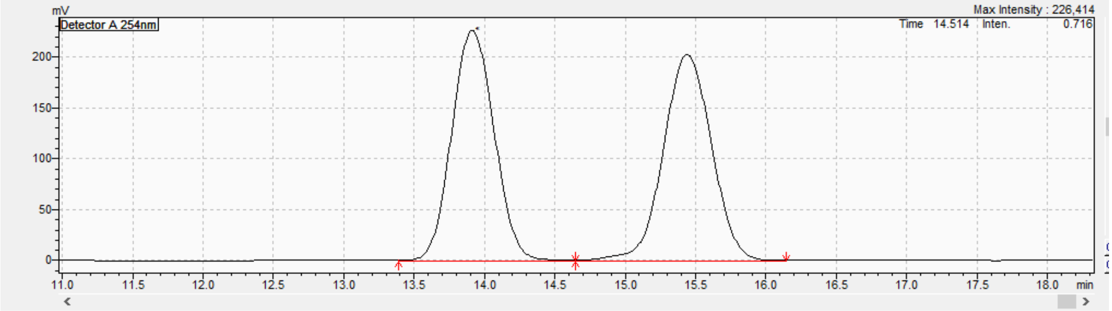

Results View - Peak Table

Peak Table | Compound | Group | Calibration Curve

Peak#	Ret. Time	Area	Height	Mark	Conc.	Unit	ID#	Name	Area%
1	13.913	4740632	226446		49.565				49.565
2	15.440	4823773	202099	V	50.435				50.435
Total		9564405	428546		100.000				100.000

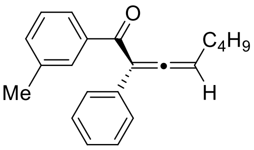

3g

(*R*)-2-phenyl-1-(*m*-tolyl)octa-2,3-dien-1-one (3g):

GP-B: 22.9 mg, 79% isolated yield; $[\alpha]_D^{25}$ = 56.3 (c 0.9, $CHCl_3$), light yellow oil, 1:30 ethyl acetate:hexanes as eluent, Rf = 0.3. ^{1}H NMR (400 MHz, $CDCl_3$) δ 7.85 – 7.65 (m, 2H), 7.47 (d, *J* = 7.2 Hz, 2H), 7.41 – 7.21 (m, 5H), 5.68 (t, *J* = 7.2 Hz, 1H), 2.41 (s, 3H), 2.29 – 2.04 (m, 2H), 1.47 – 1.34 (m, 2H), 1.34 – 1.23 (m, 2H), 0.86 (t, *J* = 7.2 Hz, 3H). ^{13}C NMR (100 MHz, $CDCl_3$) δ 210.8, 194.3, 138.6, 137.8, 133.7, 133.3, 129.9, 128.4, 128.0, 127.9, 127.4, 126.7, 108.9, 97.2, 31.1, 28.2, 22.1, 21.3, 13.8. HRMS (ESI): m/z calcd. for $C_{21}H_{23}O^+$([M+H]$^+$) = 291.1743, found = 291.1745; the ee value was 94%, t_R (minor) = 14.9 min, t_R (major) = 15.9 min (Chiralpak IC, λ = 254 nm, 1% *i*-PrOH/Hexane, flow rate = 1.0 mL/min).

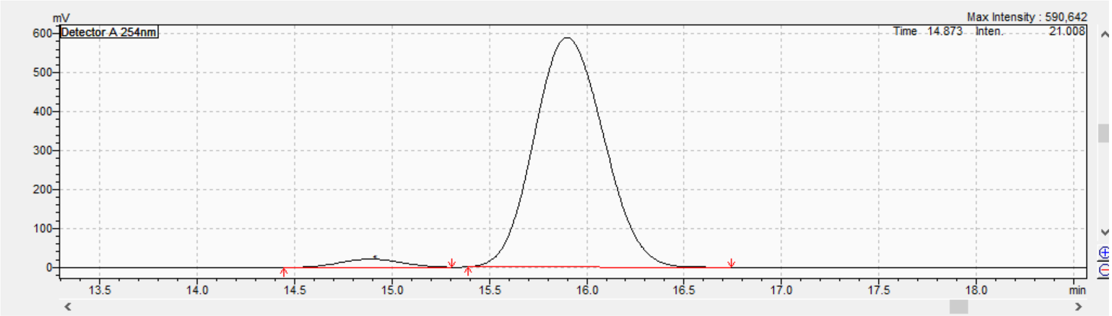

Results View - Peak Table

Peak Table | Compound | Group | Calibration Curve

Peak#	Ret. Time	Area	Height	Mark	Conc.	Unit	ID#	Name	Area%
1	14.891	460148	21041	M	3.044				3.044
2	15.898	14654970	589679	M	96.956				96.956
Total		15115118	610720		100.000				100.000

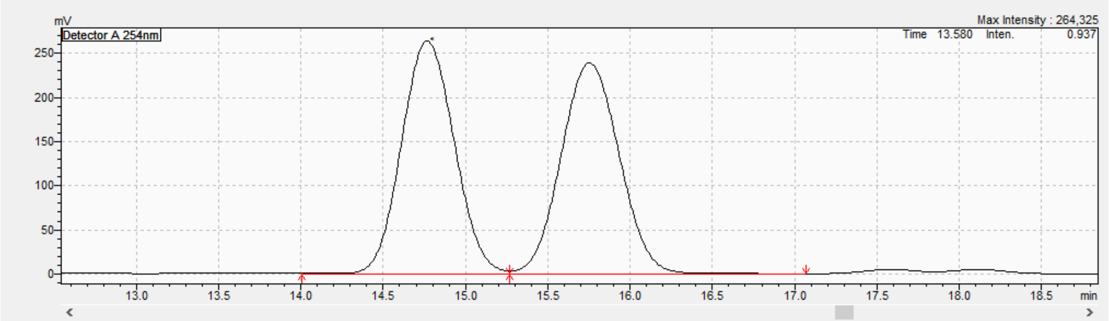

Results View - Peak Table

Peak Table | Compound | Group | Calibration Curve

Peak#	Ret. Time	Area	Height	Mark	Conc.	Unit	ID#	Name	Area%
1	14.766	5873067	263898		50.082				50.082
2	15.753	5853882	238832	V	49.918				49.918
Total		11726949	502730		100.000				100.000

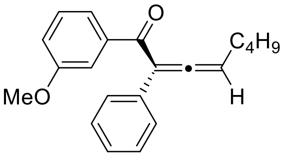

3h

(R)-1-(3-methoxyphenyl)-2-phenylocta-2,3-dien-1-one (3h):

GP-B: 20.2 mg, 66% isolated yield; $[\alpha]_D^{25}$ = 9.1 (c 0.8, $CHCl_3$), light yellow oil, 1:20 ethyl acetate:hexanes as eluent, Rf = 0.3. ^1H NMR (400 MHz, $CDCl_3$) δ 7.52 – 7.44 (m, 4H), 7.39 – 7.27 (m, 4H), 7.09 (ddd, J = 8.0, 2.4, 0.8 Hz, 1H), 5.70 (t, J = 7.2 Hz, 1H), 3.85 (s, 3H), 2.29 – 2.10 (m, 2H), 1.47 – 1.34 (m, 2H), 1.33 – 1.19 (m, 2H), 0.85 (t, J = 7.2 Hz, 3H). ^{13}C NMR (100 MHz, $CDCl_3$) δ 210.7, 193.9, 159.4, 139.9, 133.7, 129.0, 128.5, 127.9, 127.5, 122.2, 119.0, 113.7, 108.9, 97.3, 55.4, 31.1, 28.2, 22.1, 13.7. HRMS (ESI): m/z calcd. for $C_{21}H_{23}O_2^+$($[M+H]^+$) = 307.1693, found = 307.1692; the ee value was 91%, t_R (minor) = 16.8 min, t_R (major) = 14.8 min (Chiralpak IC, λ = 254 nm, 1% *i*-PrOH/Hexane, flow rate = 1.0 mL/min).

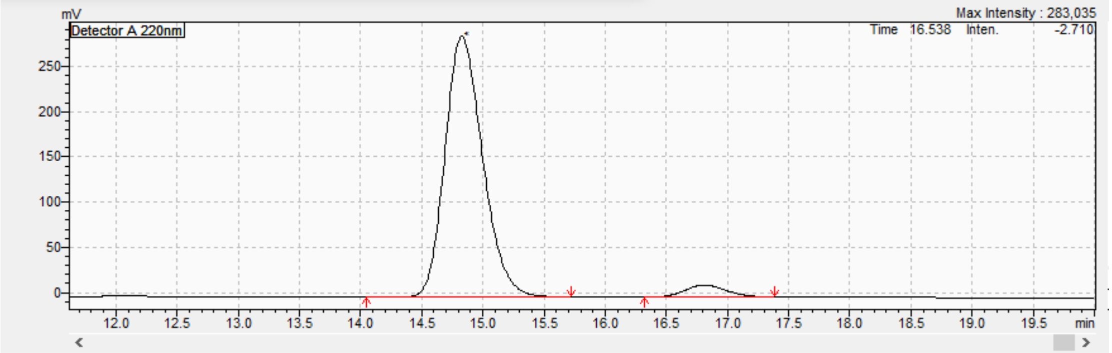

Results View - Peak Table

Peak Table | Compound | Group | Calibration Curve

Peak#	Ret. Time	Area	Height	Mark	Conc.	Unit	ID#	Name	Area%
1	14.828	6177402	287909	M	95.258				95.258
2	16.811	307503	13057	M	4.742				4.742
Total		6484905	300966		100.000				100.000

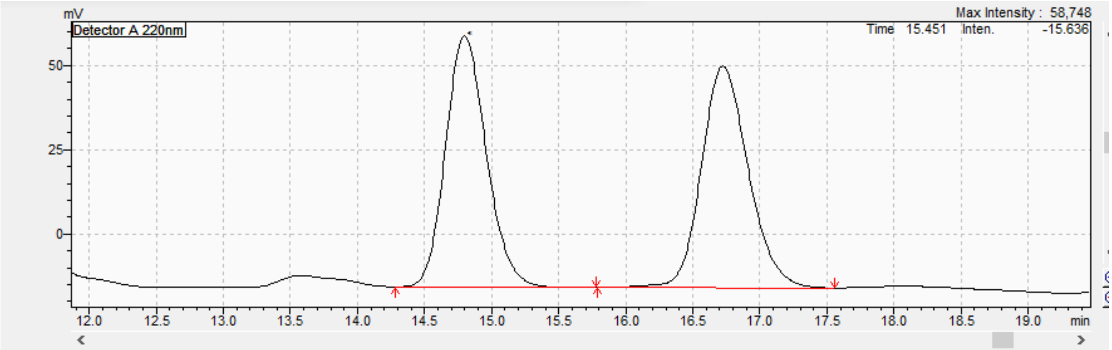

Results View - Peak Table

Peak Table | Compound | Group | Calibration Curve

Peak#	Ret. Time	Area	Height	Mark	Conc.	Unit	ID#	Name	Area%
1	14.798	1586069	74618		49.819				49.819
2	16.724	1597570	65669		50.181				50.181
Total		3183639	140287		100.000				100.000

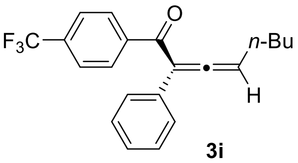

3i

(*R*)-2-phenyl-1-(4-(trifluoromethyl)phenyl)octa-2,3-dien-1-one (3i):

GP-B: 8 mg, 23% isolated yield; rr = 5.6:1, $[\alpha]_D^{25}$ = 3.4 (c 0.4, $CHCl_3$), light yellow oil, 1:20 ethyl acetate:hexanes as eluent, Rf = 0.3. ^1H NMR (600 MHz, $CDCl_3$) δ 7.95 (d, *J* = 8.0 Hz, 2H), 7.69 (d, *J* = 8.2 Hz, 2H), 7.48 (d, *J* = 7.2 Hz, 2H), 7.38 (t, *J* = 7.7 Hz, 2H), 7.31 (t, *J* = 7.4 Hz, 1H), 5.71 (t, *J* = 7.3 Hz, 1H), 2.24 – 2.09 (m, 2H), 1.42 – 1.32 (m, 2H), 1.27 – 1.17 (m, 2H), 0.83 (t, *J* = 7.3 Hz, 3H). ^{13}C NMR (150 MHz, $CDCl_3$) δ 212.6, 193.2, 141.9, 133.6 (q, *J* = 32.4 Hz), 133.0, 129.4, 128.5, 128.2, 127.8, 125.1 (q, *J* = 3.6 Hz), 123.7 (d, *J* = 272.8 Hz). 109.2, 97.8, 31.0, 28.1, 22.0, 13.7. ^{19}F NMR (471 MHz, $CDCl_3$) δ -63.0. HRMS (APCI): m/z calcd. for $C_{21}H_{20}F_3O^+$($[M+H]^+$) = 345.1461, found = 345.1462; the ee value was 73%, t_R (minor) = 6.5 min, t_R (major) = 7.1 min (Chiralpak IG, λ = 254 nm, 1% *i*-PrOH/Hexane, flow rate = 1.0 mL/min).

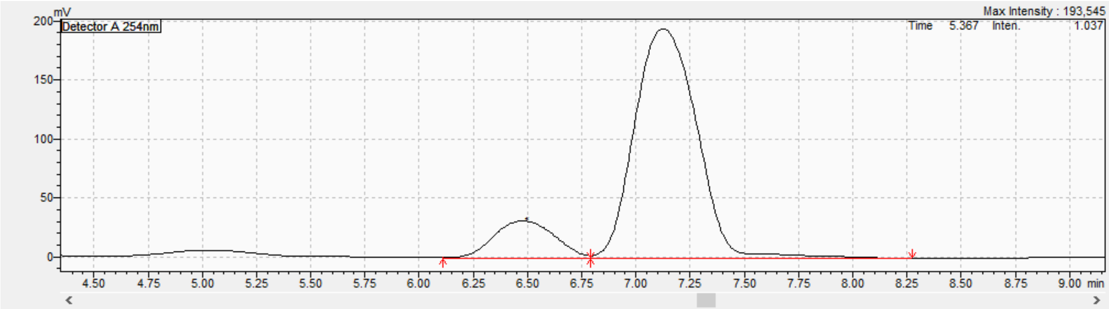

Results View - Peak Table

Peak Table | Compound | Group | Calibration Curve

Peak#	Ret. Time	Area	Height	Mark	Conc.	Unit	ID#	Name	Area%
1	6.477	611797	31413		13.673				13.673
2	7.125	3862561	194631	V	86.327				86.327
Total		4474358	226044		100.000				100.000

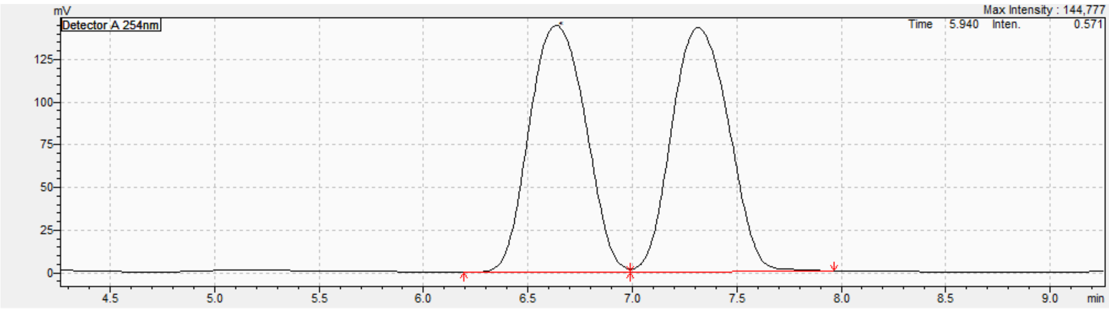

Results View - Peak Table

Peak Table | Compound | Group | Calibration Curve

Peak#	Ret. Time	Area	Height	Mark	Conc.	Unit	ID#	Name	Area%
1	6.635	2703849	144266	M	49.851				49.851
2	7.315	2720007	142678	V M	50.149				50.149
Total		5423856	286945		100.000				100.000

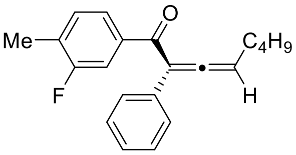

3j

(*R*)-1-(3-fluoro-4-methylphenyl)-2-phenylocta-2,3-dien-1-one (3j):

GP-B: 23.1 mg, 75% isolated yield; $[\alpha]_D^{25}$ = 19.7 (c 0.9, $CHCl_3$), light yellow oil, 1:30 ethyl acetate:hexanes as eluent, Rf = 0.3. ^{1}H NMR (400 MHz, $CDCl_3$) δ 7.81 (d, *J* = 7.6 Hz, 1H), 7.79 – 7.72 (m, 1H), 7.45 (d, *J* = 7.2 Hz, 2H), 7.36 (t, *J* = 7.6 Hz, 2H), 7.32 – 7.24 (m, 1H), 7.04 (t, *J* = 8.8 Hz, 1H), 5.70 (t, *J* = 7.2 Hz, 1H), 2.32 (d, *J* = 2.0 Hz, 3H), 2.27 – 2.10 (m, 2H), 1.47 – 1.37 (m, 2H), 1.33 – 1.23 (m, 2H), 0.86 (t, *J* = 7.2 Hz, 3H). ^{13}C NMR (100 MHz, $CDCl_3$) δ 210.29, 192.76, 164.13 (d, *J* = 252.9 Hz), 134.46 (d, *J* = 3.3 Hz), 133.6, 133.1 (d, *J* = 6.5 Hz), 129.4 (d, *J* = 9.2 Hz), 128.5, 127.9, 127.5, 124.9 (d, *J* = 17.9 Hz), 114.7 (d, *J* = 23.0 Hz), 108.8, 97.3, 31.1, 28.2, 22.1, 14.5 (d, *J* = 3.0 Hz), 13.7. ^{19}F NMR (471 MHz, $CDCl_3$) δ -110.0. HRMS (ESI): m/z calcd. for $C_{21}H_{22}FO^+$($[M+H]^+$) = 309.1649, found = 309.1650; the ee value was 90%, t_R (minor) = 10.3 min, t_R (major) = 11.1 min (Chiralpak IC, λ = 254 nm, 1% *i*-PrOH/Hexane, flow rate = 1.0 mL/min).

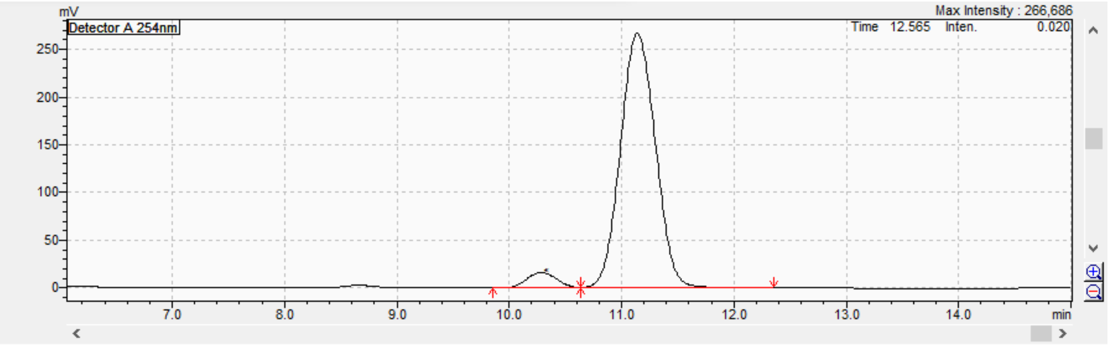

Results View - Peak Table

Peak Table | Compound | Group | Calibration Curve

Peak#	Ret. Time	Area	Height	Mark	Conc.	Unit	ID#	Name	Area%
1	10.283	304356	16183		4.997				4.997
2	11.140	5786159	266676	V	95.003				95.003
Total		6090515	282859		100.000				100.000

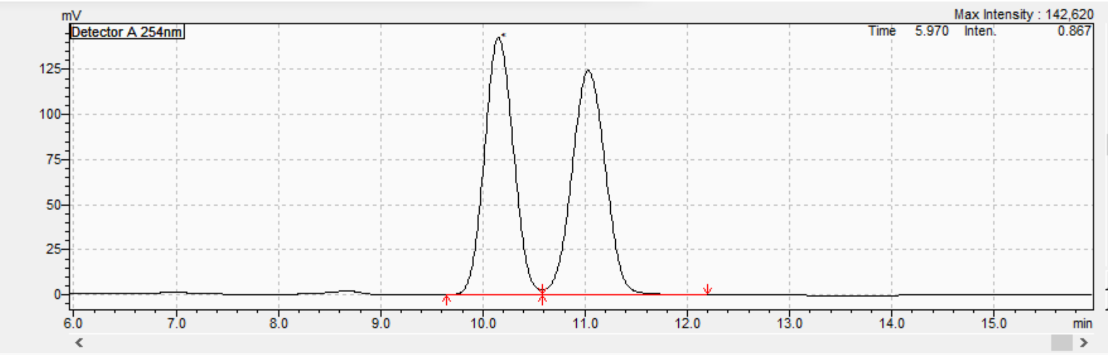

Results View - Peak Table

Peak Table | Compound | Group | Calibration Curve

Peak#	Ret. Time	Area	Height	Mark	Conc.	Unit	ID#	Name	Area%
1	10.152	2804244	142596	V	49.780				49.780
2	11.032	2829056	124370	V	50.220				50.220
Total		5633301	266966		100.000				100.000

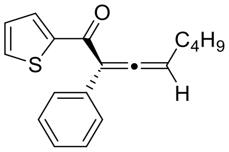

3k

(*R*)-2-phenyl-1-(thiophen-2-yl)octa-2,3-dien-1-one (3k):

GP-B: 18.9 mg, 67% isolated yield; $[\alpha]_D^{25}$ = 3.6 (c 0.5, $CHCl_3$), light yellow oil, 1:3 dichloromethane:hexanes as eluent, Rf = 0.3. ^{1}H NMR (400 MHz, $CDCl_3$) δ 7.84 (dd, *J* = 4.0, 1.2 Hz, 1H), 7.65 (dd, *J* = 4.8, 0.8 Hz, 1H), 7.46 (d, *J* = 7.2 Hz, 2H), 7.35 (t, *J* = 7.2 Hz, 2H), 7.31 – 7.24 (m, 1H), 7.11 (dd, *J* = 4.9, 3.9 Hz, 1H), 5.85 (t, *J* = 7.2 Hz, 1H), 2.44 – 2.16 (m, 2H), 1.55 – 1.44 (m, 2H), 1.42 – 1.32 (m, 2H), 0.89 (t, *J* = 7.2 Hz, 3H). ^{13}C NMR (100 MHz, $CDCl_3$) δ 209.5, 184.8, 144.0, 133.8, 133.7, 133.4, 128.4, 127.9, 127.7, 127.5, 109.1, 98.1, 31.0, 28.5, 22.2, 13.8. HRMS (ESI): m/z calcd. for $C_{18}H_{19}OS^+([M+H]^+)$ = 283.1151, found = 283.1151; the ee value was 90%, t_R (minor) = 19.3 min, t_R (major) = 17.9 min (Chiralpak IE, λ = 254 nm, 1% *i*-PrOH/Hexane, flow rate = 1.0 mL/min).

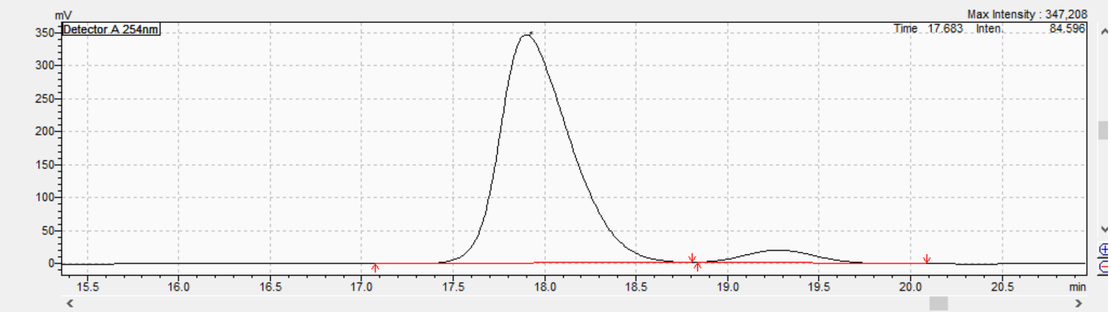

Results View - Peak Table

Peak Table | Compound | Group | Calibration Curve

Peak#	Ret. Time	Area	Height	Mark	Conc.	Unit	ID#	Name	Area%
1	17.901	9228436	345863	M	94.837				94.837
2	19.276	502435	18961	M	5.163				5.163
Total		9730871	364824		100.000				100.000

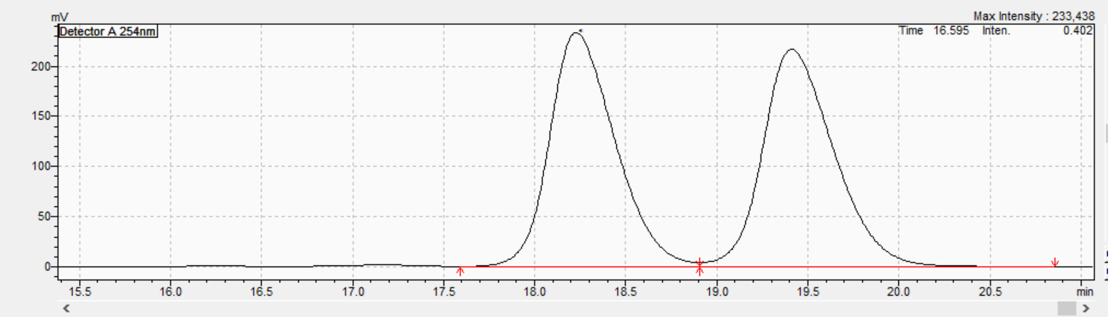

Results View - Peak Table

Peak Table | Compound | Group | Calibration Curve

Peak#	Ret. Time	Area	Height	Mark	Conc.	Unit	ID#	Name	Area%
1	18.228	5862156	233327		49.900				49.900
2	19.411	5885626	216558	V	50.100				50.100
Total		11747782	449885		100.000				100.000

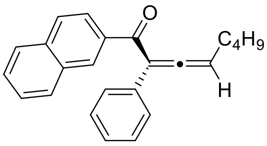

3l

(*R*)-1-(naphthalen-2-yl)-2-phenylocta-2,3-dien-1-one (3l):

GP-B: 23.1 mg, 71% isolated yield; $[\alpha]_D^{25}$ = 3.2 (c 0.3, $CHCl_3$), light yellow oil, 1:30 ethyl acetate:hexanes as eluent, Rf = 0.3. ^1H NMR (400 MHz, $CDCl_3$) δ 8.47 (s, 1H), 8.00 (dd, *J* = 8.4, 1.6 Hz, 1H), 7.93 (d, *J* = 8.0 Hz, 1H), 7.89 (d, *J* = 8.8 Hz, 2H), 7.64 – 7.51 (m, 4H), 7.41 – 7.35 (m, 2H), 7.34 – 7.28 (m, 1H), 5.72 (t, *J* = 7.2 Hz, 1H), 2.33 – 2.12 (m, 2H), 1.47 – 1.32 (m, 2H), 1.29 – 1.16 (m, 2H), 0.80 (t, *J* = 7.2 Hz, 3H). ^{13}C NMR (100 MHz, $CDCl_3$) δ 210.7, 194.0, 135.9, 135.5, 133.8, 132.3, 131.1, 129.4, 128.7, 128.5, 128.3, 128.2, 128.0, 127.8, 127.5, 126.6, 125.2, 109.1, 97.4, 31.1, 28.2, 22.1, 13.7. HRMS (ESI): m/z calcd. for $C_{24}H_{23}O^+$([M+H]$^+$) = 327.1743, found = 327.1744; the ee value was 92%, t_R (minor) = 12.7 min, t_R (major) = 13.9 min (Chiralpak IE, λ = 254 nm, 2% *i*-PrOH/Hexane, flow rate = 1.0 mL/min).

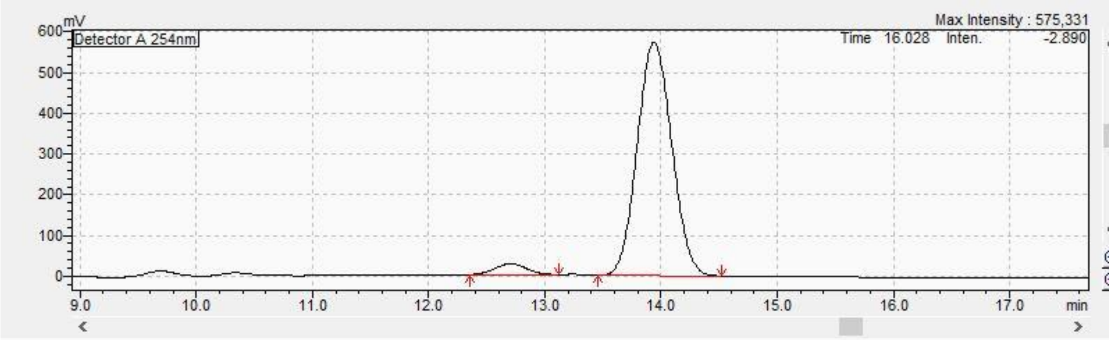

Results View - Peak Table

Peak Table | Compound | Group | Calibration Curve

Peak#	Ret. Time	Area	Height	Mark	Conc.	Unit	Area%
1	12.704	496738	26419	M	4.028		4.028
2	13.940	11836643	573749	M	95.972		95.972
Total		12333382	600168		100.000		100.000

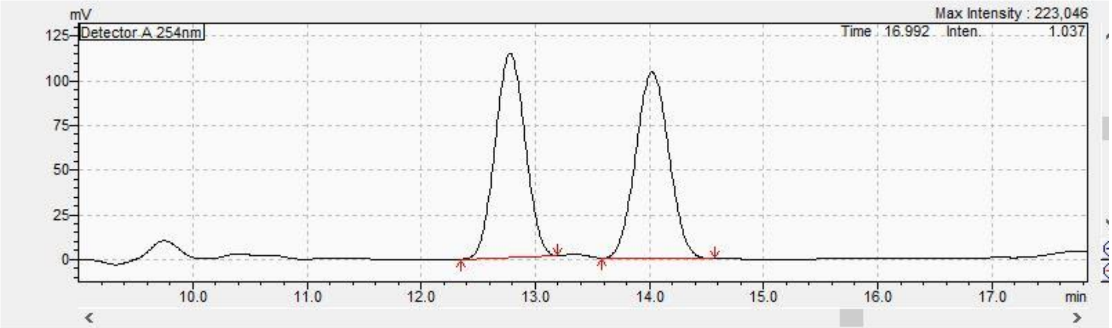

Results View - Peak Table

Peak Table | Compound | Group | Calibration Curve

Peak#	Ret. Time	Area	Height	Mark	Conc.	Unit	Area%
1	12.779	2086269	114412		49.596		49.596
2	14.024	2120245	104386		50.404		50.404
Total		4206515	218798		100.000		100.000

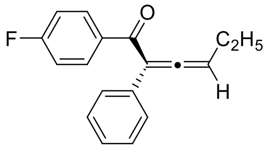

3m

(*R*)-1-(4-fluorophenyl)-2-phenylhexa-2,3-dien-1-one (3m):

GP-B: 20.7 mg, 78% isolated yield; $[\alpha]_D^{25}$ = 29.5 (c 0.8, $CHCl_3$), light yellow oil, 1:30 ethyl acetate:hexanes as eluent, Rf = 0.3. ^{1}H NMR (400 MHz, $CDCl_3$) δ 7.95 (dd, *J* = 8.8, 5.2 Hz, 2H), 7.45 (d, *J* = 7.2 Hz, 2H), 7.36 (t, *J* = 7.2 Hz, 2H), 7.32 – 7.23 (m, 1H), 7.11 (t, *J* = 8.8 Hz, 2H), 5.77 (t, *J* = 6.8 Hz, 1H), 2.25 – 2.15 (m, 2H), 1.06 (t, *J* = 7.2 Hz, 3H). ^{13}C NMR (100 MHz, $CDCl_3$) δ 210.2, 192.5, 165.5 (d, *J* = 254.1 Hz), 134.7, 134.7 (d, *J* = 3.0 Hz), 132.0 (d, *J* = 9.2 Hz), 128.5, 127.9, 127.6, 115.2 (d, *J* = 21.8 Hz), 109.4, 99.1, 21.9, 13.4. ^{19}F NMR (471 MHz, $CDCl_3$) δ -105.8. HRMS (ESI): m/z calcd. for $C_{18}H_{16}FO^+$($[M+H]^+$) = 267.1180, found = 267.1181; the ee value was 90%, t_R (minor) = 10.9 min, t_R (major) = 12.8 min (Chiralpak IC, λ = 254 nm, 1% *i*-PrOH/Hexane, flow rate = 1.0 mL/min).

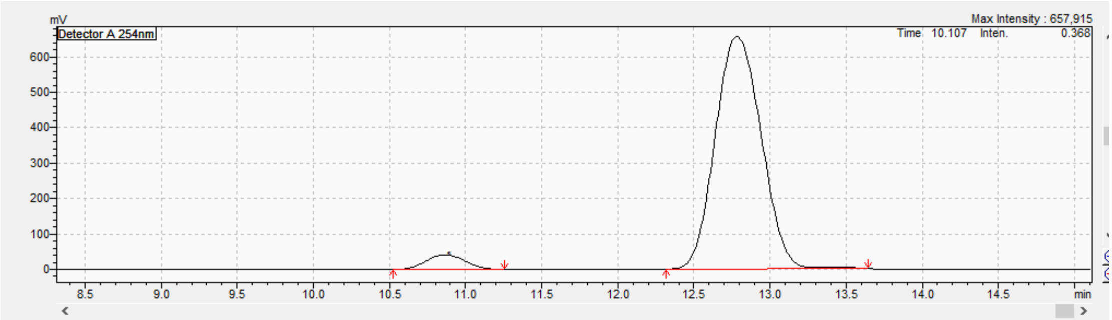

Results View - Peak Table

Peak Table | Compound | Group | Calibration Curve

Peak#	Ret. Time	Area	Height	Mark	Conc.	Unit	ID#	Name	Area%
1	10.864	723924	40835	M	4.984				4.984
2	12.783	13801227	656507	M	95.016				95.016
Total		14525151	697342		100.000				100.000

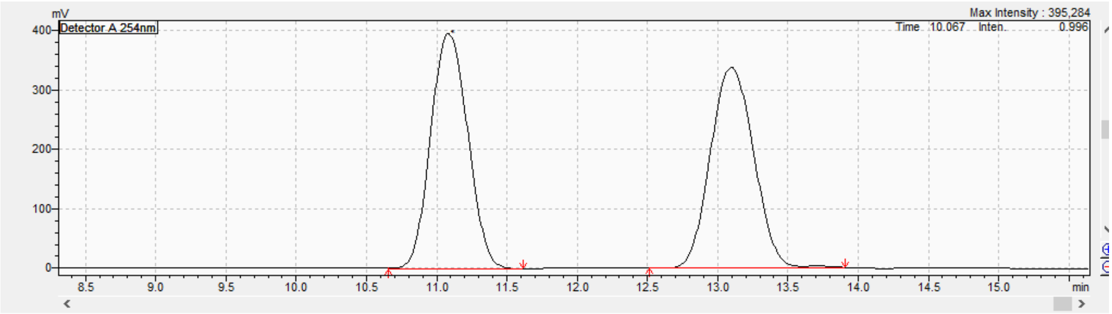

Results View - Peak Table

Peak Table | Compound | Group | Calibration Curve

Peak#	Ret. Time	Area	Height	Mark	Conc.	Unit	ID#	Name	Area%
1	11.083	7291690	396199		50.062				50.062
2	13.097	7273535	337654	M	49.938				49.938
Total		14565226	733853		100.000				100.000

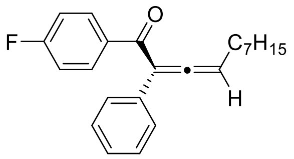

3n

(*R*)-1-(4-fluorophenyl)-2-phenylundeca-2,3-dien-1-one (3n):

GP-B: 27.2 mg, 81% isolated yield; $[\alpha]_D^{25}$ = 2.8 (c 0.5, $CHCl_3$), light yellow oil, 1:30 ethyl acetate:hexanes as eluent, Rf = 0.3. ^{1}H NMR (400 MHz, $CDCl_3$) δ 7.95 (dd, *J* = 9.2, 5.6 Hz, 2H), 7.45 (d, *J* = 7.2 Hz, 2H), 7.36 (t, *J* = 7.2 Hz, 2H), 7.32 – 7.23 (m, 1H), 7.11 (t, *J* = 8.8 Hz, 2H), 5.71 (t, *J* = 7.2 Hz, 1H), 2.30 – 2.08 (m, 2H), 1.46 – 1.37 (m, 2H), 1.30 – 1.16 (m, 8H), 0.87 (t, *J* = 6.8 Hz, 3H). ^{13}C NMR (100 MHz, $CDCl_3$) δ 210.7, 192.5, 165.5 (d, *J* = 254.0 Hz), 134.8 (d, *J* = 3.0 Hz), 133.5, 132.0 (d, *J* = 9.2 Hz), 128.5, 127.9, 127.6, 115.2 (d, *J* = 21.9 Hz), 108.8, 97.50, 31.7, 29.0, 28.5, 22.6, 14.0. ^{19}F NMR (471 MHz, $CDCl_3$) δ -105.9. HRMS (ESI): m/z calcd. for $C_{23}H_{26}FO^+$($[M+H]^+$) = 337.1962, found = 337.1964; the ee value was 90%, t_R (minor) = 14.8 min, t_R (major) = 11.5 min (Chiralcel® OJ-H, λ = 254 nm, 1% *i*-PrOH/Hexane, flow rate = 1.0 mL/min).

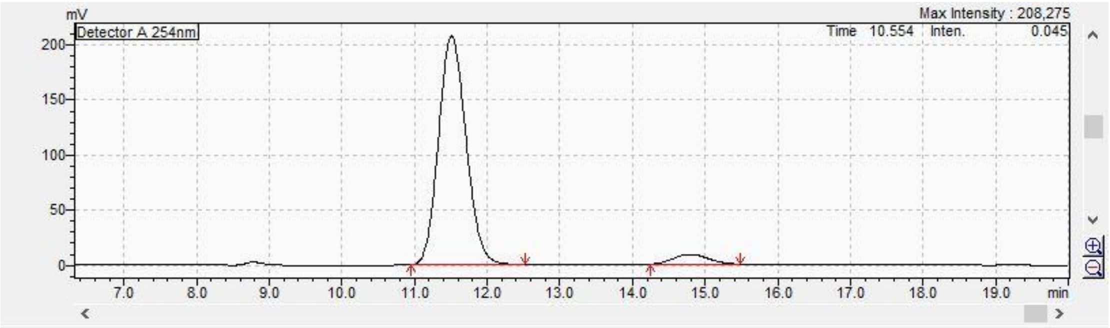

Results View - Peak Table

Peak Table | Compound | Group | Calibration Curve

Peak#	Ret. Time	Area	Height	Mark	Conc.	Unit	Area%
1	11.509	5475374	207780	M	94.838		94.838
2	14.793	298010	8949	M	5.162		5.162
Total		5773384	216730		100.000		100.000

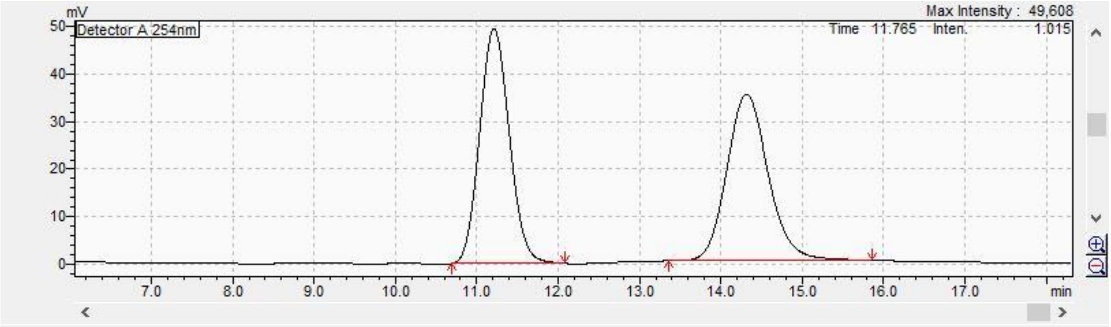

Results View - Peak Table

Peak Table | Compound | Group | Calibration Curve

Peak#	Ret. Time	Area	Height	Mark	Conc.	Unit	Area%
1	11.212	1248321	49511	M	50.106		50.106
2	14.313	1243044	35095	M	49.894		49.894
Total		2491366	84606		100.000		100.000

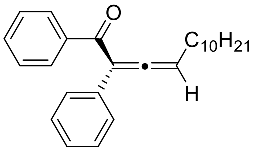

3o

(*R*)-1,2-diphenyltetradeca-2,3-dien-1-one (3o):

GP-B: 25.9 mg, 72% isolated yield; $[\alpha]_D^{25}$ = 3.6 (c 0.6, $CHCl_3$), light yellow oil, 1:30 ethyl acetate:hexanes as eluent, Rf = 0.3. ^{1}H NMR (400 MHz, $CDCl_3$) δ 7.91 (d, *J* = 6.8 Hz, 2H), 7.54 (t, *J* = 7.6 Hz, 1H), 7.49 – 7.41 (m, 4H), 7.36 (t, *J* = 7.6 Hz, 2H), 7.30 – 7.25 (m, 1H), 5.69 (t, *J* = 7.2 Hz, 1H), 2.27 – 2.03 (m, 2H), 1.44 – 1.35 (m, 2H), 1.32 – 1.16 (m, 14H), 0.89 (t, *J* = 6.9 Hz, 3H). ^{13}C NMR (100 MHz, $CDCl_3$) δ 211.1, 194.1, 138.6, 133.7, 132.5, 129.4, 128.4, 128.1, 128.0, 127.5, 108.9, 97.3, 31.9, 29.5, 29.5, 29.3, 29.0, 29.0, 28.5, 22.7, 14.1. HRMS (ESI): m/z calcd. for $C_{26}H_{33}O^+$([M+H]$^+$) = 361.2526, found = 361.2527; the ee value was 91%, t_R (minor) = 8.6 min, t_R (major) = 6.8 min (Chiralcel® OJ-H, λ = 254 nm, 2% *i*-PrOH/Hexane, flow rate = 1.0 mL/min).

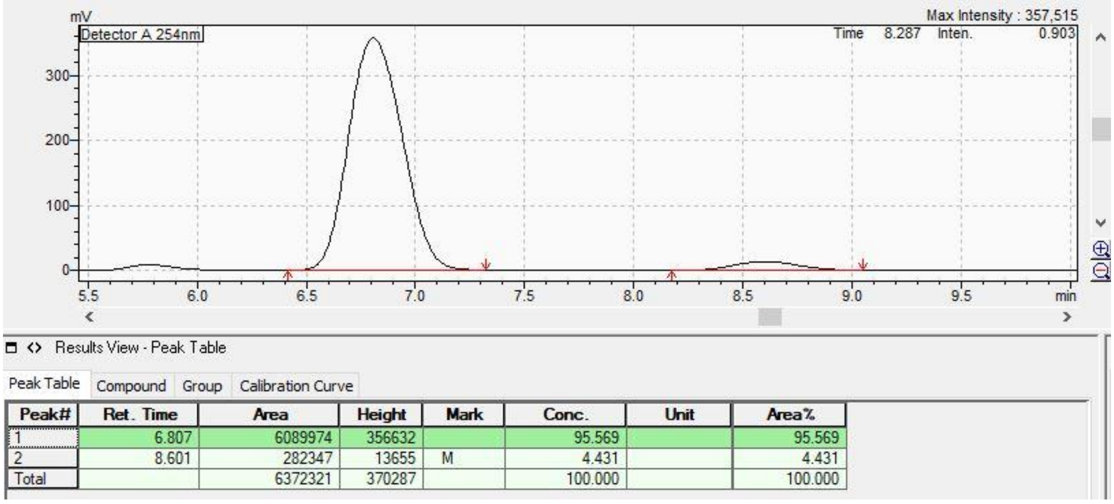

Peak#	Ret. Time	Area	Height	Mark	Conc.	Unit	Area%
1	6.807	6089974	356632		95.569		95.569
2	8.601	282347	13655	M	4.431		4.431
Total		6372321	370287		100.000		100.000

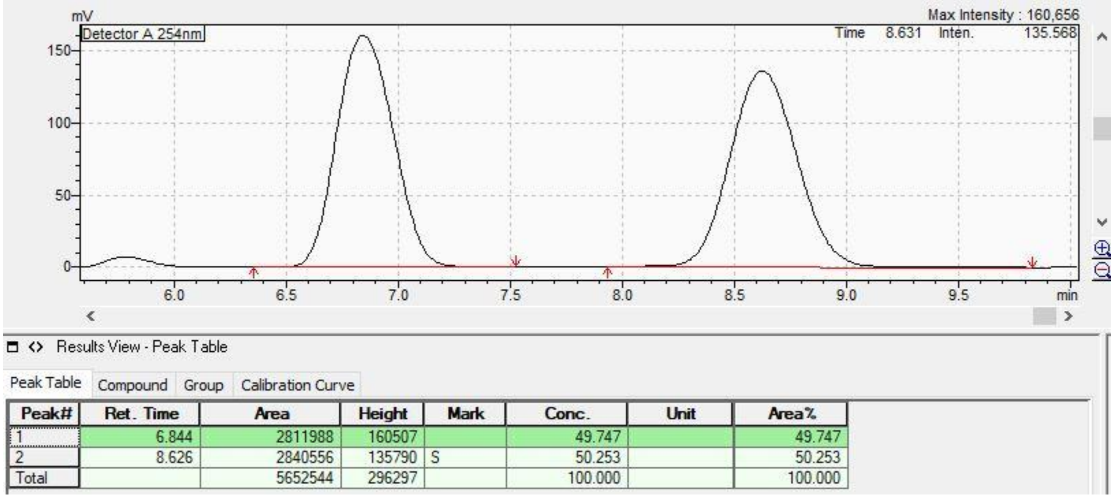

Peak#	Ret. Time	Area	Height	Mark	Conc.	Unit	Area%
1	6.844	2811988	160507		49.747		49.747
2	8.626	2840556	135790	S	50.253		50.253
Total		5652544	296297		100.000		100.000

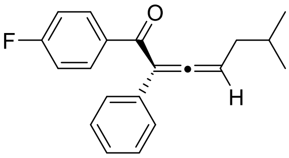

3p

(*R*)-1-(4-fluorophenyl)-6-methyl-2-phenylhepta-2,3-dien-1-one (3p):

GP-B: 25.0 mg, 85% isolated yield; $[\alpha]_D^{25}$ = 37.5 (c 1, $CHCl_3$), light yellow oil, 1:30 ethyl acetate:hexanes as eluent, Rf = 0.3. ^{1}H NMR (400 MHz, $CDCl_3$) δ 7.94 (dd, *J* = 8.8, 6.4 Hz, 2H), 7.45 (d, *J* = 7.2 Hz, 2H), 7.36 (t, *J* = 7.2 Hz, 2H), 7.31 – 7.25 (m, 1H), 7.12 (d, *J* = 8.8 Hz, 2H), 5.68 (t, *J* = 7.6 Hz, 1H), 2.18 – 1.99 (m, 2H), 1.78 – 1.61 (m, 1H), 0.87 (d, *J* = 6.8 Hz, 3H), 0.83 (d, *J* = 6.8 Hz, 3H). ^{13}C NMR (100 MHz, $CDCl_3$) δ 211.0, 192.6, 165.5 (d, *J* = 254.0 Hz), 134.8 (d, *J* = 3.0 Hz), 133.4, 131.9 (d, *J* = 9.2 Hz), 128.5, 128.0, 127.6, 115.2 (d, *J* = 21.9 Hz), 108.3, 96.1, 37.6, 28.5, 22.1, 22.1. ^{19}F NMR (471 MHz, $CDCl_3$) δ -105.9. HRMS (ESI): m/z calcd. for $C_{20}H_{20}FO^+$($[M+H]^+$) = 295.1493, found = 295.1494; the ee value was 88%, t_R (minor) = 25.4 min, t_R (major) = 18.8 min (Chiralcel® OJ-H, λ = 254 nm, 1% *i*-PrOH/Hexane, flow rate = 1.0 mL/min).

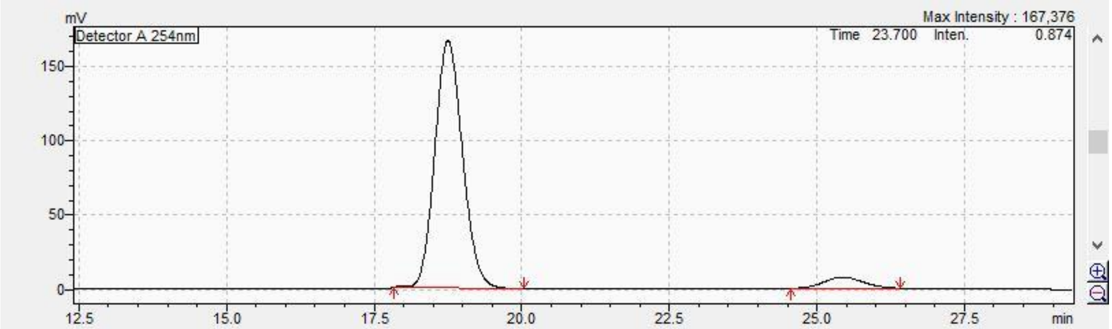

Results View - Peak Table

Peak Table | Compound | Group | Calibration Curve

Peak#	Ret. Time	Area	Height	Mark	Conc.	Unit	Area%
1	18.750	5424102	166264	M	93.986		93.986
2	25.436	347078	7481	M	6.014		6.014
Total		5771180	173745		100.000		100.000

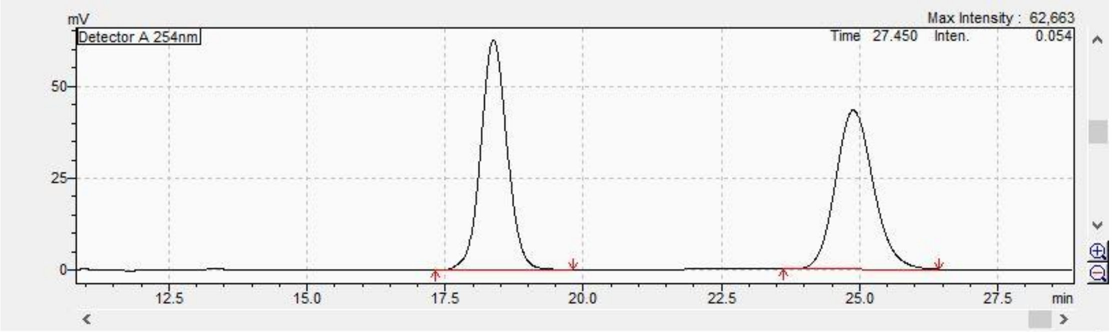

Results View - Peak Table

Peak Table | Compound | Group | Calibration Curve

Peak#	Ret. Time	Area	Height	Mark	Conc.	Unit	Area%
1	18.375	2079801	62559	M	50.391		50.391
2	24.889	2047488	43119	M	49.609		49.609
Total		4127289	105679		100.000		100.000

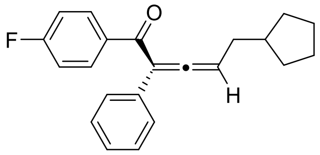

3q

(*R*)-5-cyclopentyl-1-(4-fluorophenyl)-2-phenylpenta-2,3-dien-1-one (3q):

GP-B: 25.6 mg, 80% isolated yield; $[\alpha]_D^{25}$ = 20.8 (c 1, $CHCl_3$), light yellow oil, 1:30 ethyl acetate:hexanes as eluent, Rf = 0.3. ^1H NMR (400 MHz, $CDCl_3$) δ 7.94 (dd, *J* = 8.8, 5.6 Hz, 2H), 7.45 (d, *J* = 7.6 Hz, 2H), 7.36 (t, *J* = 7.2 Hz, 2H), 7.30 – 7.24 (m, 1H), 7.11 (t, *J* = 8.8 Hz, 2H), 5.71 (t, *J* = 7.6 Hz, 1H), 2.39 – 2.10 (m, 2H), 1.96 – 1.80 (m, 1H), 1.76 – 1.63 (m, 2H), 1.62 – 1.47 (m, 4H), 1.16 – 0.98 (m, 2H). ^{13}C NMR (100 MHz, $CDCl_3$) δ 210.9, 192.5, 165.5 (d, *J* = 254.0 Hz), 134.8 (d, *J* = 3.0 Hz), 133.5, 132.0 (d, *J* = 9.2 Hz), 128.5, 127.9, 127.6, 115.2 (d, *J* = 21.8 Hz), 108.5, 96.9, 39.7, 34.8, 32.3, 32.2, 25.2. ^{19}F NMR (377 MHz, $CDCl_3$) δ -105.9. HRMS (ESI): m/z calcd. for $C_{22}H_{21}FNaO^+$($[M+Na]^+$) = 343.1469, found = 343.1470; the ee value was 90%, t_R (minor) = 10.4 min, t_R (major) = 11.3 min (Chiralpak IC, λ = 254 nm, 1% *i*-PrOH/Hexane, flow rate = 1.0 mL/min).

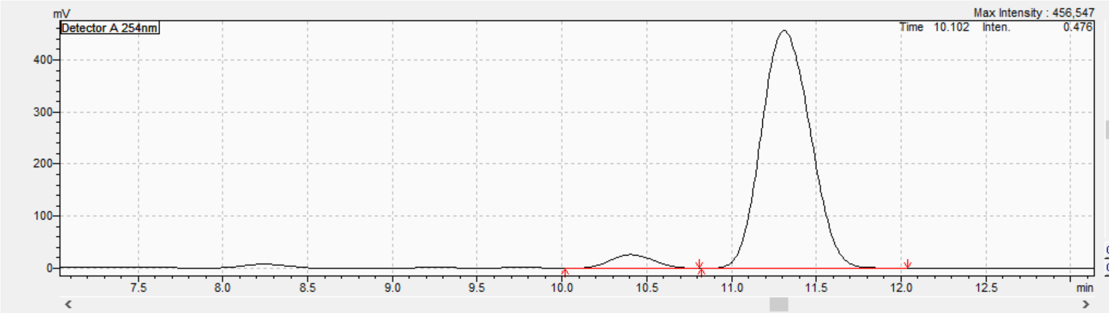

Results View - Peak Table

Peak Table | Compound | Group | Calibration Curve

Peak#	Ret. Time	Area	Height	Mark	Conc.	Unit	ID#	Name	Area%
1	10.404	455214	25864	M	4.739				4.739
2	11.310	9150950	456436	M	95.261				95.261
Total		9606164	482300		100.000				100.000

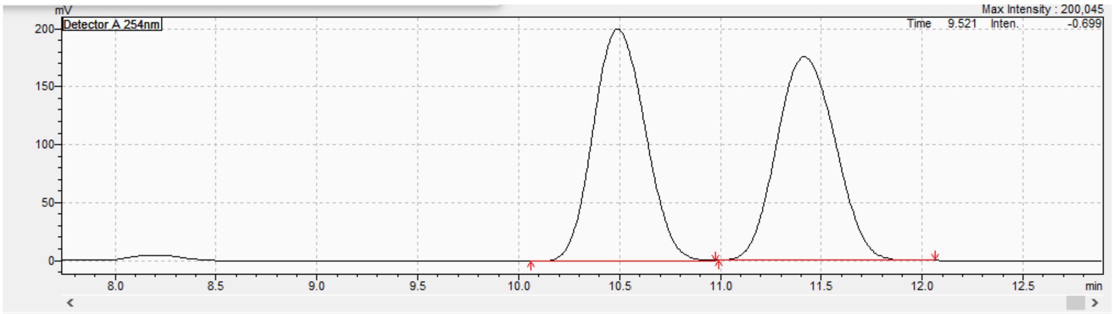

Results View - Peak Table

Peak Table | Compound | Group | Calibration Curve

Peak#	Ret. Time	Area	Height	Mark	Conc.	Unit	ID#	Name	Area%
1	10.491	3597940	200758		50.265				50.265
2	11.415	3560020	175732	M	49.735				49.735
Total		7157960	376490		100.000				100.000

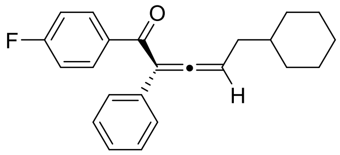

3r

(*R*)-5-cyclohexyl-1-(4-fluorophenyl)-2-phenylpenta-2,3-dien-1-one (3r):

GP-B: 24.4 mg, 73% isolated yield; $[\alpha]_D^{25}$ = 22.5 (c 1, $CHCl_3$), light yellow oil, 1:30 ethyl acetate:hexanes as eluent, Rf = 0.3. ^{1}H NMR (400 MHz, $CDCl_3$) δ 7.93 (dd, *J* = 8.8, 5.6 Hz, 2H), 7.45 (d, *J* = 6.8 Hz, 2H), 7.36 (t, *J* = 7.2 Hz, 2H), 7.30 – 7.23 (m, 1H), 7.11 (t, *J* = 8.8 Hz, 2H), 5.68 (t, *J* = 8.0 Hz, 1H), 2.17 – 2.00 (m, 2H), 1.67 – 1.58 (m, 4H), 1.55 – 1.52 (m, 1H), 1.37 – 1.27 (m, 1H), 1.21 – 1.04 (m, 3H), 0.93 – 0.76 (m, 2H). ^{13}C NMR (100 MHz, $CDCl_3$) δ 211.2, 192.6, 165.4 (d, *J* = 253.9 Hz), 134.9 (d, *J* = 3.0 Hz), 133.5, 131.9 (d, *J* = 9.1 Hz), 128.5, 128.0, 127.6, 115.2 (d, *J* = 21.9 Hz), 108.4, 96.0, 38.0, 36.2, 32.9, 32.8, 26.3, 26.1. ^{19}F NMR (471 MHz, $CDCl_3$) δ -106.1. HRMS (ESI): m/z calcd. for $C_{23}H_{24}FO^+$($[M+H]^+$) = 335.1806, found = 335.1807; the ee value was 90%, t_R (minor) = 27.3 min, t_R (major) = 22.0 min (Chiralpak IG, λ = 254 nm, 1% *i*-PrOH/Hexane, flow rate = 1.0 mL/min).

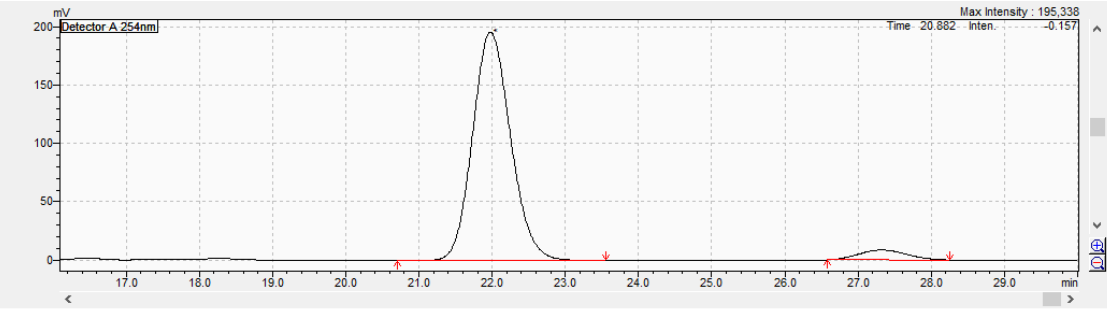

Results View - Peak Table

Peak Table | Compound | Group | Calibration Curve

Peak#	Ret. Time	Area	Height	Mark	Conc.	Unit	ID#	Name	Area%
1	21.978	7037041	195439	M	94.789				94.789
2	27.324	386848	8470	M	5.211				5.211
Total		7423890	203909		100.000				100.000

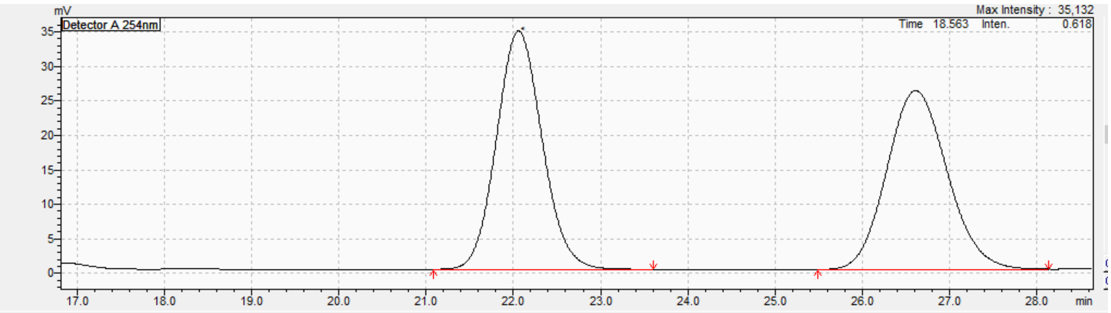

Results View - Peak Table

Peak Table | Compound | Group | Calibration Curve

Peak#	Ret. Time	Area	Height	Mark	Conc.	Unit	ID#	Name	Area%
1	22.061	1260993	34549		50.212				50.212
2	26.612	1250325	25925		49.788				49.788
Total		2511318	60474		100.000				100.000

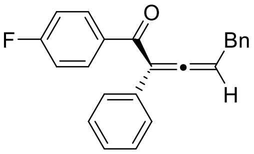

3s

(*R*)-1-(4-fluorophenyl)-2,5-diphenylpenta-2,3-dien-1-one (3s):

GP-B: 25.2 mg, 77% isolated yield; $[\alpha]_D^{25}$ = 44 (c 1, $CHCl_3$), light yellow oil, 1:30 ethyl acetate:hexanes as eluent, Rf = 0.3. ^{1}H NMR (400 MHz, $CDCl_3$) δ 7.75 (dd, *J* = 8.8, 5.6 Hz, 2H), 7.35 – 7.26 (m, 4H), 7.23 – 7.12 (m, 4H), 7.01 – 6.94 (m, 4H), 5.83 (t, *J* = 7.2 Hz, 1H), 3.54 – 3.32 (m, 2H). ^{13}C NMR (100 MHz, $CDCl_3$) δ 210.8, 192.2, 165.5 (d, *J* = 254.2 Hz), 138.4, 134.6 (d, *J* = 3.0 Hz), 133.1, 132.0 (d, *J* = 9.2 Hz), 128.5, 128.5, 127.9, 127.8, 126.7, 115.3 (d, *J* = 21.9 Hz) 109.6, 96.9, 34.7. ^{19}F NMR (471 MHz, $CDCl_3$) δ -105.7. HRMS (ESI): m/z calcd. for $C_{23}H_{18}FO^+$($[M+H]^+$) = 329.1336, found = 329.1335; the ee value was 90%, t_R (minor) = 13.1 min, t_R (major) = 15.5 min (Chiralpak IC, λ = 254 nm, 1% *i*-PrOH/Hexane, flow rate = 1.0 mL/min).

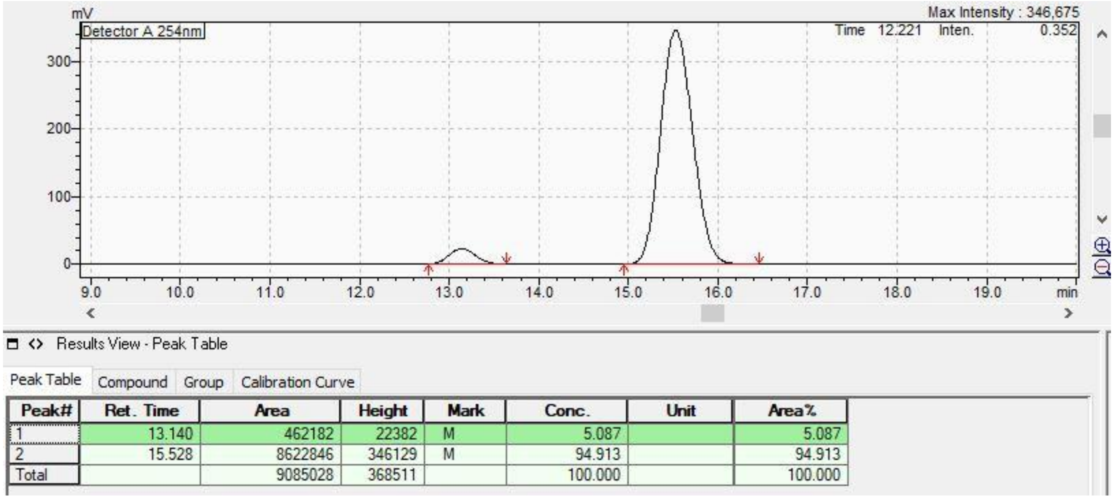

Peak#	Ret. Time	Area	Height	Mark	Conc.	Unit	Area%
1	13.140	462182	22382	M	5.087		5.087
2	15.528	8622846	346129	M	94.913		94.913
Total		9085028	368511		100.000		100.000

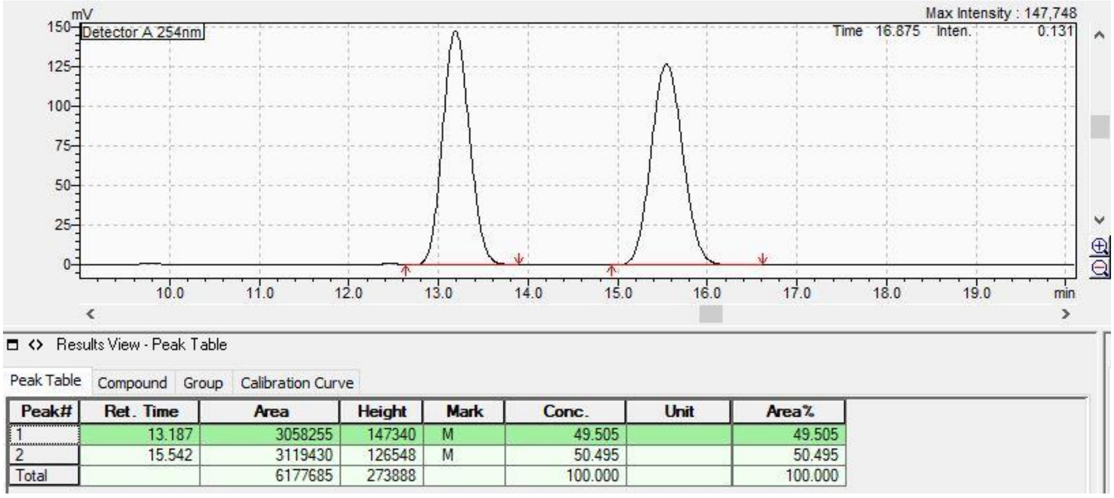

Peak#	Ret. Time	Area	Height	Mark	Conc.	Unit	Area%
1	13.187	3058255	147340	M	49.505		49.505
2	15.542	3119430	126548	M	50.495		50.495
Total		6177685	273888		100.000		100.000

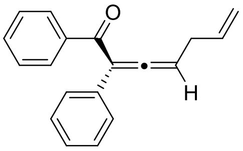

3t

(*R*)-1,2-diphenylhepta-2,3,6-trien-1-one (3t):

GP-B: 17.9 mg, 69% isolated yield; $[\alpha]_D^{25}$ = 45 (c 0.75, $CHCl_3$), light yellow oil, 1:30 ethyl acetate:hexanes as eluent, Rf = 0.3. ^{1}H NMR (400 MHz, $CDCl_3$) δ 7.93 (d, *J* = 7.2 Hz, 2H), 7.55 (t, *J* = 7.2 Hz, 2H), 7.50 – 7.41 (m, 4H), 7.37 (t, *J* = 7.2 Hz, 2H), 7.32 – 7.27 (m, 1H), 5.86 – 5.78 (m, 1H), 5.73 (t, *J* = 7.2 Hz, 1H), 5.16 – 4.90 (m, 2H), 3.05 – 2.87 (m, 2H). ^{13}C NMR (100 MHz, $CDCl_3$) δ 211.1, 193.9, 138.4, 134.8, 133.4, 132.7, 129.4, 128.5, 128.1, 128.0, 127.6, 116.6, 109.3, 95.2, 32.5. HRMS (ESI): m/z calcd. for $C_{19}H_{17}O^+$($[M+H]^+$) = 261.1274, found = 261.1275; the ee value was 90%, t_R (minor) = 14.9 min, t_R (major) = 17.0 min (Chiralpak IC, λ = 254 nm, 1% *i*-PrOH/Hexane, flow rate = 1.0 mL/min).

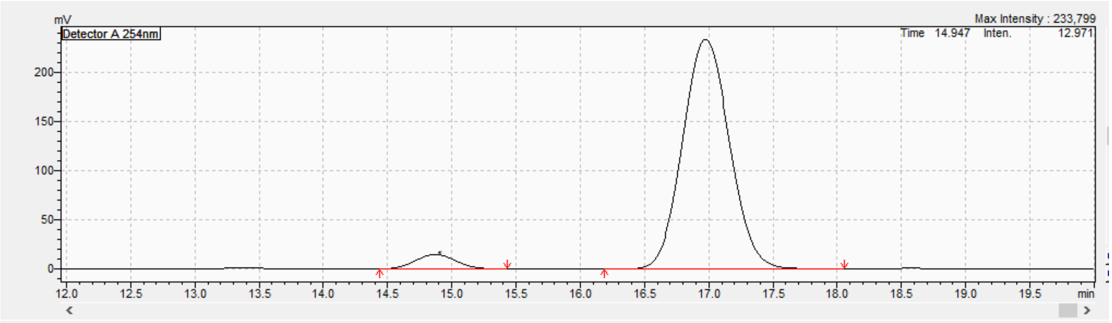

Results View - Peak Table

Peak Table | Compound | Group | Calibration Curve

Peak#	Ret. Time	Area	Height	Mark	Conc.	Unit	ID#	Name	Area%
1	14.870	323847	14856	M	5.181				5.181
2	16.974	5927318	233885		94.819				94.819
Total		6251165	248741		100.000				100.000

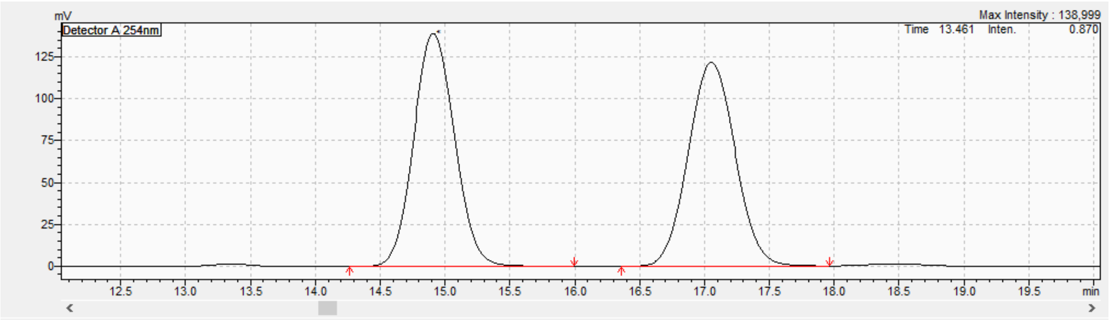

Results View - Peak Table

Peak Table | Compound | Group | Calibration Curve

Peak#	Ret. Time	Area	Height	Mark	Conc.	Unit	ID#	Name	Area%
1	14.912	3046905	139015		49.884				49.884
2	17.052	3061068	121746		50.116				50.116
Total		6107973	260761		100.000				100.000

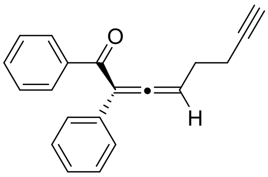

3u

(*R*)-1,2-diphenylocta-2,3-dien-7-yn-1-one (3u):

GP-B: 18.0 mg, 66% isolated yield; $[\alpha]_D^{25}$ = 10.3 (c 0.7, $CHCl_3$), light yellow oil, 1:30 ethyl acetate:hexanes as eluent, Rf = 0.3. ^{1}H NMR (400 MHz, $CDCl_3$) δ 7.92 (d, *J* = 6.8 Hz, 2H), 7.56 (t, *J* = 7.6 Hz, 1H), 7.51 – 7.42 (m, 4H), 7.36 (t, *J* = 7.2 Hz, 2H), 7.31 – 7.26 (m, 1H), 5.80 (t, *J* = 6.8 Hz, 1H), 2.48 – 2.36 (m, 2H), 2.33 – 2.23 (m, 2H), 1.89 (t, *J* = 2.4 Hz, 1H). ^{13}C NMR (100 MHz, $CDCl_3$) δ 210.2, 193.7, 138.3, 133.2, 132.8, 129.4, 128.5, 128.2, 128.0, 127.7, 109.6, 95.7, 82.8, 69.5, 27.8, 18.3. HRMS (ESI): m/z calcd. for $C_{20}H_{17}O^+$($[M+H]^+$) = 273.1274, found = 273.1275; the ee value was 92%, t_R (minor) = 37.4 min, t_R (major) = 42.4 min (Chiralcel® OJ-H, λ = 254 nm, 5% *i*-PrOH/Hexane, flow rate = 1.0 mL/min).

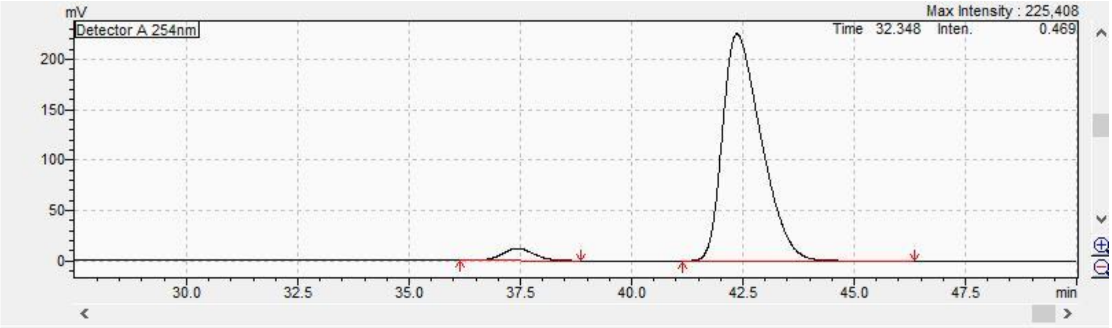

Results View - Peak Table

Peak Table | Compound | Group | Calibration Curve

Peak#	Ret. Time	Area	Height	Mark	Conc.	Unit	Area%
1	37.438	564497	11708	M	3.972		3.972
2	42.367	13647114	225366		96.028		96.028
Total		14211612	237074		100.000		100.000

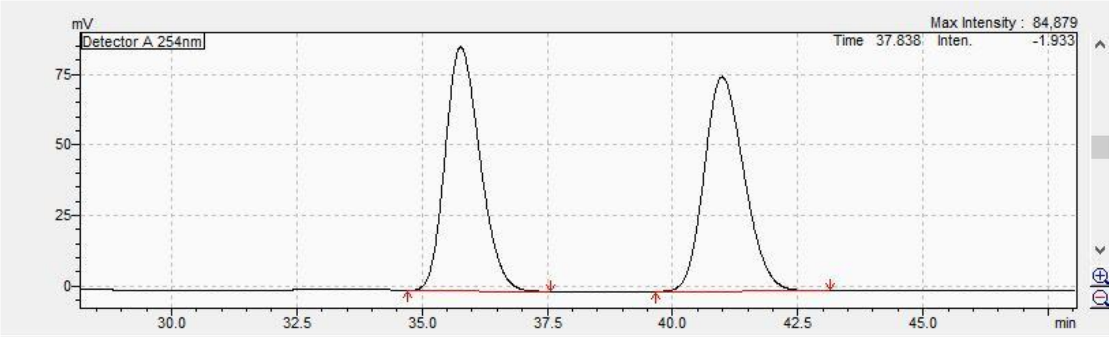

Results View - Peak Table

Peak Table | Compound | Group | Calibration Curve

Peak#	Ret. Time	Area	Height	Mark	Conc.	Unit	Area%
1	35.770	4181710	86550		49.837		49.837
2	40.988	4209034	76016		50.163		50.163
Total		8390744	162566		100.000		100.000

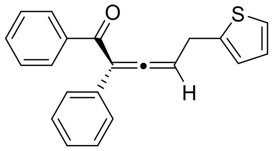

3v

(*R*)-1,2-diphenyl-5-(thiophen-2-yl)penta-2,3-dien-1-one (3v):

GP-B: 20.2 mg, 64% isolated yield; $[\alpha]_D^{25}$ = 21.5 (c 0.8, $CHCl_3$), light yellow oil, 1:3 dichloromethane:hexanes as eluent, Rf = 0.3. ^1H NMR (400 MHz, $CDCl_3$) δ 7.86 (d, J = 7.2 Hz, 2H), 7.55 (t, J = 7.6 Hz, 1H), 7.48 – 7.34 (m, 6H), 7.30 (t, J = 7.2 Hz, 1H), 7.13 (dd, J = 4.8, 0.8 Hz, 1H), 6.89 (dd, J = 4.8, 3.2 Hz, 1H), 6.71 (dd, J = 3.6, 1.2 Hz, 1H), 3.76 – 3.64 (m, 2H). ^{13}C NMR (100 MHz, $CDCl_3$) δ 210.5, 193.6, 141.0, 138.3, 133.1, 132.8, 129.4, 128.5, 128.2, 128.04, 127.8, 126.9, 125.3, 124.2, 110.1, 96.3, 29.1. HRMS (ESI): m/z calcd. for $C_{21}H_{12}NaOS^+$([M+Na]$^+$) = 339.0814, found = 339.0816; the ee value was 90%, t_R (minor) = 10.7 min, t_R (major) = 11.3 min (Chiralpak IC, λ = 254 nm, 4% *i*-PrOH/Hexane, flow rate = 1.0 mL/min).

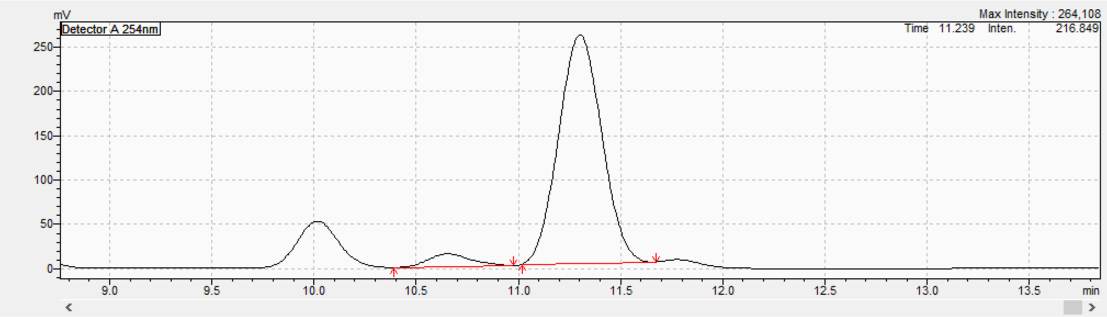

Results View - Peak Table

Peak Table | Compound | Group | Calibration Curve

Peak#	Ret. Time	Area	Height	Mark	Conc.	Unit	ID#	Name	Area%
1	10.655	208752	14449	M	5.226				5.226
2	11.301	3786121	257920	M	94.774				94.774
Total		3994873	272369		100.000				100.000

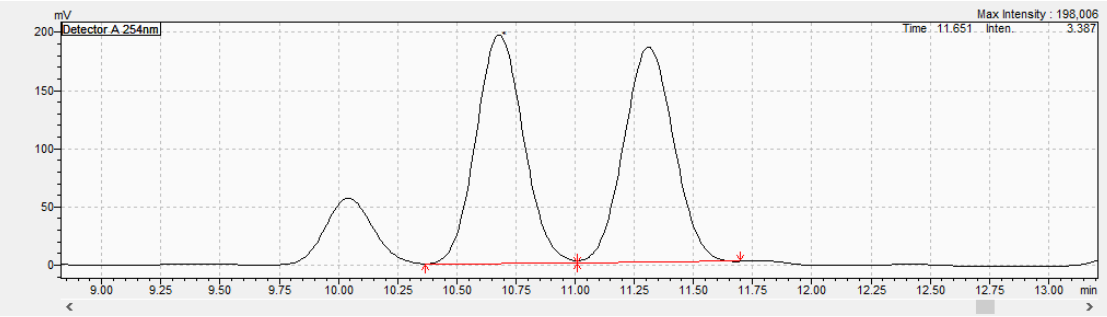

Results View - Peak Table

Peak Table | Compound | Group | Calibration Curve

Peak#	Ret. Time	Area	Height	Mark	Conc.	Unit	ID#	Name	Area%
1	10.679	2728728	196276	M	50.169				50.169
2	11.310	2710341	184885	V M	49.831				49.831
Total		5439069	381160		100.000				100.000

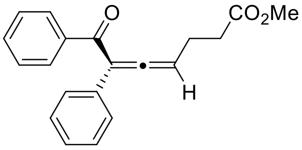

3w

methyl (*R*)-7-oxo-6,7-diphenylhepta-4,5-dienoate (3w):

GP-B: 21.1 mg, 69% isolated yield; $[\alpha]_D^{25}$ = 10.1 (c 0.9, $CHCl_3$), light yellow oil, 1:20 ethyl acetate:hexanes as eluent, Rf = 0.3. ^{1}H NMR (400 MHz, $CDCl_3$) δ 7.91 (d, *J* = 7.2 Hz, 2H), 7.56 (t, *J* = 7.6 Hz, 1H), 7.49 – 7.41 (m, 4H), 7.35 (t, *J* = 7.2 Hz, 2H), 7.32 – 7.25 (m, 1H), 5.78 (t, *J* = 6.4 Hz, 1H), 3.56 (s, 3H), 2.54 – 2.38 (m, 4H). ^{13}C NMR (100 MHz, $CDCl_3$) δ 209.9, 193.6, 172.7, 138.2, 133.2, 132.8, 129.4, 128.5, 128.2, 127.9, 127.7, 109.9, 96.0, 51.7, 32.9, 23.7. HRMS (ESI): m/z calcd. for $C_{20}H_{19}O_3^+$([M+H]$^+$) = 307.1329, found = 307.1330; the ee value was 90%, t_R (minor) = 11.9 min, t_R (major) = 13.6 min (Chiralpak IC, λ = 254 nm, 15% *i*-PrOH/Hexane, flow rate = 1.0 mL/min).

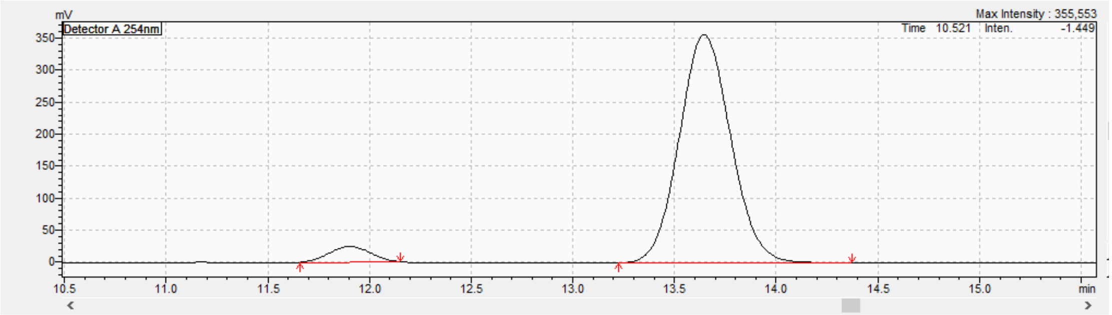

Results View - Peak Table

Peak Table | Compound | Group | Calibration Curve

Peak#	Ret. Time	Area	Height	Mark	Conc.	Unit	ID#	Name	Area%
1	11.903	340748	24194	M	5.145				5.145
2	13.647	6282308	356529	M	94.855				94.855
Total		6623056	380724		100.000				100.000

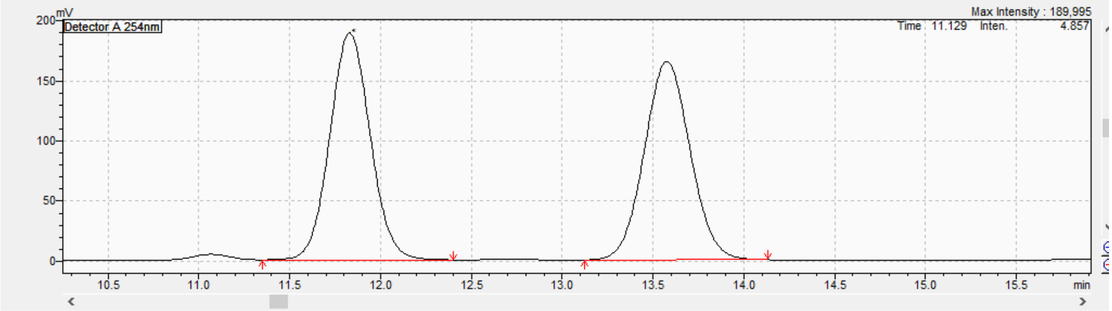

Results View - Peak Table

Peak Table | Compound | Group | Calibration Curve

Peak#	Ret. Time	Area	Height	Mark	Conc.	Unit	ID#	Name	Area%
1	11.830	2917533	188837	M	50.100				50.100
2	13.576	2905883	165173	M	49.900				49.900
Total		5823417	354010		100.000				100.000

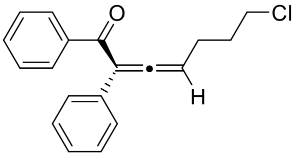

3x

(*R*)-7-chloro-1,2-diphenylhepta-2,3-dien-1-one (3x):

GP-B: 22.2 mg, 75% isolated yield; $[\alpha]_D^{25}$ = 5.6 (c 0.5, $CHCl_3$), light yellow oil, 1:30 ethyl acetate:hexanes as eluent, Rf = 0.3. ^{1}H NMR (400 MHz, $CDCl_3$) δ 7.91 (d, *J* = 6.8 Hz, 2H), 7.56 (t, *J* = 7.6 Hz, 1H), 7.48 – 7.34 (m, 6H), 7.32 – 7.27 (m, 1H), 5.70 (t, *J* = 7.2 Hz, 1H), 3.43 (t, *J* = 6.4 Hz, 2H), 2.38 – 2.32 (m, 2H), 2.00 – 1.77 (m, 2H). ^{13}C NMR (100 MHz, $CDCl_3$) δ 210.7, 193.8, 138.4, 133.3, 132.8, 129.4, 128.5, 128.2, 127.9, 127.7, 109.5, 95.8, 43.9, 31.5, 25.6. HRMS (ESI): m/z calcd. for $C_{19}H_{18}ClO^+$($[M+H]^+$) = 297.1041, found = 297.1042; the ee value was 86%, t_R (minor) = 13.1 min, t_R (major) = 14.0 min (Chiralpak IC, λ = 254 nm, 2% *i*-PrOH/Hexane, flow rate = 1.0 mL/min).

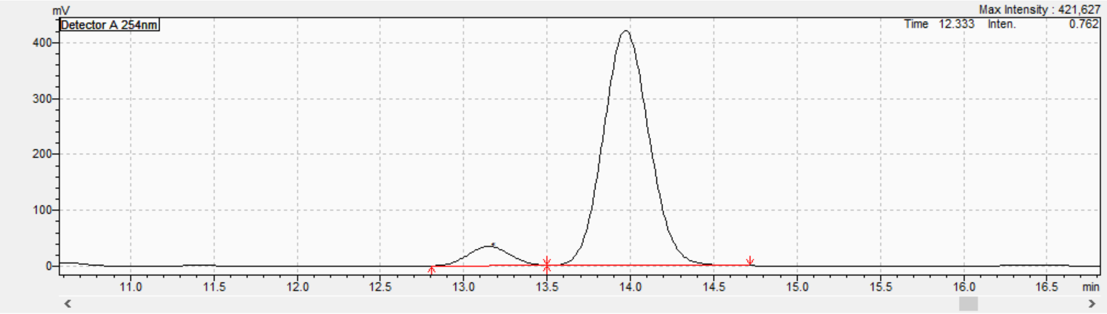

Results View - Peak Table

Peak Table | Compound | Group | Calibration Curve

Peak#	Ret. Time	Area	Height	Mark	Conc.	Unit	ID#	Name	Area%
1	13.150	596025	34143	M	6.908				6.908
2	13.971	8031760	419936	M	93.092				93.092
Total		8627785	454080		100.000				100.000

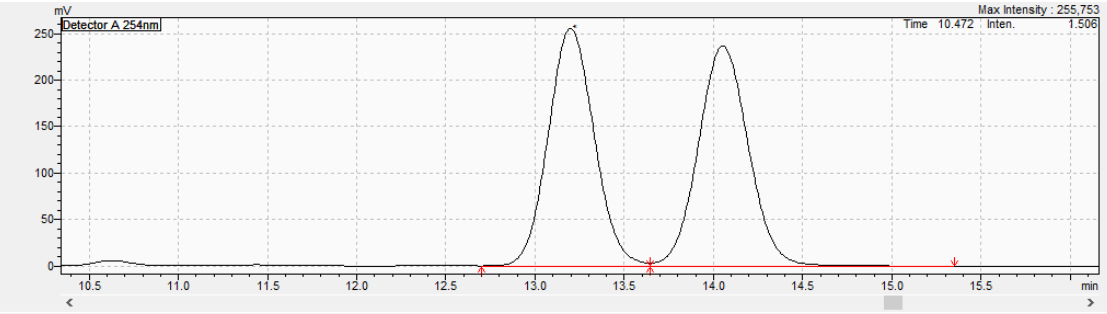

Results View - Peak Table

Peak Table | Compound | Group | Calibration Curve

Peak#	Ret. Time	Area	Height	Mark	Conc.	Unit	ID#	Name	Area%
1	13.199	4594443	255874		49.829				49.829
2	14.052	4625945	237344	V	50.171				50.171
Total		9220388	493218		100.000				100.000

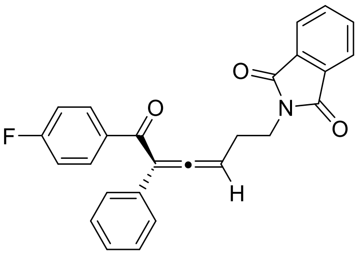

3y

(*R*)-2-(6-(4-fluorophenyl)-6-oxo-5-phenylhexa-3,4-dien-1-yl)isoindoline-1,3-dione (3y):

GP-B: 29.6 mg, 72% isolated yield; $[\alpha]_D^{25}$ = 5.8 (c 0.6, $CHCl_3$), light yellow oil, 1:15 to 1:10 ethyl acetate:hexanes as eluent, Rf = 0.3. ^{1}H NMR (400 MHz, $CDCl_3$) δ 7.90 (dd, *J* = 8.8, 5.6 Hz, 2H), 7.80 – 7.75 (m, 2H), 7.74 – 7.68 (m, 2H), 7.34 – 7.28 (m, 2H), 7.22 – 7.16 (m, 3H), 7.06 (d, *J* = 8.7 Hz, 2H), 5.73 (t, *J* = 7.6 Hz, 1H), 3.99 – 3.56 (m, 2H), 2.81 – 2.41 (m, 2H). ^{13}C NMR (100 MHz, $CDCl_3$) δ 210.4, 191.9, 168.1, 165.5 (d, *J* = 254.5 Hz), 134.3 (d, *J* = 3.0 Hz), 134.0, 131.9 (d, *J* = 9.3 Hz), 131.9, 131.8, 128.4, 127.8, 127.7, 123.3, 115.4 (d, *J* = 21.9 Hz), 108.9, 93.7, 36.9, 28.0. ^{19}F NMR (377 MHz, $CDCl_3$) δ -105.0. HRMS (ESI): m/z calcd. for $C_{26}H_{19}FNO_3^+$([M+H]$^+$) = 412.1343, found = 412.1345; the ee value was 86%, t_R (minor) = 32.7 min, t_R (major) = 35.3 min (Chiralpak IG, λ = 254 nm, 15% *i*-PrOH/Hexane, flow rate = 1.0 mL/min).

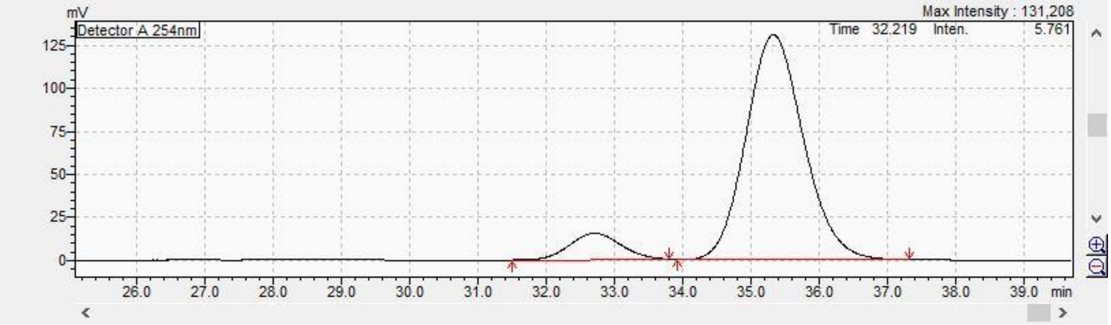

Results View - Peak Table

Peak Table | Compound | Group | Calibration Curve

Peak#	Ret. Time	Area	Height	Mark	Conc.	Unit	Area%
1	32.708	796314	15260	M	9.422		9.422
2	35.331	7654950	130631	M	90.578		90.578
Total		8451264	145891		100.000		100.000

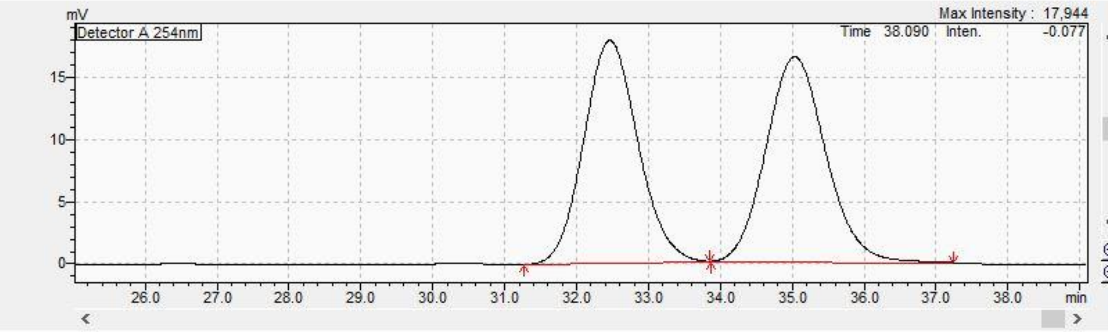

Results View - Peak Table

Peak Table | Compound | Group | Calibration Curve

Peak#	Ret. Time	Area	Height	Mark	Conc.	Unit	Area%
1	32.463	936213	17875	M	49.641		49.641
2	35.032	949766	16468	M	50.359		50.359
Total		1885979	34344		100.000		100.000

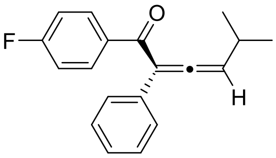

3z

(*R*)-1-(4-fluorophenyl)-5-methyl-2-phenylhexa-2,3-dien-1-one (3z):

GP-B: 20.4 mg, 73% isolated yield; $[\alpha]_D^{25}$ = 15.6 (c 0.8, $CHCl_3$), light yellow oil, 1:30 ethyl acetate:hexanes as eluent, Rf = 0.3. ^{1}H NMR (400 MHz, $CDCl_3$) δ 7.95 (dd, *J* = 8.8, 5.6 Hz, 2H), 7.45 (d, *J* = 7.2 Hz, 2H), 7.36 (t, *J* = 7.6 Hz, 2H), 7.31 – 7.24 (m, 1H), 7.11 (t, *J* = 8.8 Hz, 2H), 5.73 (d, *J* = 6.8 Hz, 1H), 2.60 – 2.41 (m, 1H), 1.05 (t, *J* = 6.8 Hz, 6H). ^{13}C NMR (100 MHz, $CDCl_3$) δ 209.1, 192.4, 165.4 (d, *J* = 254.0 Hz), 134.6 (d, *J* = 3.0 Hz), 133.5, 132.0 (d, *J* = 9.2 Hz), 128.5, 127.8, 127.6, 115.2 (d, *J* = 21.9 Hz), 110.1, 104.6, 28.9, 22.4, 22.4. ^{19}F NMR (471 MHz, $CDCl_3$) δ -105.9. HRMS (ESI): m/z calcd. for $C_{19}H_{18}FO^+$($[M+H]^+$) = 281.1336, found = 281.1337; the ee value was 90%, t_R (minor) = 17.4 min, t_R (major) = 15.1 min (Chiralpak IG, λ = 254 nm, 1% *i*-PrOH/Hexane, flow rate = 1.0 mL/min).

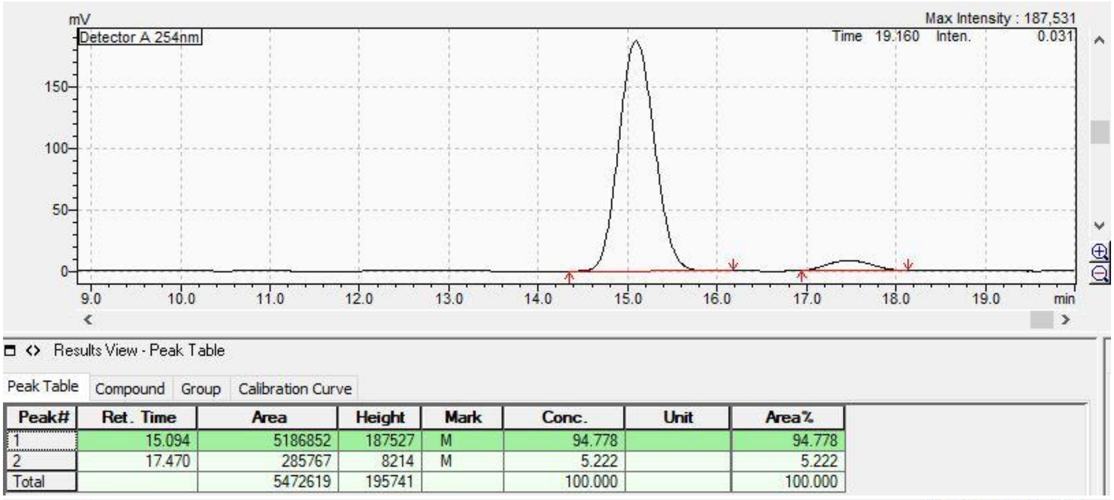

Peak#	Ret. Time	Area	Height	Mark	Conc.	Unit	Area%
1	15.094	5186852	187527	M	94.778		94.778
2	17.470	285767	8214	M	5.222		5.222
Total		5472619	195741		100.000		100.000

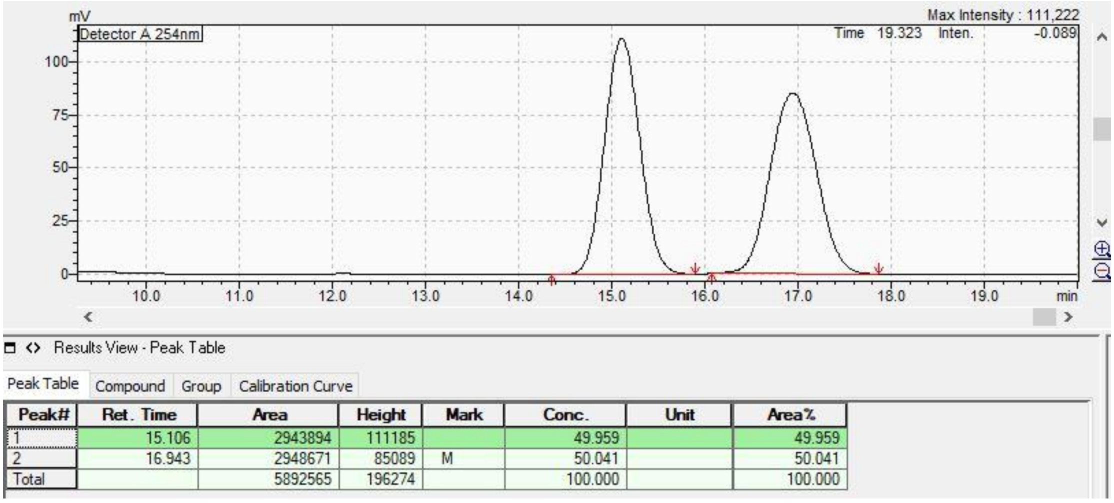

Peak#	Ret. Time	Area	Height	Mark	Conc.	Unit	Area%
1	15.106	2943894	111185		49.959		49.959
2	16.943	2948671	85089	M	50.041		50.041
Total		5892565	196274		100.000		100.000

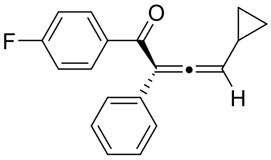

3aa

(*R*)-4-cyclopropyl-1-(4-fluorophenyl)-2-phenylbuta-2,3-dien-1-one (3aa):

GP-B: 17.0 mg, 61% isolated yield; $[\alpha]_D^{25}$ = 14.1 (c 0.6, $CHCl_3$), light yellow oil, 1:30 ethyl acetate:hexanes as eluent, Rf = 0.3. ^{1}H NMR (400 MHz, $CDCl_3$) δ 7.98 (dd, *J* = 8.8, 5.6 Hz, 2H), 7.44 (d, *J* = 7.2 Hz, 2H), 7.36 (t, *J* = 7.6 Hz, 2H), 7.31 – 7.24 (m, 1H), 7.11 (t, *J* = 8.8 Hz, 3H), 5.52 (d, *J* = 8.0 Hz, 1H), 1.51 – 1.41 (m, 1H), 0.87 – 0.76 (m, 2H), 0.50 – 0.37 (m, 2H). ^{13}C NMR (100 MHz, $CDCl_3$) δ 210.5, 192.1, 165.6 (d, *J* = 254.2 Hz), 134.5 (d, *J* = 3.0 Hz), 133.4, 132.1 (d, *J* = 9.2 Hz), 128.5, 127.9, 127.7, 115.2 (d, *J* = 21.9 Hz), 110.1, 102.0, 9.5, 7.0, 6.9. ^{19}F NMR (471 MHz, $CDCl_3$) δ -105.7. HRMS (ESI): m/z calcd. for $C_{19}H_{16}FO^+$($[M+H]^+$) = 279.1180, found = 279.1181; the ee value was 89%, t_R (minor) = 11.7 min, t_R (major) = 12.9 min (Chiralpak IC, λ = 254 nm, 1% *i*-PrOH/Hexane, flow rate = 1.0 mL/min).

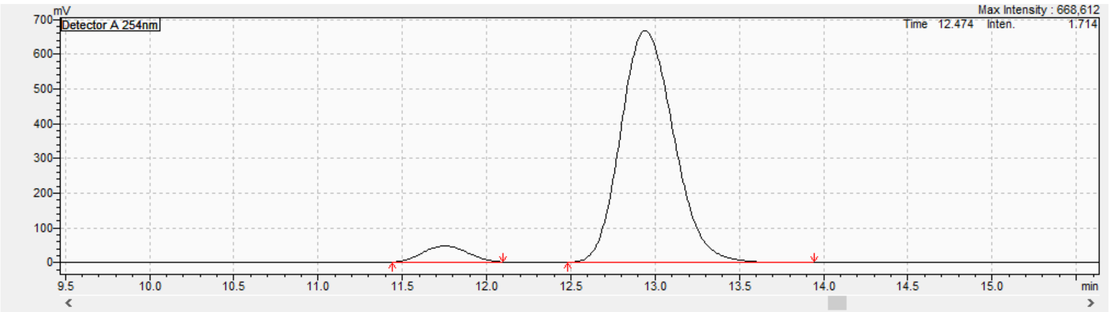

Results View - Peak Table

Peak Table | Compound | Group | Calibration Curve

Peak#	Ret. Time	Area	Height	Mark	Conc.	Unit	ID#	Name	Area%
1	11.749	855600	46426	M	5.542				5.542
2	12.941	14583648	667279	M	94.458				94.458
Total		15439248	713706		100.000				100.000

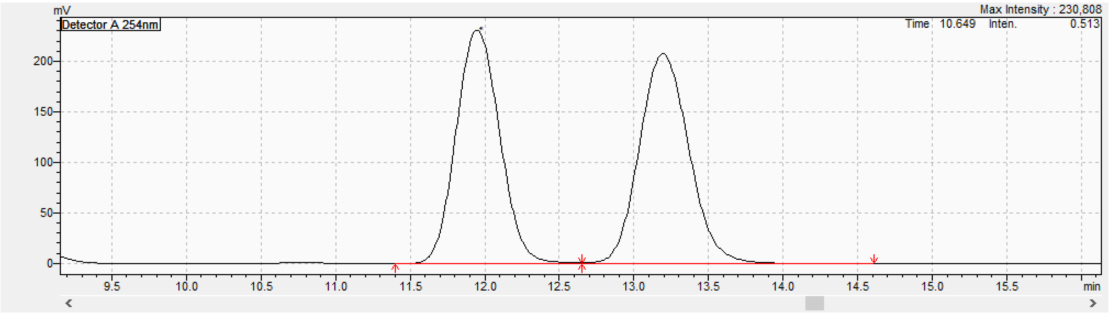

Results View - Peak Table

Peak Table | Compound | Group | Calibration Curve

Peak#	Ret. Time	Area	Height	Mark	Conc.	Unit	ID#	Name	Area%
1	11.948	4714484	231135	M	49.538				49.538
2	13.195	4802515	207504	V M	50.462				50.462
Total		9516999	438639		100.000				100.000

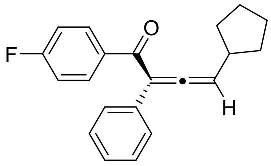

3ab

(*R*)-4-cyclopentyl-1-(4-fluorophenyl)-2-phenylbuta-2,3-dien-1-one (3ab):

GP-B: 19.6 mg, 64% isolated yield; $[\alpha]_D^{25}$ = 4.2 (c 0.5, $CHCl_3$), light yellow oil, 1:30 ethyl acetate:hexanes as eluent, Rf = 0.3. ^1H NMR (400 MHz, $CDCl_3$) δ 7.95 (dd, *J* = 8.8, 5.6 Hz, 2H), 7.45 (d, *J* = 7.2 Hz, 2H), 7.36 (t, *J* = 7.6 Hz, 2H), 7.31 – 7.24 (m, 1H), 7.10 (t, *J* = 8.8 Hz, 2H), 5.76 (d, *J* = 7.2 Hz, 1H), 2.73 – 2.58 (m, 1H), 1.92 – 1.75 (m, 2H), 1.63 – 1.52 (m, 4H), 1.44 – 1.29 (m, 2H). ^{13}C NMR (100 MHz, $CDCl_3$) δ 209.9, 192.4, 165.4 (d, *J* = 254.0 Hz), 134.7 (d, *J* = 3.0 Hz), 133.5, 132.0 (d, *J* = 9.2 Hz), 128.5, 127.8, 127.6, 115.1 (d, *J* = 21.9 Hz), 109.6, 102.4, 39.3, 32.9, 32.6, 24.8, 24.8. ^{19}F NMR (471 MHz, $CDCl_3$) δ -106.0. HRMS (ESI): m/z calcd. for $C_{21}H_{20}FO^+$([M+H]$^+$) = 307.1493, found = 307.1496; the ee value was 90%, t_R (minor) = 22.2 min, t_R (major) = 20.6 min (Chiralpak IG, λ = 254 nm, 1% *i*-PrOH/Hexane, flow rate = 1.0 mL/min).

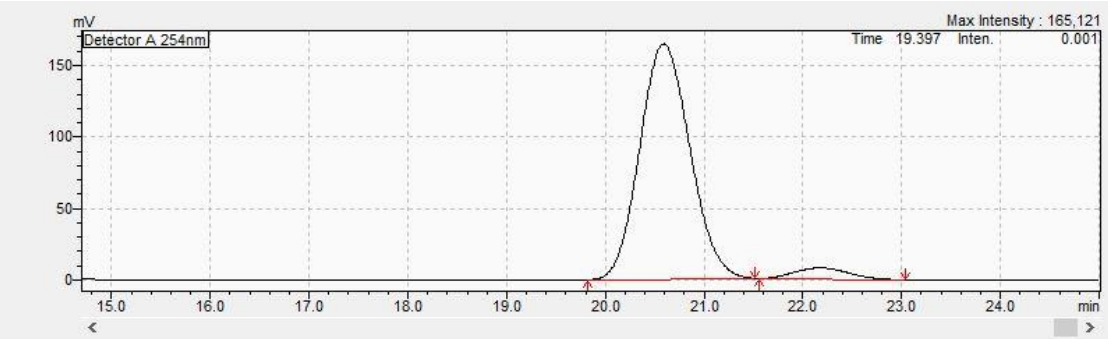

Results View - Peak Table

Peak Table | Compound | Group | Calibration Curve

Peak#	Ret. Time	Area	Height	Mark	Conc.	Unit	Area%
1	20.591	5808727	164472	M	95.182		95.182
2	22.172	294048	7744	M	4.818		4.818
Total		6102775	172217		100.000		100.000

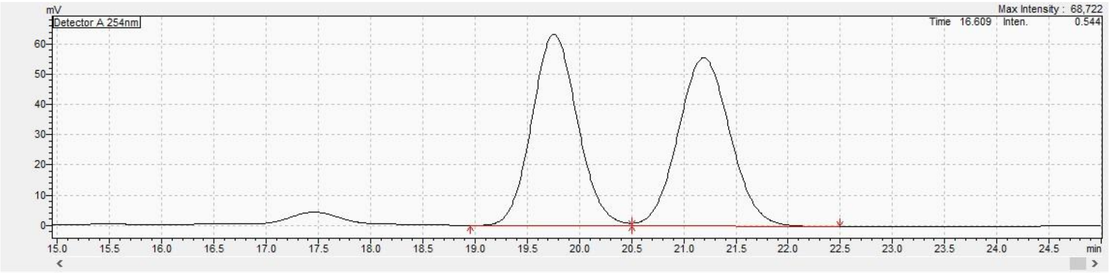

Results View - Peak Table

Peak Table | Compound | Group | Calibration Curve

Peak#	Ret. Time	Area	Height	Mark	Conc.	Unit	ID#	Name	Area%
1	19.757	1926980	63475		49.874				49.874
2	21.191	1936680	55889	V	50.126				50.126
Total		3863660	119364		100.000				100.000

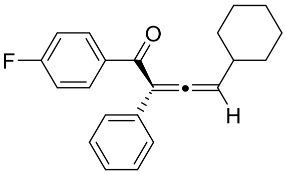

3ac

(*R*)-4-cyclohexyl-1-(4-fluorophenyl)-2-phenylbuta-2,3-dien-1-one (3ac):

GP-B: 22.4 mg, 70% isolated yield; $[\alpha]_D^{25}$ = 15.4 (c 0.9, $CHCl_3$), light yellow oil, 1:30 ethyl acetate:hexanes as eluent, Rf = 0.3. ^{1}H NMR (400 MHz, $CDCl_3$) δ 7.94 (dd, *J* = 8.8, 5.6 Hz, 2H), 7.45 (d, *J* = 7.2 Hz, 2H), 7.36 (t, *J* = 7.2 Hz, 2H), 7.32 – 7.24 (m, 1H), 7.10 (t, *J* = 8.8 Hz, 2H), 5.71 (d, *J* = 6.8 Hz, 1H), 2.26 – 2.14 (m, 1H), 1.83 – 1.62 (m, 4H), 1.35 – 1.21 (m, 3H), 1.19 – 1.05 (m, 3H). ^{13}C NMR (100 MHz, $CDCl_3$) δ 209.7, 192.5, 165.4 (d, *J* = 254.0 Hz), 134.7 (d, *J* = 3.0 Hz), 133.6, 132.0 (d, *J* = 9.2 Hz), 128.5, 127.8, 127.6, 115.1 (d, *J* = 21.8 Hz), 109.8, 103.3, 38.0, 32.9, 32.8, 25.8, 25.8. ^{19}F NMR (377 MHz, $CDCl_3$) δ -105.9. HRMS (ESI): m/z calcd. for $C_{22}H_{22}FO^+$($[M+H]^+$) = 321.1649, found = 321.1652; the ee value was 92%, t_R (minor) = 16.0 min, t_R (major) = 20.1 min (Chiralpak IG, λ = 254 nm, 1% *i*-PrOH/Hexane, flow rate = 1.0 mL/min).

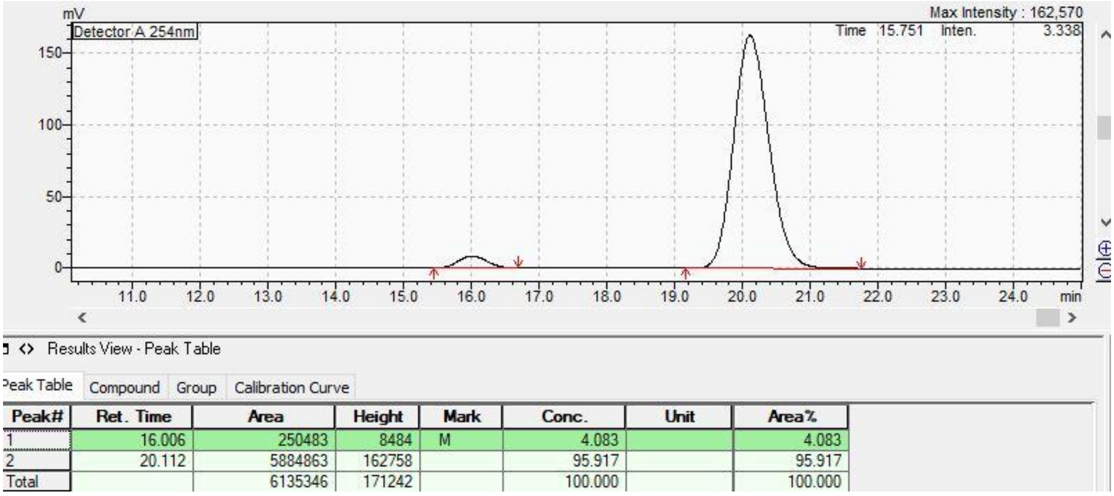

Peak#	Ret. Time	Area	Height	Mark	Conc.	Unit	Area%
1	16.006	250483	8484	M	4.083		4.083
2	20.112	5884863	162758		95.917		95.917
Total		6135346	171242		100.000		100.000

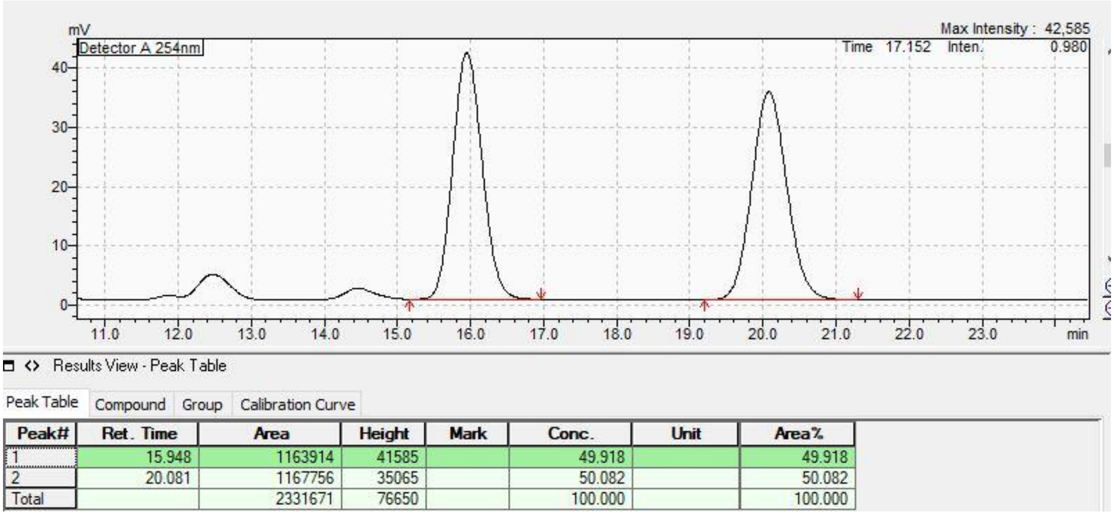

Peak#	Ret. Time	Area	Height	Mark	Conc.	Unit	Area%
1	15.948	1163914	41585		49.918		49.918
2	20.081	1167756	35065		50.082		50.082
Total		2331671	76650		100.000		100.000

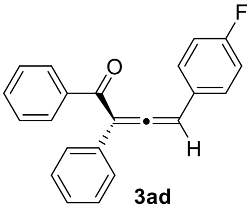

3ad

(*R*)-4-(4-fluorophenyl)-1,2-diphenylbuta-2,3-dien-1-one (3ad):

GP-B: 12 mg, 38% isolated yield; $[\alpha]_D^{25}$ = 2.7 (c 0.6, $CHCl_3$), light yellow oil, 1:15 ethyl acetate:hexanes as eluent, Rf = 0.3. ^{1}H NMR (500 MHz, $CDCl_3$) δ 7.97 (d, *J* = 7.2 Hz, 2H), 7.58 – 7.48 (m, 3H), 7.42 – 7.36 (m, 4H), 7.33 – 7.29 (m, 3H), 7.03 (t, *J* = 8.6 Hz, 2H), 6.72 (s, 1H). ^{13}C NMR (125 MHz, $CDCl_3$) δ 211.7, 192.7, 162.5 (d, *J* = 248.5 Hz), 137.9, 133.2, 132.7, 129.4, 129.0 (d, *J* = 8.3 Hz), 128.7, 128.3, 128.6, 128.0 (d, *J* = 3.2 Hz), 127.9, 116.0 (d, *J* = 21.9 Hz), 112.4, 99.6. ^{19}F NMR (471 MHz, $CDCl_3$) δ -113.1. HRMS (APCI): m/z calcd. for $C_{22}H_{16}FO^+$($[M+H]^+$) = 315.1180, found = 315.1181; the ee value was 16%, t_R (minor) = 10.9 min, t_R (major) = 11.8 min (Chiralpak IC, λ = 254 nm, 1% *i*-PrOH/Hexane, flow rate = 1.0 mL/min).

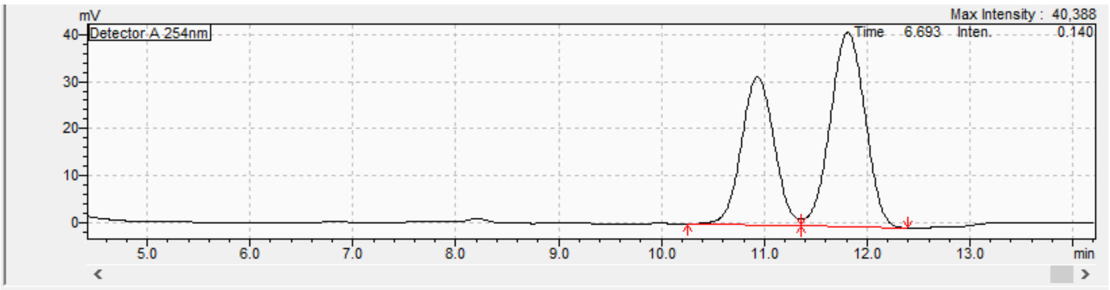

Results View - Peak Table

Peak Table | Compound | Group | Calibration Curve

Peak#	Ret. Time	Area	Height	Mark	Conc.	Area%
1	10.933	695011	31410		41.815	41.815
2	11.811	967099	41228	V	58.185	58.185
Total		1662110	72638		100.000	100.000

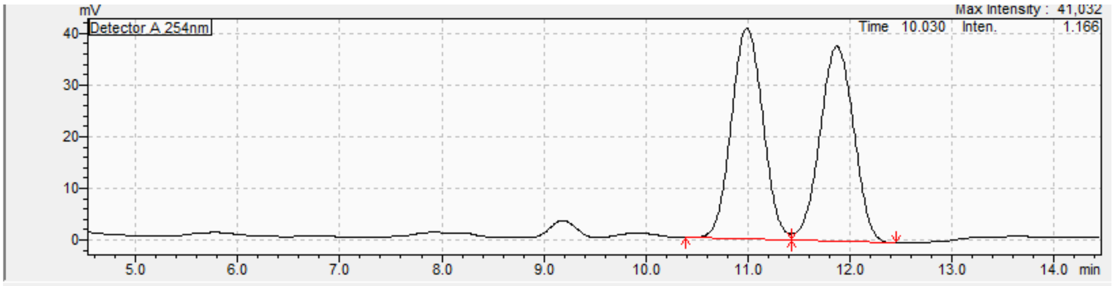

Results View - Peak Table

Peak Table | Compound | Group | Calibration Curve

Peak#	Ret. Time	Area	Height	Mark	Conc.	Area%
1	10.989	881144	40906		50.264	50.264
2	11.873	871889	37785	V	49.736	49.736
Total		1753033	78691		100.000	100.000

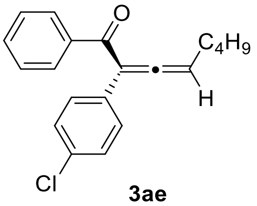

3ae

(*R*)-2-(4-chlorophenyl)-1-phenylocta-2,3-dien-1-one (3ae):

GP-B: 23.9 mg, 77% isolated yield; $[\alpha]_D^{25}$ = 43.7 (c 1, $CHCl_3$), light yellow oil, 1:30 ethyl acetate:hexanes as eluent, Rf = 0.3. ^{1}H NMR (400 MHz, $CDCl_3$) δ 7.88 (d, J = 6.8 Hz, 2H), 7.55 (t, J = 7.6 Hz, 1H), 7.46 – 7.40 (m, 4H), 7.33 (d, J = 8.8 Hz, 2H), 5.70 (t, J = 7.2 Hz, 1H), 2.26 – 2.07 (m, 2H), 1.44 – 1.32 (m, 2H), 1.31 – 1.20 (m, 2H), 0.84 (t, J = 7.2 Hz, 3H). ^{13}C NMR (100 MHz, $CDCl_3$) δ 211.3, 193.8, 138.4, 133.3, 132.6, 132.1, 129.3, 129.3, 128.6, 128.1, 108.1, 97.7, 31.0, 28.1, 22.1, 13.7. HRMS (ESI): m/z calcd. for $C_{20}H_{20}ClO^+([M+H]^+)$ = 311.1197, found = 311.1196; the ee value was 90%, t_R (minor) = 25.9 min, t_R (major) = 28.4 min (Chiralpak IG, λ = 254 nm, 1% *i*-PrOH/Hexane, flow rate = 1.0 mL/min).

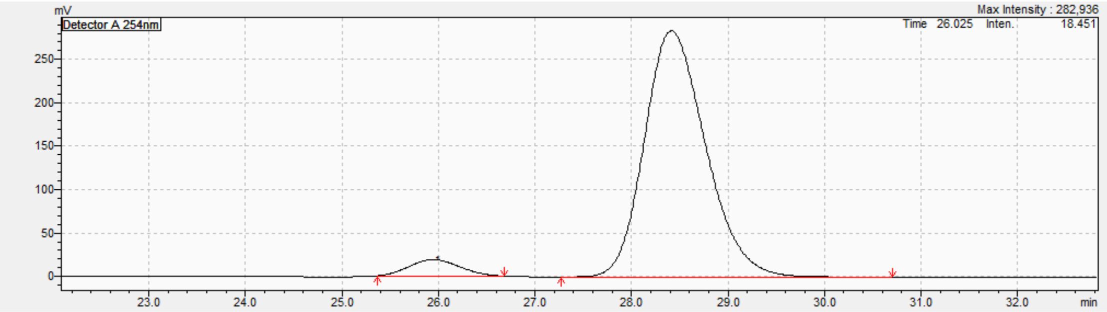

Results View - Peak Table

Peak Table | Compound | Group | Calibration Curve

Peak#	Ret. Time	Area	Height	Mark	Conc.	Unit	ID#	Name	Area%
1	25.945	683672	18818	M	5.189				5.189
2	28.415	12490604	283501		94.811				94.811
Total		13174276	302319		100.000				100.000

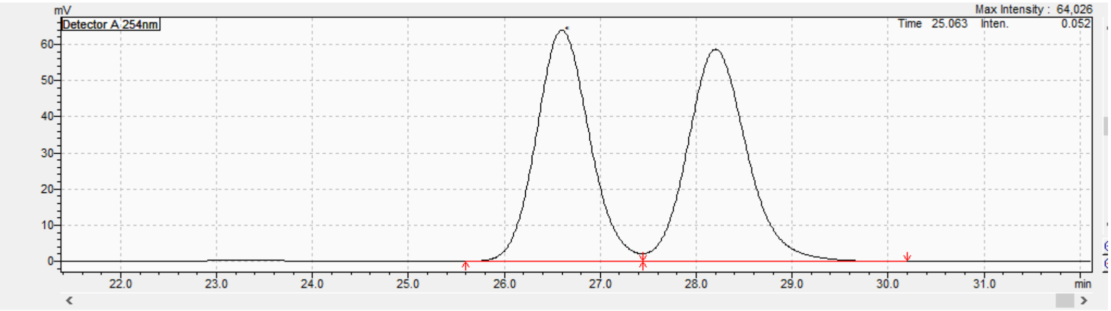

Results View - Peak Table

Peak Table | Compound | Group | Calibration Curve

Peak#	Ret. Time	Area	Height	Mark	Conc.	Unit	ID#	Name	Area%
1	26.600	2456797	63997		49.436				49.436
2	28.203	2512813	58668	V	50.564				50.564
Total		4969610	122665		100.000				100.000

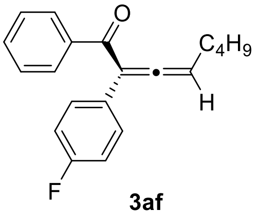

3af

(*R*)-2-(4-fluorophenyl)-1-phenylocta-2,3-dien-1-one (3af):

GP-B: 23.2 mg, 79% isolated yield; $[\alpha]_D^{25}$ = 22.6 (c 1, $CHCl_3$), light yellow oil, 1:30 ethyl acetate:hexanes as eluent, Rf = 0.3. ^{1}H NMR (400 MHz, $CDCl_3$) δ 7.88 (d, *J* = 7.2 Hz, 2H), 7.55 (t, *J* = 7.6 Hz, 1H), 7.48 – 7.40 (m, 4H), 7.05 (t, *J* = 8.8 Hz, 2H), 5.69 (t, *J* = 7.6 Hz, 1H), 2.28 – 2.07 (m, 2H), 1.44 – 1.31 (m, 2H), 1.30 – 1.19 (m, 2H), 0.84 (t, *J* = 7.2 Hz, 3H). ^{13}C NMR (100 MHz, $CDCl_3$) δ 211.4, 211.4, 194.1, 162.2 (d, *J* = 247.0 Hz), 138.6, 132.6, 129.8 (d, *J* = 8.1 Hz), 129.5 (d, *J* = 3.4 Hz), 129.3, 128.1, 115.4 (d, *J* = 21.6 Hz), 108.1, 97.4, 31.0, 28.1, 22.0, 13.7. ^{19}F NMR (471 MHz, $CDCl_3$) δ -114.6. HRMS (ESI): m/z calcd. for $C_{20}H_{20}FO^+$([M+H]$^+$) = 295.1493, found = 295.1491; the ee value was 92%, t_R (minor) = 22.0 min, t_R (major) = 11.5 min (Chiralcel® OJ-H, λ = 254 nm, 1% *i*-PrOH/Hexane, flow rate = 1.0 mL/min).

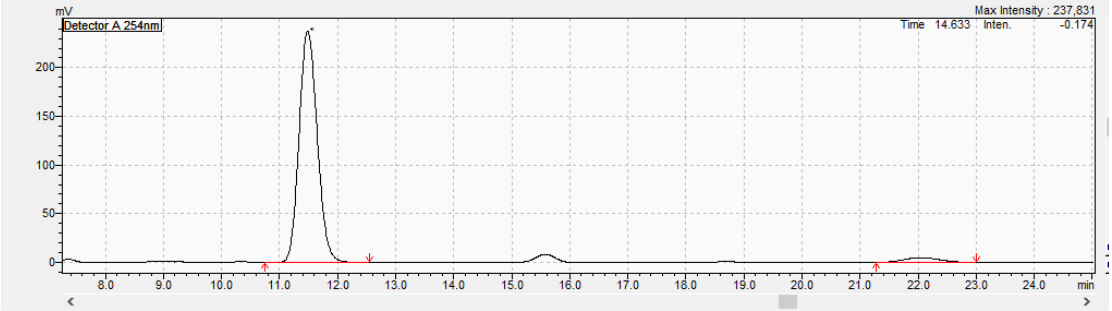

Results View - Peak Table

Peak Table | Compound | Group | Calibration Curve

Peak#	Ret. Time	Area	Height	Mark	Conc.	Unit	ID#	Name	Area%
1	11.486	5160743	237855	M	95.918				95.918
2	22.043	219620	5078	M	4.082				4.082
Total		5380363	242933		100.000				100.000

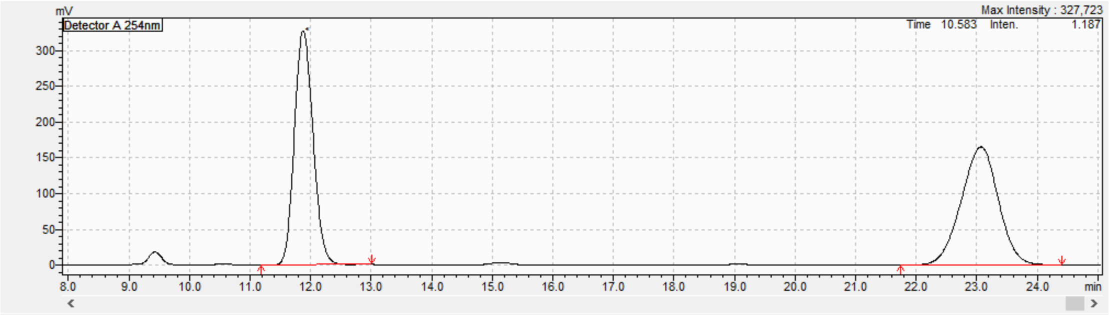

Results View - Peak Table

Peak Table | Compound | Group | Calibration Curve

Peak#	Ret. Time	Area	Height	Mark	Conc.	Unit	ID#	Name	Area%
1	11.877	6989660	326977	M	49.807				49.807
2	23.071	7043843	164952	M	50.193				50.193
Total		14033503	491929		100.000				100.000

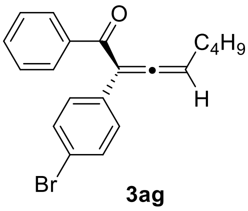

3ag

(*R*)-2-(4-bromophenyl)-1-phenylocta-2,3-dien-1-one (3ag):

GP-B: 28.7 mg, 79% isolated yield; $[\alpha]_D^{25}$ = 14.3 (c 0.9, $CHCl_3$), light yellow oil, 1:30 ethyl acetate:hexanes as eluent, Rf = 0.3. ^{1}H NMR (400 MHz, $CDCl_3$) δ 7.87 (d, *J* = 7.2 Hz, 2H), 7.55 (t, *J* = 7.6 Hz, 1H), 7.50 – 7.39 (m, 4H), 7.36 (d, *J* = 8.8 Hz, 2H), 5.69 (t, *J* = 7.2 Hz, 1H), 2.26 – 2.10 (m, 2H), 1.49 – 1.33 (m, 2H), 1.32 – 1.22 (m, 2H), 0.84 (t, *J* = 7.2 Hz, 3H). ^{13}C NMR (100 MHz, $CDCl_3$) δ 211.3, 193.7, 138.4, 132.6, 131.5, 129.7, 129.3, 128.1, 121.5, 108.1, 97.7, 31.0, 28.1, 22.1, 13.7. HRMS (ESI): m/z calcd. for $C_{20}H_{20}BrO^+$($[M+H]^+$) = 355.0692, found = 355.0691; the ee value was 88%, t_R (minor) = 9.8 min, t_R (major) = 10.5 min (Chiralpak IC, λ = 254 nm, 1% *i*-PrOH/Hexane, flow rate = 1.0 mL/min).

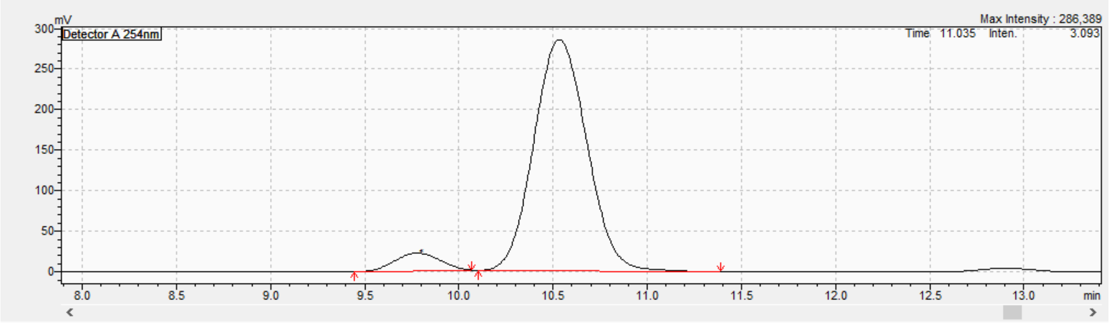

Results View - Peak Table

Peak Table | Compound | Group | Calibration Curve

Peak#	Ret. Time	Area	Height	Mark	Conc.	Unit	ID#	Name	Area%
1	9.779	365085	21823	M	6.155				6.155
2	10.533	5566643	285125	M	93.845				93.845
Total		5931728	306948		100.000				100.000

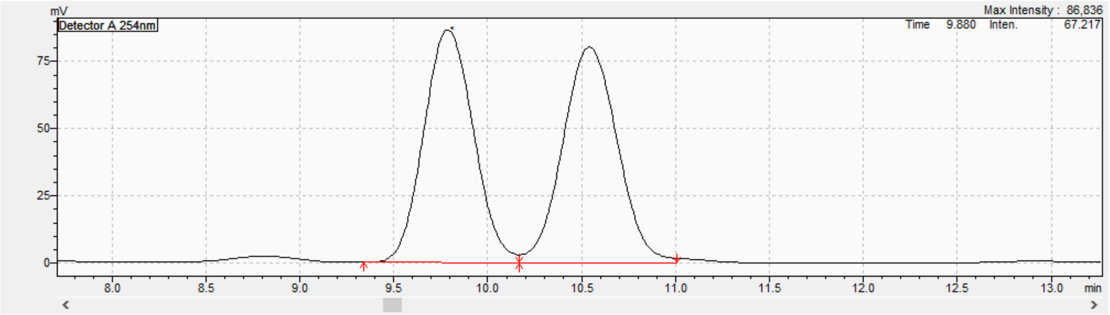

Results View - Peak Table

Peak Table | Compound | Group | Calibration Curve

Peak#	Ret. Time	Area	Height	Mark	Conc.	Unit	ID#	Name	Area%
1	9.787	1598285	86612		49.656				49.656
2	10.541	1620456	80211	V M	50.344				50.344
Total		3218741	166823		100.000				100.000

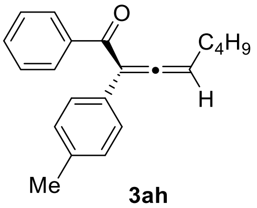

3ah

(*R*)-1-phenyl-2-(*p*-tolyl)octa-2,3-dien-1-one (3ah):

GP-B: 20.9 mg, 72% isolated yield; $[\alpha]_D^{25}$ = 51.1 (c 0.8, $CHCl_3$), light yellow oil, 1:30 ethyl acetate:hexanes as eluent, Rf = 0.3. ^{1}H NMR (400 MHz, $CDCl_3$) δ 7.90 (d, *J* = 6.8 Hz, 2H), 7.54 (t, *J* = 7.2 Hz, 1H), 7.43 (t, *J* = 7.6 Hz, 2H), 7.36 (d, *J* = 8.4 Hz, 2H), 7.17 (d, *J* = 8.0 Hz, 2H), 5.67 (t, *J* = 7.2 Hz, 1H), 2.36 (s, 3H), 2.23 – 2.10 (m, 2H), 1.44 – 1.33 (m, 2H), 1.31 – 1.20 (m, 2H), 0.84 (t, *J* = 7.2 Hz, 3H). ^{13}C NMR (100 MHz, $CDCl_3$) δ 210.8, 194.3, 138.7, 137.3, 132.4, 130.7, 129.4, 129.2, 128.0, 127.9, 108.8, 97.1, 31.1, 28.2, 22.1, 21.2, 13.7. HRMS (ESI): m/z calcd. for $C_{21}H_{23}O^+$($[M+H]^+$) = 291.1743, found = 291.1743; the ee value was 92%, t_R (minor) = 16.7 min, t_R (major) = 21.8 min (Chiralpak IC, λ = 254 nm, 1% *i*-PrOH/Hexane, flow rate = 1.0 mL/min).

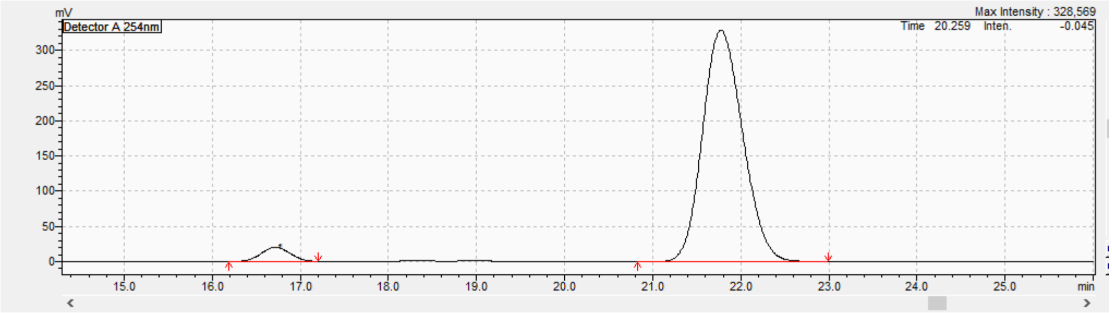

Results View - Peak Table

Peak Table | Compound | Group | Calibration Curve

Peak#	Ret. Time	Area	Height	Mark	Conc.	Unit	ID#	Name	Area%
1	16.718	460485	20212	M	4.243				4.243
2	21.774	10393417	328477	M	95.757				95.757
Total		10853902	348689		100.000				100.000

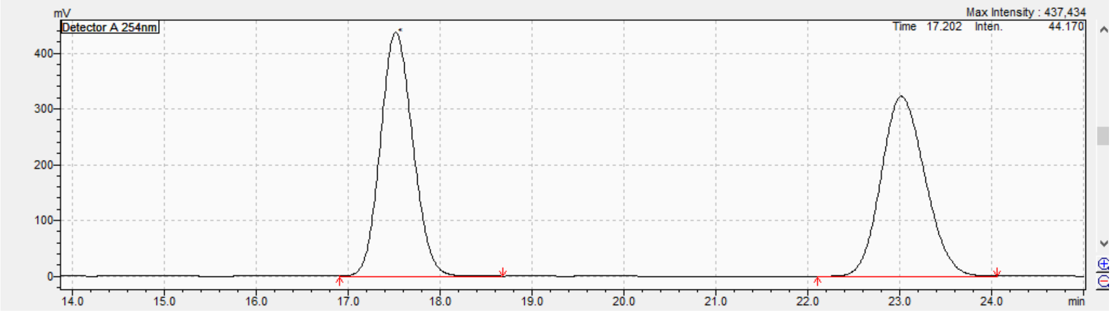

Results View - Peak Table

Peak Table | Compound | Group | Calibration Curve

Peak#	Ret. Time	Area	Height	Mark	Conc.	Unit	ID#	Name	Area%
1	17.519	10714432	437475		50.028				50.028
2	23.023	10702462	322560		49.972				49.972
Total		21416894	760035		100.000				100.000

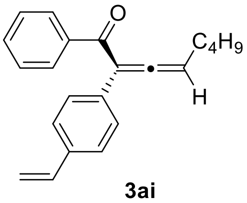

3ai

(*R*)-1-phenyl-2-(4-vinylphenyl)octa-2,3-dien-1-one (3ai):

GP-B: 16.0 mg, 53% isolated yield; $[\alpha]_D^{25}$ = 18.9 (c 0.7, $CHCl_3$), light yellow oil, 1:30 ethyl acetate:hexanes as eluent, Rf = 0.3. ^{1}H NMR (400 MHz, $CDCl_3$) δ 7.90 (d, *J* = 7.2 Hz, 2H), 7.55 (t, *J* = 8.0 Hz, 1H), 7.47 – 7.31 (m, 6H), 6.72 (dd, *J* = 22.4, 10.8 Hz, 1H), 5.76 (d, *J* = 17.2 Hz, 1H), 5.70 (t, *J* = 7.2 Hz, 1H), 5.25 (d, *J* = 10.8 Hz, 1H), 2.26 – 2.11 (m, 2H), 1.44 – 1.35 (m, 2H), 1.31 – 1.20 (m, 2H), 0.84 (t, *J* = 7.2 Hz, 3H). ^{13}C NMR (100 MHz, $CDCl_3$) δ 210.9, 194.1, 138.6, 136.8, 136.4, 133.1, 132.6, 129.4, 128.1, 128.1, 126.3, 114.0, 108.7, 97.5, 31.1, 28.2, 22.1, 13.7. HRMS (ESI): m/z calcd. for $C_{22}H_{23}O^+([M+H]^+)$ = 303.1743, found = 303.1744; the ee value was 90%, t_R (minor) = 14.5 min, t_R (major) = 23.0 min (Chiralpak IC, λ = 254 nm, 1% *i*-PrOH/Hexane, flow rate = 1.0 mL/min).

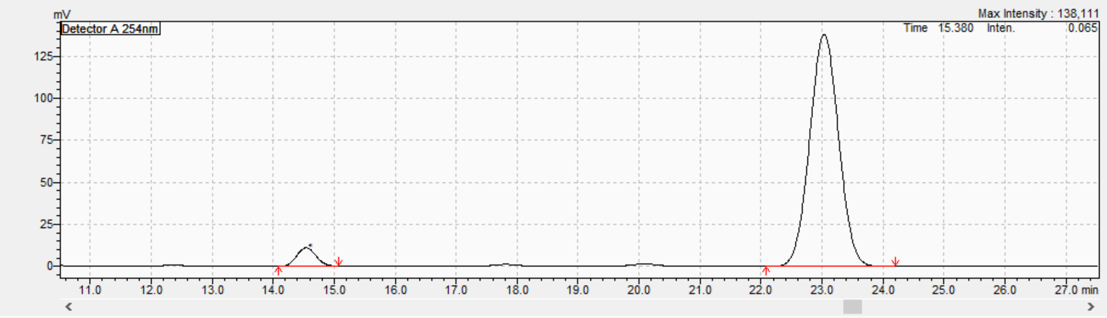

Results View - Peak Table

Peak Table | Compound | Group | Calibration Curve

Peak#	Ret. Time	Area	Height	Mark	Conc.	Unit	ID#	Name	Area%
1	14.540	241735	10944	M	5.044				5.044
2	23.033	4551004	138145		94.956				94.956
Total		4792739	149089		100.000				100.000

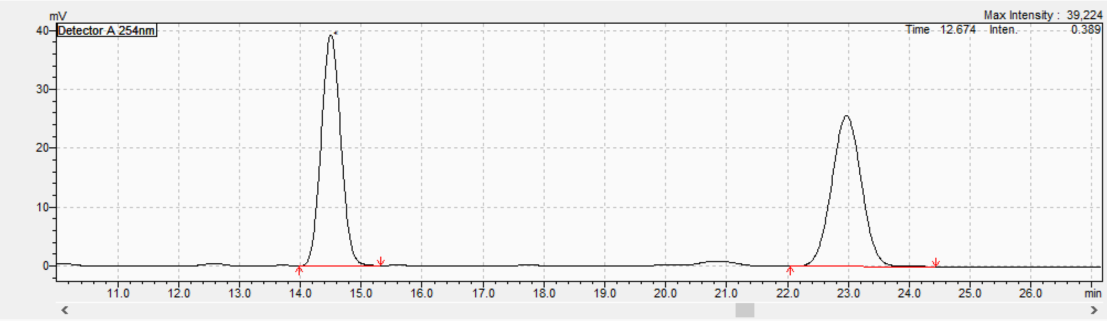

Results View - Peak Table

Peak Table | Compound | Group | Calibration Curve

Peak#	Ret. Time	Area	Height	Mark	Conc.	Unit	ID#	Name	Area%
1	14.501	879607	39254		50.309				50.309
2	22.971	868796	25581		49.691				49.691
Total		1748403	64835		100.000				100.000

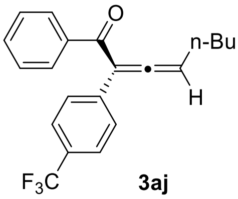

(*R*)-1-phenyl-2-(4-(trifluoromethyl)phenyl)octa-2,3-dien-1-one (3aj):

GP-B: 16.0 mg, 47% isolated yield; $[\alpha]_D^{25}$ = 4.0 (c 0.8, $CHCl_3$), light yellow oil, 1:20 ethyl acetate:hexanes as eluent, Rf = 0.3. ^{1}H NMR (600 MHz, $CDCl_3$) δ 7.91 (d, *J* = 7.2 Hz, 2H), 7.71 – 7.56 (m, 5H), 7.52 – 7.40 (m, 2H), 5.78 (t, *J* = 7.2 Hz, 1H), 2.28 – 2.18 (m, 2H), 1.44 – 1.34 (m, 2H), 1.34 – 1.25 (m, 2H), 0.87 (t, *J* = 7.2 Hz, 3H). ^{13}C NMR (150 MHz, $CDCl_3$) δ 211.8, 193.5, 138.3, 137.5, 132.8, 129.4 (q, *J* = 32.9 Hz), 129.3, 128.3, 128.2, 125.4 (q, *J* = 3.6 Hz), 124.2 (q, *J* = 272.4 Hz), 108.0, 97.9, 31.0, 28.1, 22.1, 13.7. ^{19}F NMR (471 MHz, $CDCl_3$) δ -62.6. HRMS (APCI): m/z calcd. for $C_{21}H_{20}F_3O^+$($[M+H]^+$) = 345.1461, found = 345.1462; the ee value was 51%, t_R (minor) = 6.1 min, t_R (major) = 7.1 min (Chiralcel® OJ-H, λ = 254 nm, 2% *i*-PrOH/Hexane, flow rate = 1.0 mL/min).

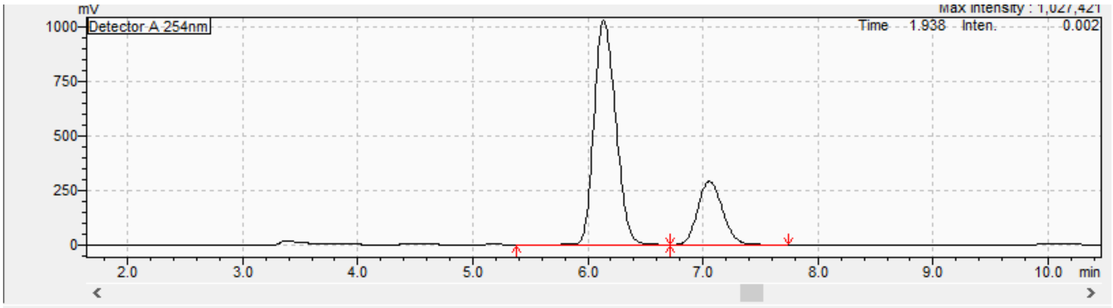

Results View - Peak Table

Peak Table | Compound | Group | Calibration Curve

Peak#	Ret. Time	Area	Height	Mark	Conc.	Area%
1	6.137	13528431	1027527		75.690	75.690
2	7.056	4345139	289629		24.310	24.310
Total		17873570	1317156		100.000	100.000

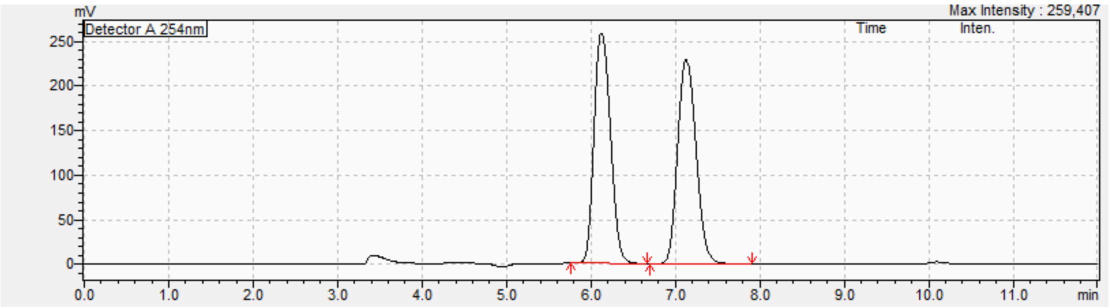

Results View - Peak Table

Peak Table | Compound | Group | Calibration Curve

Peak#	Ret. Time	Area	Height	Mark	Conc.	Area%
1	6.125	3490620	258317		49.994	49.994
2	7.129	3491483	228548		50.006	50.006
Total		6982103	486866		100.000	100.000

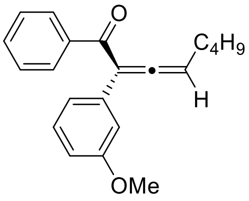

3ak

(*R*)-2-(3-methoxyphenyl)-1-phenylocta-2,3-dien-1-one (3ak):

GP-B: 19.0 mg, 62% isolated yield; $[\alpha]_D^{25}$ = 45.0 (c 0.8, $CHCl_3$), light yellow oil, 1:15 ethyl acetate:hexanes as eluent, Rf = 0.3. ^{1}H NMR (400 MHz, $CDCl_3$) δ 7.91 (d, *J* = 6.8 Hz, 2H), 7.55 (t, *J* = 7.2 Hz, 1H), 7.43 (t, *J* = 7.6 Hz, 2H), 7.31 – 7.25 (m, 1H), 7.09 – 7.03 (m, 2H), 6.88 – 6.79 (m, 1H), 5.69 (t, *J* = 7.2 Hz, 1H), 3.81 (s, 3H), 2.24 – 2.11 (m, 2H), 1.44 – 1.34 (m, 2H), 1.30 – 1.20 (m, 2H), 0.84 (t, *J* = 7.2 Hz, 3H). ^{13}C NMR (100 MHz, $CDCl_3$) δ 210.8, 194.0, 159.6, 138.6, 135.0, 132.6, 129.4, 128.1, 120.5, 113.4, 113.3, 108.8, 97.4, 55.2, 31.1, 28.2, 22.1, 13.7. HRMS (ESI): m/z calcd. for $C_{21}H_{23}O_2^+$([M+H]$^+$) = 307.1693, found = 307.1695; the ee value was 95%, t_R (minor) = 20.1 min, t_R (major) = 22.6 min (Chiralpak IC, λ = 254 nm, 2% *i*-PrOH/Hexane, flow rate = 1.0 mL/min).

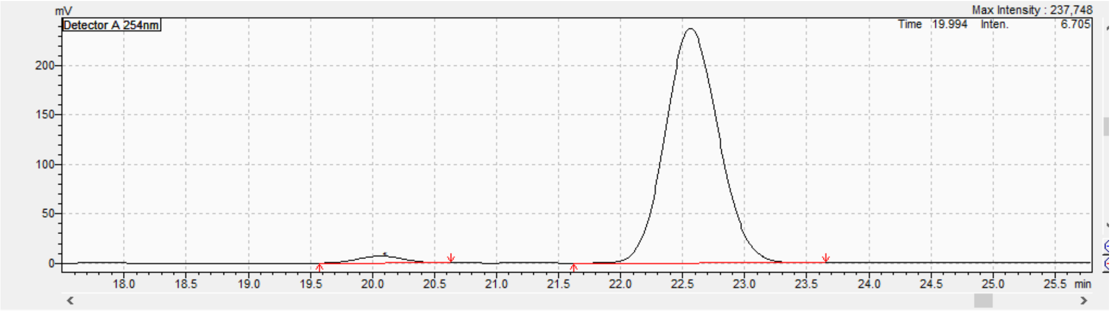

Results View - Peak Table

Peak Table | Compound | Group | Calibration Curve

Peak#	Ret. Time	Area	Height	Mark	Conc.	Unit	ID#	Name	Area%
1	20.064	182833	7071	M	2.513				2.513
2	22.564	7092731	237601		97.487				97.487
Total		7275564	244672		100.000				100.000

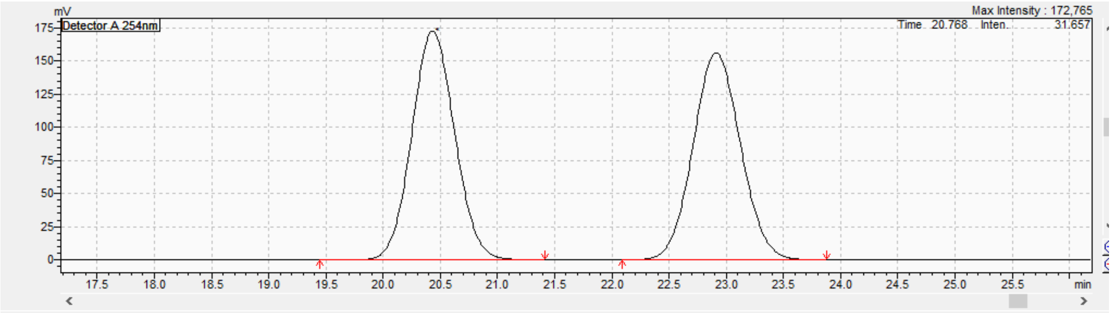

Results View - Peak Table

Peak Table | Compound | Group | Calibration Curve

Peak#	Ret. Time	Area	Height	Mark	Conc.	Unit	ID#	Name	Area%
1	20.433	4518432	172767		50.005				50.005
2	22.911	4517532	156162		49.995				49.995
Total		9035964	328929		100.000				100.000

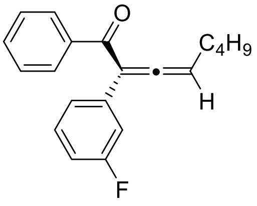

3al

(*R*)-2-(3-fluorophenyl)-1-phenylocta-2,3-dien-1-one (3al):

GP-B: 16.2 mg, 55% isolated yield; $[\alpha]_D^{25}$ = 6.0 (c 0.6, $CHCl_3$), light yellow oil, 1:30 ethyl acetate:hexanes as eluent, Rf = 0.3. ^{1}H NMR (400 MHz, $CDCl_3$) δ 7.89 (d, *J* = 7.2 Hz, 2H), 7.55 (t, *J* = 7.2 Hz, 1H), 7.44 (t, *J* = 8.0 Hz, 2H), 7.35 – 7.19 (m, 3H), 7.00 – 6.95 (m, 1H), 5.72 (t, *J* = 7.2 Hz, 1H), 2.31 – 2.01 (m, 2H), 1.51 – 1.32 (m, 2H), 1.30 – 1.17 (m, 2H), 0.84 (t, *J* = 7.2 Hz, 3H). ^{13}C NMR (100 MHz, $CDCl_3$) δ 211.2, 193.6, 162.9 (d, *J* = 245.1 Hz), 138.4, 135.9 (d, *J* = 8.0 Hz), 132.7, 129.8 (d, *J* = 8.4 Hz), 129.3, 128.1, 123.6 (d, *J* = 2.9 Hz), 114.9 (d, *J* = 23.0 Hz), 114.4 (d, *J* = 21.2 Hz), 108.1 (d, *J* = 2.5 Hz), 97.8, 31.0, 28.1, 22.1, 13.7. ^{19}F NMR (471 MHz, $CDCl_3$) δ -113.2. HRMS (ESI): m/z calcd. for $C_{20}H_{20}FO^+$($[M+H]^+$) = 295.1493, found = 295.1495; the ee value was 83%, t_R (minor) = 12.9 min, t_R (major) = 8.3 min (Chiralcel® OJ-H, λ = 254 nm, 2% *i*-PrOH/Hexane, flow rate = 1.0 mL/min).

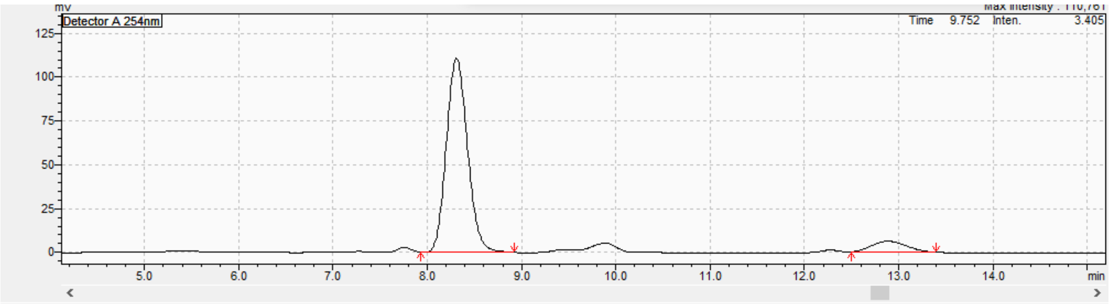

Results View - Peak Table

Peak Table | Compound | Group | Calibration Curve

Peak#	Ret. Time	Area	Height	Mark	Conc.	Unit	ID#	Name	Area%
1	8.309	1727303	110666	M	91.758				91.758
2	12.886	155150	6087	M	8.242				8.242
Total		1882453	116753		100.000				100.000

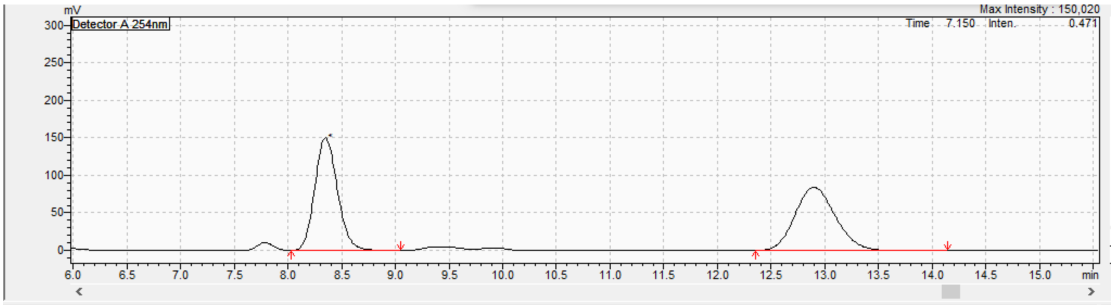

Results View - Peak Table

Peak Table | Compound | Group | Calibration Curve

Peak#	Ret. Time	Area	Height	Mark	Conc.	Unit	ID#	Name	Area%
1	8.349	2219581	150377		50.077				50.077
2	12.898	2212788	84554		49.923				49.923
Total		4432369	234930		100.000				100.000

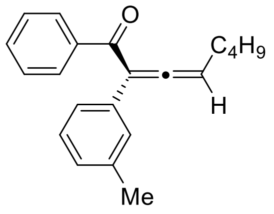

3am

(*R*)-1-phenyl-2-(*m*-tolyl)octa-2,3-dien-1-one (3am):

GP-B: 20.0 mg, 69% isolated yield; $[\alpha]_D^{25}$ = 13.7 (c 0.6, $CHCl_3$), light yellow oil, 1:30 ethyl acetate:hexanes as eluent, Rf = 0.3. ^{1}H NMR (400 MHz, $CDCl_3$) δ 7.83 (d, *J* = 7.2 Hz, 2H), 7.47 (t, *J* = 7.2 Hz, 1H), 7.36 (t, *J* = 7.6 Hz, 2H), 7.23 – 7.17 (m, 3H), 7.07 – 6.97 (m, 1H), 5.60 (t, *J* = 7.2 Hz, 1H), 2.28 (s, 3H), 2.20 – 2.01 (m, 2H), 1.37 – 1.26 (m, 2H), 1.25 – 1.09 (m, 2H), 0.77 (t, *J* = 7.2 Hz, 3H). ^{13}C NMR (100 MHz, $CDCl_3$) δ 210.8, 194.2, 138.6, 138.0, 133.6, 132.5, 129.4, 128.6, 128.4, 128.3, 128.0, 125.1, 109.0, 97.2, 31.1, 28.2, 22.1, 21.5, 13.7. HRMS (ESI): m/z calcd. for $C_{21}H_{23}O^+$([M+H]$^+$) = 291.1743, found = 291.1746; the ee value was 90%, t_R (minor) = 22.3 min, t_R (major) = 18.3 min (Chiralcel® OJ-H, λ = 254 nm, 2% *i*-PrOH/Hexane, flow rate = 1.0 mL/min).

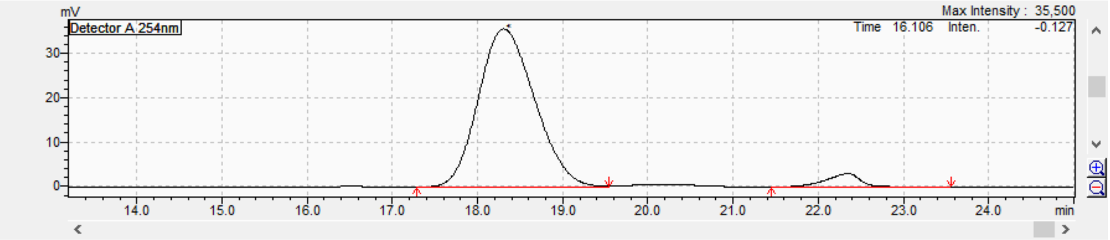

Results View - Peak Table

Peak Table | Compound | Group | Calibration Curve

Peak#	Ret. Time	Area	Height	Mark	Conc.	Unit	ID#	Name	Area%
1	18.307	1639139	35644		94.940				94.940
2	22.348	87354	3041		5.060				5.060
Total		1726493	38685		100.000				100.000

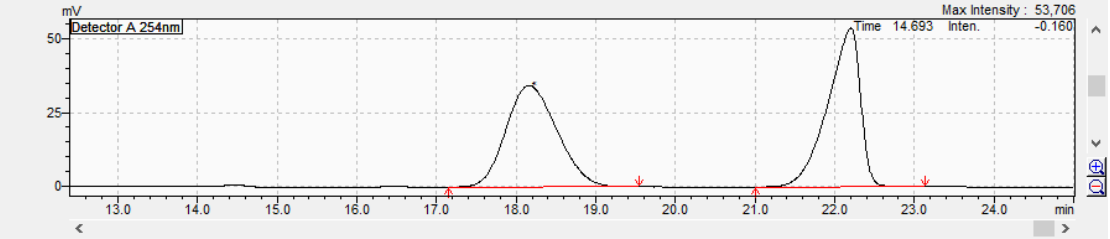

Results View - Peak Table

Peak Table | Compound | Group | Calibration Curve

Peak#	Ret. Time	Area	Height	Mark	Conc.	Unit	ID#	Name	Area%
1	18.160	1537226	34466		50.020				50.020
2	22.201	1535972	53889		49.980				49.980
Total		3073198	88356		100.000				100.000

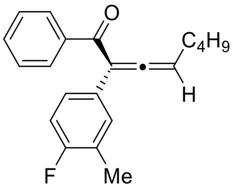

3an

(*R*)-2-(4-fluoro-3-methylphenyl)-1-phenylocta-2,3-dien-1-one (3an):

GP-B: 21.6 mg, 70% isolated yield; $[\alpha]_D^{25}$ = 6.4 (c 0.5, $CHCl_3$), light yellow oil, 1:30 ethyl acetate:hexanes as eluent, Rf = 0.3. ^1H NMR (400 MHz, $CDCl_3$) δ 7.88 (d, *J* = 7.2 Hz, 2H), 7.55 (t, *J* = 7.2 Hz, 1H), 7.43 (t, *J* = 7.6 Hz, 2H), 7.35 – 7.23 (m, 2H), 7.02 – 6.93 (m, 1H), 5.67 (t, *J* = 7.2 Hz, 1H), 2.27 (d, *J* = 2.0 Hz, 3H), 2.22 – 2.11 (m, 2H), 1.44 – 1.34 (m, 2H), 1.30 – 1.20 (m, 2H), 0.84 (t, *J* = 7.2 Hz, 3H). ^{13}C NMR (100 MHz, $CDCl_3$) δ 211.1, 194.2, 160.8 (d, *J* = 245.8 Hz), 138.6, 132.6, 131.1 (d, *J* = 5.2 Hz), 129.3, 129.2 (d, *J* = 3.7 Hz), 128.0, 127.1 (d, *J* = 8.1 Hz), 124.8 (d, *J* = 17.6 Hz), 115.0 (d, *J* = 22.6 Hz), 108.2, 97.3, 31.0, 28.2, 22.1, 14.6 (d, *J* = 3.0 Hz), 13.7. ^{19}F NMR (471 MHz, $CDCl_3$) δ -118.9. HRMS (ESI): m/z calcd. for $C_{21}H_{22}FO^+$($[M+H]^+$) = 309.1649, found = 309.1646; the ee value was 91%, t_R (minor) = 14.3 min, t_R (major) = 9.8 min (Chiralcel® OJ-H, λ = 254 nm, 1% *i*-PrOH/Hexane, flow rate = 1.0 mL/min).

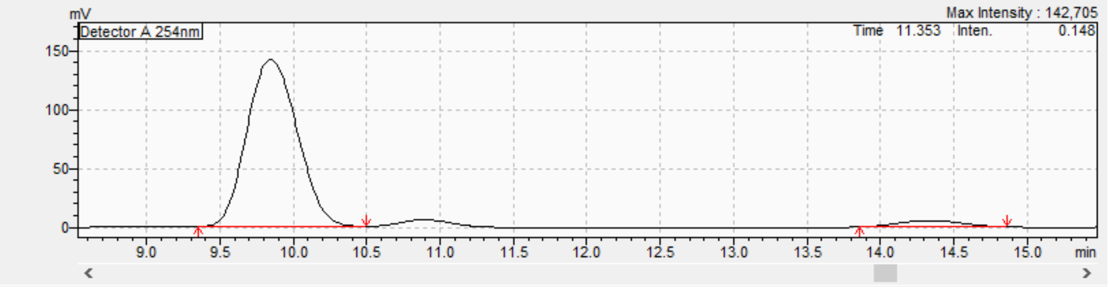

Results View - Peak Table

Peak Table | Compound | Group | Calibration Curve

Peak#	Ret. Time	Area	Height	Mark	Conc.	Area%
1	9.845	3376870	141810		95.306	95.306
2	14.335	166325	5519	M	4.694	4.694
Total		3543195	147329		100.000	100.000

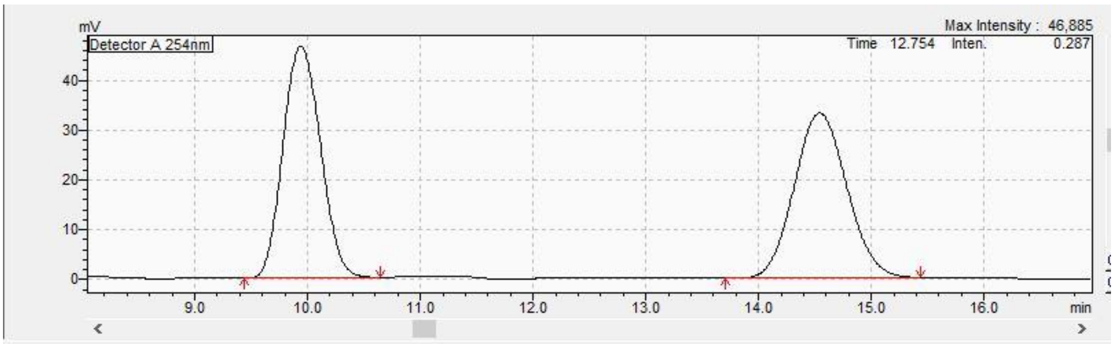

Results View - Peak Table

Peak Table | Compound | Group | Calibration Curve

Peak#	Ret. Time	Area	Height	Mark	Conc.	Unit	Area%
1	9.939	1078734	46577		50.019		50.019
2	14.543	1077921	33169	M	49.981		49.981
Total		2156655	79746		100.000		100.000

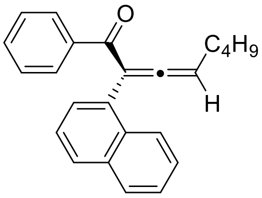

3ao

(*R*)-2-(naphthalen-1-yl)-1-phenylocta-2,3-dien-1-one (3ao):

GP-B: 17.9 mg, 55% isolated yield; $[\alpha]_D^{25}$ = 21.2 (c 0.8, $CHCl_3$), light yellow oil, 1:30 ethyl acetate:hexanes as eluent, Rf = 0.3. ^{1}H NMR (400 MHz, $CDCl_3$) δ 7.93 (d, J = 7.2 Hz, 2H), 7.84 – 7.75 (m, 3H), 7.50 (t, J = 7.6 Hz, 1H), 7.46 – 7.33 (m, 6H), 5.52 (t, J = 7.2 Hz, 1H), 2.17 – 2.05 (m, 2H), 1.36 – 1.26 (m, 2H), 1.20 – 1.10 (m, 2H), 0.74 (t, J = 7.2 Hz, 3H). ^{13}C NMR (100 MHz, $CDCl_3$) δ 213.5, 194.1, 138.3, 133.9, 132.3, 132.3, 131.6, 129.3, 128.5, 128.5, 128.1, 127.9, 126.2, 125.7, 125.5, 125.2, 107.8, 94.8, 31.0, 28.1, 22.1, 13.7. HRMS (ESI): m/z calcd. for $C_{24}H_{23}O^+$($[M+H]^+$) = 327.1743, found = 327.1748; the ee value was 90%, t_R (minor) = 10.1 min, t_R (major) = 15.8 min (Chiralcel® OJ-H, λ = 254 nm, 10% *i*-PrOH/Hexane, flow rate = 1.0 mL/min)

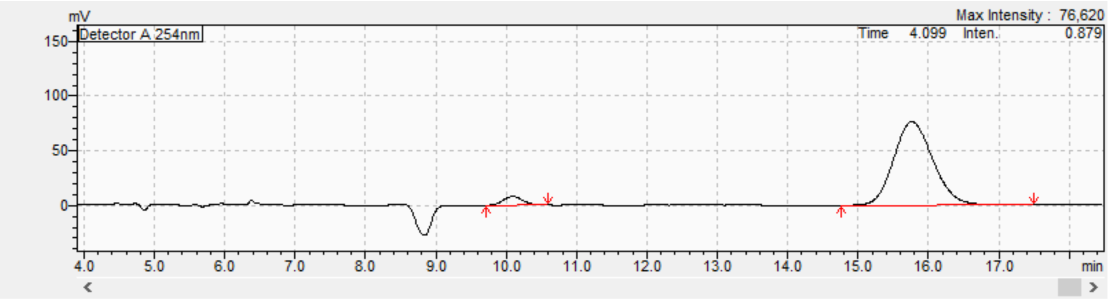

Results View - Peak Table

Peak Table | Compound | Group | Calibration Curve

Peak#	Ret. Time	Area	Height	Mark	Conc.	Area%
1	10.089	157676	7968	M	5.070	5.0
2	15.764	2952367	76333		94.930	94.9
Total		3110043	84301		100.000	100.0

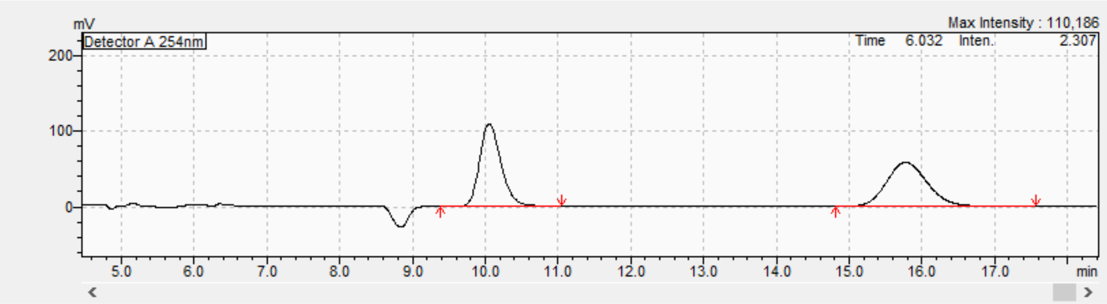

Results View - Peak Table

Peak Table | Compound | Group | Calibration Curve

Peak#	Ret. Time	Area	Height	Mark	Conc.	Area%
1	10.056	2244951	109470	M	49.856	49.8
2	15.775	2257902	58089		50.144	50.1
Total		4502853	167559		100.000	100.0

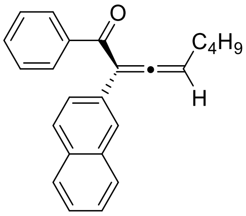

3ap

(*R*)-2-(naphthalen-2-yl)-1-phenylocta-2,3-dien-1-one (3ap):

GP-B: 20.5 mg, 63% isolated yield; $[\alpha]_D^{25}$ = 15.1 (c 0.5, $CHCl_3$), light yellow oil, 1:30 ethyl acetate:hexanes as eluent, Rf = 0.3. ^{1}H NMR (400 MHz, $CDCl_3$) δ 8.00 – 7.94 (m, 3H), 7.86 – 7.78 (m, 3H), 7.62 – 7.54 (m, 2H), 7.50 – 7.43 (m, 4H), 5.77 (t, *J* = 7.2 Hz, 1H), 2.32 – 2.13 (m, 2H), 1.47 – 1.38 (m, 2H), 1.34 – 1.23 (m, 2H), 0.86 (t, *J* = 7.2 Hz, 3H). ^{13}C NMR (100 MHz, $CDCl_3$) δ 210.9, 194.2, 138.6, 133.4, 132.7, 132.7, 130.9, 129.5, 128.2, 128.1, 128.0, 127.5, 126.9, 126.1, 126.1, 125.8, 109.0, 97.7, 31.1, 28.2, 22.1, 13.8. HRMS (ESI): m/z calcd. for $C_{24}H_{23}O^+$([M+H]$^+$) = 327.1743, found = 327.1746; the ee value was 80%, t_R (minor) = 22.9 min, t_R (major) = 33.7 min (Chiralpak IC, λ = 254 nm, 1% *i*-PrOH/Hexane, flow rate = 1.0 mL/min)

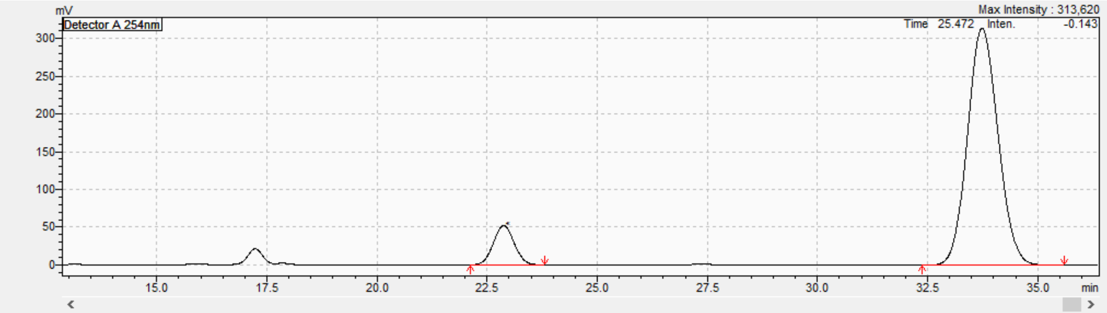

Results View - Peak Table

Peak Table | Compound | Group | Calibration Curve

Peak#	Ret. Time	Area	Height	Mark	Conc.	Unit	ID#	Name	Area%
1	22.877	1721133	52234	M	10.174				10.174
2	33.737	15196282	313967		89.826				89.826
Total		16917415	366200		100.000				100.000

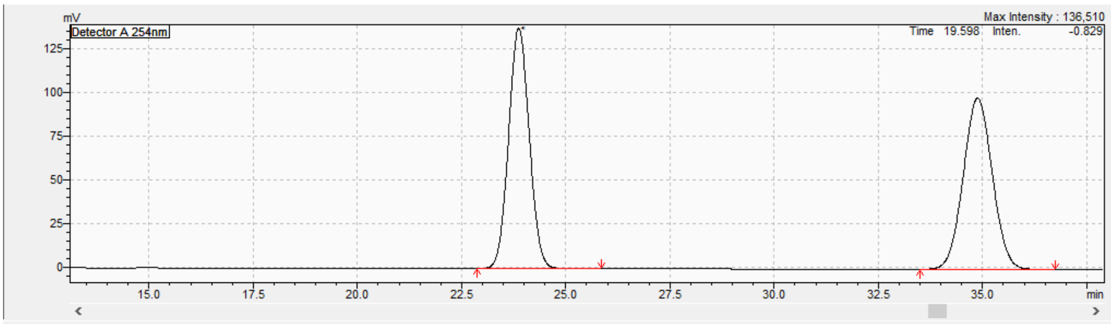

Results View - Peak Table

Peak Table | Compound | Group | Calibration Curve

Peak#	Ret. Time	Area	Height	Mark	Conc.	Unit	ID#	Name	Area%
1	23.870	4747789	137469		49.970				49.970
2	34.881	4753419	97837		50.030				50.030
Total		9501207	235305		100.000				100.000

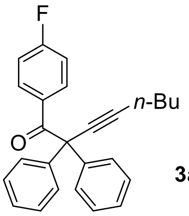

3a-1

1-(4-fluorophenyl)-2,2-diphenyloct-3-yn-1-one (3a-1):
^{1}H NMR (400 MHz, $CDCl_3$) δ 8.08 – 7.94 (m, 2H), 7.44 – 7.26 (m, 10H), 6.95 (t, *J* = 8.8 Hz, 2H), 2.29 (t, *J* = 7.0 Hz, 2H), 1.51 – 1.40 (m, 2H), 1.38 – 1.23 (m, 2H), 0.84 (t, *J* = 7.2 Hz, 3H). ^{13}C NMR (100 MHz, $CDCl_3$) δ 194.6, 165.0 (d, *J* = 254.6 Hz), 141.1, 133.6 (d, *J* = 9.1 Hz), 131.3 (d, *J* = 3.1 Hz), 128.6, 128.3, 127.4, 114.6 (d, *J* = 21.8 Hz), 91.7, 81.5, 61.5, 30.4, 21.9, 18.7, 13.5. ^{19}F NMR (377 MHz, $CDCl_3$) δ -106.0. HRMS (ESI): m/z calcd. for $C_{26}H_{24}FO^{+}$($[M+H]^{+}$) = 371.1806, found = 371.1805.

11. Copy of NMR Spectra

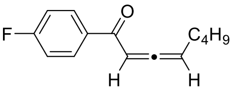

1a

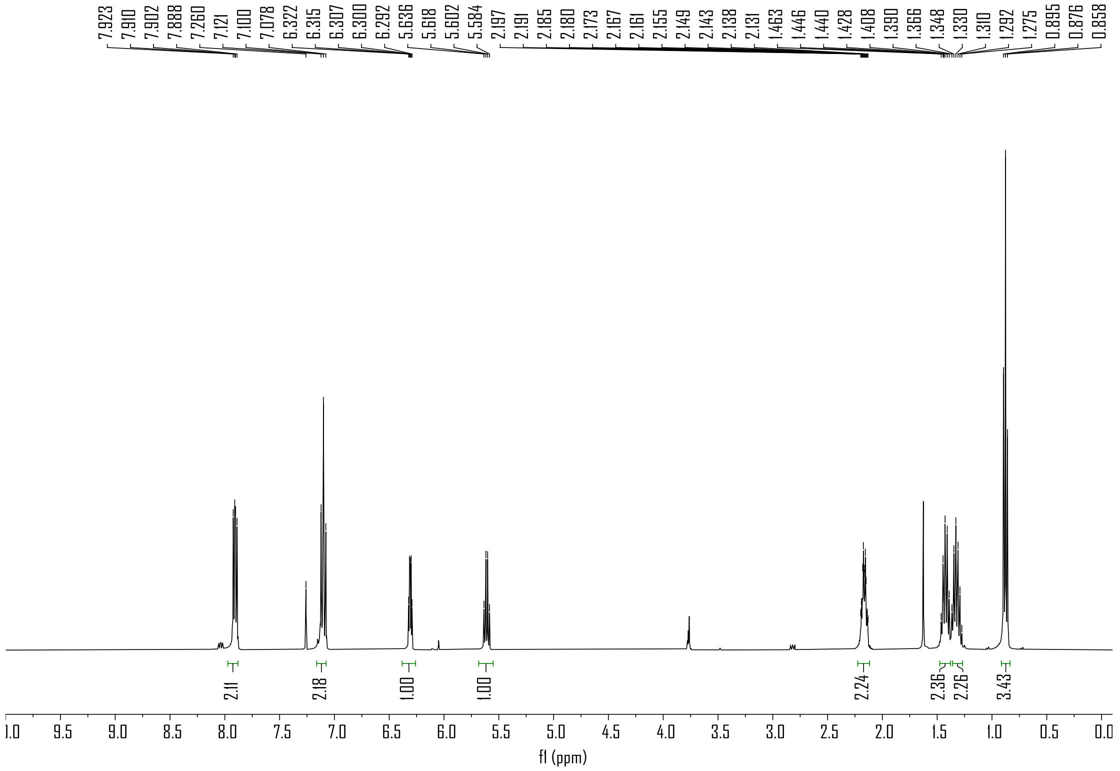

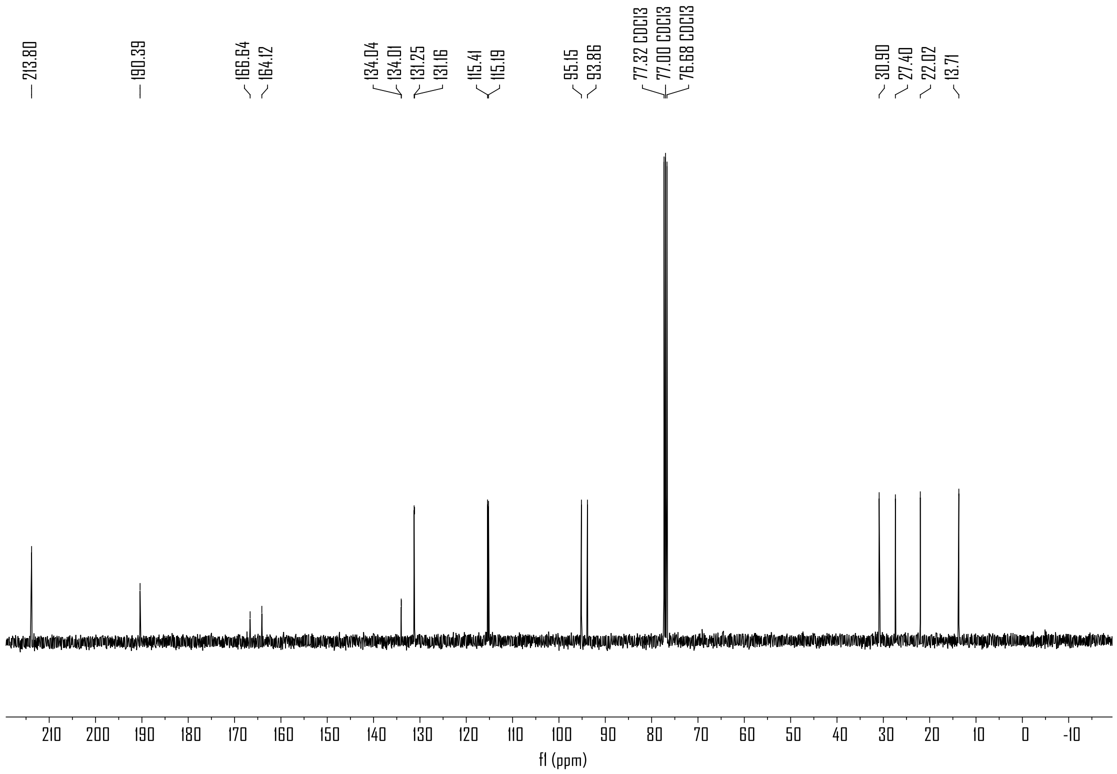
213.80
190.39
166.64
164.12
134.04
134.01
131.25
131.16
115.41
115.19
95.15
93.86
77.32 CDCl3
77.00 CDCl3
76.68 CDCl3
30.90
27.40
22.02
13.71
f1 (ppm)

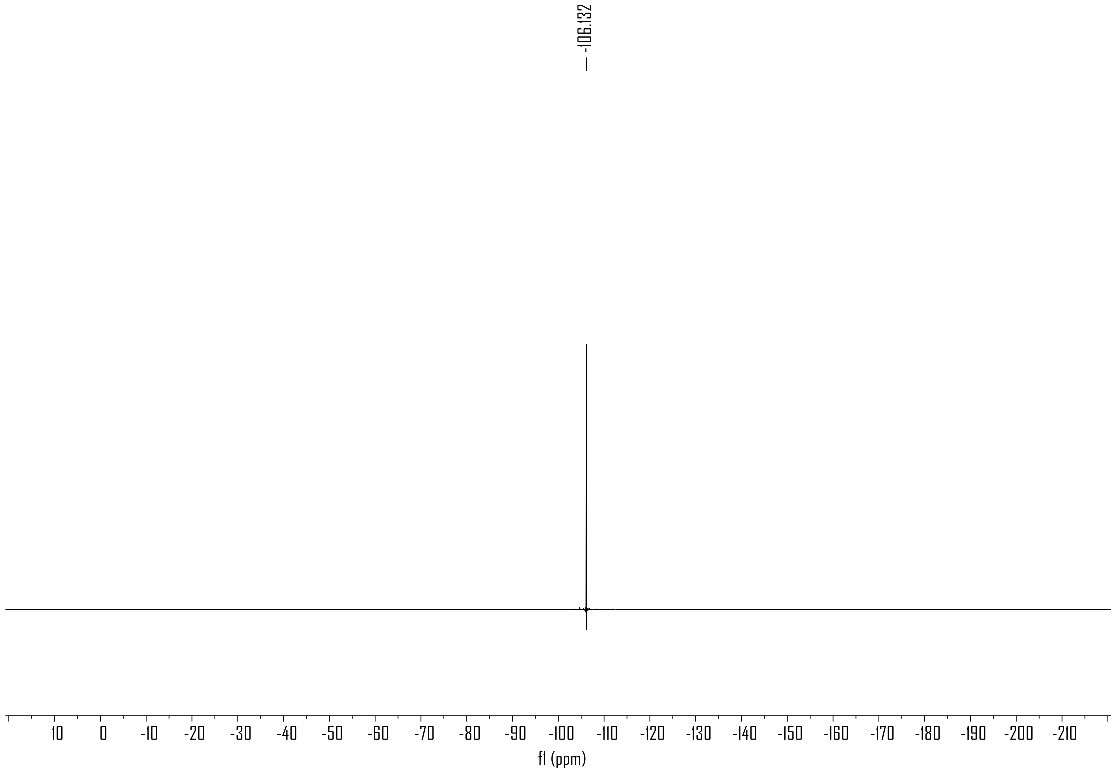
-106.132
f1 (ppm)

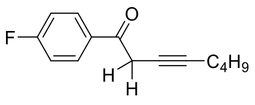
F
O
H
H
C4H9

1a'

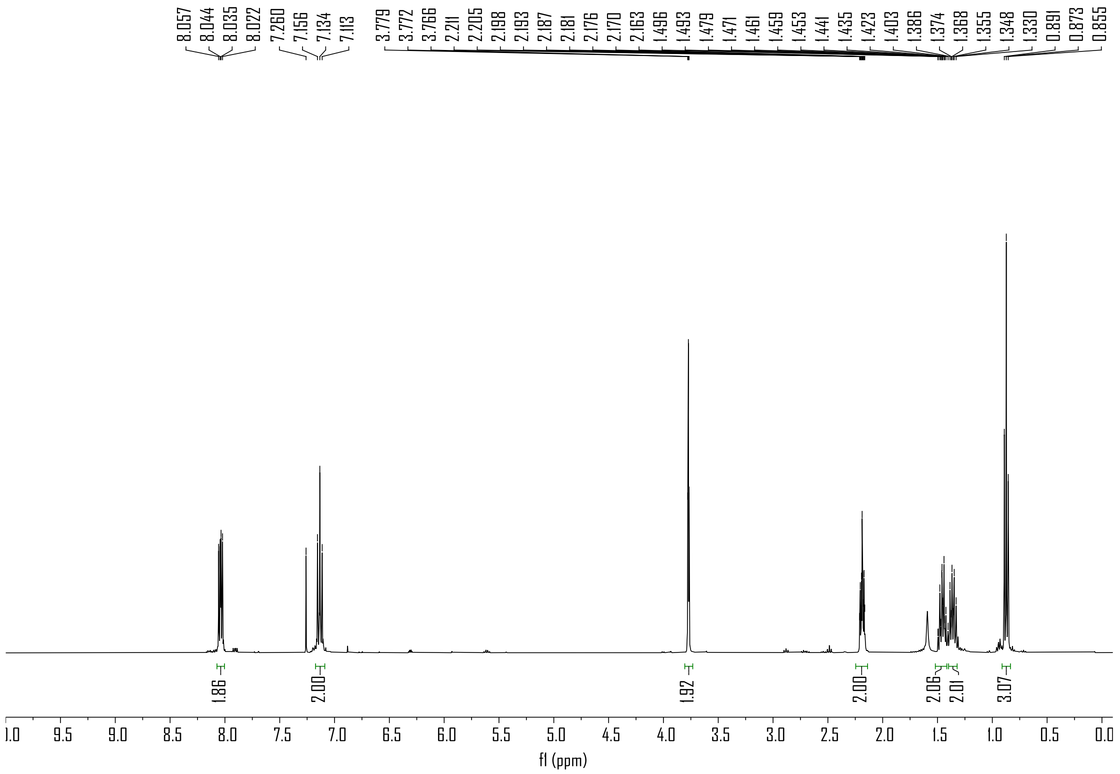
8.057
8.044
8.035
8.022
7.260
7.156
7.134
7.113
3.779
3.772
3.766
2.211
2.205
2.198
2.193
2.187
2.181
2.176
2.170
2.163
1.496
1.493
1.479
1.471
1.461
1.459
1.453
1.441
1.435
1.423
1.403
1.386
1.374
1.368
1.355
1.348
1.330
0.891
0.873
0.855
1.86
2.00
1.92
2.00
2.06
2.01
3.07
9.5
9.0
8.5
8.0
7.5
7.0
6.5
6.0
5.5
5.0
4.5
4.0
3.5
3.0
2.5
2.0
1.5
1.0
0.5
f1 (ppm)

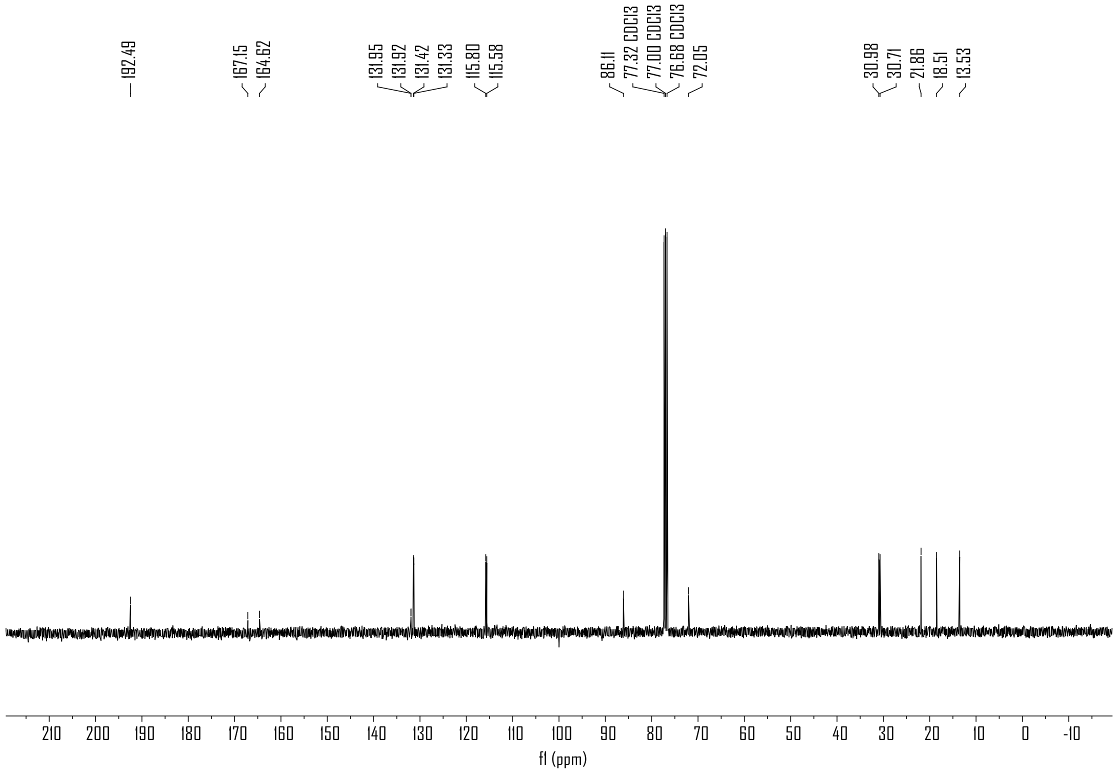
192.49
167.15
164.62
131.95
131.92
131.42
131.33
115.80
115.58
86.11
77.32 CDCl3
77.00 CDCl3
76.68 CDCl3
72.05
30.98
30.71
21.86
18.51
13.53
210
200
190
180
170
160
150
140
130
120
110
100
90
80
70
60
50
40
30
20
10
0
-10
f1 (ppm)

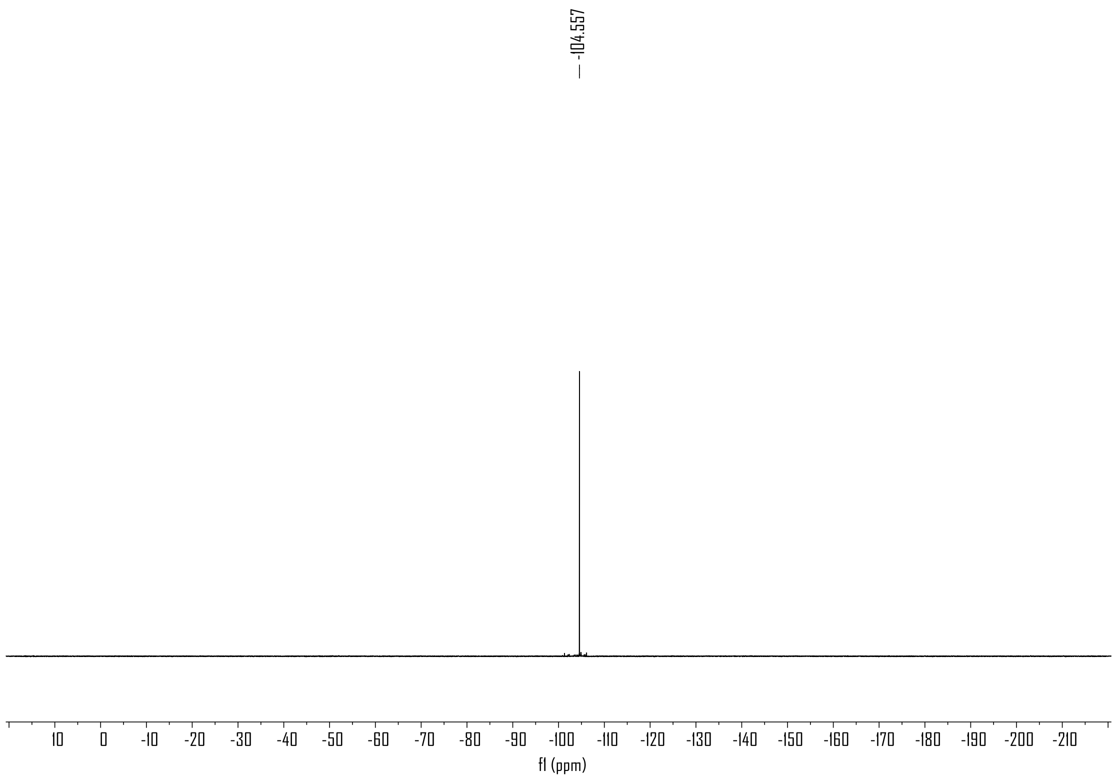
—-104.557
10 0 -10 -20 -30 -40 -50 -60 -70 -80 -90 -100 -110 -120 -130 -140 -150 -160 -170 -180 -190 -200 -210
f1 (ppm)

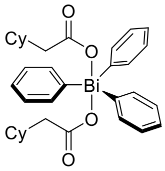
Cy
O
O
Bi
O
Cy
O

2ah'

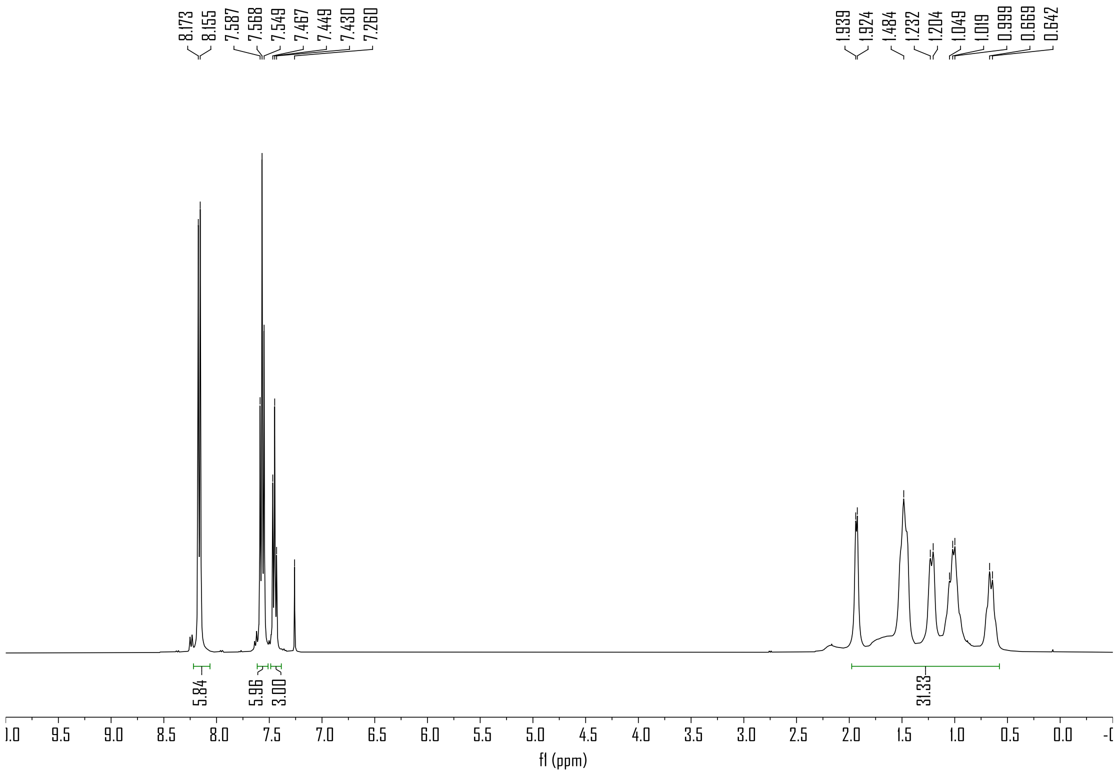
8.173
8.155
7.587
7.568
7.549
7.467
7.449
7.430
7.260
1.939
1.924
1.484
1.232
1.204
1.049
1.019
0.999
0.669
0.642
5.84
5.96
3.00
31.33
f1 (ppm)

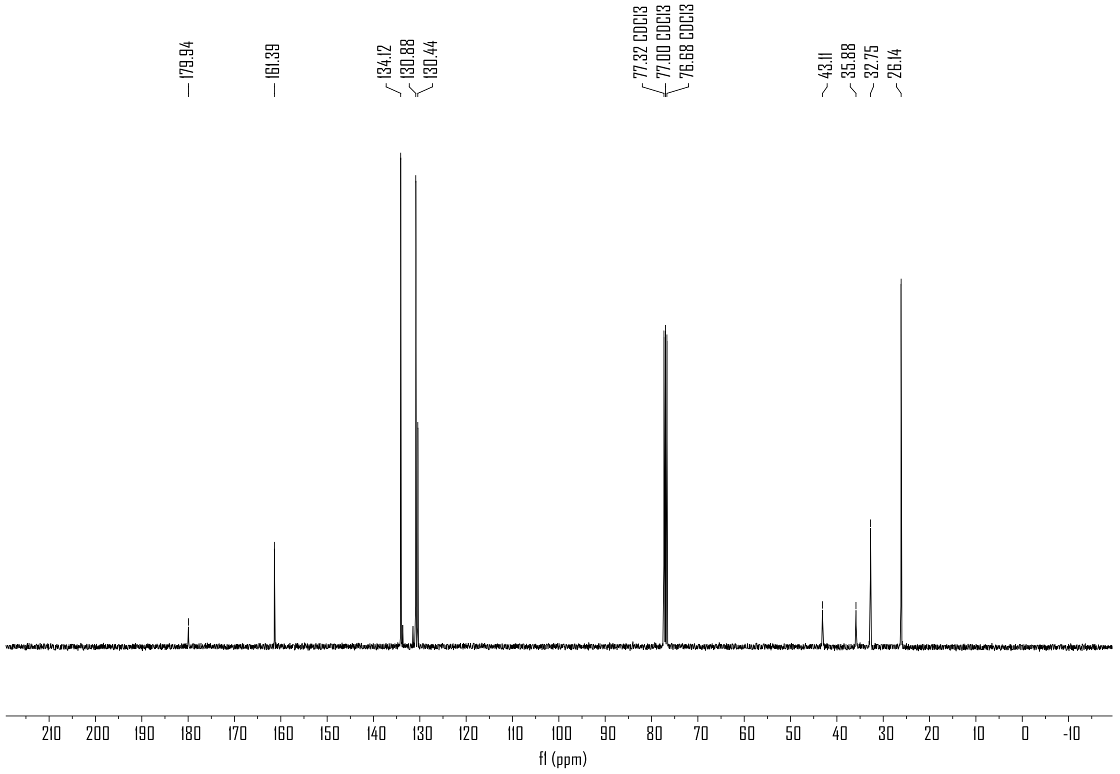
179.94
161.39
134.12
130.88
130.44
77.32 CDCl3
77.00 CDCl3
76.68 CDCl3
43.11
35.88
32.75
26.14
f1 (ppm)

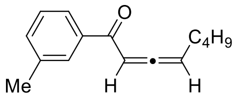
O
C4H9
Me
H
H

1g

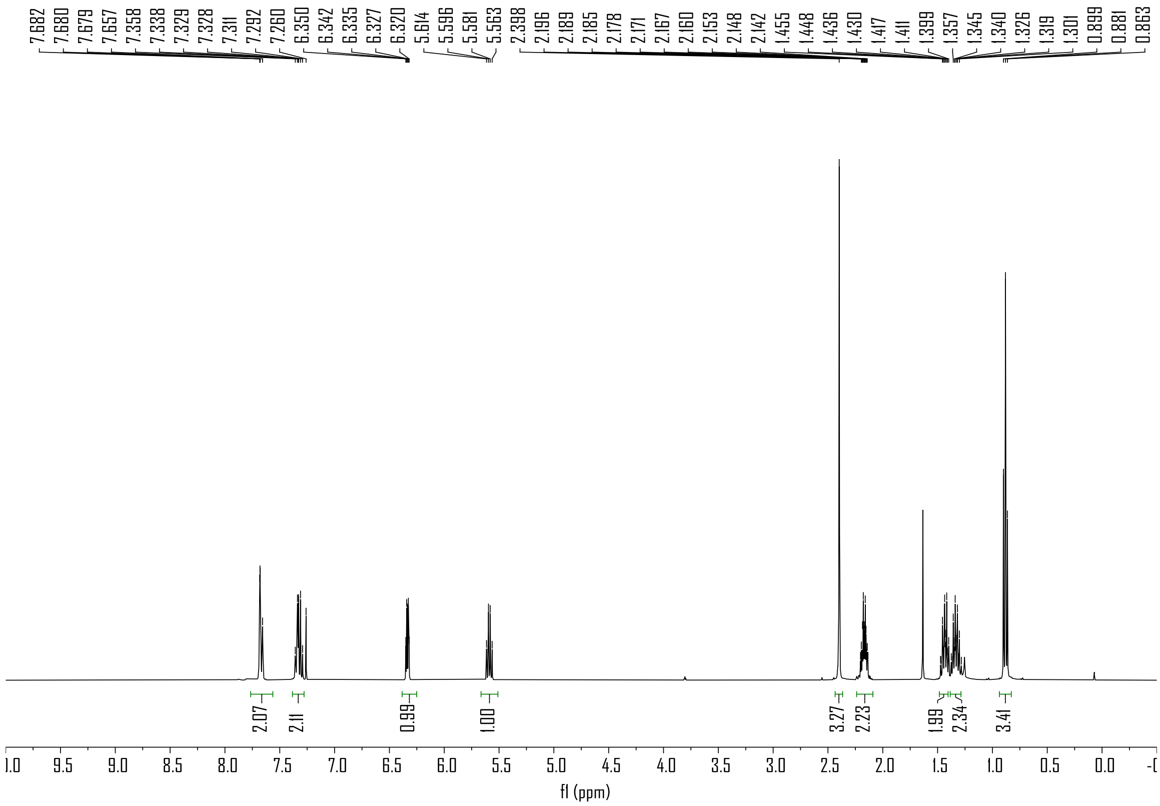
7.682 7.680 7.679 7.657 7.358 7.338 7.329 7.328 7.311 7.292 7.260 6.350 6.342 6.335 6.327 6.320 5.614 5.596 5.581 5.563 2.398 2.196 2.189 2.185 2.178 2.171 2.167 2.160 2.153 2.148 2.142 1.455 1.448 1.436 1.430 1.417 1.411 1.399 1.357 1.345 1.340 1.326 1.319 1.301 0.899 0.881 0.863
2.07 2.11 0.99 1.00 3.27 2.23 1.99 2.34 3.41
9.5 9.0 8.5 8.0 7.5 7.0 6.5 6.0 5.5 5.0 4.5 4.0 3.5 3.0 2.5 2.0 1.5 1.0 0.5 0.0
f1 (ppm)

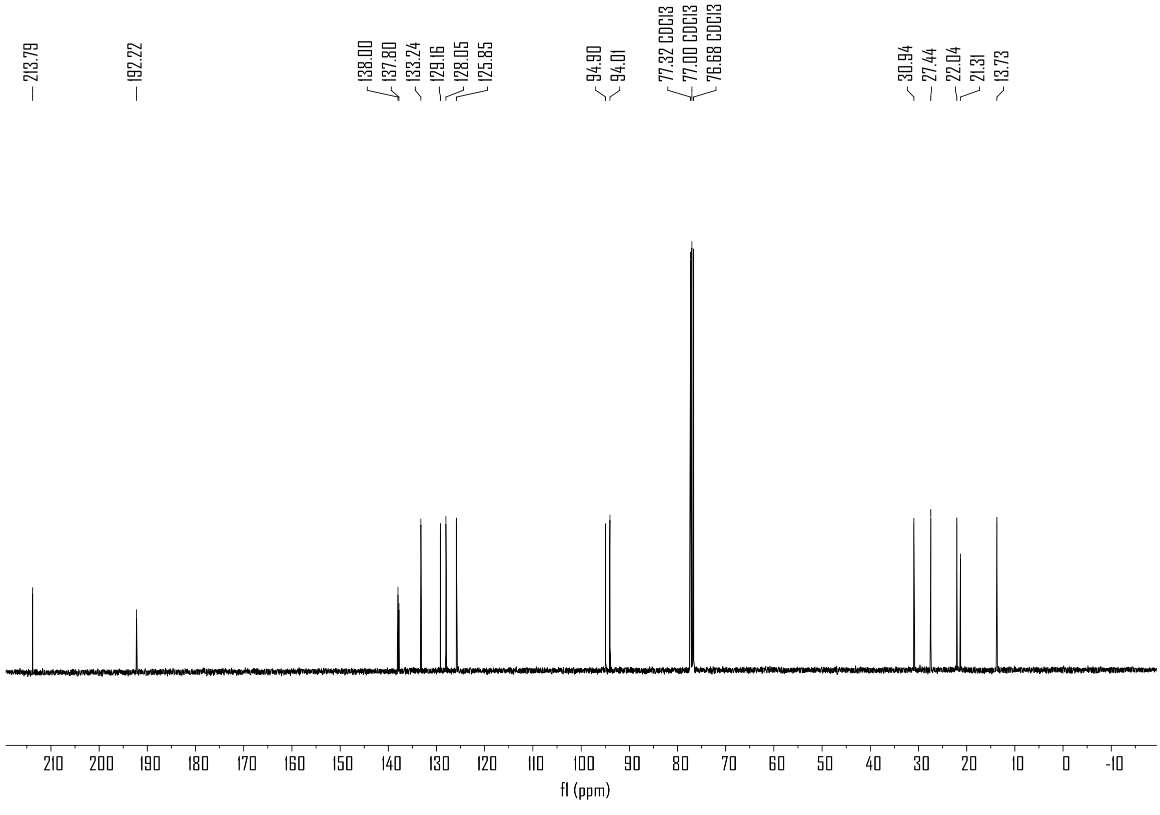
213.79
192.22
138.00 137.80 133.24 129.16 128.05 125.85
94.90 94.01
77.32 CDCl3 77.00 CDCl3 76.68 CDCl3
30.94 27.44 22.04 21.31 13.73
210 200 190 180 170 160 150 140 130 120 110 100 90 80 70 60 50 40 30 20 10 0 -10
f1 (ppm)

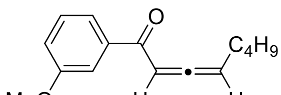
O
C4H9
MeO
H
H

1h

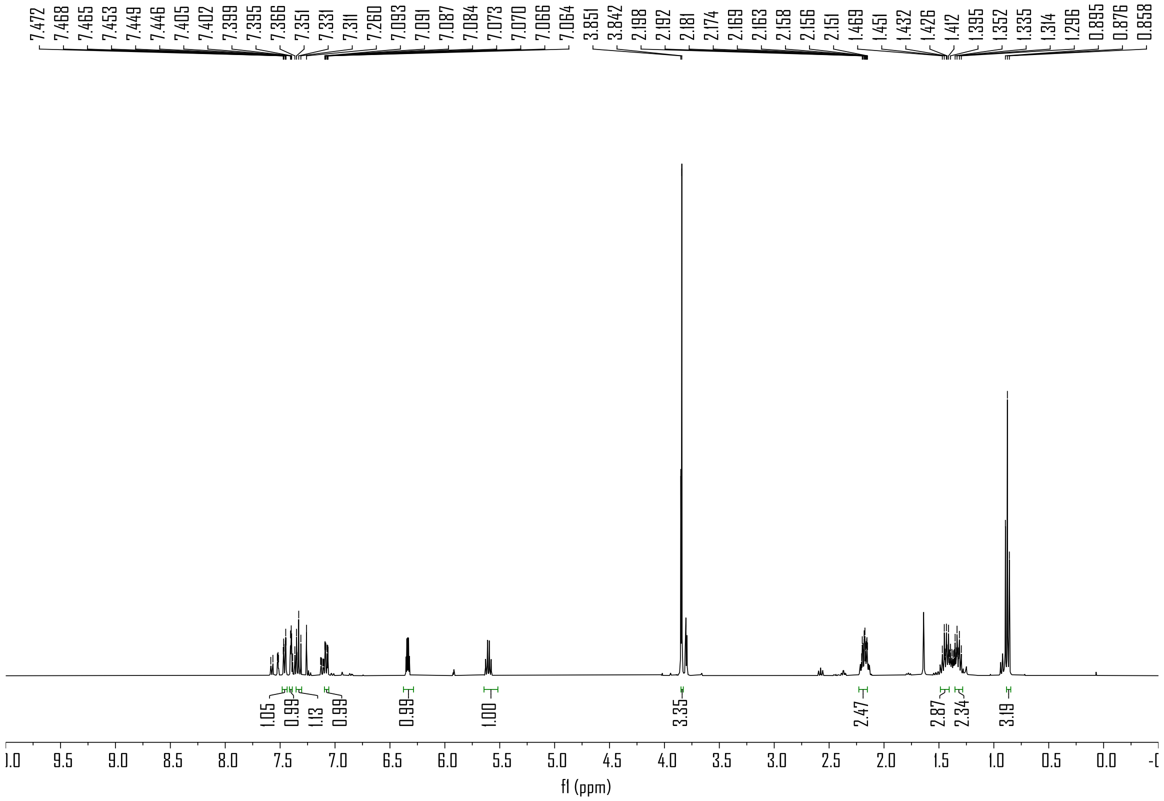
7.472
7.468
7.465
7.453
7.449
7.446
7.405
7.402
7.399
7.395
7.366
7.351
7.331
7.311
7.260
7.093
7.091
7.087
7.084
7.073
7.070
7.066
7.064
3.851
3.842
2.198
2.192
2.181
2.174
2.169
2.163
2.158
2.156
2.151
1.469
1.451
1.432
1.426
1.412
1.395
1.352
1.335
1.314
1.296
0.895
0.876
0.858
1.05
0.99
1.13
0.99
0.99
1.00
3.35
2.47
2.87
2.34
3.19
f1 (ppm)

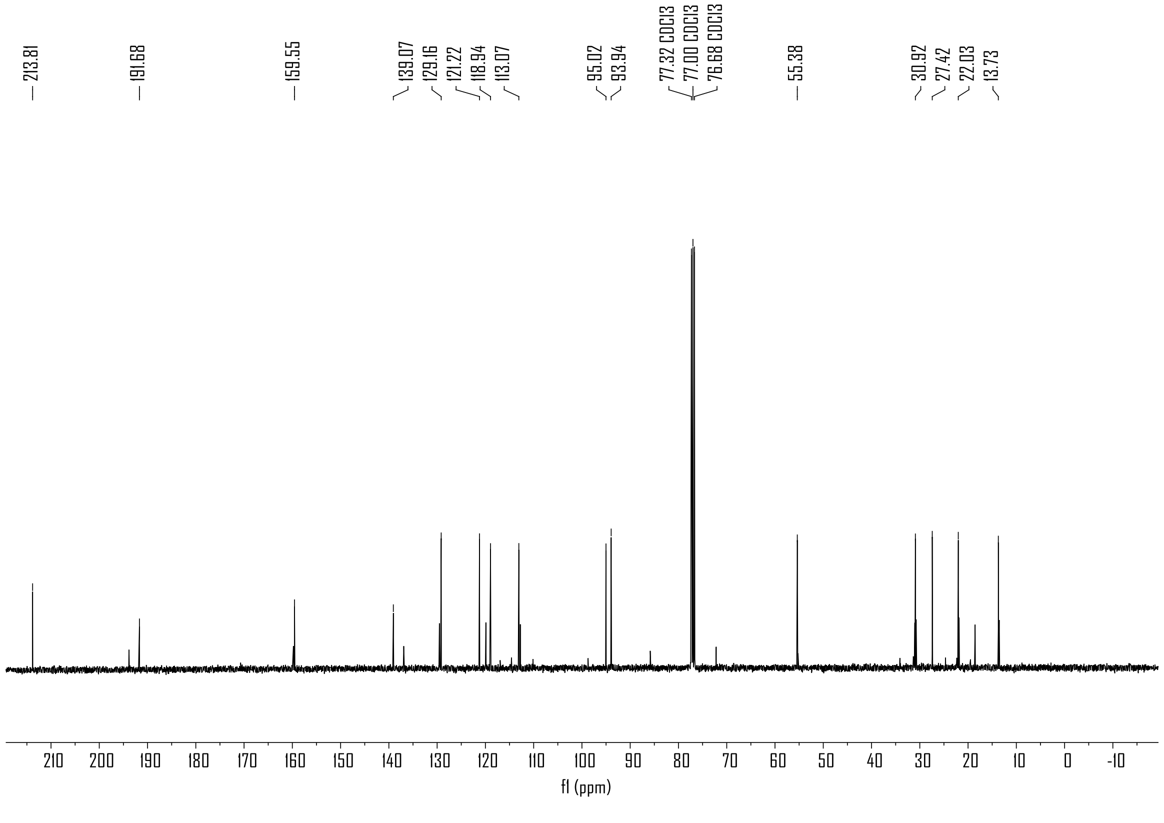
213.81
191.68
159.55
139.07
129.16
121.22
118.94
113.07
95.02
93.94
77.32 CDCl3
77.00 CDCl3
76.68 CDCl3
55.38
30.92
27.42
22.03
13.73
f1 (ppm)

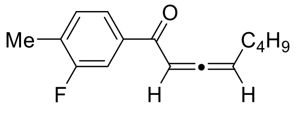
Me
F
O
H
H
C4H9

1i

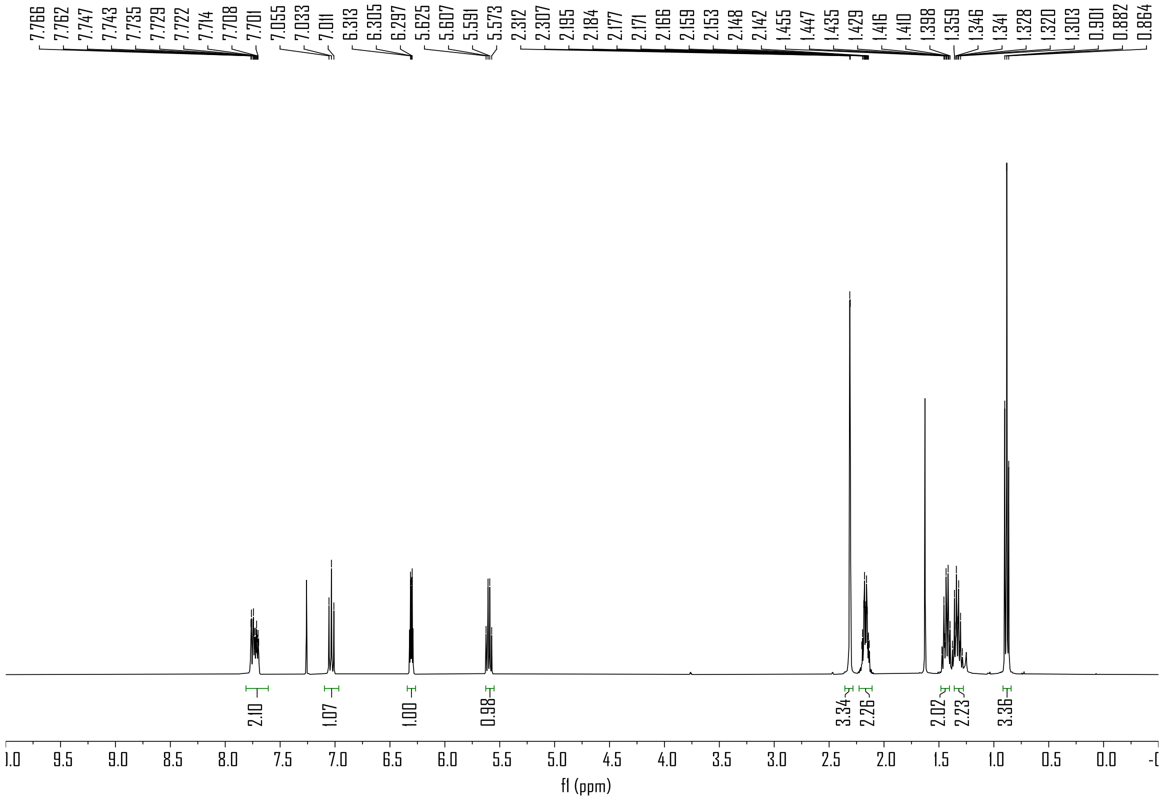

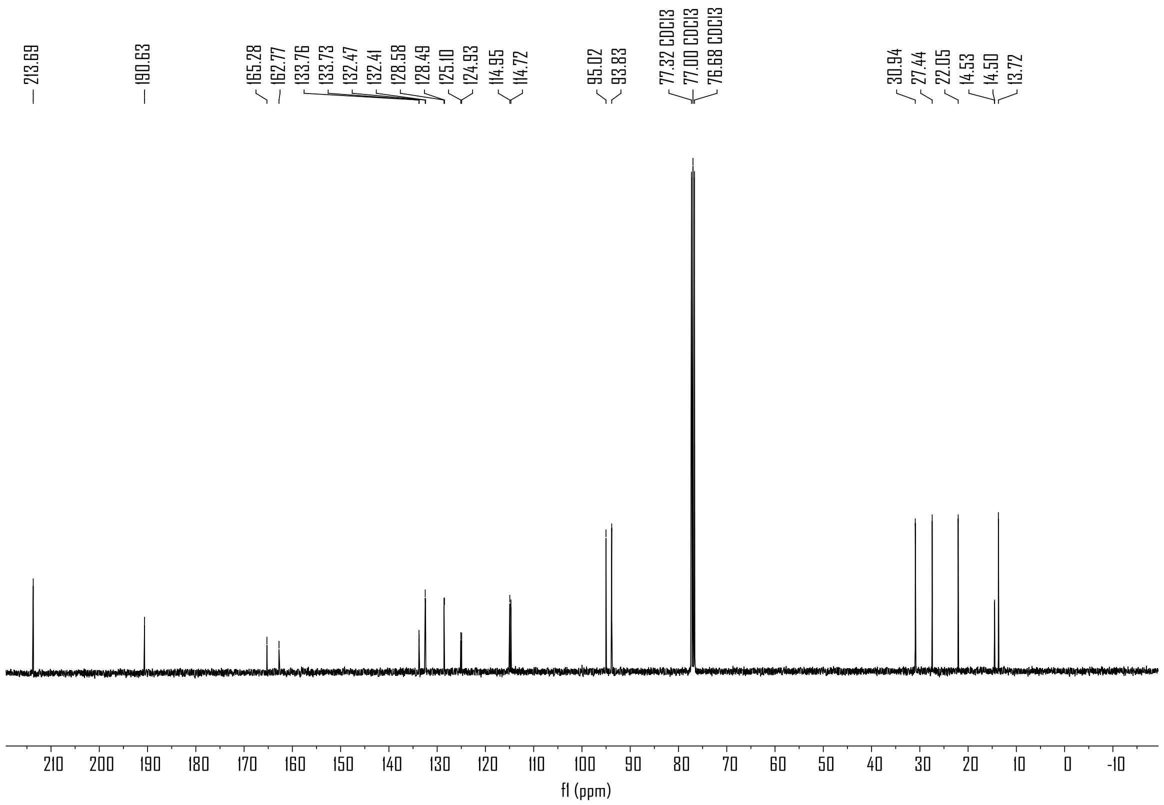

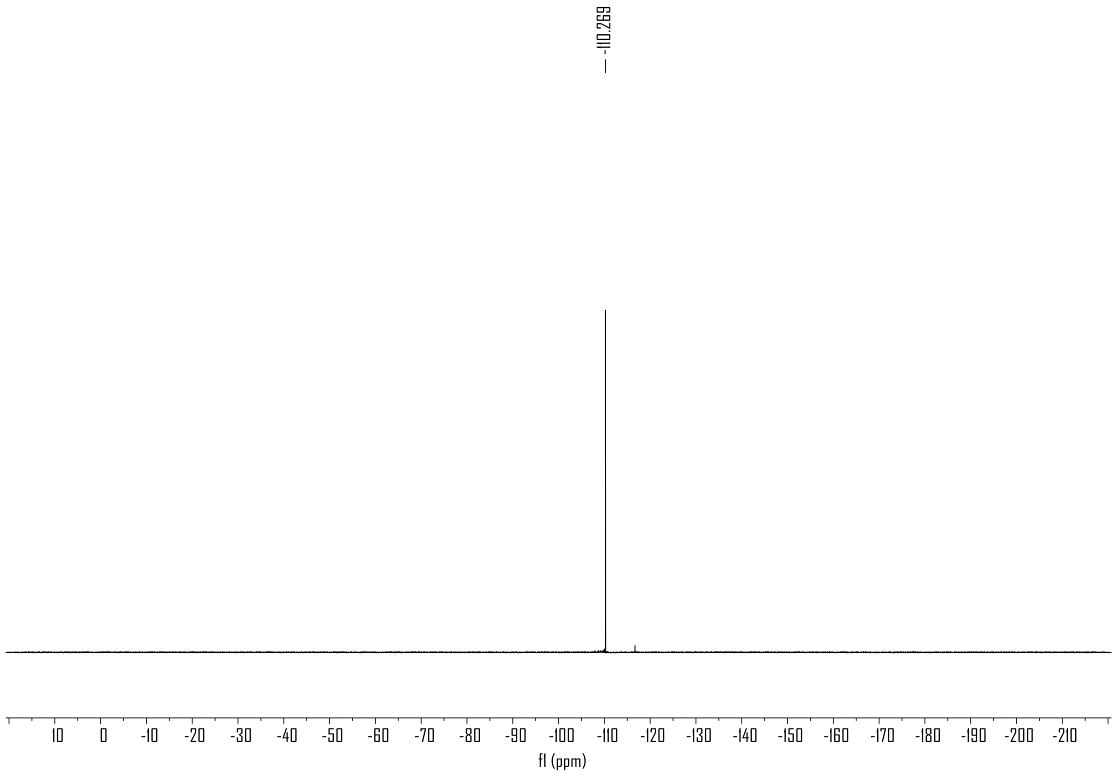
-110.269
10
0
-10
-20
-30
-40
-50
-60
-70
-80
-90
-100
-110
-120
-130
-140
-150
-160
-170
-180
-190
-200
-210
f1 (ppm)

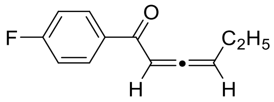
F
O
C2H5
H
H

1l

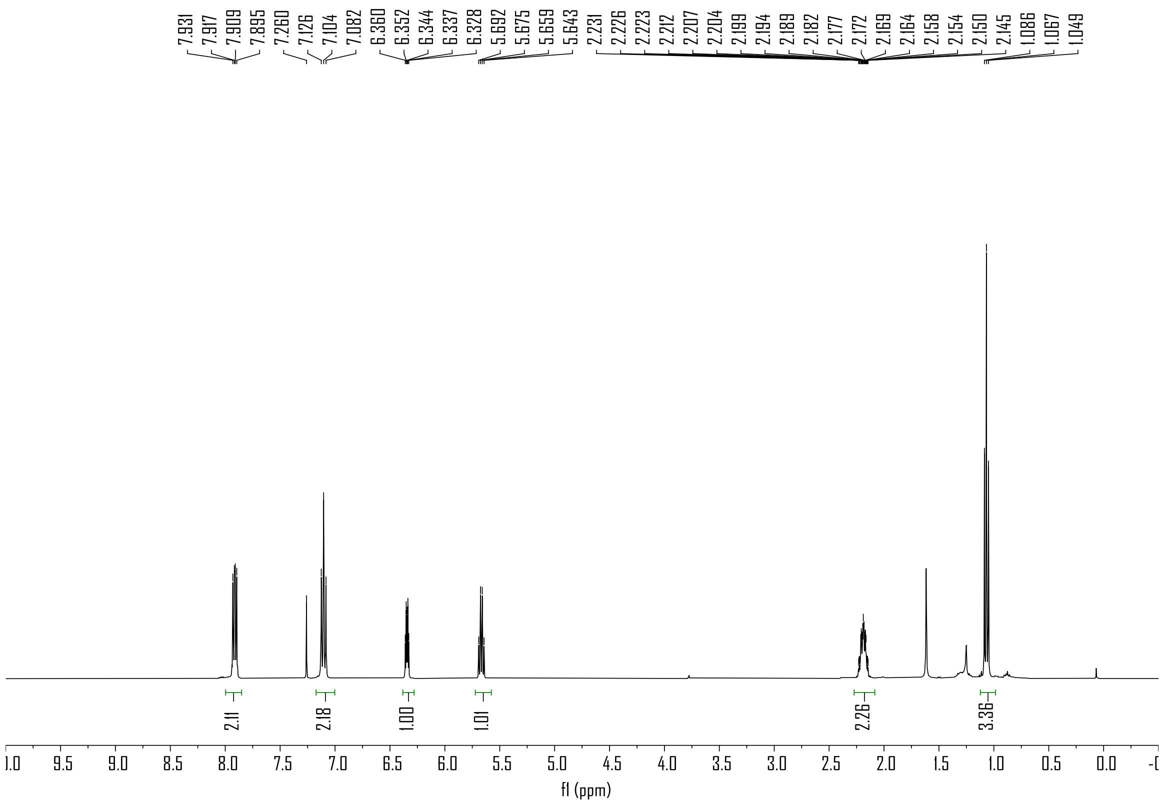
7.931
7.917
7.909
7.895
7.260
7.126
7.104
7.082
6.360
6.352
6.344
6.337
6.328
5.692
5.675
5.659
5.643
2.231
2.226
2.223
2.212
2.207
2.204
2.199
2.194
2.189
2.182
2.177
2.172
2.169
2.164
2.158
2.154
2.150
2.145
1.086
1.067
1.049
2.11
2.18
1.00
1.01
2.26
3.36
fl (ppm)

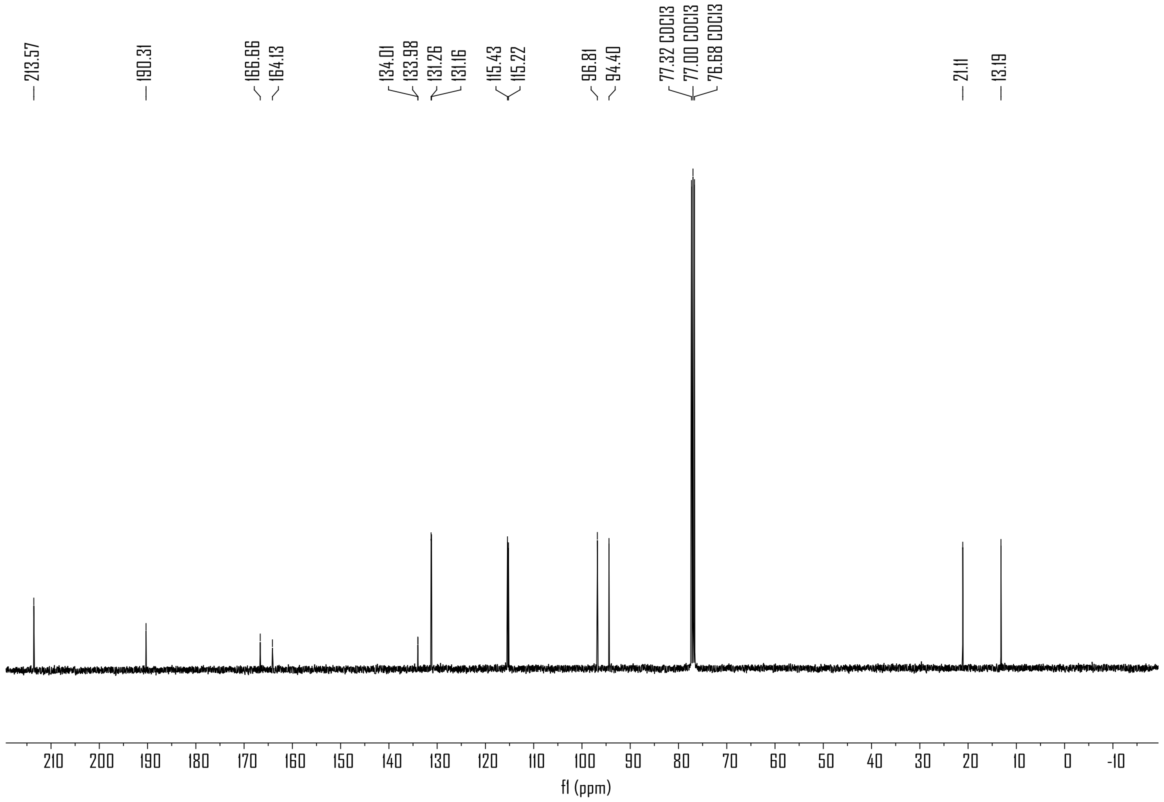
213.57
190.31
166.66
164.13
134.01
133.98
131.26
131.16
115.43
115.22
96.81
94.40
77.32 CDCl3
77.00 CDCl3
76.68 CDCl3
21.11
13.19
fl (ppm)

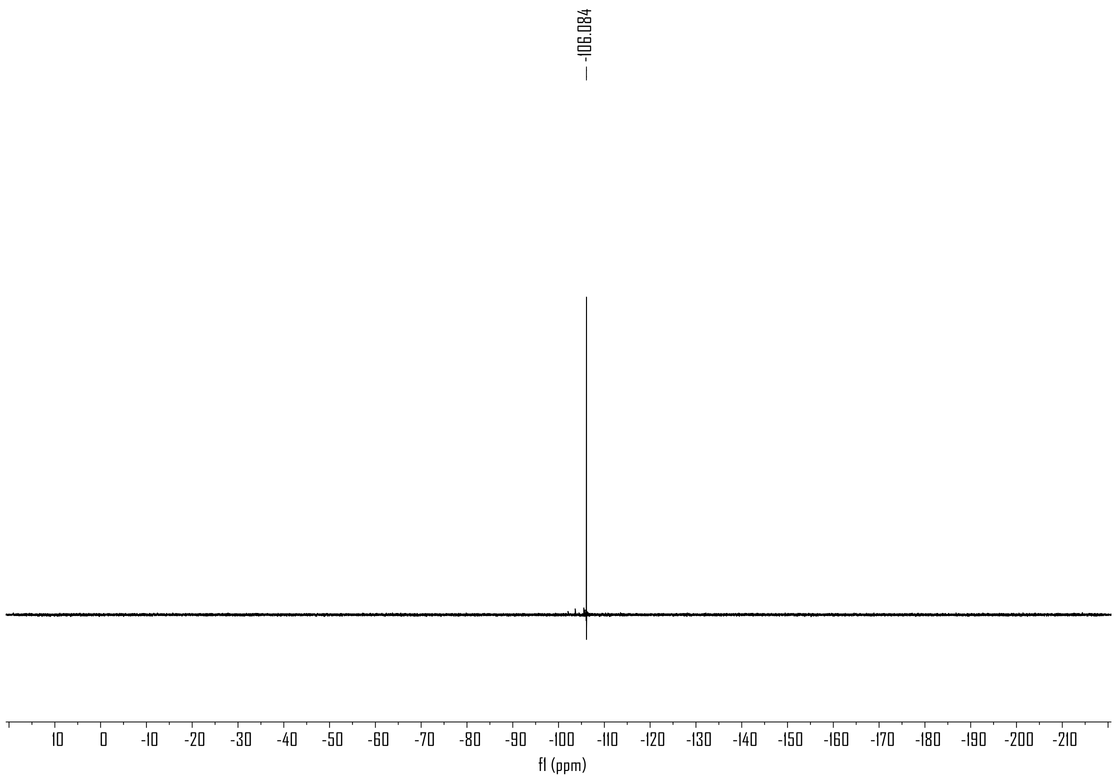

-106.084
10
0
-10
-20
-30
-40
-50
-60
-70
-80
-90
-100
-110
-120
-130
-140
-150
-160
-170
-180
-190
-200
-210
f1 (ppm)

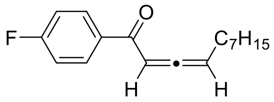

F
O
C7H15
H
H

1m

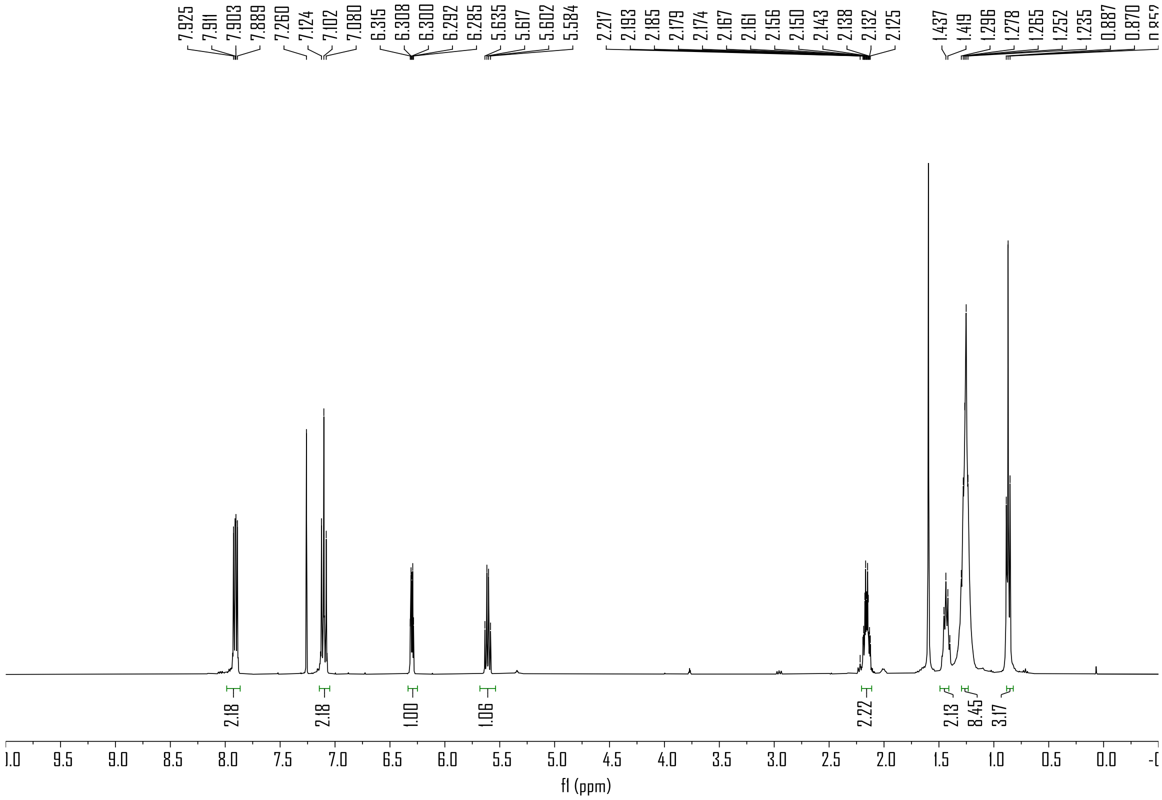
7.925
7.911
7.903
7.889
7.260
7.124
7.102
7.080
6.315
6.308
6.300
6.292
6.285
5.635
5.617
5.602
5.584
2.217
2.193
2.185
2.179
2.174
2.167
2.161
2.156
2.150
2.143
2.138
2.132
2.125
1.437
1.419
1.296
1.278
1.265
1.252
1.235
0.887
0.870
2.18
2.18
1.00
1.06
2.22
2.13
8.45
3.17
fl (ppm)

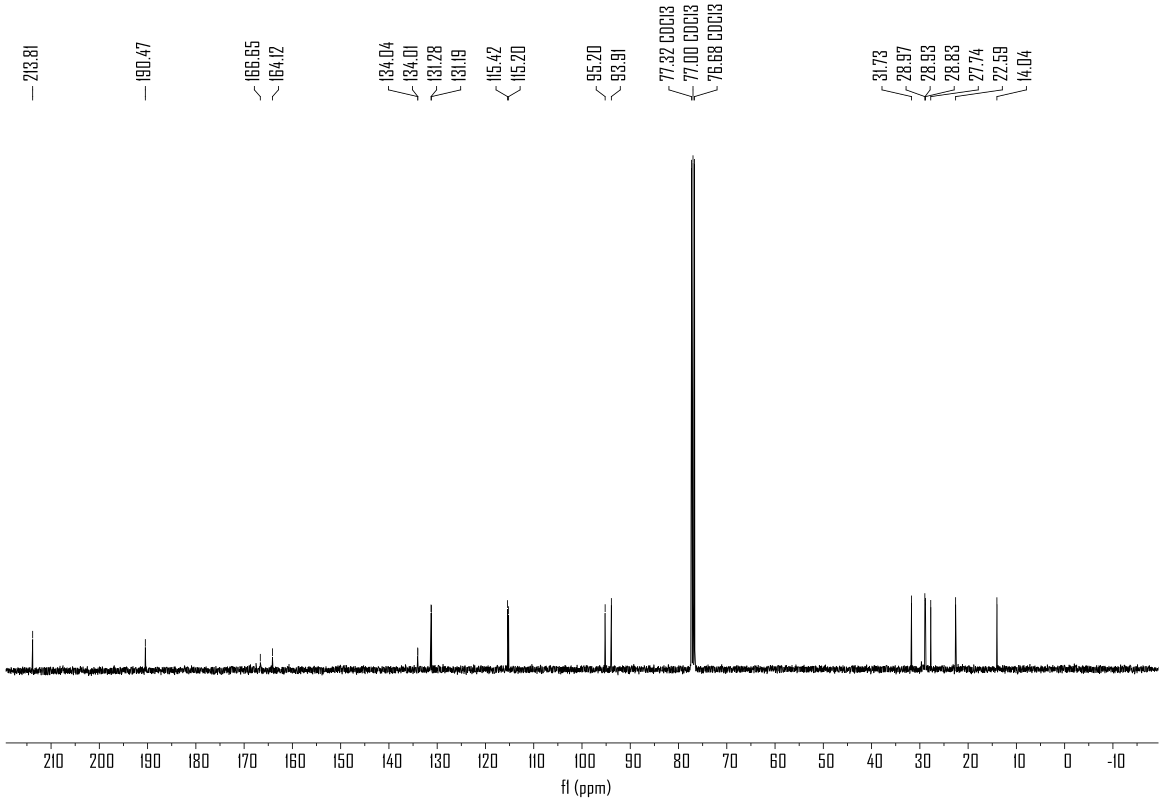
213.81
190.47
166.65
164.12
134.04
134.01
131.28
131.19
115.42
115.20
95.20
93.91
77.32 CDCl3
77.00 CDCl3
76.68 CDCl3
31.73
28.97
28.93
28.83
27.74
22.59
14.04
fl (ppm)

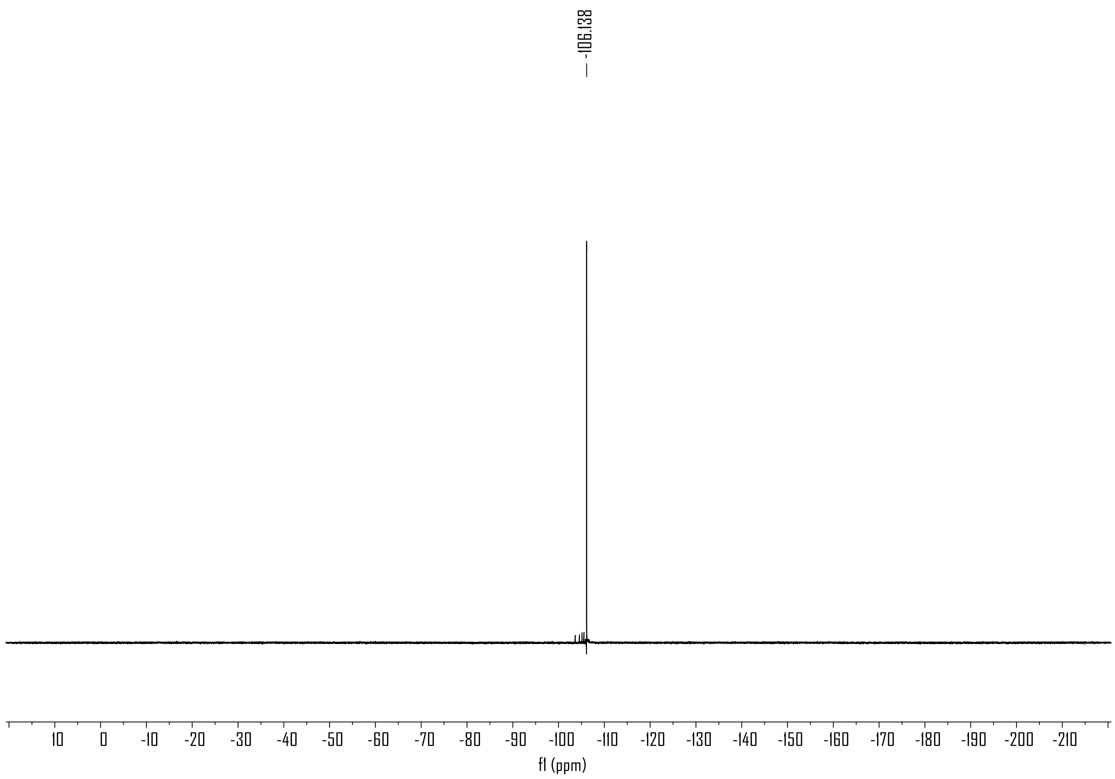

-106.138
10
0
-10
-20
-30
-40
-50
-60
-70
-80
-90
-100
-110
-120
-130
-140
-150
-160
-170
-180
-190
-200
-210
f1 (ppm)

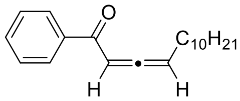

O
C10H21
H
H

1n

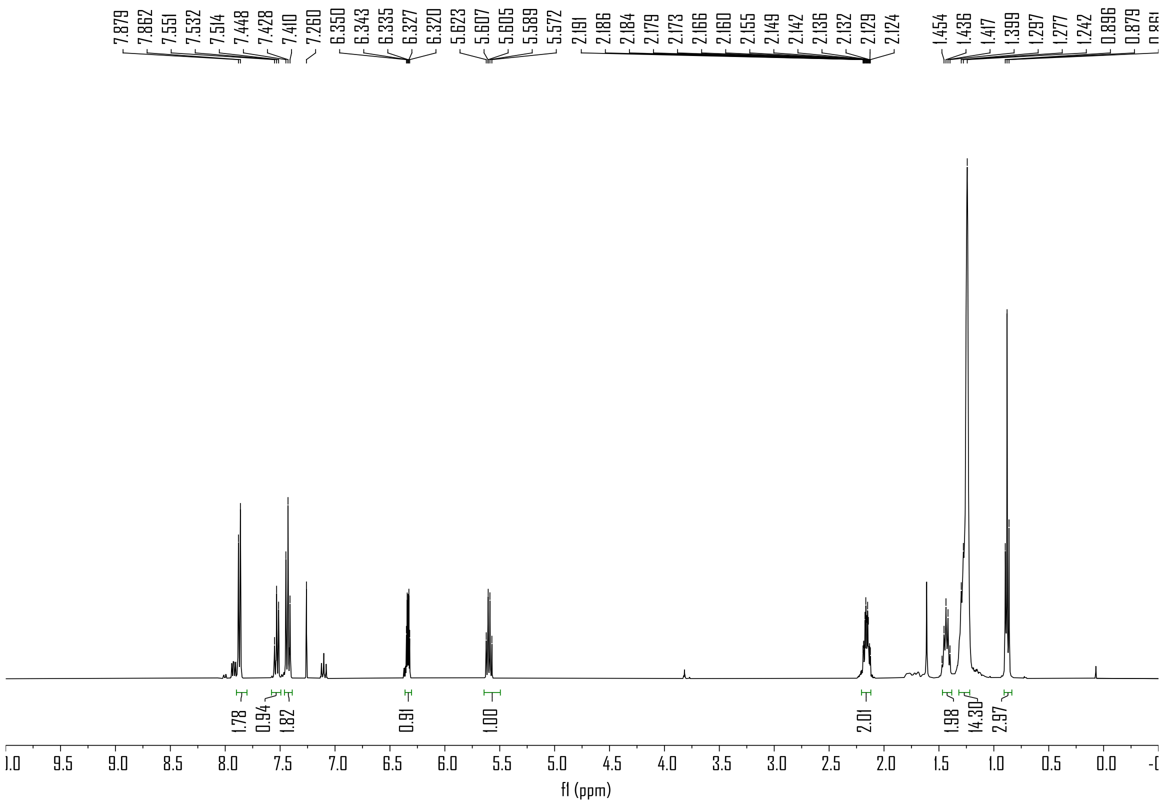
7.879
7.862
7.551
7.532
7.514
7.448
7.428
7.410
7.260
6.350
6.343
6.335
6.327
6.320
5.623
5.607
5.605
5.589
5.572
2.191
2.186
2.184
2.179
2.173
2.166
2.160
2.155
2.149
2.142
2.136
2.132
2.129
2.124
1.454
1.436
1.417
1.399
1.297
1.277
1.242
0.896
0.879
1.78
0.94
1.82
0.91
1.00
2.01
1.98
14.30
2.97
f1 (ppm)

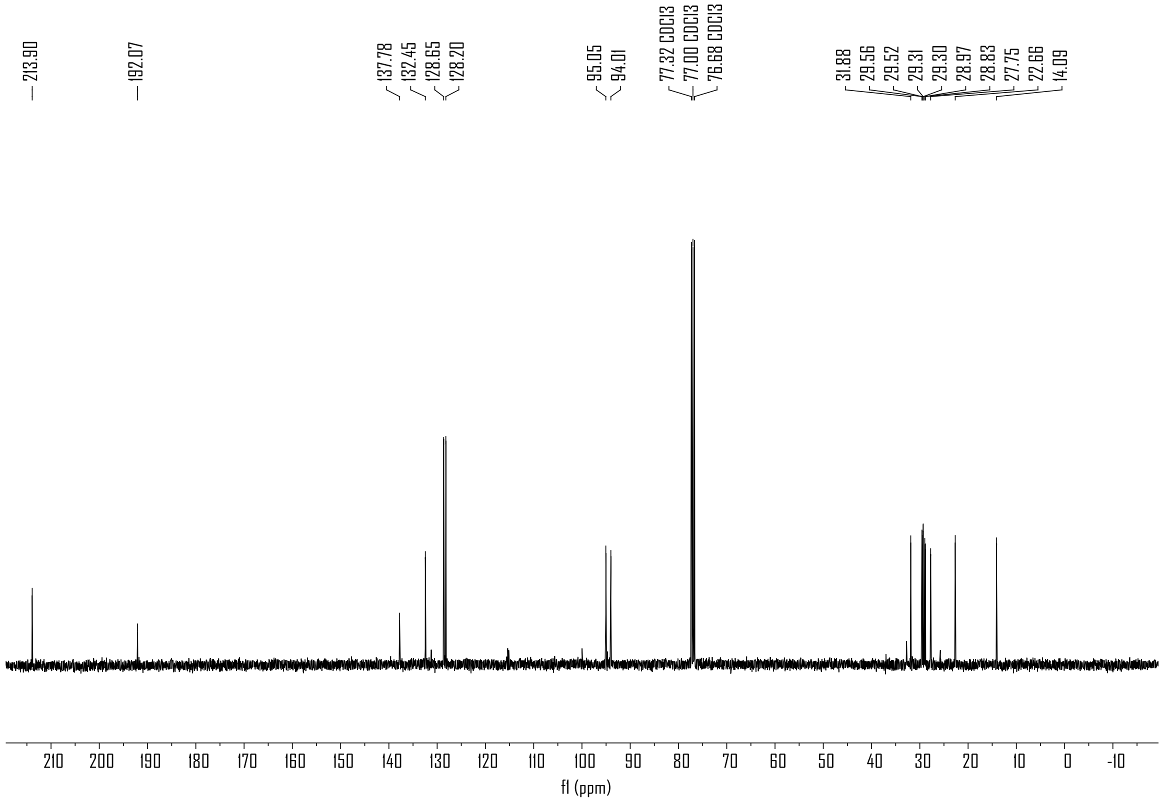
213.90
192.07
137.78
132.45
128.65
128.20
95.05
94.01
77.32 CDCl3
77.00 CDCl3
76.68 CDCl3
31.88
29.56
29.52
29.31
29.30
28.97
28.83
27.75
22.66
14.09
f1 (ppm)

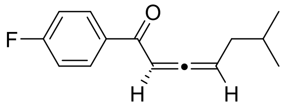
F
O
H
H

1o

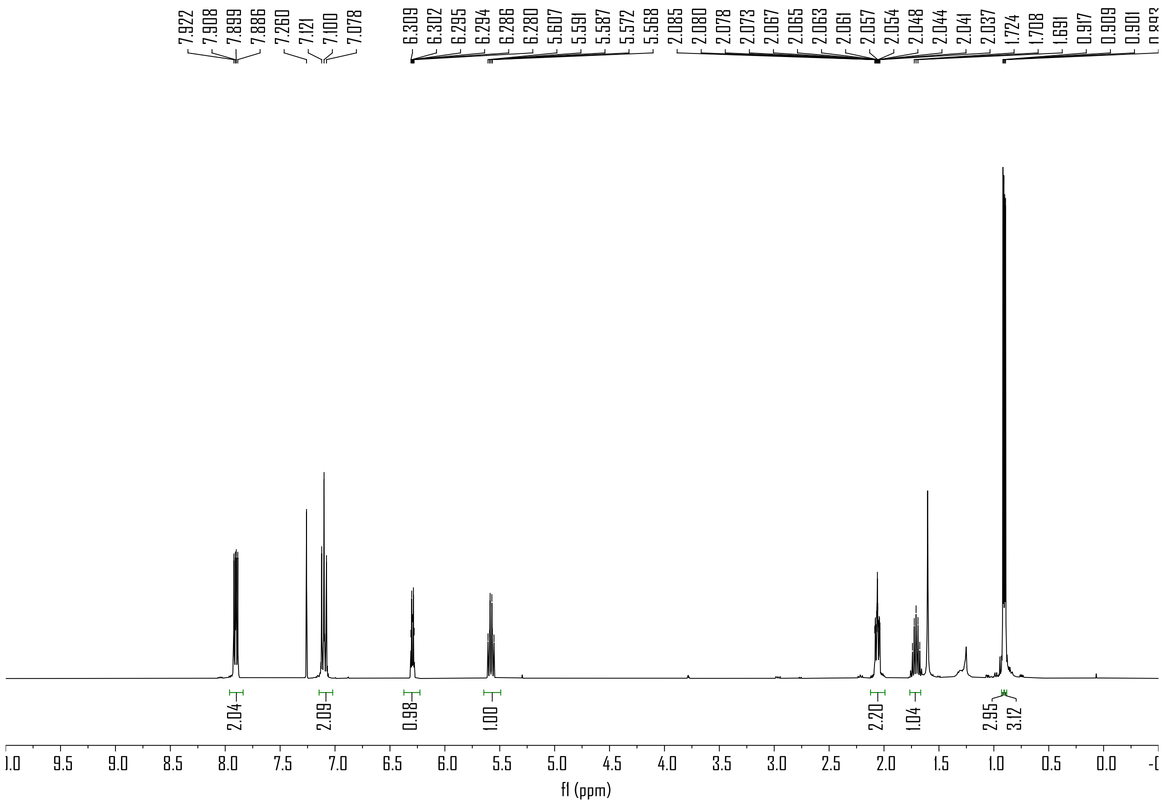
7.922
7.908
7.899
7.886
7.260
7.121
7.100
7.078
6.309
6.302
6.295
6.294
6.286
6.280
5.607
5.591
5.587
5.572
5.568
2.085
2.080
2.078
2.073
2.067
2.065
2.063
2.061
2.057
2.054
2.048
2.044
2.041
2.037
1.724
1.708
1.691
0.917
0.909
0.901
2.04
2.09
0.98
1.00
2.20
1.04
2.95
3.12
f1 (ppm)

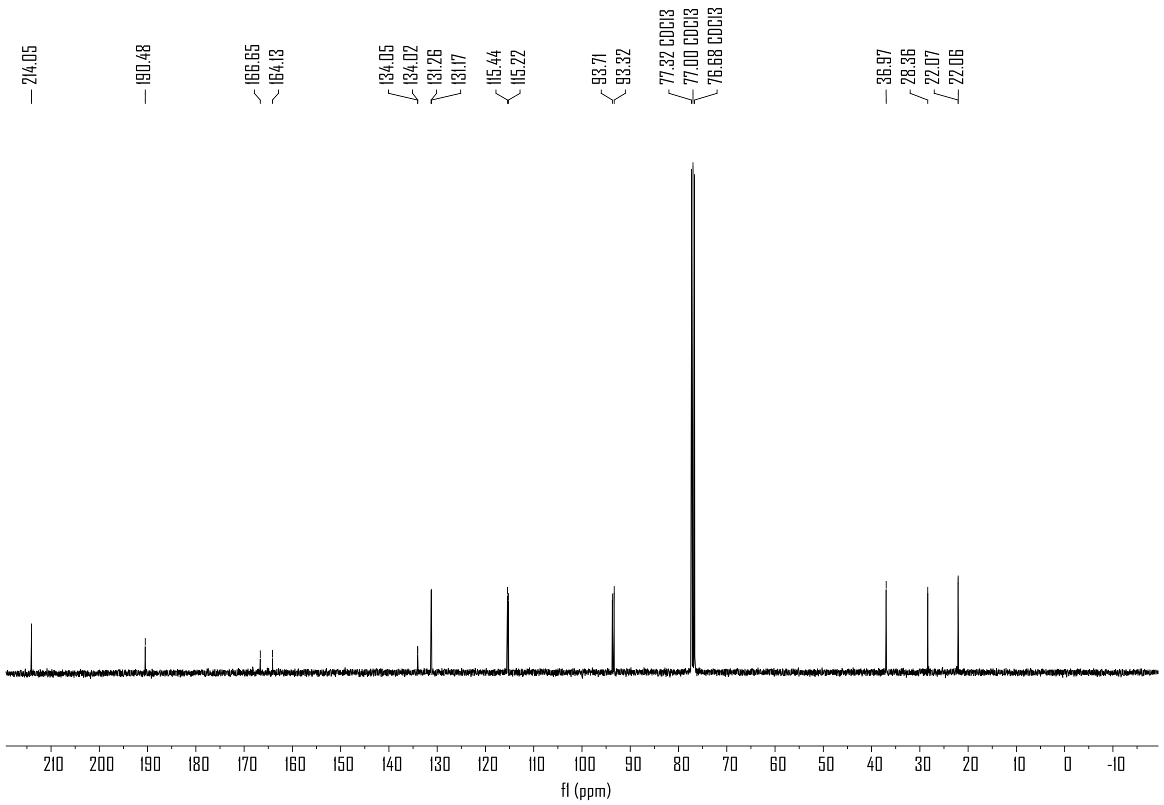
214.05
190.48
166.65
164.13
134.05
134.02
131.26
131.17
115.44
115.22
93.71
93.32
77.32 CDCl3
77.00 CDCl3
76.68 CDCl3
36.97
28.36
22.07
22.06
f1 (ppm)

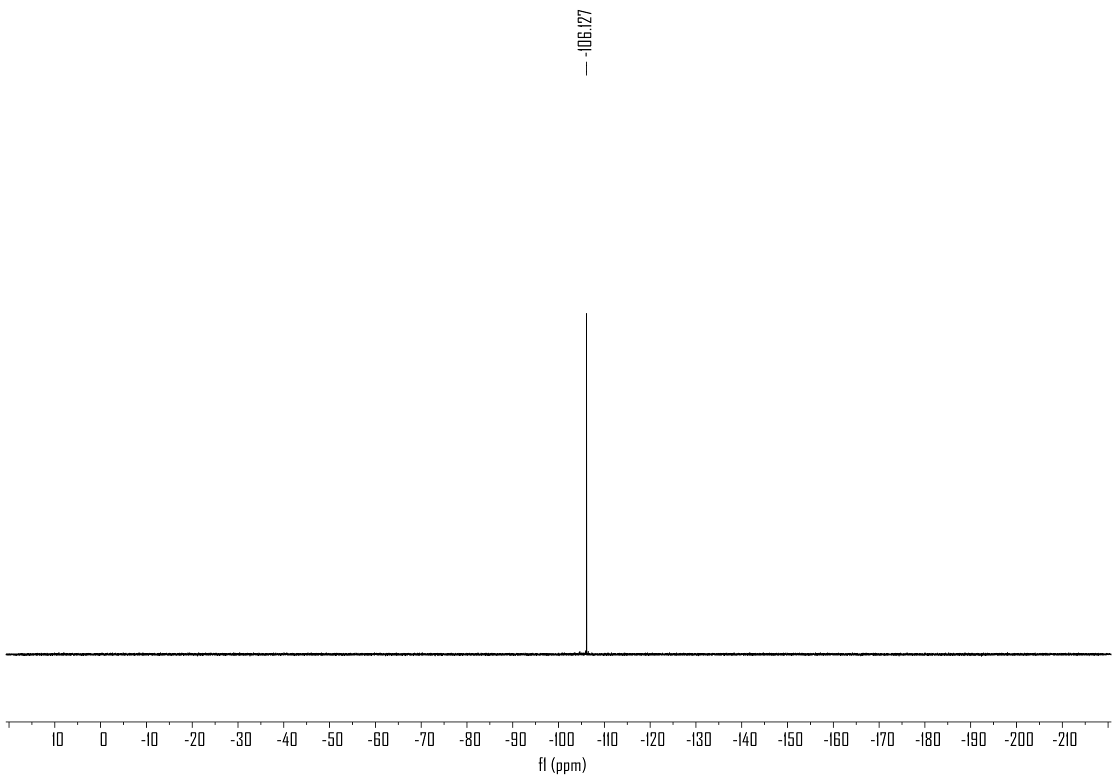
-106.127
10 0 -10 -20 -30 -40 -50 -60 -70 -80 -90 -100 -110 -120 -130 -140 -150 -160 -170 -180 -190 -200 -210
f1 (ppm)

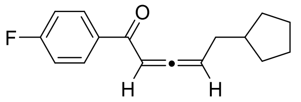
F
O
H
H

1p

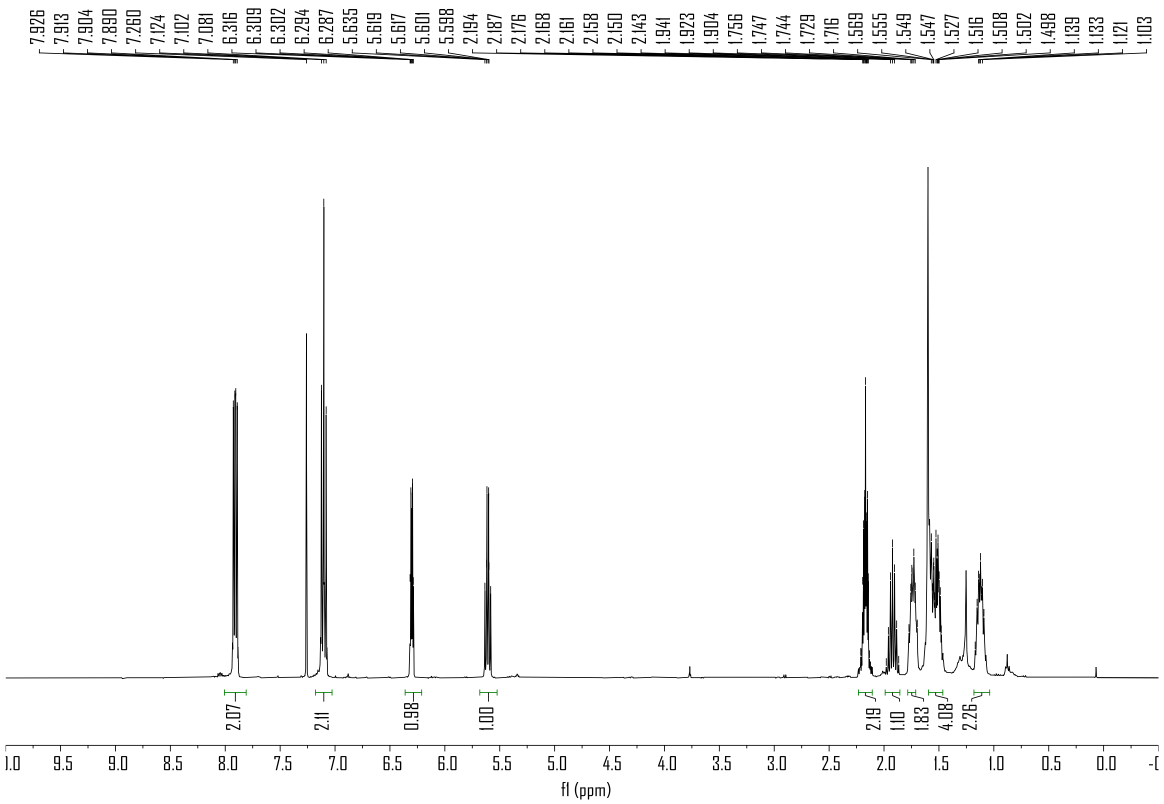
7.926
7.913
7.904
7.890
7.260
7.124
7.102
7.081
6.316
6.309
6.302
6.294
6.287
5.635
5.619
5.617
5.601
5.598
2.194
2.187
2.176
2.168
2.161
2.158
2.150
2.143
1.941
1.923
1.904
1.756
1.747
1.744
1.729
1.716
1.569
1.555
1.549
1.547
1.527
1.516
1.508
1.502
1.498
1.139
1.133
1.121
1.103
2.07
2.11
0.98
1.00
2.19
1.10
1.83
4.08
2.26
f1 (ppm)

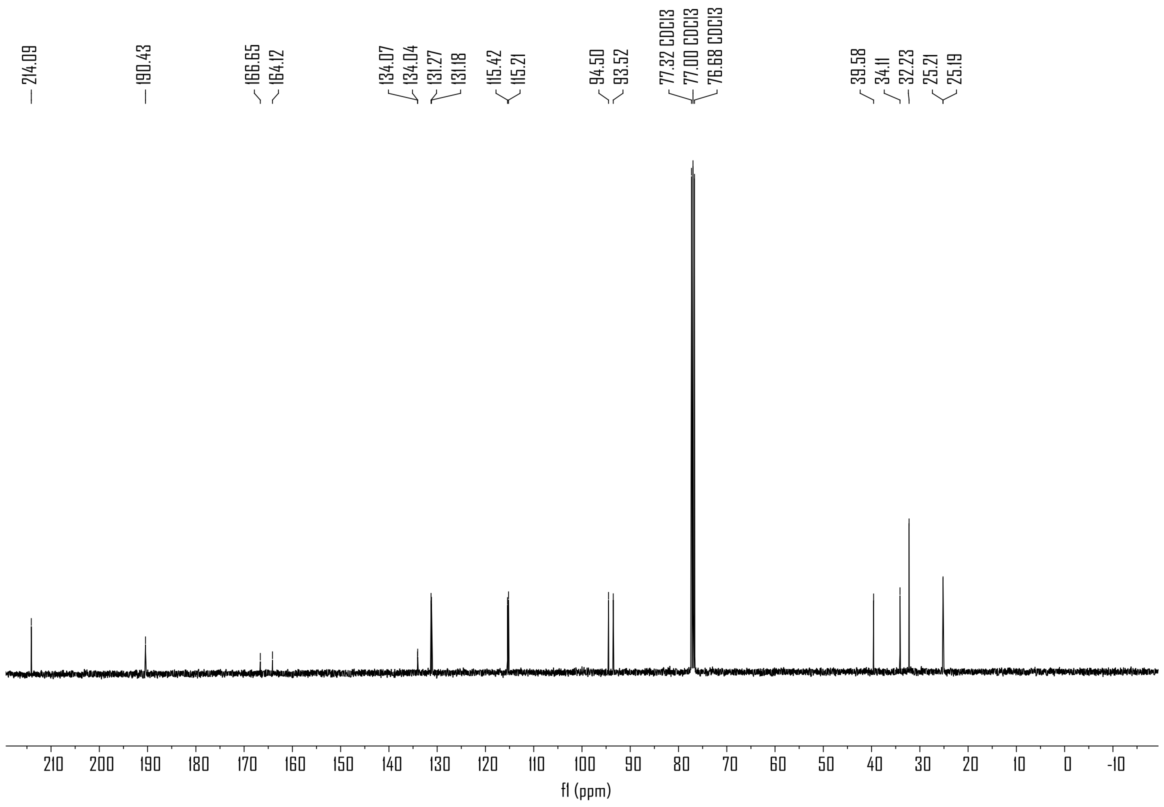
214.09
190.43
166.65
164.12
134.07
134.04
131.27
131.18
115.42
115.21
94.50
93.52
77.32 CDCl3
77.00 CDCl3
76.68 CDCl3
39.58
34.11
32.23
25.21
25.19
f1 (ppm)

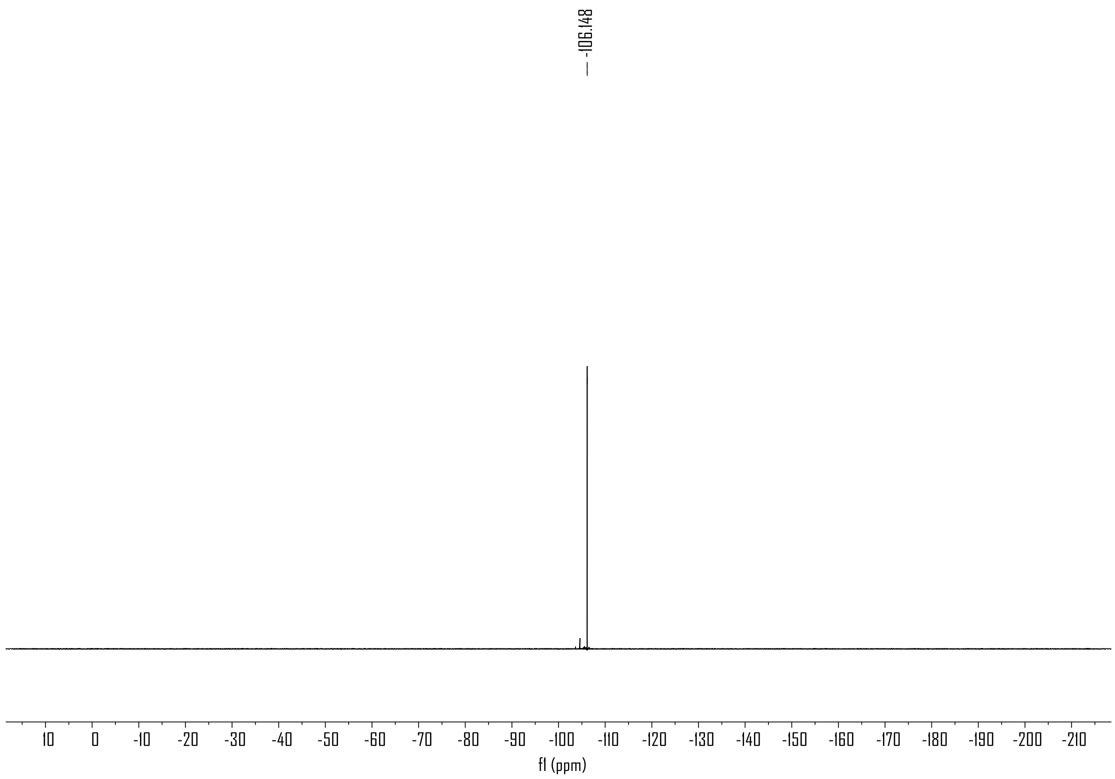
-106.148
10
0
-10
-20
-30
-40
-50
-60
-70
-80
-90
-100
-110
-120
-130
-140
-150
-160
-170
-180
-190
-200
-210
f1 (ppm)

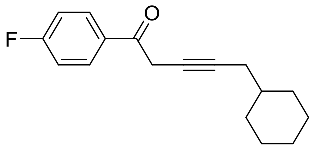
F
O

1q

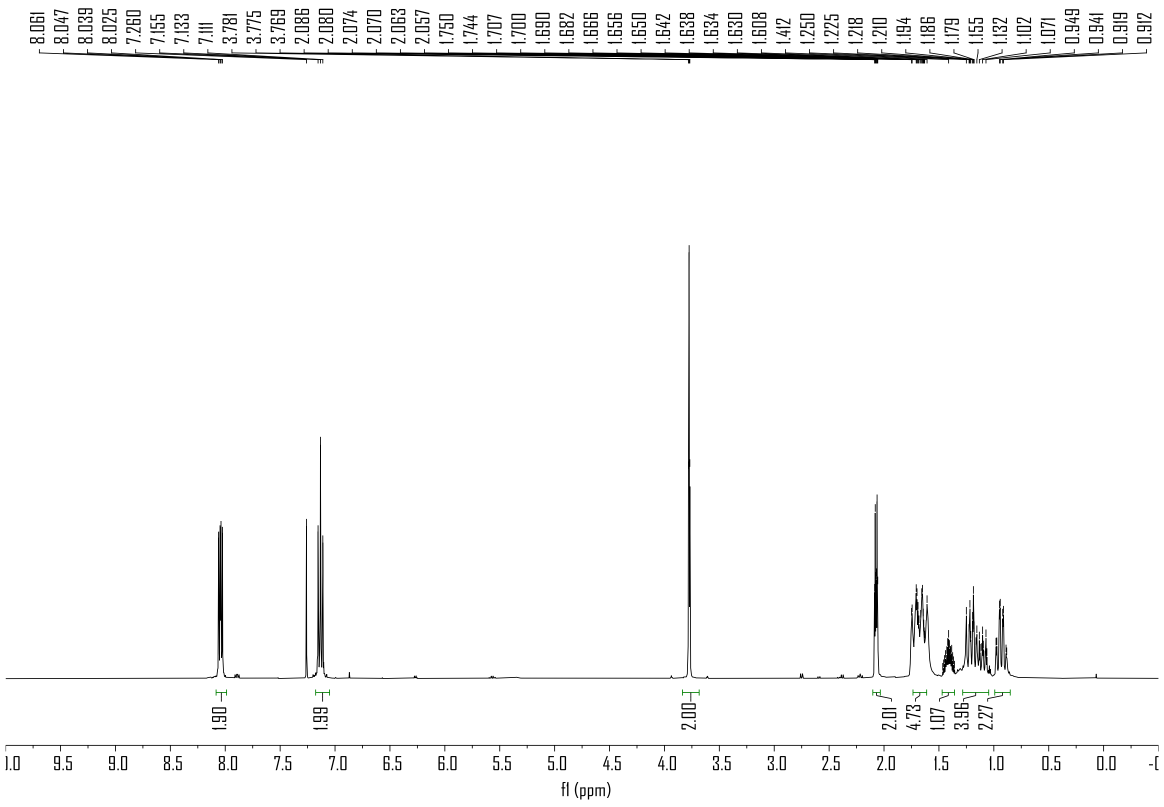
8.061 8.047 8.039 8.025 7.260 7.155 7.133 7.111 3.781 3.775 3.769 2.086 2.080 2.074 2.070 2.063 2.057 1.750 1.744 1.707 1.700 1.690 1.682 1.666 1.656 1.650 1.642 1.638 1.634 1.630 1.608 1.412 1.250 1.225 1.218 1.210 1.194 1.186 1.179 1.155 1.132 1.102 1.071 0.949 0.941 0.919 0.912
1.90
1.99
2.00
2.01
4.73
1.07
3.96
2.27
fl (ppm)

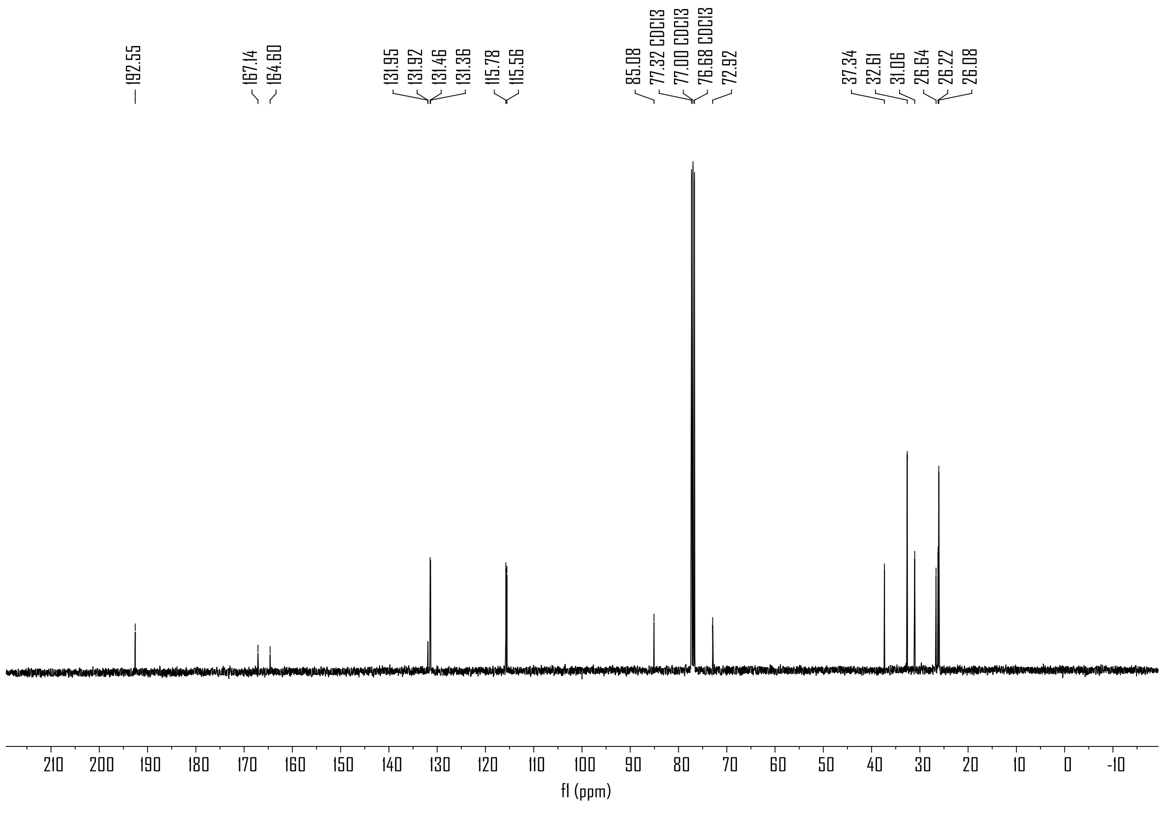
192.55
167.14
164.60
131.95
131.92
131.46
131.36
115.78
115.56
85.08
77.32 CDCl3
77.00 CDCl3
76.68 CDCl3
72.92
37.34
32.61
31.06
26.64
26.22
26.08
fl (ppm)

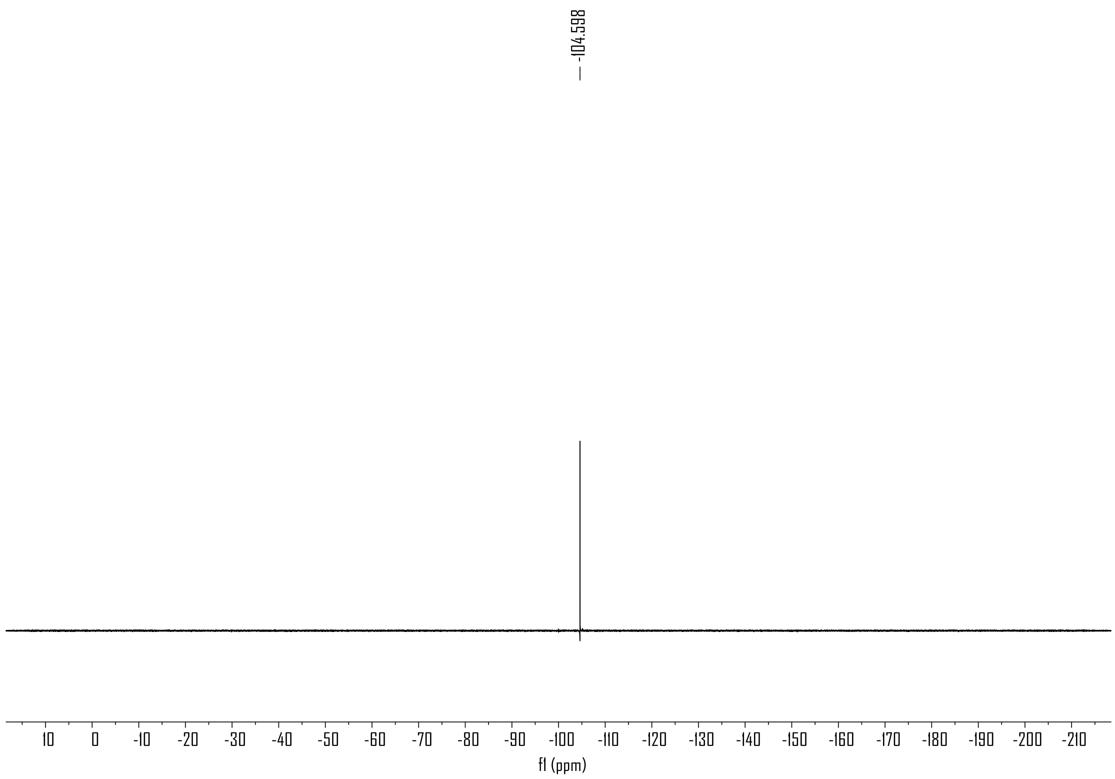

-104.598
10
0
-10
-20
-30
-40
-50
-60
-70
-80
-90
-100
-110
-120
-130
-140
-150
-160
-170
-180
-190
-200
-210
f1 (ppm)

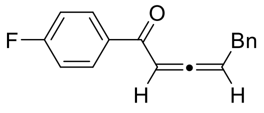

F
O
Bn
H
H

1r

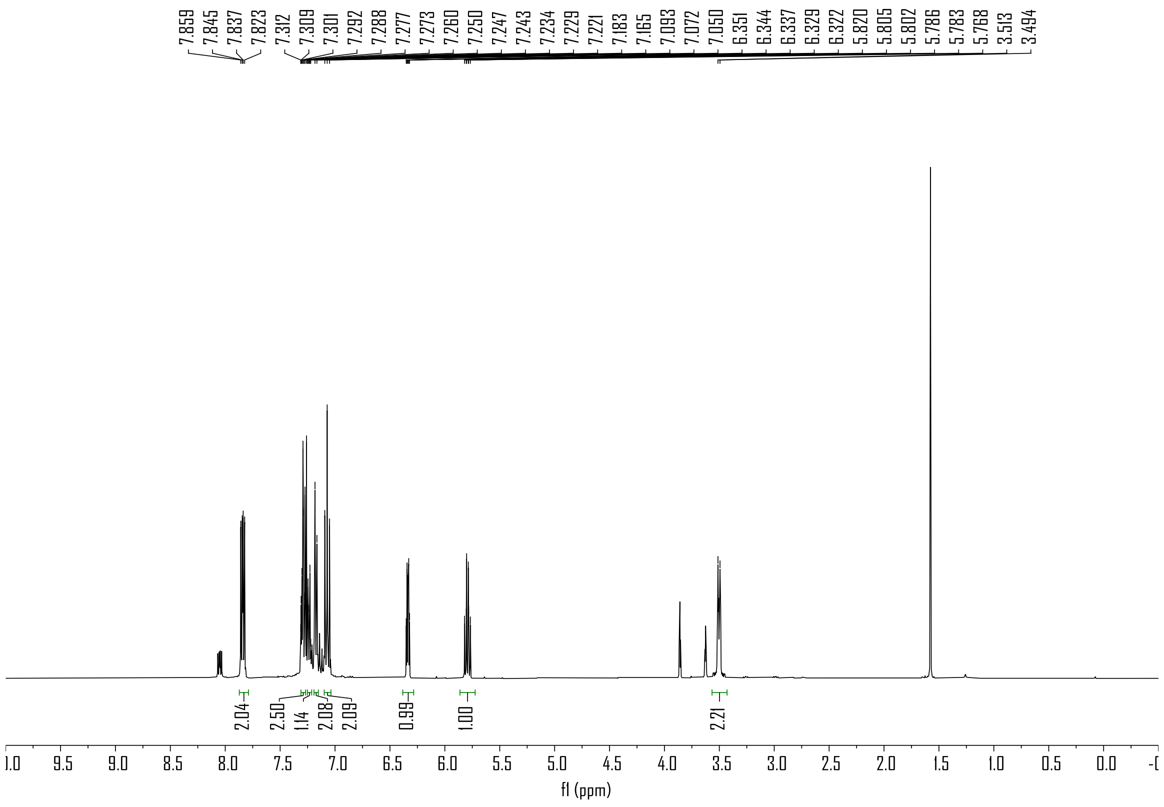

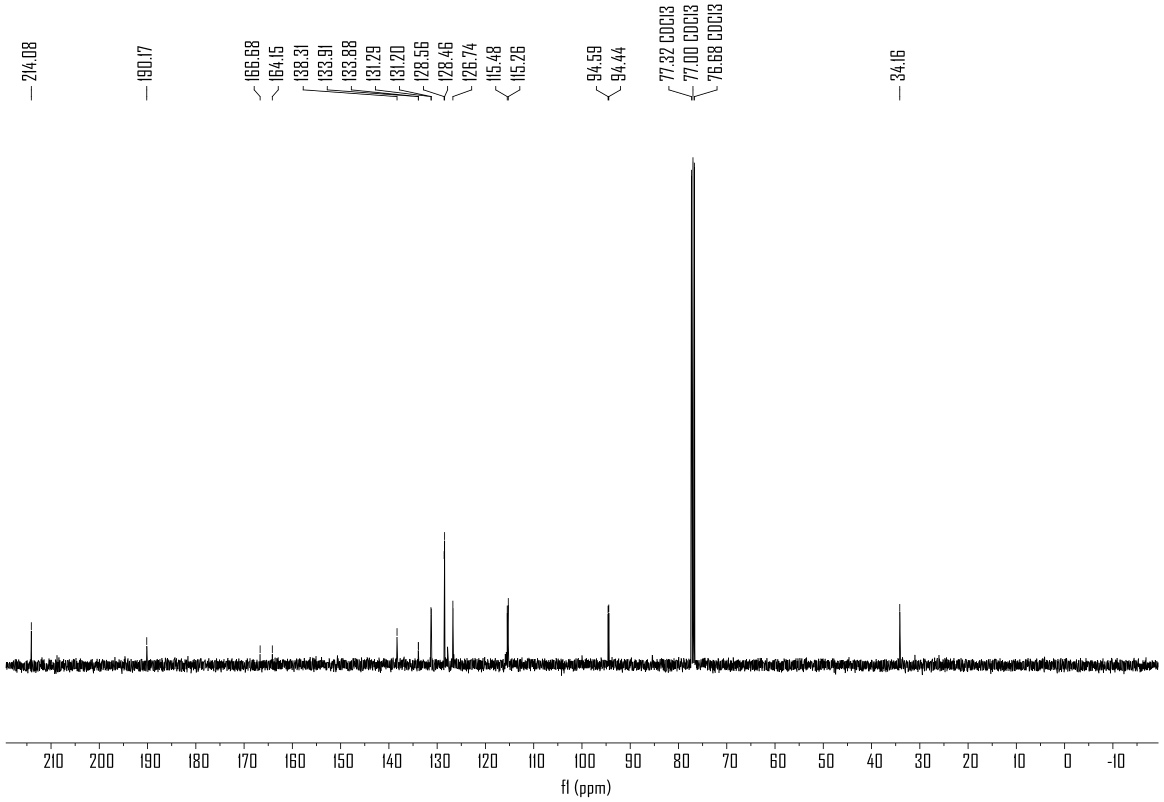

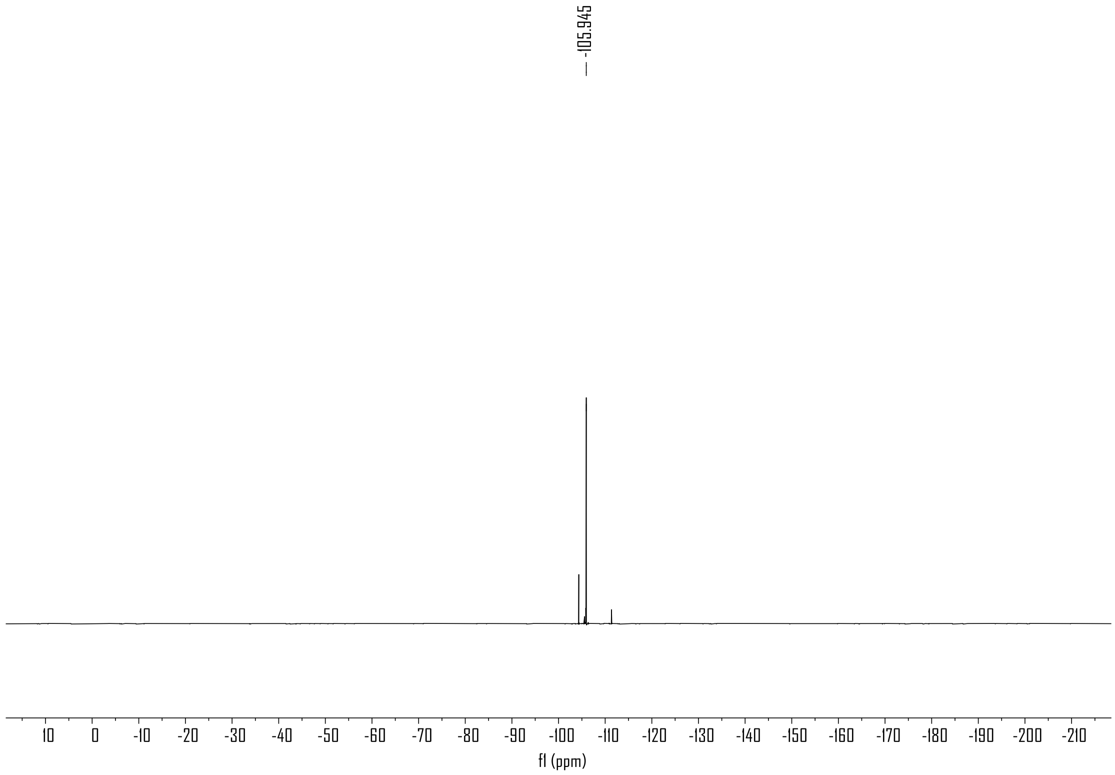
-105.945
10 0 -10 -20 -30 -40 -50 -60 -70 -80 -90 -100 -110 -120 -130 -140 -150 -160 -170 -180 -190 -200 -210
f1 (ppm)

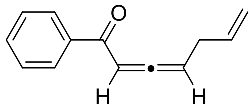
O
H
H

1s

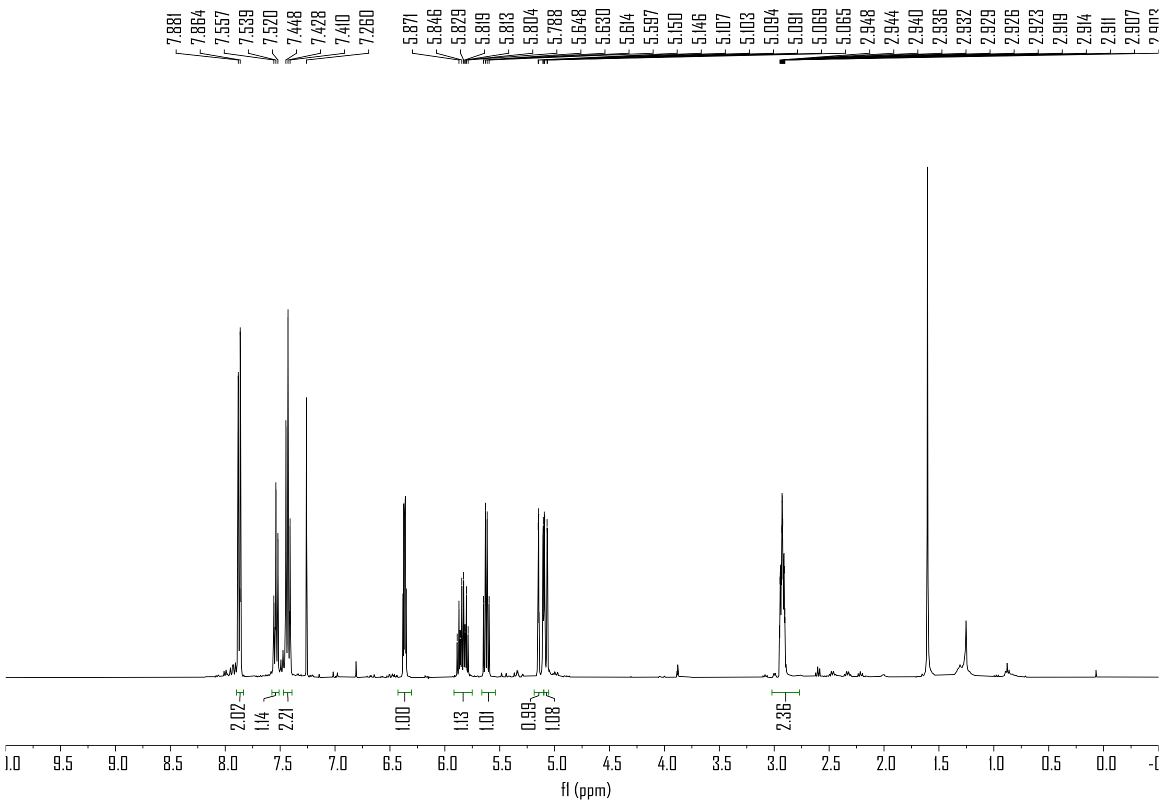

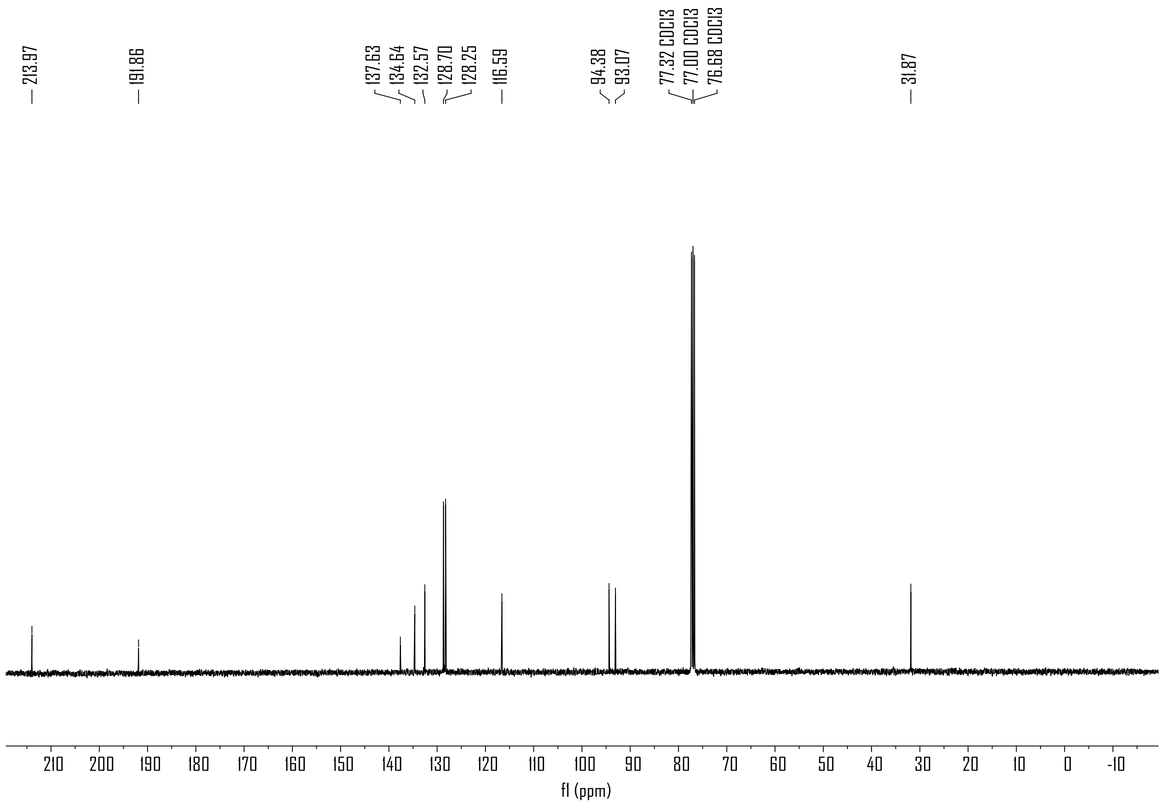

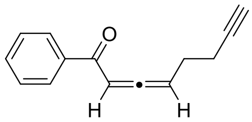

1t

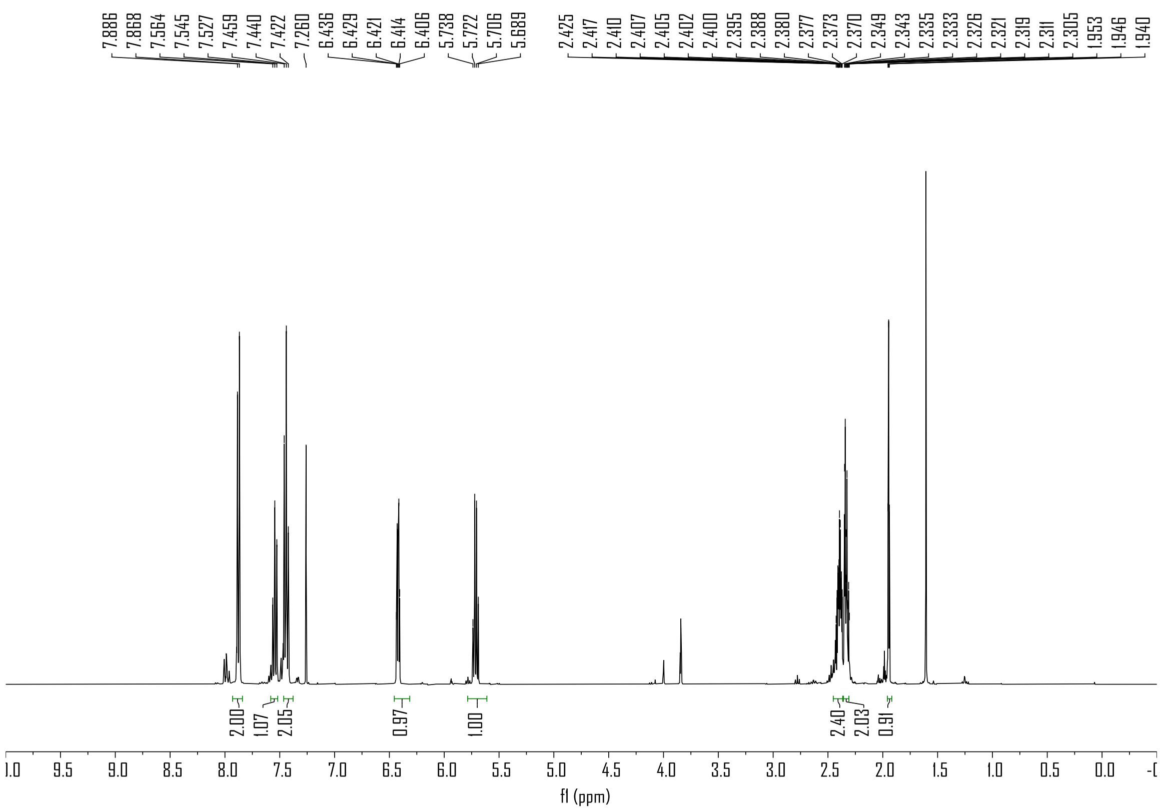
7.886 7.868 7.564 7.545 7.527 7.459 7.440 7.422 7.260 6.436 6.429 6.421 6.414 6.406 5.738 5.722 5.706 5.689 2.425 2.417 2.410 2.407 2.405 2.402 2.400 2.395 2.388 2.380 2.377 2.373 2.370 2.349 2.343 2.335 2.333 2.326 2.321 2.319 2.311 2.305 1.953 1.946 1.940
2.00 1.07 2.05 0.97 1.00 2.40 2.03 0.91
9.5 9.0 8.5 8.0 7.5 7.0 6.5 6.0 5.5 5.0 4.5 4.0 3.5 3.0 2.5 2.0 1.5 1.0 0.5 0.0
f1 (ppm)

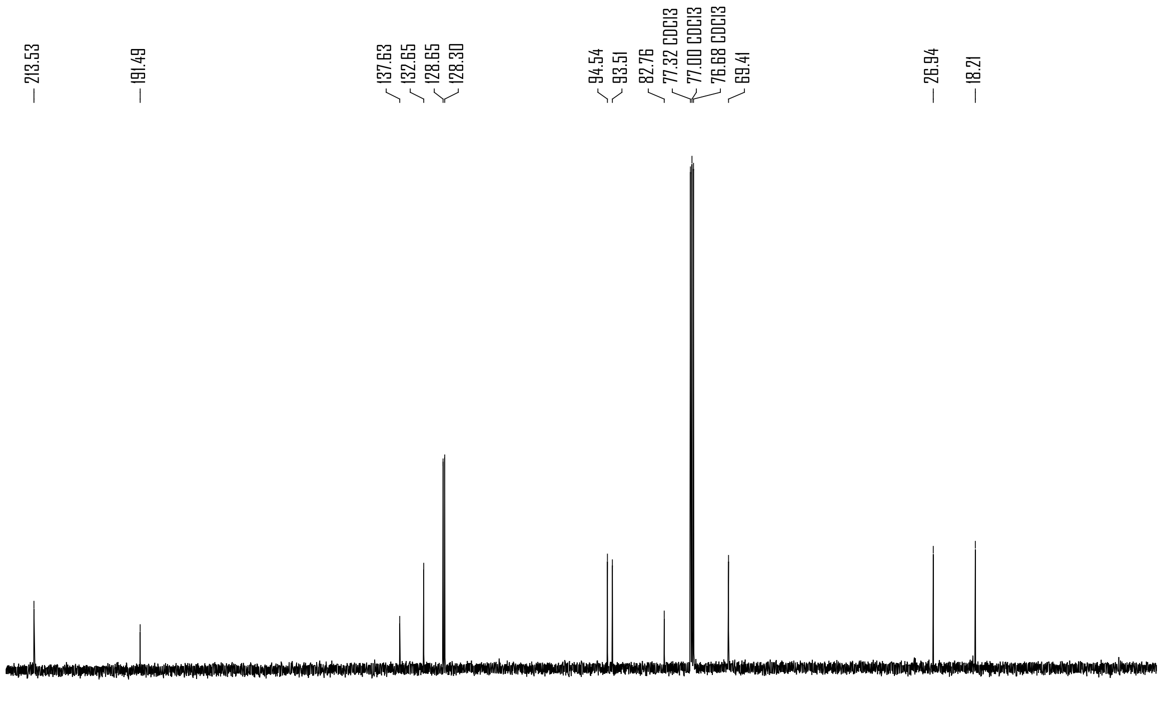
213.53 191.49 137.63 132.65 128.65 128.30 94.54 93.51 82.76 77.32 CDCl3 77.00 CDCl3 76.68 CDCl3 69.41 26.94 18.21
210 200 190 180 170 160 150 140 130 120 110 100 90 80 70 60 50 40 30 20 10 0 -10
f1 (ppm)

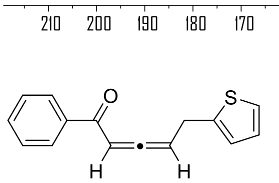
O
S
H
H

1u

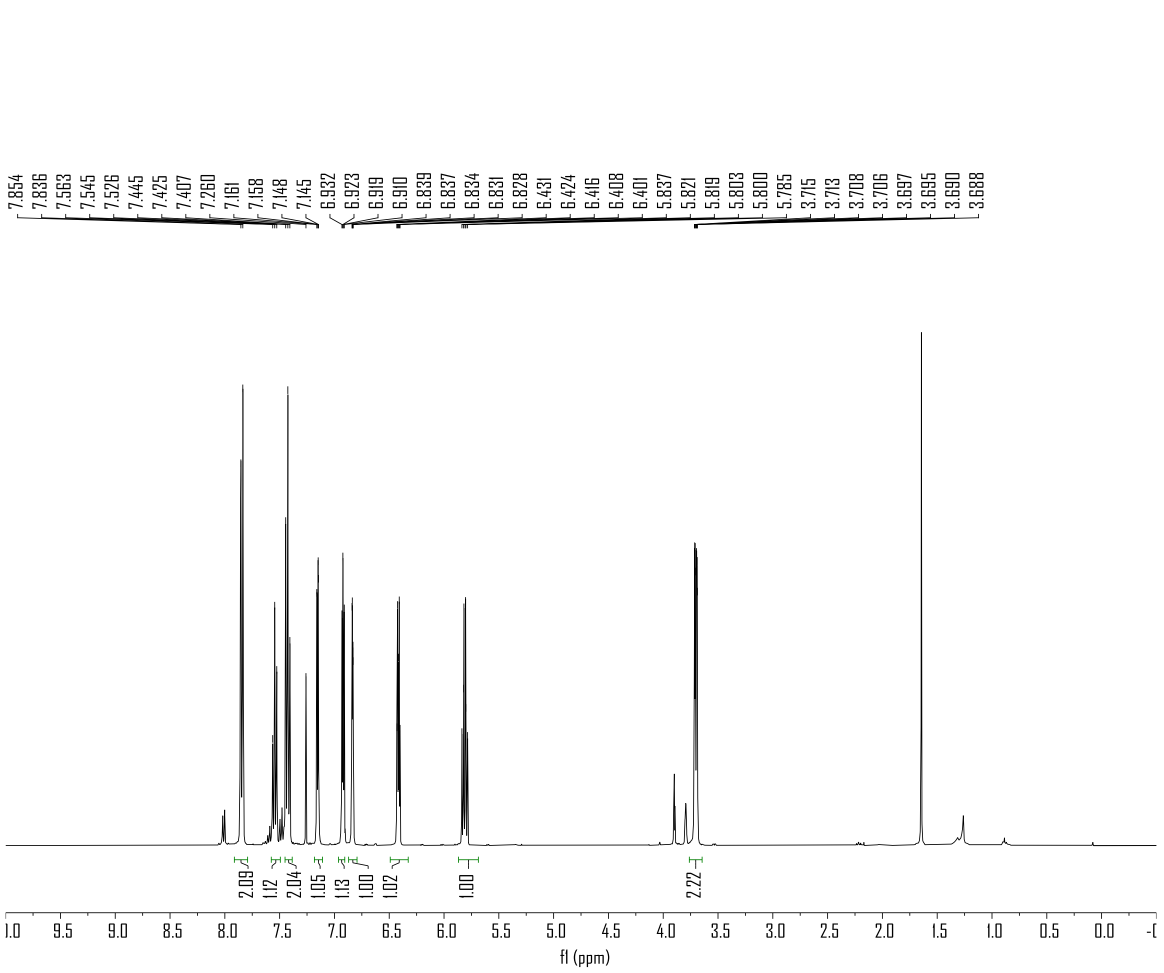
7.854 7.836 7.563 7.545 7.526 7.445 7.425 7.407 7.260 7.161 7.158 7.148 7.145 6.932 6.923 6.919 6.910 6.839 6.837 6.834 6.831 6.828 6.431 6.424 6.416 6.408 6.401 5.837 5.821 5.819 5.803 5.800 5.785 3.715 3.713 3.708 3.706 3.697 3.695 3.690 3.688
2.09 1.12 2.04 1.05 1.13 1.00 1.02 1.00 2.22
f1 (ppm)

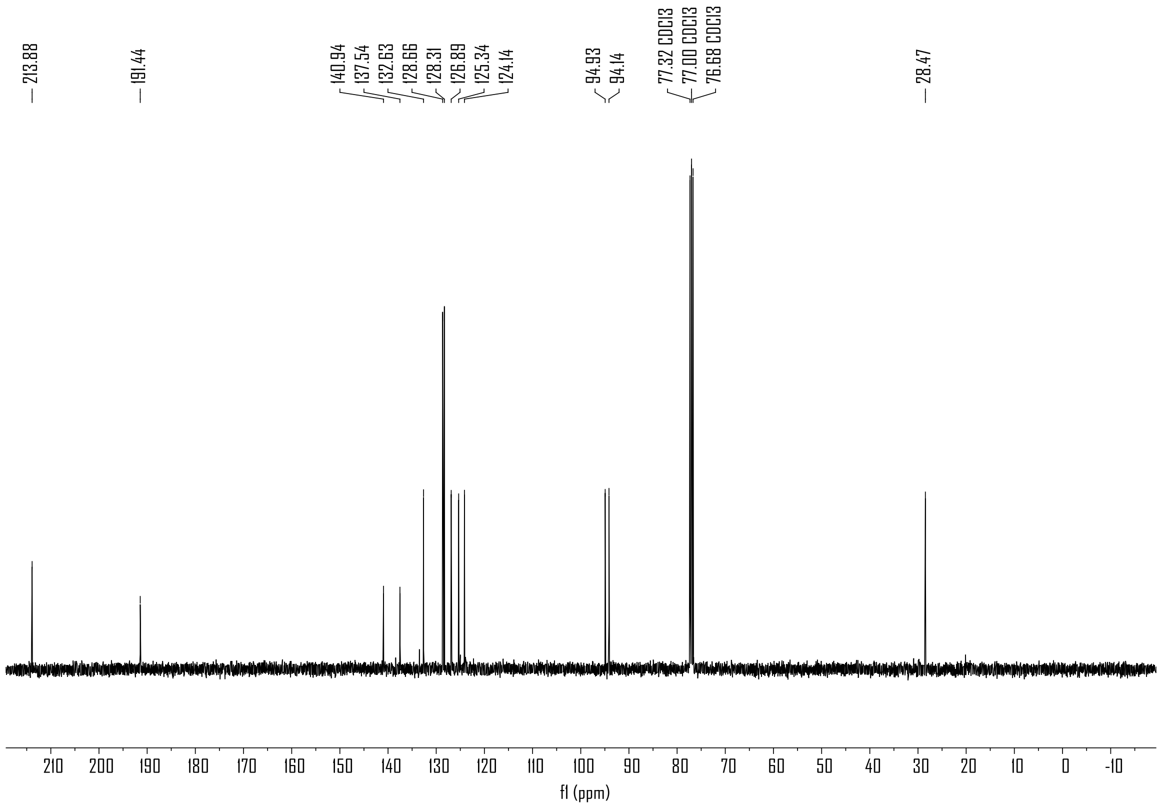
213.88 191.44 140.94 137.54 132.63 128.66 128.31 126.89 125.34 124.14 94.93 94.14 77.32 CDCl3 77.00 CDCl3 76.68 CDCl3 28.47
f1 (ppm)

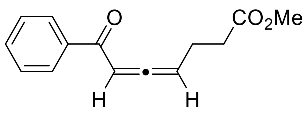
O
CO2Me
H
H

1v

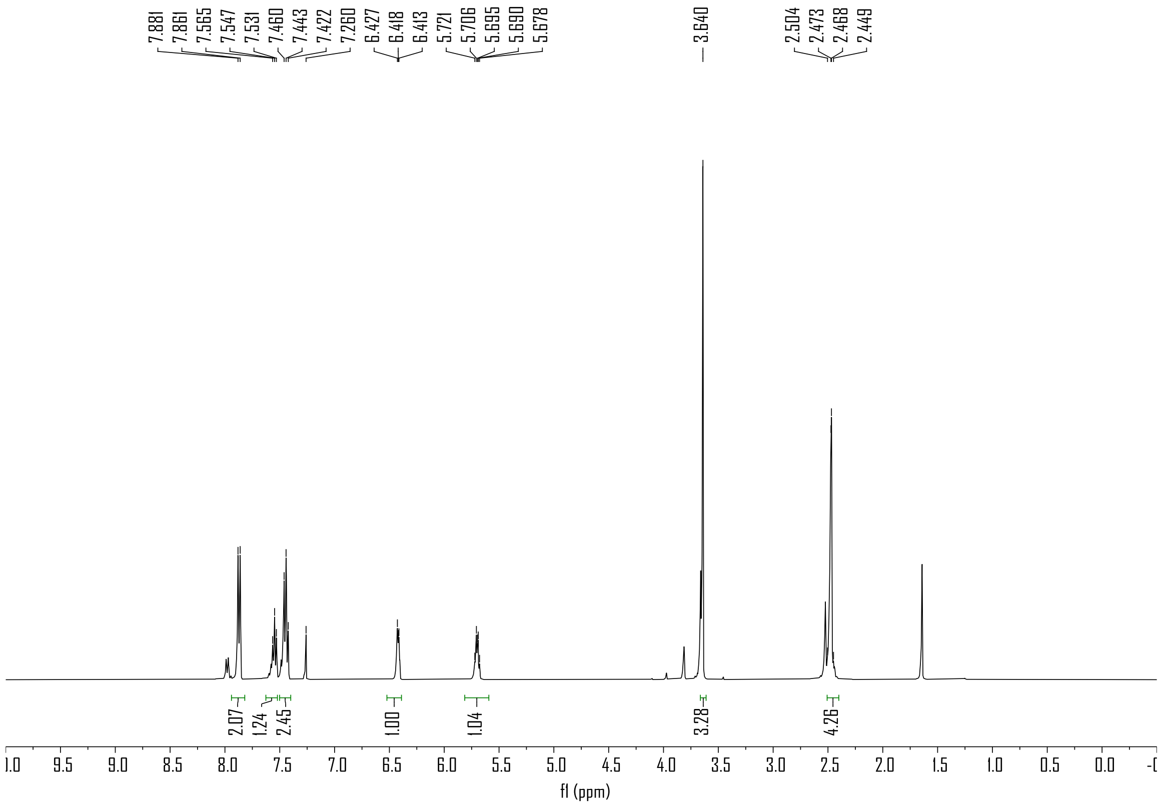
7.881
7.861
7.565
7.547
7.531
7.460
7.443
7.422
7.260
6.427
6.418
6.413
5.721
5.706
5.695
5.690
5.678
3.640
2.504
2.473
2.468
2.449
2.07
1.24
2.45
1.00
1.04
3.28
4.26
f1 (ppm)

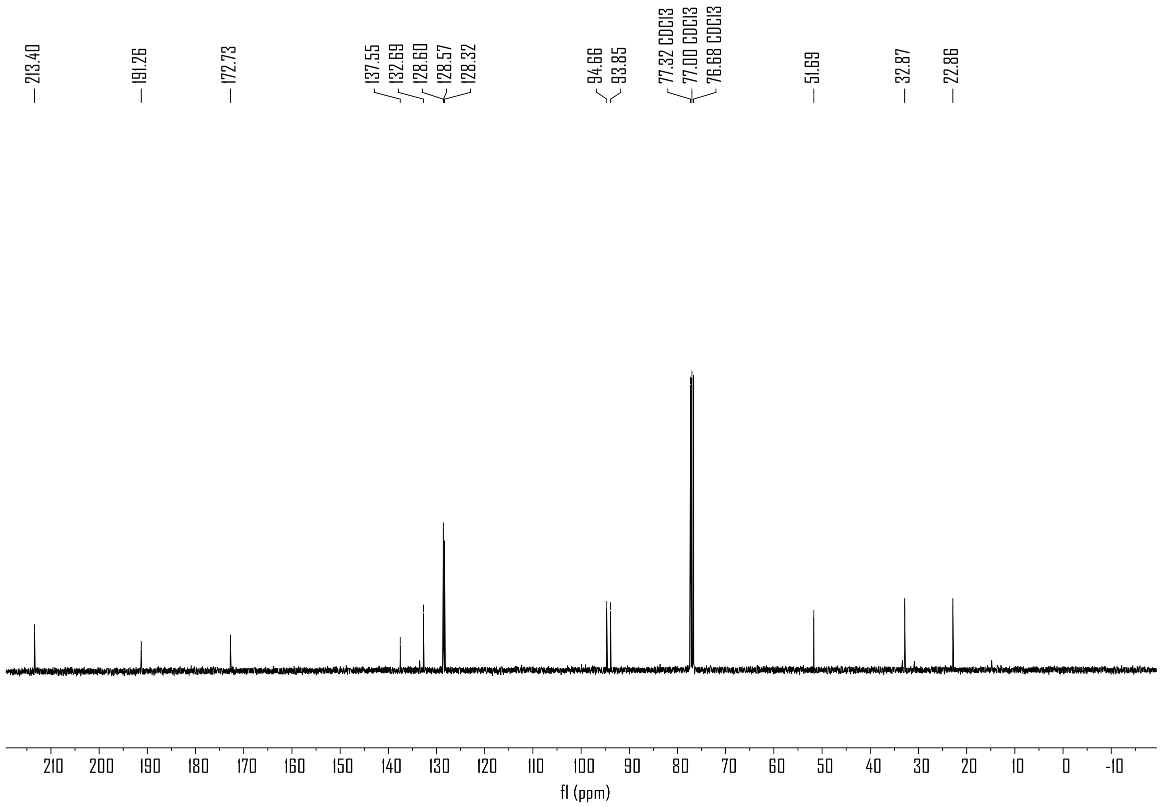
213.40
191.26
172.73
137.55
132.69
128.60
128.57
128.32
94.66
93.85
77.32 CDCl3
77.00 CDCl3
76.68 CDCl3
51.69
32.87
22.86
f1 (ppm)

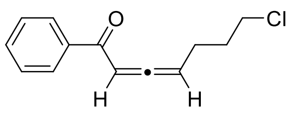
O
Cl
H
H

1w

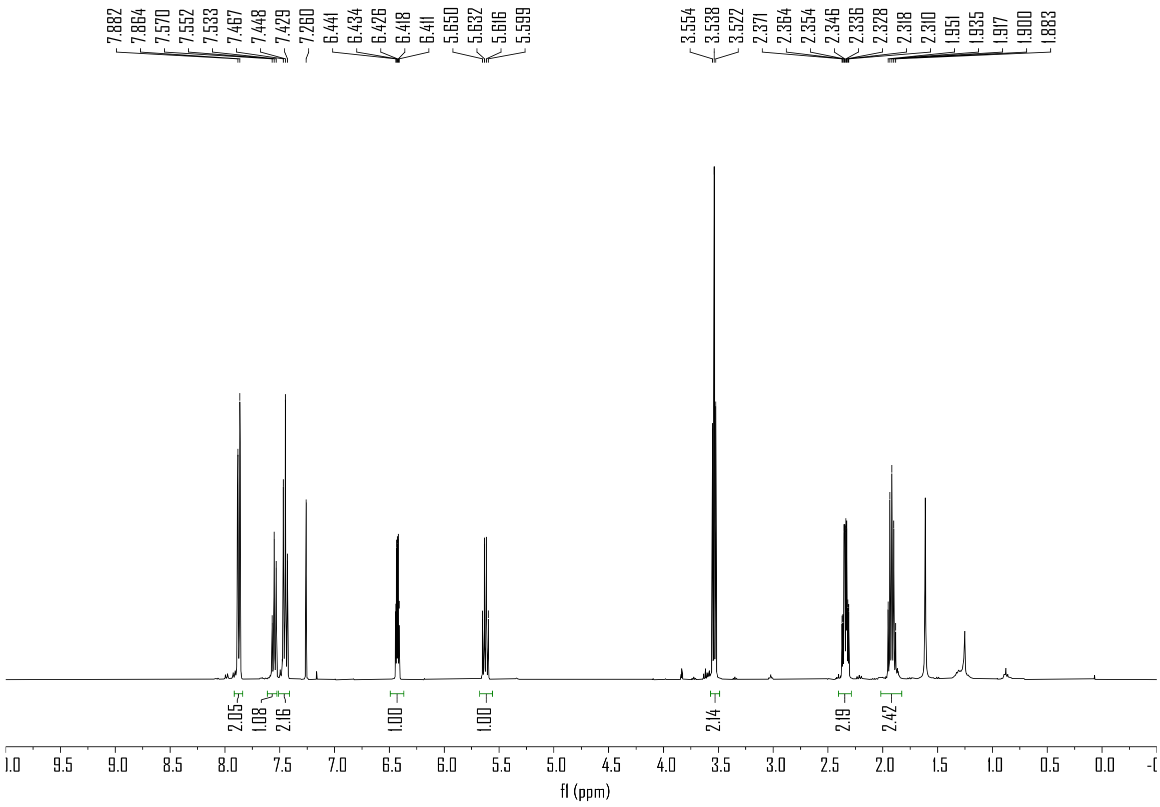
7.882
7.864
7.570
7.552
7.533
7.467
7.448
7.429
7.260
6.441
6.434
6.426
6.418
6.411
5.650
5.632
5.616
5.599
3.554
3.538
3.522
2.371
2.364
2.354
2.346
2.336
2.328
2.318
2.310
1.951
1.935
1.917
1.900
1.883
2.05
1.08
2.16
1.00
1.00
2.14
2.19
2.42
f1 (ppm)

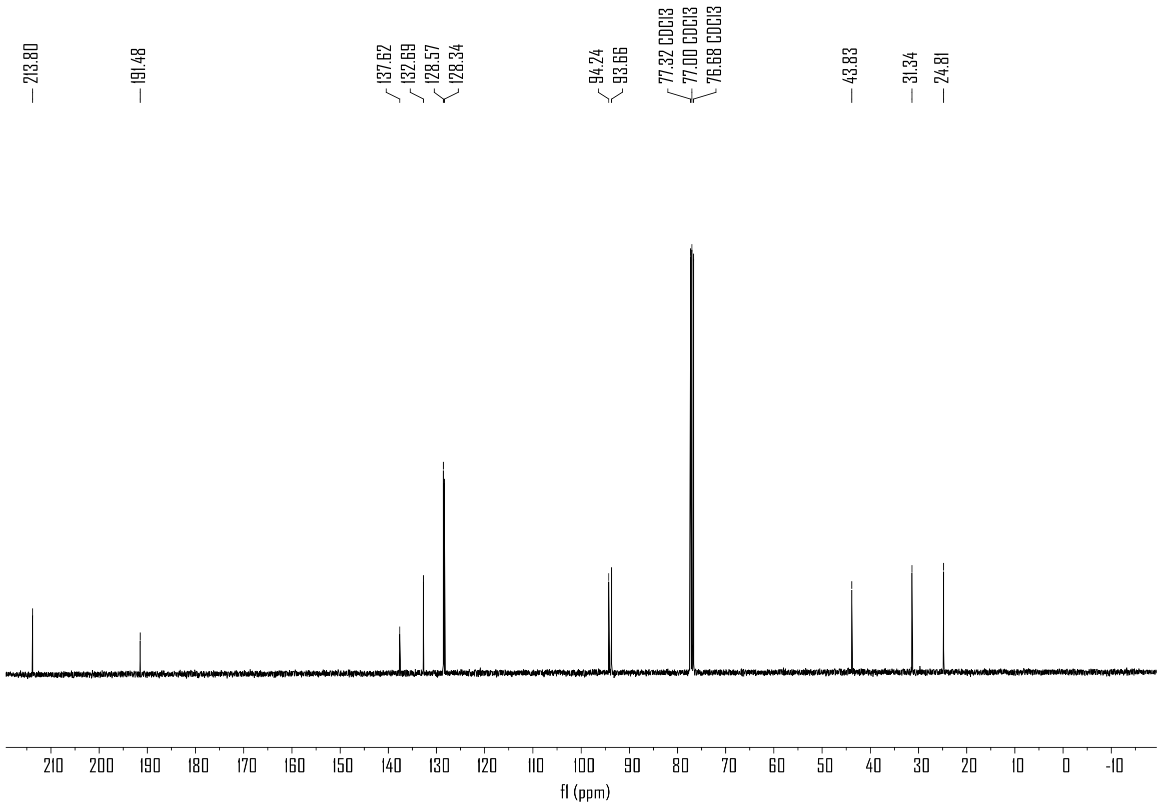
213.80
191.48
137.62
132.69
128.57
128.34
94.24
93.66
77.32 CDCl3
77.00 CDCl3
76.68 CDCl3
43.83
31.34
24.81
f1 (ppm)

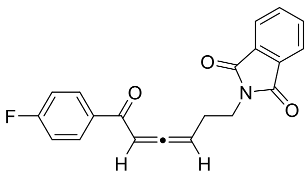

1x

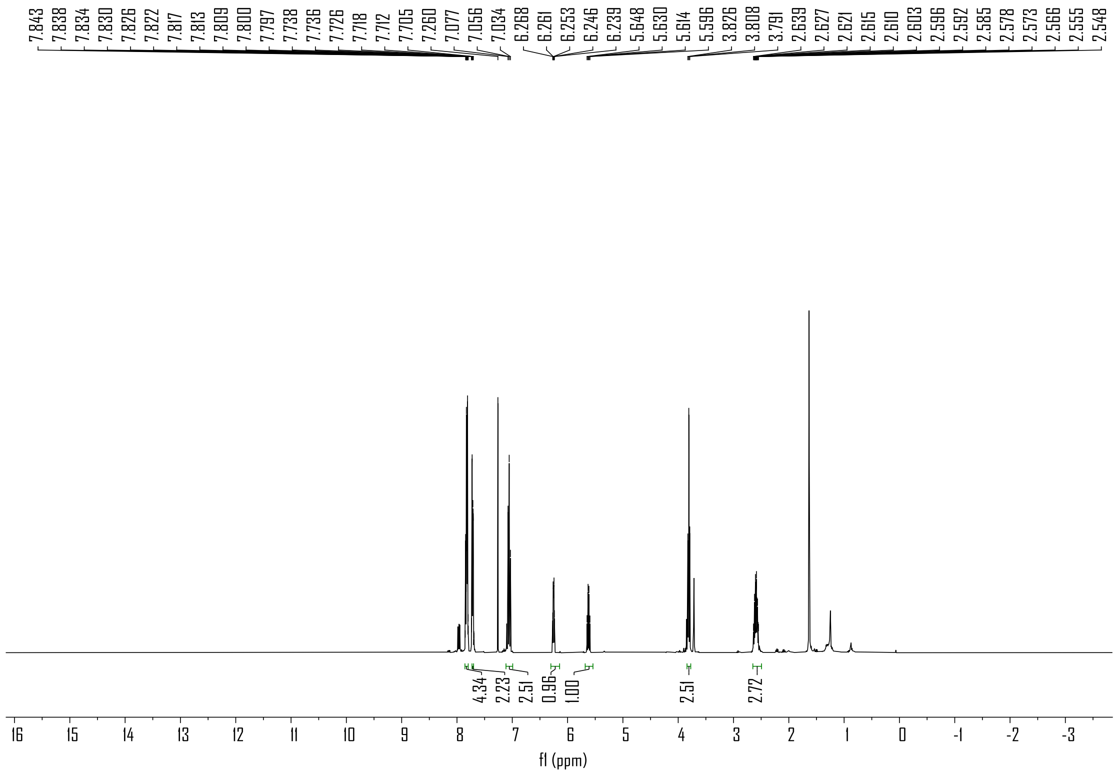
7.843 7.838 7.834 7.830 7.826 7.822 7.817 7.813 7.809 7.800 7.797 7.738 7.736 7.726 7.718 7.712 7.705 7.260 7.077 7.056 7.034 6.268 6.261 6.253 6.246 6.239 5.648 5.630 5.614 5.596 3.826 3.808 3.791 2.639 2.627 2.621 2.615 2.610 2.603 2.596 2.592 2.585 2.578 2.573 2.566 2.555 2.548
4.34 2.23 2.51 0.96 1.00 2.51 2.72
16 15 14 13 12 11 10 9 8 7 6 5 4 3 2 1 0 -1 -2 -3
f1 (ppm)

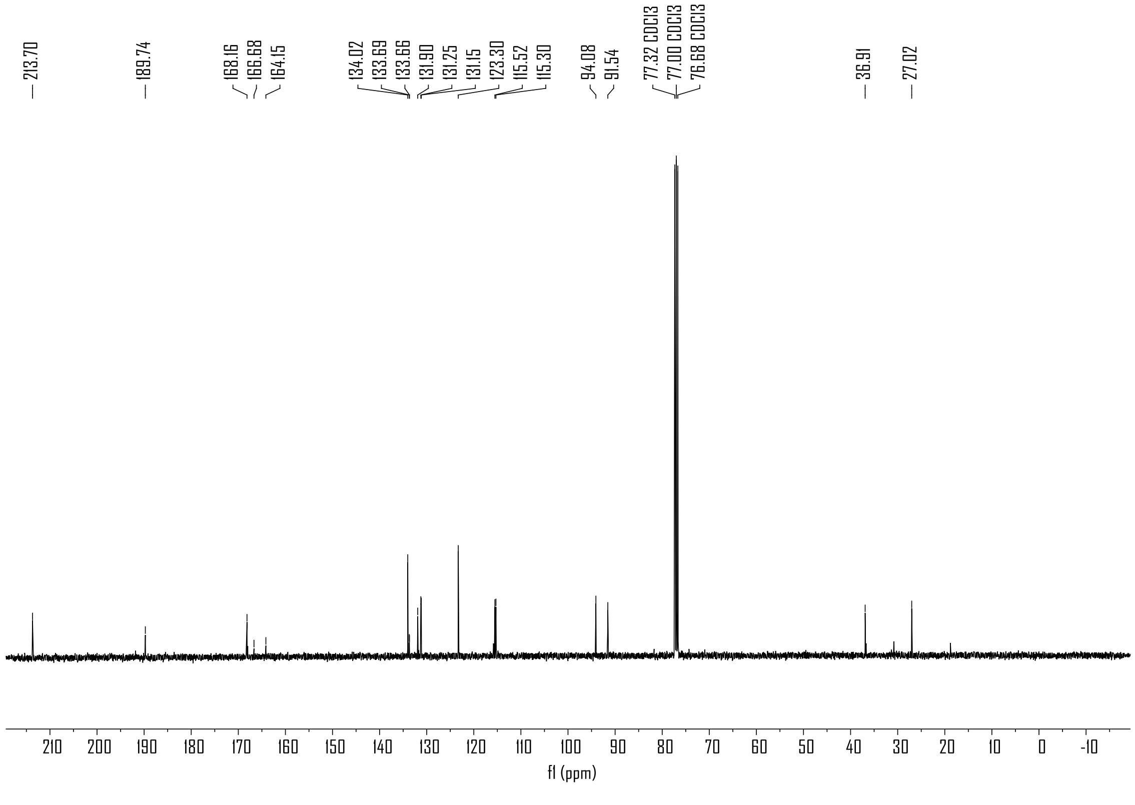
213.70 189.74 168.16 166.68 164.15 134.02 133.69 133.66 131.90 131.25 131.15 123.30 115.52 115.30 94.08 91.54 77.32 CDCl3 77.00 CDCl3 76.68 CDCl3 36.91 27.02
210 200 190 180 170 160 150 140 130 120 110 100 90 80 70 60 50 40 30 20 10 0 -10
f1 (ppm)

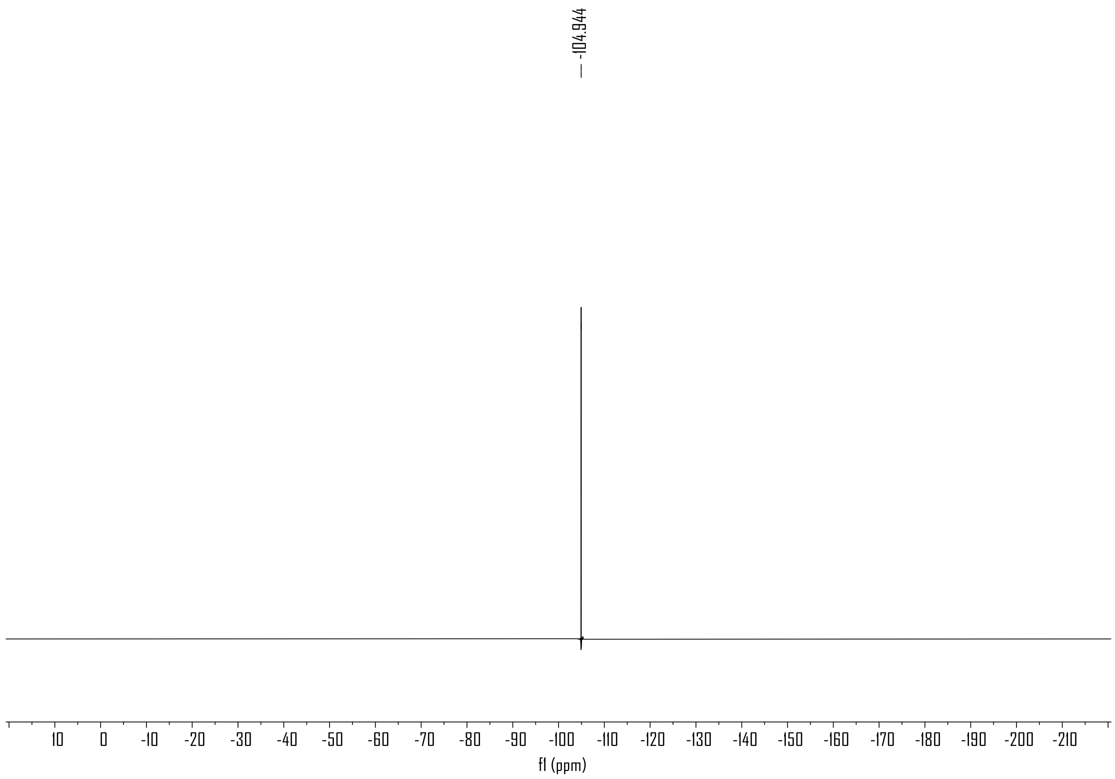
-104.944
10 0 -10 -20 -30 -40 -50 -60 -70 -80 -90 -100 -110 -120 -130 -140 -150 -160 -170 -180 -190 -200 -210
f1 (ppm)

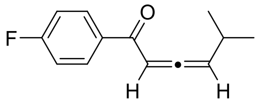
F
O
H
H

1y

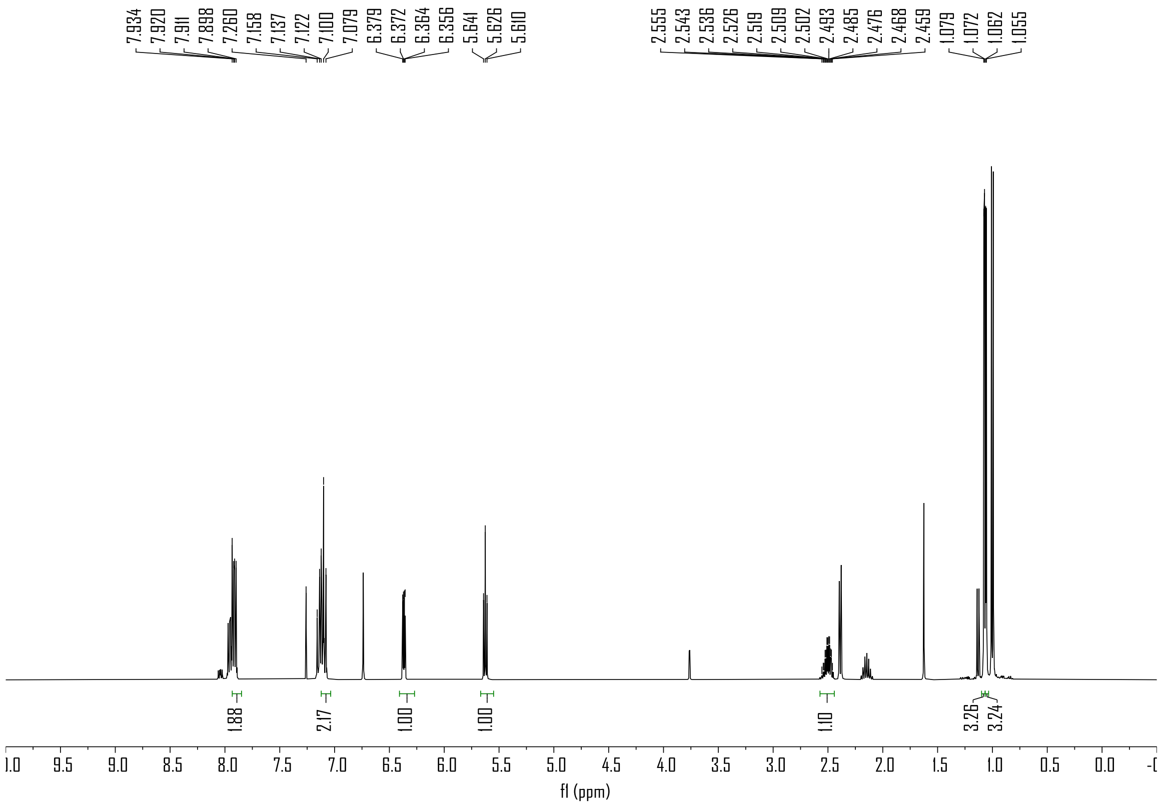
7.934
7.920
7.911
7.898
7.260
7.158
7.137
7.122
7.100
7.079
6.379
6.372
6.364
6.356
5.641
5.626
5.610
2.555
2.543
2.536
2.526
2.519
2.509
2.502
2.493
2.485
2.476
2.468
2.459
1.079
1.072
1.062
1.055
1.88
2.17
1.00
1.00
1.10
3.26
3.24
f1 (ppm)

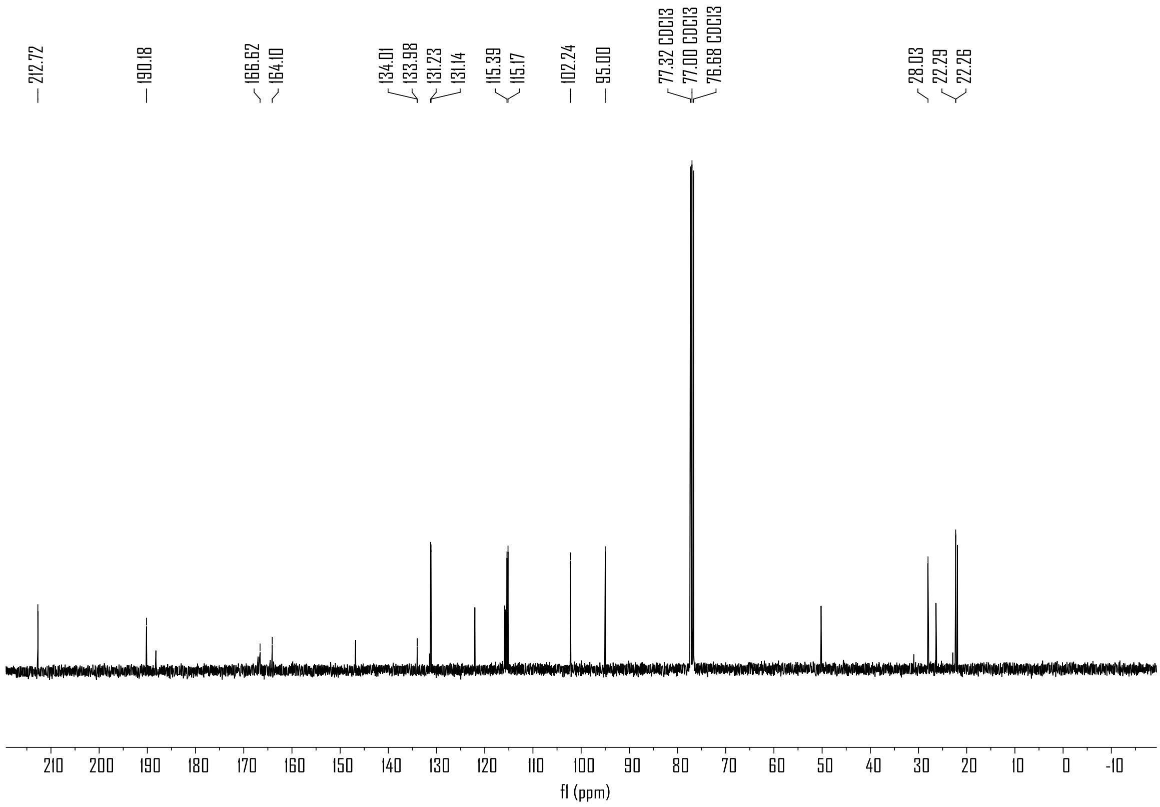
212.72
190.18
166.62
164.10
134.01
133.98
131.23
131.14
115.39
115.17
102.24
95.00
77.32 CDCl3
77.00 CDCl3
76.68 CDCl3
28.03
22.29
22.26
f1 (ppm)

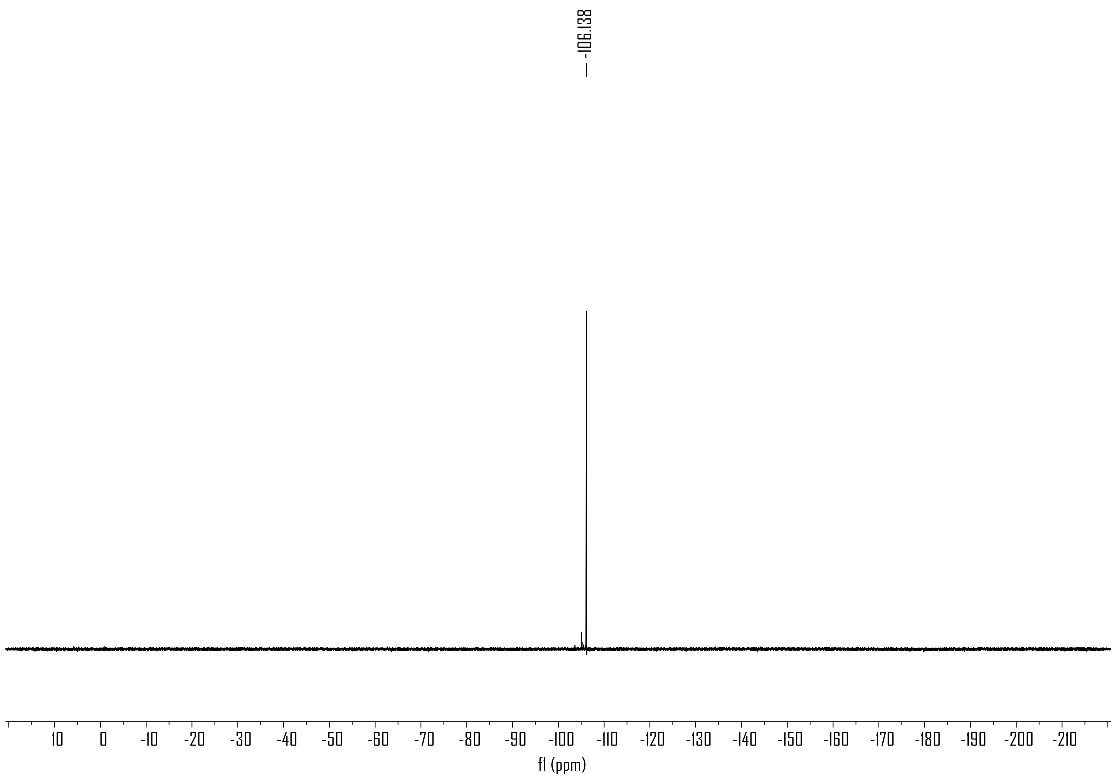

-106.138
10 0 -10 -20 -30 -40 -50 -60 -70 -80 -90 -100 -110 -120 -130 -140 -150 -160 -170 -180 -190 -200 -210
f1 (ppm)

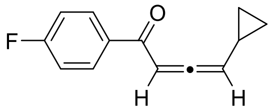

F
O
H
H

1z

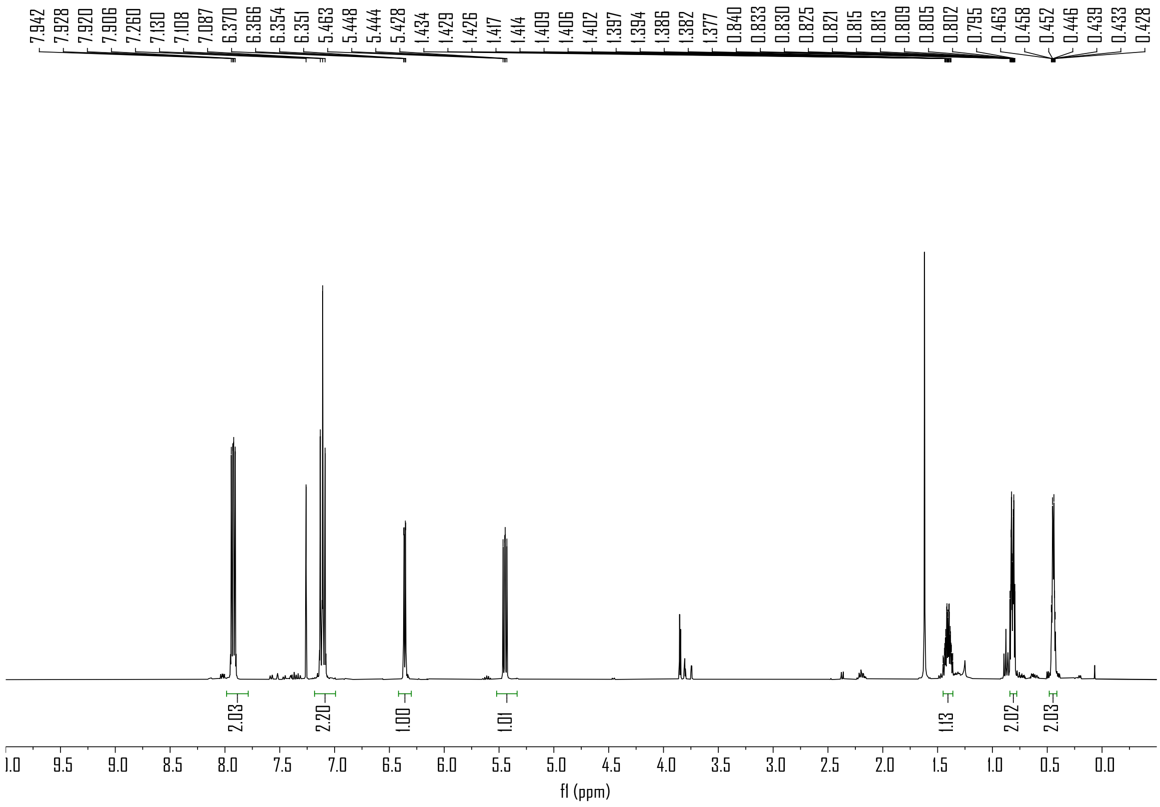
7.942
7.928
7.920
7.906
7.260
7.130
7.108
7.087
6.370
6.366
6.354
6.351
5.463
5.448
5.444
5.428
1.434
1.429
1.426
1.417
1.414
1.409
1.406
1.402
1.397
1.394
1.386
1.382
1.377
0.840
0.833
0.830
0.825
0.821
0.815
0.813
0.809
0.805
0.802
0.795
0.463
0.458
0.452
0.446
0.439
0.433
0.428
2.03
2.20
1.00
1.01
1.13
2.02
2.03
f1 (ppm)

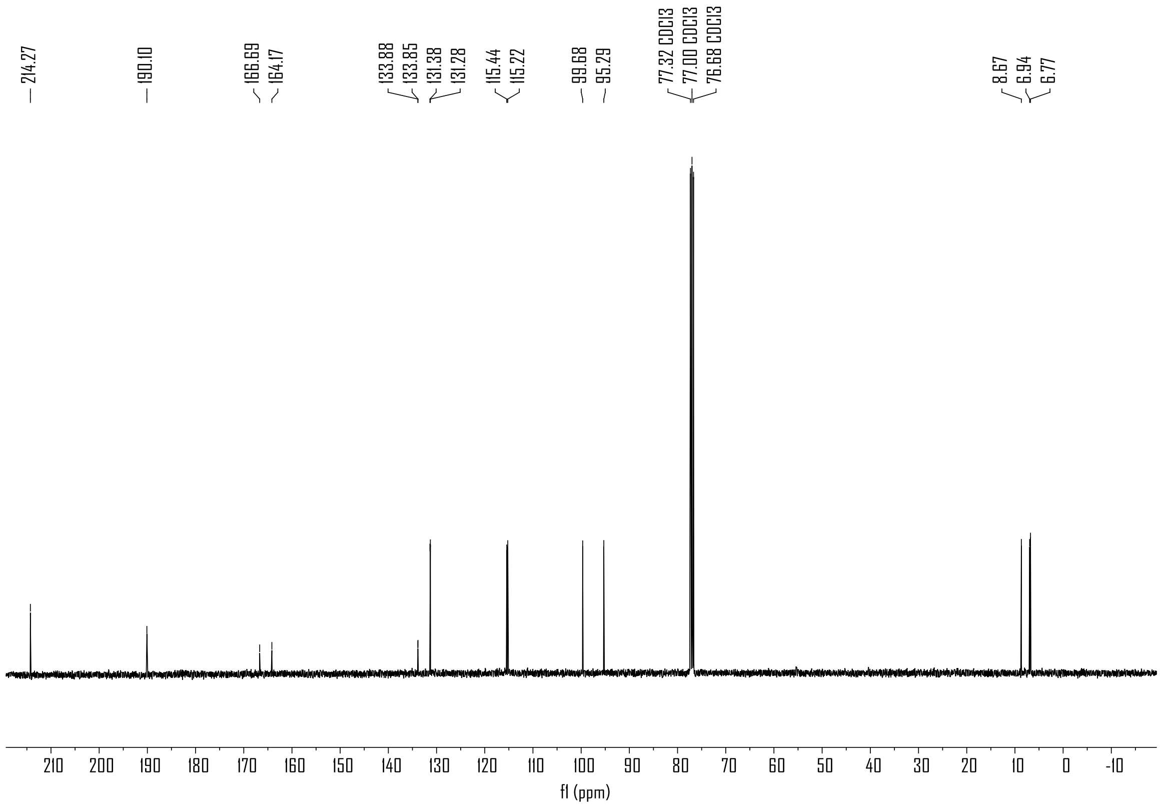
214.27
190.10
166.69
164.17
133.88
133.85
131.38
131.28
115.44
115.22
99.68
95.29
77.32 CDCl3
77.00 CDCl3
76.68 CDCl3
8.67
6.94
6.77
f1 (ppm)

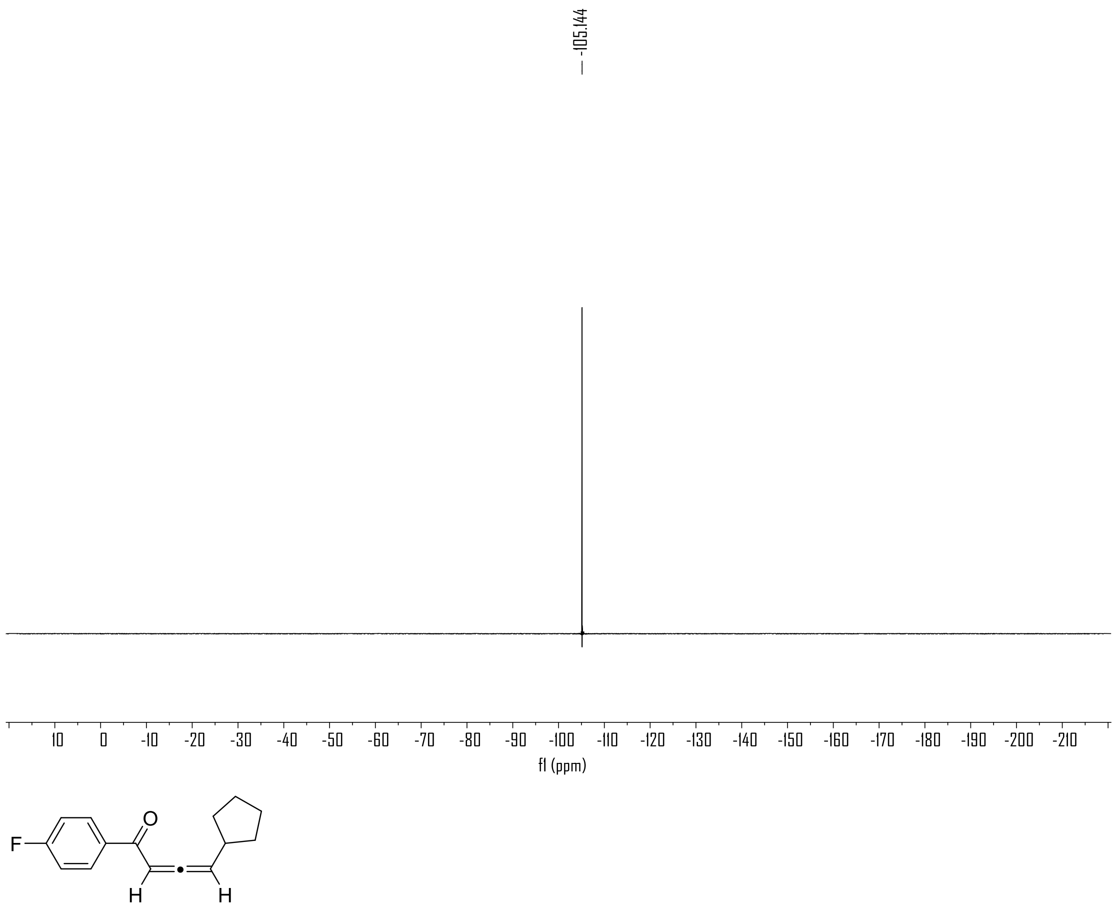
-105.144
10 0 -10 -20 -30 -40 -50 -60 -70 -80 -90 -100 -110 -120 -130 -140 -150 -160 -170 -180 -190 -200 -210
f1 (ppm)
F
O
H
H

1aa

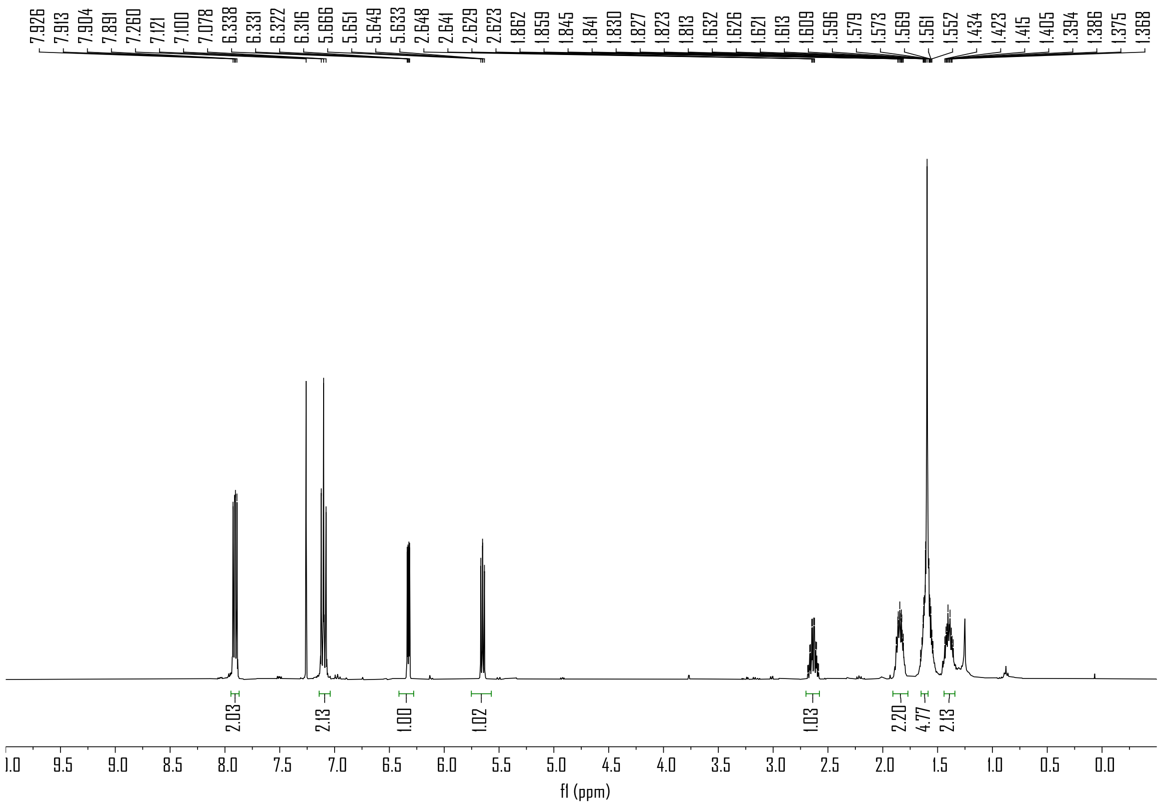
7.926
7.913
7.904
7.891
7.260
7.121
7.100
7.078
6.338
6.331
6.322
6.316
5.666
5.651
5.649
5.633
2.648
2.641
2.629
2.623
1.862
1.859
1.845
1.841
1.830
1.827
1.823
1.813
1.632
1.626
1.621
1.613
1.609
1.596
1.579
1.573
1.569
1.561
1.552
1.434
1.423
1.415
1.405
1.394
1.386
1.375
1.368
2.03
2.13
1.00
1.02
1.03
2.20
4.77
2.13
f1 (ppm)

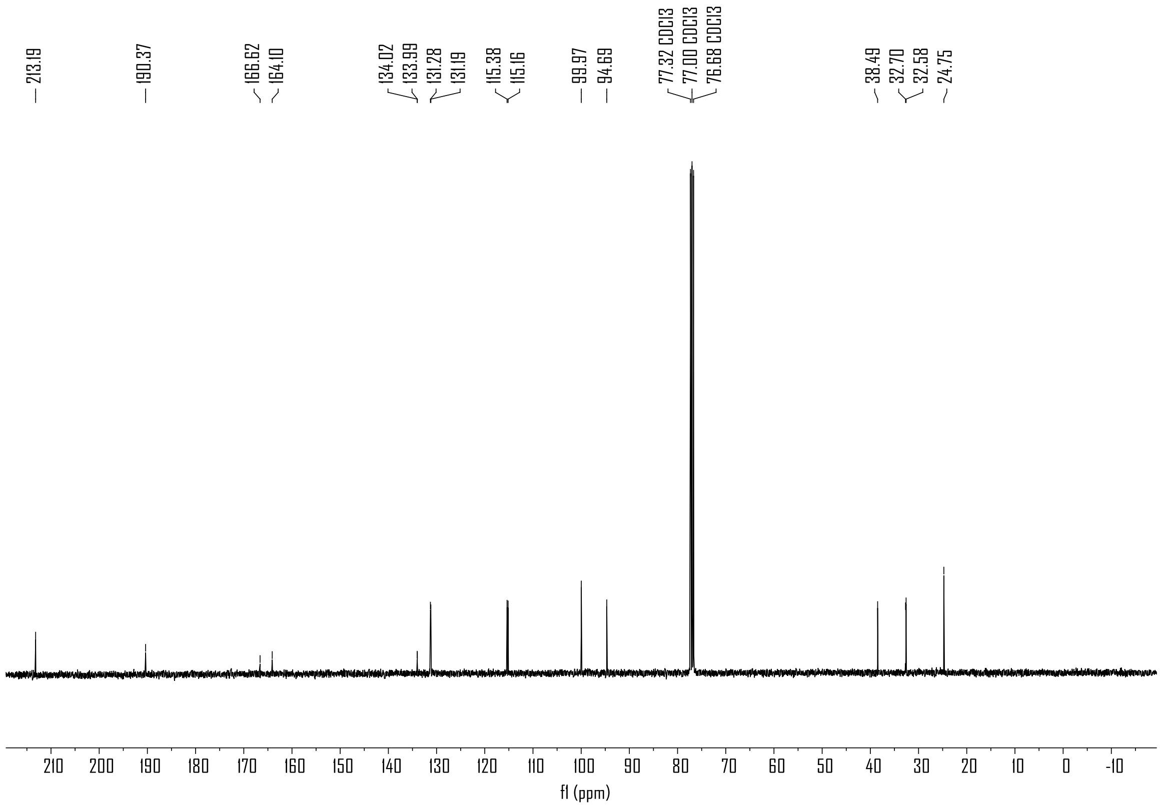
213.19
190.37
166.62
164.10
134.02
133.99
131.28
131.19
115.38
115.16
99.97
94.69
77.32 CDCl3
77.00 CDCl3
76.68 CDCl3
38.49
32.70
32.58
24.75
f1 (ppm)

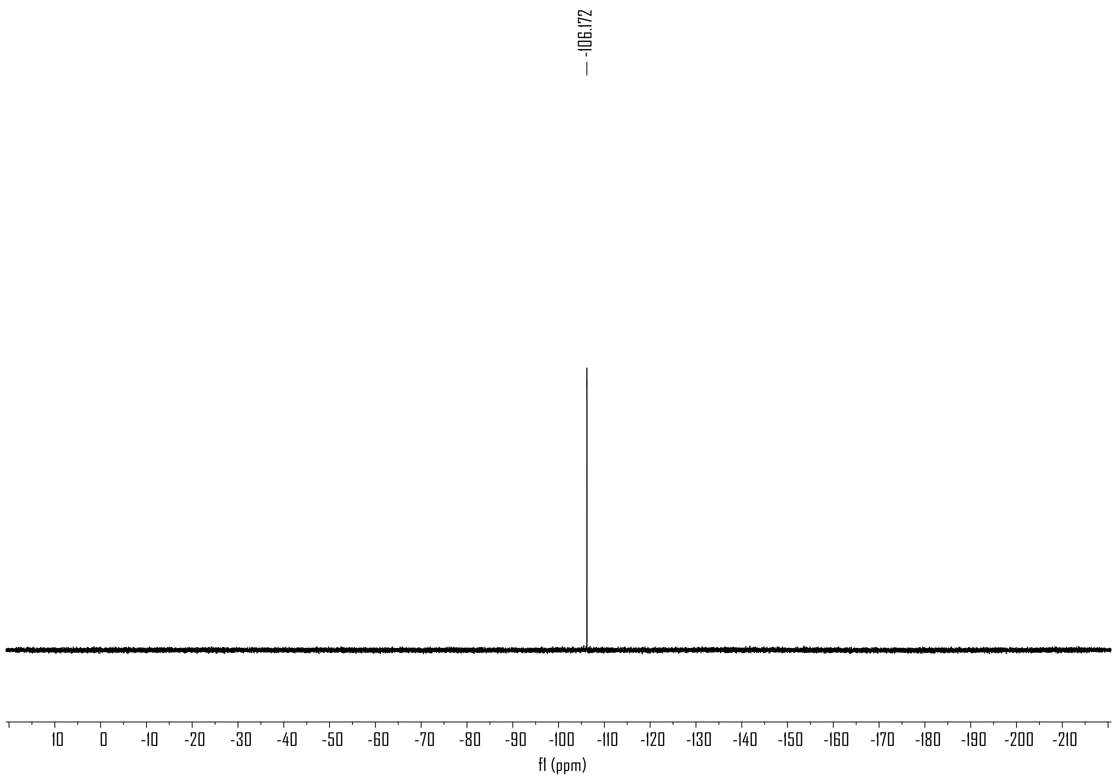
-106.172
10 0 -10 -20 -30 -40 -50 -60 -70 -80 -90 -100 -110 -120 -130 -140 -150 -160 -170 -180 -190 -200 -210
f1 (ppm)

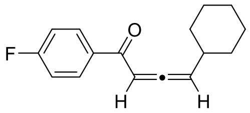
F
O
H
H

1ab

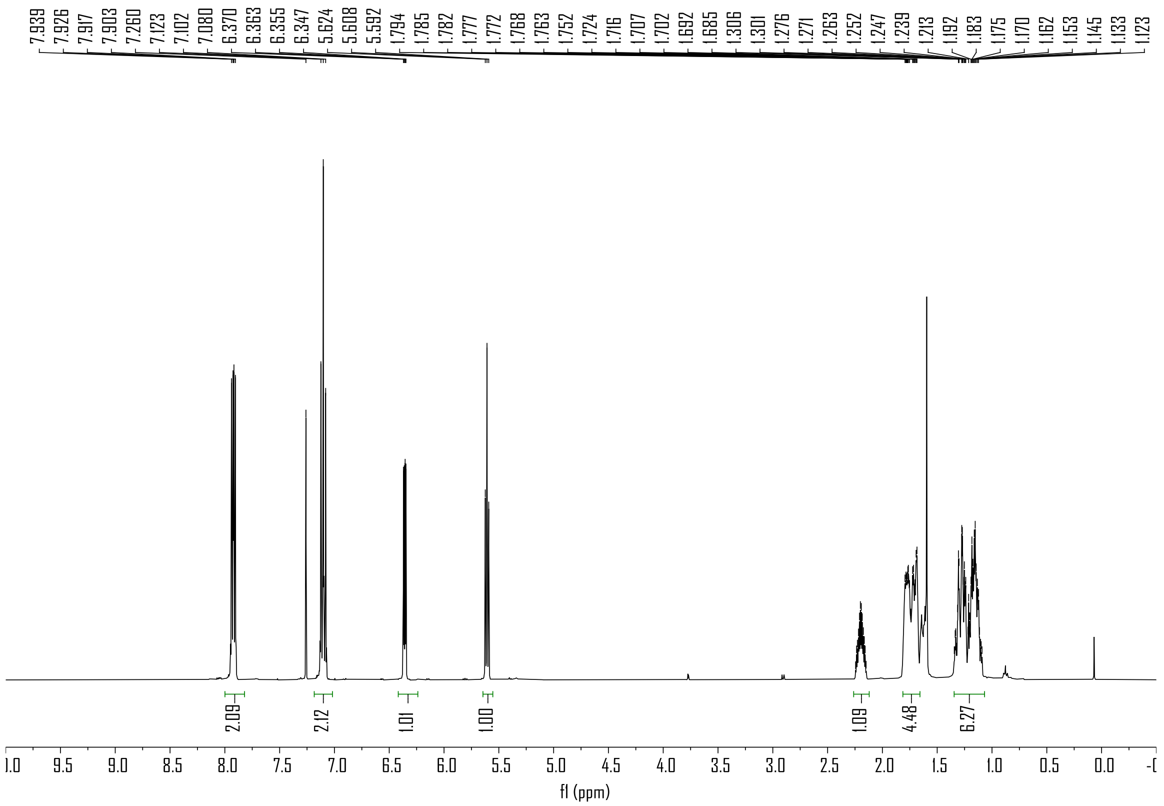
7.939
7.926
7.917
7.903
7.260
7.123
7.102
7.080
6.370
6.363
6.355
6.347
5.624
5.608
5.592
1.794
1.785
1.782
1.777
1.772
1.768
1.763
1.752
1.724
1.716
1.707
1.702
1.692
1.685
1.306
1.301
1.276
1.271
1.263
1.252
1.247
1.239
1.213
1.192
1.183
1.175
1.170
1.162
1.153
1.145
1.133
1.123
2.09
2.12
1.01
1.00
1.09
4.48
6.27
f1 (ppm)

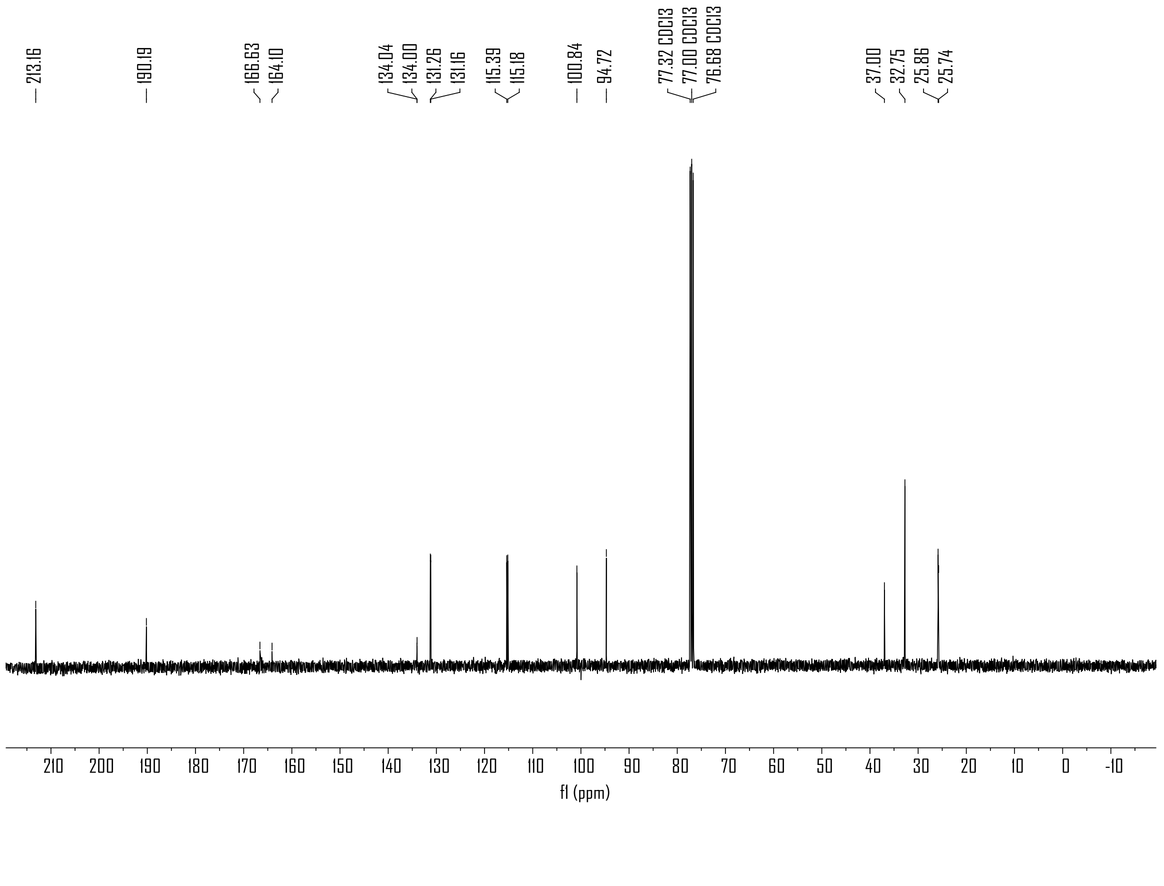
213.16
190.19
166.63
164.10
134.04
134.00
131.26
131.16
115.39
115.18
100.84
94.72
77.32 CDCl3
77.00 CDCl3
76.68 CDCl3
37.00
32.75
25.86
25.74
f1 (ppm)

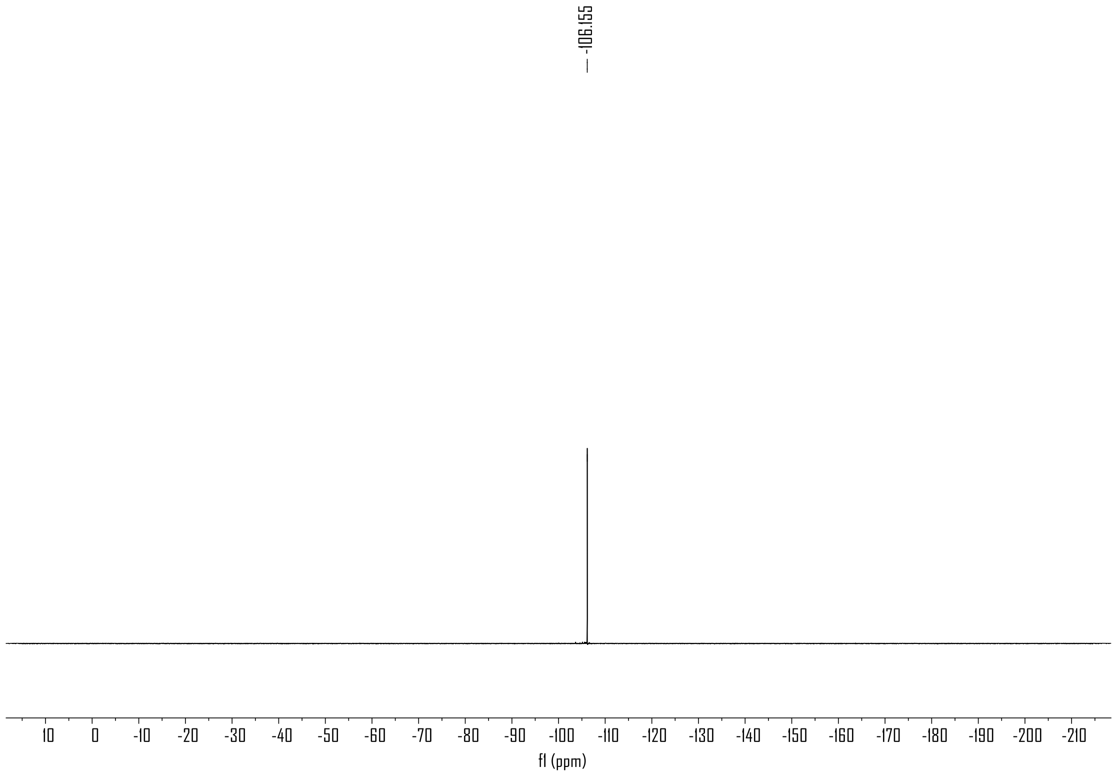
-106.155
10 0 -10 -20 -30 -40 -50 -60 -70 -80 -90 -100 -110 -120 -130 -140 -150 -160 -170 -180 -190 -200 -210
f1 (ppm)

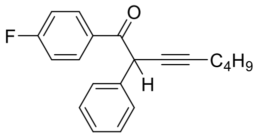
F
O
C4H9
H

(*rac*)-**3a'**

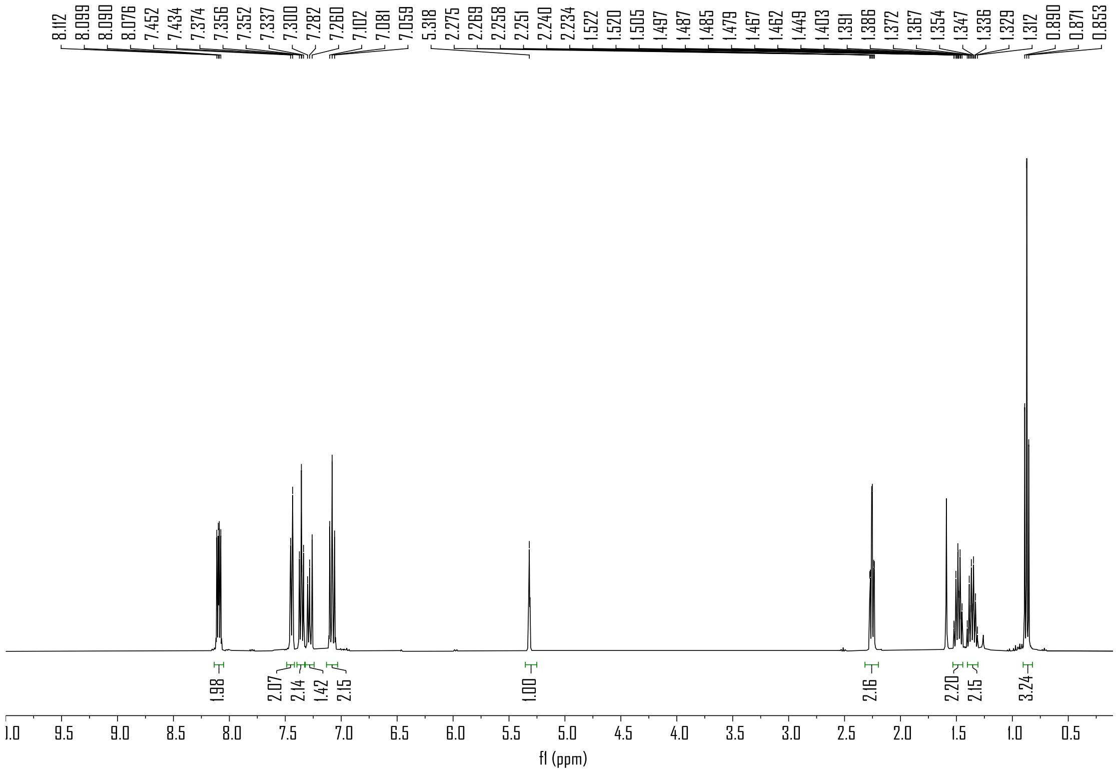
8.112 8.099 8.090 8.076 7.452 7.434 7.374 7.356 7.352 7.337 7.300 7.282 7.260 7.102 7.081 7.059 5.318 2.275 2.269 2.258 2.251 2.240 2.234 1.522 1.520 1.505 1.497 1.487 1.485 1.479 1.467 1.462 1.449 1.403 1.391 1.386 1.372 1.367 1.354 1.347 1.336 1.329 1.312 0.890 0.871 0.853
1.98 2.07 2.14 1.42 2.15 1.00 2.16 2.20 2.15 3.24
1.0 9.5 9.0 8.5 8.0 7.5 7.0 6.5 6.0 5.5 5.0 4.5 4.0 3.5 3.0 2.5 2.0 1.5 1.0 0.5
f1 (ppm)

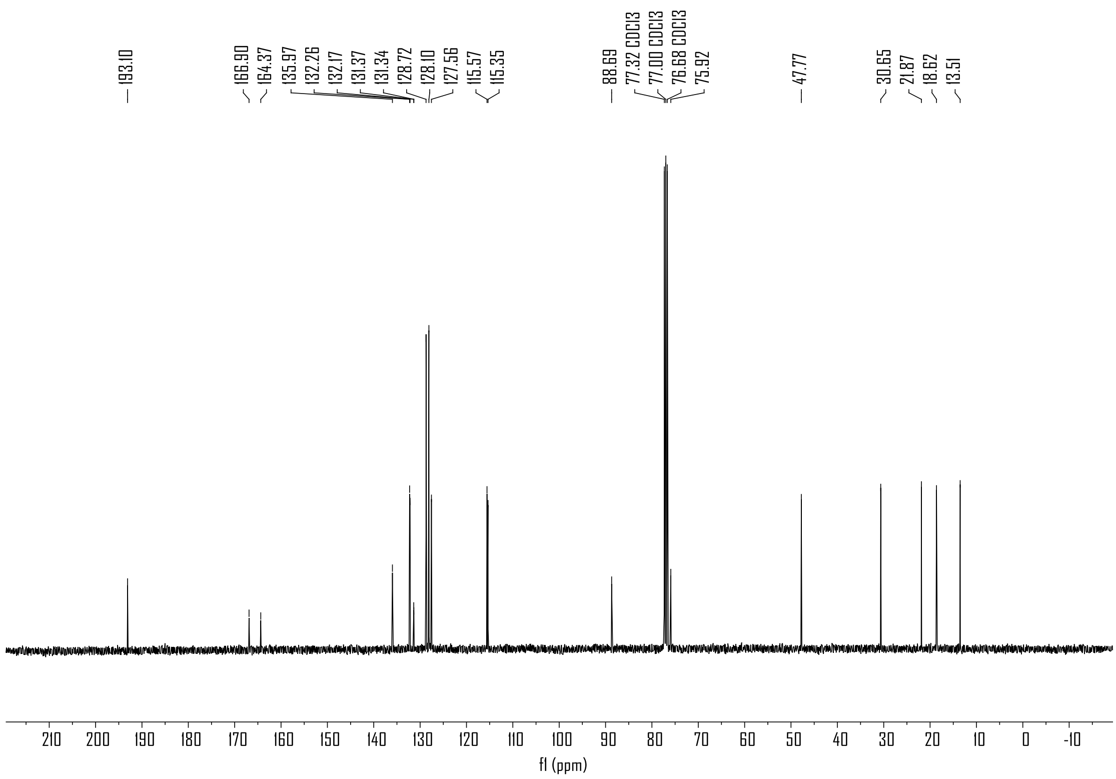
193.10
166.90
164.37
135.97
132.26
132.17
131.37
131.34
128.72
128.10
127.56
115.57
115.35
88.69
77.32 CDCl3
77.00 CDCl3
76.68 CDCl3
75.92
47.77
30.65
21.87
18.62
13.51
210 200 190 180 170 160 150 140 130 120 110 100 90 80 70 60 50 40 30 20 10 0 -10
f1 (ppm)

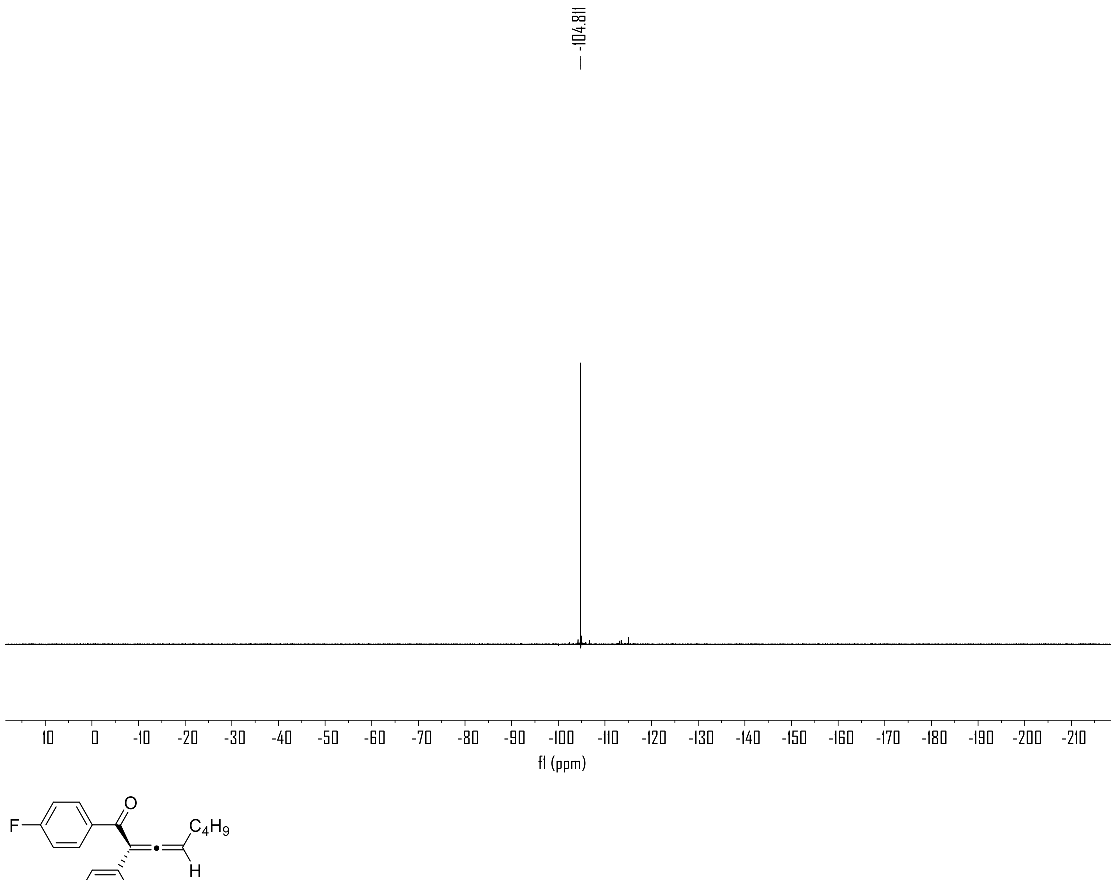
-104.811
10 0 -10 -20 -30 -40 -50 -60 -70 -80 -90 -100 -110 -120 -130 -140 -150 -160 -170 -180 -190 -200 -210
f1 (ppm)

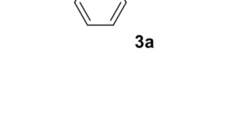
F
O
C4H9
H

3a

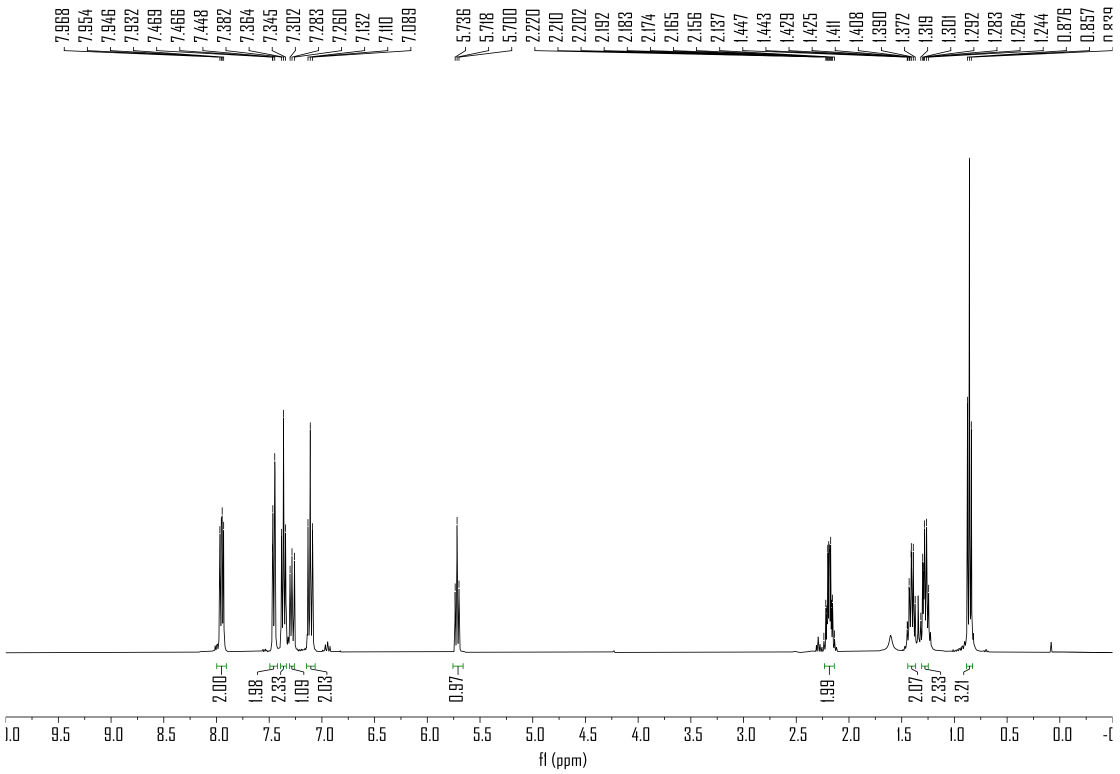

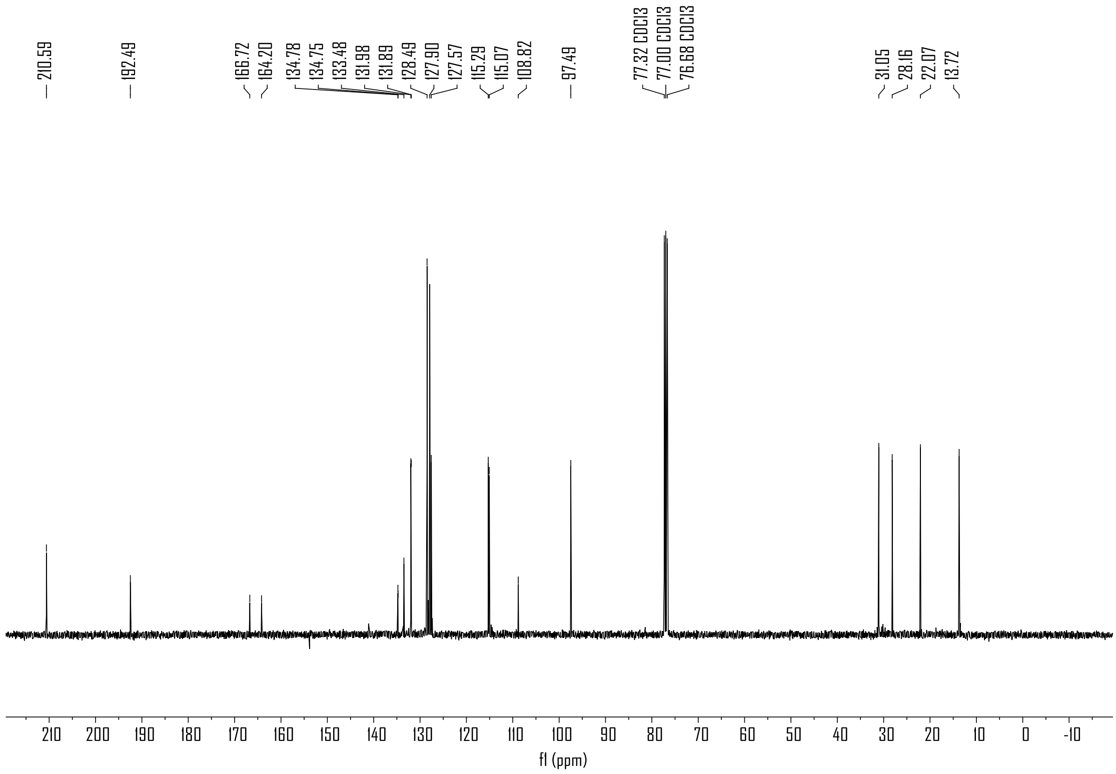
210.59
192.49
166.72
164.20
134.78
134.75
133.48
131.98
131.89
128.49
127.90
127.57
115.29
115.07
108.82
97.49
77.32 CDCl3
77.00 CDCl3
76.68 CDCl3
31.05
28.16
22.07
13.72
210 200 190 180 170 160 150 140 130 120 110 100 90 80 70 60 50 40 30 20 10 0 -10
f1 (ppm)

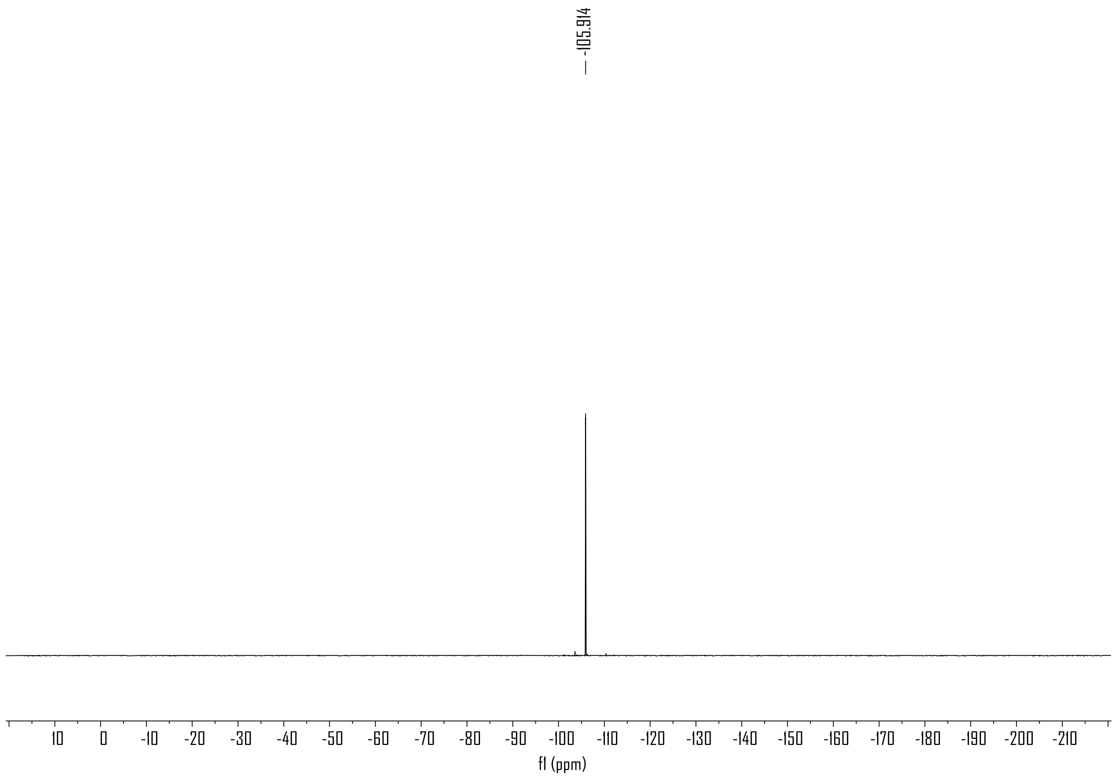
-105.914
10 0 -10 -20 -30 -40 -50 -60 -70 -80 -90 -100 -110 -120 -130 -140 -150 -160 -170 -180 -190 -200 -210
f1 (ppm)

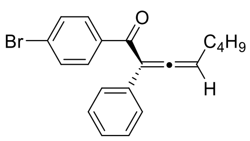
Br
O
C4H9
H

3b

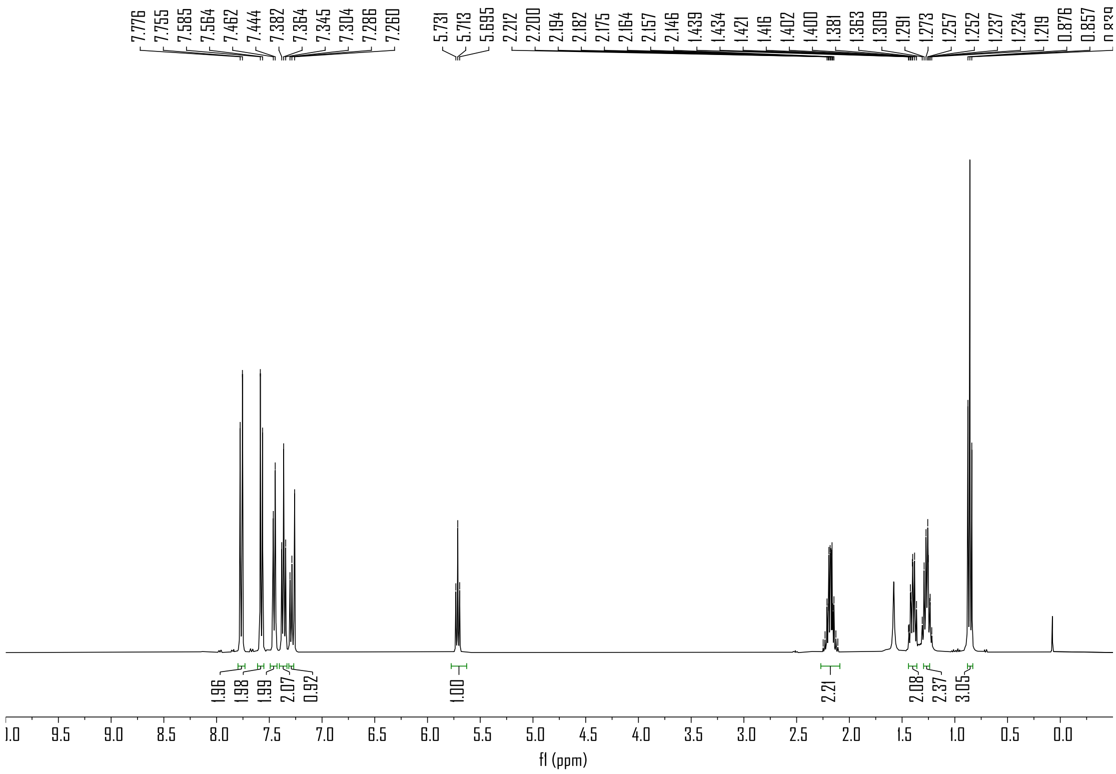
7.776
7.755
7.585
7.564
7.462
7.444
7.382
7.364
7.345
7.304
7.286
7.260
5.731
5.713
5.695
2.212
2.200
2.194
2.182
2.175
2.164
2.157
2.146
1.439
1.434
1.421
1.416
1.402
1.400
1.381
1.363
1.309
1.291
1.273
1.257
1.252
1.237
1.234
1.219
0.876
0.857
1.96
1.98
1.99
2.07
0.92
1.00
2.21
2.08
2.37
3.05
fl (ppm)

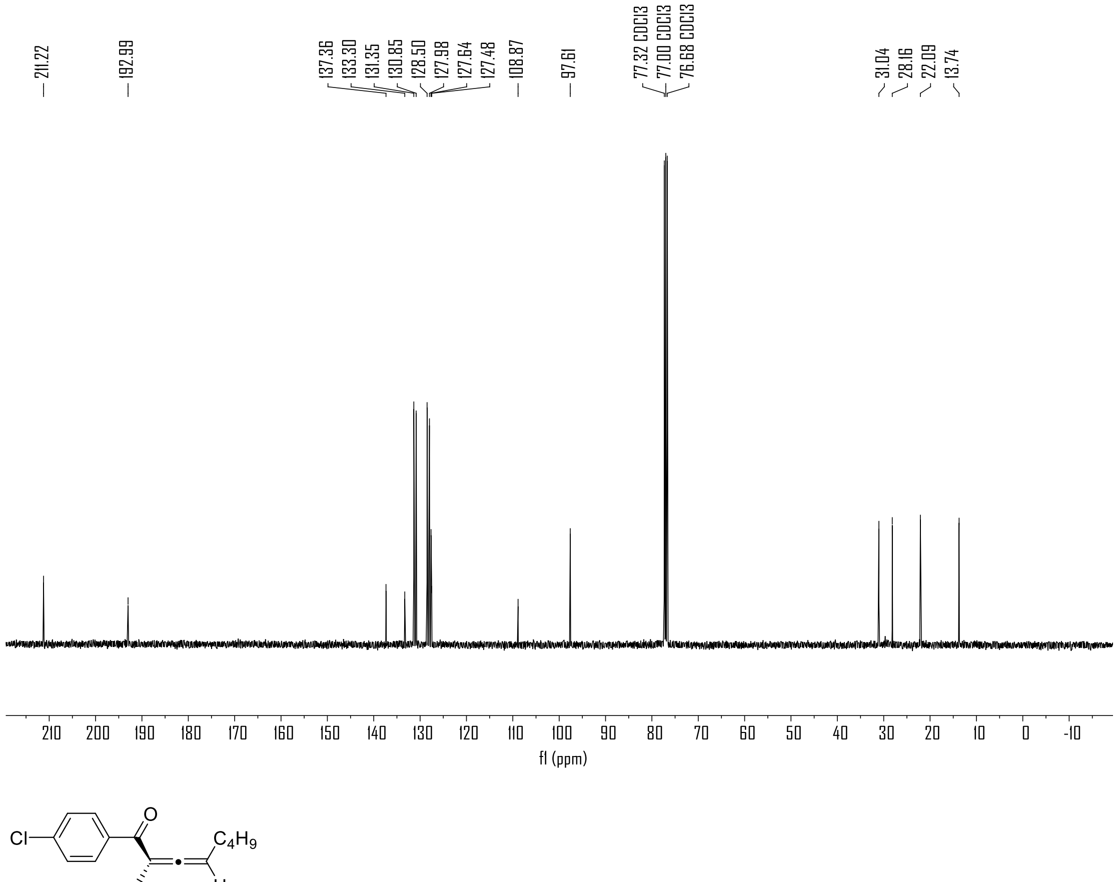
211.22
192.99
137.36
133.30
131.35
130.85
128.50
127.98
127.64
127.48
108.87
97.61
77.32 CDCl3
77.00 CDCl3
76.68 CDCl3
31.04
28.16
22.09
13.74
fl (ppm)

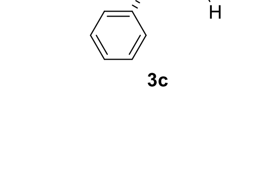
Cl
O
C4H9
H

3c

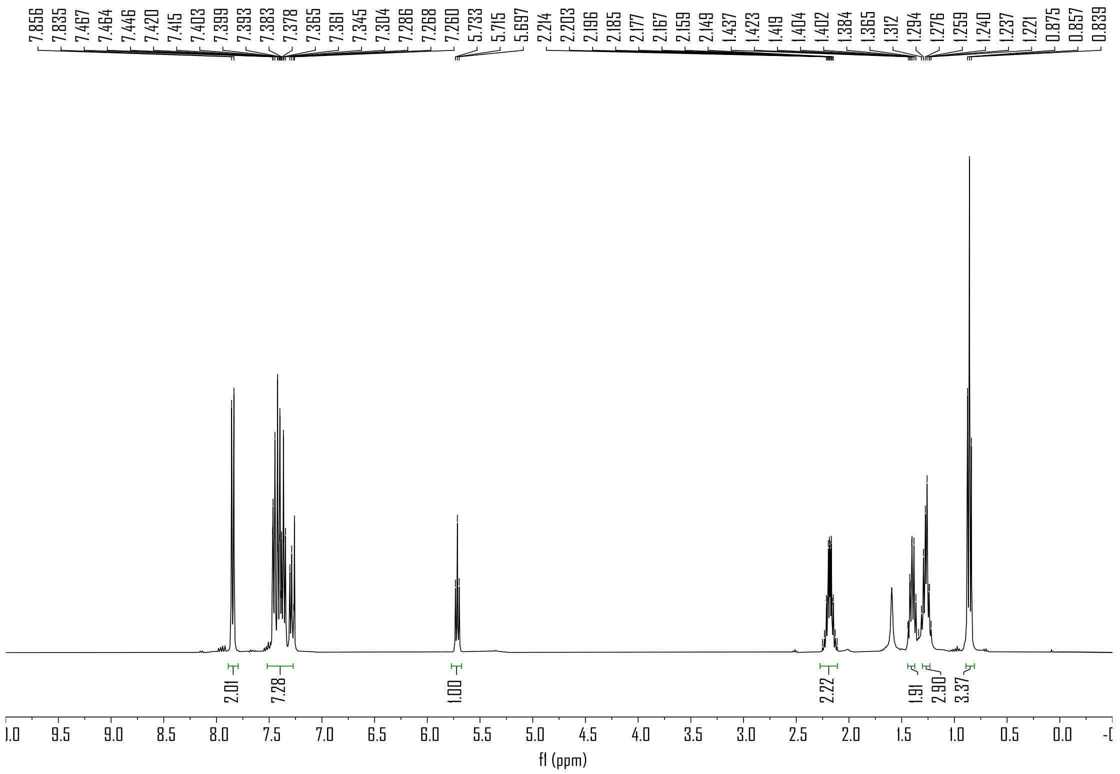
7.856 7.835 7.467 7.464 7.446 7.420 7.415 7.403 7.399 7.393 7.383 7.378 7.365 7.361 7.345 7.304 7.286 7.268 7.260 5.733 5.715 5.697 2.214 2.203 2.196 2.185 2.177 2.167 2.159 2.149 1.437 1.423 1.419 1.404 1.402 1.384 1.365 1.312 1.294 1.276 1.259 1.240 1.237 1.221 0.875 0.857 0.839
2.01
7.28
1.00
2.22
1.91
2.90
3.37
f1 (ppm)

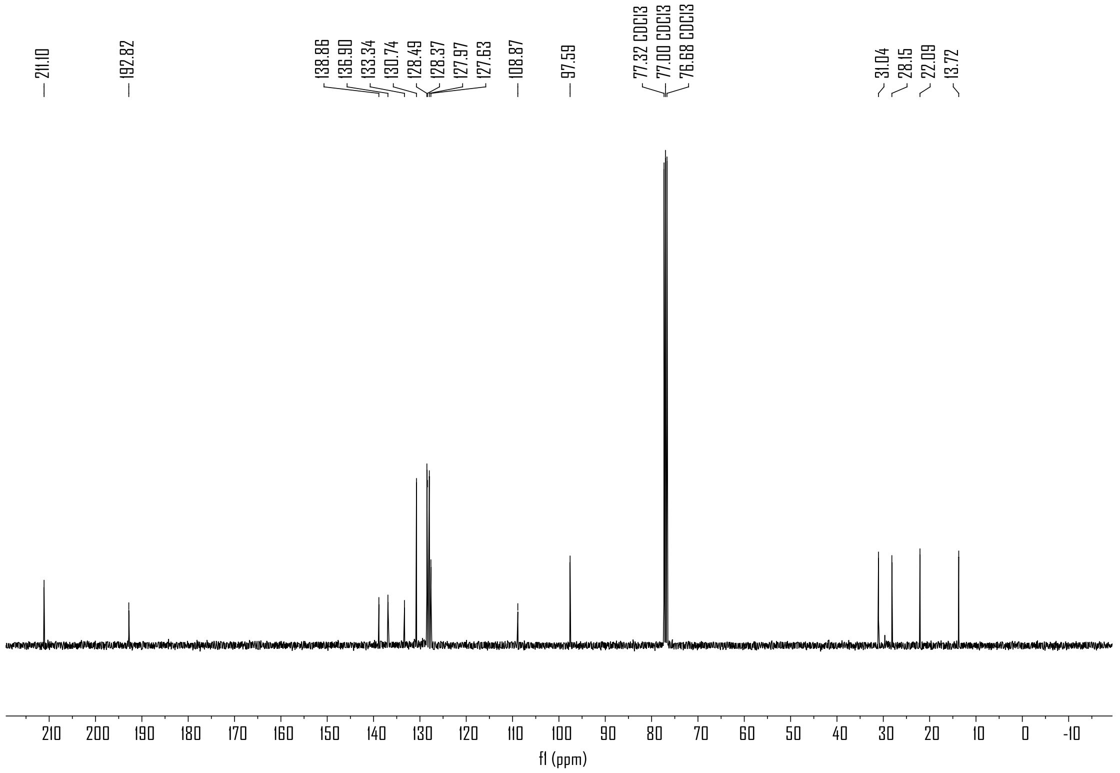
211.10
192.82
138.86 136.90 133.34 130.74 128.49 128.37 127.97 127.63
108.87
97.59
77.32 CDCl3
77.00 CDCl3
76.68 CDCl3
31.04 28.15 22.09 13.72
f1 (ppm)

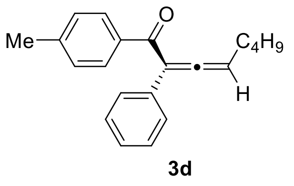
Me
O
C4H9
H

3d

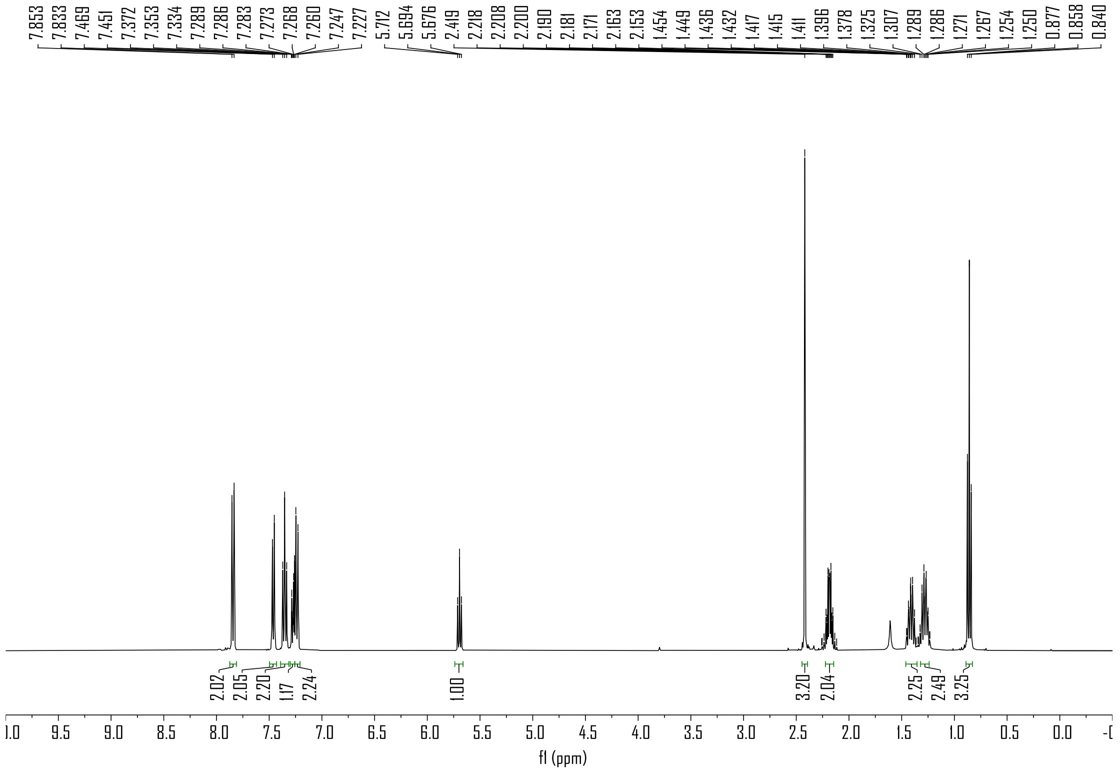
7.853 7.833 7.469 7.451 7.372 7.353 7.334 7.289 7.286 7.283 7.273 7.268 7.260 7.247 7.227
5.712 5.694 5.676 2.419 2.218 2.208 2.200 2.190 2.181 2.171 2.163 2.153 1.454 1.449 1.436 1.432 1.417 1.415 1.411 1.396 1.378 1.325 1.307 1.289 1.286 1.271 1.267 1.254 1.250 0.877 0.858 0.840
2.02 2.05 2.20 1.17 2.24 1.00 3.20 2.04 2.25 2.49 3.25
f1 (ppm)

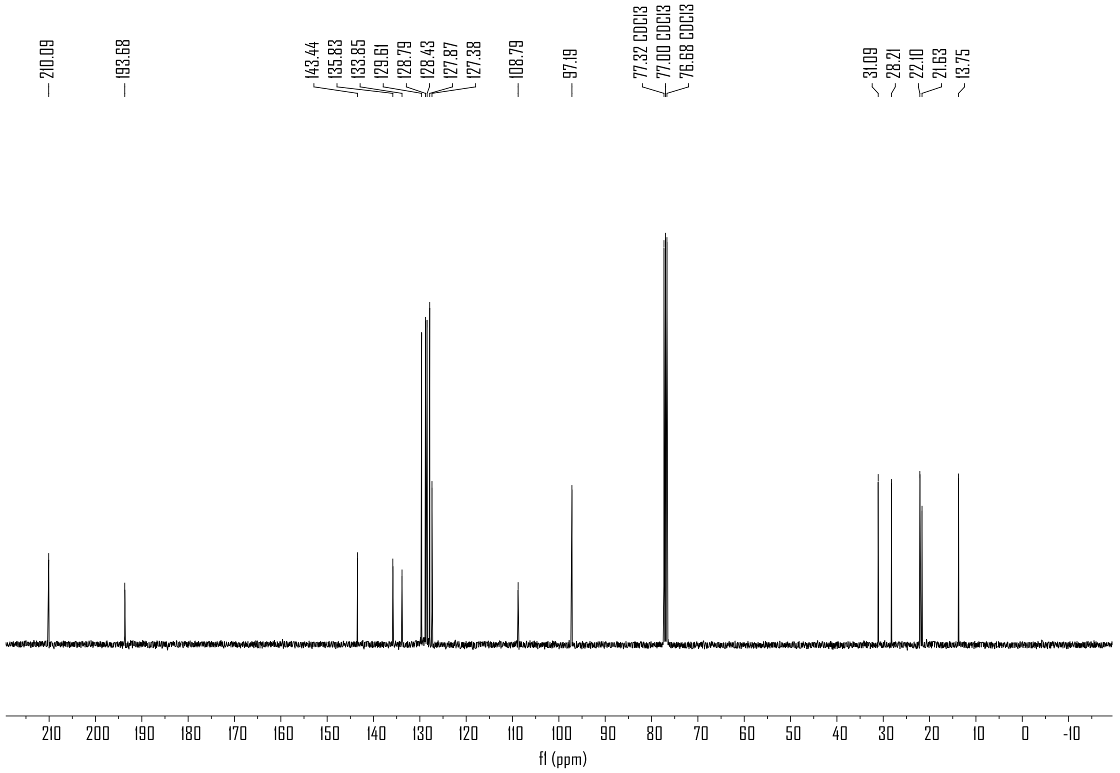
210.09
193.68
143.44 135.83 133.85 129.61 128.79 128.43 127.87 127.38
108.79
97.19
77.32 CDCl3
77.00 CDCl3
76.68 CDCl3
31.09 28.21 22.10 21.63 13.75
f1 (ppm)

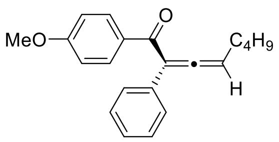
MeO
O
C_4H_9
H

3e

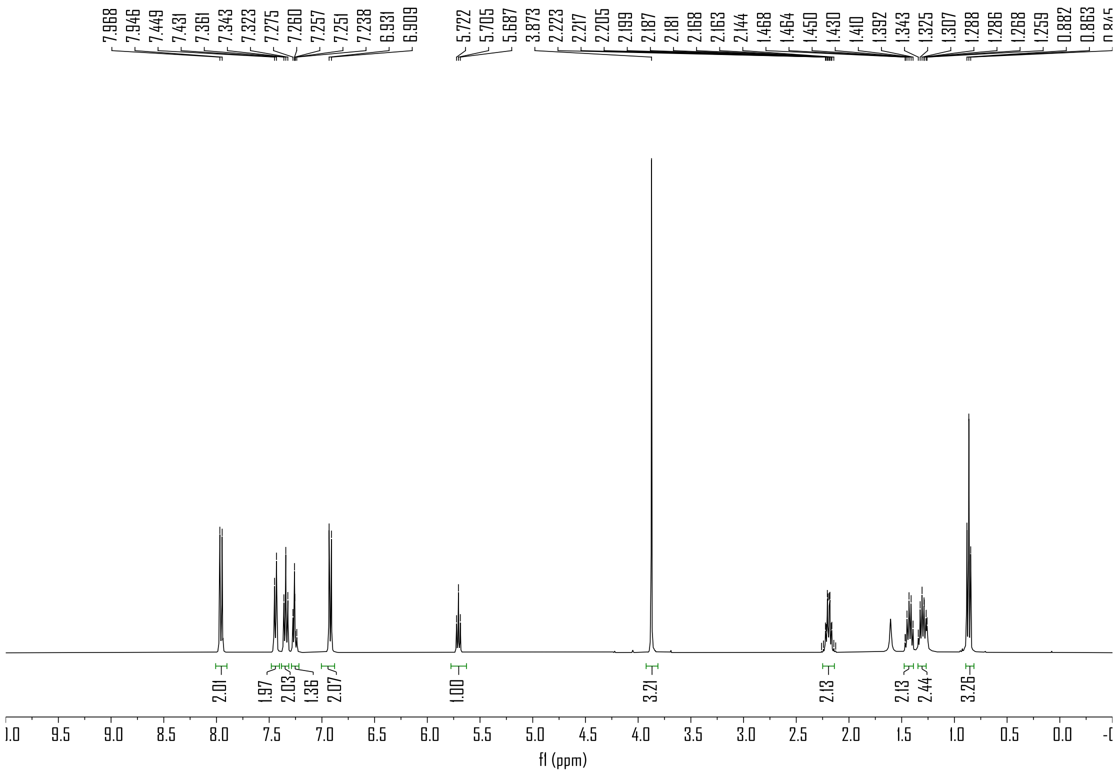
7.968
7.946
7.449
7.431
7.361
7.343
7.323
7.275
7.260
7.257
7.251
7.238
6.931
6.909
5.722
5.705
5.687
3.873
2.223
2.217
2.205
2.199
2.187
2.181
2.168
2.163
2.144
1.468
1.464
1.450
1.430
1.410
1.392
1.343
1.325
1.307
1.288
1.286
1.268
1.259
0.882
0.863
2.01
1.97
2.03
1.36
2.07
1.00
3.21
2.13
2.13
2.44
3.26
9.5
9.0
8.5
8.0
7.5
7.0
6.5
6.0
5.5
5.0
4.5
4.0
3.5
3.0
2.5
2.0
1.5
1.0
0.5
0.0
f1 (ppm)

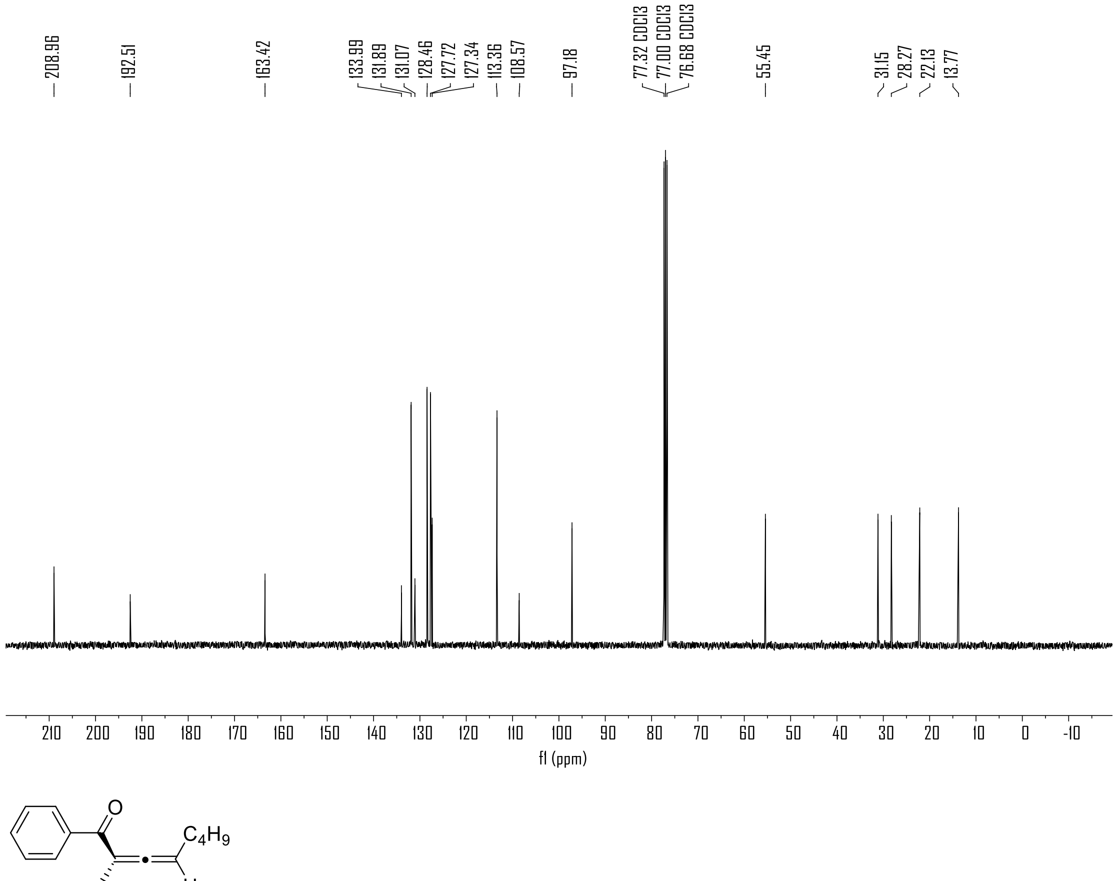
208.96
192.51
163.42
133.99
131.89
131.07
128.46
127.72
127.34
113.36
108.57
97.18
77.32 CDCl3
77.00 CDCl3
76.68 CDCl3
55.45
31.15
28.27
22.13
13.77
210
200
190
180
170
160
150
140
130
120
110
100
90
80
70
60
50
40
30
20
10
0
-10
f1 (ppm)

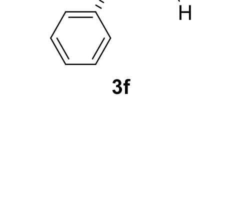
O
C4H9
H

3f

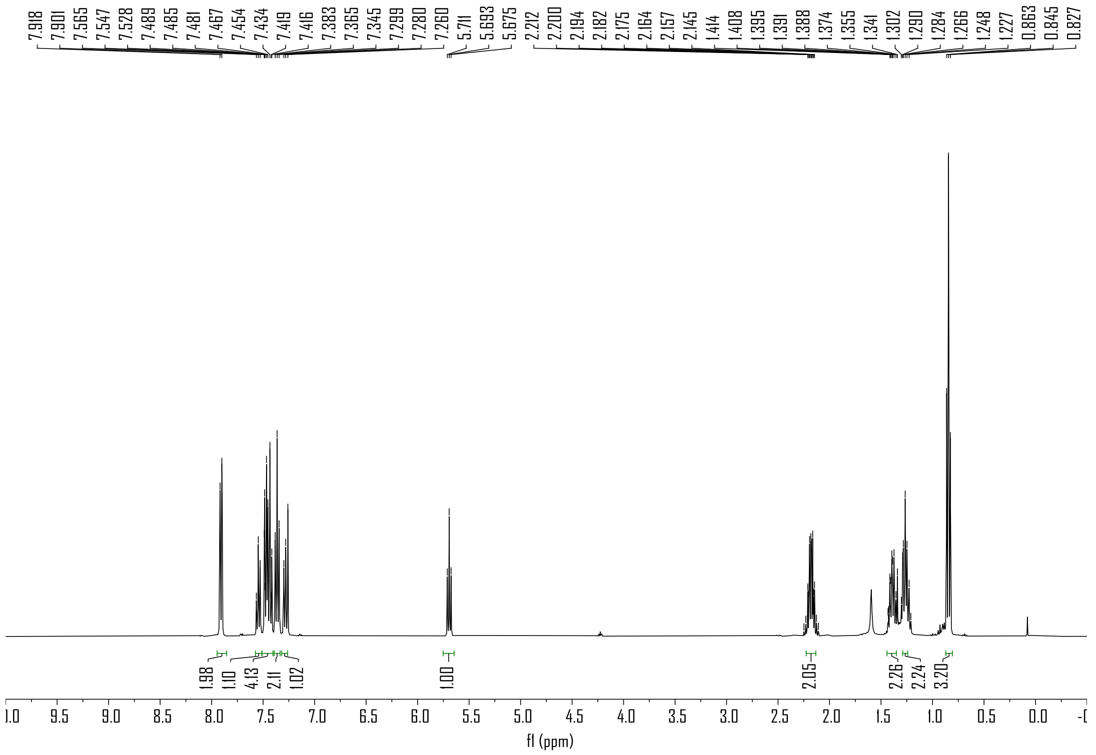
7.918
7.901
7.565
7.547
7.528
7.489
7.485
7.481
7.467
7.454
7.434
7.419
7.416
7.383
7.365
7.345
7.299
7.280
7.260
5.711
5.693
5.675
2.212
2.200
2.194
2.182
2.175
2.164
2.157
2.145
1.414
1.408
1.395
1.391
1.388
1.374
1.355
1.341
1.302
1.290
1.284
1.266
1.248
1.227
0.863
0.845
0.827
1.98
1.10
4.13
2.11
1.02
1.00
2.05
2.26
2.24
3.20
fl (ppm)

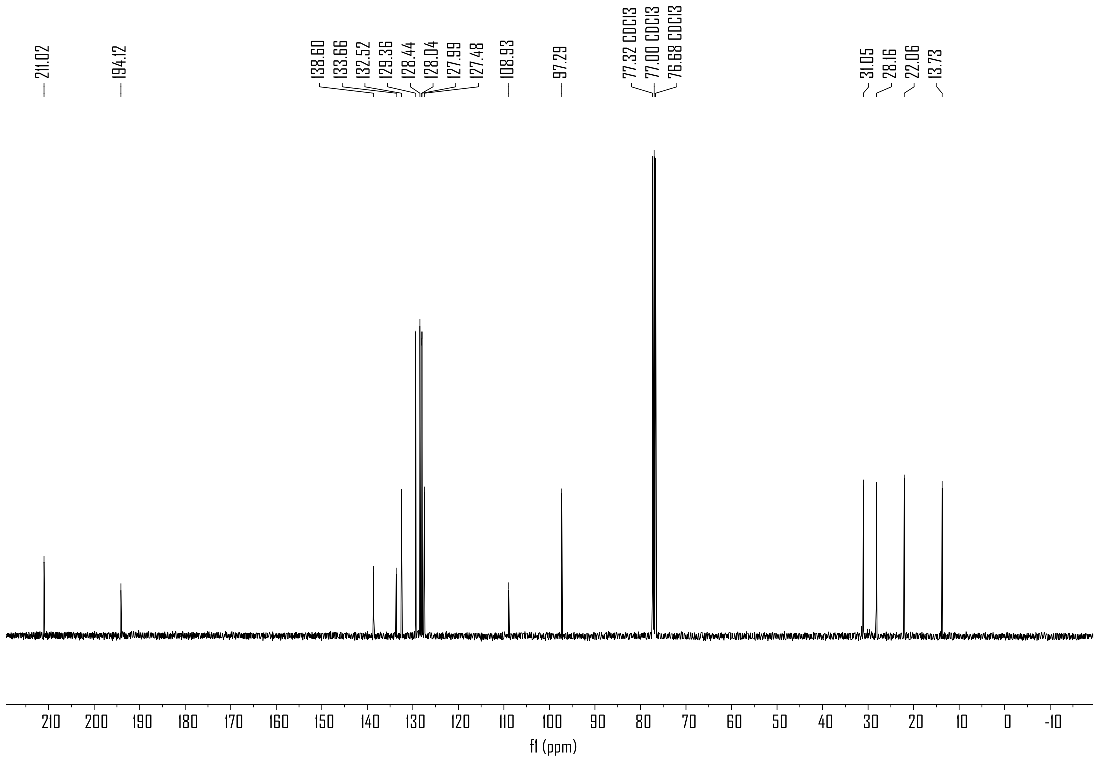
211.02
194.12
138.60
133.66
132.52
129.36
128.44
128.04
127.99
127.48
108.93
97.29
77.32 CDCl3
77.00 CDCl3
76.68 CDCl3
31.05
28.16
22.06
13.73
fl (ppm)

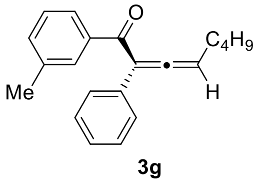
O
C_4H_9
Me
H

3g

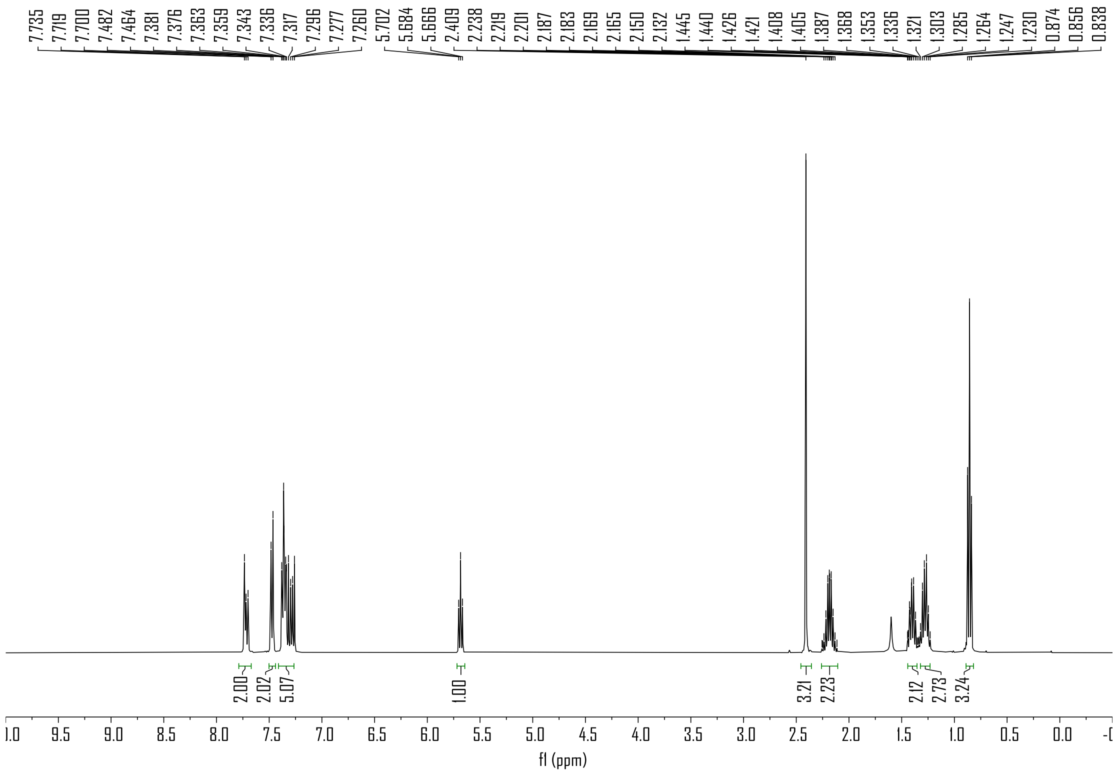
7.735 7.719 7.700 7.482 7.464 7.381 7.376 7.363 7.359 7.343 7.336 7.317 7.296 7.277 7.260 5.702 5.684 5.666 2.409 2.238 2.219 2.201 2.187 2.183 2.169 2.165 2.150 2.132 1.445 1.440 1.426 1.421 1.408 1.405 1.387 1.368 1.353 1.336 1.321 1.303 1.285 1.264 1.247 1.230 0.874 0.856 0.838
2.00 2.02 5.07 1.00 3.21 2.23 2.12 2.73 3.24
fl (ppm)

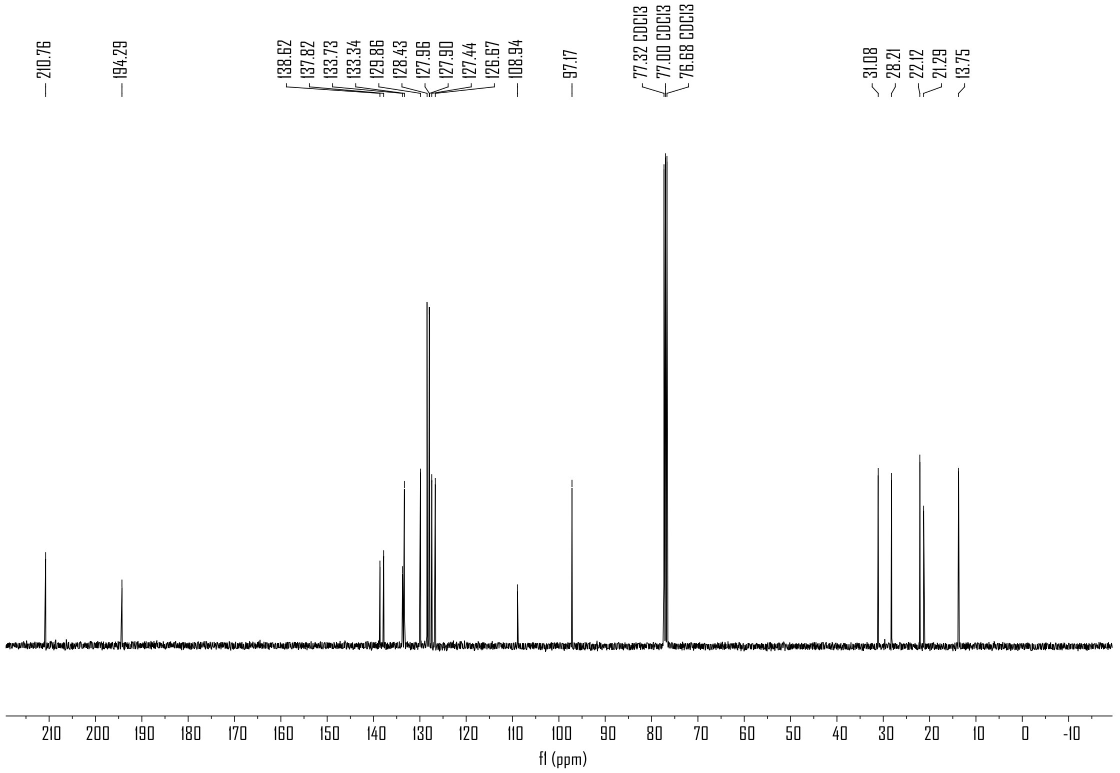
210.76 194.29 138.62 137.82 133.73 133.34 129.86 128.43 127.96 127.90 127.44 126.67 108.94 97.17 77.32 CDCl3 77.00 CDCl3 76.68 CDCl3 31.08 28.21 22.12 21.29 13.75
fl (ppm)

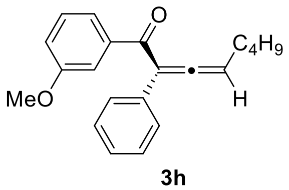
O
C4H9
MeO
H

3h

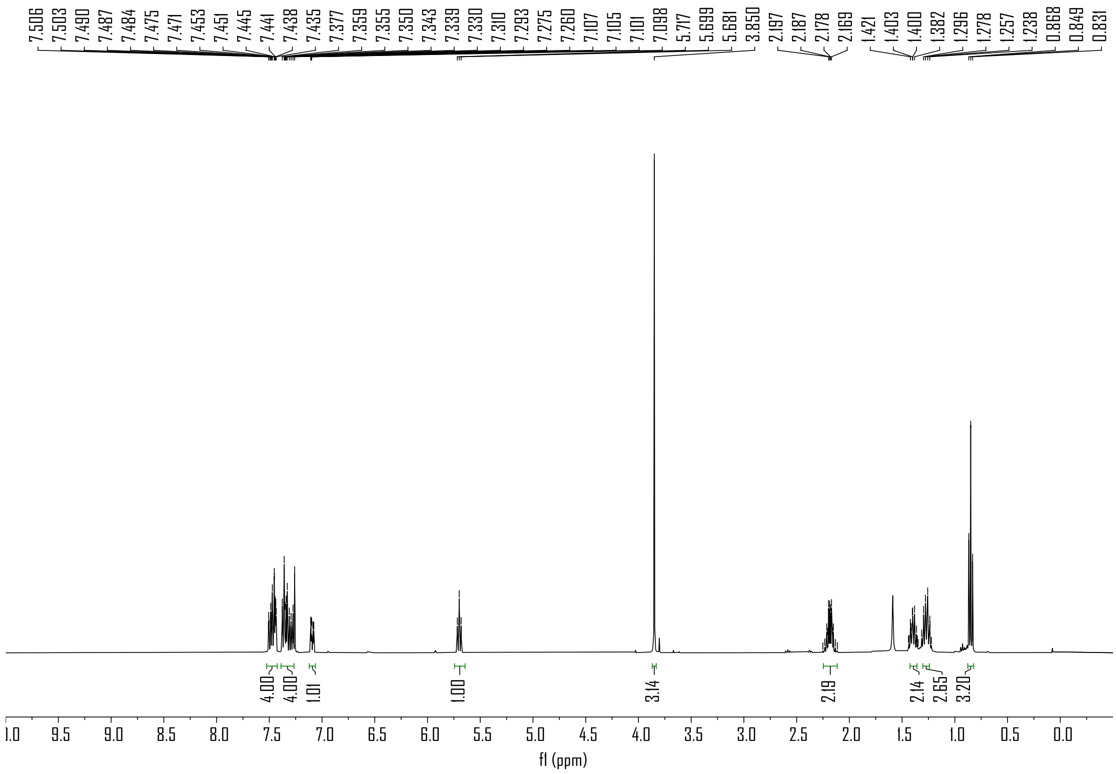
7.506 7.503 7.490 7.487 7.484 7.475 7.471 7.453 7.451 7.445 7.441 7.438 7.435 7.377 7.359 7.355 7.350 7.343 7.339 7.330 7.310 7.293 7.275 7.260 7.107 7.105 7.101 7.098 5.717 5.699 5.681 3.850 2.197 2.187 2.178 2.169 1.421 1.403 1.400 1.382 1.296 1.278 1.257 1.238 0.868 0.849 0.831
4.00
4.00
1.01
1.00
3.14
2.19
2.14
2.65
3.20
1.0 9.5 9.0 8.5 8.0 7.5 7.0 6.5 6.0 5.5 5.0 4.5 4.0 3.5 3.0 2.5 2.0 1.5 1.0 0.5 0.0
f1 (ppm)

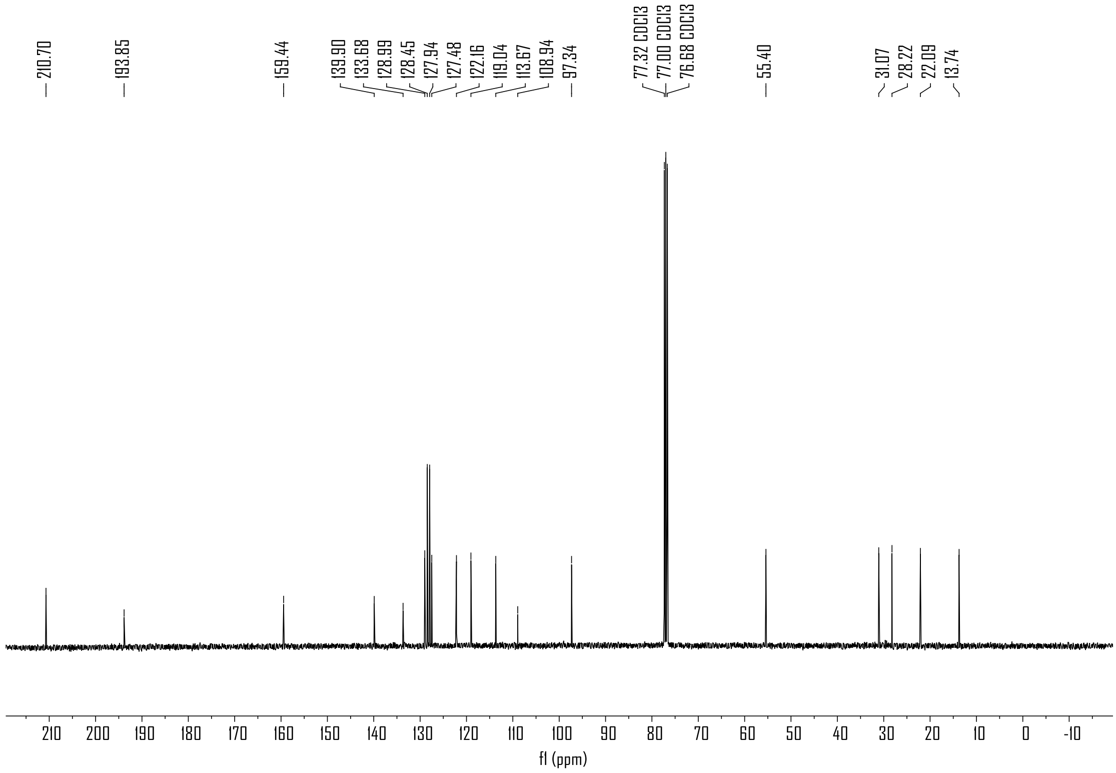
210.70
193.85
159.44
139.90 133.68 128.99 128.45 127.94 127.48 122.16 119.04 113.67 108.94
97.34
77.32 CDCl3 77.00 CDCl3 76.68 CDCl3
55.40
31.07 28.22 22.09 13.74
210 200 190 180 170 160 150 140 130 120 110 100 90 80 70 60 50 40 30 20 10 0 -10
f1 (ppm)

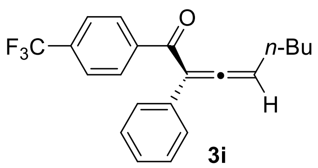
F3C
O
n-Bu
H

3i

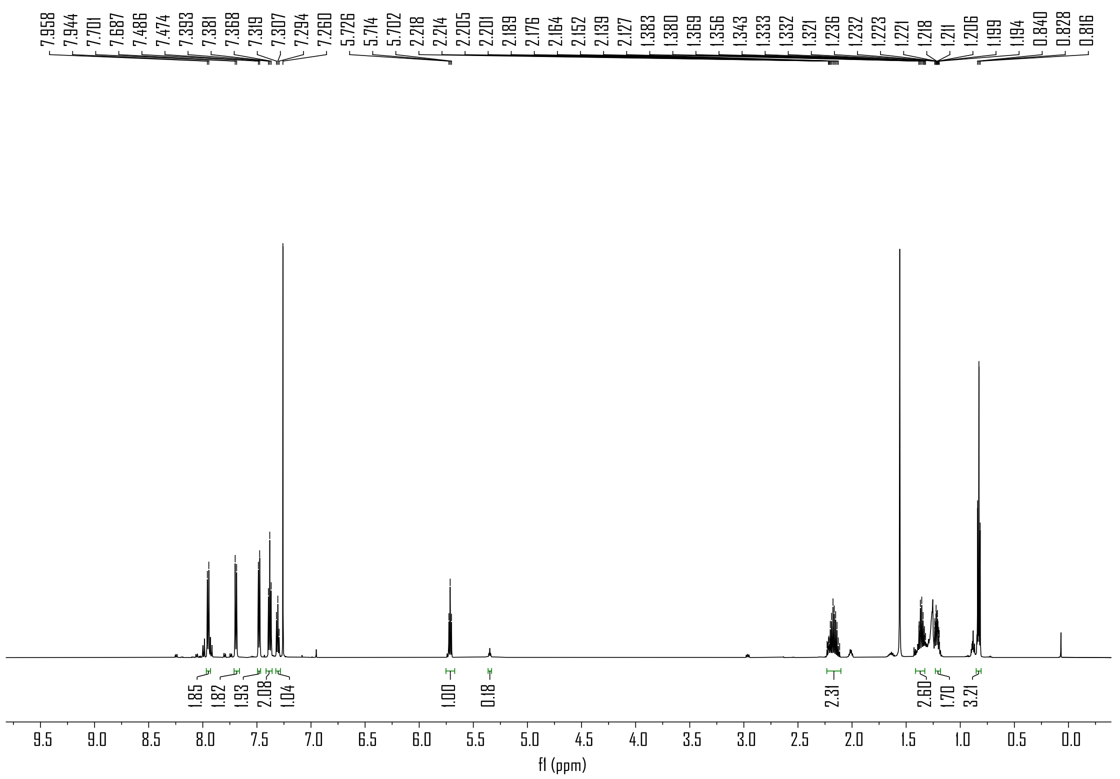

7.958
7.944
7.701
7.687
7.486
7.474
7.393
7.381
7.368
7.319
7.307
7.294
7.260
5.726
5.714
5.702
2.218
2.214
2.205
2.201
2.189
2.176
2.164
2.152
2.139
2.127
1.383
1.380
1.369
1.356
1.343
1.333
1.332
1.321
1.236
1.232
1.223
1.221
1.218
1.211
1.206
1.199
1.194
0.840
0.828
0.816
1.85
1.82
1.93
2.08
1.04
1.00
0.18
2.31
2.60
1.70
3.21
9.5 9.0 8.5 8.0 7.5 7.0 6.5 6.0 5.5 5.0 4.5 4.0 3.5 3.0 2.5 2.0 1.5 1.0 0.5 0.0
f1 (ppm)

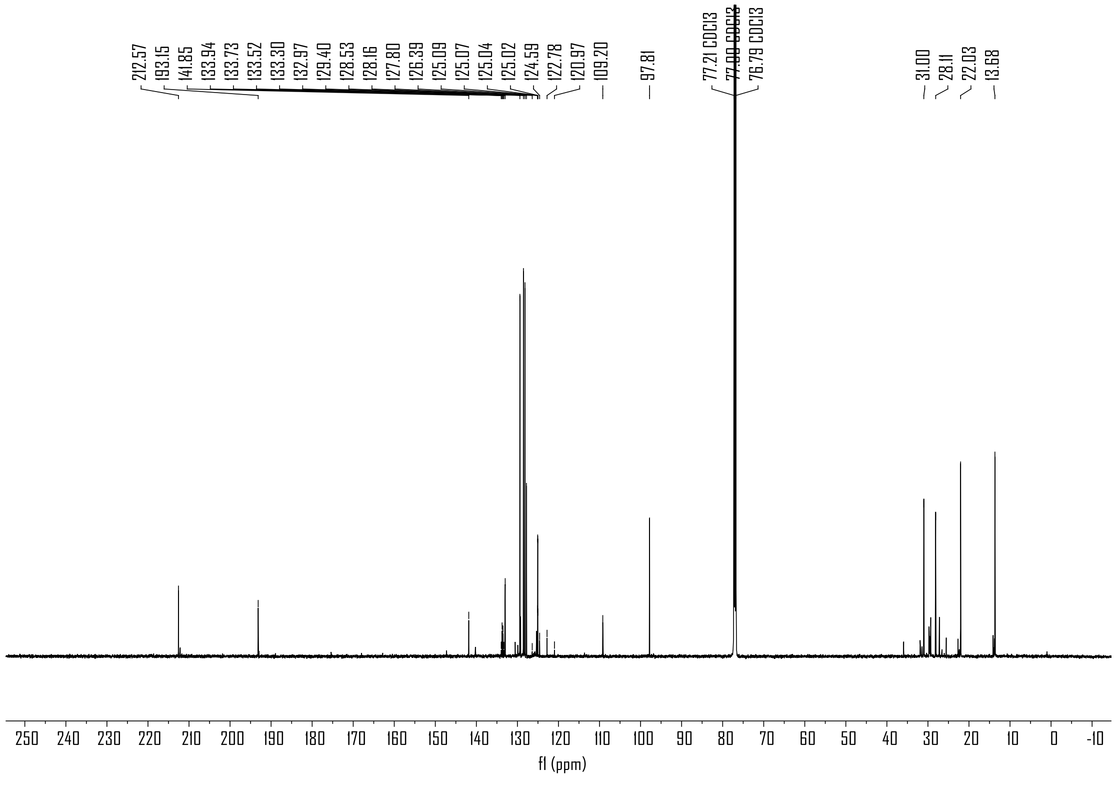

212.57
193.15
141.85
133.94
133.73
133.52
133.30
132.97
129.40
128.53
128.16
127.80
126.39
125.09
125.07
125.04
125.02
124.59
122.78
120.97
109.20
97.81
77.21 CDCl3
77.00 CDCl3
76.79 CDCl3
31.00
28.11
22.03
13.68
250 240 230 220 210 200 190 180 170 160 150 140 130 120 110 100 90 80 70 60 50 40 30 20 10 0 -10
f1 (ppm)

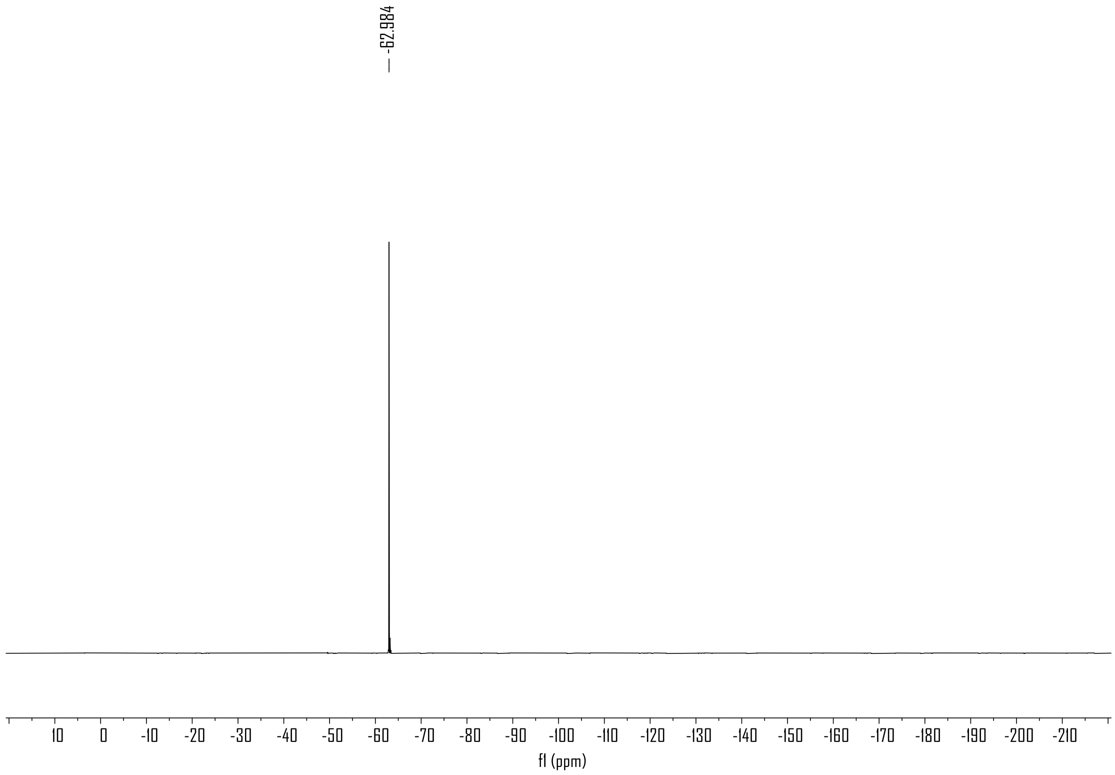

--62.984
10
0
-10
-20
-30
-40
-50
-60
-70
-80
-90
-100
-110
-120
-130
-140
-150
-160
-170
-180
-190
-200
-210
f1 (ppm)

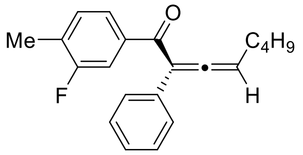

Me
F
O
C4H9
H

3j

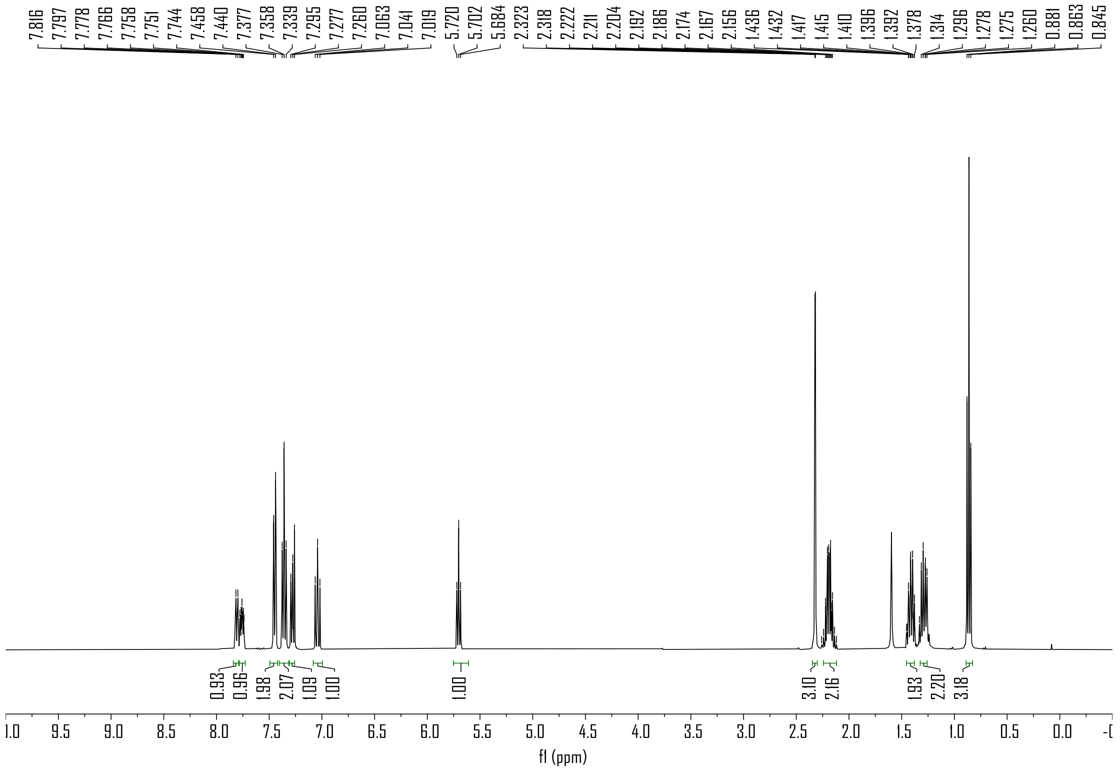
7.816 7.797 7.778 7.766 7.758 7.751 7.744 7.458 7.440 7.377 7.358 7.339 7.295 7.277 7.260 7.063 7.041 7.019 5.720 5.702 5.684 2.323 2.318 2.222 2.211 2.204 2.192 2.186 2.174 2.167 2.156 1.436 1.432 1.417 1.415 1.410 1.396 1.392 1.378 1.314 1.296 1.278 1.275 1.260 0.881 0.863 0.845
0.93 0.96 1.98 2.07 1.09 1.00 1.00 3.10 2.16 1.93 2.20 3.18
f1 (ppm)

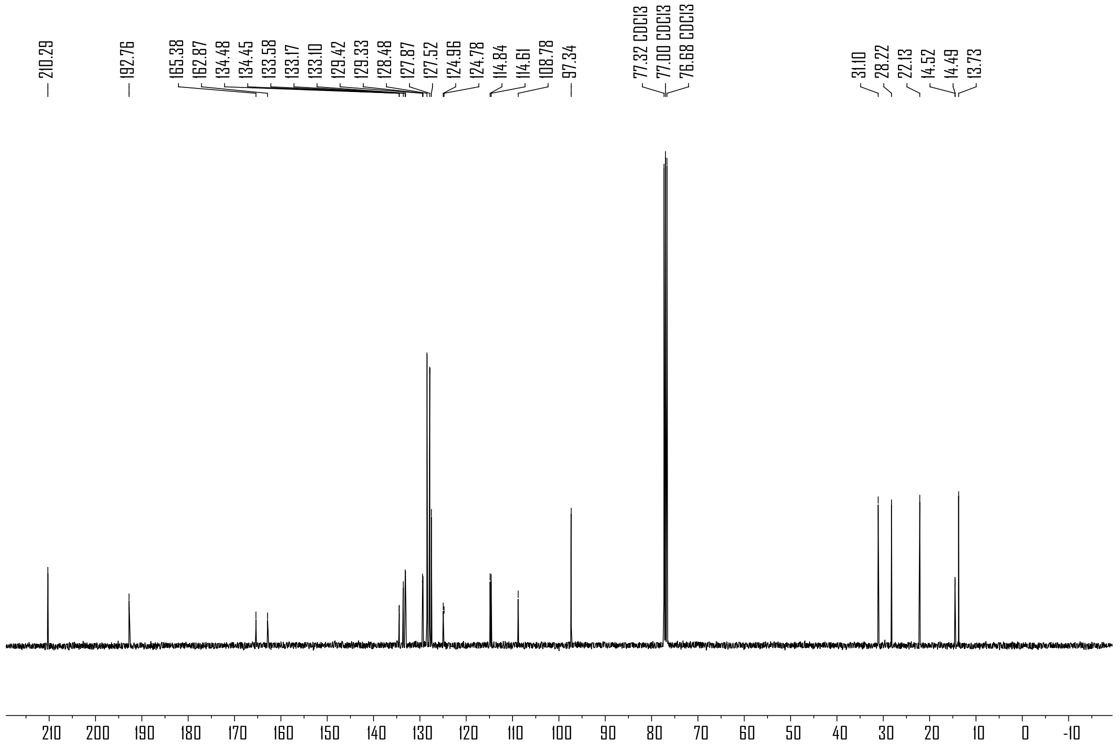
210.29 192.76 165.38 162.87 134.48 134.45 133.58 133.17 133.10 129.42 129.33 128.48 127.87 127.52 124.96 124.78 114.84 114.61 108.78 97.34
77.32 CDCl3 77.00 CDCl3 76.68 CDCl3
31.10 28.22 22.13 14.52 14.49 13.73
f1 (ppm)

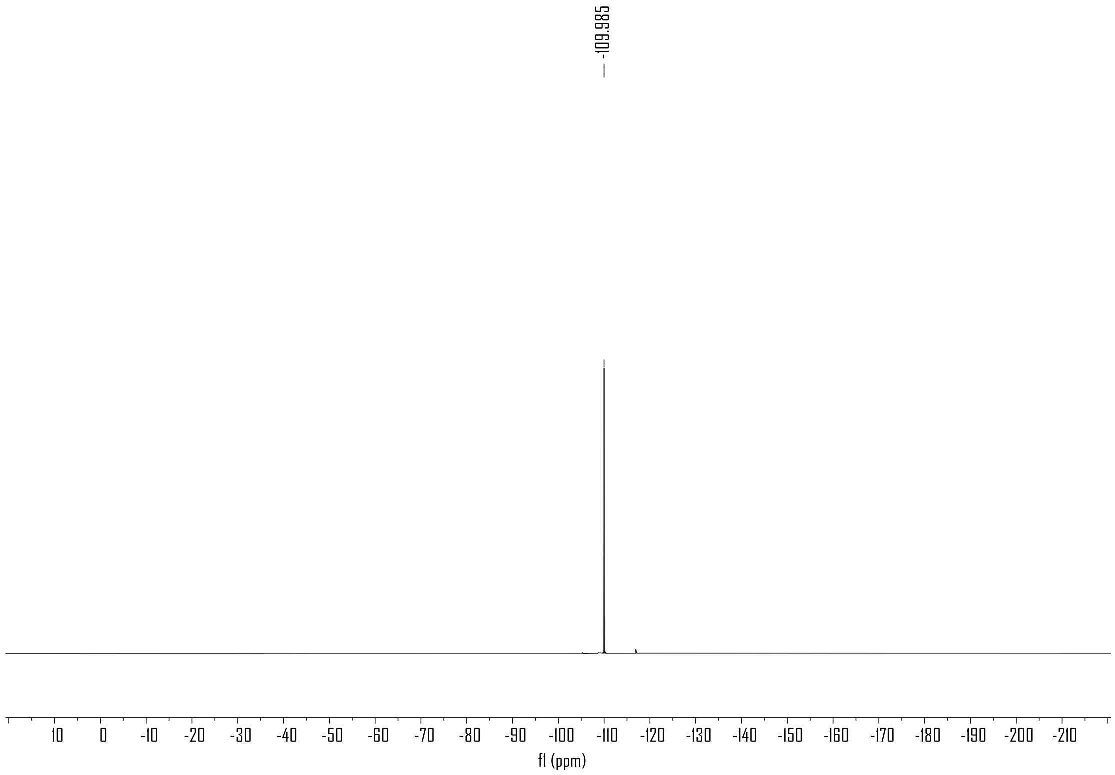
-109.985
10 0 -10 -20 -30 -40 -50 -60 -70 -80 -90 -100 -110 -120 -130 -140 -150 -160 -170 -180 -190 -200 -210
f1 (ppm)

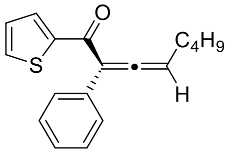
O
S
C4H9
H

3k

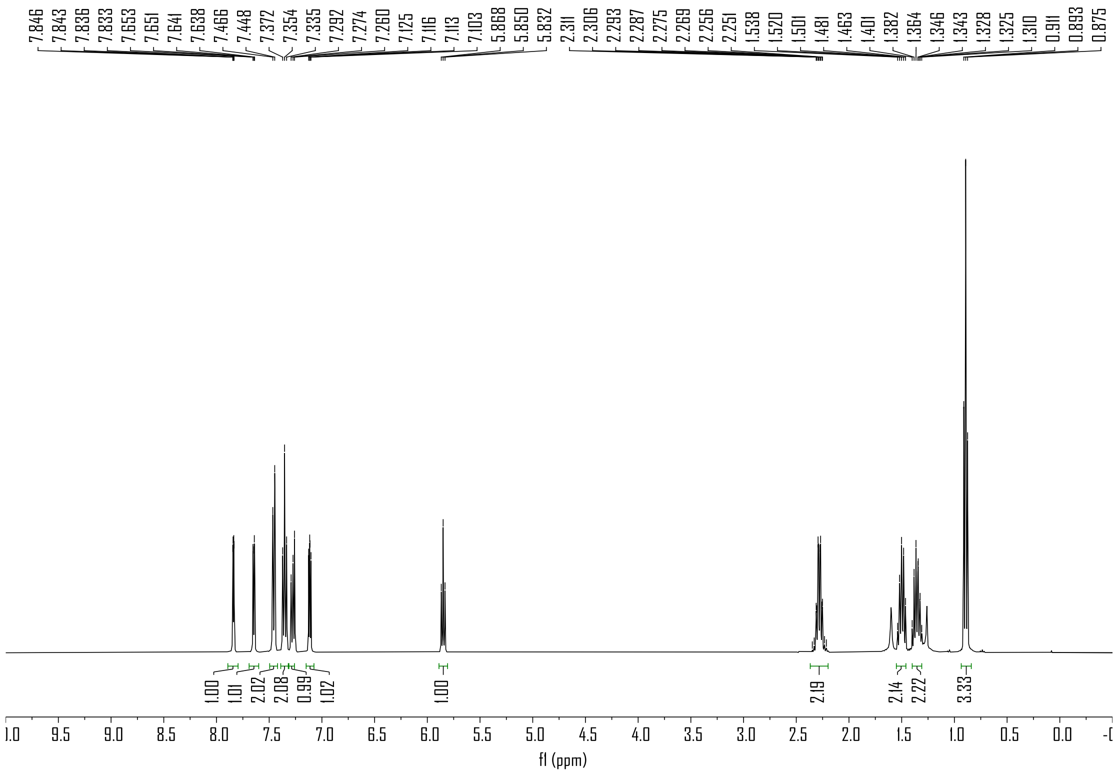
7.846 7.843 7.836 7.833 7.653 7.651 7.641 7.638 7.466 7.448 7.372 7.354 7.335 7.292 7.274 7.260 7.125 7.116 7.113 7.103 5.868 5.850 5.832 2.311 2.306 2.293 2.287 2.275 2.269 2.256 2.251 1.538 1.520 1.501 1.481 1.463 1.401 1.382 1.364 1.346 1.343 1.328 1.325 1.310 0.911 0.893 0.875
1.00 1.01 2.02 2.08 0.99 1.02 1.00 2.19 2.14 2.22 3.33
9.5 9.0 8.5 8.0 7.5 7.0 6.5 6.0 5.5 5.0 4.5 4.0 3.5 3.0 2.5 2.0 1.5 1.0 0.5 0.0
f1 (ppm)

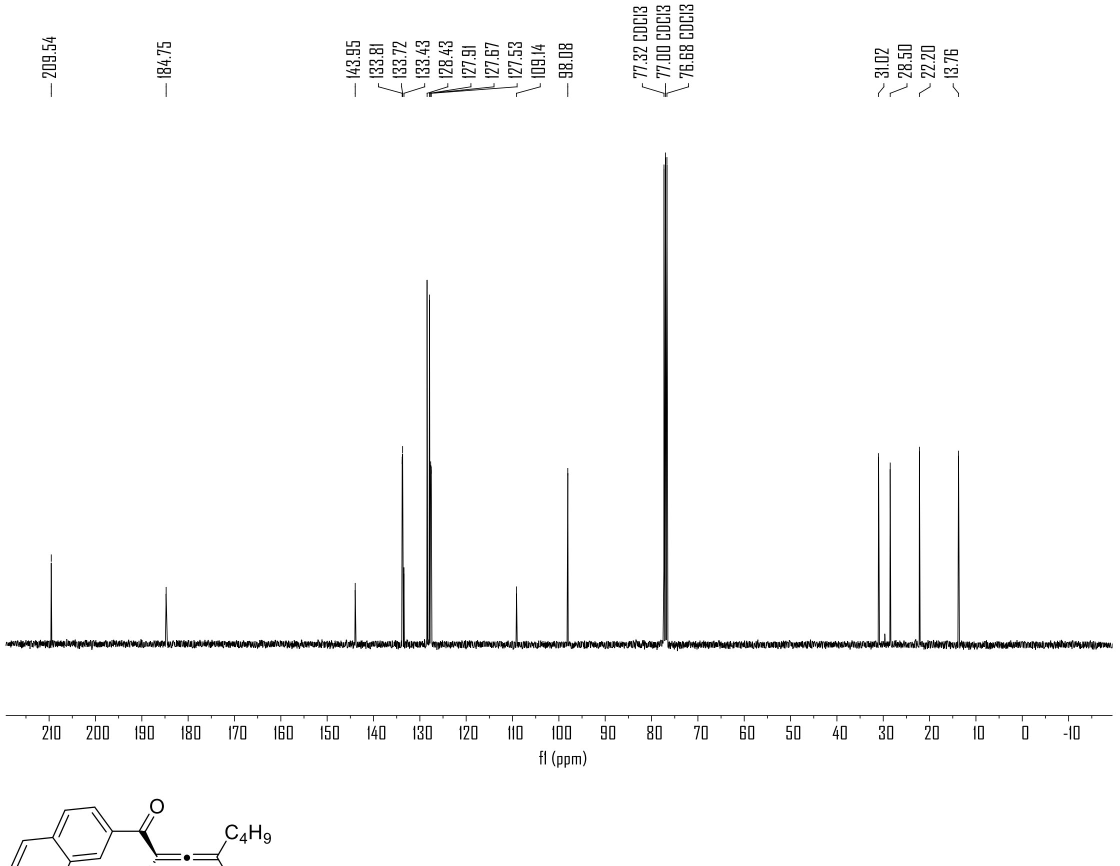
209.54 184.75 143.95 133.81 133.72 133.43 128.43 127.91 127.67 127.53 109.14 98.08 77.32 CDCl3 77.00 CDCl3 76.68 CDCl3 31.02 28.50 22.20 13.76
210 200 190 180 170 160 150 140 130 120 110 100 90 80 70 60 50 40 30 20 10 0 -10
f1 (ppm)

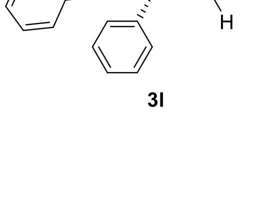
O
C_4H_9
H

3l

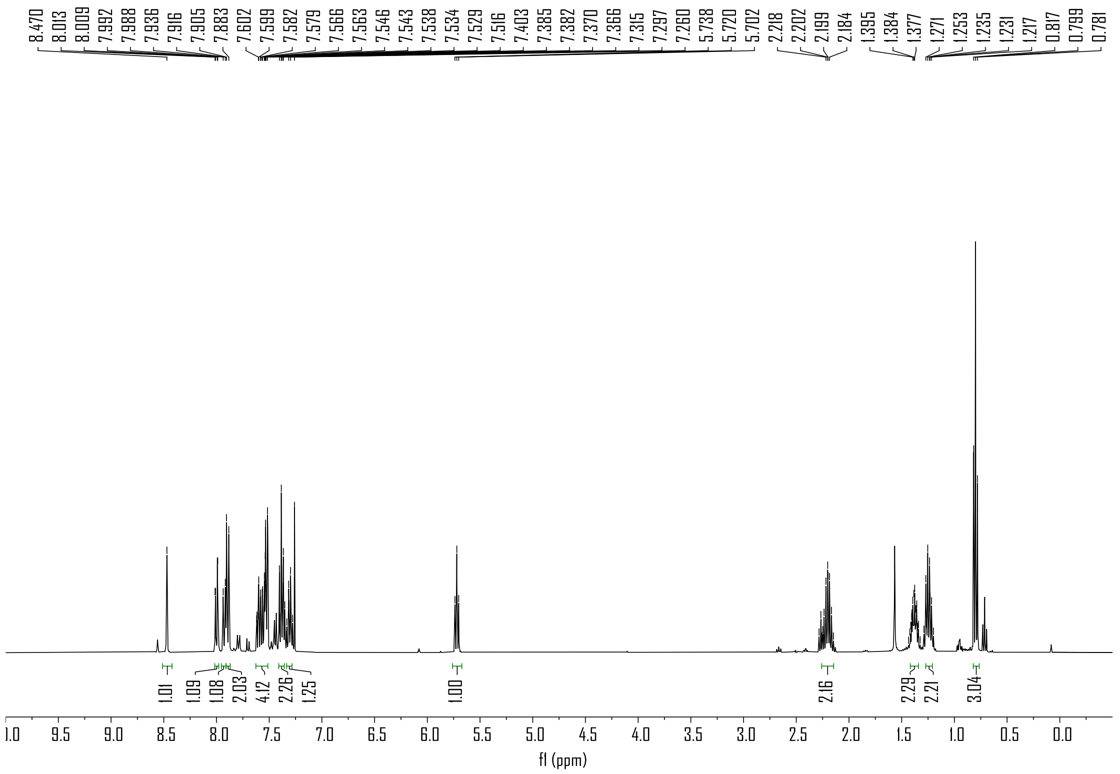
8.470 8.013 8.009 7.992 7.988 7.936 7.916 7.905 7.883 7.602 7.599 7.582 7.579 7.566 7.563 7.546 7.543 7.538 7.534 7.529 7.516 7.403 7.385 7.382 7.370 7.366 7.315 7.297 7.260 5.738 5.720 5.702 2.218 2.202 2.199 2.184 1.395 1.384 1.377 1.271 1.253 1.235 1.231 1.217 0.817 0.799 0.781
1.01 1.09 1.08 2.03 4.12 2.26 1.25 1.00 2.16 2.29 2.21 3.04
fl (ppm)

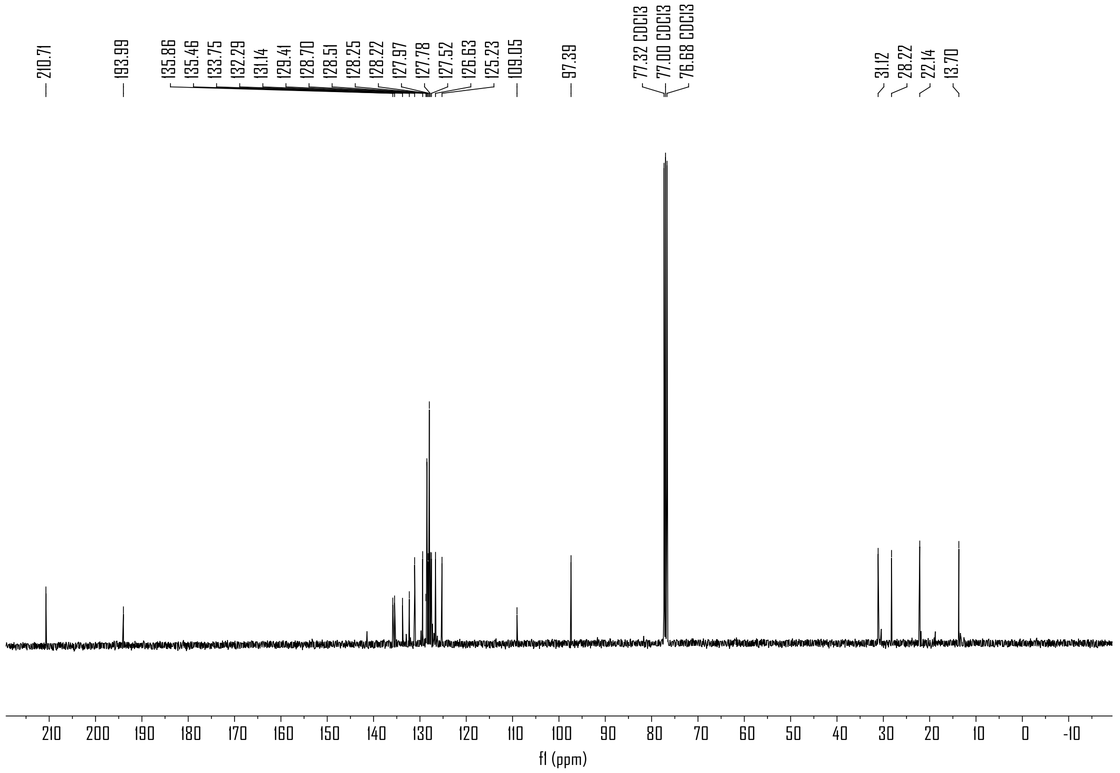
210.71 193.99 135.86 135.46 133.75 132.29 131.14 129.41 128.70 128.51 128.25 128.22 127.97 127.78 127.52 126.63 125.23 109.05 97.39 77.32 CDCl3 77.00 CDCl3 76.68 CDCl3 31.12 28.22 22.14 13.70
fl (ppm)

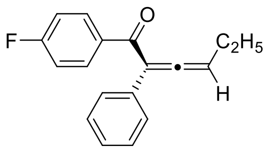
F
O
C2H5
H

3m

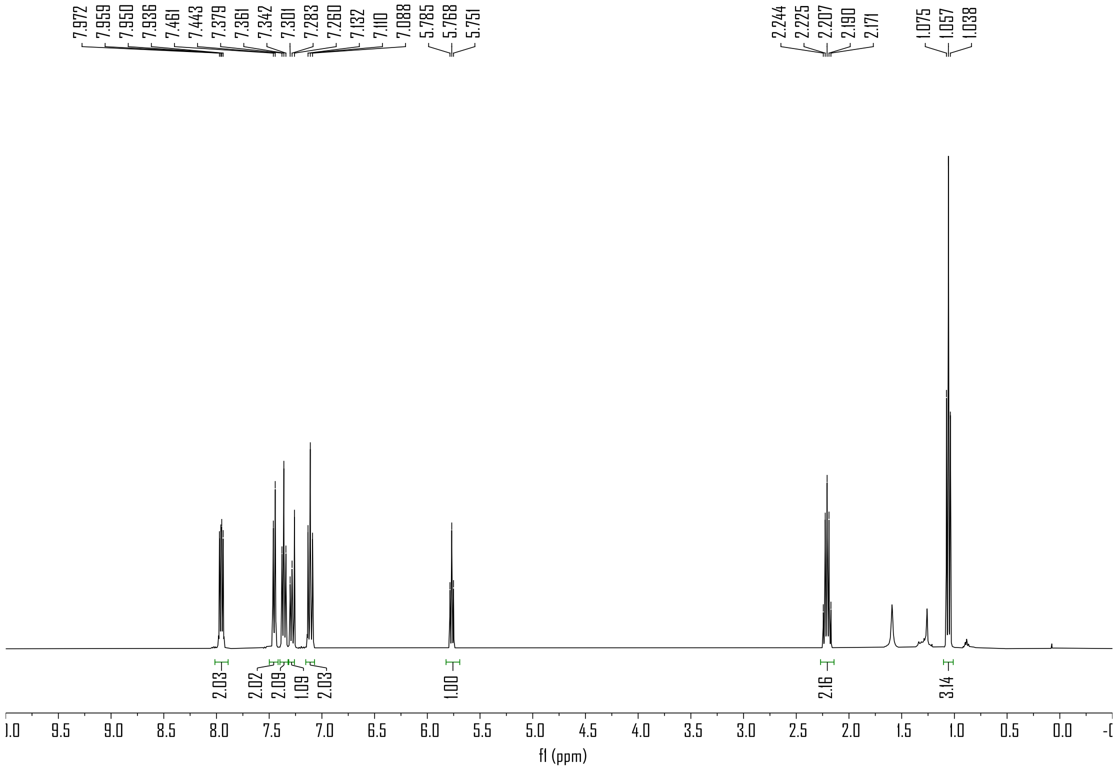
7.972
7.959
7.950
7.936
7.461
7.443
7.379
7.361
7.342
7.301
7.283
7.260
7.132
7.110
7.088
5.785
5.768
5.751
2.244
2.225
2.207
2.190
2.171
1.075
1.057
1.038
2.03
2.02
2.09
1.09
2.03
1.00
2.16
3.14
f1 (ppm)

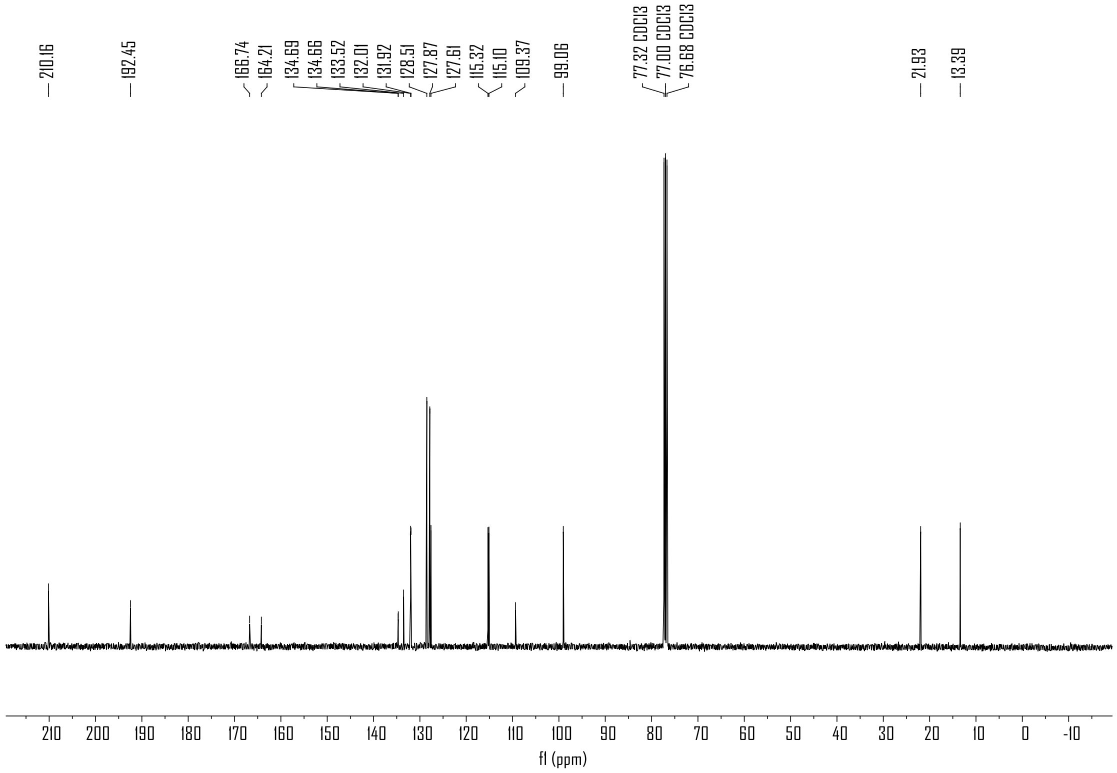
210.16
192.45
166.74
164.21
134.69
134.66
133.52
132.01
131.92
128.51
127.87
127.61
115.32
115.10
109.37
99.06
77.32 CDCl3
77.00 CDCl3
76.68 CDCl3
21.93
13.39
f1 (ppm)

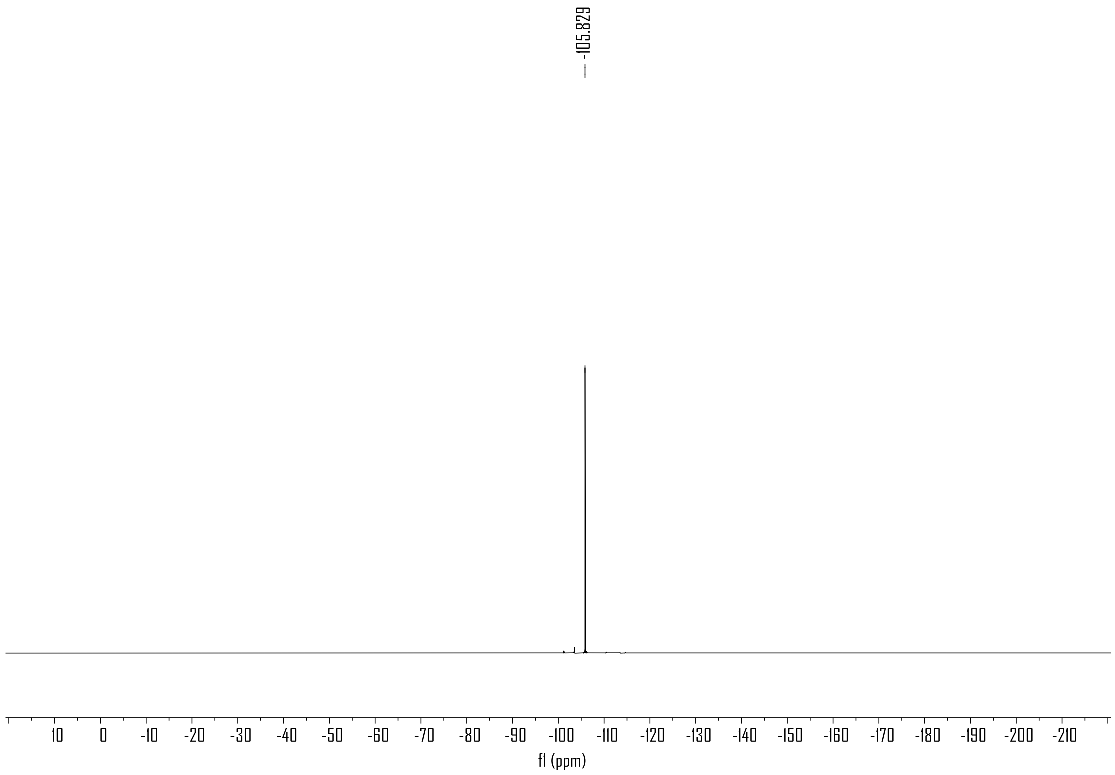
-105.829
10
0
-10
-20
-30
-40
-50
-60
-70
-80
-90
-100
-110
-120
-130
-140
-150
-160
-170
-180
-190
-200
-210
f1 (ppm)

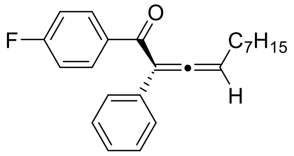
F
O
C7H15
H

3n

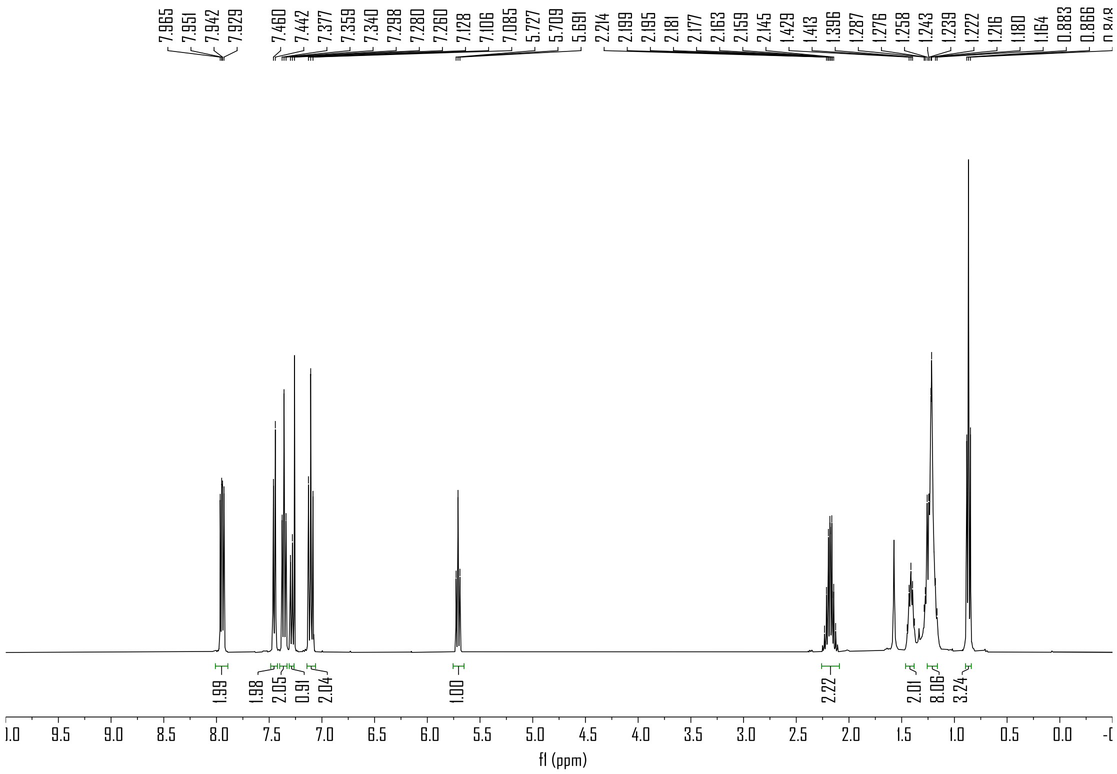
7.965
7.951
7.942
7.929
7.460
7.442
7.377
7.359
7.340
7.298
7.280
7.260
7.128
7.106
7.085
5.727
5.709
5.691
2.214
2.199
2.195
2.181
2.177
2.163
2.159
2.145
1.429
1.413
1.396
1.287
1.276
1.258
1.243
1.239
1.222
1.216
1.180
1.164
0.883
0.866
1.99
1.98
2.05
0.91
2.04
1.00
2.22
2.01
8.06
3.24
fl (ppm)

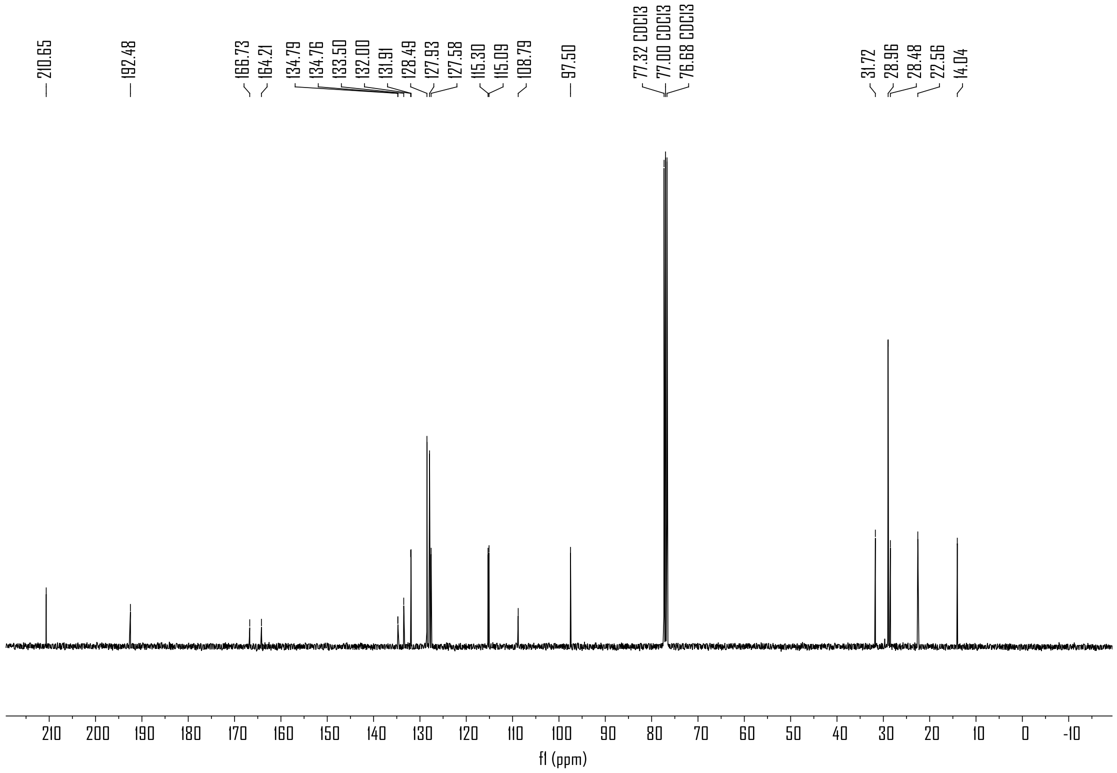
210.65
192.48
166.73
164.21
134.79
134.76
133.50
132.00
131.91
128.49
127.93
127.58
115.30
115.09
108.79
97.50
77.32 CDCl3
77.00 CDCl3
76.68 CDCl3
31.72
28.96
28.48
22.56
14.04
fl (ppm)

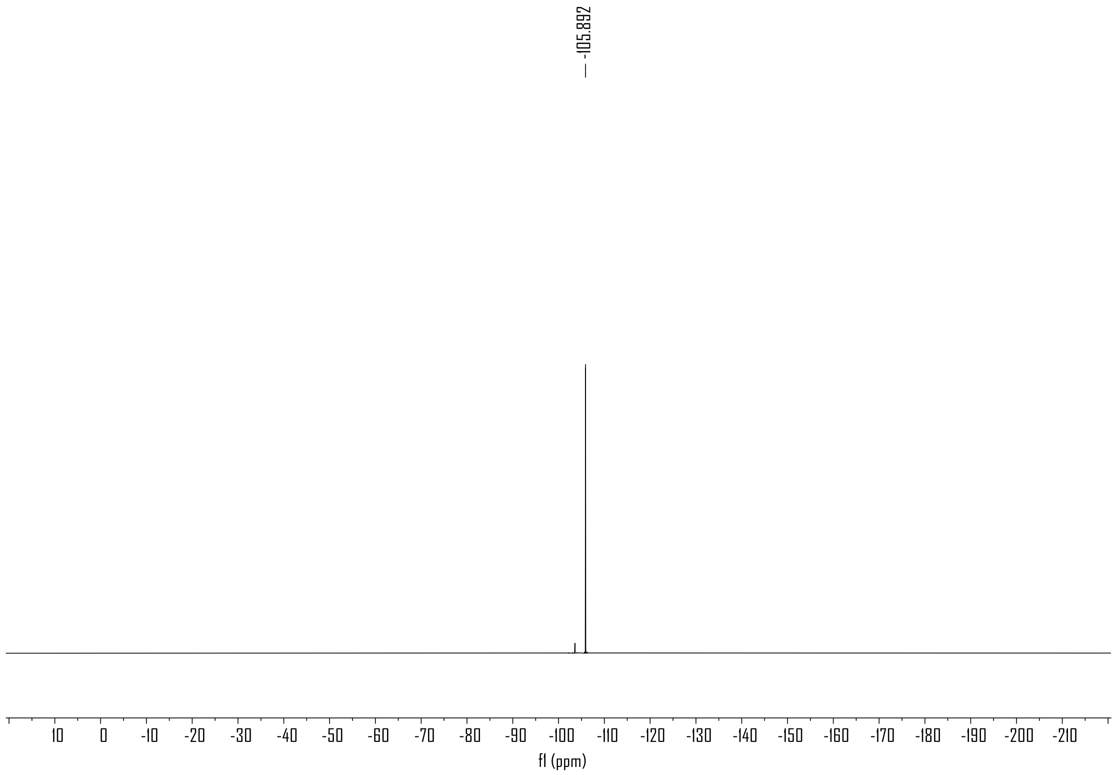
-105.892
10
0
-10
-20
-30
-40
-50
-60
-70
-80
-90
-100
-110
-120
-130
-140
-150
-160
-170
-180
-190
-200
-210
f1 (ppm)

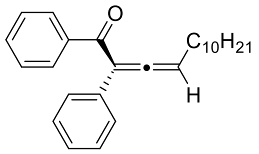
O
$C_{10}H_{21}$
H

3o

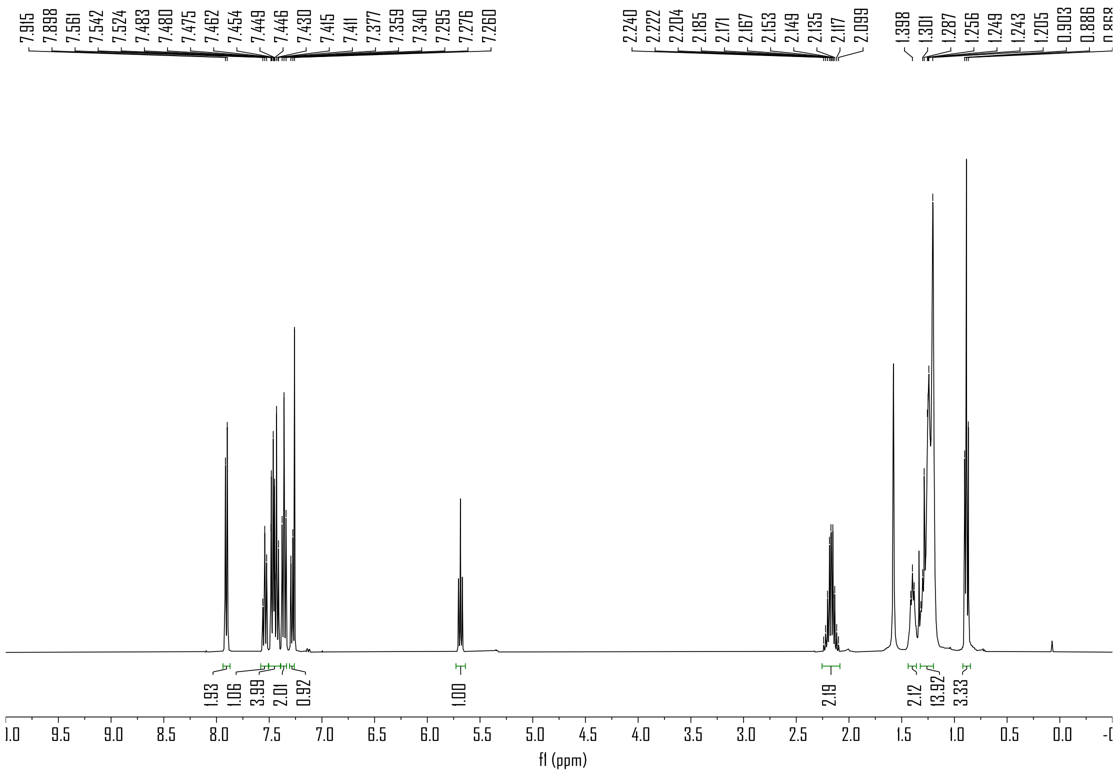
7.915
7.898
7.561
7.542
7.524
7.483
7.480
7.475
7.462
7.454
7.449
7.446
7.430
7.415
7.411
7.377
7.359
7.340
7.295
7.276
7.260
2.240
2.222
2.204
2.185
2.171
2.167
2.153
2.149
2.135
2.117
2.099
1.398
1.301
1.287
1.256
1.249
1.243
1.205
0.903
0.886
1.93
1.06
3.99
2.01
0.92
1.00
2.19
2.12
13.92
3.33
9.5
9.0
8.5
8.0
7.5
7.0
6.5
6.0
5.5
5.0
4.5
4.0
3.5
3.0
2.5
2.0
1.5
1.0
0.5
0.0
f1 (ppm)

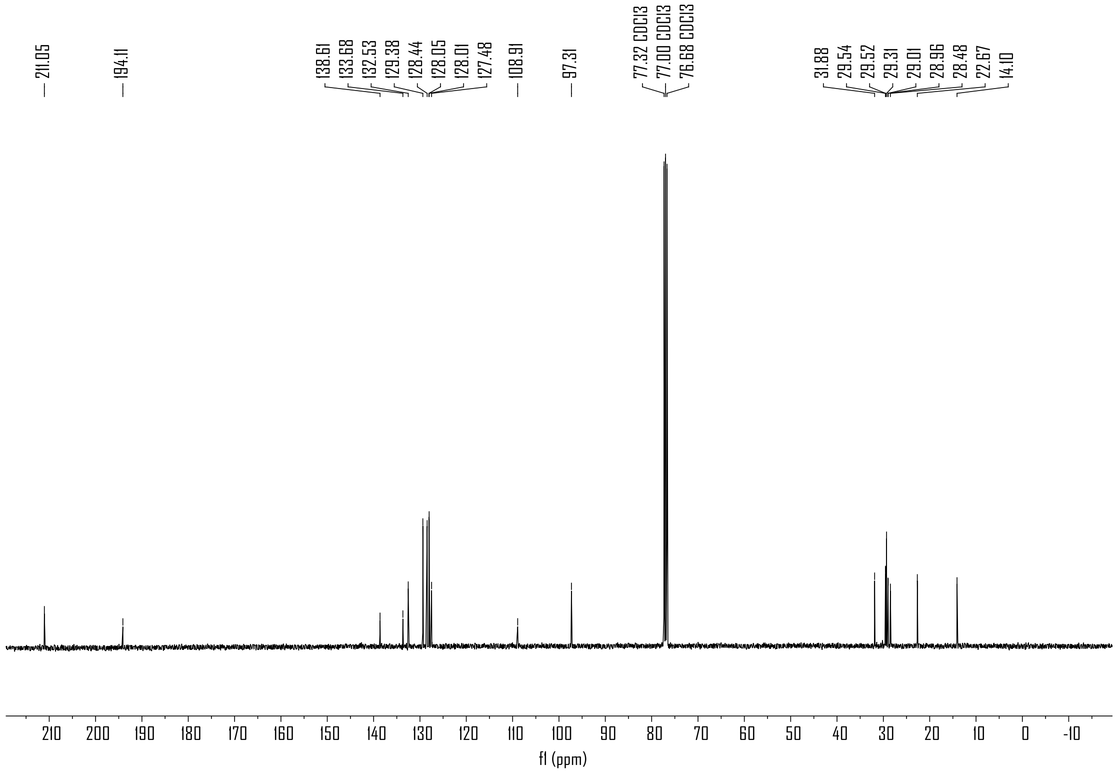
211.05
194.11
138.61
133.68
132.53
129.38
128.44
128.05
128.01
127.48
108.91
97.31
77.32 CDCl3
77.00 CDCl3
76.68 CDCl3
31.88
29.54
29.52
29.31
29.01
28.96
28.48
22.67
14.10
210
200
190
180
170
160
150
140
130
120
110
100
90
80
70
60
50
40
30
20
10
0
-10
f1 (ppm)

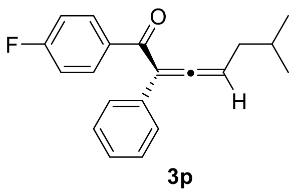
F
O
H

3p

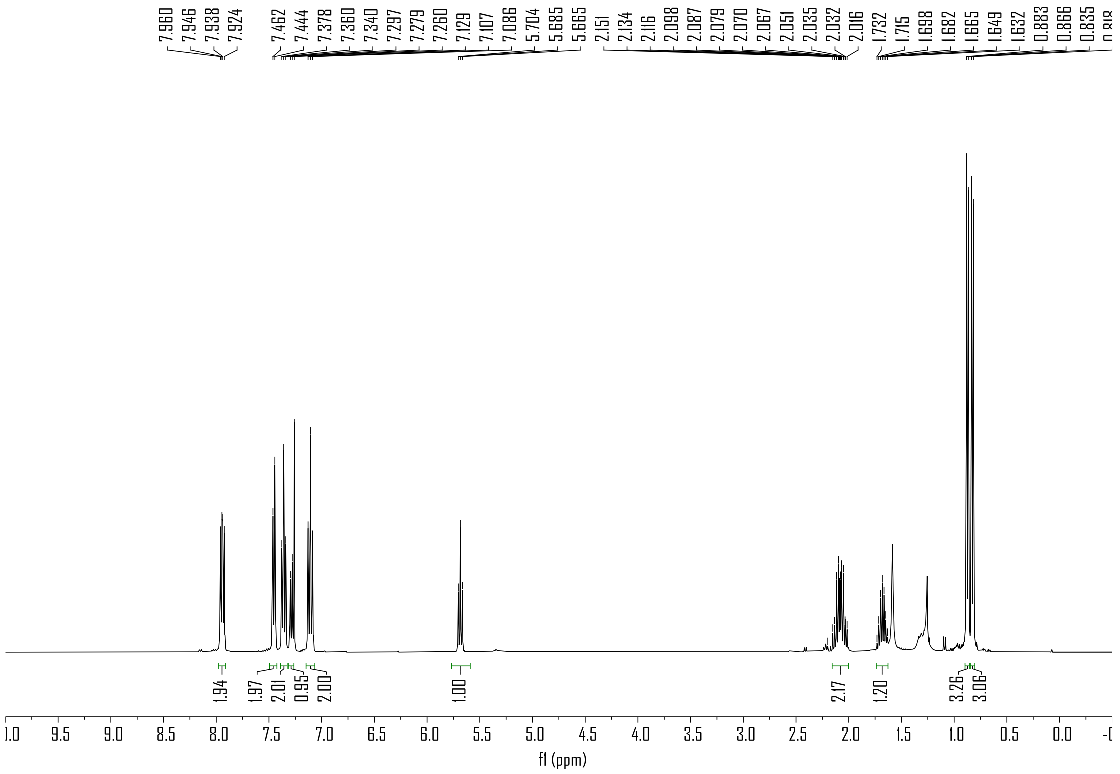
7.960
7.946
7.938
7.924
7.462
7.444
7.378
7.360
7.340
7.297
7.279
7.260
7.129
7.107
7.086
5.704
5.685
5.665
2.151
2.134
2.116
2.098
2.087
2.079
2.070
2.067
2.051
2.035
2.032
2.016
1.732
1.715
1.698
1.682
1.665
1.649
1.632
0.883
0.866
0.835
1.94
1.97
2.01
0.95
2.00
1.00
2.17
1.20
3.25
3.06
9.5 9.0 8.5 8.0 7.5 7.0 6.5 6.0 5.5 5.0 4.5 4.0 3.5 3.0 2.5 2.0 1.5 1.0 0.5 0.0
f1 (ppm)

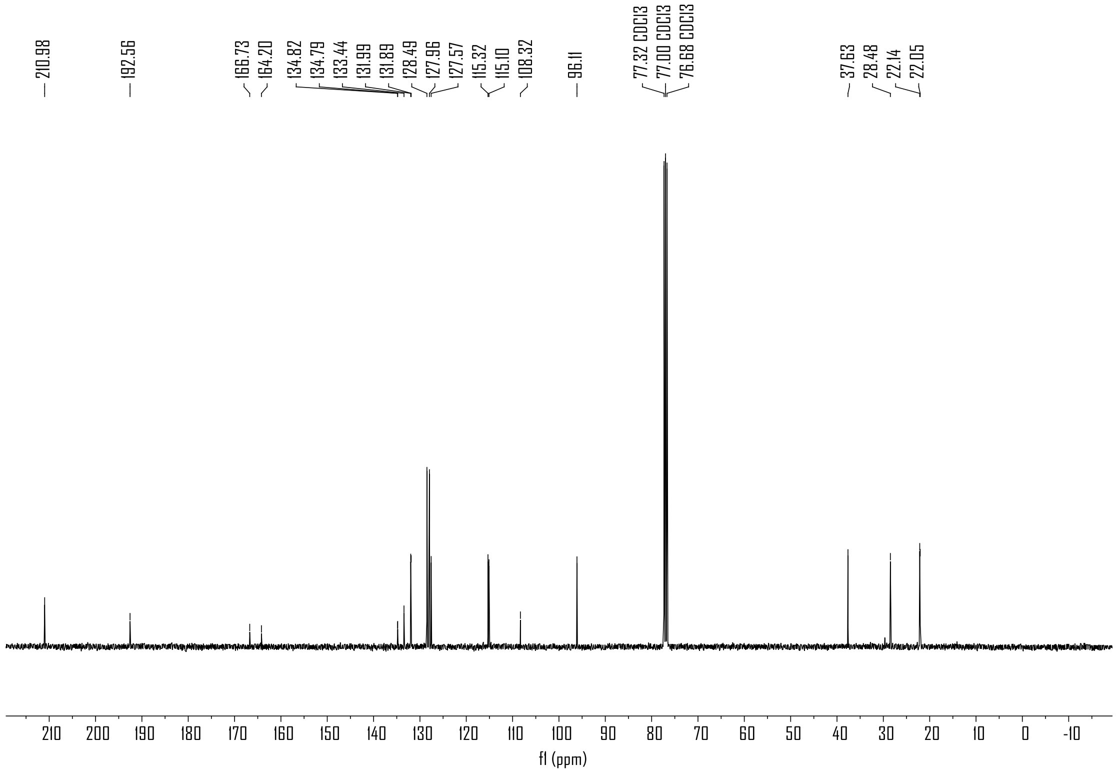
210.98
192.56
166.73
164.20
134.82
134.79
133.44
131.99
131.89
128.49
127.96
127.57
115.32
115.10
108.32
96.11
77.32 CDCl3
77.00 CDCl3
76.68 CDCl3
37.63
28.48
22.14
22.05
210 200 190 180 170 160 150 140 130 120 110 100 90 80 70 60 50 40 30 20 10 0 -10
f1 (ppm)

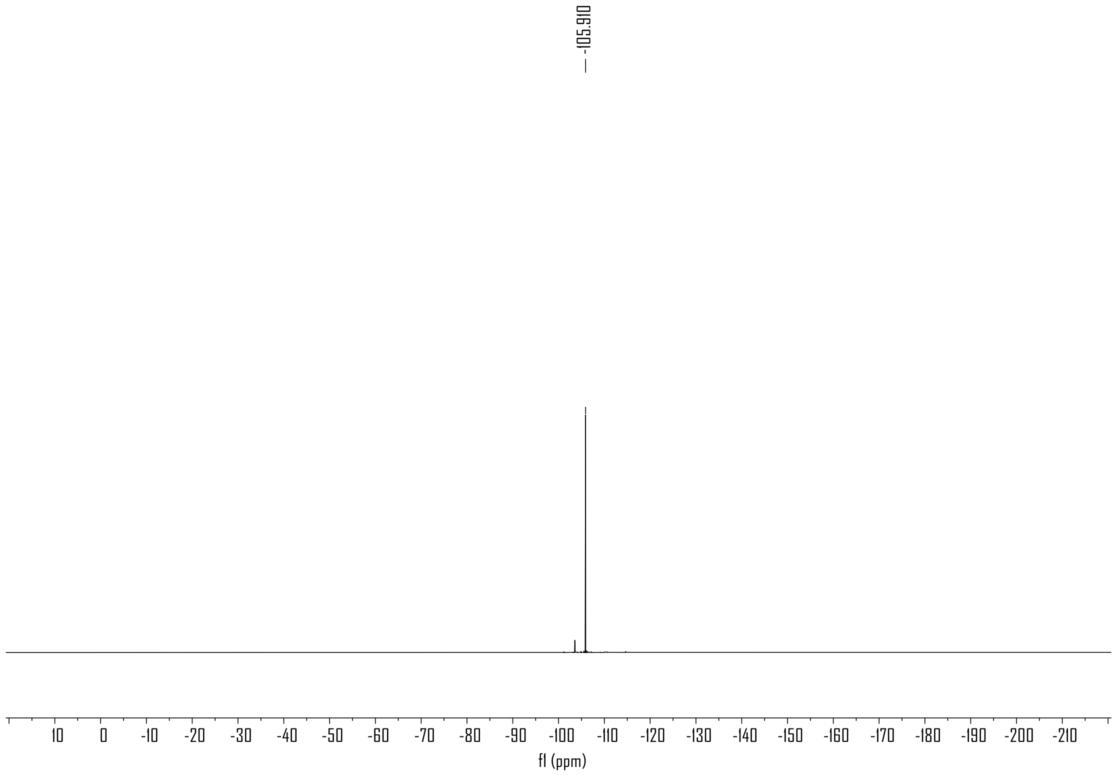

-105.910
10 0 -10 -20 -30 -40 -50 -60 -70 -80 -90 -100 -110 -120 -130 -140 -150 -160 -170 -180 -190 -200 -210
f1 (ppm)

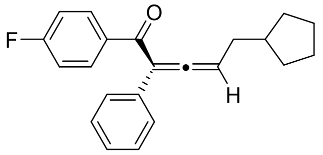

F
O
H

3q

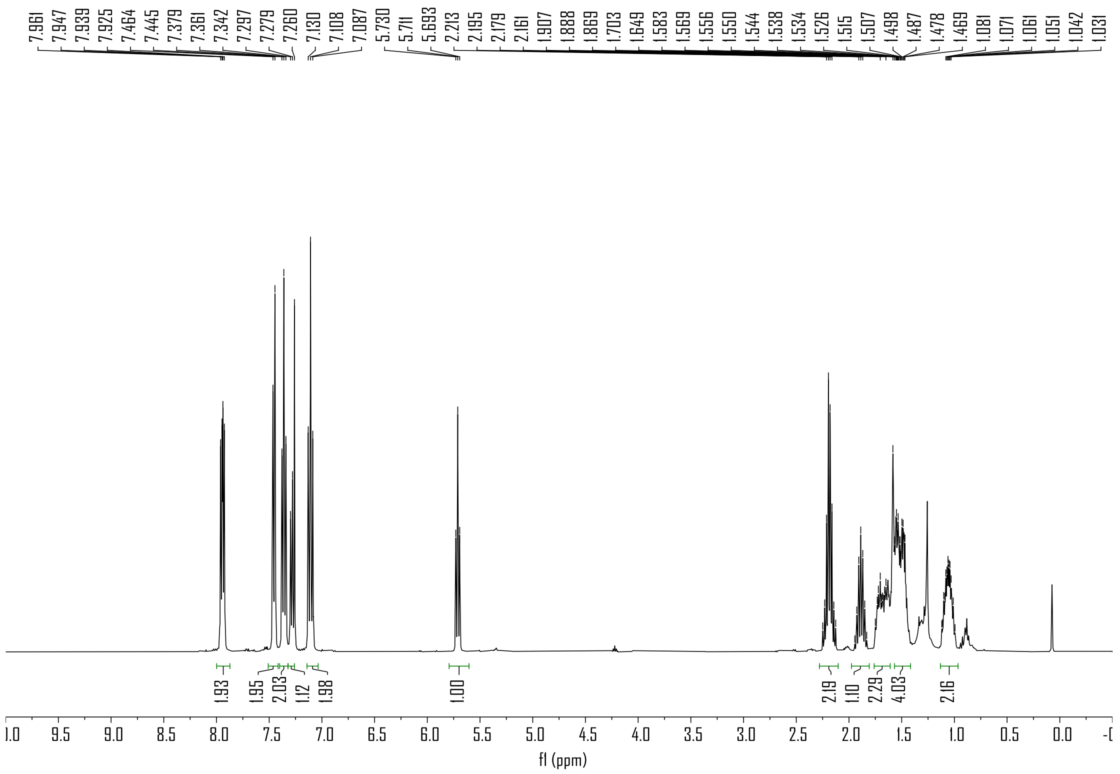
7.961 7.947 7.939 7.925 7.464 7.445 7.379 7.361 7.342 7.297 7.279 7.260 7.130 7.108 7.087 5.730 5.711 5.693 2.213 2.195 2.179 2.161 1.907 1.888 1.869 1.703 1.649 1.583 1.569 1.556 1.550 1.544 1.538 1.534 1.526 1.515 1.507 1.498 1.487 1.478 1.469 1.081 1.071 1.061 1.051 1.042 1.031
1.93 1.95 2.03 1.12 1.98 1.00 2.19 1.10 2.29 4.03 2.16
fl (ppm)

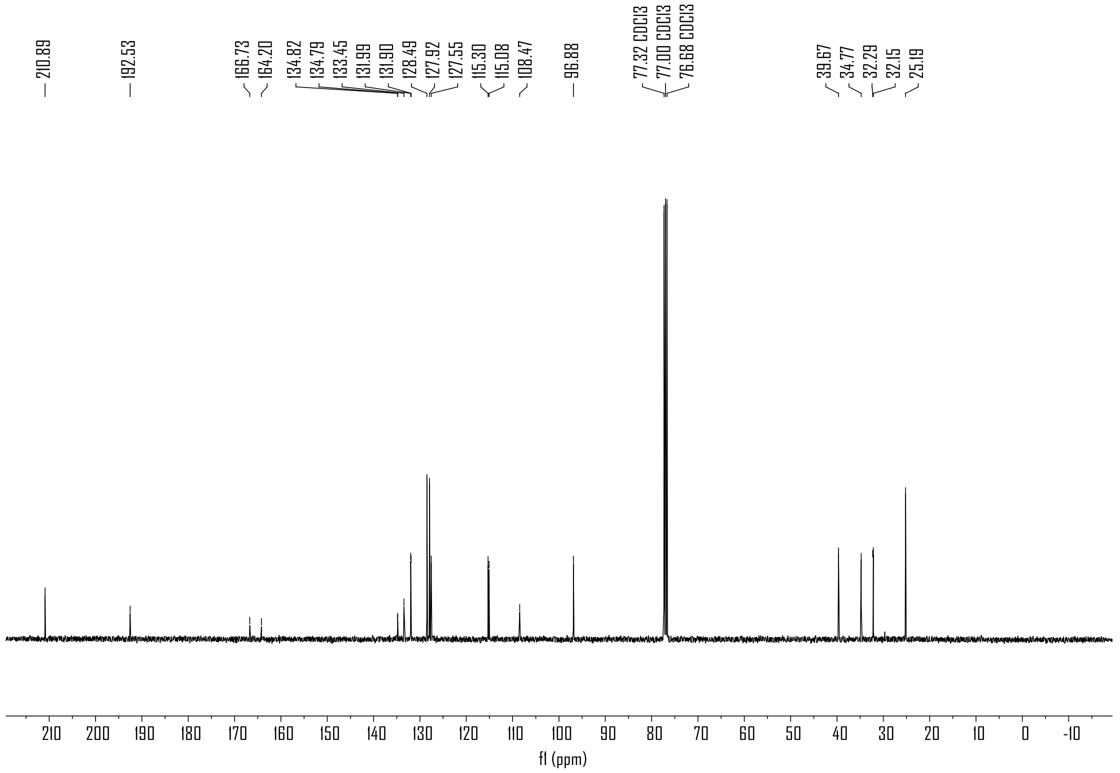
210.89 192.53 166.73 164.20 134.82 134.79 133.45 131.99 131.90 128.49 127.92 127.55 115.30 115.08 108.47 96.88
77.32 CDCl3
77.00 CDCl3
76.68 CDCl3
39.67 34.77 32.29 32.15 25.19
fl (ppm)

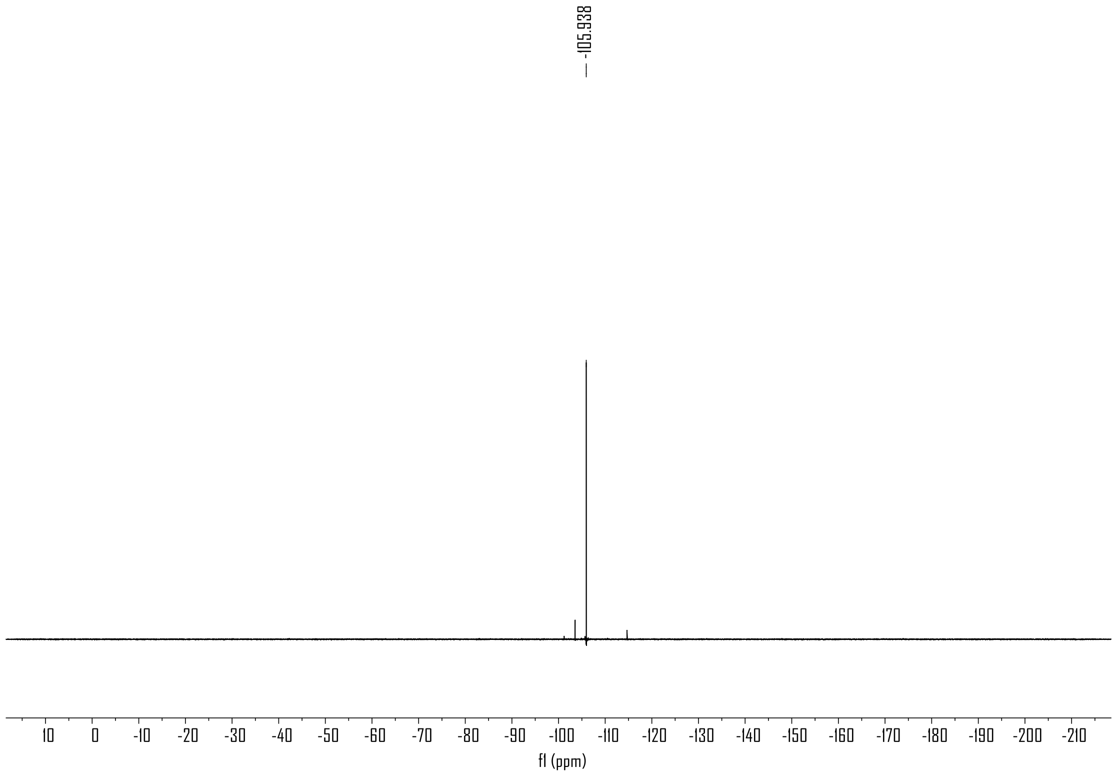
-105.938
10
0
-10
-20
-30
-40
-50
-60
-70
-80
-90
-100
-110
-120
-130
-140
-150
-160
-170
-180
-190
-200
-210
f1 (ppm)

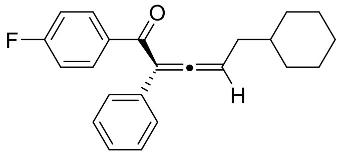
F
O
H

3r

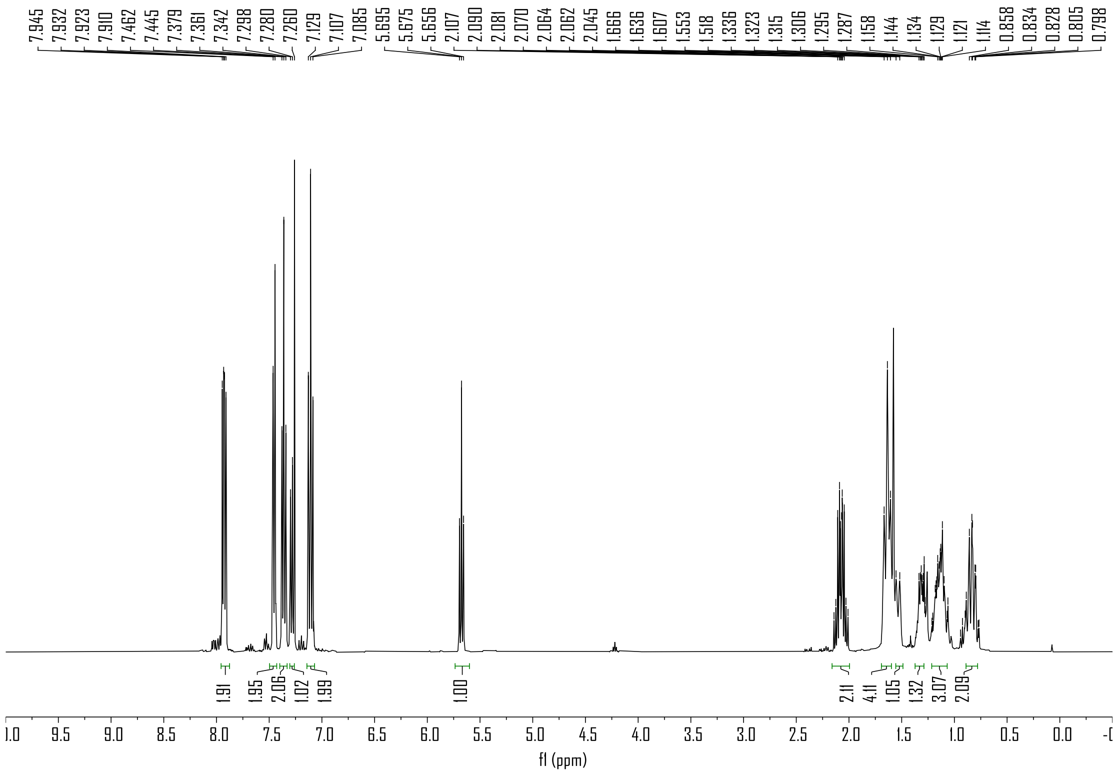

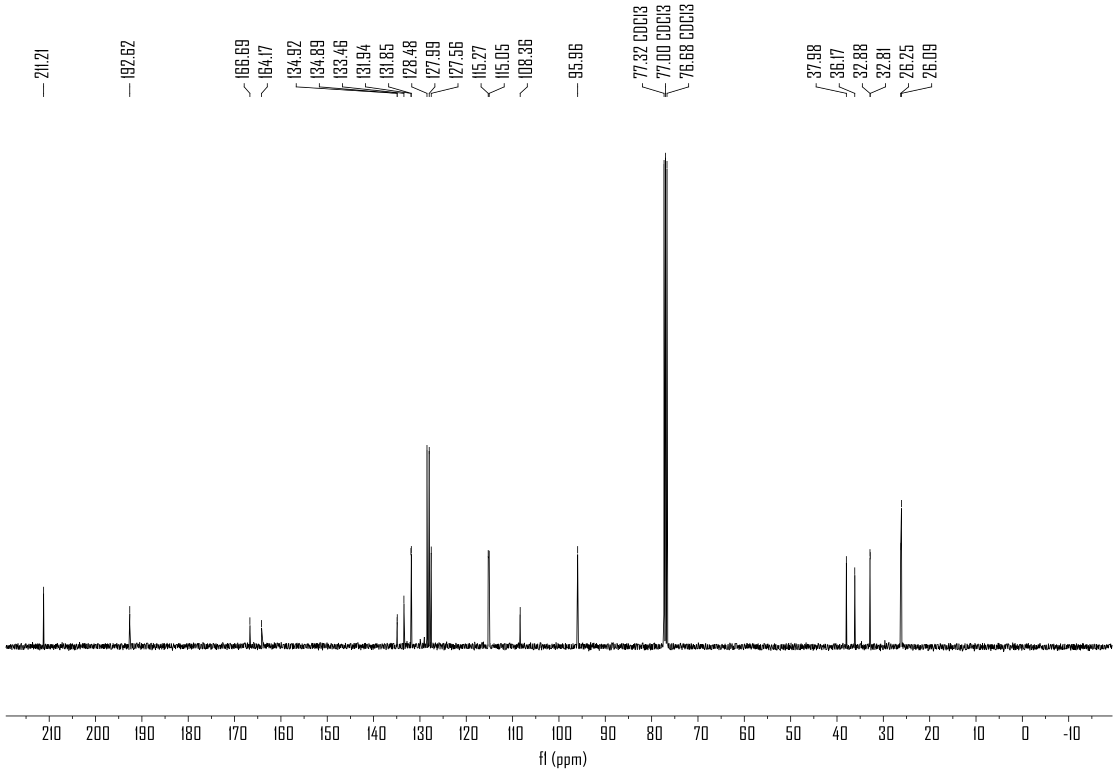

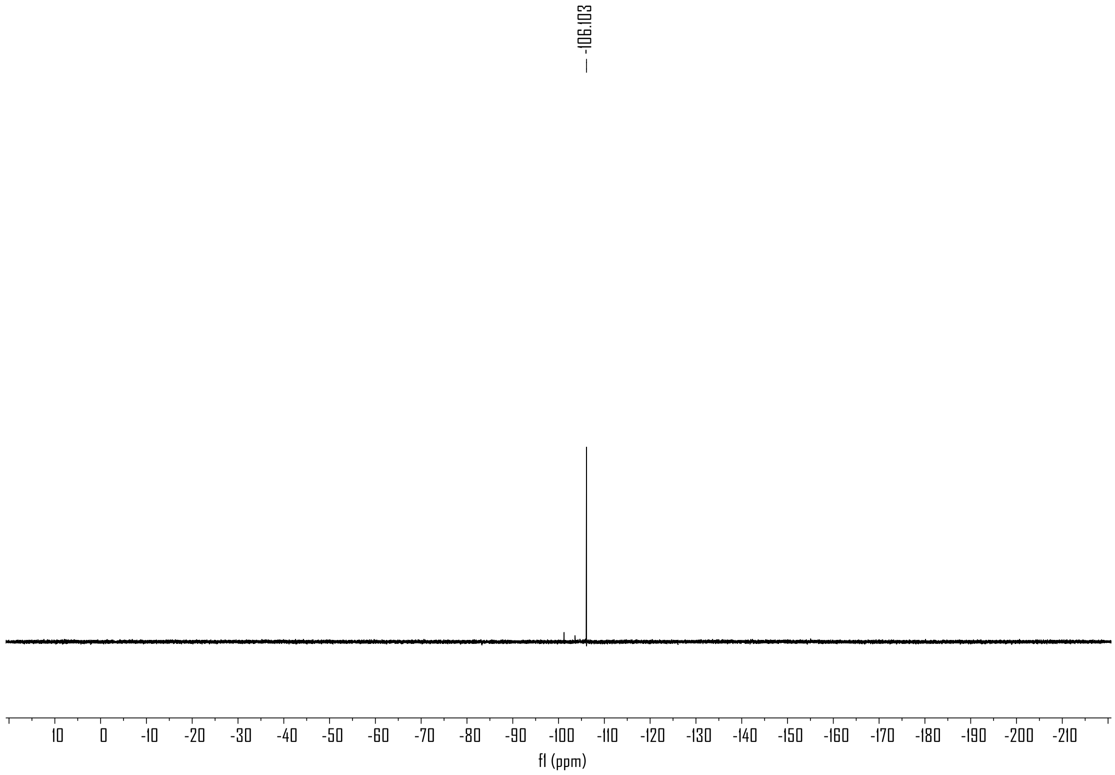
-106.103
10
0
-10
-20
-30
-40
-50
-60
-70
-80
-90
-100
-110
-120
-130
-140
-150
-160
-170
-180
-190
-200
-210
f1 (ppm)

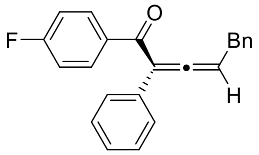
F
O
Bn
H

3s

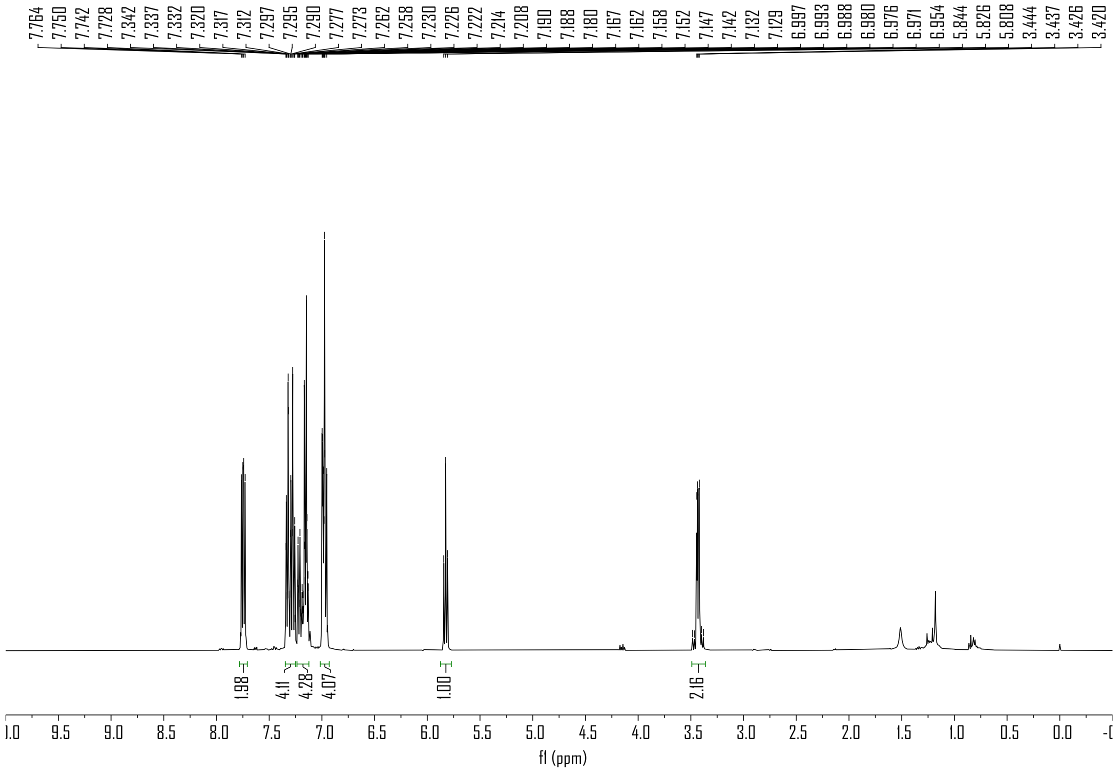
7.764
7.750
7.742
7.728
7.342
7.337
7.332
7.320
7.317
7.312
7.297
7.295
7.290
7.277
7.273
7.262
7.258
7.230
7.226
7.222
7.214
7.208
7.190
7.188
7.180
7.167
7.162
7.158
7.152
7.147
7.142
7.132
7.129
6.997
6.993
6.988
6.980
6.976
6.971
6.954
5.844
5.826
5.808
3.444
3.437
3.426
3.420
1.98
4.11
4.28
4.07
1.00
2.16
f1 (ppm)

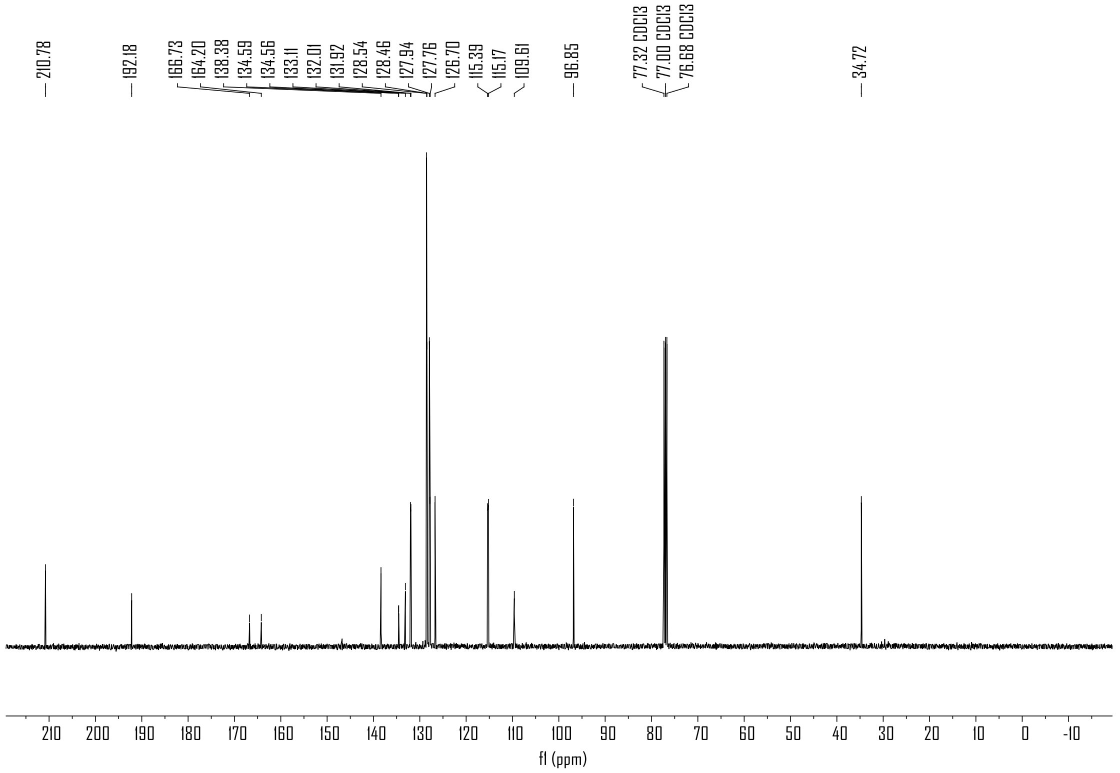
210.78
192.18
166.73
164.20
138.38
134.59
134.56
133.11
132.01
131.92
128.54
128.46
127.94
127.76
126.70
115.39
115.17
109.61
96.85
77.32 CDCl3
77.00 CDCl3
76.68 CDCl3
34.72
f1 (ppm)

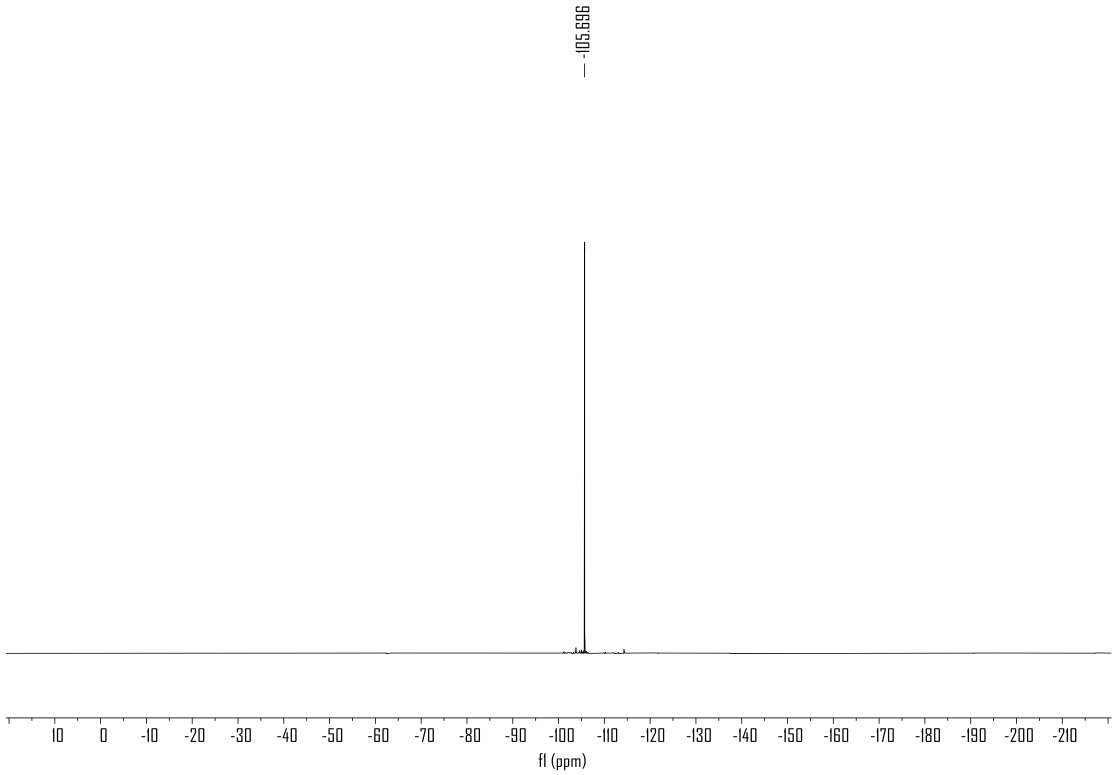

-105.696
10
0
-10
-20
-30
-40
-50
-60
-70
-80
-90
-100
-110
-120
-130
-140
-150
-160
-170
-180
-190
-200
-210
f1 (ppm)

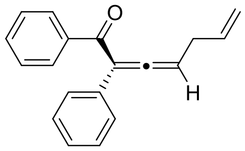

O
H

3t

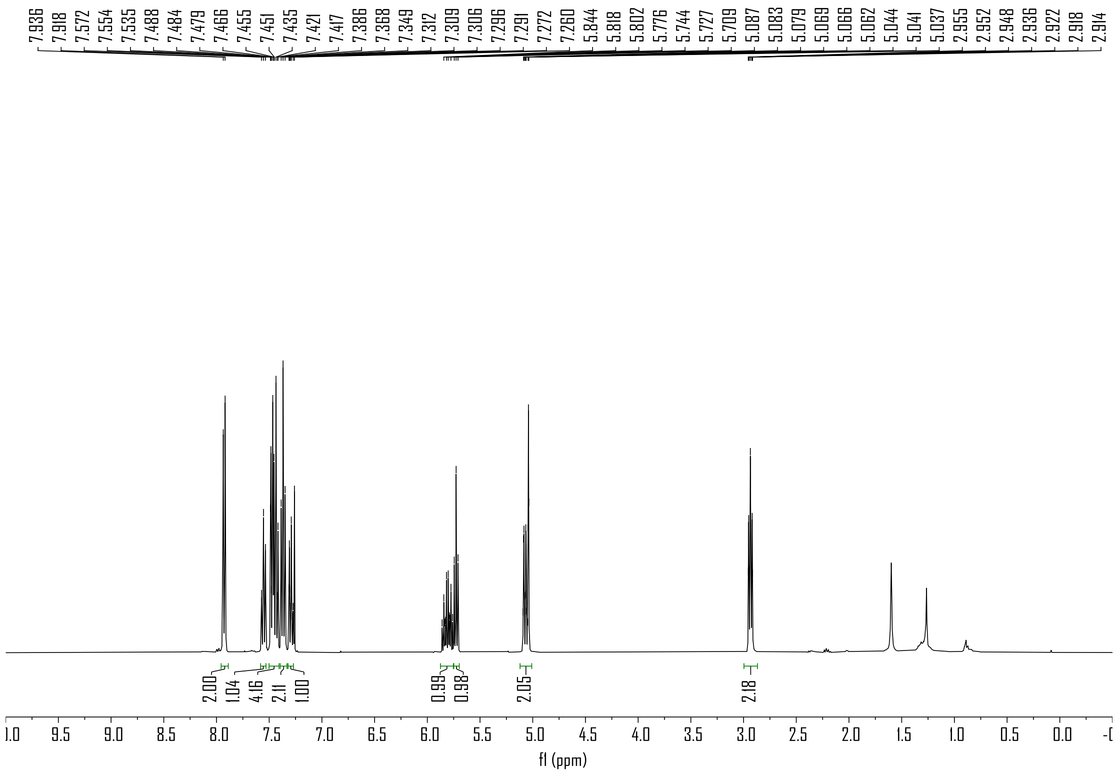
7.936 7.918 7.572 7.554 7.535 7.488 7.484 7.479 7.466 7.455 7.451 7.435 7.421 7.417 7.386 7.368 7.349 7.312 7.309 7.306 7.296 7.291 7.272 7.260 5.844 5.818 5.802 5.776 5.744 5.727 5.709 5.087 5.083 5.079 5.069 5.066 5.062 5.044 5.041 5.037 2.955 2.952 2.948 2.936 2.922 2.918 2.914
2.00 1.04 4.16 2.11 1.00 0.99 0.98 2.05 2.18
f1 (ppm)

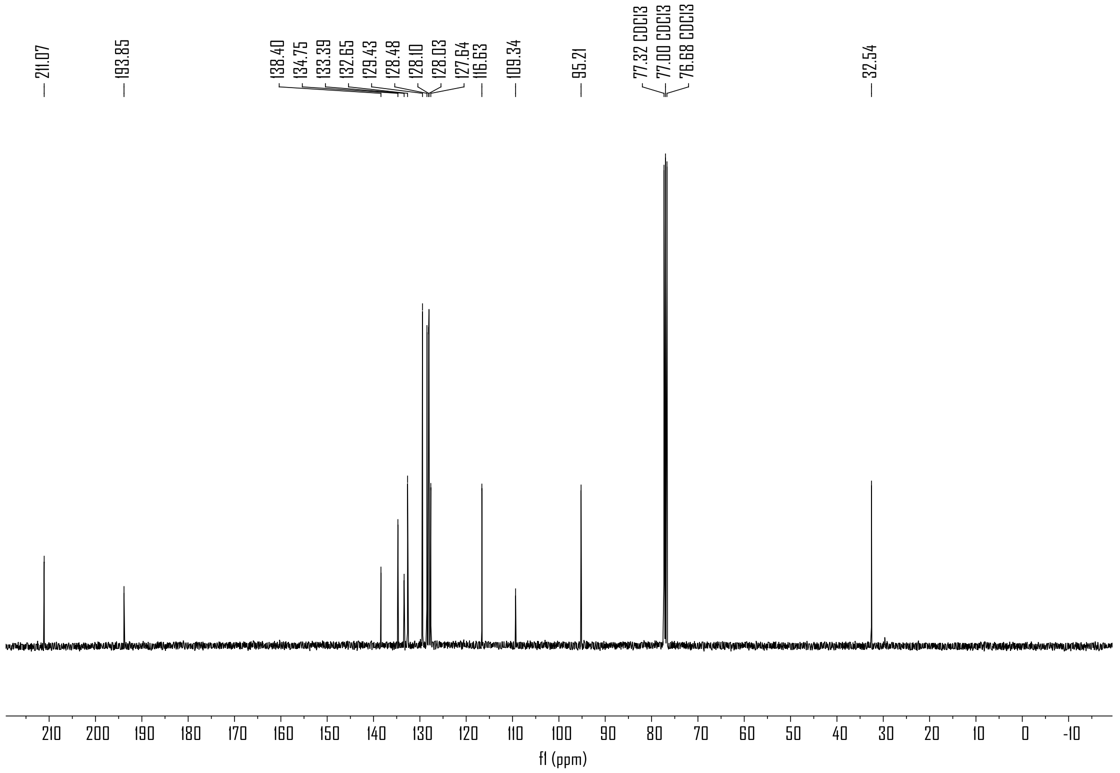
211.07
193.85
138.40 134.75 133.39 132.65 129.43 128.48 128.10 128.03 127.64 116.63
109.34
95.21
77.32 CDCl3
77.00 CDCl3
76.68 CDCl3
32.54
f1 (ppm)

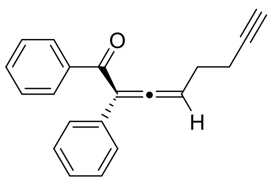
O
H

3u

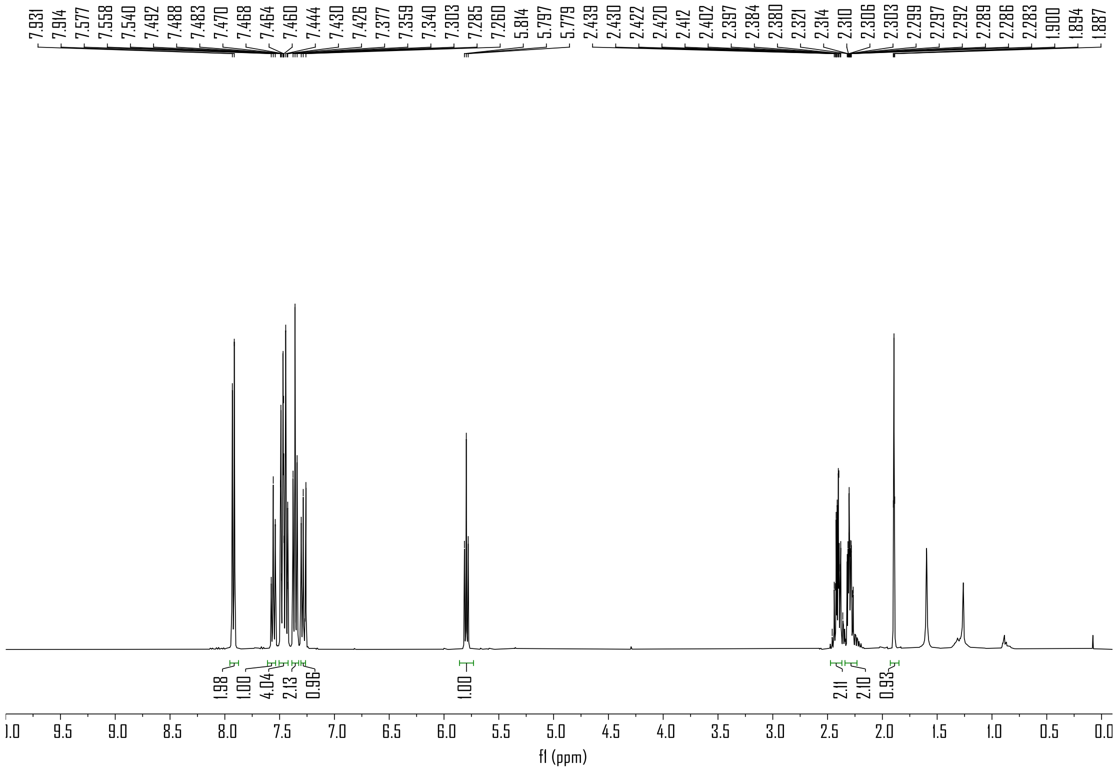

7.931
7.914
7.577
7.558
7.540
7.492
7.488
7.483
7.470
7.468
7.464
7.460
7.444
7.430
7.426
7.377
7.359
7.340
7.303
7.285
7.260
5.814
5.797
5.779
2.439
2.430
2.422
2.420
2.412
2.402
2.397
2.384
2.380
2.321
2.314
2.310
2.306
2.303
2.299
2.297
2.292
2.289
2.286
2.283
1.900
1.894
1.887
1.98
1.00
4.04
2.13
0.96
1.00
2.11
2.10
0.93
f1 (ppm)

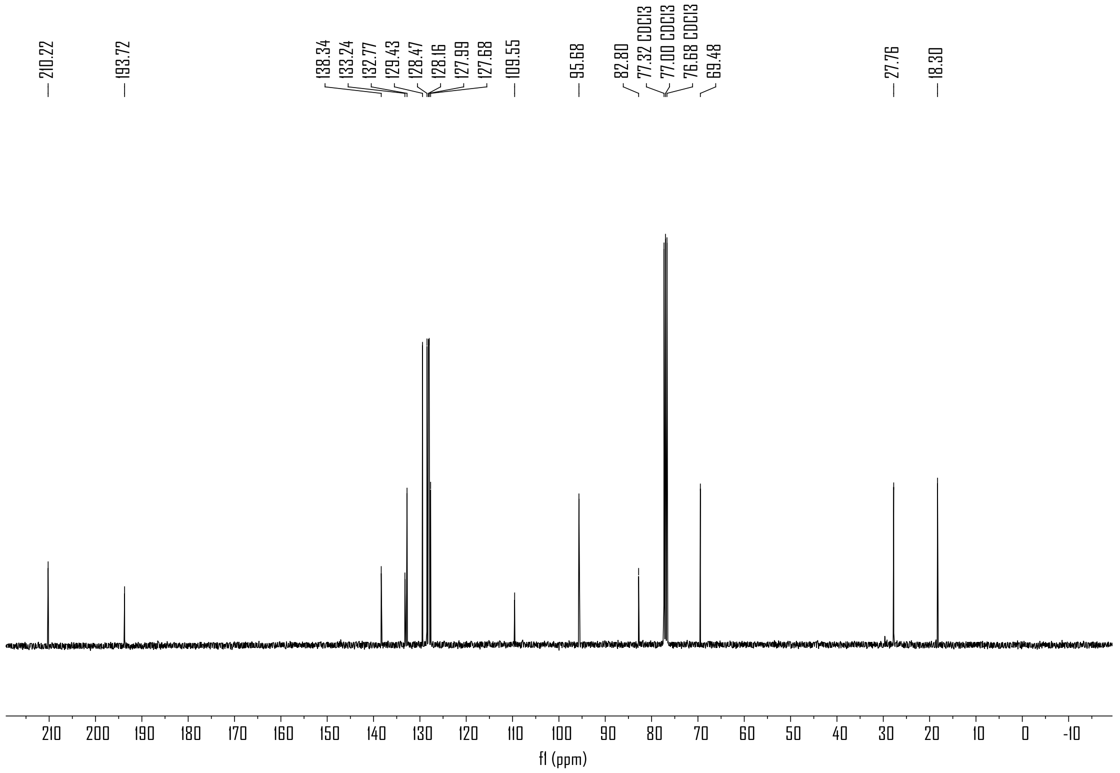

210.22
193.72
138.34
133.24
132.77
129.43
128.47
128.16
127.99
127.68
109.55
95.68
82.80
77.32 CDCl3
77.00 CDCl3
76.68 CDCl3
69.48
27.76
18.30
f1 (ppm)

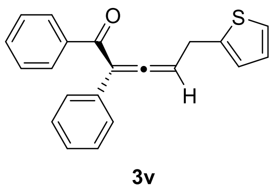

O
S
H

3v

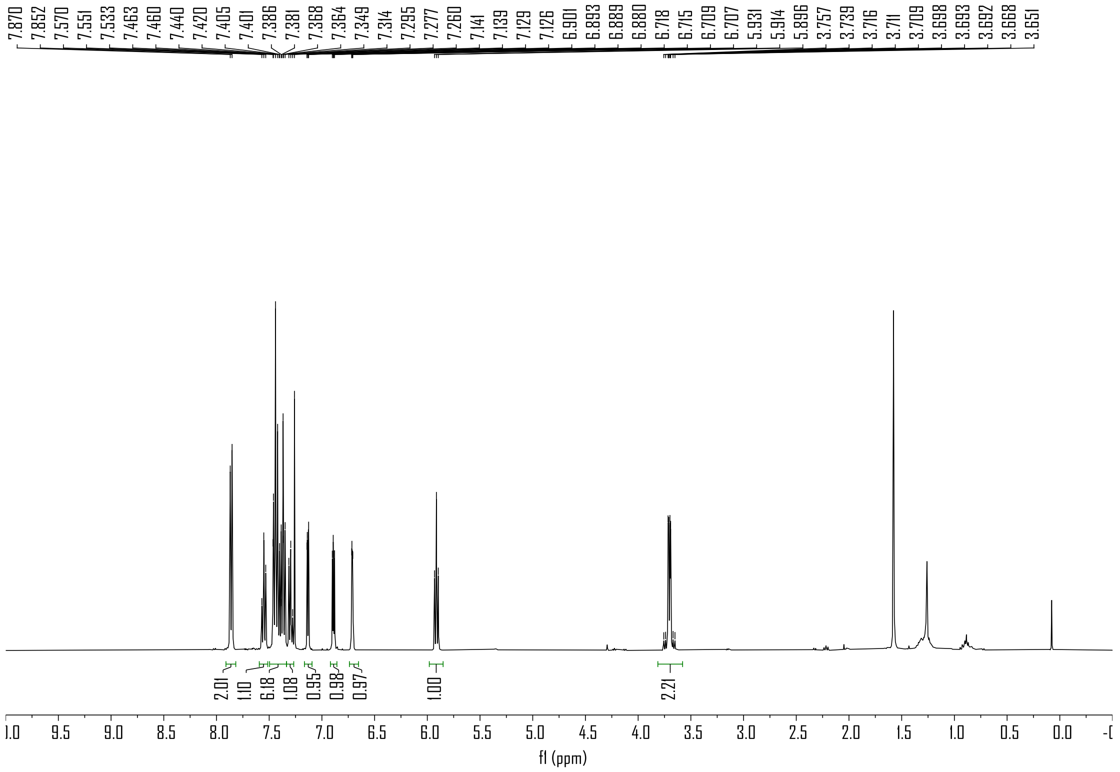
7.870 7.852 7.570 7.551 7.533 7.463 7.460 7.440 7.420 7.405 7.401 7.386 7.381 7.368 7.364 7.349 7.314 7.295 7.277 7.260 7.141 7.139 7.129 7.126 6.901 6.893 6.889 6.880 6.718 6.715 6.709 6.707 5.931 5.914 5.896 3.757 3.739 3.716 3.711 3.709 3.698 3.693 3.692 3.668 3.651
2.01 1.10 6.18 1.08 0.95 0.98 0.97 1.00 2.21
f1 (ppm)

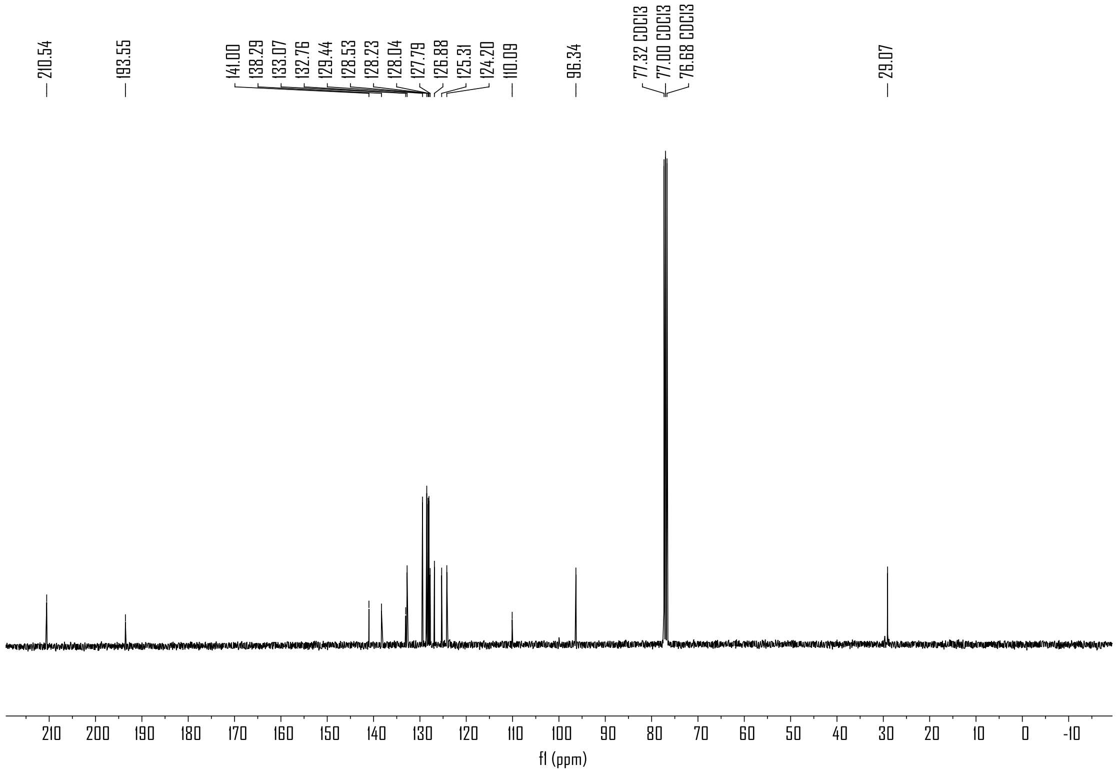
210.54 193.55 141.00 138.29 133.07 132.76 129.44 128.53 128.23 128.04 127.79 126.88 125.31 124.20 110.09 96.34 77.32 CDCl3 77.00 CDCl3 76.68 CDCl3 29.07
f1 (ppm)

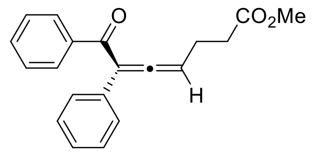
O
CO2Me
H

3w

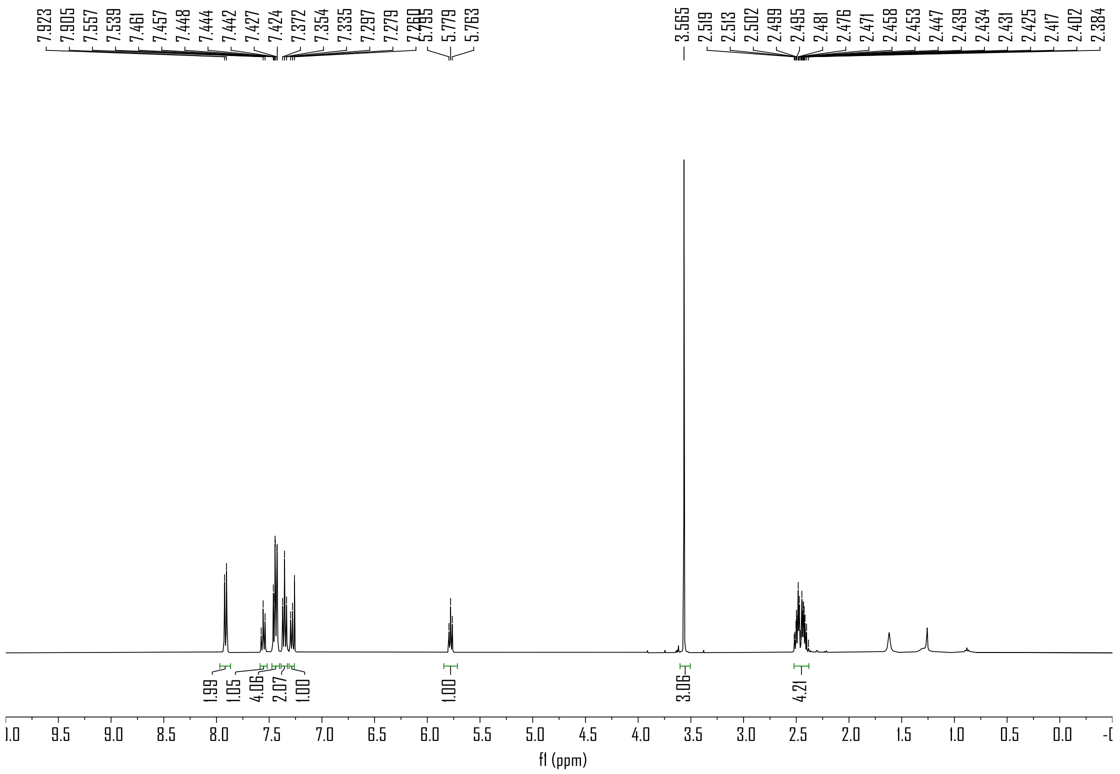
7.923
7.905
7.557
7.539
7.461
7.457
7.448
7.444
7.442
7.427
7.424
7.372
7.354
7.335
7.297
7.279
7.260
5.795
5.779
5.763
3.565
2.519
2.513
2.502
2.499
2.495
2.481
2.476
2.471
2.458
2.453
2.447
2.439
2.434
2.431
2.425
2.417
2.402
2.384
1.99
1.05
4.06
2.07
1.00
1.00
3.06
4.21
fl (ppm)

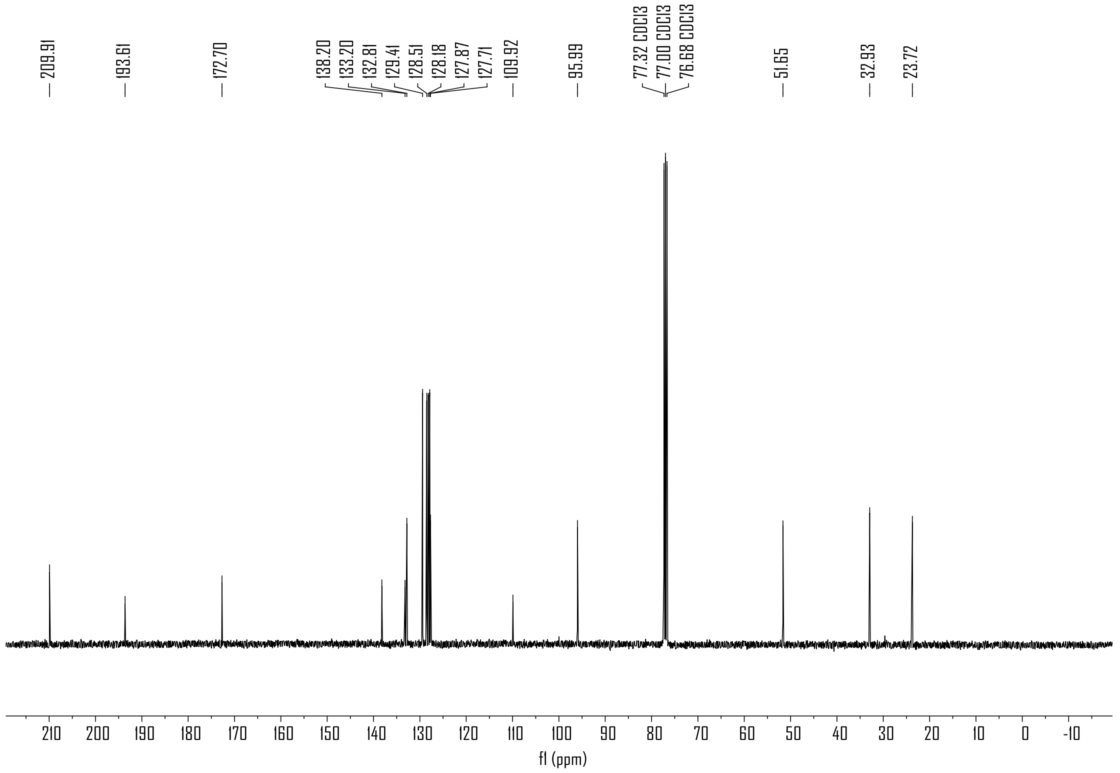
209.91
193.61
172.70
138.20
133.20
132.81
129.41
128.51
128.18
127.87
127.71
109.92
95.99
77.32 CDCl3
77.00 CDCl3
76.68 CDCl3
51.65
32.93
23.72
fl (ppm)

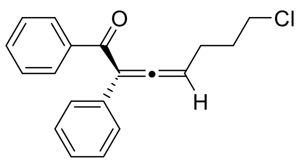
O
Cl
H

3x

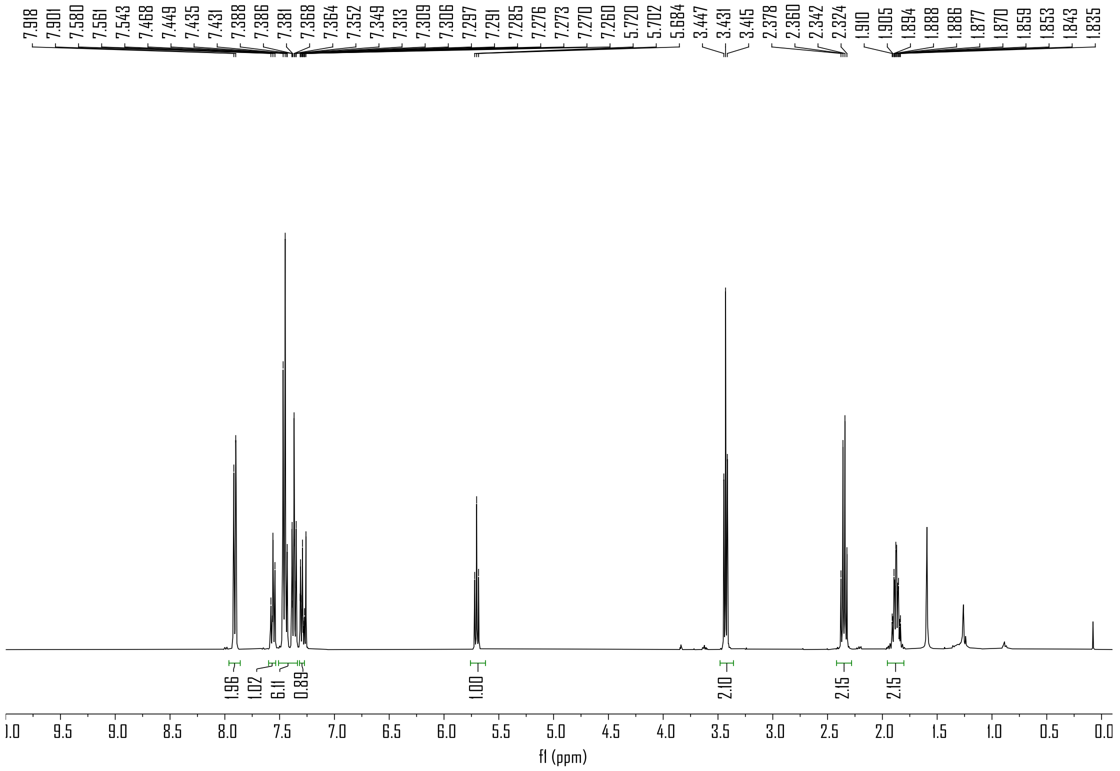

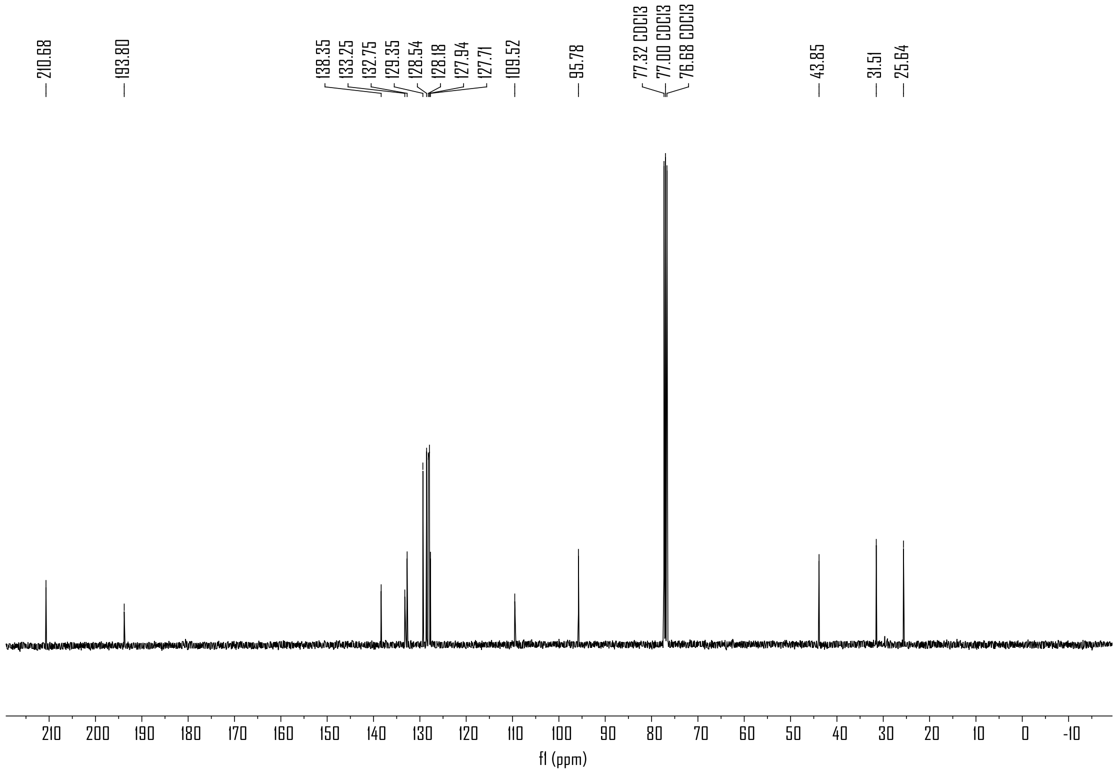

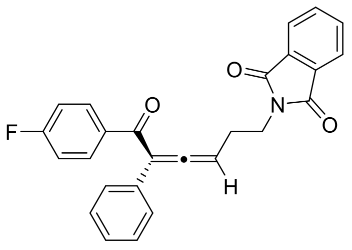
O
N
O
F
O
H

3y

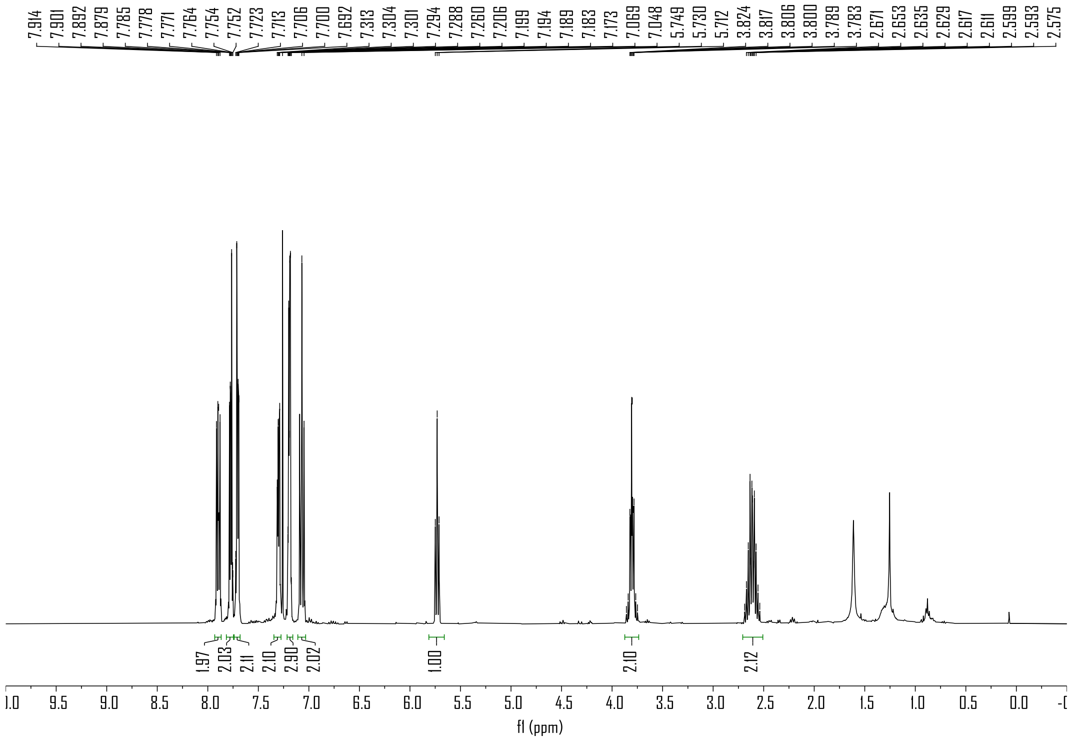
7.914
7.901
7.892
7.879
7.785
7.778
7.771
7.764
7.754
7.752
7.723
7.713
7.706
7.700
7.692
7.313
7.304
7.301
7.294
7.288
7.260
7.206
7.199
7.194
7.189
7.183
7.173
7.069
7.048
5.749
5.730
5.712
3.824
3.817
3.806
3.800
3.789
3.783
2.671
2.653
2.635
2.629
2.617
2.611
2.599
2.593
2.575
1.97
2.03
2.11
2.10
2.90
2.02
1.00
2.10
2.12
9.5
9.0
8.5
8.0
7.5
7.0
6.5
6.0
5.5
5.0
4.5
4.0
3.5
3.0
2.5
2.0
1.5
1.0
0.5
0.0
f1 (ppm)

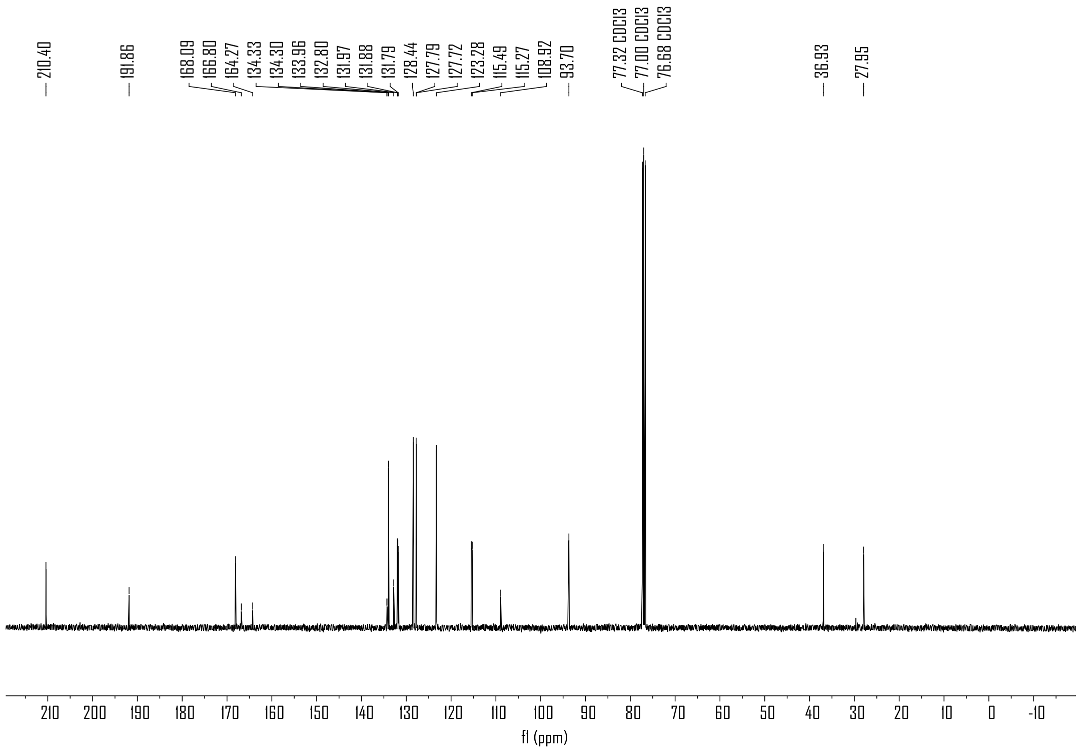
210.40
191.86
168.09
166.80
164.27
134.33
134.30
133.96
132.80
131.97
131.88
131.79
128.44
127.79
127.72
123.28
115.49
115.27
108.92
93.70
77.32 CDCl3
77.00 CDCl3
76.68 CDCl3
36.93
27.95
210 200 190 180 170 160 150 140 130 120 110 100 90 80 70 60 50 40 30 20 10 0 -10
f1 (ppm)

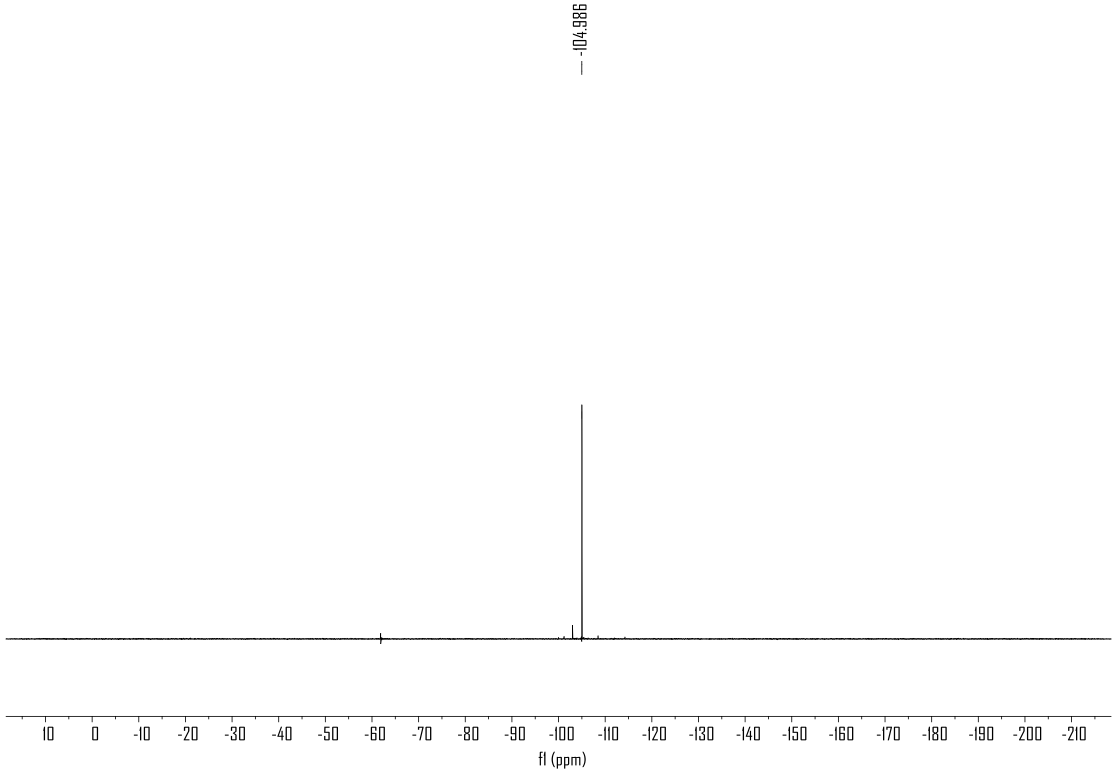
-104.986
10 0 -10 -20 -30 -40 -50 -60 -70 -80 -90 -100 -110 -120 -130 -140 -150 -160 -170 -180 -190 -200 -210
f1 (ppm)

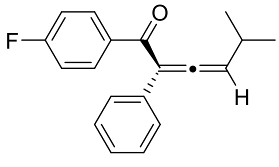
F
O
H

3z

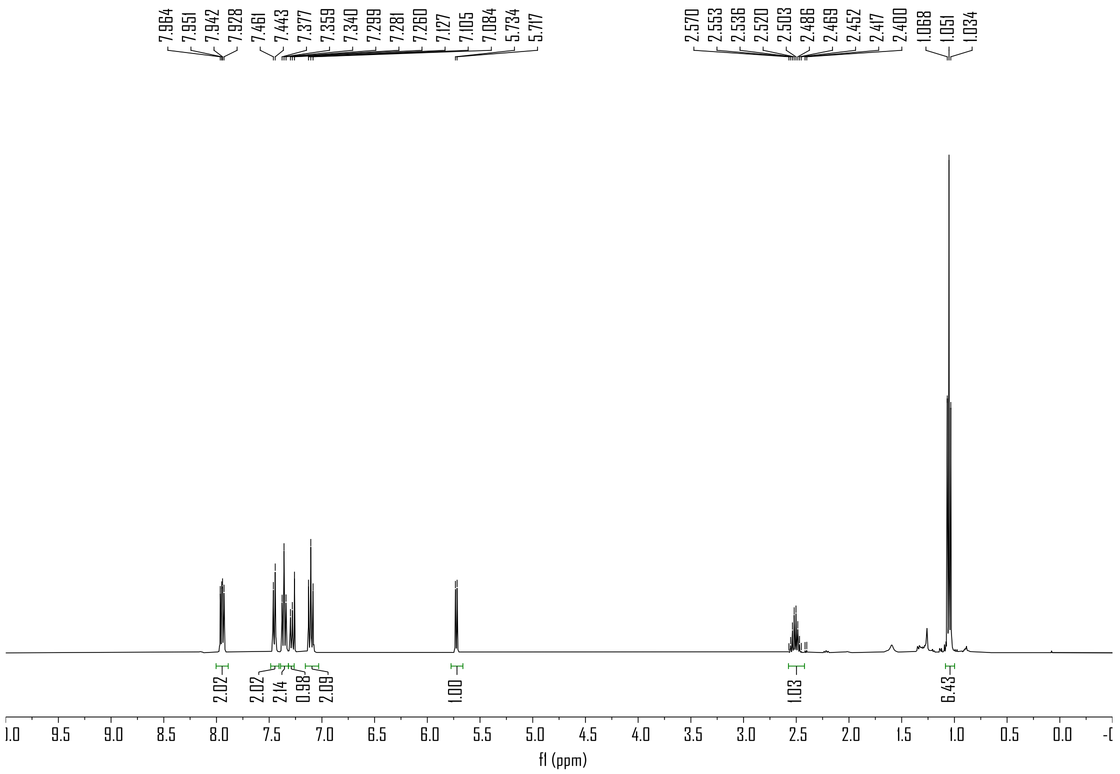

7.964
7.951
7.942
7.928
7.461
7.443
7.377
7.359
7.340
7.299
7.281
7.260
7.127
7.105
7.084
5.734
5.717
2.570
2.553
2.536
2.520
2.503
2.486
2.469
2.452
2.417
2.400
1.068
1.051
1.034
2.02
2.02
2.14
0.98
2.09
1.00
1.03
6.43
9.5
9.0
8.5
8.0
7.5
7.0
6.5
6.0
5.5
5.0
4.5
4.0
3.5
3.0
2.5
2.0
1.5
1.0
0.5
0.0
f1 (ppm)

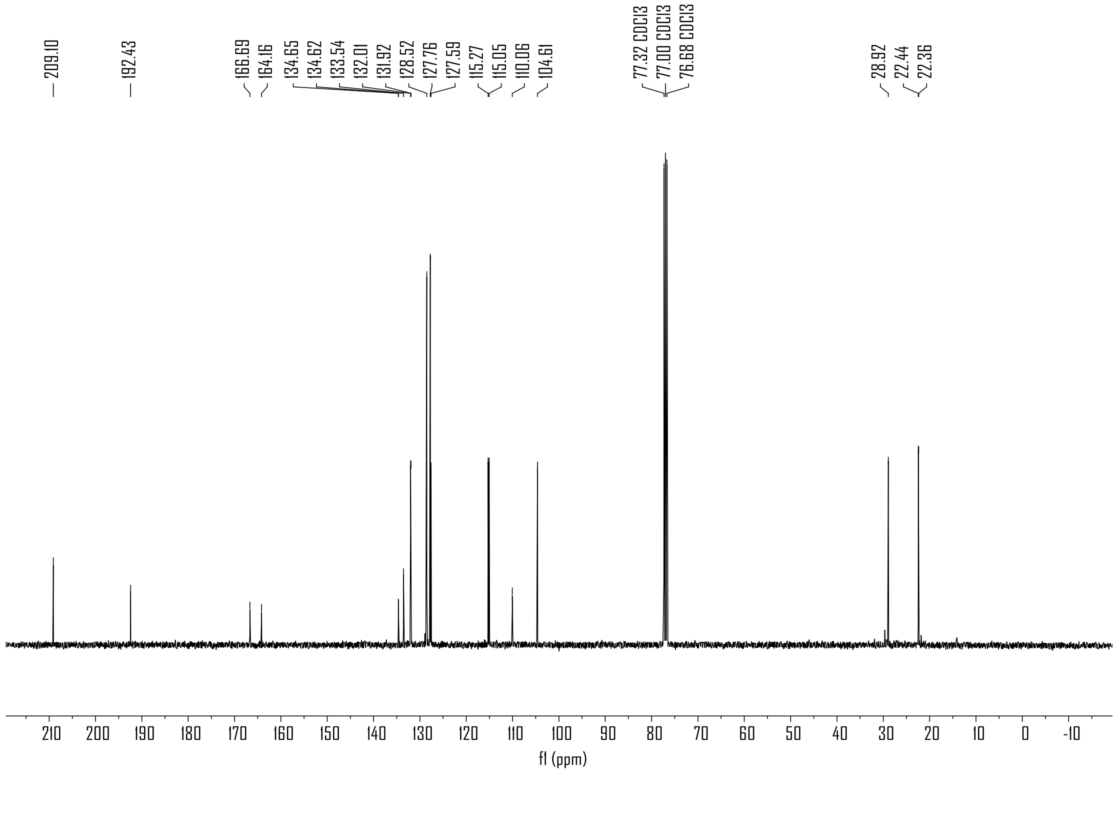

209.10
192.43
166.69
164.16
134.65
134.62
133.54
132.01
131.92
128.52
127.76
127.59
115.27
115.05
110.06
104.61
77.32 CDCl3
77.00 CDCl3
76.68 CDCl3
28.92
22.44
22.36
210
200
190
180
170
160
150
140
130
120
110
100
90
80
70
60
50
40
30
20
10
0
-10
f1 (ppm)

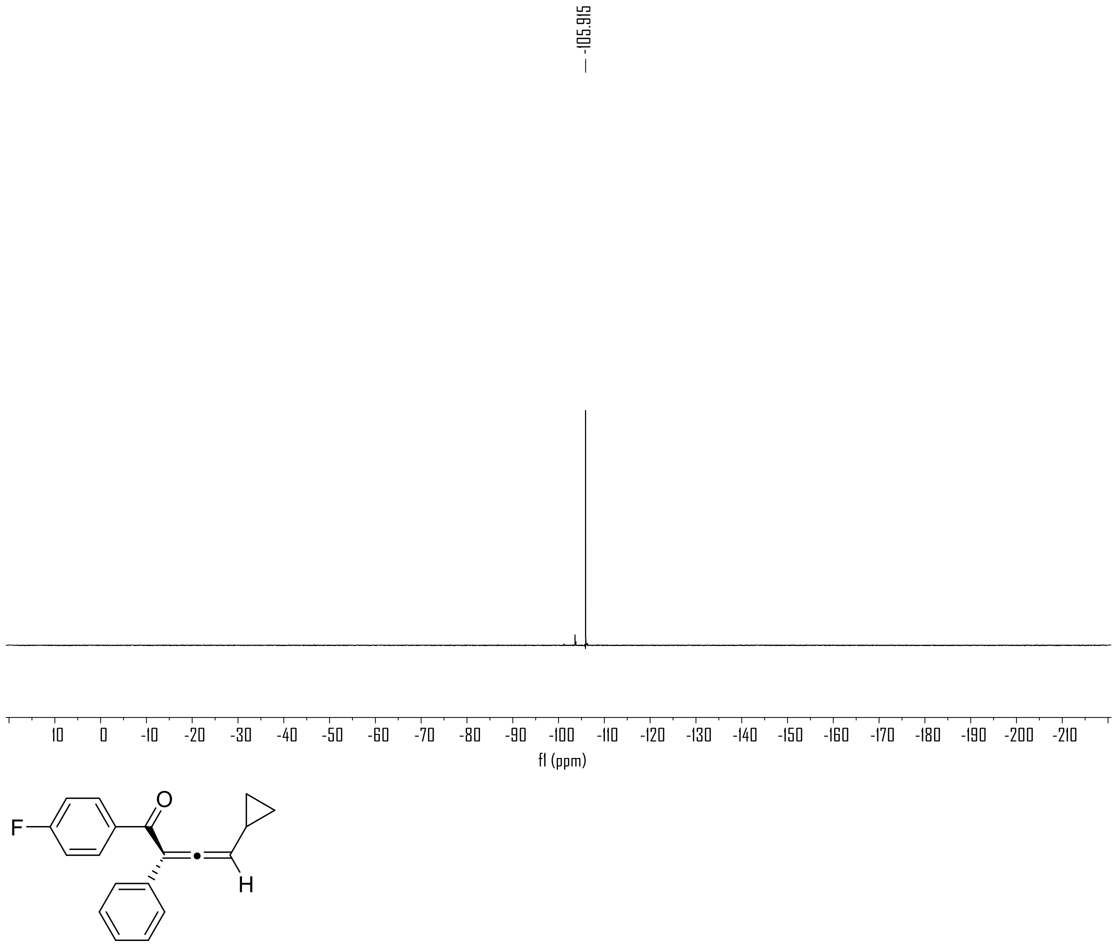
-105.915
10
0
-10
-20
-30
-40
-50
-60
-70
-80
-90
-100
-110
-120
-130
-140
-150
-160
-170
-180
-190
-200
-210
f1 (ppm)
F
O
H

3aa

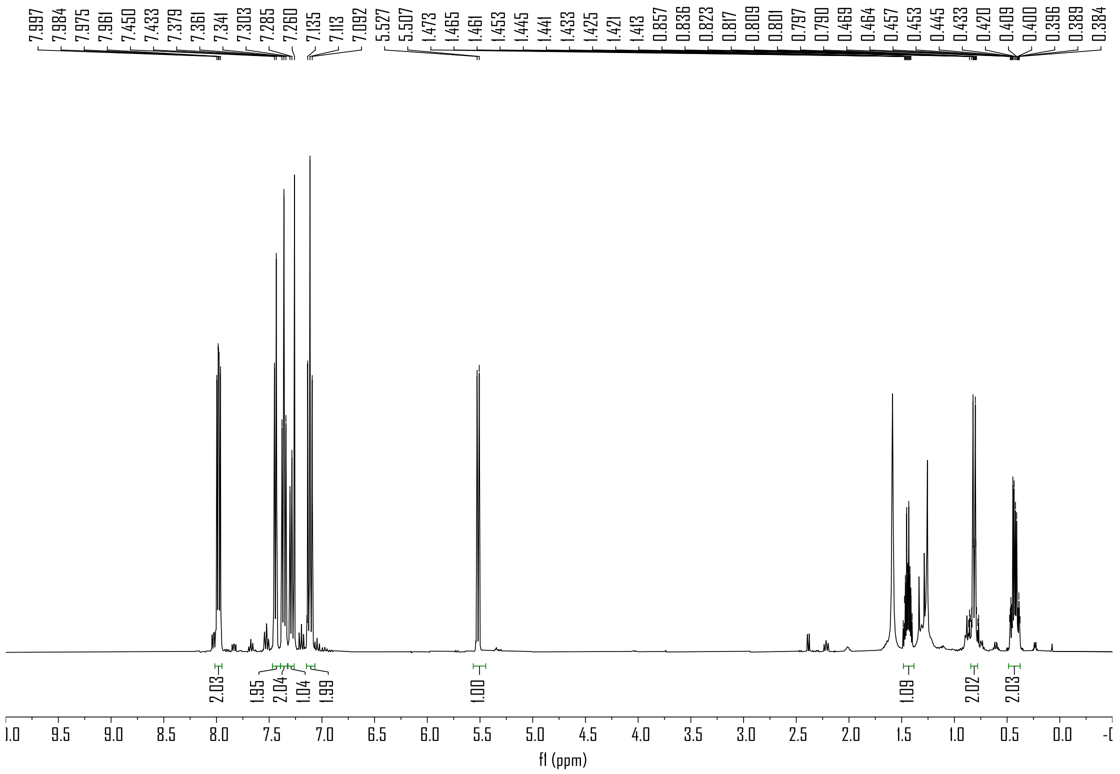
7.997 7.984 7.975 7.961 7.450 7.433 7.379 7.361 7.341 7.303 7.285 7.260 7.135 7.113 7.092 5.527 5.507 1.473 1.465 1.461 1.453 1.445 1.441 1.433 1.425 1.421 1.413 0.857 0.836 0.823 0.817 0.809 0.801 0.797 0.790 0.469 0.464 0.457 0.453 0.445 0.433 0.420 0.409 0.400 0.396 0.389 0.384
2.03 1.95 2.04 1.04 1.99 1.00 1.09 2.02 2.03
f1 (ppm)

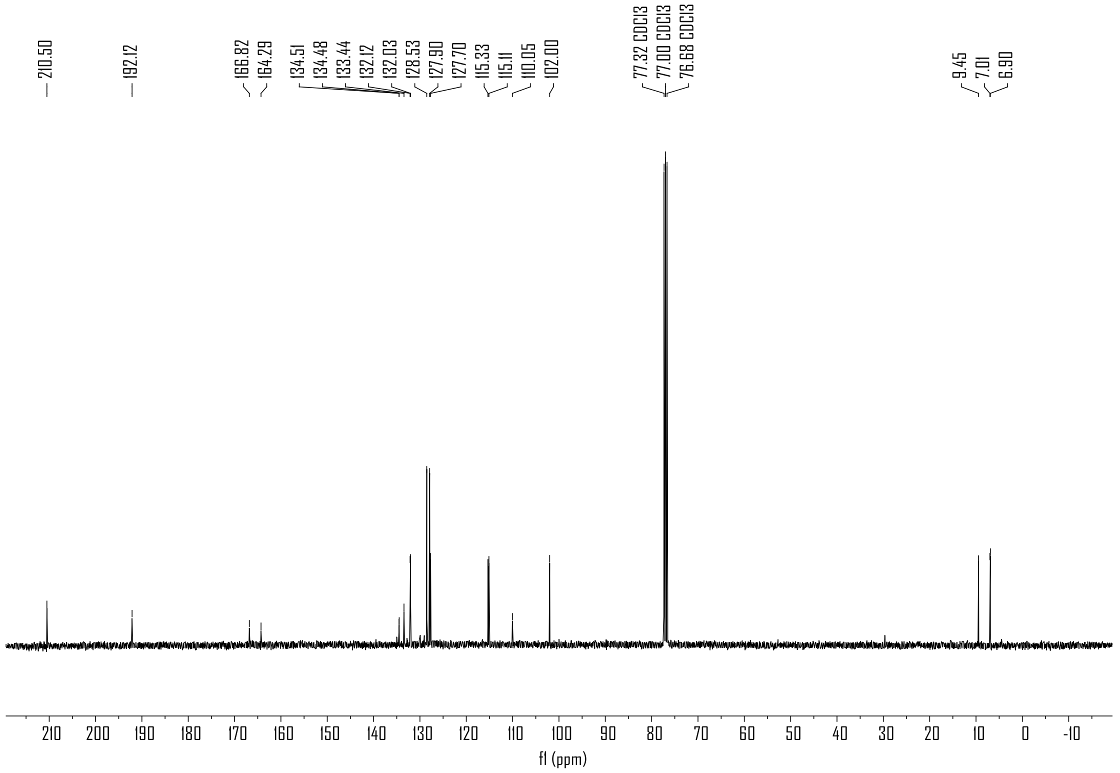
210.50 192.12 166.82 164.29 134.51 134.48 133.44 132.12 132.03 128.53 127.90 127.70 115.33 115.11 110.05 102.00
77.32 CDCl3
77.00 CDCl3
76.68 CDCl3
9.45 7.01 6.90
f1 (ppm)

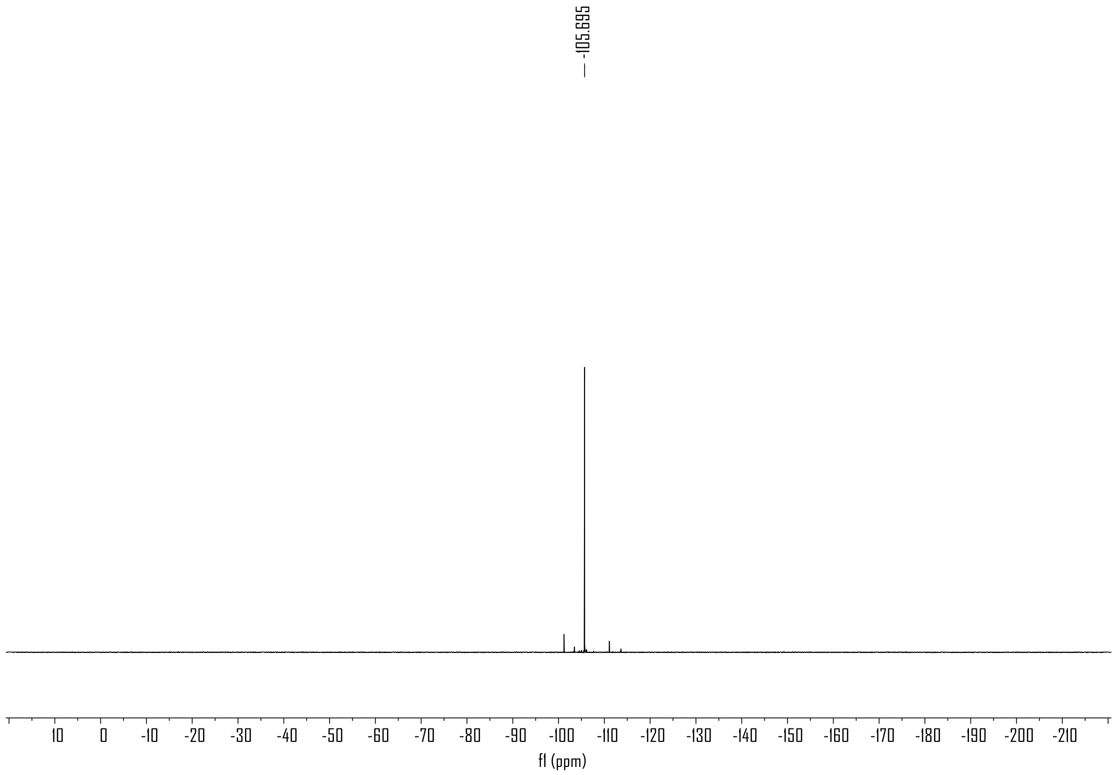

-105.695
10
0
-10
-20
-30
-40
-50
-60
-70
-80
-90
-100
-110
-120
-130
-140
-150
-160
-170
-180
-190
-200
-210
f1 (ppm)

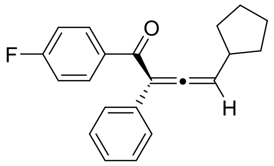

F
O
H

3ab

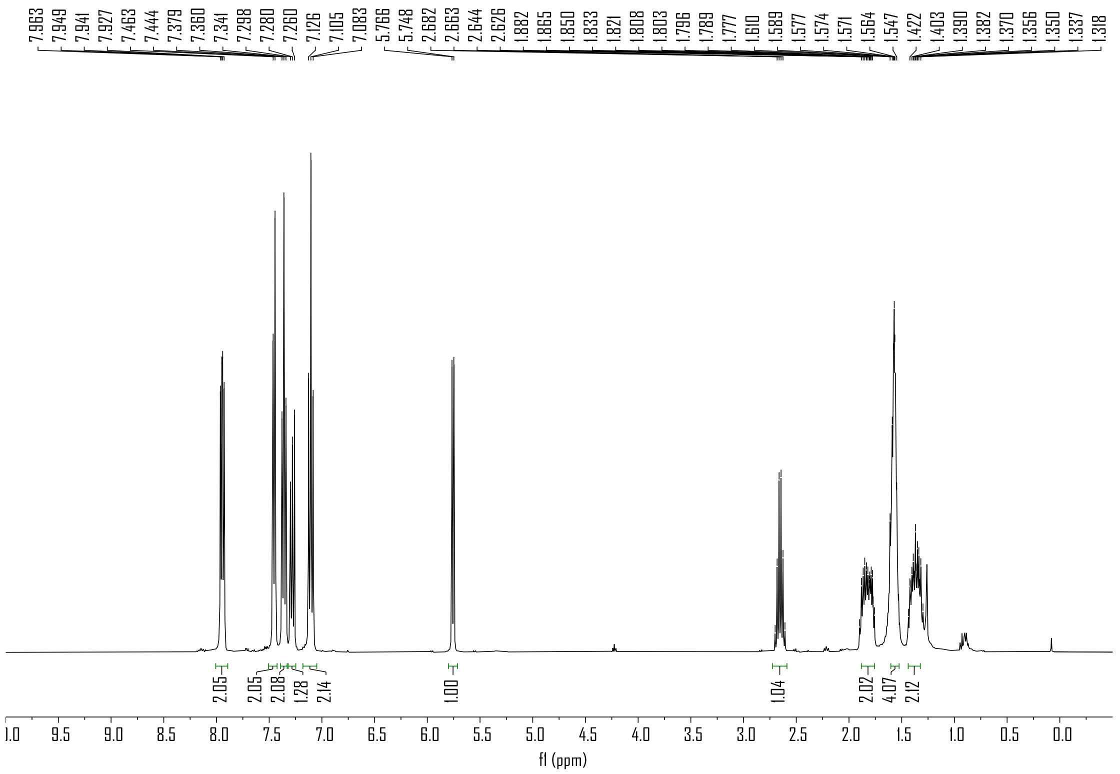
7.963
7.949
7.941
7.927
7.463
7.444
7.379
7.360
7.341
7.298
7.280
7.260
7.126
7.105
7.083
5.766
5.748
2.682
2.663
2.644
2.626
1.882
1.865
1.850
1.833
1.821
1.808
1.803
1.796
1.789
1.777
1.610
1.589
1.577
1.574
1.571
1.564
1.547
1.422
1.403
1.390
1.382
1.370
1.356
1.350
1.337
1.318
2.05
2.05
2.08
1.28
2.14
1.00
1.04
2.02
4.07
2.12
f1 (ppm)

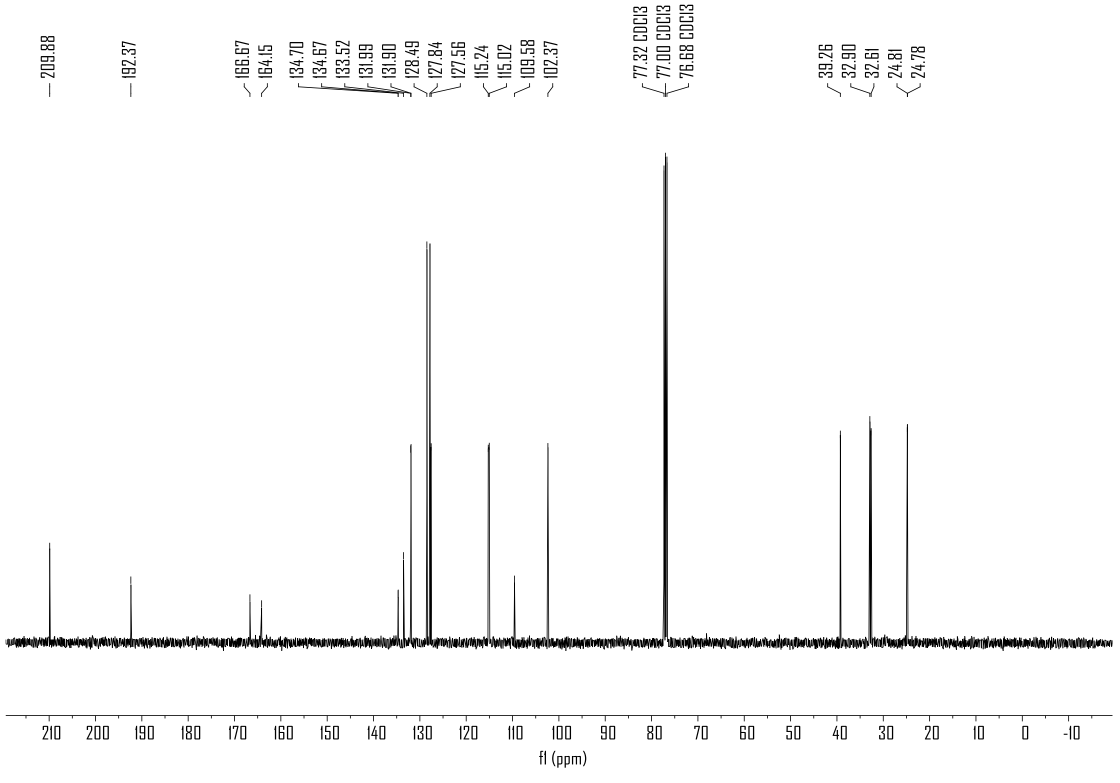
209.88
192.37
166.67
164.15
134.70
134.67
133.52
131.99
131.90
128.49
127.84
127.56
115.24
115.02
109.58
102.37
77.32 CDCl3
77.00 CDCl3
76.68 CDCl3
39.26
32.90
32.61
24.81
24.78
f1 (ppm)

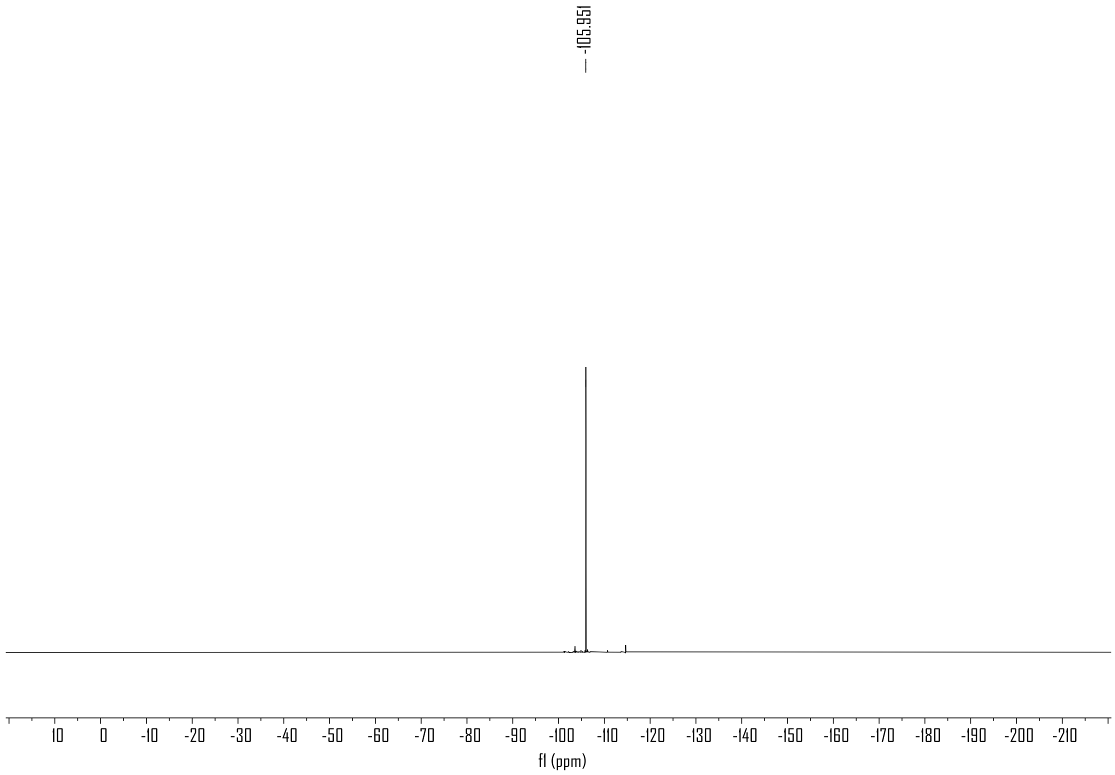
-105.951
10
0
-10
-20
-30
-40
-50
-60
-70
-80
-90
-100
-110
-120
-130
-140
-150
-160
-170
-180
-190
-200
-210
f1 (ppm)

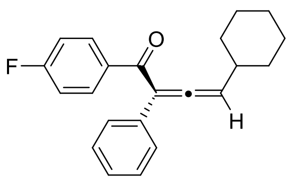
F
O
H

3ac

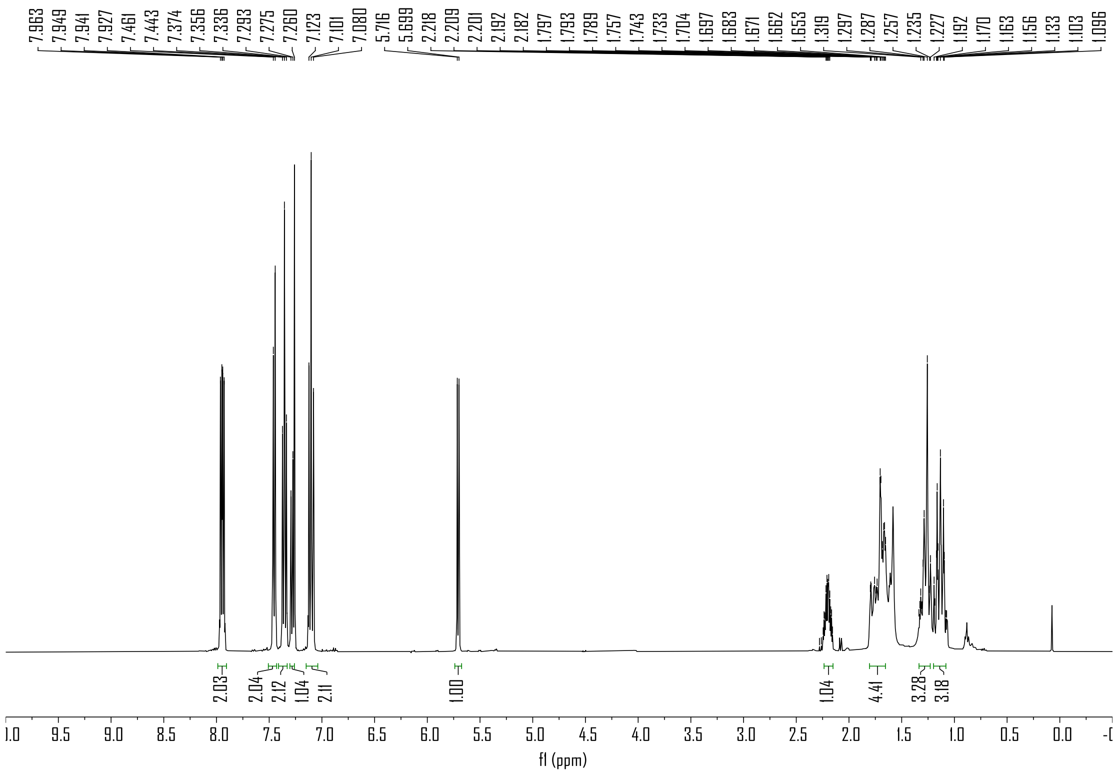
7.963
7.949
7.941
7.927
7.461
7.443
7.374
7.356
7.336
7.293
7.275
7.260
7.123
7.101
7.080
5.716
5.699
2.218
2.209
2.201
2.192
2.182
1.797
1.793
1.789
1.757
1.743
1.733
1.704
1.697
1.683
1.671
1.662
1.653
1.319
1.297
1.287
1.257
1.235
1.227
1.192
1.170
1.163
1.156
1.133
1.103
1.096
2.03
2.04
2.12
1.04
2.11
1.00
1.04
4.41
3.28
3.18
fl (ppm)

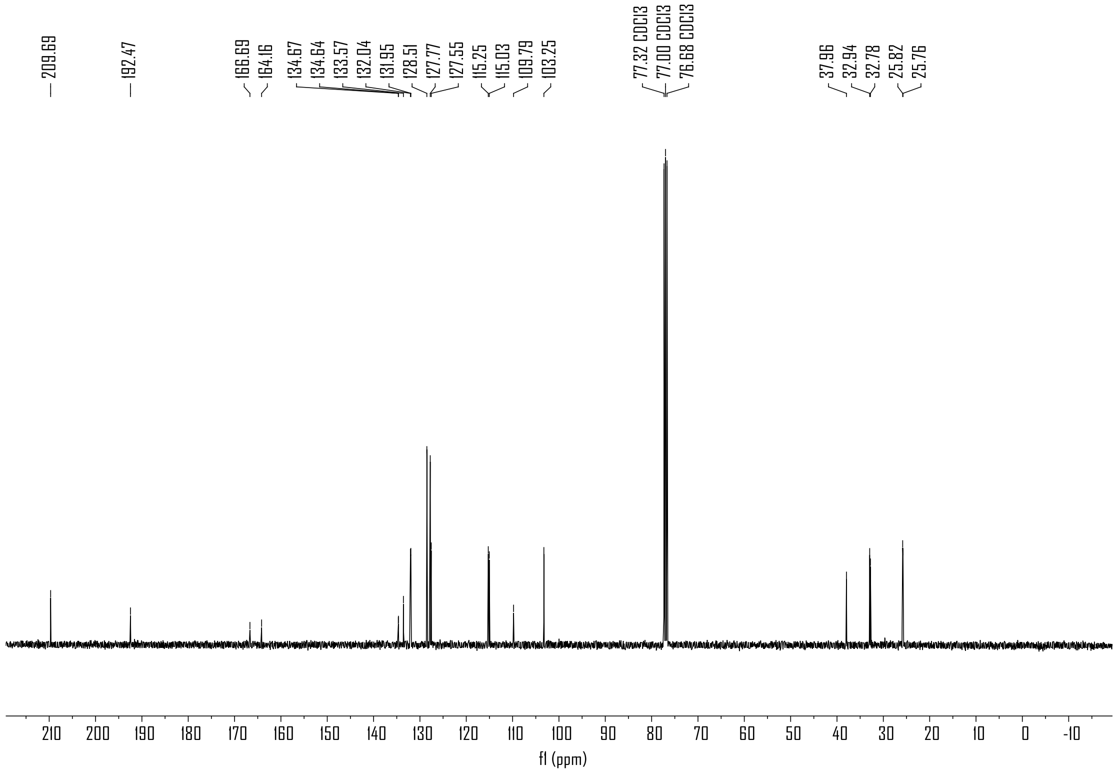
209.69
192.47
166.69
164.16
134.67
134.64
133.57
132.04
131.95
128.51
127.77
127.55
115.25
115.03
109.79
103.25
77.32 CDCl3
77.00 CDCl3
76.68 CDCl3
37.96
32.94
32.78
25.82
25.76
fl (ppm)

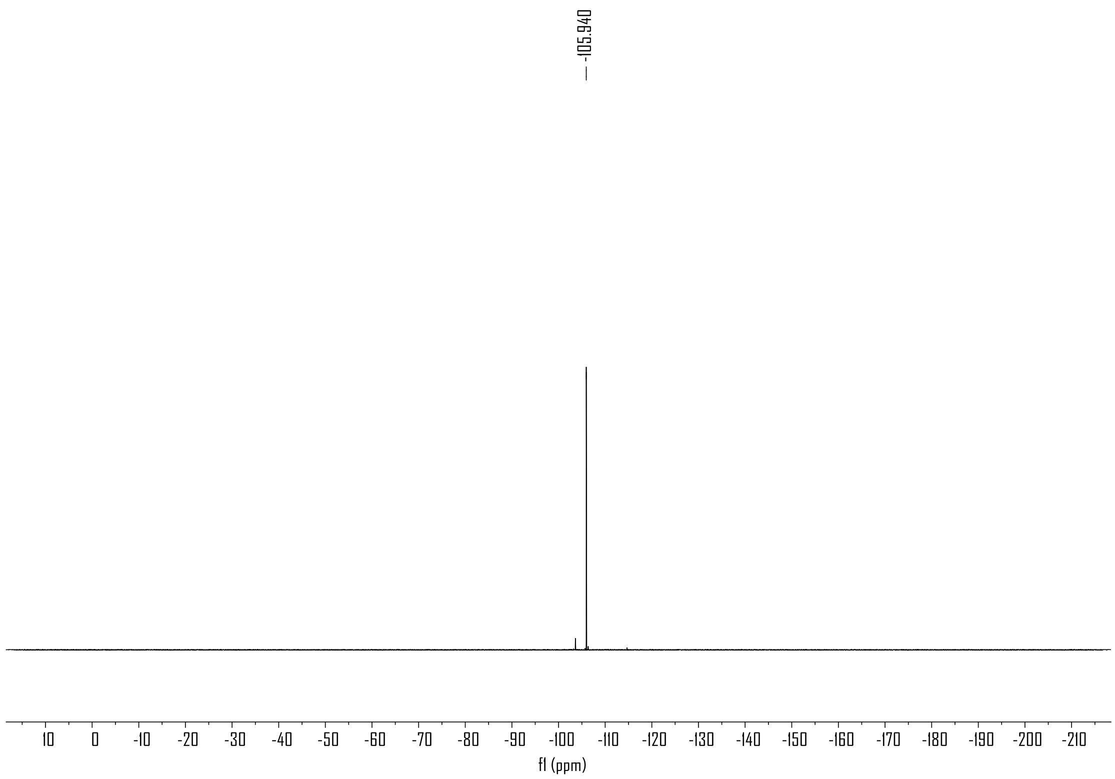
-105.940
10 0 -10 -20 -30 -40 -50 -60 -70 -80 -90 -100 -110 -120 -130 -140 -150 -160 -170 -180 -190 -200 -210
f1 (ppm)

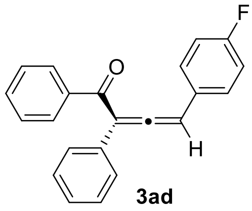
F
O
H

3ad

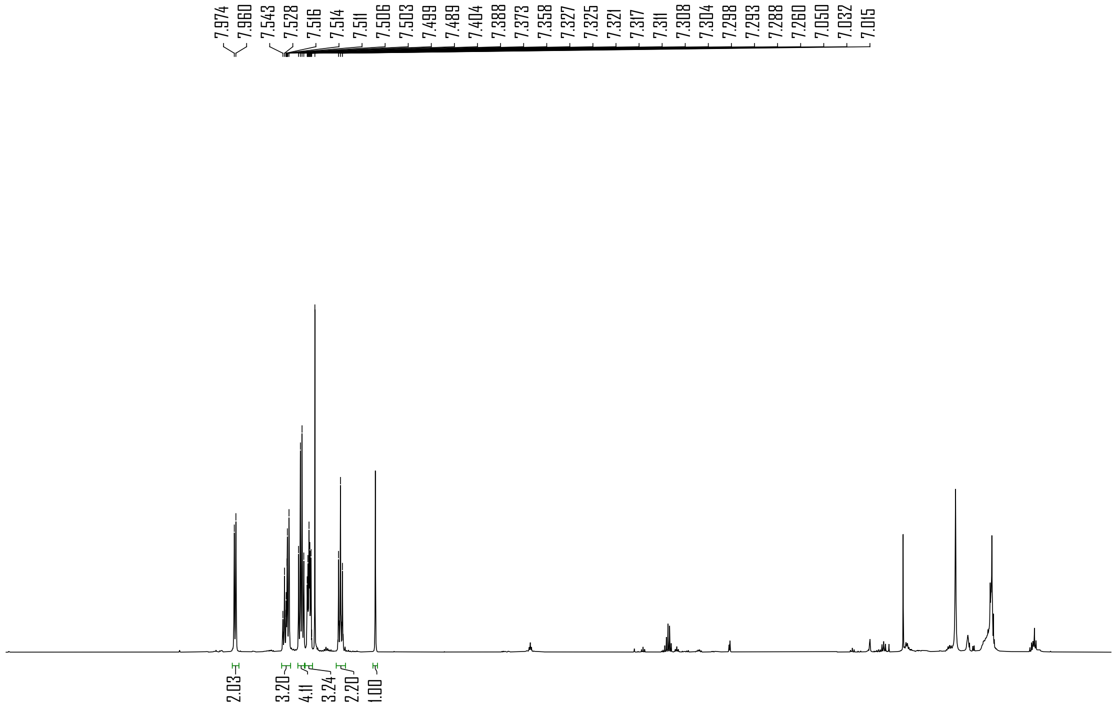
7.974
7.960
7.543
7.528
7.516
7.514
7.511
7.506
7.503
7.499
7.489
7.404
7.388
7.373
7.358
7.327
7.325
7.321
7.317
7.311
7.308
7.304
7.298
7.293
7.288
7.260
7.050
7.032
7.015
2.03
3.20
4.11
3.24
2.20
1.00
9.5 9.0 8.5 8.0 7.5 7.0 6.5 6.0 5.5 5.0 4.5 4.0 3.5 3.0 2.5 2.0 1.5 1.0 0.5
f1 (ppm)

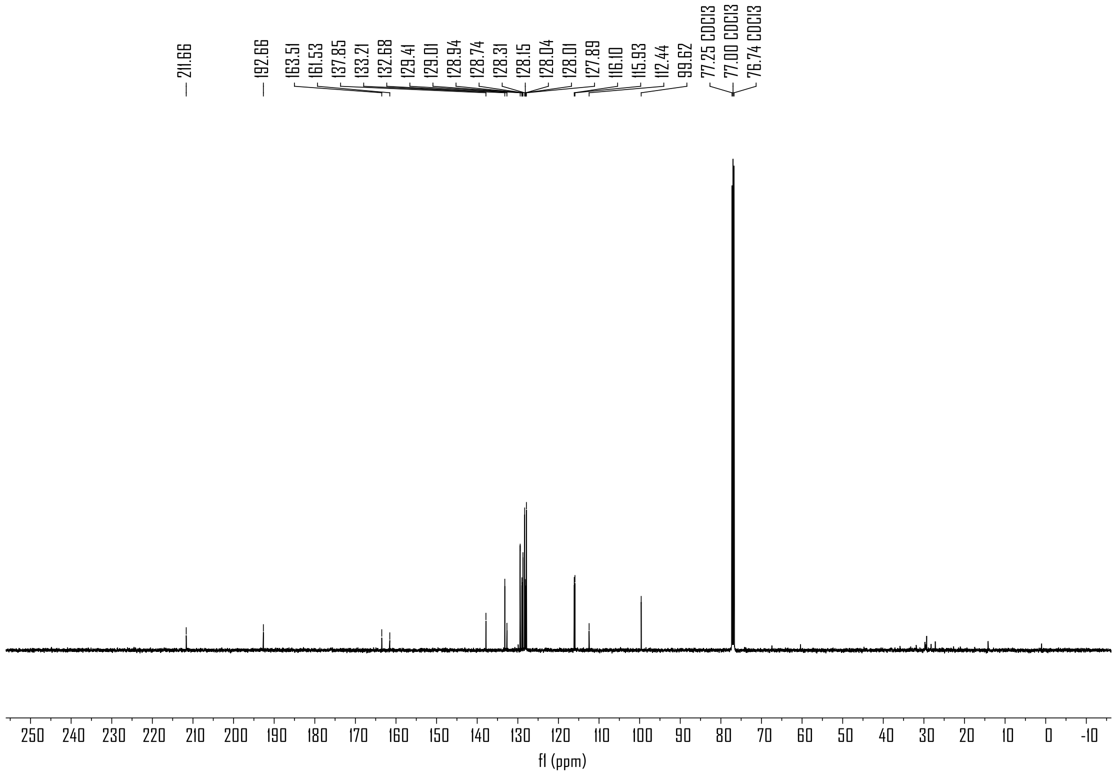
211.66
192.66
163.51
161.53
137.85
133.21
132.68
129.41
129.01
128.94
128.74
128.31
128.15
128.04
128.01
127.89
116.10
115.93
112.44
99.62
77.25 CDCl3
77.00 CDCl3
76.74 CDCl3
f1 (ppm)

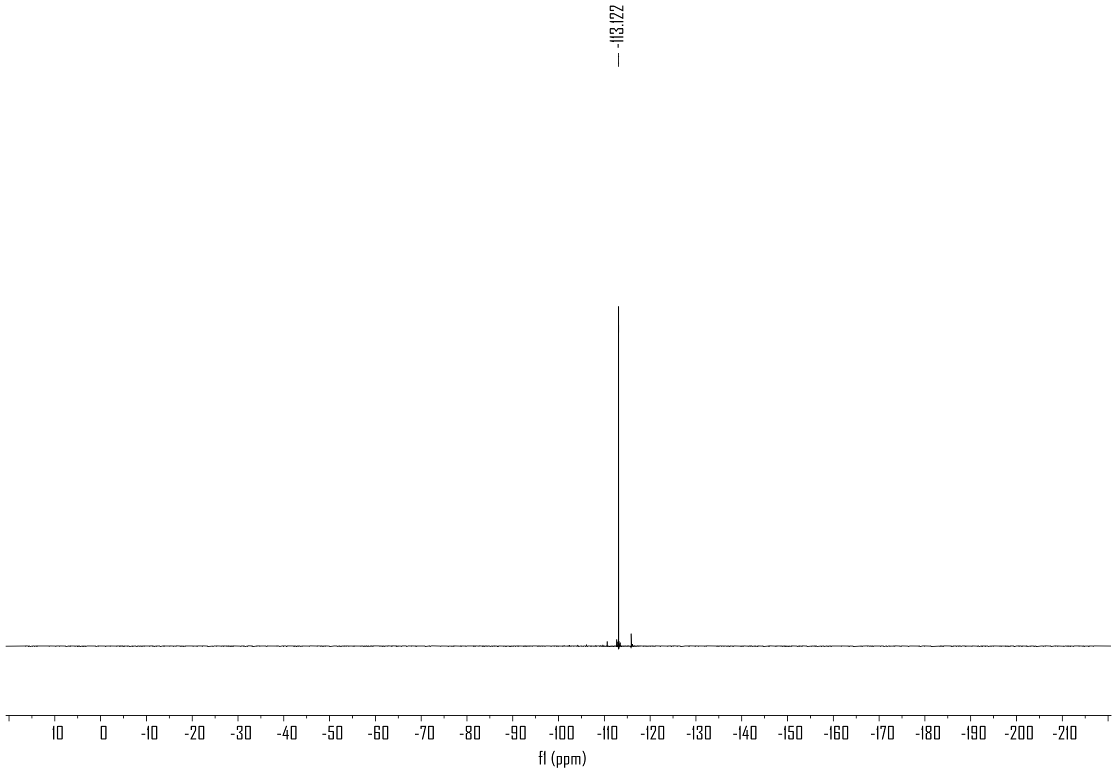
-113.122
f1 (ppm)

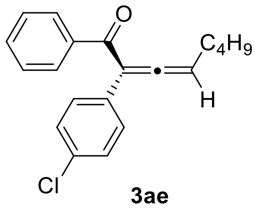
O
C4H9
H
Cl

3ae

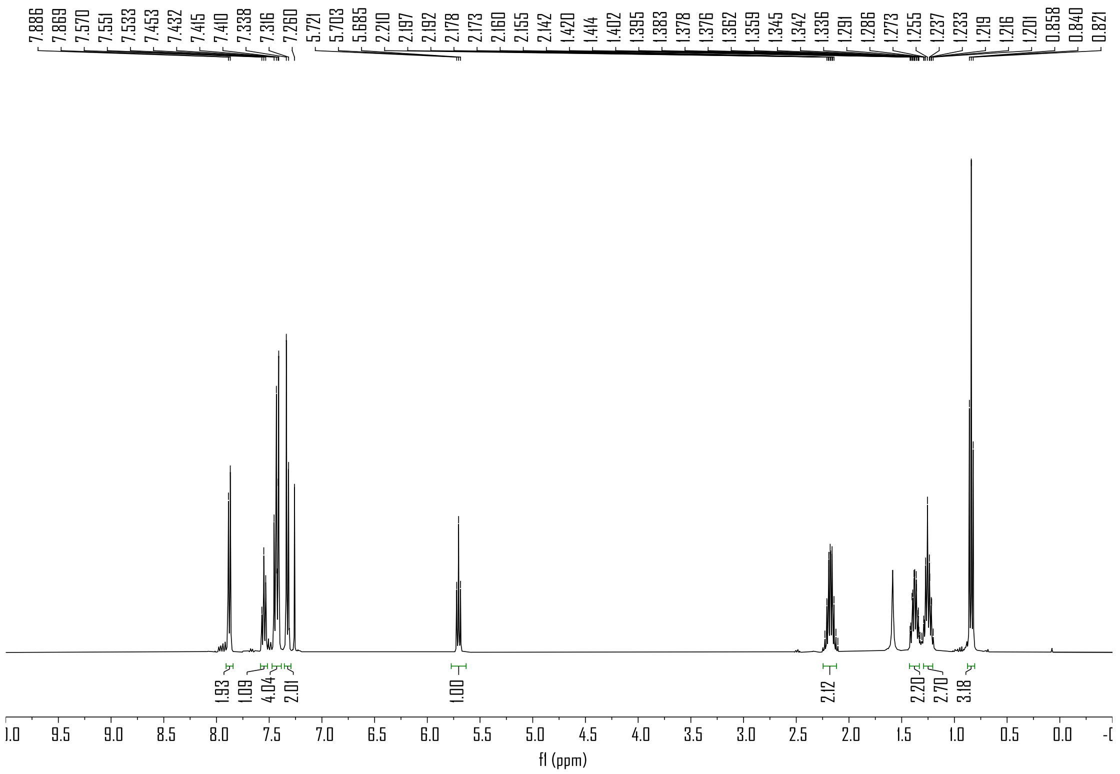
7.886
7.869
7.570
7.551
7.533
7.453
7.432
7.415
7.410
7.338
7.316
7.260
5.721
5.703
5.685
2.210
2.197
2.192
2.178
2.173
2.160
2.155
2.142
1.420
1.414
1.402
1.395
1.383
1.378
1.376
1.362
1.359
1.345
1.342
1.336
1.291
1.286
1.273
1.255
1.237
1.233
1.219
1.216
1.201
0.858
0.840
0.821
1.93
1.09
4.04
2.01
1.00
2.12
2.20
2.70
3.18
f1 (ppm)

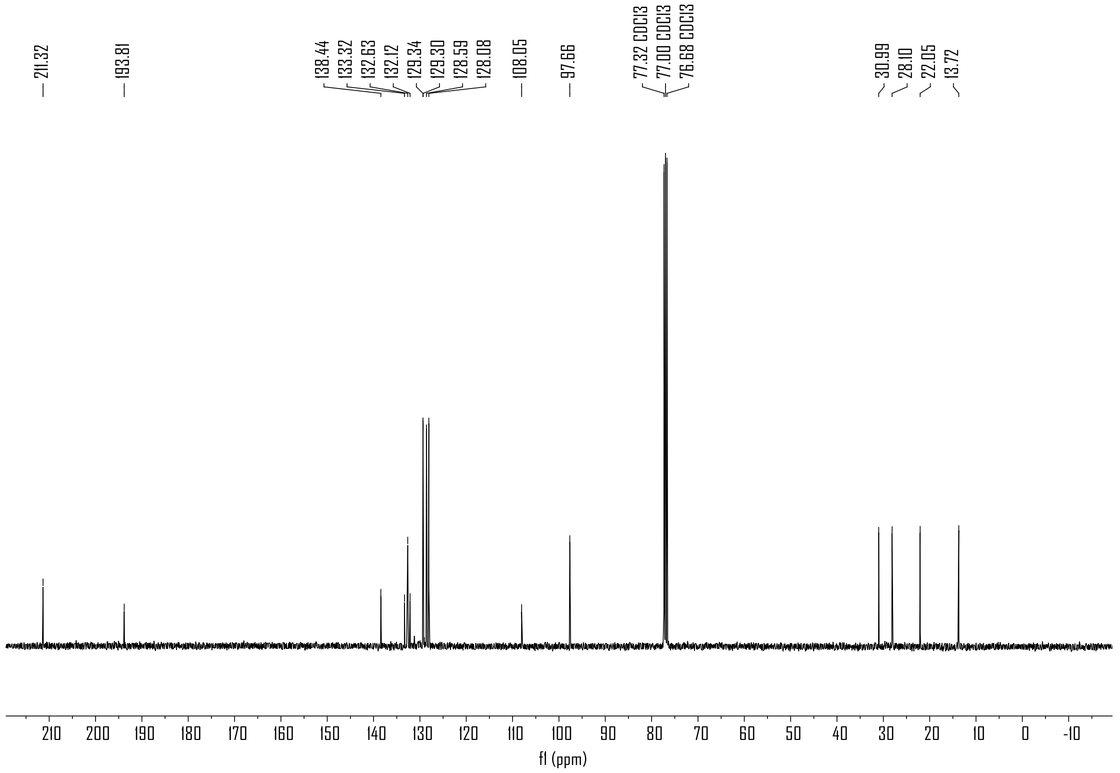
211.32
193.81
138.44
133.32
132.63
132.12
129.34
129.30
128.59
128.08
108.05
97.66
77.32 CDCl3
77.00 CDCl3
76.68 CDCl3
30.99
28.10
22.05
13.72
f1 (ppm)

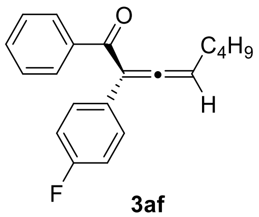
O
C4H9
H
F

3af

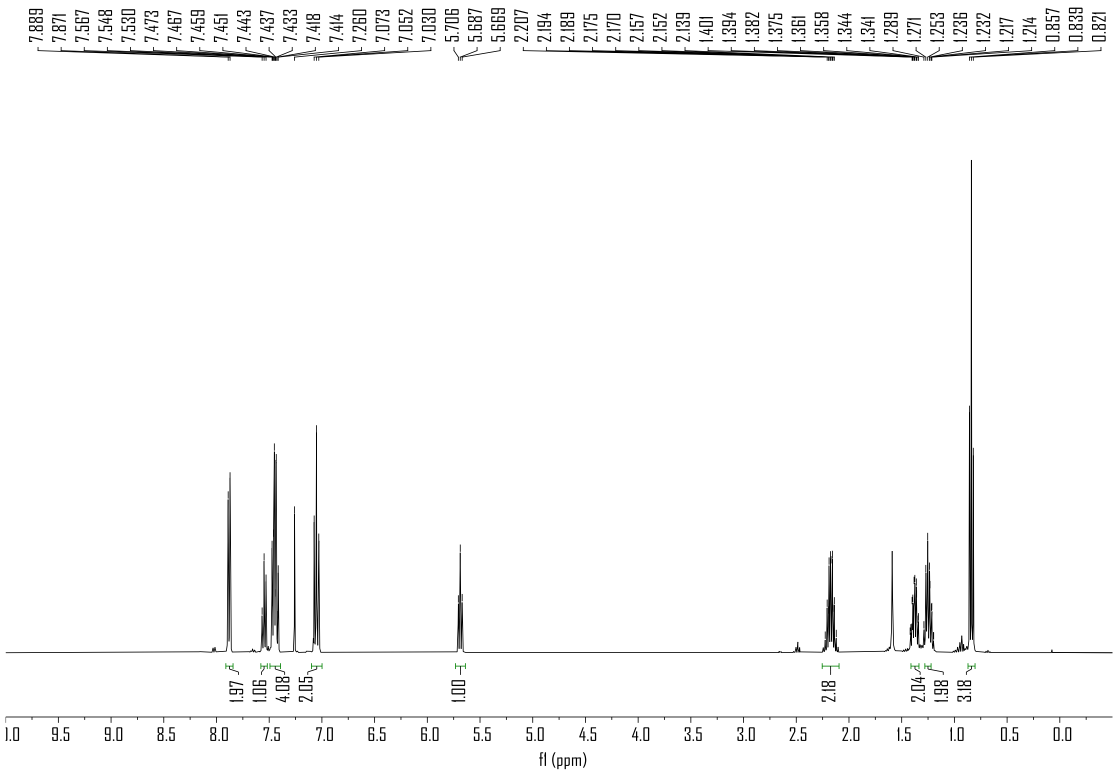
7.889
7.871
7.567
7.548
7.530
7.473
7.467
7.459
7.451
7.443
7.437
7.433
7.418
7.414
7.260
7.073
7.052
7.030
5.706
5.687
5.669
2.207
2.194
2.189
2.175
2.170
2.157
2.152
2.139
1.401
1.394
1.382
1.375
1.361
1.358
1.344
1.341
1.289
1.271
1.253
1.236
1.232
1.217
1.214
0.857
0.839
0.821
1.97
1.06
4.08
2.05
1.00
2.18
2.04
1.98
3.18
1.0 9.5 9.0 8.5 8.0 7.5 7.0 6.5 6.0 5.5 5.0 4.5 4.0 3.5 3.0 2.5 2.0 1.5 1.0 0.5 0.0
f1 (ppm)

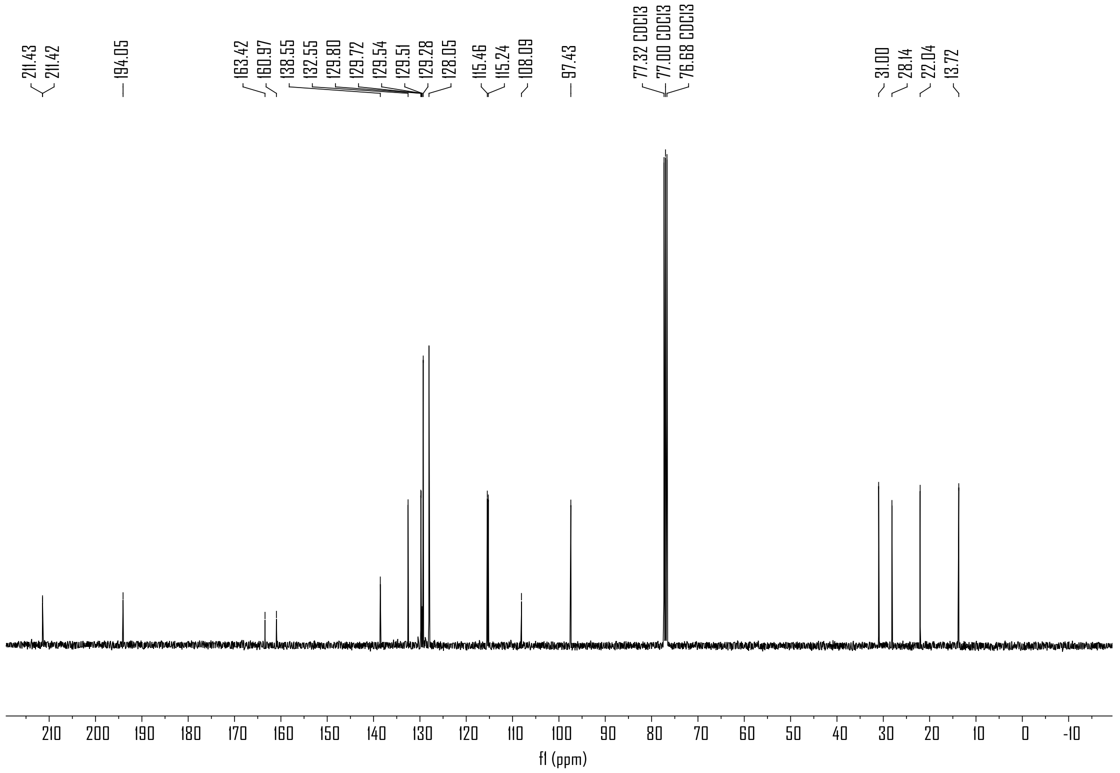
211.43
211.42
194.05
163.42
160.97
138.55
132.55
129.80
129.72
129.54
129.51
129.28
128.05
115.46
115.24
108.09
97.43
77.32 CDCl3
77.00 CDCl3
76.68 CDCl3
31.00
28.14
22.04
13.72
210 200 190 180 170 160 150 140 130 120 110 100 90 80 70 60 50 40 30 20 10 0 -10
f1 (ppm)

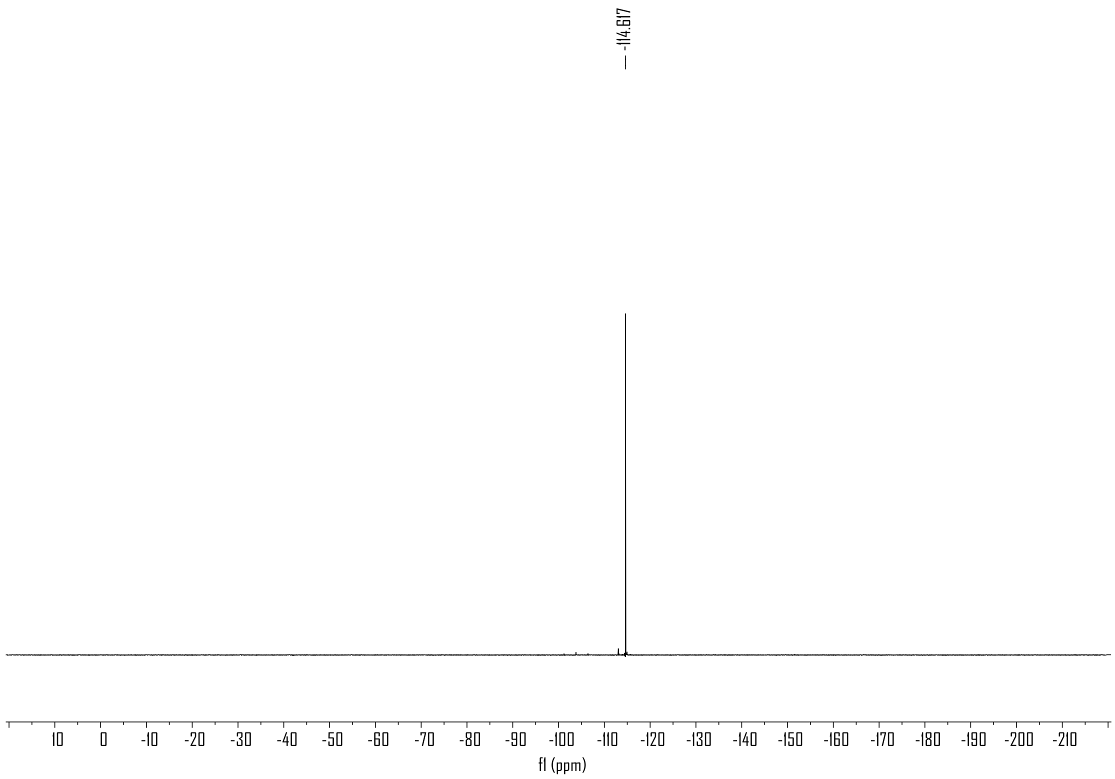
-114.617
10
0
-10
-20
-30
-40
-50
-60
-70
-80
-90
-100
-110
-120
-130
-140
-150
-160
-170
-180
-190
-200
-210
f1 (ppm)

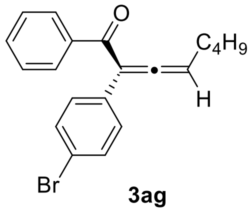
O
C_4H_9
H
Br

3ag

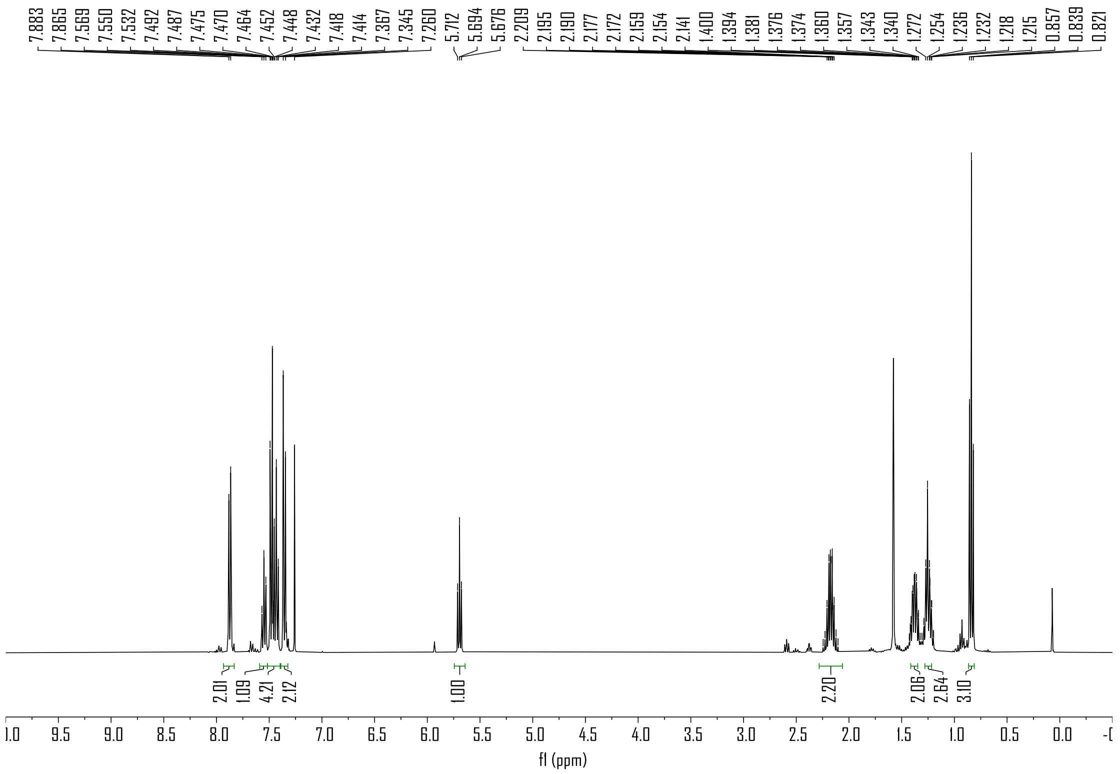
7.883 7.865 7.569 7.550 7.532 7.492 7.487 7.475 7.470 7.464 7.452 7.448 7.432 7.418 7.414 7.367 7.345 7.260
5.712 5.694 5.676
2.209 2.195 2.190 2.177 2.172 2.159 2.154 2.141 1.400 1.394 1.381 1.376 1.374 1.360 1.357 1.343 1.340 1.272 1.254 1.236 1.232 1.218 1.215 0.857 0.839 0.821
2.01 1.09 4.21 2.12
1.00
2.20
2.06 2.64 3.10
9.5 9.0 8.5 8.0 7.5 7.0 6.5 6.0 5.5 5.0 4.5 4.0 3.5 3.0 2.5 2.0 1.5 1.0 0.5 0.0
f1 (ppm)

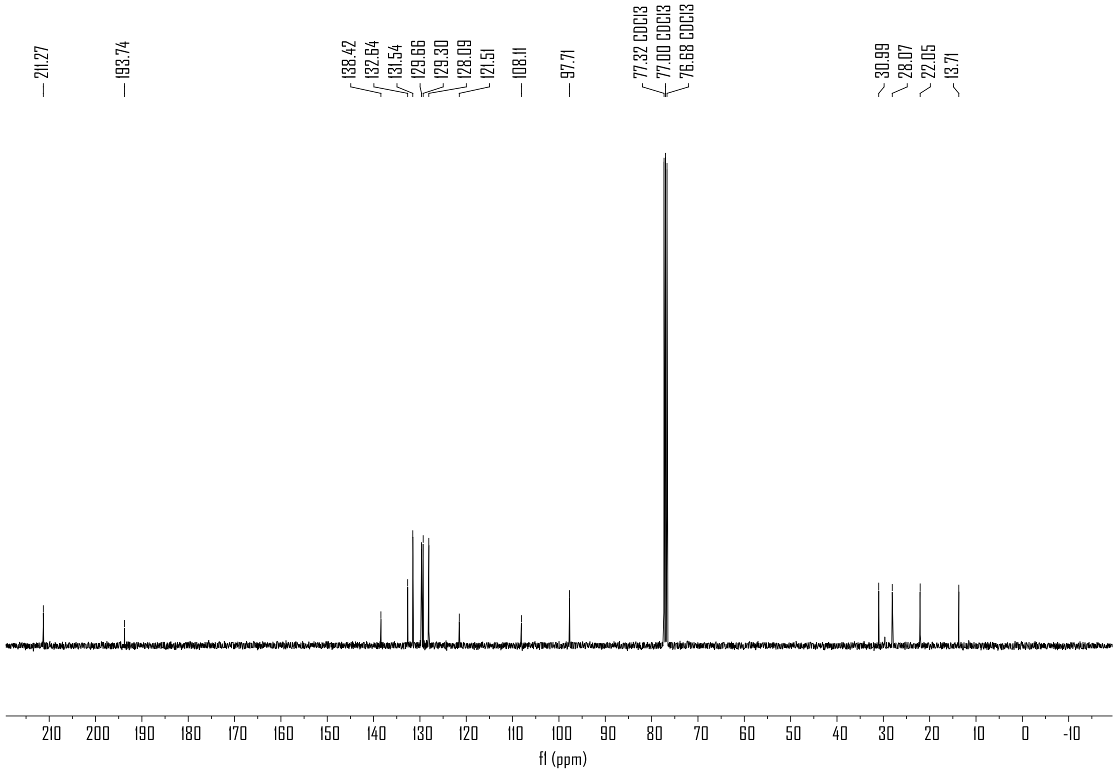
211.27
193.74
138.42 132.64 131.54 129.66 129.30 128.09 121.51
108.11
97.71
77.32 CDCl3 77.00 CDCl3 76.68 CDCl3
30.99 28.07 22.05 13.71
210 200 190 180 170 160 150 140 130 120 110 100 90 80 70 60 50 40 30 20 10 0 -10
f1 (ppm)

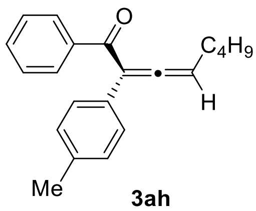
O
C4H9
H
Me

3ah

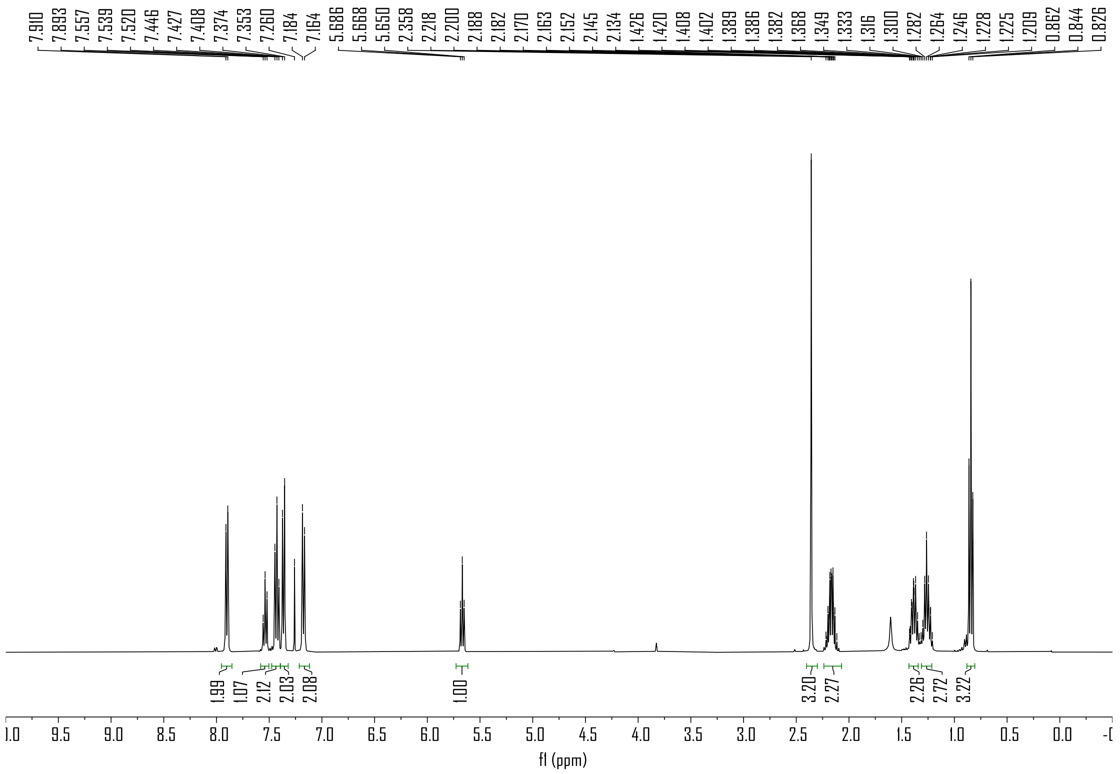
7.910 7.893 7.557 7.539 7.520 7.446 7.427 7.408 7.374 7.353 7.260 7.184 7.164 5.686 5.668 5.650 2.358 2.218 2.200 2.188 2.182 2.170 2.163 2.152 2.145 2.134 1.426 1.420 1.408 1.402 1.389 1.386 1.382 1.368 1.349 1.333 1.316 1.300 1.282 1.264 1.246 1.228 1.225 1.209 0.862 0.844 0.826
1.99 1.07 2.12 2.03 2.08 1.00 3.20 2.27 2.26 2.72 3.22
f1 (ppm)

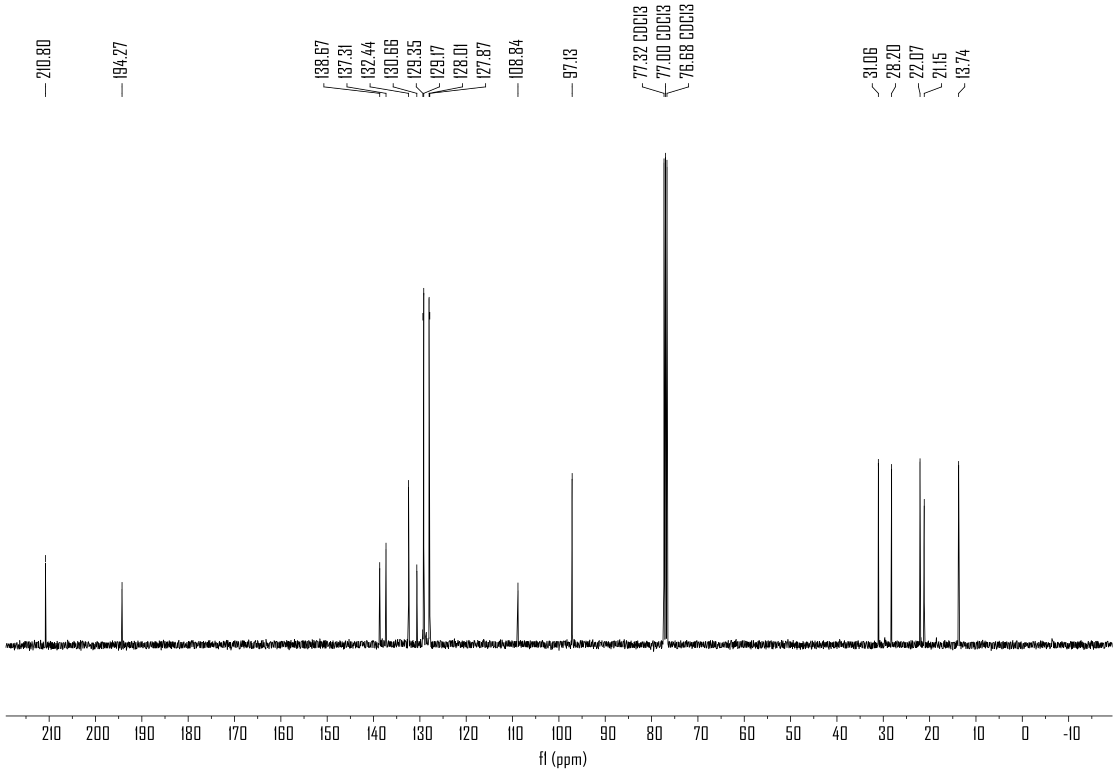
210.80 194.27 138.67 137.31 132.44 130.66 129.35 129.17 128.01 127.87 108.84 97.13 77.32 CDCl3 77.00 CDCl3 76.68 CDCl3 31.06 28.20 22.07 21.15 13.74
f1 (ppm)

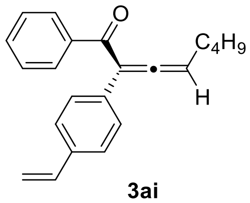
O
C4H9
H

3ai

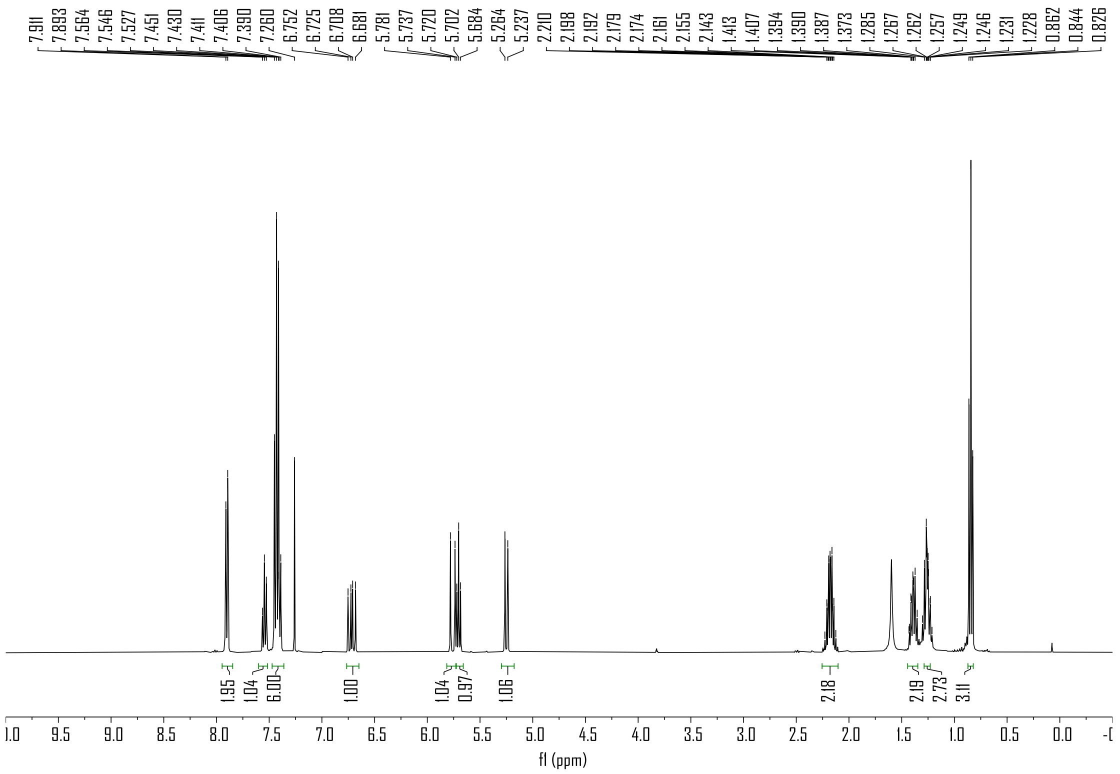
7.911
7.893
7.564
7.546
7.527
7.451
7.430
7.411
7.406
7.390
7.260
6.752
6.725
6.708
6.681
5.781
5.737
5.720
5.702
5.684
5.264
5.237
2.210
2.198
2.192
2.179
2.174
2.161
2.155
2.143
1.413
1.407
1.394
1.390
1.387
1.373
1.285
1.267
1.262
1.257
1.249
1.246
1.231
1.228
0.862
0.844
0.826
1.95
1.04
6.00
1.00
1.04
0.97
1.06
2.18
2.19
2.73
3.11
f1 (ppm)

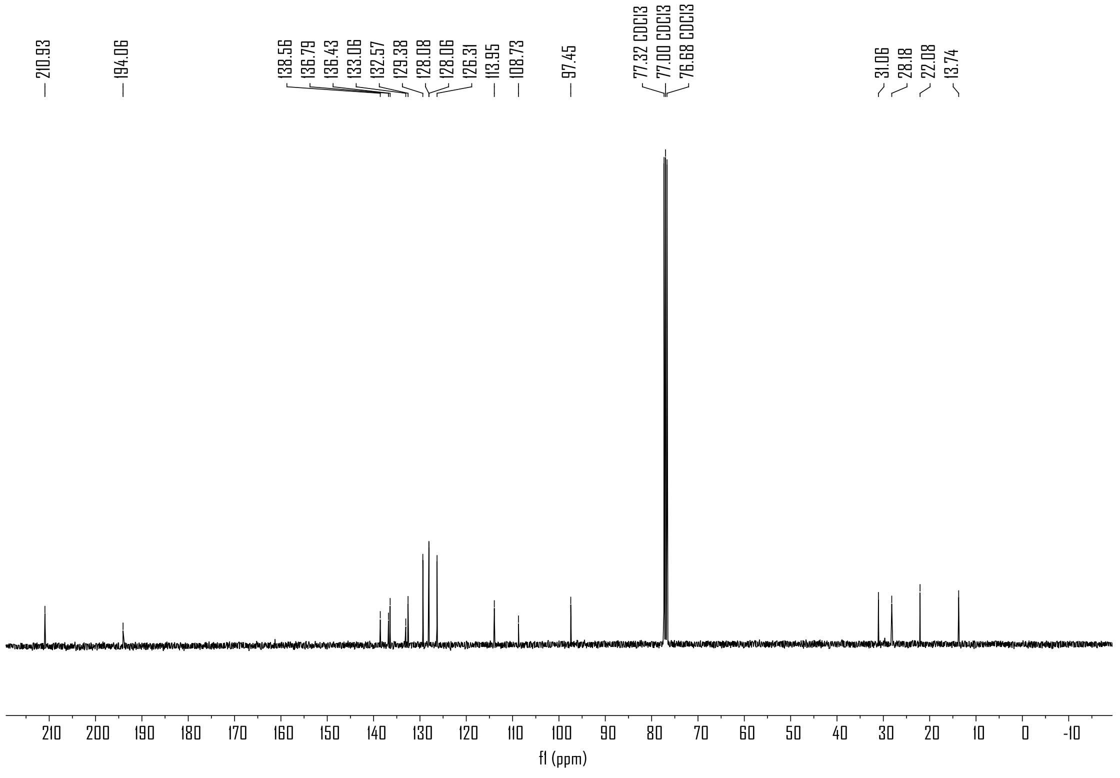
210.93
194.06
138.56
136.79
136.43
133.06
132.57
129.38
128.08
128.06
126.31
113.95
108.73
97.45
77.32 CDCl3
77.00 CDCl3
76.68 CDCl3
31.06
28.18
22.08
13.74
f1 (ppm)

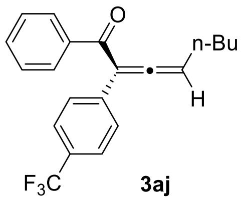
O
n-Bu
H
F3C

3aj

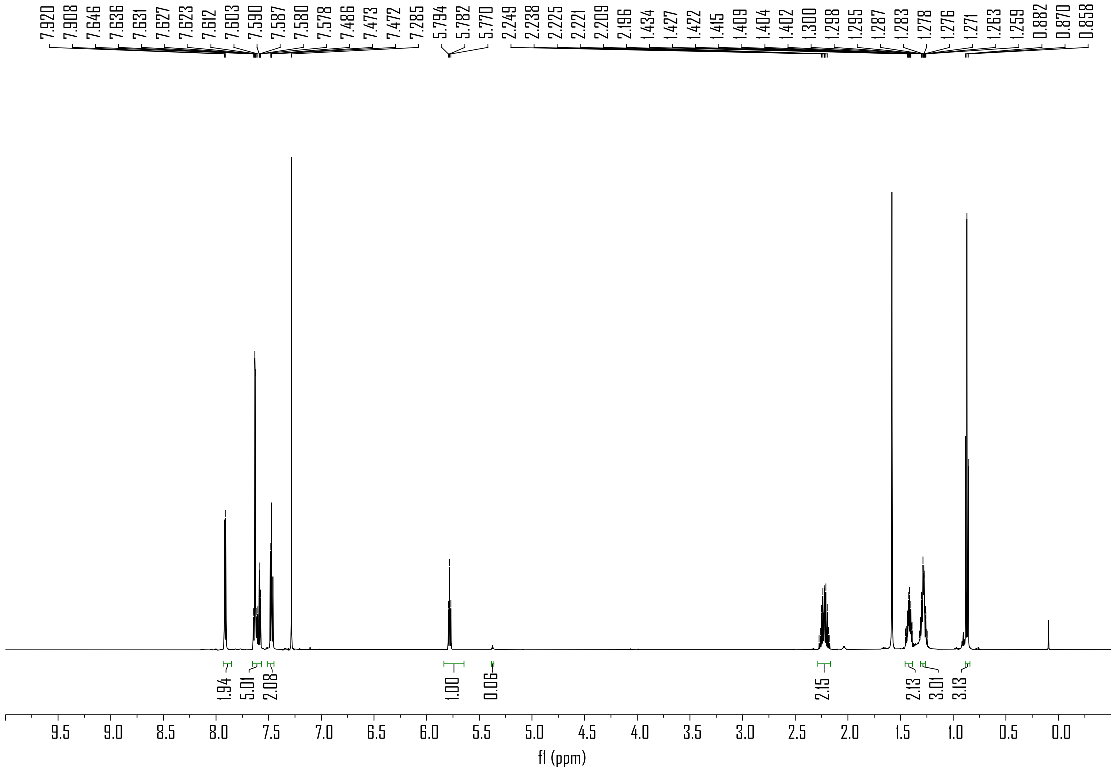
7.920
7.908
7.646
7.636
7.631
7.627
7.623
7.612
7.603
7.590
7.587
7.580
7.578
7.486
7.473
7.472
7.285
5.794
5.782
5.770
2.249
2.238
2.225
2.221
2.209
2.196
1.434
1.427
1.422
1.415
1.409
1.404
1.402
1.300
1.298
1.295
1.287
1.283
1.278
1.276
1.271
1.263
1.259
0.882
0.870
0.858
1.94
5.01
2.08
1.00
0.06
2.15
2.13
3.01
3.13
9.5
9.0
8.5
8.0
7.5
7.0
6.5
6.0
5.5
5.0
4.5
4.0
3.5
3.0
2.5
2.0
1.5
1.0
0.5
0.0
f1 (ppm)

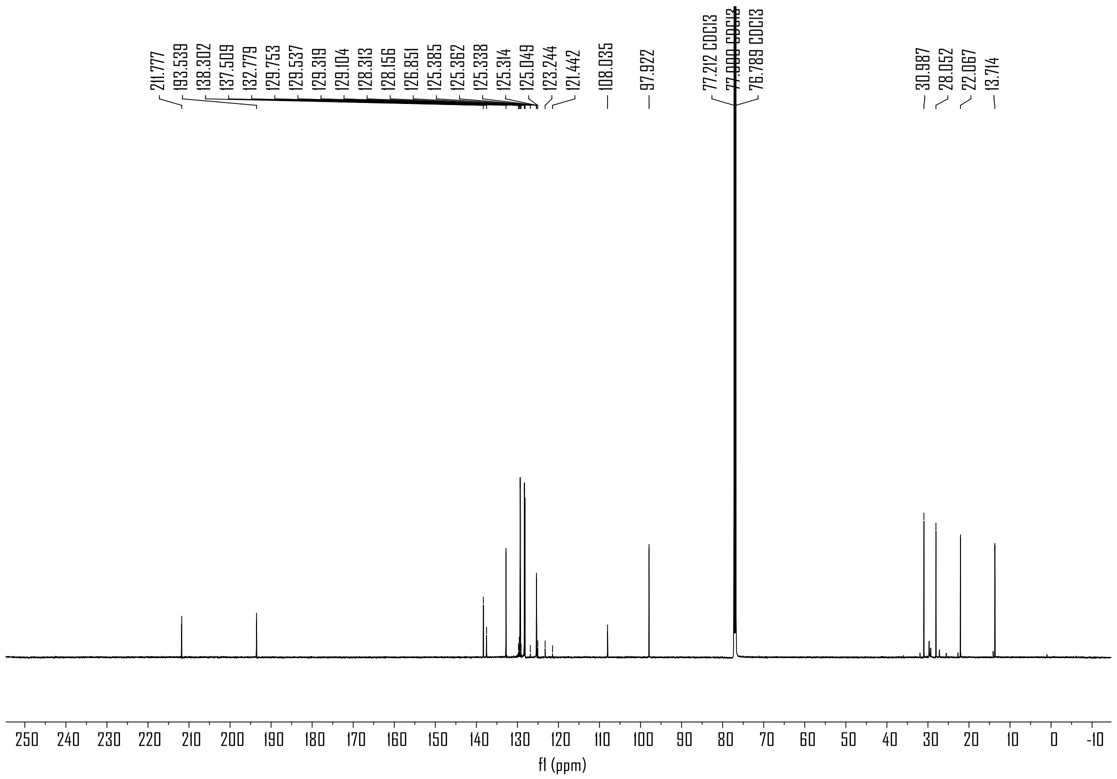
211.777
193.539
138.302
137.509
132.779
129.753
129.537
129.319
129.104
128.313
128.156
126.851
125.385
125.362
125.338
125.314
125.049
123.244
121.442
108.035
97.922
77.212 CDCl3
77.000 CDCl3
76.789 CDCl3
30.987
28.052
22.067
13.714
250 240 230 220 210 200 190 180 170 160 150 140 130 120 110 100 90 80 70 60 50 40 30 20 10 0 -10
f1 (ppm)

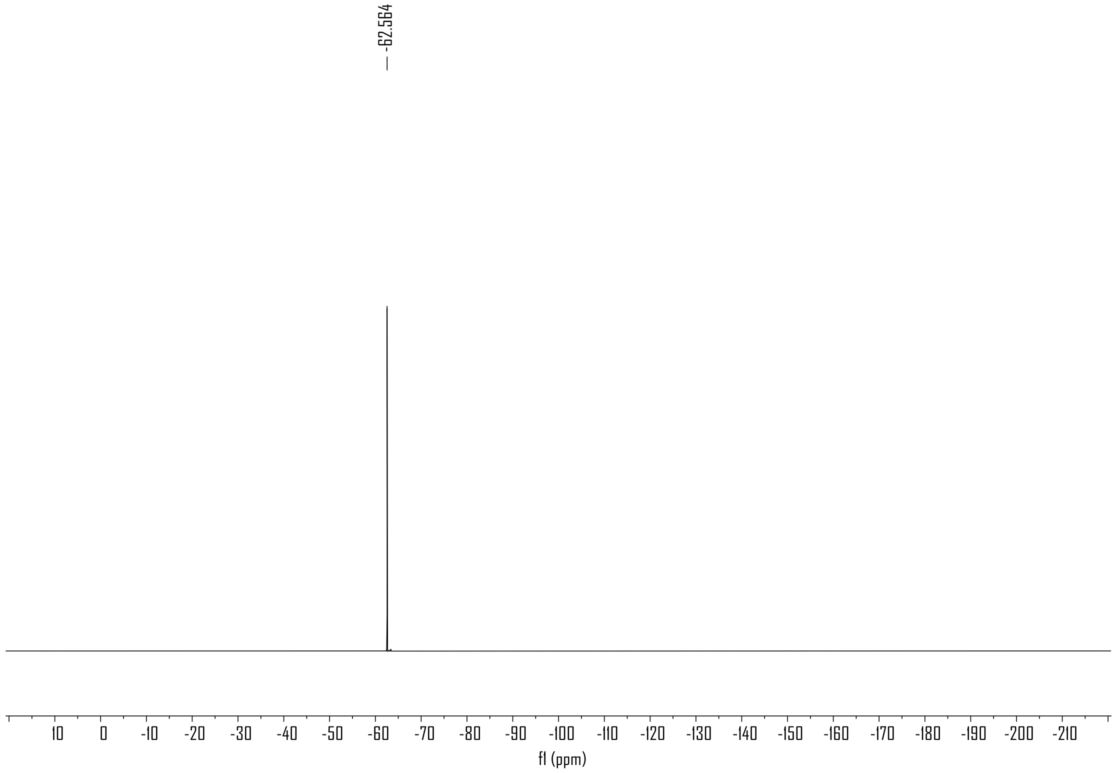
-62.564
10 0 -10 -20 -30 -40 -50 -60 -70 -80 -90 -100 -110 -120 -130 -140 -150 -160 -170 -180 -190 -200 -210
f1 (ppm)

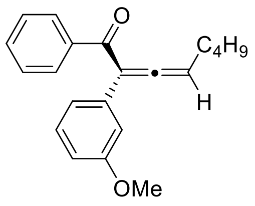
O
C4H9
H
OMe

3ak

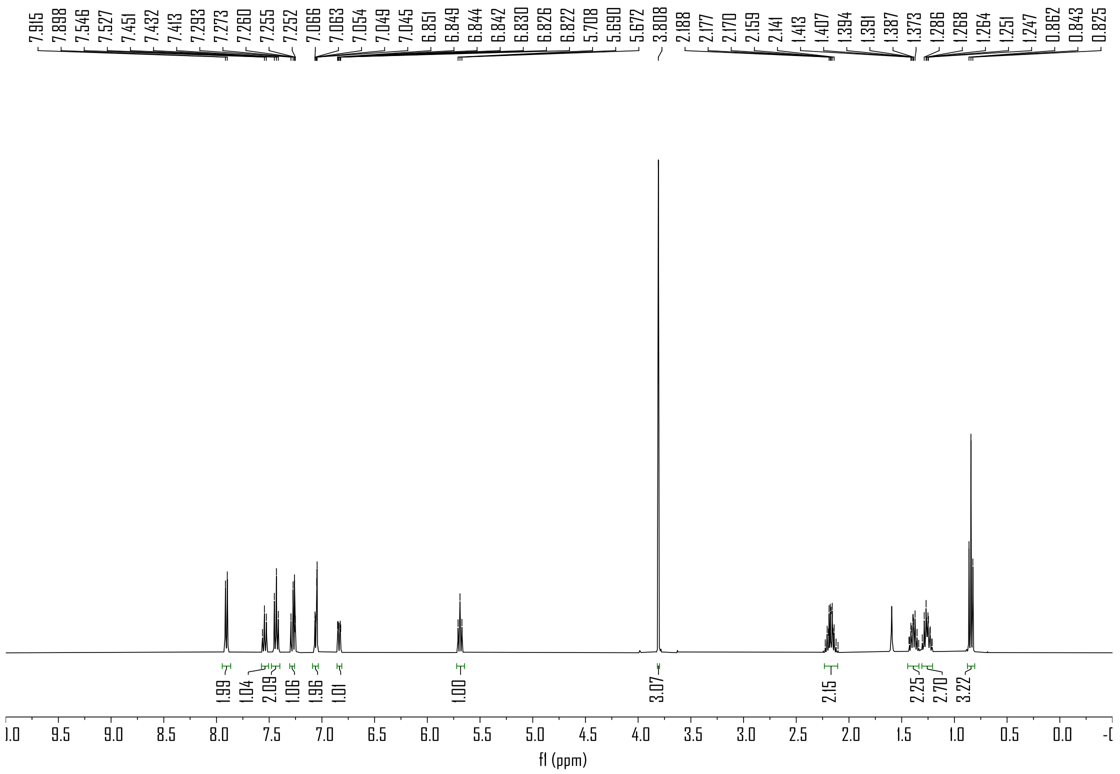
7.915
7.898
7.546
7.527
7.451
7.432
7.413
7.293
7.273
7.260
7.255
7.252
7.066
7.063
7.054
7.049
7.045
6.851
6.849
6.844
6.842
6.830
6.826
6.822
5.708
5.690
5.672
3.808
2.188
2.177
2.170
2.159
2.141
1.413
1.407
1.394
1.391
1.387
1.373
1.286
1.268
1.264
1.251
1.247
0.862
0.843
0.825
1.99
1.04
2.09
1.06
1.96
1.01
1.00
3.07
2.15
2.25
2.70
3.22
f1 (ppm)

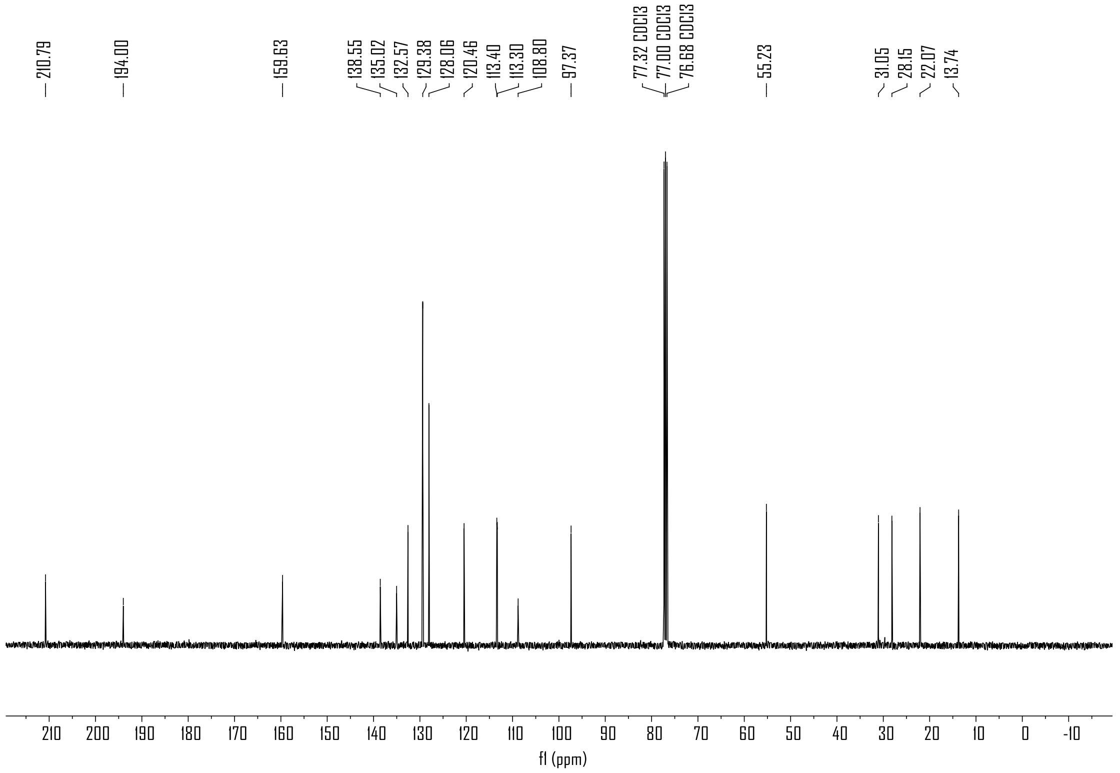
210.79
194.00
159.63
138.55
135.02
132.57
129.38
128.06
120.46
113.40
113.30
108.80
97.37
77.32 CDCl3
77.00 CDCl3
76.68 CDCl3
55.23
31.05
28.15
22.07
13.74
f1 (ppm)

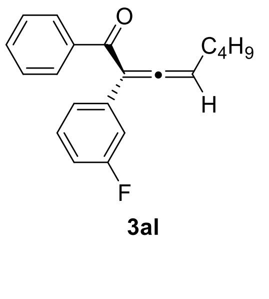
O
C4H9
H
F

3al

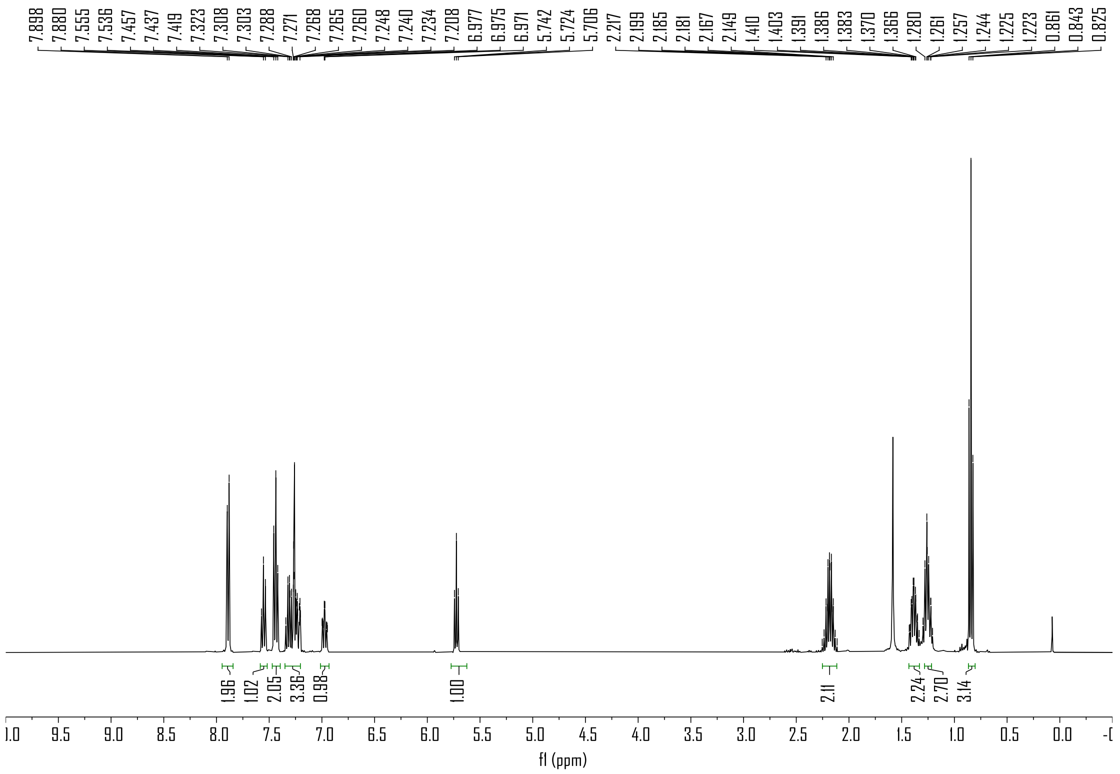

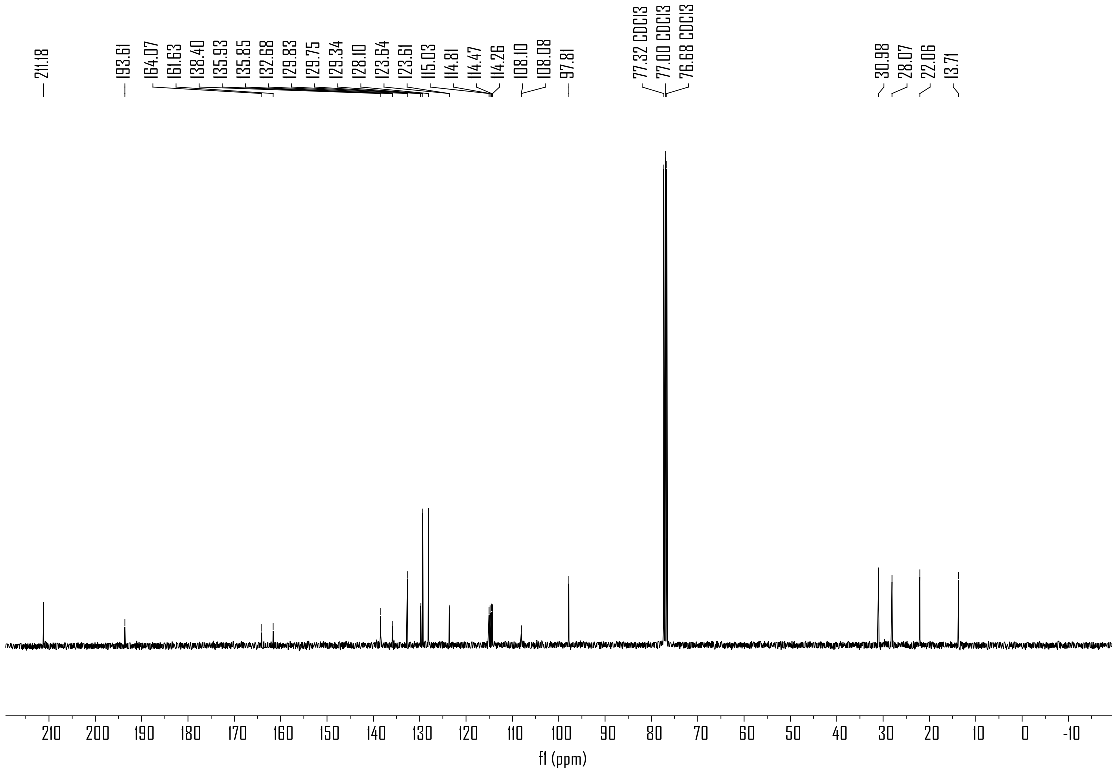

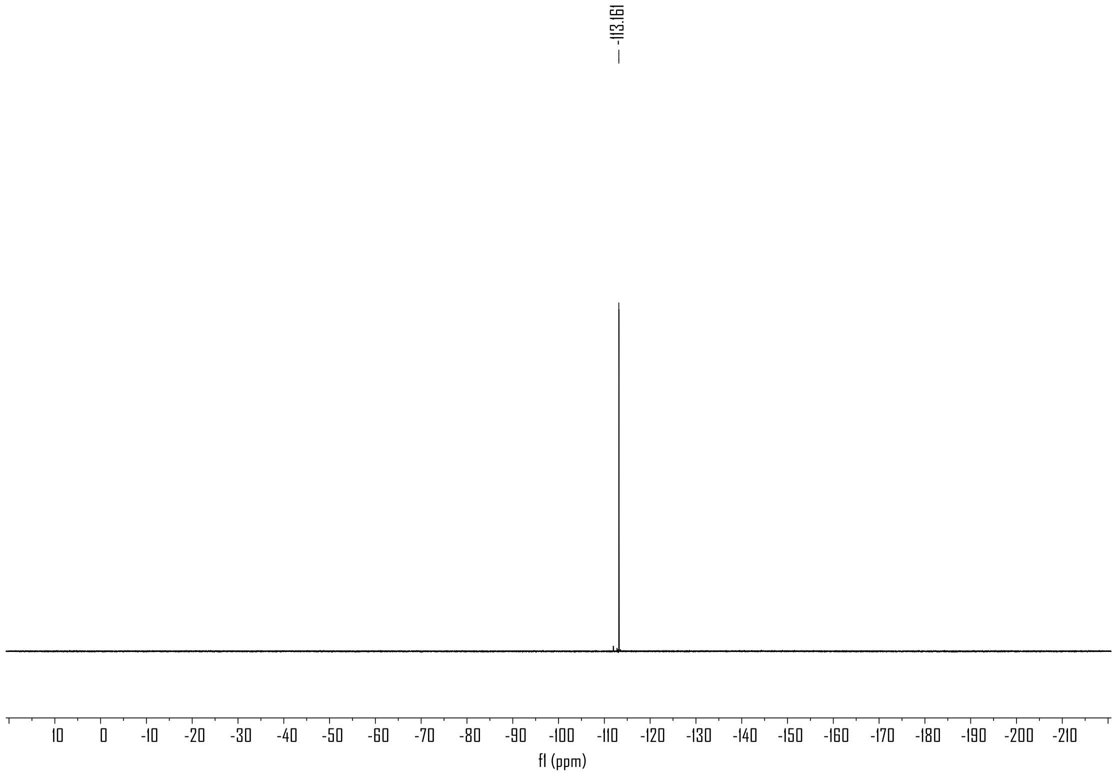
-113.161
10 0 -10 -20 -30 -40 -50 -60 -70 -80 -90 -100 -110 -120 -130 -140 -150 -160 -170 -180 -190 -200 -210
f1 (ppm)

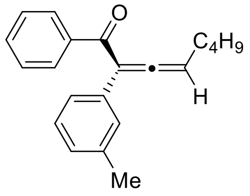
O
C4H9
H
Me

3am

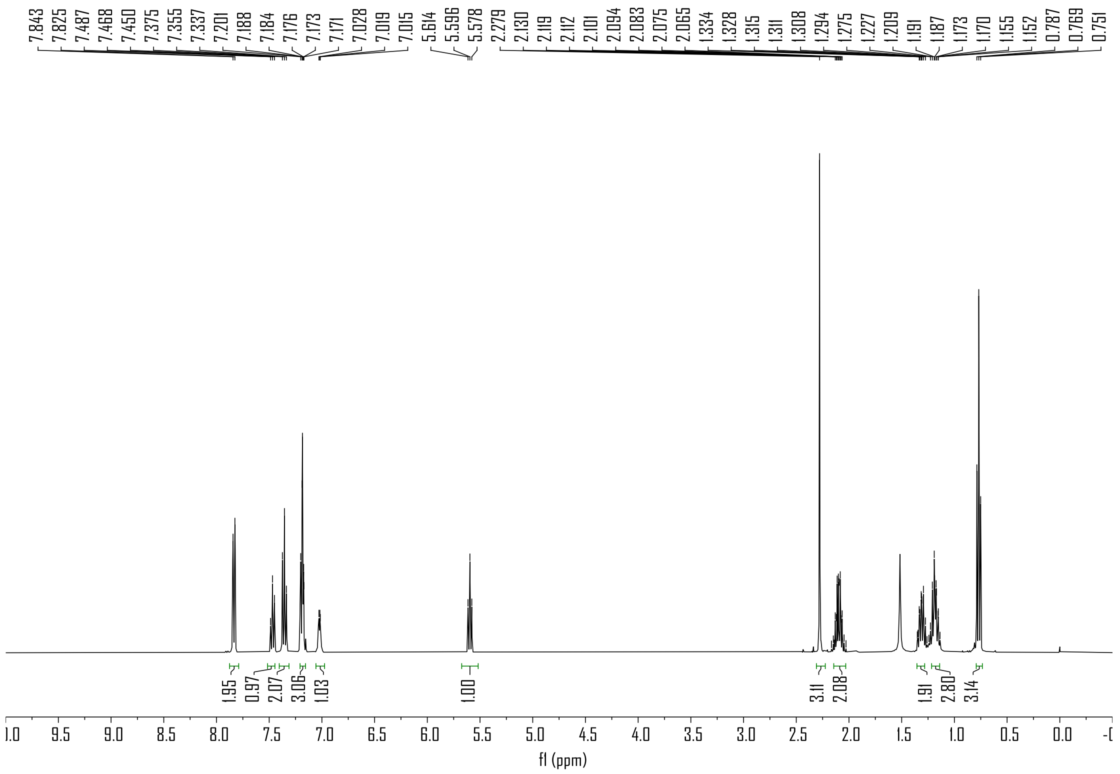
7.843
7.825
7.487
7.468
7.450
7.375
7.355
7.337
7.201
7.188
7.184
7.176
7.173
7.171
7.028
7.019
7.015
5.614
5.596
5.578
2.279
2.130
2.119
2.112
2.101
2.094
2.083
2.075
2.065
1.334
1.328
1.315
1.311
1.308
1.294
1.275
1.227
1.209
1.191
1.187
1.173
1.170
1.155
1.152
0.787
0.769
0.751
1.95
0.97
2.07
3.06
1.03
1.00
3.11
2.08
1.91
2.80
3.14
fl (ppm)

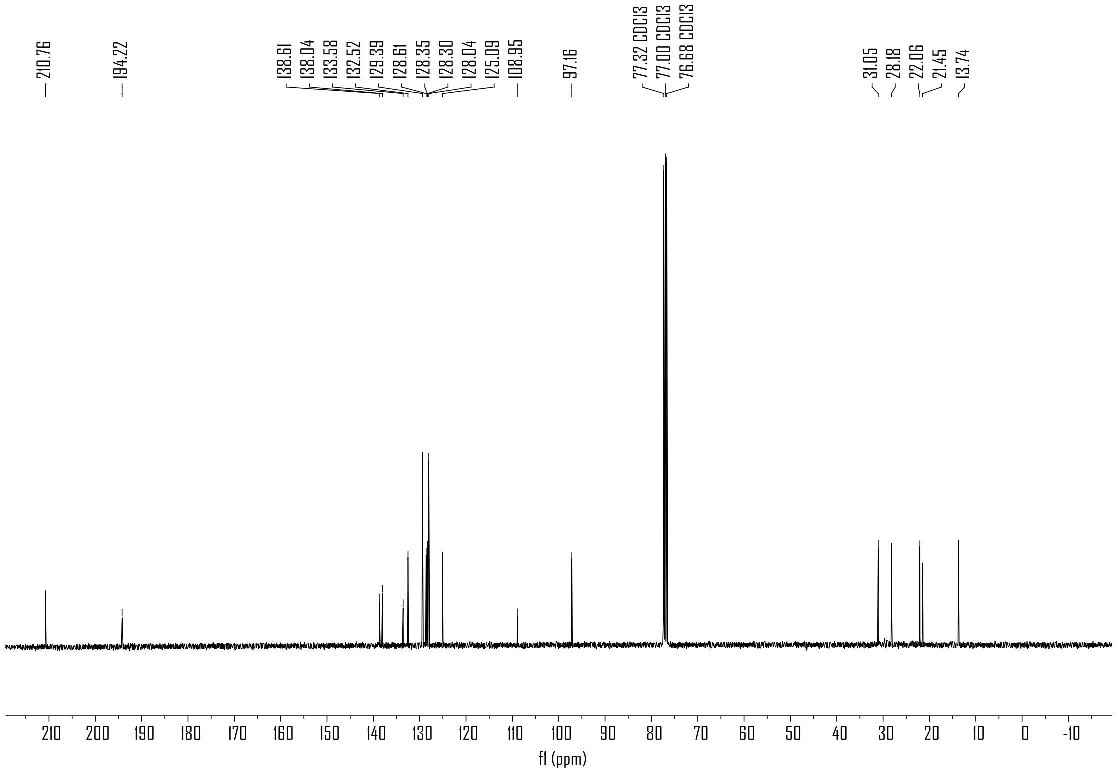
210.76
194.22
138.61
138.04
133.58
132.52
129.39
128.61
128.35
128.30
128.04
125.09
108.95
97.16
77.32 CDCl3
77.00 CDCl3
76.68 CDCl3
31.05
28.18
22.06
21.45
13.74
fl (ppm)

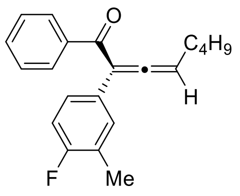
O
C4H9
H
F
Me

3an

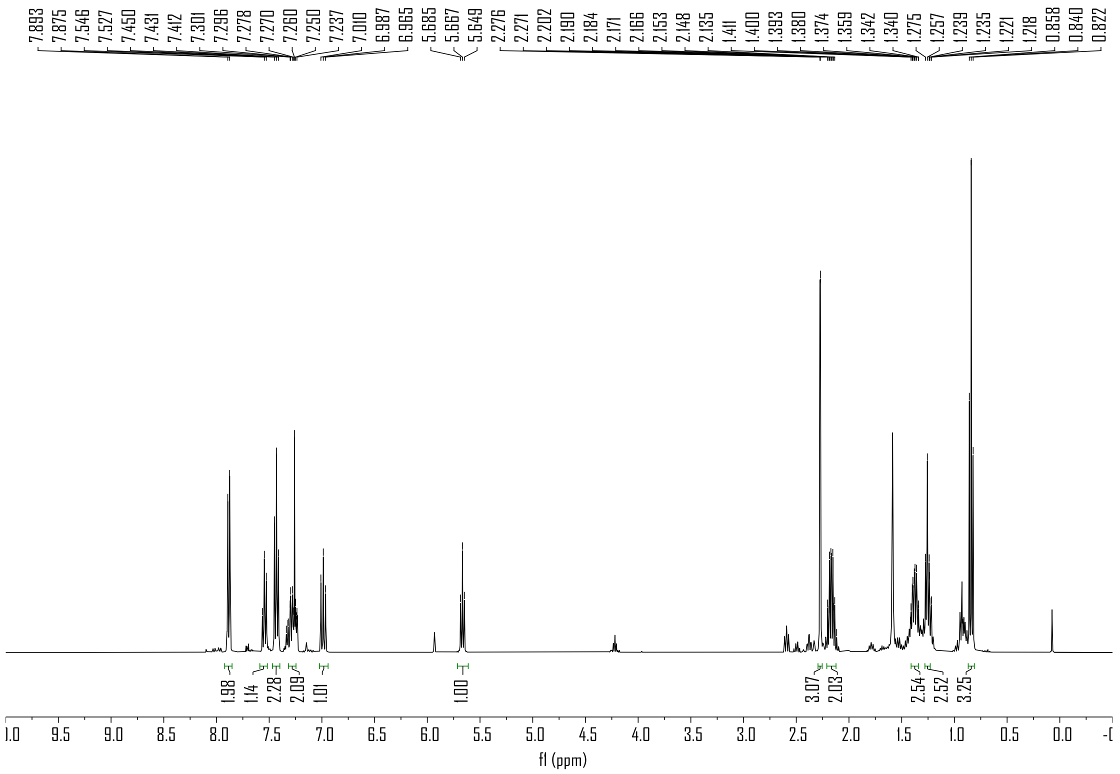
7.893
7.875
7.546
7.527
7.450
7.431
7.412
7.301
7.296
7.278
7.270
7.260
7.250
7.237
7.010
6.987
6.965
5.685
5.667
5.649
2.276
2.271
2.202
2.190
2.184
2.171
2.166
2.153
2.148
2.135
1.411
1.400
1.393
1.380
1.374
1.359
1.342
1.340
1.275
1.257
1.239
1.235
1.221
1.218
0.858
0.840
0.822
1.98
1.14
2.28
2.09
1.01
1.00
3.07
2.03
2.54
2.52
3.25
f1 (ppm)

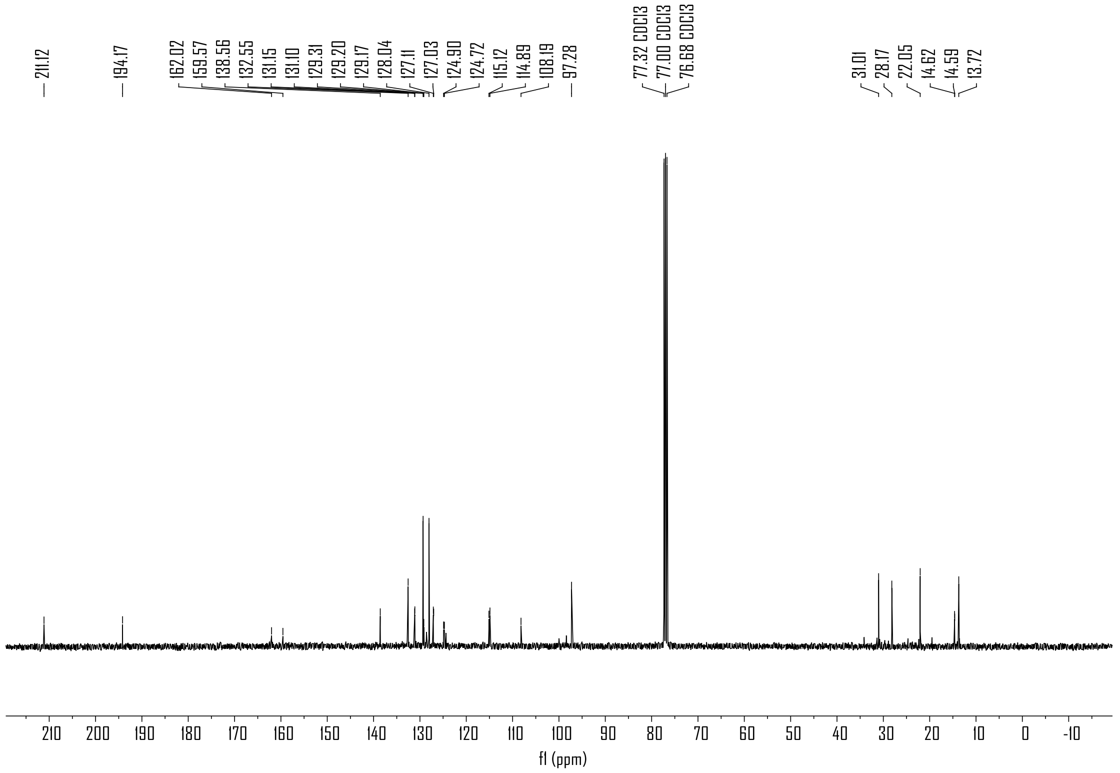
211.12
194.17
162.02
159.57
138.56
132.55
131.15
131.10
129.31
129.20
129.17
128.04
127.11
127.03
124.90
124.72
115.12
114.89
108.19
97.28
77.32 CDCl3
77.00 CDCl3
76.68 CDCl3
31.01
28.17
22.05
14.62
14.59
13.72
f1 (ppm)

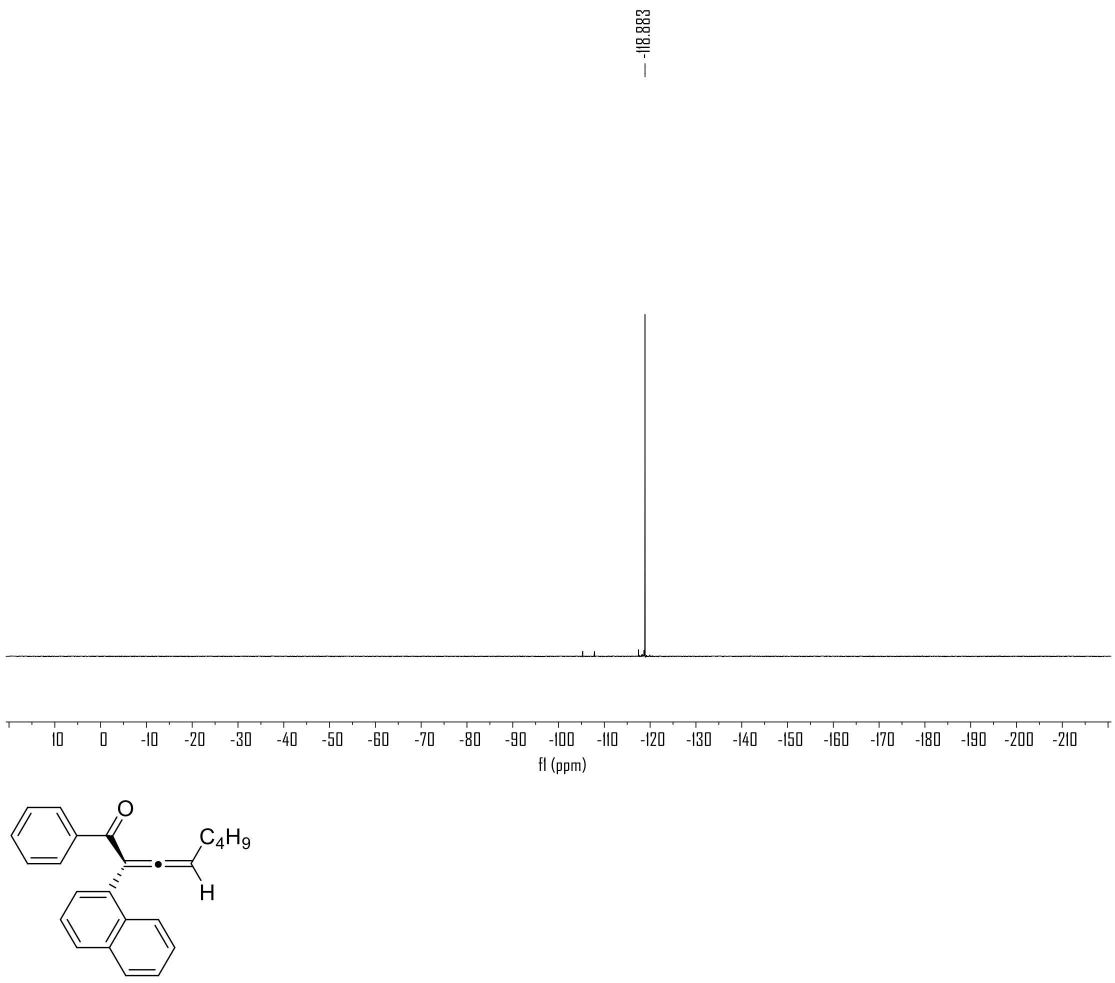
-118.883
10
0
-10
-20
-30
-40
-50
-60
-70
-80
-90
-100
-110
-120
-130
-140
-150
-160
-170
-180
-190
-200
-210
f1 (ppm)
O
C_4H_9
H

3ao

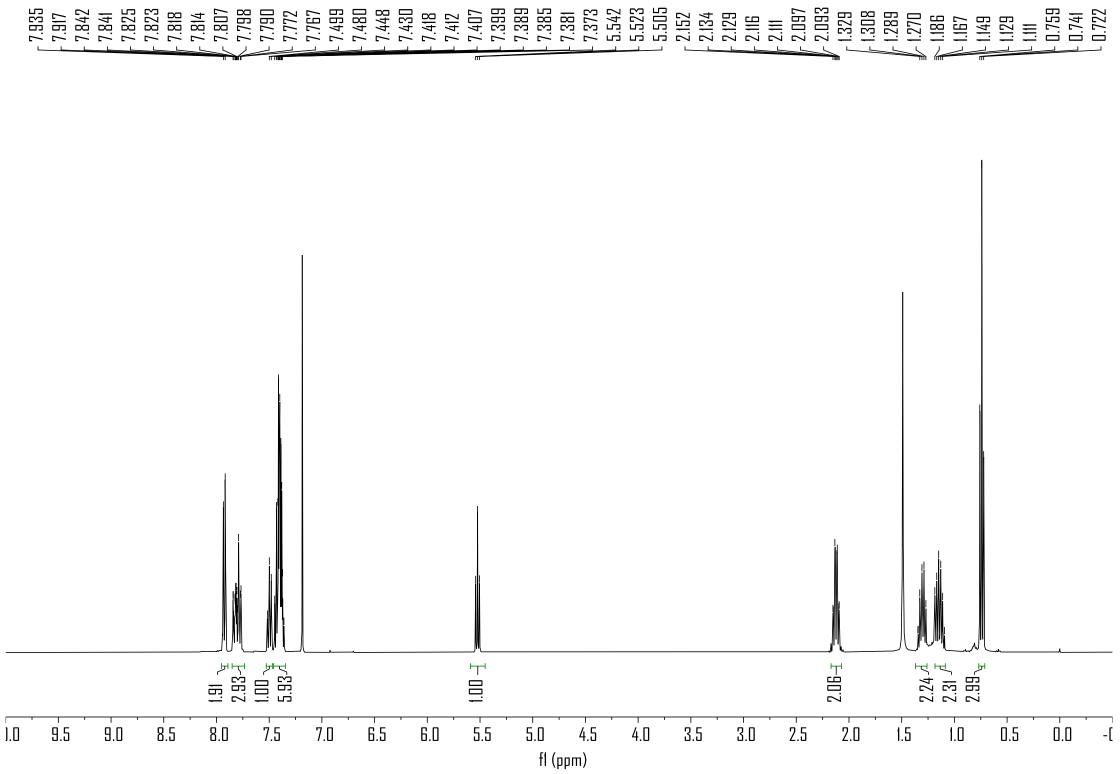
7.935
7.917
7.842
7.841
7.825
7.823
7.818
7.814
7.807
7.798
7.790
7.772
7.767
7.499
7.480
7.448
7.430
7.418
7.412
7.407
7.399
7.389
7.385
7.381
7.373
5.542
5.523
5.505
2.152
2.134
2.129
2.116
2.111
2.097
2.093
1.329
1.308
1.289
1.270
1.186
1.167
1.149
1.129
1.111
0.759
0.741
0.722
1.91
2.93
1.00
5.93
1.00
2.06
2.24
2.31
2.99
fl (ppm)

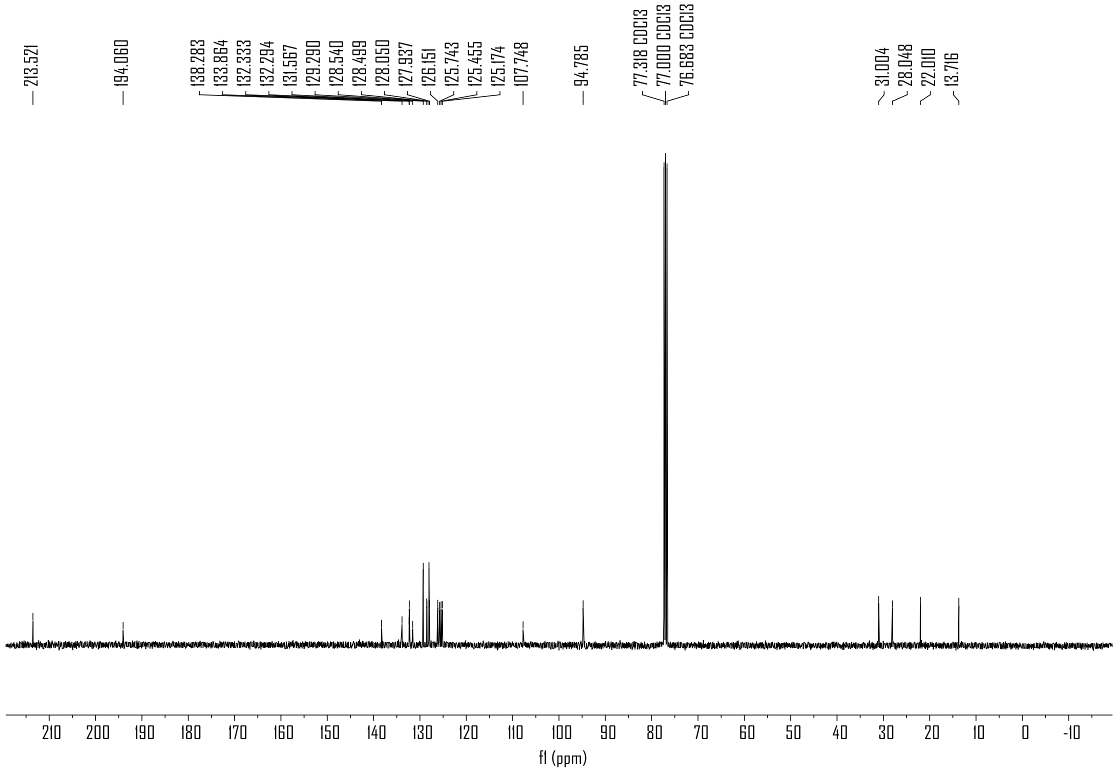
213.521
194.060
138.283
133.864
132.333
132.294
131.567
129.290
128.540
128.499
128.050
127.937
126.151
125.743
125.455
125.174
107.748
94.785
77.318 CDCl3
77.000 CDCl3
76.683 CDCl3
31.004
28.048
22.010
13.716
fl (ppm)

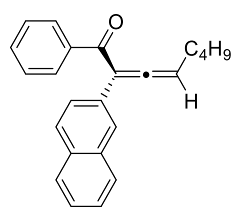
O
C4H9
H

3ap

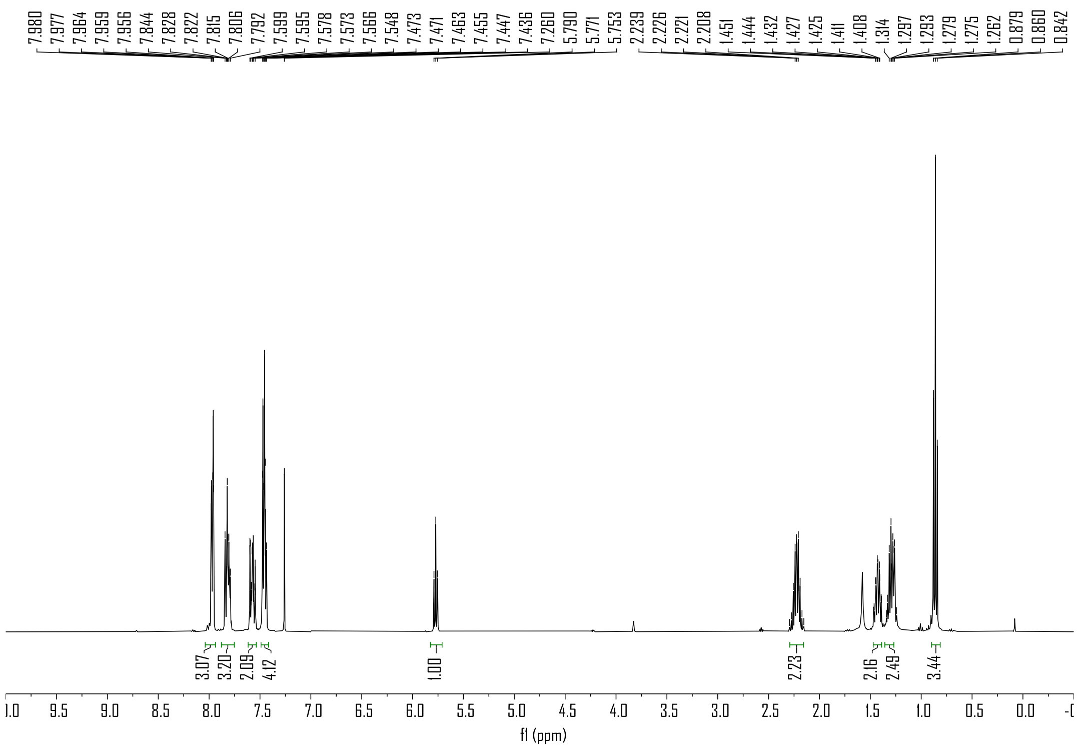
7.980 7.977 7.964 7.959 7.956 7.844 7.828 7.822 7.815 7.806 7.792 7.599 7.595 7.578 7.573 7.566 7.548 7.473 7.471 7.463 7.455 7.447 7.436 7.260 5.790 5.771 5.753 2.239 2.226 2.221 2.208 1.451 1.444 1.432 1.427 1.425 1.411 1.408 1.314 1.297 1.293 1.279 1.275 1.262 0.879 0.860 0.842
3.07
3.20
2.09
4.12
1.00
2.23
2.16
2.49
3.44
9.5 9.0 8.5 8.0 7.5 7.0 6.5 6.0 5.5 5.0 4.5 4.0 3.5 3.0 2.5 2.0 1.5 1.0 0.5 0.0
f1 (ppm)

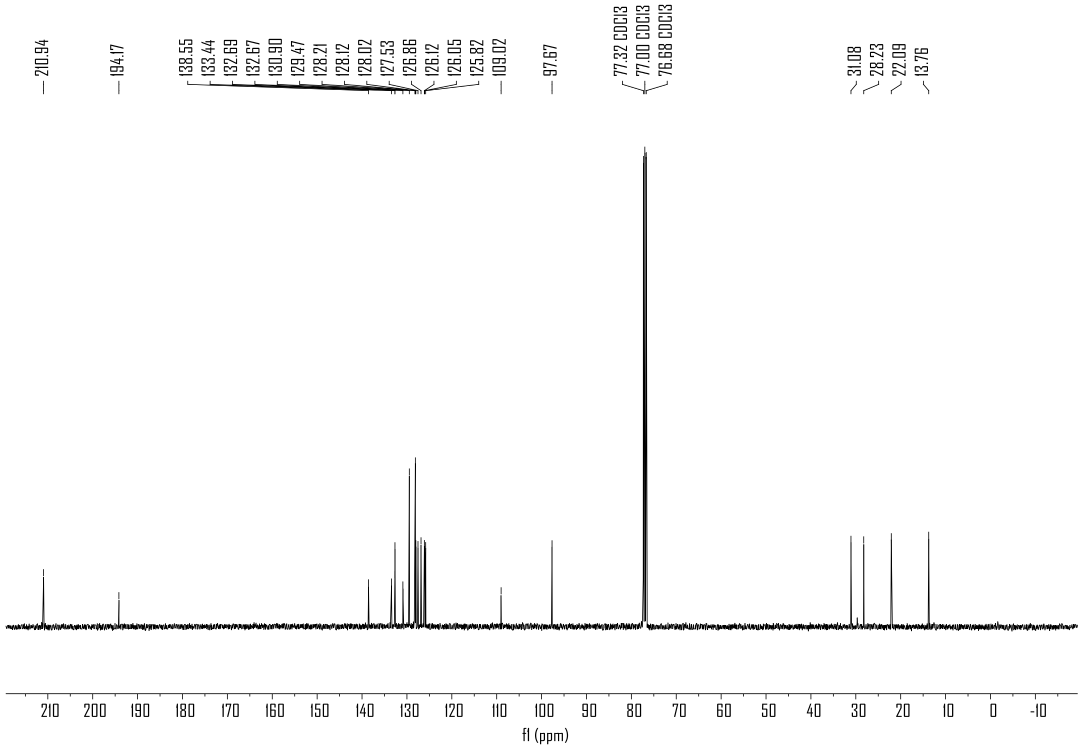
210.94
194.17
138.55 133.44 132.69 132.67 130.90 129.47 128.21 128.12 128.02 127.53 126.86 126.12 126.05 125.82
109.02
97.67
77.32 CDCl3
77.00 CDCl3
76.68 CDCl3
31.08
28.23
22.09
13.76
210 200 190 180 170 160 150 140 130 120 110 100 90 80 70 60 50 40 30 20 10 0 -10
f1 (ppm)

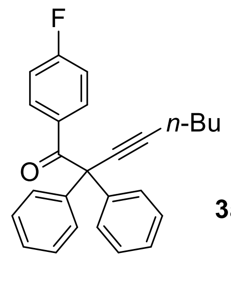
F
O
n-Bu

3a-1

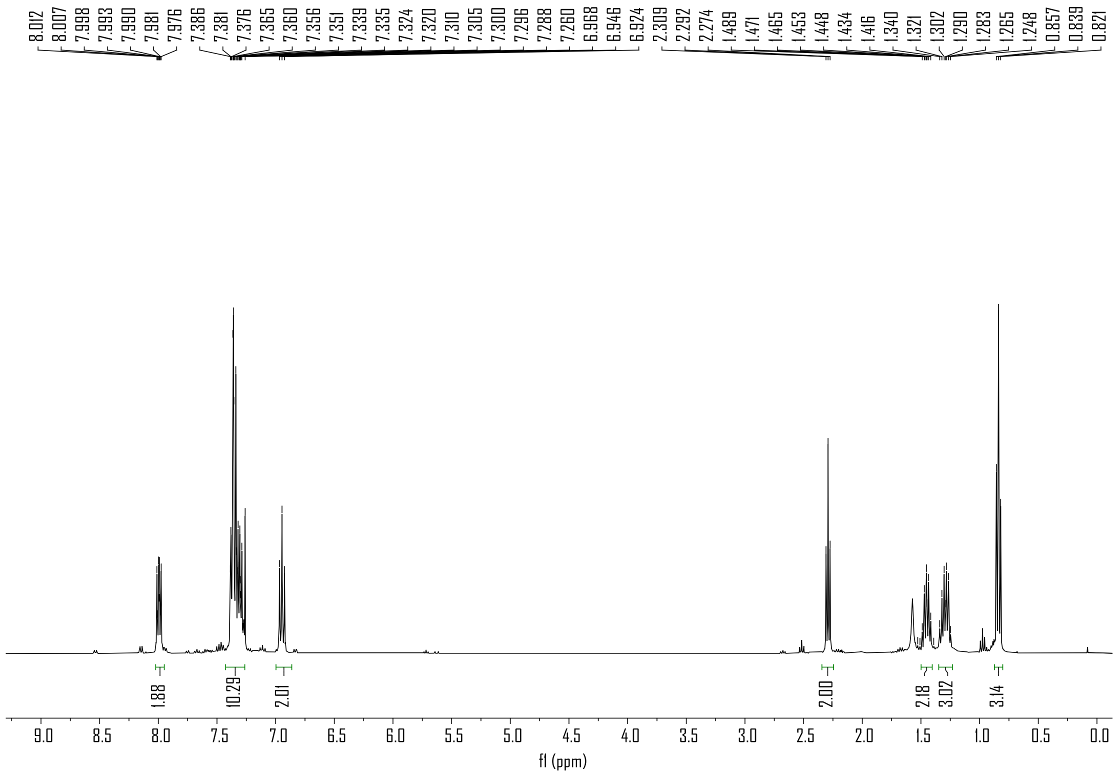

8.012
8.007
7.998
7.993
7.990
7.981
7.976
7.386
7.381
7.376
7.365
7.360
7.356
7.351
7.339
7.335
7.324
7.320
7.310
7.305
7.300
7.296
7.288
7.260
6.968
6.946
6.924
2.309
2.292
2.274
1.489
1.471
1.465
1.453
1.448
1.434
1.416
1.340
1.321
1.302
1.290
1.283
1.265
1.248
0.857
0.839
0.821
1.88
10.29
2.01
2.00
2.18
3.02
3.14
9.0 8.5 8.0 7.5 7.0 6.5 6.0 5.5 5.0 4.5 4.0 3.5 3.0 2.5 2.0 1.5 1.0 0.5 0.0
f1 (ppm)

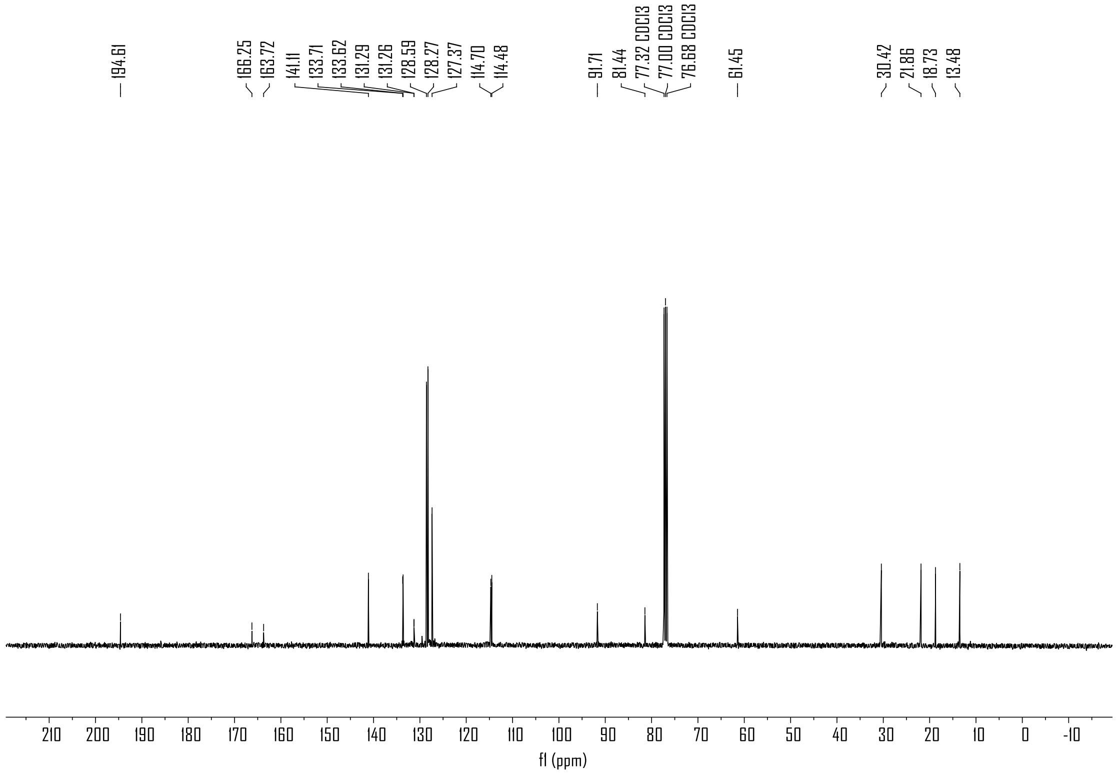

194.61
166.25
163.72
141.11
133.71
133.62
131.29
131.26
128.59
128.27
127.37
114.70
114.48
91.71
81.44
77.32 CDCl3
77.00 CDCl3
76.68 CDCl3
61.45
30.42
21.86
18.73
13.48
210 200 190 180 170 160 150 140 130 120 110 100 90 80 70 60 50 40 30 20 10 0 -10
f1 (ppm)

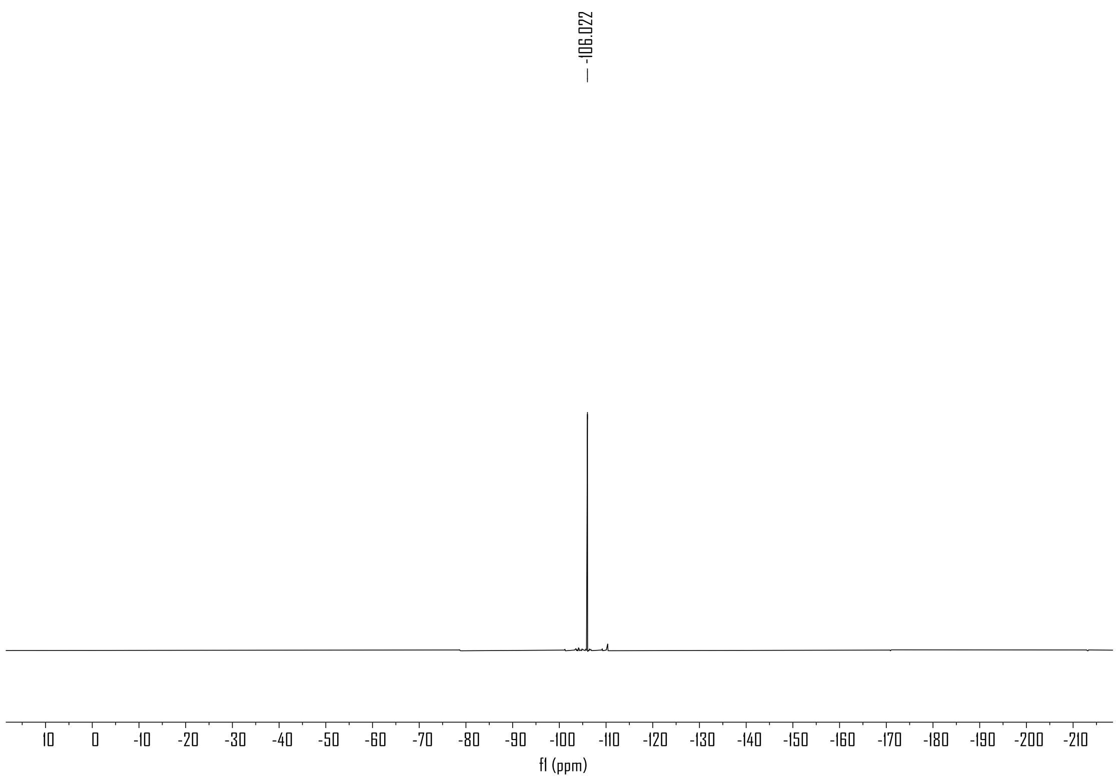
-106.022
10 0 -10 -20 -30 -40 -50 -60 -70 -80 -90 -100 -110 -120 -130 -140 -150 -160 -170 -180 -190 -200 -210
f1 (ppm)

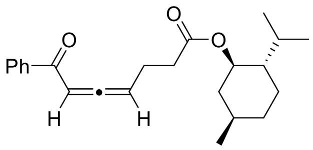
Ph
O
O
O
H
H

1aq

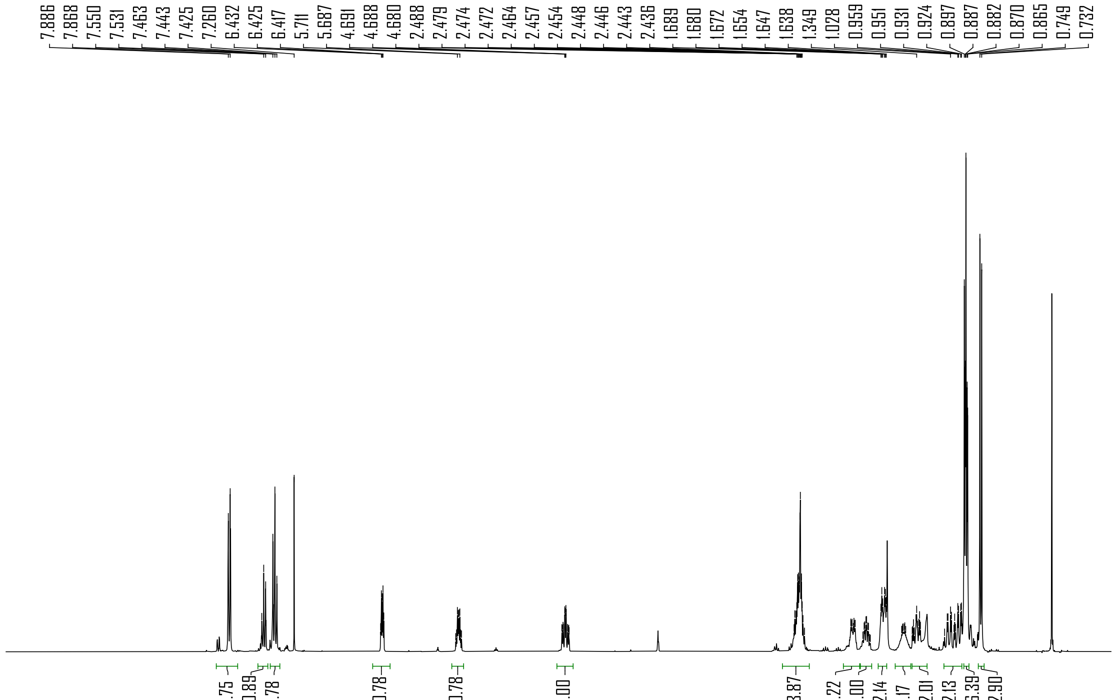
7.886
7.868
7.550
7.531
7.463
7.443
7.425
7.260
6.432
6.425
6.417
5.711
5.687
4.691
4.688
4.680
2.488
2.479
2.474
2.472
2.464
2.457
2.454
2.448
2.446
2.443
2.436
1.689
1.680
1.672
1.654
1.647
1.638
1.349
1.028
0.959
0.951
0.931
0.924
0.897
0.887
0.882
0.870
0.865
0.749
0.732
1.75
0.89
1.78
0.78
0.78
1.00
3.87
1.22
1.00
2.14
1.17
2.01
2.13
6.39
2.90
9.5 9.0 8.5 8.0 7.5 7.0 6.5 6.0 5.5 5.0 4.5 4.0 3.5 3.0 2.5 2.0 1.5 1.0 0.5 0.0
f1 (ppm)

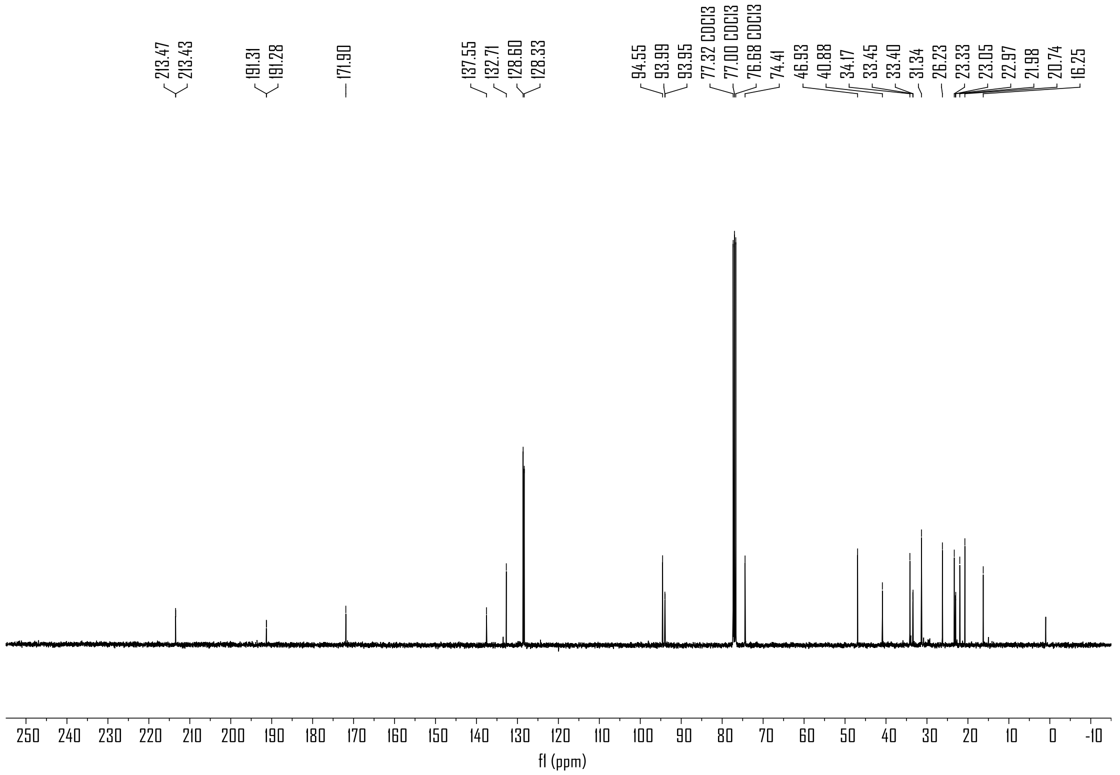
213.47
213.43
191.31
191.28
171.90
137.55
132.71
128.60
128.33
94.55
93.99
93.95
77.32 CDCl3
77.00 CDCl3
76.68 CDCl3
74.41
46.93
40.88
34.17
33.45
33.40
31.34
26.23
23.33
23.05
22.97
21.98
20.74
16.25
f1 (ppm)

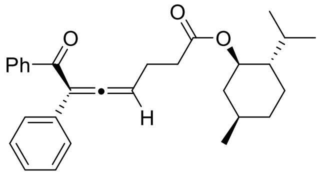
Ph
O
O
O
H

3aq

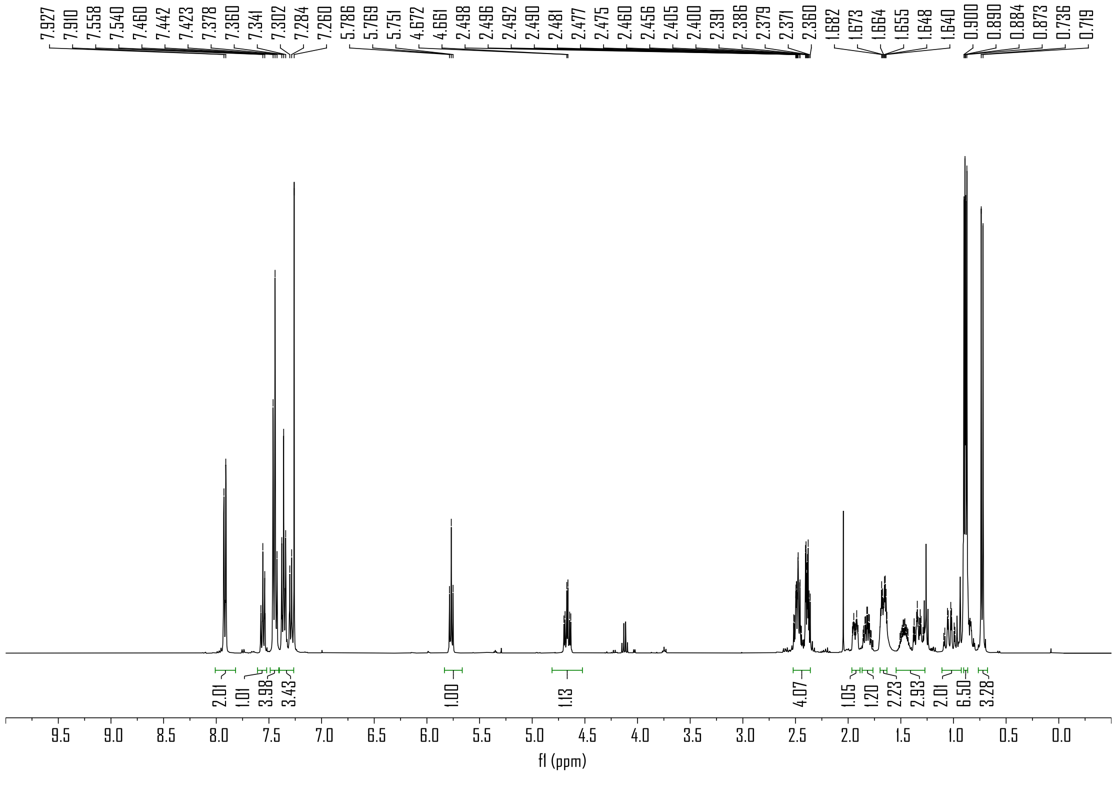
7.927
7.910
7.558
7.540
7.460
7.442
7.423
7.378
7.360
7.341
7.302
7.284
7.260
5.786
5.769
5.751
4.672
4.661
2.498
2.496
2.492
2.490
2.481
2.477
2.475
2.460
2.456
2.405
2.400
2.391
2.386
2.379
2.371
2.360
1.682
1.673
1.664
1.655
1.648
1.640
0.900
0.890
0.884
0.873
0.736
0.719
2.01
1.01
3.98
3.43
1.00
1.13
4.07
1.05
1.20
2.23
2.93
2.01
6.50
3.28
f1 (ppm)

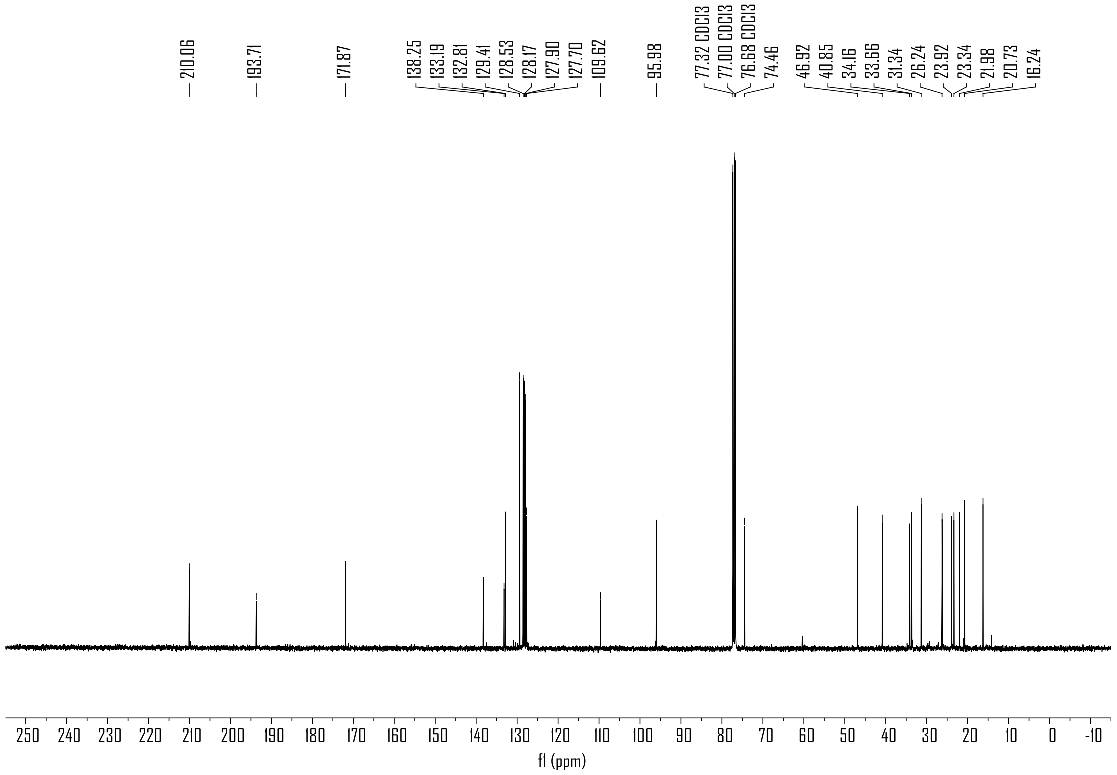
210.06
193.71
171.87
138.25
133.19
132.81
129.41
128.53
128.17
127.90
127.70
109.62
95.98
77.32 CDCl3
77.00 CDCl3
76.68 CDCl3
74.46
46.92
40.85
34.16
33.66
31.34
26.24
23.92
23.34
21.98
20.73
16.24
250 240 230 220 210 200 190 180 170 160 150 140 130 120 110 100 90 80 70 60 50 40 30 20 10 0 -10
f1 (ppm)

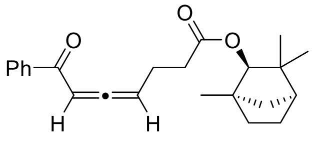
Ph
O
O
O
H
H

1ar

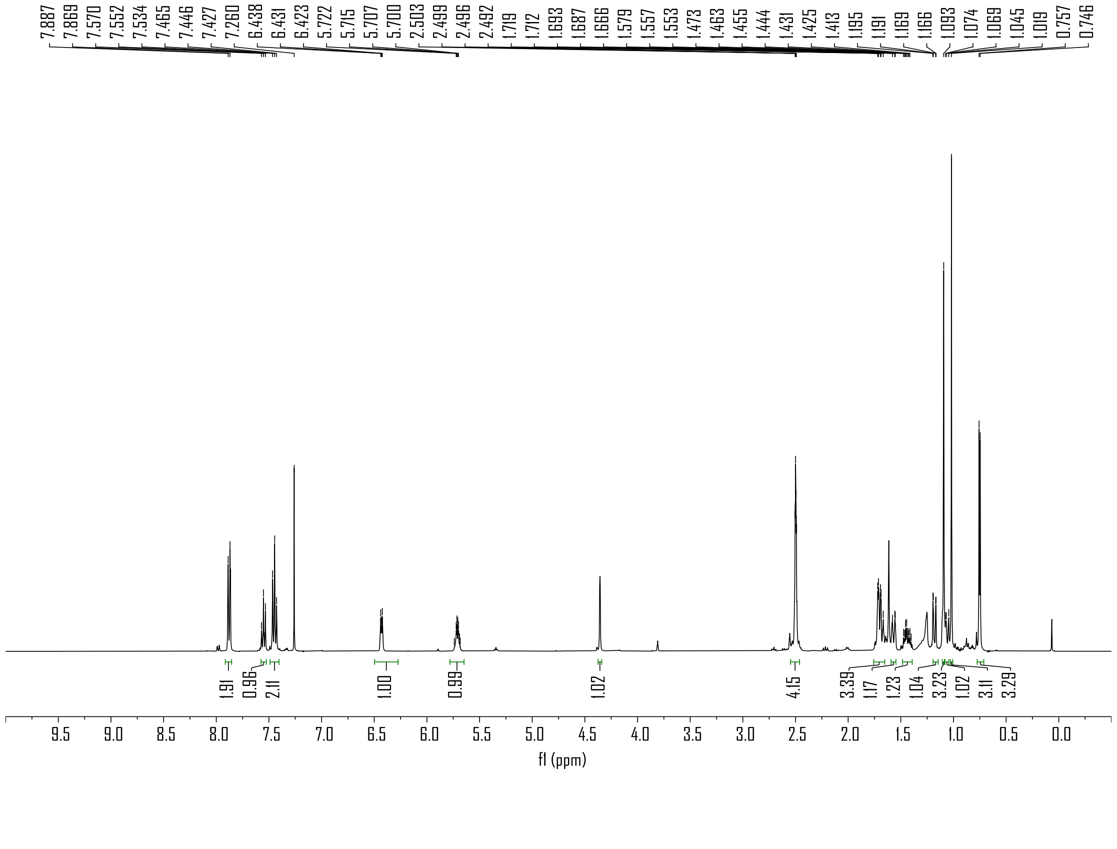
7.887
7.869
7.570
7.552
7.534
7.465
7.446
7.427
7.260
6.438
6.431
6.423
5.722
5.715
5.707
5.700
2.503
2.499
2.496
2.492
1.719
1.712
1.693
1.687
1.666
1.579
1.557
1.553
1.473
1.463
1.455
1.444
1.431
1.425
1.413
1.195
1.191
1.169
1.166
1.093
1.074
1.069
1.045
1.019
0.757
0.746
1.91
0.96
2.11
1.00
0.99
1.02
4.15
3.39
1.17
1.23
1.04
3.23
1.02
3.11
3.29
9.5 9.0 8.5 8.0 7.5 7.0 6.5 6.0 5.5 5.0 4.5 4.0 3.5 3.0 2.5 2.0 1.5 1.0 0.5 0.0
f1 (ppm)

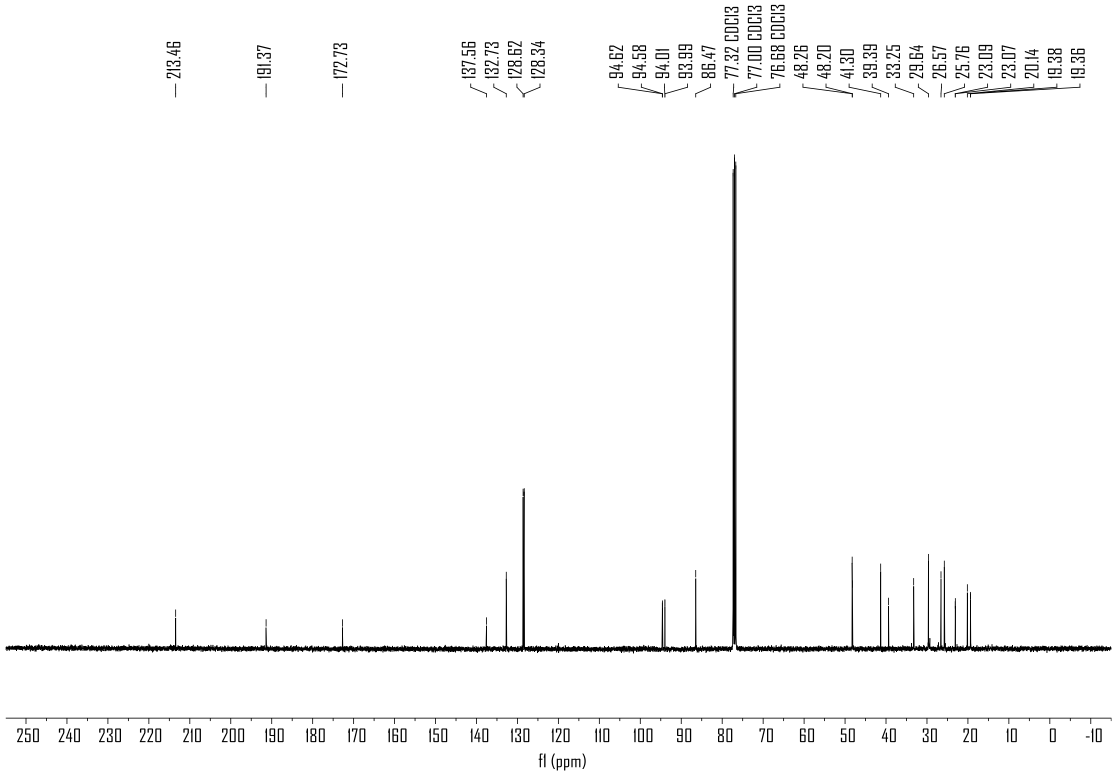
213.46
191.37
172.73
137.56
132.73
128.62
128.34
94.62
94.58
94.01
93.99
86.47
77.32 CDCl3
77.00 CDCl3
76.68 CDCl3
48.26
48.20
41.30
39.39
33.25
29.64
26.57
25.76
23.09
23.07
20.14
19.38
19.36
f1 (ppm)

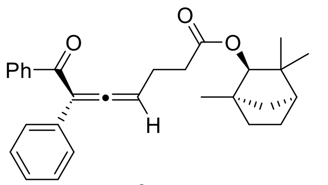
Ph
O
O
O
H

3ar

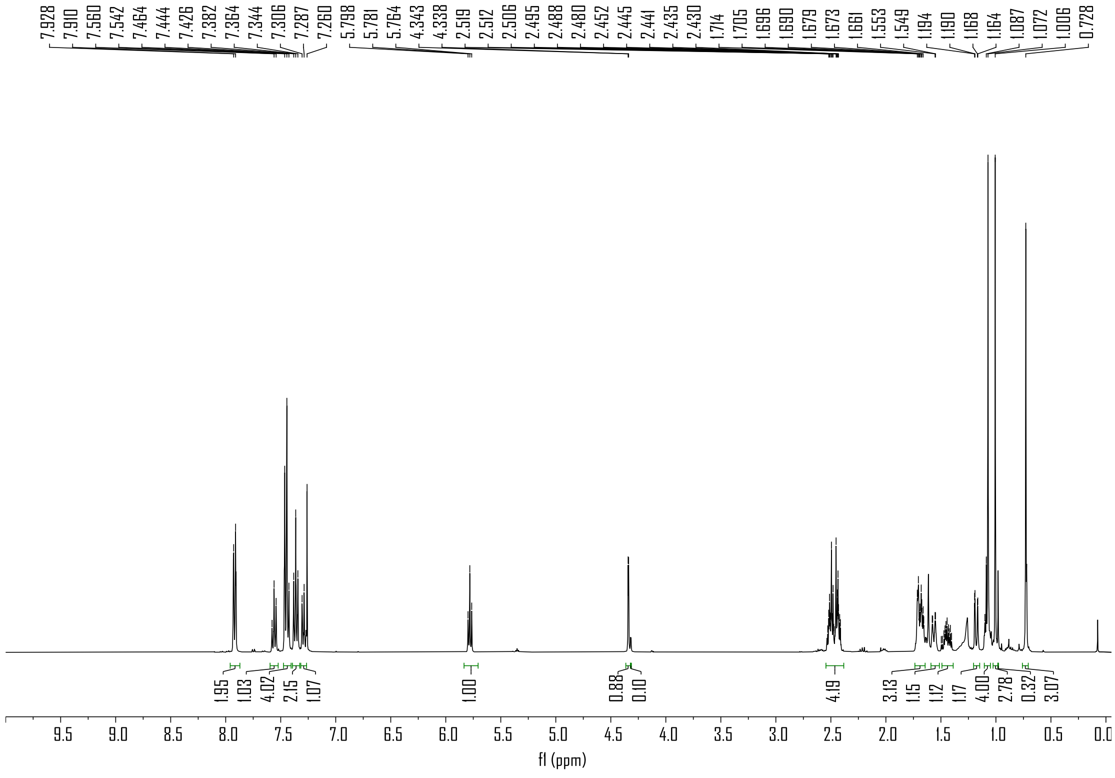
7.928
7.910
7.560
7.542
7.464
7.444
7.426
7.382
7.364
7.344
7.306
7.287
7.260
5.798
5.781
5.764
4.343
4.338
2.519
2.512
2.506
2.495
2.488
2.480
2.452
2.445
2.441
2.435
2.430
1.714
1.705
1.696
1.690
1.679
1.673
1.661
1.553
1.549
1.194
1.190
1.168
1.164
1.087
1.072
1.006
0.728
1.95
1.03
4.02
2.15
1.07
1.00
0.88
0.10
4.19
3.13
1.15
1.12
1.17
4.00
2.78
0.32
3.07
f1 (ppm)

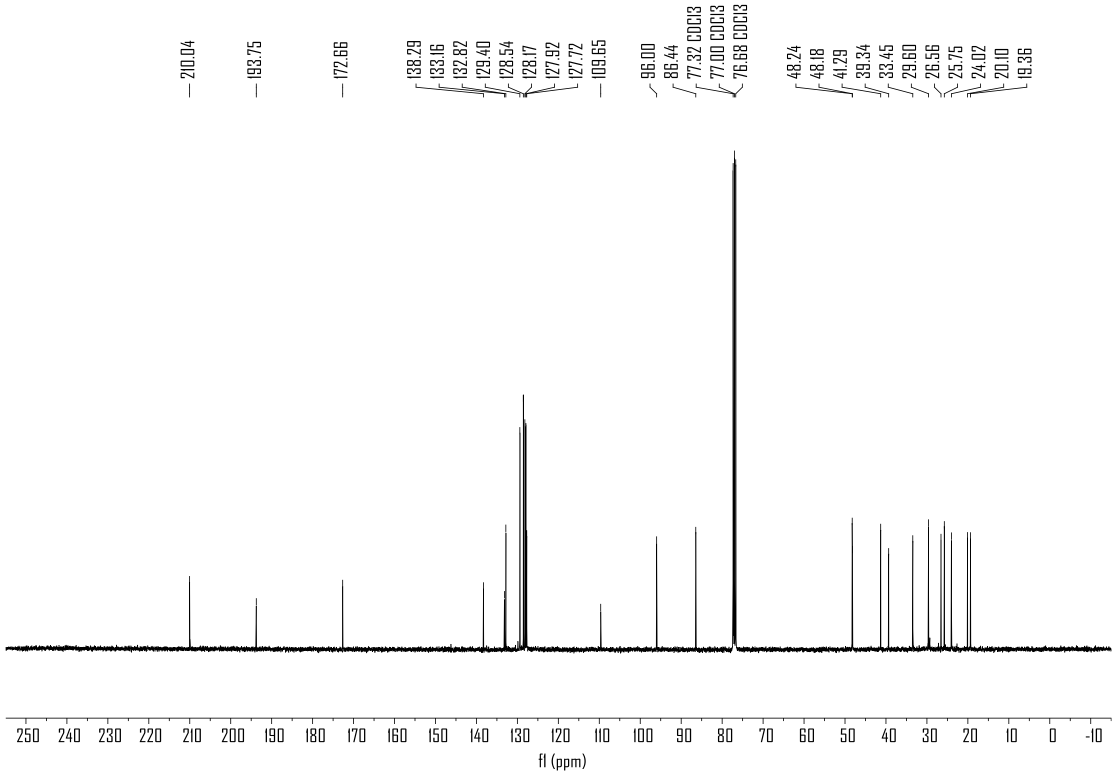
210.04
193.75
172.66
138.29
133.16
132.82
129.40
128.54
128.17
127.92
127.72
109.65
96.00
86.44
77.32 CDCl3
77.00 CDCl3
76.68 CDCl3
48.24
48.18
41.29
39.34
33.45
29.60
26.56
25.75
24.02
20.10
19.36
250 240 230 220 210 200 190 180 170 160 150 140 130 120 110 100 90 80 70 60 50 40 30 20 10 0 -10
f1 (ppm)

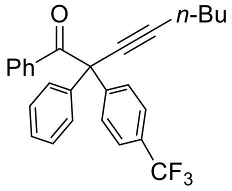
O
n-Bu
Ph
CF3

4a

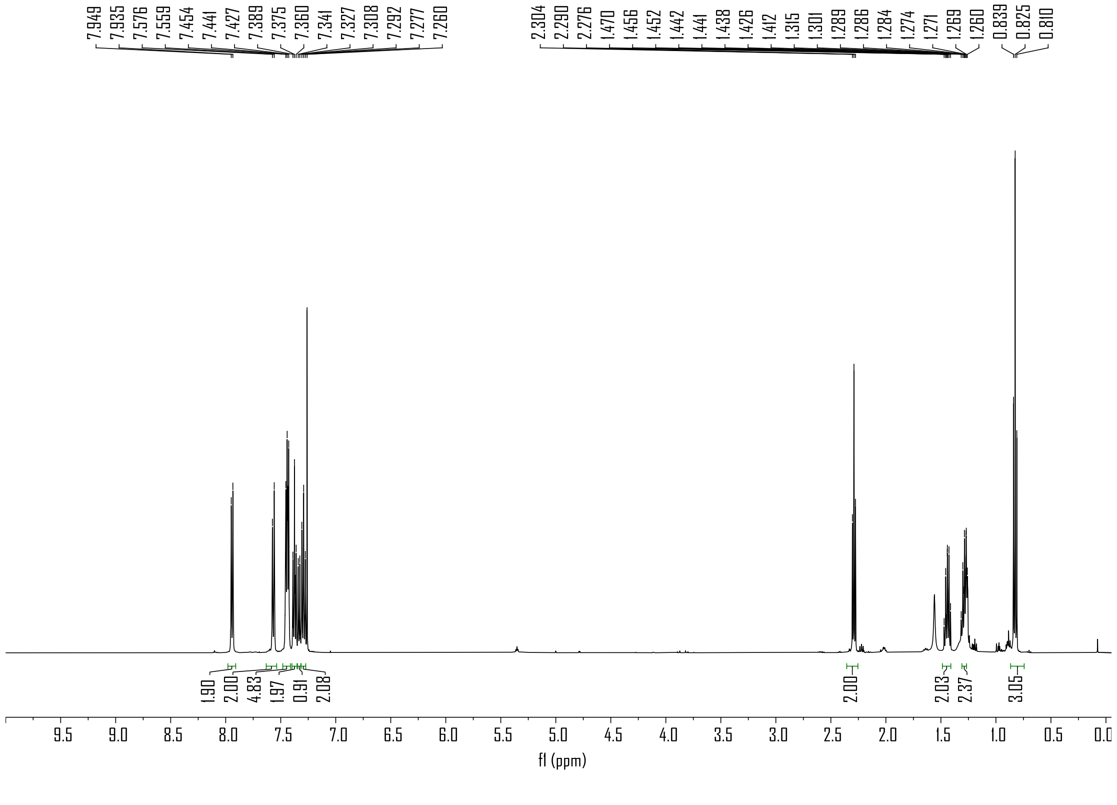
7.949
7.935
7.576
7.559
7.454
7.441
7.427
7.389
7.375
7.360
7.341
7.327
7.308
7.292
7.277
7.260
2.304
2.290
2.276
1.470
1.456
1.452
1.442
1.441
1.438
1.426
1.412
1.315
1.301
1.289
1.286
1.284
1.274
1.271
1.269
1.260
0.839
0.825
0.810
1.90
2.00
4.83
1.97
0.91
2.08
2.00
2.03
2.37
3.05
9.5 9.0 8.5 8.0 7.5 7.0 6.5 6.0 5.5 5.0 4.5 4.0 3.5 3.0 2.5 2.0 1.5 1.0 0.5 0.0
f1 (ppm)

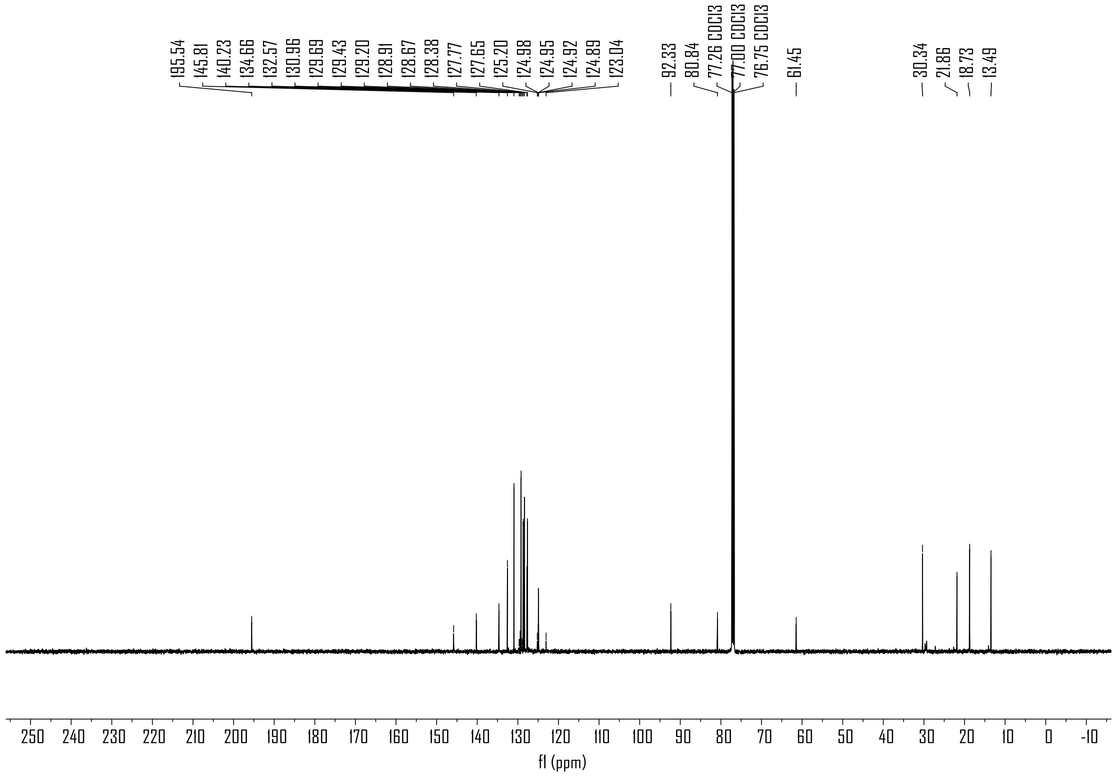
195.54
145.81
140.23
134.66
132.57
130.96
129.69
129.43
129.20
128.91
128.67
128.38
127.77
127.65
125.20
124.98
124.95
124.92
124.89
123.04
92.33
80.84
77.26 CDCl3
77.00 CDCl3
76.75 CDCl3
61.45
30.34
21.86
18.73
13.49
250 240 230 220 210 200 190 180 170 160 150 140 130 120 110 100 90 80 70 60 50 40 30 20 10 0 -10
f1 (ppm)

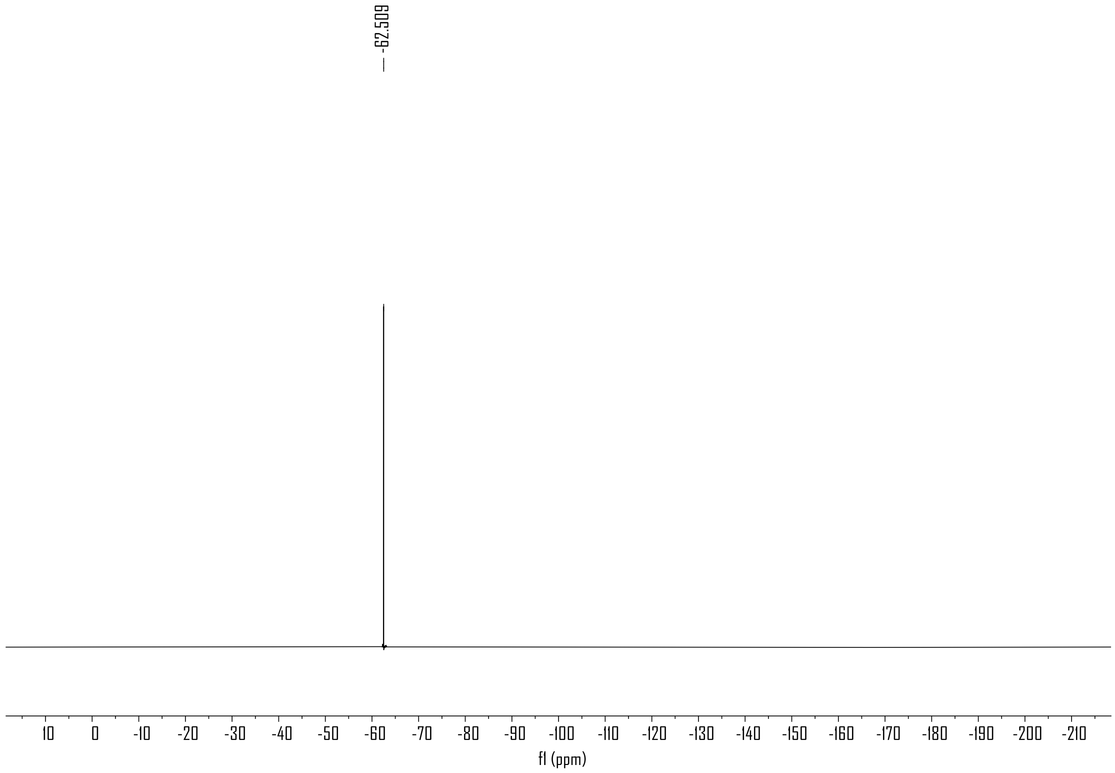
-62.509
10 0 -10 -20 -30 -40 -50 -60 -70 -80 -90 -100 -110 -120 -130 -140 -150 -160 -170 -180 -190 -200 -210
f1 (ppm)

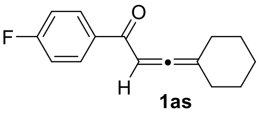
F
O
H

1as

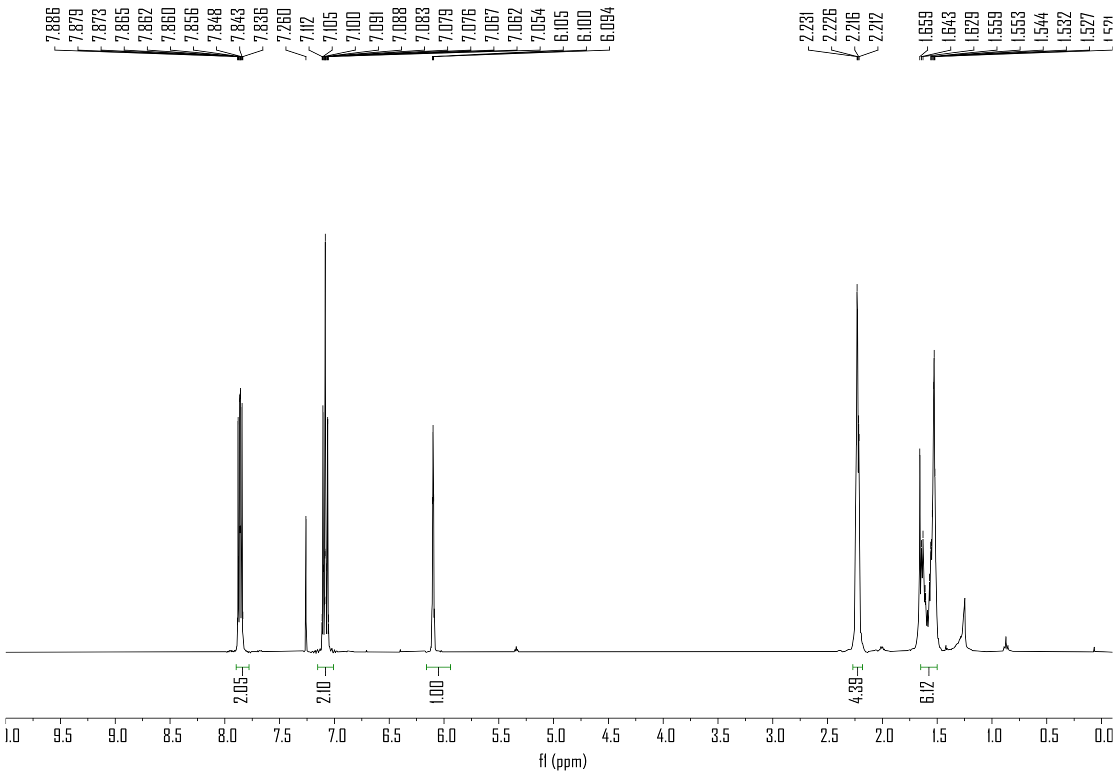
7.886
7.879
7.873
7.865
7.862
7.860
7.856
7.848
7.843
7.836
7.260
7.112
7.105
7.100
7.091
7.088
7.083
7.079
7.076
7.067
7.062
7.054
6.105
6.100
6.094
2.231
2.226
2.216
2.212
1.659
1.643
1.629
1.559
1.553
1.544
1.532
1.527
2.05
2.10
1.00
4.39
6.12
f1 (ppm)

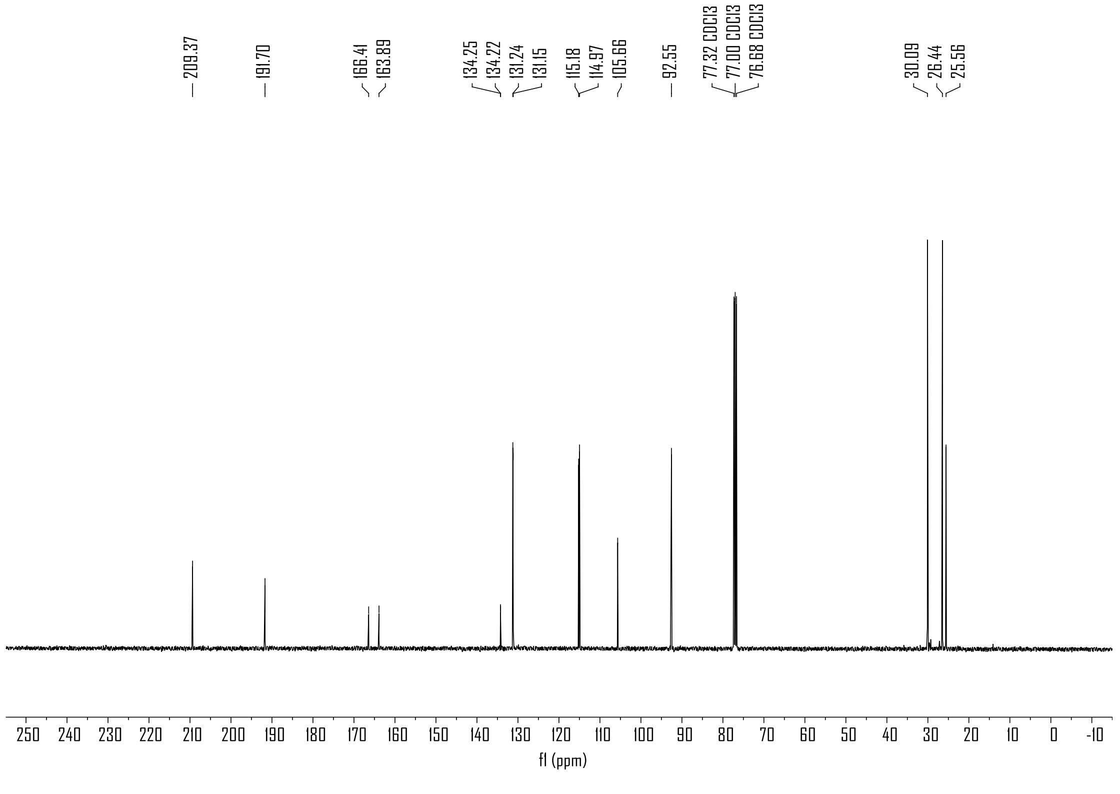
209.37
191.70
166.41
163.89
134.25
134.22
131.24
131.15
115.18
114.97
105.66
92.55
77.32 CDCl3
77.00 CDCl3
76.68 CDCl3
30.09
26.44
25.56
f1 (ppm)

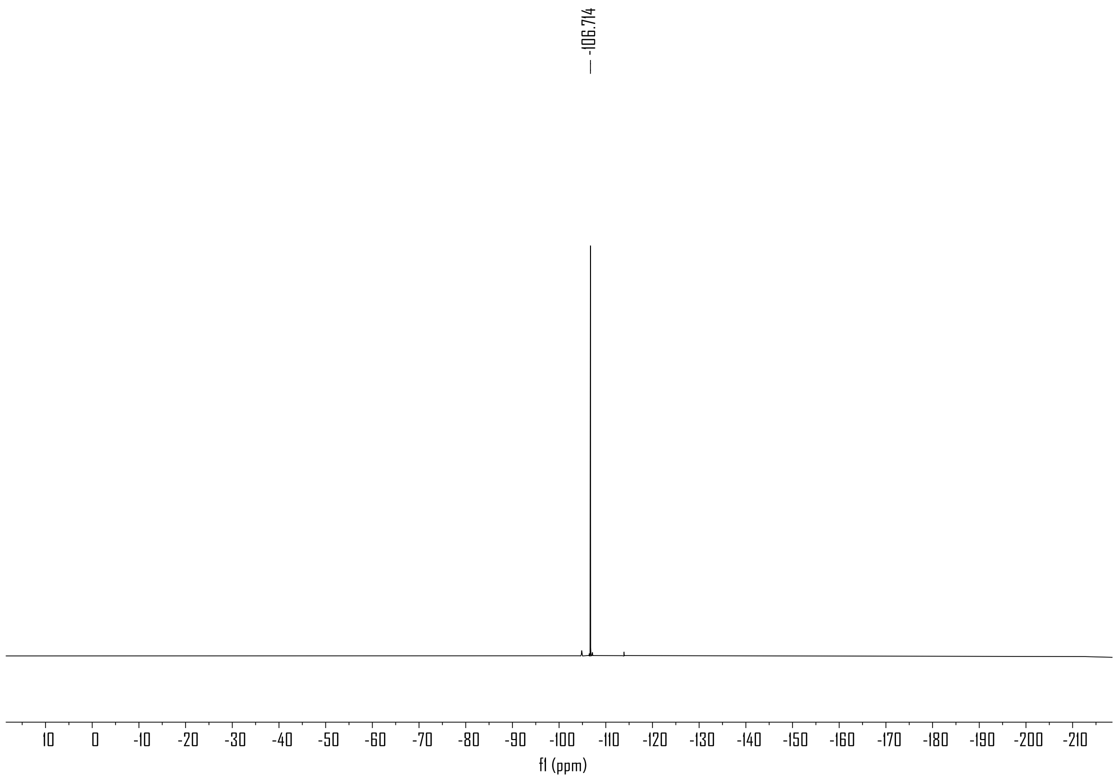

12. References

[1] H. Y. Kim, J. Y. Li, K. Oh, "Studies on Elimination Pathways of *β*-Halovinyl Ketones Leading to Allenyl and Propargyl Ketones and Furans under the Action of Mild Bases" *J. Org. Chem.* **2012**, *77*, 11132-11145.

[2] J. Son, T. W. Reidl, K. H. Kim, D. J. Wink, L. L. Anderson, "Generation and Rearrangement of *N*,*O*-dialkenylhydroxylamines for the Synthesis of 2-Aminotetrahydrofurans" *Angew. Chem. Int. Ed.* **2018**, *57*, 6597–6600.

[3] R. Hou, Z. Wang, J. Peng, Y. Yuan, J. Zhang, D. Wang, T. Sun, "Conformational Preferences of Allene Ketones in Lewis Base Catalysis: Synthesis of 4*H*-pyrans and 3,4-dihydro-2*H*-pyrans via α-Regioselective [4+2] Annulations of γ-Substituted Allene Ketones and Activated Alkenes" *Asian J. Org. Chem.* **2021**, *10*, 3334–3342.

[4] F. Challenger, V. K. Wilson, "XXXIII.—Dicyanates and Dibenzoates of Triphenylbismuthine and Triphenylstibine" *J. Chem. Soc.* **1927**, 209–213.

[5] H. Gilman, H. L. Yale, "Some New Pentavalent Salts of Triarylbismuth Compounds" *J. Am. Chem. Soc.* **1951**, *73*, 4470–4471.

[6] D. H. R. Barton, N. Y. Bhatnagar, J. -C. Blazejewski, B. Charpiot, J. -P. Finet, D. J. Lester, W. B. Motherwell, M. T. B. Papoula, S. P. Stanforth, "Pentavalent Organobismuth Reagents. Part 2. the Phenylation of Phenols" *J. Chem. Soc. Perkin Trans. 1* **1985**, 2657-2665.

[7] D. H. R. Barton, D. M. X. Donnelly, J-P. Finet, P. H. Stenson, "The Chemistry of Pentavalent Organobismuth Reagents: Part XII. Synthesis of Isoflavanones and 3-aryl-4-hydroxycoumarins" *Tetrahedron* **1988**, *40*, 6387-6396.

[8] H. Szuki, T. Ikegami, Y. Matanoa, N. A.zuma, "Unexpected Formation of Triarylbismuth Diformates in the Oxidation of Triarylbismuthines with Ozone at Low Temperatures" *J. Chem. Soc., Perkin Trans. 1* **1993**, *20*, 2411–2415.

[9] A. F. M. M. Rahman, T. Murafuji, M. Ishibashi, Y. Miyoshi, Y. Sugihara, "Effect of π-Accepting Substituent on the Reactivity and Spectroscopic Characteristics of Triarylbismuthanes and Triarylbismuth Dihalides" *J. Organomet. Chem.* **2004**, *689*, 3395–3401.

[10] A. P. M. Robertson, N. Burford, R. McDonald, M. J. Ferguson, "Coordination Complexes of $Ph_3Sb_2^+$ and $Ph_3Bi_2^+$: Beyond Pnictonium Cations" *Angew. Chem. Int. Ed.* **2014**, *53*, 3480–3483.

[11] A. Fnaiche, B. Bueno, C. MuMullin, A. Gagnon, "On the Barton Copper-Catalyzed C3-Arylation of Indoles using Triarylbismuth Bis(trifluoroacetate) Reagents" *ChemPlusChem* **2023**, *88*, 1–12.

[12] K. K. Ghosh, A. Uttry, A. Koldemir, M. Ong, M. Gemmeren, "Direct β-C(sp^3)−H Acetoxylation of Aliphatic Carboxylic Acids" *Org. Lett.* **2019**, *21*, 7154-7157.

[13] J. Li, T. Du, G. Zhang, Y. Peng, "3-Bromooxindoles as Nucleophiles in Asymmetric Organocatalytic Mannich Reactions with *N*-Ts-imines" *Chem. Commun.* **2013**, *49*, 1330–1332.

[14] S. Meninno, T. Fuoco, C. Tedesco, A. Lattanzi, "Straightforward Enantioselective Success to γ-Butyrolactones Bearing an All-Carbon β-Quaternary Stereocenter" *Org. Lett.* **2014**, *16*, 4746–4749.

[15] J. L. Meloche, P. T. Vednor, J. B. Gianino, A. G. Oliver, B. L. Ashfeld, "Titanocene-Catalyzed Metallation of Propargylic Acetates in Homopropargyl Alcohol Synthesis" *Tetrahedron Lett.* **2014**, *55*, 5025–5028.

[16] J. Wang, F. He, X. Yang, "Asymmetric Construction of Acyclic Quaternary Stereocenters via Direct Enantioselective Additions of α-Alkynyl Ketones to Allenamides" *Nat. Commun.* **2021**, *12*, 6700.

[17] CYLview, 1.0b; C. Y. Legault, Université de Sherbrooke, 2009 (http://www.cylview.org).

[18] J. H. Brewster, "A Useful Model of Optical Activity. I. Open Chain Compounds" *J. Am. Chem. Soc.* **1959**, *81*, 5475–5483.

[19] G. Lowe, "The Absolute Configuration of Allenes" *Chem. Commun. (London)* **1965**, 411–413.
